# Unravelling Structural Dynamics, Supramolecular Behavior, and Chiroptical Properties of Enantiomerically Pure Macrocyclic Tertiary Ureas and Thioureas

**DOI:** 10.1021/acs.joc.2c02319

**Published:** 2022-12-08

**Authors:** Natalia Prusinowska, Joanna Szymkowiak, Marcin Kwit

**Affiliations:** † Faculty of Chemistry, Adam Mickiewicz University, Uniwersytetu Poznańskiego 8, 61 614 Poznan, Poland; ‡ Faculty of Science, Department of Chemistry University of British Columbia, 2036 Main Mall, Vancouver, British Columbia, Canada V6T 1Z1

## Abstract

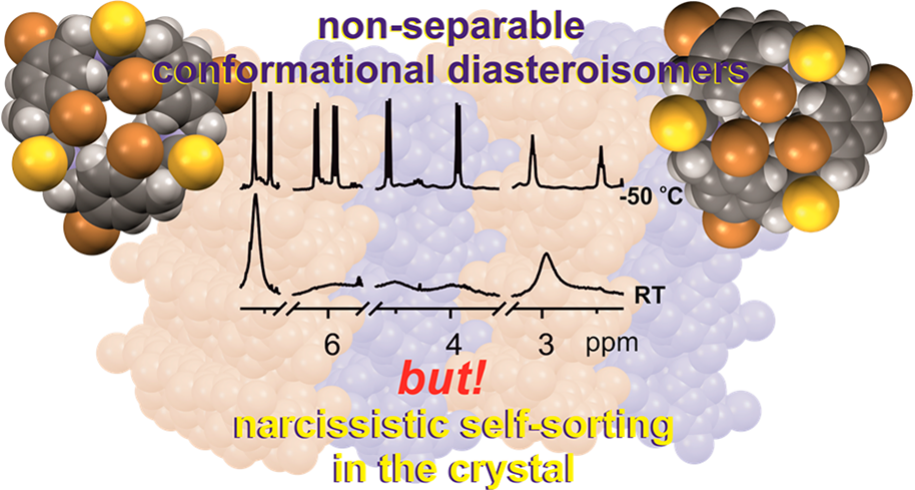

The introduction of urea or thiourea functionality to the macrocycle skeleton represents an alternative way to control conformational dynamics of chiral, polyamines of a figure-shaped periodical structure. Formally highly symmetrical, these macrocycles may adapt diverse conformations, depending on the nature of an amide linker and on a substitution pattern within the aromatic units. The type of heteroatom X in the N–C(=X)–N units present in each vertex of the macrocycle core constitutes the main factor determining the chiroptical properties. In contrast to the urea-containing derivatives, the electronic circular dichroism of thioureas is controlled by the chiral neighborhood closest to the chromophore. The dynamically induced exciton couplet is observed when the biphenyl chromophores are present in the macrocycle core. In the solid state, the seemingly disordered molecules may create ordered networks stabilized by intermolecular S···halogen, H···halogen, and S···H interactions. The presence of two bromine substituents in each aromatic unit in thiourea-derived trianglamine gives rise to a self-sorting phenomenon in the crystal. In solution, this particular macrocycle exists as a dynamic equimolar mixture of two conformational diastereoisomers, differing in the spatial (clockwise and counter clockwise) arrangement of the C–Br bonds. In the crystal lattice, macrocycles of a given handedness assemble into homohelical layers.

## Introduction

Macrocycles of diverse structures constitute one of the most important general classes of organic compounds of numerous applications in chemistry, biochemistry, and material sciences.^1−3^ Despite the current progress, the difficulties in synthesis and/or postsynthetic modifications are often recognized as the bottleneck that limits the wide use of macrocycles.^4,5^ On the other hand, wide availability and possibility of pre- and postsynthetic modifications made the chiral macro- and polycyclic polyaza compounds of periodical structure valuable synthetic targets for creating new (supra)molecular systems of assumed properties.^6−8^ Since the first successful synthesis of the basic triangular polyimine (so-called *trianglimine*), the numerous applications of these imine-derived, regular-shaped chiral macrocycles as ligands in asymmetric synthesis, molecular receptors, shift reagents in NMR spectroscopy, and light-emitting materials have confirmed the great, but still not fully used, potential of the compounds.^8−18^ On the contrary, in the case of similar in shape imide-based triangular macrocycles, the number of applications has been considered reverse to difficulties in their synthesis and purification.^19^

The presence of supramolecular synthon-forming functional groups justifies expectations associated with the use of polyaza macrocyclic and cage compounds as molecular building blocks (tectons) in molecular tectonics.^20−22^ However, one has to remember that other factors, namely, the shape-complementarity and ability to form specific host–guest complexes, may significantly affect the way of organization of the molecules in the solid state.^23^ In some cases, the poor shape complementarities lead to obtaining the supramolecular organic frameworks (SOFs), characterized by intrinsic permanent porosity.^24−29^

Only recently, Chaix,^30^ He,^18,31^ Dey,^32−34^ Liu,^35^ Ding,^36^ and co-workers have proven the ability of macrocyclic chiral polyimines to selectively capture gases or to recognize and separate small organic molecules.^37−40^ However, only little is known about the similar properties of the reduced counterparts of macrocyclic polyimines. This is apparently due to much higher structural dynamics of polyamines resulting in the adaptation of different conformations by the given macrocycle.^41^ The control over structural dynamics can be achieved by proper postsynthetic *N*-functionalization of the macrocycle, usually by *N*-alkylation of secondary amine groups or by the introduction of an aminal unit in each fragment of vicinal diamine present in the molecule.^42,43^

In the crystal phase, the triangular macrocycles having methylene bridges form channels and voids capable of entrapping small (solvent) molecules.^41,42^ The selectivity toward specific molecules is a function of the size matching between the guest and the trianglamine host’s cavity, and in certain cases, the full separation of linear alkanes from linear and branched alkanes mixture has been achieved.^36,44−46^ These results have been considered promising in terms of further industrial applications and are in line with the recently observed trend of searching for new, preferably chiral selectors of organic molecules with low molecular masses.^47−52^

However, due to the reversibility of the reaction, thus susceptibility to hydrolysis of the aminal product, this particular modification has provided macrocyclic derivatives of the usability limited to nonacidic and water-free conditions. On the contrary, irreversible bonding of two adjacent amino groups by amide and thioamide bonds is an alternative option to control the macrocycle structure and its conformational dynamics. To date, there have only been a few examples known where urea and thiourea units have been introduced to each of the basic trianglamine and isotrianglamine vertexes.^53^

Apart from the complex conformational dynamics, these compounds are characterized by contrasting propensity to the formation of metallogels with silver and copper ions. While the enantiomerically pure thiourea-derived trianglamine has appeared to be a low-molecular supergelator forming thixotropy-exhibited metallogel, other molecules under study did not show any gelling abilities. Additionally, the solid-state and solution structure of urea- and thiourea-derived trianglamines turned out to be significantly different.^53^

Bearing in mind the importance of urea and thiourea moieties in catalysis and supramolecular chemistry,^54−63^ and, on the other hand, the growing role of periodical macrocyclic systems in material chemistry, we have decided to expand the study on other representatives of trianglamine, isotrianglamine, and rhombamine families. As the further spectacular applications of this type of macrocycles in various fields of chemistry originated from basic research, this work is aimed at the structural study of the series of macrocyclic urea and thiourea derivatives by means of complementary experimental and theoretical methods. Since the compounds are chiral, we will put an emphasis on their chiroptical properties, which have not been the subject of in-depth investigations. The premise of this part of the study is an attempt to answer the question, to what extend the analysis of electronic circular dichroism spectra of compounds containing urea and thiourea chromophores, in a fixed *Z*,*Z* conformation, would provide useful structural information.^64^

## Result and Discussion

### Synthesis and Structure of **1**–**9** from NMR Measurements and DFT Calculations

**Chart 1:**
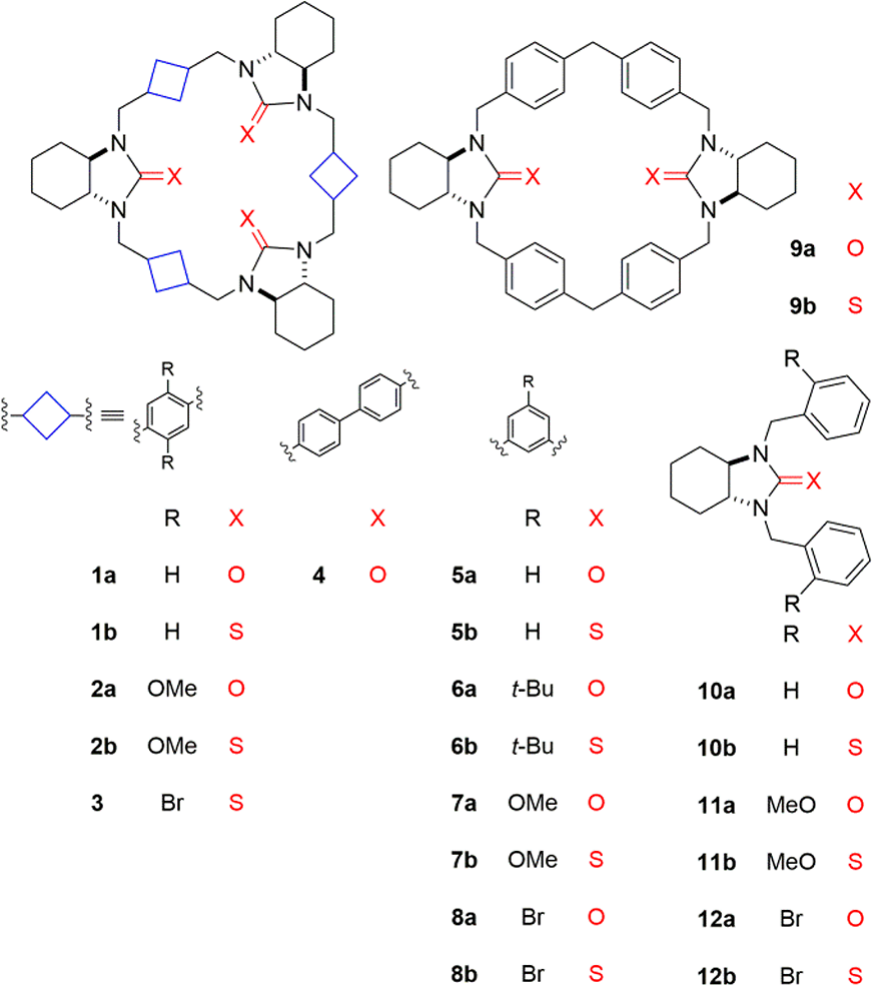
Structures of the Compounds under Study

The macrocyclic urea and thiourea derivatives **1**–**9** (Chart 1) have been obtained through a three-step synthesis, starting from optically pure *trans*-(1*R*,2*R*)-diaminocyclohexane and respective dialdehyde accordingly to the previously described procedures.^11,13,14,65−67^ After condensation, the polyimine macrocyclic products have been reduced by sodium borohydride without prior purification. The resulting crude polyamines have been subjected to the final step, which is a reaction with an excess of carbonyldiimidazole or thiocarbonyldiimidazole, respectively (1 equiv per one diaminocyclohexane unit). The yields of isolated and chromatographically purified products varied from 15 to 77%. The HR mass spectra, together with other spectroscopic techniques, have confirmed the full derivatization of the given macrocyclic amine.^68^

**Figure 1:**
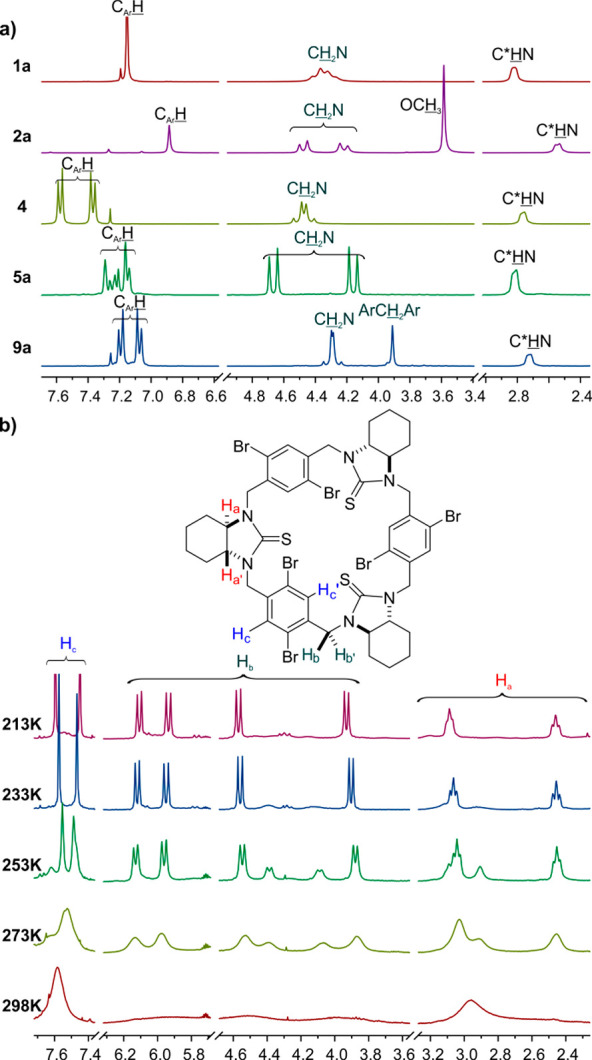
(a) Diagnostic region of ^1^H NMR spectra (600 MHz, CDCl_3_) of **1a**, **2a**, **4**, **5a**, and **9a**. (b) Parts of the temperature-dependent ^1^H NMR spectra (600 MHz, CDCl_3_) of **3**.

The intuitively assumed preference for symmetrical structures of such chiral and periodical macrocycles should, in principle, correspond to the number of signals on the NMR spectra.^53−69^ In general, the ^1^H NMR spectra of urea derivatives have shown fewer sharp signals than their thiourea counterparts. The ^1^H NMR spectra of the urea-derived trianglamines **1a**–**2a** might be considered simple. In the diagnostic region (4–8 ppm), one can distinguish three sets of signals. Two of them, visible in the region between 4 and 5 ppm, constitute doublets that come from benzylic NCH_2_(Ar) protons. The remaining third signal, which covers all aromatic protons, appears at around 7 ppm (Figure 1a).

The macrocycle **4**, having a more extended biphenyl aromatic linker, is an exception. Since the differences in chemical shifts are comparable to the ^3^*J* coupling constant, all the benzylic protons in the ^1^H NMR spectrum of **4** appear as a quartet at 4.47 ppm, whereas aromatic protons give two doublets at 7.37 and 7.57 ppm and of an equal integration (Figure 1a). Both the above-discussed ^1^H and ^13^C NMR (deposited in the Supporting Information, SI) spectra confirm symmetry of the given urea-derived trianglamine molecules.

The formal replacement of oxygen atoms by more sterically demanding sulfur atoms has significantly affected the structural preferences of the respective thioureas. The macrocycle **1b**, at room temperature, exists as a slowly interconverting mixture of a symmetrical and nonsymmetrical cone and partial-cone conformers.^53^ An introduction of substituents to the aromatic rings additionally slows the conformational changes, as revealed from temperature-dependent ^1^H NMR spectra of **3**.

In the ^1^H NMR spectrum of **3** measured at room temperature, it has been not possible to determine the exact number nor the integration of the observed fuzzy signals. However, with the lowering temperature, the spectra simplify and the stepwise appearance of sharp signals is observed. At –60 °C, in the diagnostic region of the ^1^H NMR spectrum (Figure 1b), two sets of signals appear. Each of them consists of two doublets originating from benzyl protons (H_b_) and one sharp singlet in the aromatic region of the spectrum, which covers all aromatic protons H_c_. Both sets of signals are characterized by the same integration; thus, one can assume the existence of two highly symmetrical conformational diastereoisomers (rotamers) of the given macrocycle. The number of the diagnostic signals corresponding to the *C*_3_-symmetrical cone conformers differ in the spatial (clockwise and counter clockwise) arrangement of the direction of bromine atoms (*vide infra*).^70^

More consistent results have been obtained for isotrianglamine derivatives **5**–**8**. The typical ^1^H NMR spectrum of these derivatives exhibits four signals in the diagnostic region 4–8 ppm. In the case of ureas **5a**–**8a** (see Figure 1a), two doublets characterized by large coupling constant ^3^*J* ca. 16 Hz appear at around 4.0 and 4.7 ppm, and as one could expect, they originate from diastereotopic NCH_2_Ar benzyl protons. These protons are deshielded (shift by ca. 0.5 ppm, downfield) when the thiourea moieties have been introduced to the macrocycle skeleton. In the aromatic region of the ^1^H NMR spectra of **5**–**8**, two singlets of the mutual integration 1:2 are observed. The chemical shifts of these aromatic protons are neither dependent on the kind of the linker nor on the electronic properties of the substituent at C5 position of the aromatic ring. Taking into account the above-mentioned facts, the isotrianglamine-based macrocycles can be considered to have the highest available *C*_3_ symmetry.

The rhombamine derivatives **9a** and **9b** follow the trend observed earlier for trianglamine derivatives **1**–**3**. The ^1^H NMR spectra of **9a** (diagnostic region of spectrum is shown in Figure 1a) and **9b** confirm the nontrivial symmetry of the molecules.

To gain insight into the structure of the compounds under study, we have conducted some calculations for model trianglamine derivatives **1a**, **1b**, **3**, and **4** as well as for urea and thiourea derivatives of basic isotrianglamine and rhombamine, **5a**, **5b** and **9a**, **9b**, respectively (see the SI for details).^71^ As these calculations have constituted the starting point for the subsequent calculation of chiroptical properties, the geometries were initially optimized in the gas phase and reoptimized with the use of the solvent model,^72^ if necessary.^73^ The IEFPCM model has mimicked the polar solvent used for circular dichroism measurements (acetonitrile, dichloromethane or chloroform). The calculation results are juxtaposed in Tables S1–S14, whereas structures of individual low-energy conformers of **1a**, **1b**, **3**, **4**, **5a**, **5b**, and **9a**, **9b** are shown in Figures S24–S37. It should be pointed out that the effect of the solvent on the structure and chiroptical properties of the model compounds is negligible (see Figures S38–S43). However, in the particular cases of **4** and **5a**, the solvent effect has affected the energetic relationships between conformers.

**Figure 2:**
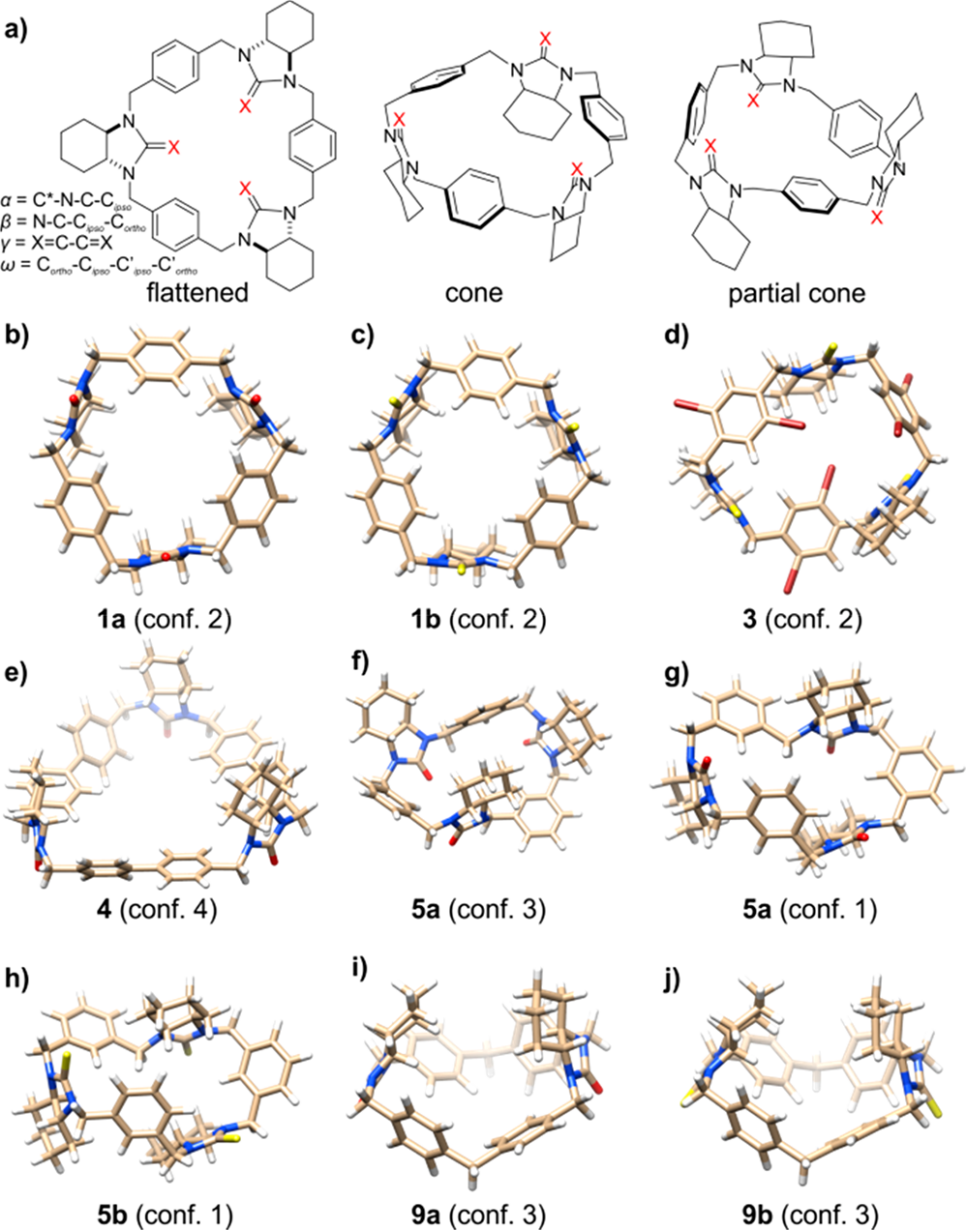
(a) General structure of the main conformational types of urea and thiourea derivatives of polyamine macrocycles with definition of torsion and pseudotorsion angles α, β, γ, and ω. Calculated at the B3LYP/6-311G(d,p) level structures of the lowest energy conformers of macrocycles: (b) **1a**; (c) **1b**; (d) **3**; (e) **4**; (f) **5a** (calculated in gas phase); (g) **5a** (calculated with the use of acetonitrile solvent model); (h) **5b**; (i) **9a**; and (j) **9b**.

To be consistent with the initial study, the structure of each of the macrocycles can be characterized by sets of torsion angles β, α, α′, β′, and γ.^53^ The angles α (defined as C*–N–C–C_*ipso*_) and β (defined here as N–C–C_*ipso*_–C_*ortho*_(X)) describe the “local” conformation around each diaminocyclohexane unit. On the contrary, the pseudotorsion angles γ (X=C···C=X) can be considered as a “global” property, as these angles describe spatial orientation of the C=X (X = O or S) bonds. An analysis of the mutual orientation of the C=X (X = O or S) bonds gives the premise for a proper classification of the macrocycle conformation (see Figure 2a). All the structural data are juxtaposed in Table S15 in the SI.

In the case of the trianglamine derivatives, the highest available *D*_3_ symmetry is restricted to the flattened conformer. In this particular structure, all of the C=X units lie on the same plane; therefore, the pseudotorsion angles γ equal 0°. However, due to the significant electrostatic and steric repulsions, which cause deformation of the valence angles and, thus, increase the energy of the molecule, none of the trianglamine derivatives will adapt such a particular conformation. Reducing the symmetry of a given molecule is associated with the release of steric strains and adapting a vaselike (cone) conformation, characterized by lower *C*_3_ symmetry and parallel orientation of each C=X bond (the values of pseudotorsion γ angles oscillate around 0°). In the lowest energy and *C*_3_-symmetrical conformers of both **1a** and **1b** (Figure 2b,c), the sequences of torsion angles β, α, α′, and β′ have been estimated as *A*^+^, *G*^+^, *G*^–^, *G*^–^. In the *C*_1_-symmetrical partial cone conformers, one of the C=X bonds is oriented antiparallelly to the remaining two and the torsion angles β, α, α′, and β′ adapt *A*^+^, *A*^–^, *A*^+^, *G*^+^; *G*^+^, *A*^+^, *G*^–^, *A*^–^; *G*^+^, *A*^+^, *G*^–^, *G*^–^ sequences (see Table S15).

The lowest energy conformer no. 2 of **3**, adapts a partial cone conformation, stabilized by a set of weak hydrogen–heteroatom interactions. The *C*_3_-symmetrical cone conformer no. 1 is slightly higher in energy (*ΔΔG* = 0.4 kcal mol^–1^), and the remaining partial cone conformer no. 3 and (quasi)cone conformer no. 4 of **3** are found to be nonsymmetrical. The bromine atoms might be oriented either clockwise or counterclockwise, or one of the aromatic rings might adapt the opposite conformation to the other two aromatic rings. In conformer nos. 1, 2, and 3 of **3**, the lower macrocyclic rim is characterized by a counter clockwise arrangement of bromine atoms, whereas in conformer no. 4, the local symmetry is disturbed by one of the aromatic rings, which adapts conformation opposite to the remaining two.

All of the calculated low-energy structures of isotrianglamine derivatives **5a** and **5b** are characterized by the trivial *C*_1_ symmetry (Figure 2f–h). This is apparently due to the 1,3-substitution pattern in aromatic rings, which allows the ring to be bent to maximize the attractive C=X···H and C–H···π interactions. Taking into account the relative orientation of C=X bonds as the element determining and distinguishing the conformer type, one can say that the dominant conformers of **5a** and **5b** are those of the partial cone type. It is worth noting that in the case of **5a** the polar environment (acetonitrile solvent model) affects the energetic relationships between stable conformers. In the gas phase, conformer no. 3 of **5a** is dominated and its relative *ΔΔ*G energy is lower by about 0.7 kcal mol^–1^ than of the second in a row conformer no. 1 of **5a**. However, when the calculations were repeated with the use of acetonitrile solvent model, both conformer nos. 1 and 3 turned out almost equal in energy (the calculated difference is 0.04 kcal mol^–1^ in favor of conformer no. 1).

In the above-discussed compounds, the conformational freedom is mostly restricted to the up and down flipping of (tio)urea moiety and to the (hampered) rotation of the aromatic linker. However, when the linker consists of two aromatic parts, as in **4** and **9**, the molecule gains additional degrees of freedom associated with the rotation of aromatic rings. Due to the size of the macrocycle cavity, each side of the macrocycle might adjust its conformation independently to others. The introduction of an angle ω (defined as C_*ortho*_–C_*ipso*_–C_*ipso*_–C_*ortho*_) allows for a convenient description of the aromatic linker conformation and, in fact, defines *P* or *M* helicity of the given moiety. The latter structural feature might be further correlated with chiroptical properties of a molecule.

The lowest energy and *P* helical conformer no. 4 of **4** (shown in Figure 2e) is characterized by the sequences of torsion angles β, α, α′, β′ estimated as *A*^–^, *G*^–^, *A*^+^, *G*^+^, and by parallel orientation of each C=O bonds (the torsion γ angles adapt *S*^+^ conformation). In conformer no. 1, that is slightly higher in energy (by 0.02 kcal mol^–1^), the sequence of torsion angles β, α, α′, β′ remains the same as in conformer no. 4; however, the torsion γ angles adopt the *S*^–^ conformation. More importantly, the helicity of the biphenyl linkers is opposite. The third, low-energy partial cone conformer no. 6 has only a 5% share in the conformational equilibrium and is characterized by *MMP* helicity of biphenyl moieties. The remaining stable cone and partial cone conformers are characterized by higher energies and thus do not participate in conformational equilibrium.

The presence of the polar environment (acetonitrile) has not significantly affected the structure of a given conformer. However, in such conditions, homohelical conformer no. 4 (over 80% of abundance) turns out to be the most abundant one followed by higher in energy cone *MMP* conformer no. 3 and partial cone conformers no. 6 and 7, characterized by *MMP* and *PPP* helicity, respectively.

The case of rhombamine derivatives **9** is very consistent (the lowest energy structures are shown in Figure 2i,j). Among the four stable conformers found by calculations, there are only two structures, namely cone and partial cone conformers no. 3 and 4, respectively, which fall into the 2 kcal mol^–1^ energy window. Regardless of the polarity of the heteroatom, both conformers are *C*_2_-symmetrical and characterized by the same *P* helicity of the aromatic linker, although the exact values of the ω angles are different (ca. 30° for conformers no. 3 and ca. 80° for conformers no. 4). For cone conformers, the sequences of the torsion angles β, α, α′, β′ are estimated as *A*^–^, *G*^–^, *A*^+^, *G*^+^, whereas the partial cone conformers are characterized by *A*^+^, *A*^+^, *G*^–^, *A*^–^ sequence of β, α, α′, β′ angles. Despite their more compact “bowl-shaped” structure, cone conformers dominate in conformational equilibrium and the energy preference for such a structure is more visible in the polar environment mimicked by the solvent model. In the extreme case of **9b**, the cone conformer no. 3 represents the only entity thermally available in dichloromethane.

The discrepancy observed for some cases between experimental NMR and theoretical DFT results concerning the preferred structure of a given macrocycle can be explained as a different approach to the problem. One has to remember that in the time-scale of standard NMR measurements, the “average” structure of a given compound is observed. The theoretical calculations allow for taking selected “snapshots” from the whole conformational space. Additionally, even the most perfect theoretical method will provide results with some errors. The error in energy, estimated for DFT calculations, is ca. 1.5 kcal mol^–1^,^74^ and in the cases discussed here, the stabilized electrostatic interactions seem to be overestimated.

### Chiroptical Properties of Urea and Thiourea-Derived Macrocycles **1**–**9**

The electronic circular dichroism (ECD) spectra of macrocyclic derivatives **1**–**9** have been measured in solvents of contrasting polarity–nonpolar cyclohexane and highly polar acetonitrile. In selected cases, where solubility in these solvents was so limited that it was not possible to record the ECD spectra, dichloromethane was used.

The ECD spectra of **1a**–**9a** (see examples shown in Figure 3 and remaining spectra in Figures S2–S23) are characterized by a relatively limited number of non-zero Cotton effects (CEs).

**Figure 3:**
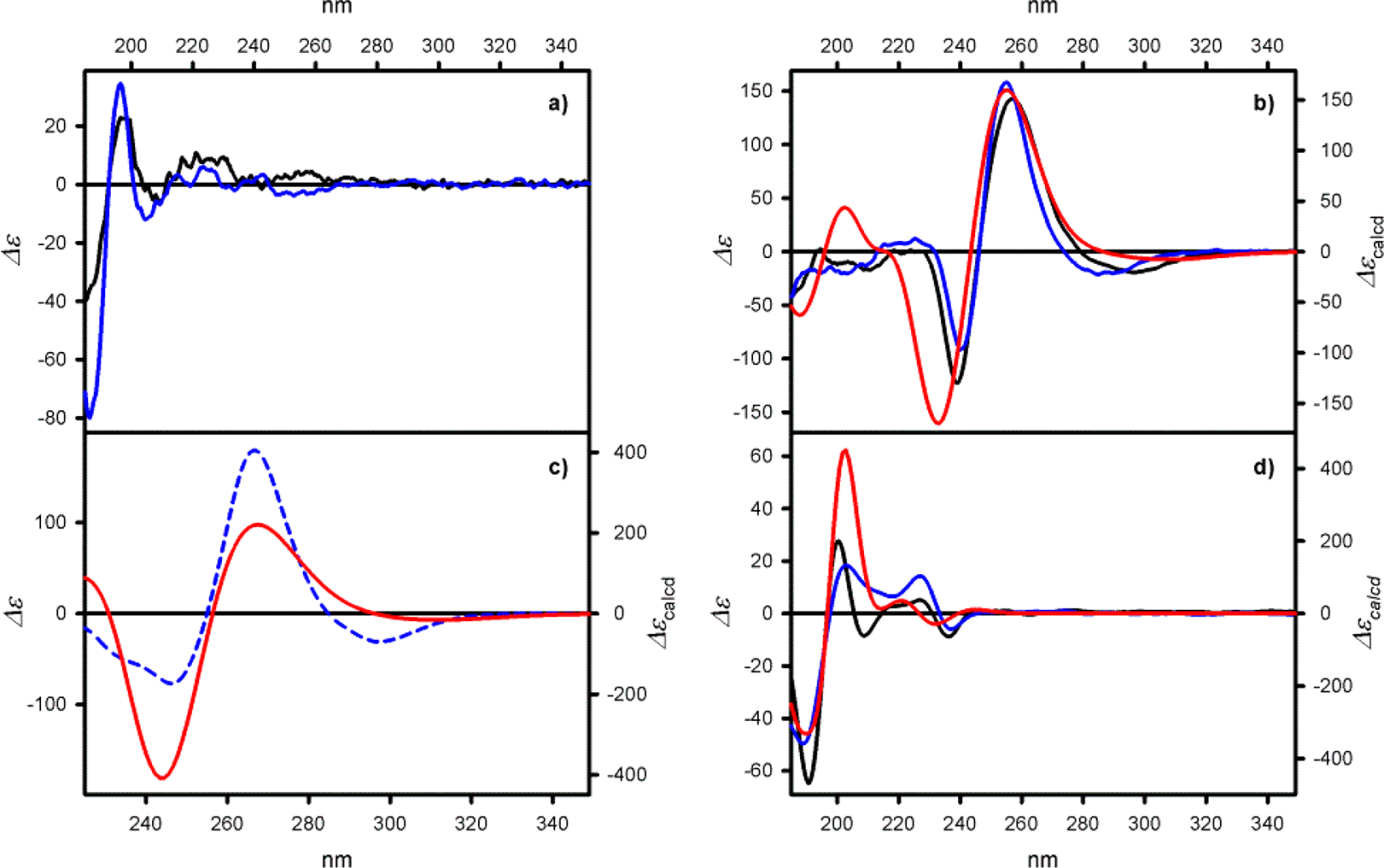
Example of ECD spectra of (a) **1a**, (b) **1b**, (c) **3**, and (d) **9a**, measured in cyclohexane (solid black lines), acetonitrile (solid blue lines), or dichloromethane (dashed blue lines). The calculated at the TD-CAM-B3LYP/6-311++G(d,p) level ECD spectra for (b) **1b**, (c) **3**, and **9a** (red lines). The calculated ECD spectra have been Boltzmann-averaged based on ΔΔ*G* values. Wavelengths have been corrected to match the experimental UV maxima.

For **1a**, four Cotton effects of low and middle intensity are observed. The most intense CEs appear in the higher energy region of the ECD spectrum at around 200 nm. In this particular high energy region, the part of the ECD spectrum of **1a** resembles the ECD spectrum of nonfunctionalized trianglamine.^42^ The most intense ^1^B_a_ electronic transitions, which are considered independent from the rotation of the benzene rings, are polarized along the chromophore axis connecting the 1,4-benzene carbon atoms. The Cotton effects generated by ^1^B_a_ electronic transitions in each pair of arene chromophores reveal a positive exciton couplet.

Replacing hydrogen atoms with methoxy groups in **2a** has caused a bathochromic shift of the absorption bands, which is associated with the hyperchromic effect. For example, the amplitude (*A* = *Δε*_long_ – *Δε*_short_) of exciton-type CEs, originating from an interaction between electronic dipole transition moments polarized along the long axis of the chromophores, equals *A* = −200, which is 10-fold higher than the ones found in the case of **1a**.

ECD spectra of **5a** and **6a**, measured in cyclohexane, show similar shape and sequence of CEs. In the ECD spectrum of **5a**, there are three optically active bands of medium intensity and negative signs, which appear at ca. 240, 210, and 195 nm. In the ECD spectrum of basic **6a**, the number of bands, observed in the spectral region below 250 nm, is reduced. The broad CD band at around 205 nm, would cover the CEs observed at 210 and 195 nm for **7a**. The addition of polar substituents at the C5 positions has simplified the CD spectra. The ECD spectra of **7a** and **8a** exhibit exciton-type positive couplets between 205 and 195 nm of amplitudes *A* = +90 and *A* = 80 (only visible in acetonitrile), respectively, for **7a** and **8a**, followed by weak −CEs appearing at around 245 nm.

The presence of four nonconjugated aromatic chromophores in **9a** has made the ECD spectrum more complex. The sequence of CEs, observed from lower to higher energies, is negative/positive/negative/positive/negative. The latter two form a positive exciton-type couplet.

The significant differences between ECD spectra of respective urea and thiourea derivatives are clearly visible. In the case of **1b**–**3** and **9b**, the observed CEs are as follows: long-wavelength positive CE at around 300 nm of moderate amplitude, a strong couplet of CEs: positive at around 255 nm and negative at ca. 245 nm. In the case of isotrianglamine derivatives **5b**–**8b**, there are additional short-wavelength CEs appearing between 225 and 190 nm and of the sequence +/−/+. The data coherence indicates that mutual orientation of aromatic chromophores is not pertinent for CEs observed in the spectral region between 300 and 245 nm. In other words, ECD spectra of thiourea derivatives **1b**–**9b** in the first approximation reflect the stereochemistry of the amine moiety rather than the conformation of the macrocycle.

ECD spectra, measured for acyclic analogues of macrocyclic amines **10**–**12**,^75−78^ confirm this hypothesis. The model compounds **10**–**12** have corresponded to one vertex of a given macrocycle. The observed structure–spectra relationships follow the general trends visible for their macrocyclic counterparts; i.e., the coherent results have been obtained for thiourea derivatives **10b**–**12b**, whereas the ECD spectra of ureas **10a**–**12a** have been characterized by greater variability.

**Figure 4:**
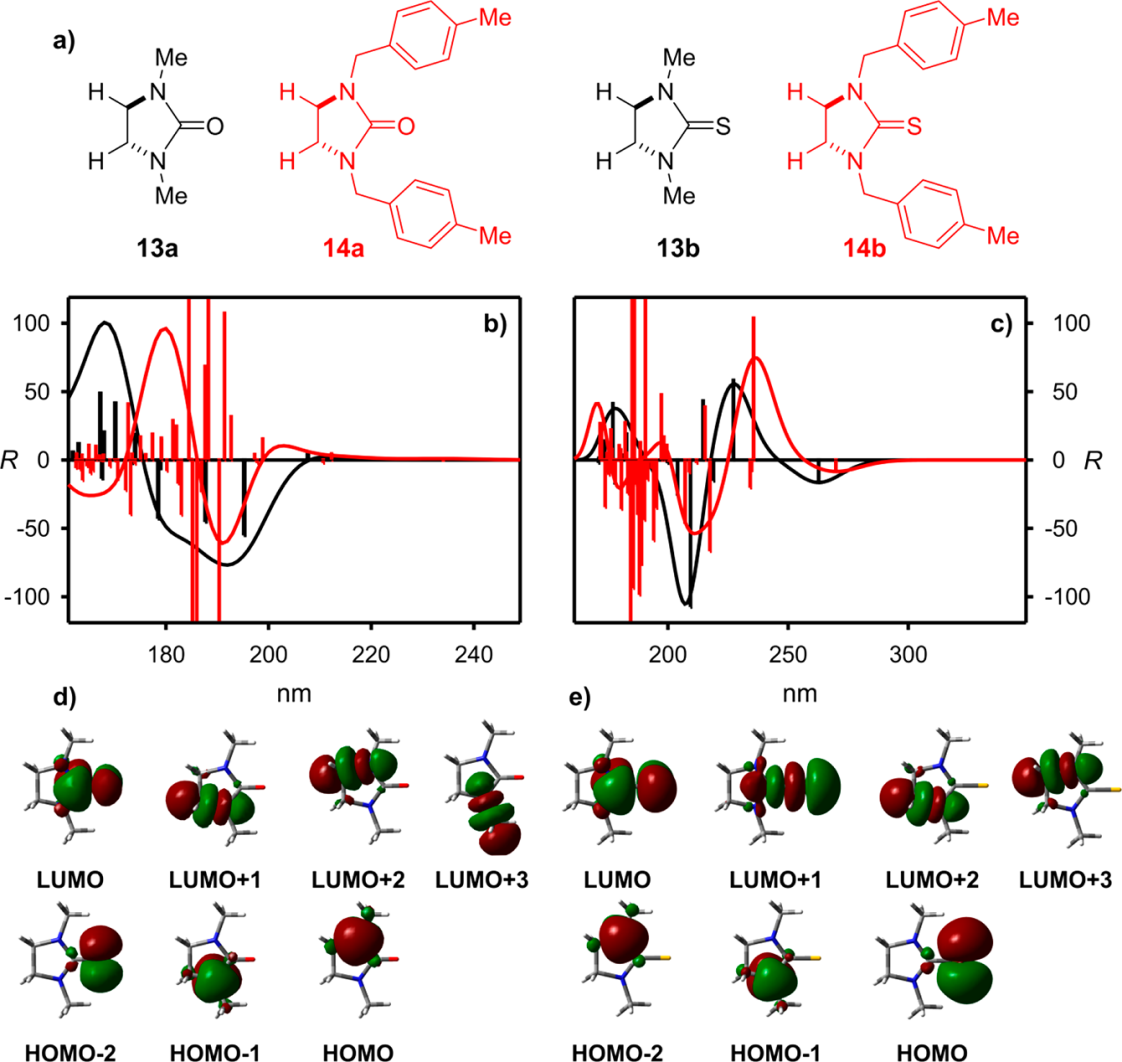
(a) Structures of model compounds **13a**,**b** and **14a**,**b**. Calculated at the TD-CAM-B3LYP/6-311++G(d,p) ECD spectra of model compounds (b) **13a** (black lines) and **14a** (red lines) and (c) **13b** (black lines) and **14b** (red lines). Vertical bars represent calculated rotatory strengths. Wavelengths were not corrected. Molecular orbitals involved in the low-energy electronic transitions in (d) **13a** and (e) **13b**.

The calculations of ECD spectra for model “in silico” generated compounds **13** and **14** (Figure 4a–c) shed additional light on the origin of the observed Cotton effects.

The ECD spectrum calculated for “pure” tertiary urea chromophore **13a** (Figure 4b and Figure S44) consists of a dozen optically active electronic transitions, which, after Gaussian approximation, give three main CEs. The first one, of the positive sign, is very weak and originated mainly from the HOMO–LUMO transition involving a nitrogen atom lone pair (*n*_N_) and π* orbitals. The next four electronic transitions generate negative rotatory strengths and reveal a negative CE at the higher energy region. These electronic transitions involve HOMO, HOMO–1 and LUMO+1, LUMO+2, and LUMO+3 orbitals (see Figure 4d and Figure S45). In contrast to the *n*_O_–π and π–π* transitions,^79^ typical of an amide chromophore, the electronic transitions generating negative CE involve mainly nitrogen lone pairs (*n*_N_–π* transitions) and virtual orbitals of higher energy (*n*_N_–RY* transitions). Note, that the third calculated CE, which appears at a high energy region (below 180 nm), has been hardly visible in the real systems. The shape of the ECD spectrum calculated for model **13b** is very close to the experimental spectra of thiourea derivatives **1b**–**12b**. The first long-wavelength negative CEs calculated for **13b** originate from single electronic transitions involving electrons from sulfur lone pairs and HOMO+1 orbitals (Figure 4c,e). The second and the third CEs, which form quasi-exciton couplet, originate from electronic transitions involving nitrogen lone pairs (HOMO and LUMO+1 orbitals, *n*_N_–RY* transitions). It is worth adding that the *n*_S_–π* transition, expected for such a chromophore, is characterized by low oscillatory and rotatory strengths.

The contrasting results have been obtained for the model *N*-benzylureas and thioureas **14a** and **14b**, respectively. ECD spectrum of **14a** is a superposition of a large number of electronic transitions, and the effects, having their source from transitions within the urea chromophore, have been overwhelmed by π–π* electronic transitions within aromatic chromophores. However, the presence of aromatic groups in **14b** affects only the higher energy region of the spectrum. The lower energy spectral region is virtually the same for **13b** and **14b**.

**Figure 5:**
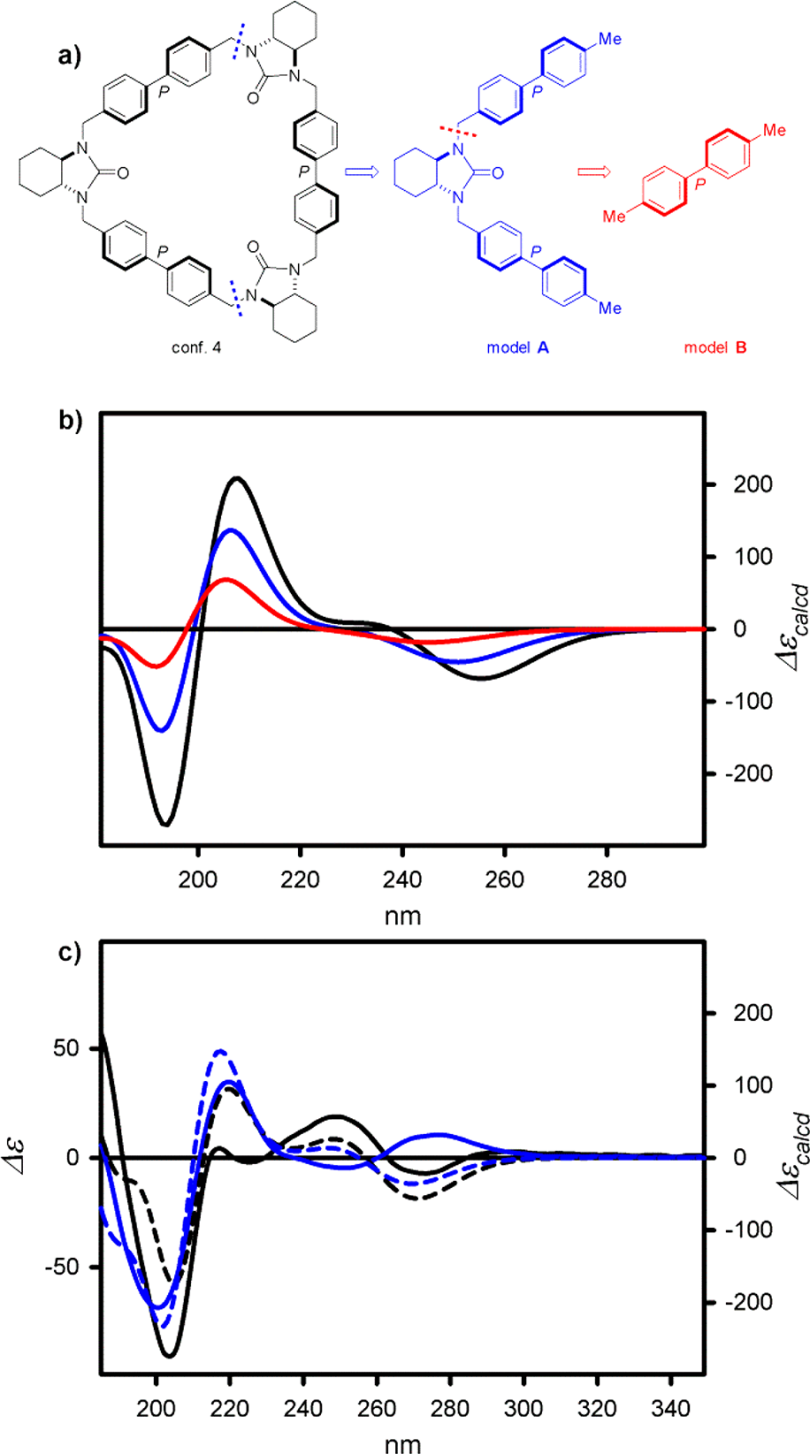
(a) Structures of model compounds **A** and **B** originating from conformer no. 4 of **4**. (b) Calculated at the TD-CAM-B3LYP/6-311++G(d,p) ECD spectra of conformer no. 4 of **4** (black line) and model compounds **A** (blue line) and **B** (red line). Wavelengths have been not corrected. (c) ECD spectra of **4** measured in cyclohexane (solid black lines) and acetonitrile (solid blue line) and the ECD spectra of **4** calculated at the TD-DFT level in the gas phase (dashed black line) and with the use of solvent model of acetonitrile (dashed blue line). The calculated ECD spectra have been Boltzmann-averaged. Wavelengths have been corrected to match the experimental UV maxima.

Macrocycle **4** represents by far the most complex and, thus, the most interesting system. The presence of the biphenyl chromophore, in principle, should allow for an interpretation of ECD spectra of **4** in terms of exciton coupling.^79,80^ The experimental and calculated ECD spectra of **4** and of the model compounds have been shown in Figure 5.

Elongation of the chromophoric system in **4**, and therefore a change of electronic properties, has been revealed in the appearance of two strong absorption UV bands. The first one, observed at around 250 nm, is reflected in the ECD spectrum as bisignate Cotton effects of a low amplitude. The higher energy UV band at around 200 nm is associated with a positive exciton couplet. At first approximation, the sign of this lower energy Cotton effect can be associated with the helicity of the biphenyl moiety,^79−84^ whereas the higher energy region may be dominated by interchromophoric interactions. However, the allowed low-energy electronic transition of ^1^B_a_ type (appeared at ca. 250 nm in experimental UV spectrum) is polarized along the long axis of the chromophore;^81−84^ thus, if so, this transition should be responsible for the interchromophoric exciton effects. The electronic excitations of ^1^B_b_ type, that are higher in energy, are polarized perpendicularly to the long axis of chromophore. Having assumed no exchange of electrons between phenyl fragments, these transitions should constitute a model example of exciton interactions.^79^ Calculations of theoretical UV and ECD spectra, conducted for the model (4,4′-dimethyl)biphenyl molecule (**15**), have helped us with an analysis of the experimental ECD spectrum of **4** (see Figure S46). For the sake of such calculations, we have generated 36 conformers of **15**, varied by the values of the twist angle ω (10 degrees step, from −180° to +180°). The ECD spectra calculated for individual conformers of **15** exhibit at least three main Cotton effects, which correlate with two UV absorption bands. The intensities of low-energy UV and ECD bands are highly dependent on the twist angle ω and change periodically (see Figure S47). The highest intensity of the low-energy UV band is achieved for the planar molecule, for which the calculated rotatory strengths are zero. As one can expect, the appearance of low-energy CD band(s) is achieved for the twisted chromophore. The maximum amplitude of calculated CEs has been estimated for structures twisted by ±120° and ±60°. Both UV and ECD bands gradually vanished when the aromatic parts became perpendicular. The amplitudes of calculated higher energy Cotton effects exhibit strong dependence on the biphenyl twist and change periodically as well. However, the relationship between the structure and the chiroptical response is not straightforward, as one can expect from a simple interacting excitons model present in the literature.^79^ In the calculated ECD spectra for conformers of **15**, the higher energy Cotton effects have reached the extreme amplitudes for structures characterized by the value of a twist angle, such as ±150°, ±110°, ±80, and ±30°. On the other hand, one should bear in mind that the exciton model is fully valid, as long as the interacting chromophores do not exchange electrons. Biphenyl follows such a simple model only in a specific, narrow range of values of the twist angle ω. To sum up this paragraph, the results obtained so far suggest that the observed ECD spectrum of **4** reflects rather the dynamic helicity of the chromophore(s) than an effect of interchromophoric interactions between pairs of neighboring biphenyls.

To confirm this hypothesis, we, at first, calculated ECD spectra for the representative low-energy *C*_3_-symmetrical, and homohelical conformers no. 1 and no. 4 and for the heterohelical (*MPP*) conformer no. 3. Additionally, all these structures were divided into two parts **A** and **B**, for which the ECD spectra were calculated at the same level of theory as it has been done for their parent molecules (see Figure 5a,**b** and Figure S104). Model structure **A** may be treated as one vertex and two sides of the parent triangle, whereas model structure **B** is represented by one side of the triangle, namely, the biphenyl chromophore. It is worth noting that the torsion angles and bond lengths were exactly the same in models **A** and **B** as in the parent triangular molecules.

It is evident that the calculated spectra for a given series of model structures are of the same shape. Amplitudes of respective Cotton effects consistently increase with each added chromophoric subunit. In the case of *MP*-heterohelical structure **A**, rooted from conformer no. 3 of **4**, the resulting ECD spectrum exhibits rather dominant influence of *M*-helical chromophore on net chiroptical properties, than the effect of interchromophoric interactions. If any, these interactions might be treated as negligible.

An increase of the environment polarity has enhanced the amplitude of short-wavelength CEs keeping the sequence unchanged. A more pronounced effect is visible for low-energy bands. The sequence of CEs appearing at around 275 and 250 nm is negative/positive (−/+) when the measurements have been done in nonpolar cyclohexane. In acetonitrile, however, the sequence of signs (+/−) has been mirrored, which may be associated either with the change of biphenyl helicity from *M* to *P* or with the change of relative populations of low energy species, as the calculations performed with the use of IEFPCM solvent model indicated (*vide supra*).

To fully correlate the structure with chiroptical properties, we have calculated theoretical ECD spectra of **4**, for molecules in nonpolar as well as in polar environments. In general, the matching between experimental and calculated ECD spectra of **4** (Figure 5c) is good. In particular, a very good agreement is visible in a higher energy region of spectra measured in acetonitrile and calculated with the use of IEFPCM model. However, in this case, the calculated low-energy bands exhibit the opposite signs to the experimental ones. The possible reason is not a perfect reproduction of the real conformational equilibrium by the method used for computations and overestimation of contribution of conformer no. 4.^85^

A similar calculation procedure applied for macrocycles **1a**, **1b**, **3**, **5a**, **5b**, **9a**, and **9b** has provided mixed results (see Figures S48–S75). While ECD spectra of thiourea-derived macrocycles have been very well reproduced, the reproduction of experimental ECD spectra of **1a** and **5a** was rather poor, with an exception of **9a**. Noticeably, reproduction of the ECD spectrum of **9a** measured in cyclohexane by theoretical calculations is good. However, even better agreement is achieved between the spectrum measured in acetonitrile and the one calculated with the use of the acetonitrile solvent model. It is worth noting that the ECD spectrum of **9a** is affected mostly by intrachromophoric interactions, more than the interactions between neighboring aromatic rings (see Figure S106). Therefore, the “chiroptical behavior” of **9a** is similar to that found for **4a**, and for both cases one can note dynamic induction of optical activity in the aromatic linker.

### Single-Crystal X-ray Diffraction Studies

The presence of functional groups and the possibility of adaptation of different stable conformations has made the macrocycles **1**–**9** good candidates for being molecular building blocks (tectons) in molecular tectonics. This part of the study corresponds well with an increasing interest, observed in recent years, in applications of chiral, periodical, and (formally) symmetrical polyaza macrocycles in various aspects of material chemistry and crystal engineering.^86−91^ In partiuclar, the manipulation of the shape complementarity between macrocyclic molecules would give a premise to the formation of various supramolecular architectures stabilized by the guest molecules.

For most of the cases, the attempts to obtain crystalline materials from **1**–**9**, suitable for further study, despite various crystallization procedures applied, led to the formation of amorphous materials. However, among the macrocycles tested, compounds **1a**, **1b**, **3**, and **8b** have turned out promising candidates for the formation of definable crystalline materials.

Finally, single crystals suitable for X-ray analysis have been obtained by slow evaporation of tetrahydrofuran solution (form **1a_I**) or by slow diffusion of diethyl ether vapors to chloroform solutions of respective macrocycle (forms **1a_II**, **1b_II**, **3**, **8b**). Compounds **1a**, **1b**, and **8b** crystallize in an orthorhombic system in the *P*2_1_2_1_2_1_ space group, while compound **3** crystallizes in a triclinic system in the space group *P*1.^92^

Depending on the solvent used in crystallization, trianglamine **1a** has formed solvates with THF and water–crystal form **1a_I** or chloroform–crystal form **1a_II**. Previously, we have found that in the crystals of the macrocycle **1b** (form **1b_I**, space group *P*2_1_) the asymmetry unit contains two molecules of macrocycle and three solvent (ethyl acetate) molecules.^53^ In this study, we have obtained a crystal form of **1b** with chloroform inclusion—a crystal form **1b_II**. Also compounds **3** and **8b** have crystallized as solvates with chloroform.

**Figure 6:**
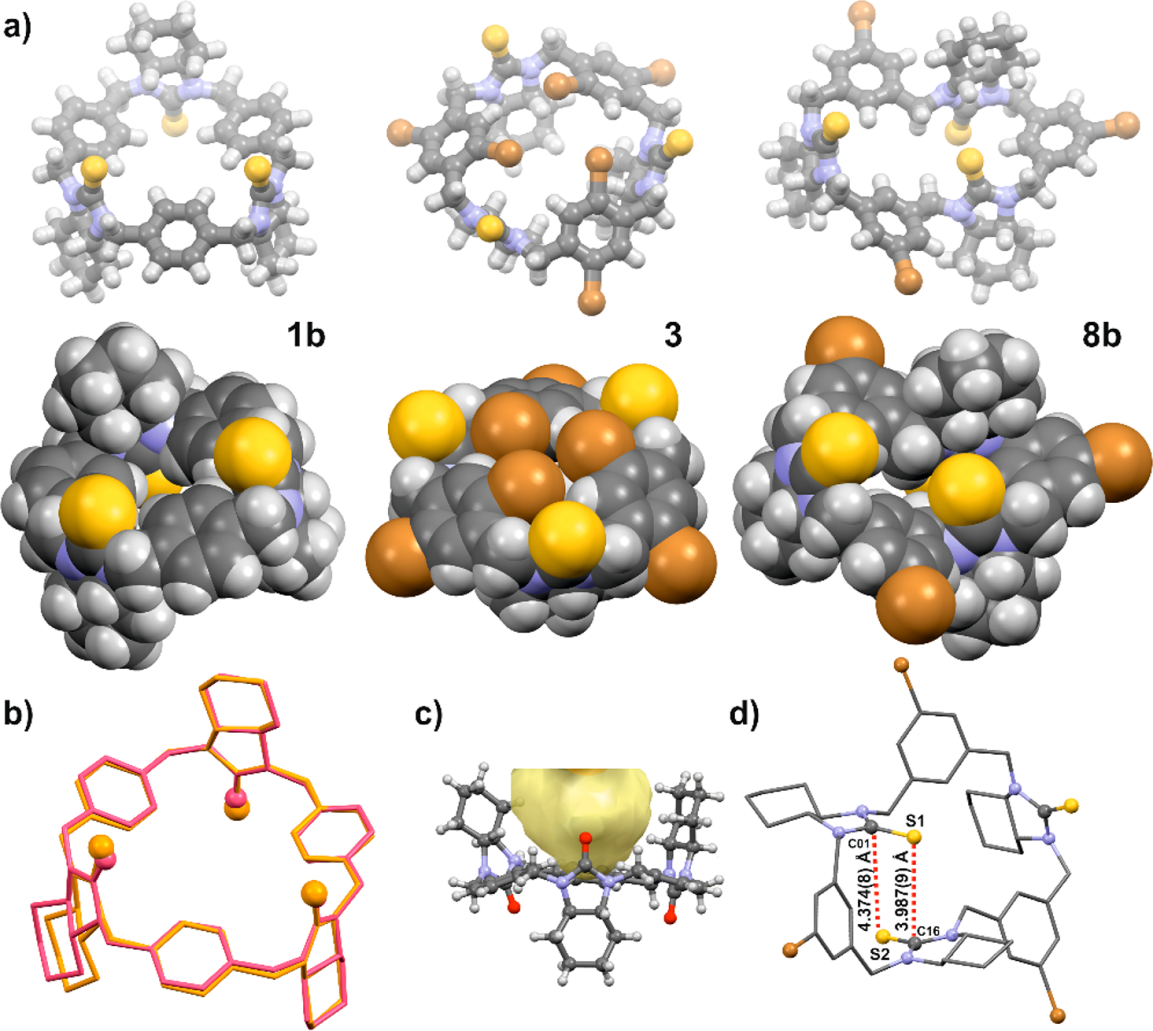
(a) Structures of macrocycles **1b**, **3**, and **8b** in the crystals represented as balls and sticks style and as a spacefills model in the upper and lower panel, respectively. (b) Overlaid molecular structures of basic urea-derived trianglamine **1a** (pink color) and thiourea trianglamine **1b** (orange color) from crystal forms **1a_II** and **1b_II**, respectively. Hydrogen atoms were omitted for clarity; oxygen and sulfur atoms are distinguished in a ball style. (c) Illustration of a void created by molecule **1a** in the crystal. (d) Arrangement of molecule **8b** in the solid state and measured distances between thiourea groups: S1···C16 and S2···C1 (marked in red dashed lines).

Formally symmetrical (*D*_3_ or *C*_3_ molecular symmetry), the urea and thiourea derivatives of trianglamines and isotrianglamine in the crystal phase have not taken full advantage of symmetry and adapt either *C*_1_ (**1a**, **1b**, **8b**) or *quasi*-*C*_3_ symmetry (**3**). In the latter case (in fact, formally nonsymmetrical), only small deviations from *C*_3_ symmetry are noticed. At the molecular level, investigated macrocyclic polyamines adapt cone and partial cone conformations (Figure 6a).

In the crystals, urea or thiourea macrocycles with a nonsubstituted aromatic ring adapt a partial cone conformation with the arrangement of aromatic linkers in the macrocycle rim plane (defined by nitrogen atoms) and with one cyclohexane ring facing up and two facing down (Figure 6b). This arrangement creates a kind of “aliphatic forceps” cavity (Figure 6c), in which capturing a solvent molecule takes place. Incorporation of two bromine atoms into each of the aromatic linkers results in the change of molecular conformation to a cone, with arrangement of thiourea groups perpendicular to a mean macrocycle plane and bromine atoms (from an upper rim) facing each other. A folded structure is observed for macrocycles embellished with only one bromine atom in each of the aromatic linkers (at C5 positions). However, taking into account the mutual arrangement of C=S bonds, this particular structure might be still classified as a partial cone conformer (the sequence of pseudotorsion angles γ, has been found as follows: *T*^*+*^*, S*^*–*^*, T*^*–*^). This apparently chaotic structure shows a peculiar order. In this particular case, bromine atoms are facing outside of the macrocycle’s ring. One of the thiourea groups is located perpendicularly to molecular ring, while two thiourea groups are facing each other. The whole structure is stabilized by weak S···C dipole–dipole interactions (Figure 6d).

### Self-Sorting Phenomenon and Supramolecular Architectures in the Crystals of Macrocycles

In the crystals, supramolecular structures of macrocycles are stabilized by weak intermolecular noncovalent interactions, unless they form solvent-less phases. In the solvated crystals, hydrogen bonds formed between host and guest molecules are the structure-driven forces. Incorporating oxygen atoms via urea motifs into a macrocycle skeleton provides additional possibilities to the hydrogen bonding, C–H···O, also between host molecules.

Introduction of sulfur atoms in thiourea motifs has significantly expanded the possibilities of creating new noncovalent interactions (see: Hirshfeld surface analysis in the SI). Sulfur, an electron-rich entity, constitutes an excellent candidate for the hydrogen/halogen bond acceptor. In the case of **1b**, the structure of the macrocycle is stabilized by C–H···S interactions. The supramolecular system could be expanded by adding guest molecules containing chlorine atoms, e.g., chloroform. Here, significant contribution to stabilized noncovalent interactions between guest and host molecules is given by C–Cl···S halogen and C–H···Cl hydrogen bonds.

**Figure 7:**
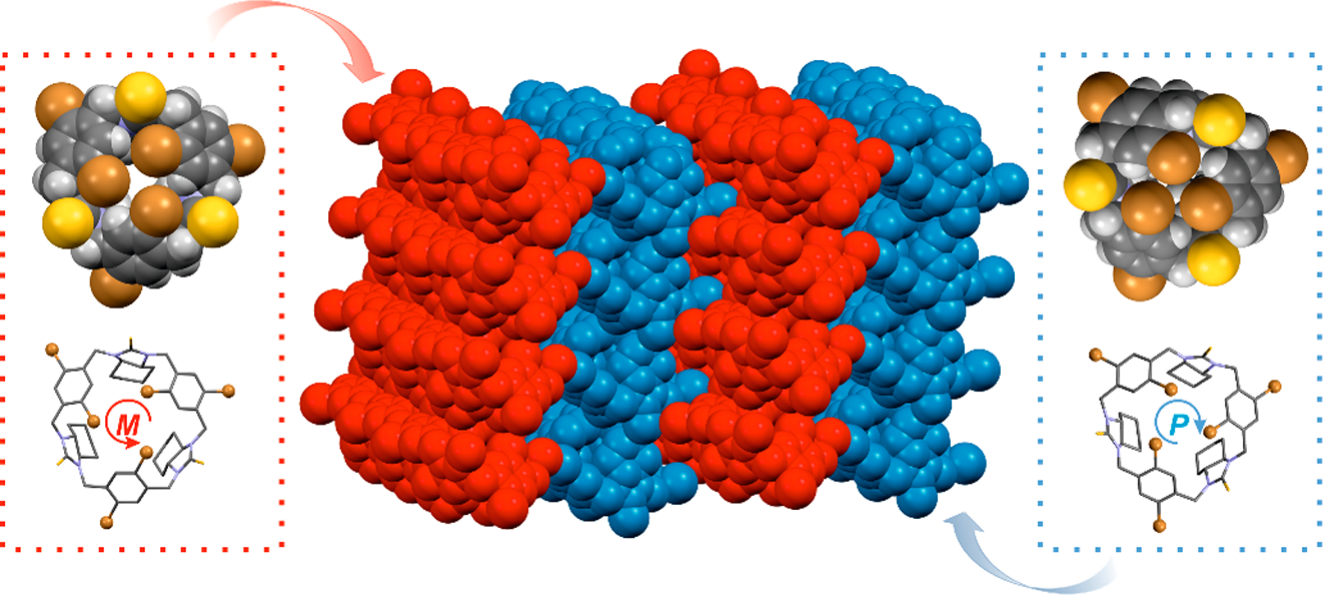
Isomers of **3** and their narcissistic self-sorting in the crystal. Diasteroisomers of **3** have been distinguished in red and blue colors.

Trianglamine **3** containing two bromine atoms in each of the aromatic linkers has formed solvated crystals which belong to the space group *P*_1_. The crystal phase is characterized by the presence of two symmetry independent macrocyclic molecules in a triclinic unit cell. Molecules of **3** form two rotational isomers that differ in the spatial arrangement of the direction of bromine atoms. When looking at a single molecule, bromine atoms can be arranged either in a counter clockwise (red arrow in Figure 7) or a clockwise direction (blue arrow in Figure 7) and, therefore, characterized in terms of helicity as *M* or *P* diastereoisomers, respectively. This quasi (ignoring the chirality of the amine) mirror-image relationship reflects in sequences of respective torsion and pseudotorsion angles, which characterize the molecular conformation. The sequences of pseudotorsion angles γ found in the crystal structures, are found as follows: *S*^*–*^*, S*^*–*^*, S*^*–*^ and *S*^*+*^*, S*^*+*^*, S*^*+*^, respectively, for *M* or *P* diastereoisomer. In the crystals, both, left-handed and right-handed isomers occurr in the same amount, which corresponds well to the results of the NMR study. Furthermore, in the crystals of **3**, the narcissistic self-sorting phenomenon has taken place.^23,93−95^ The given “helical *M* or *P* isomers” form “homohelical” *M* or *P* molecular layers.

**Figure 8:**
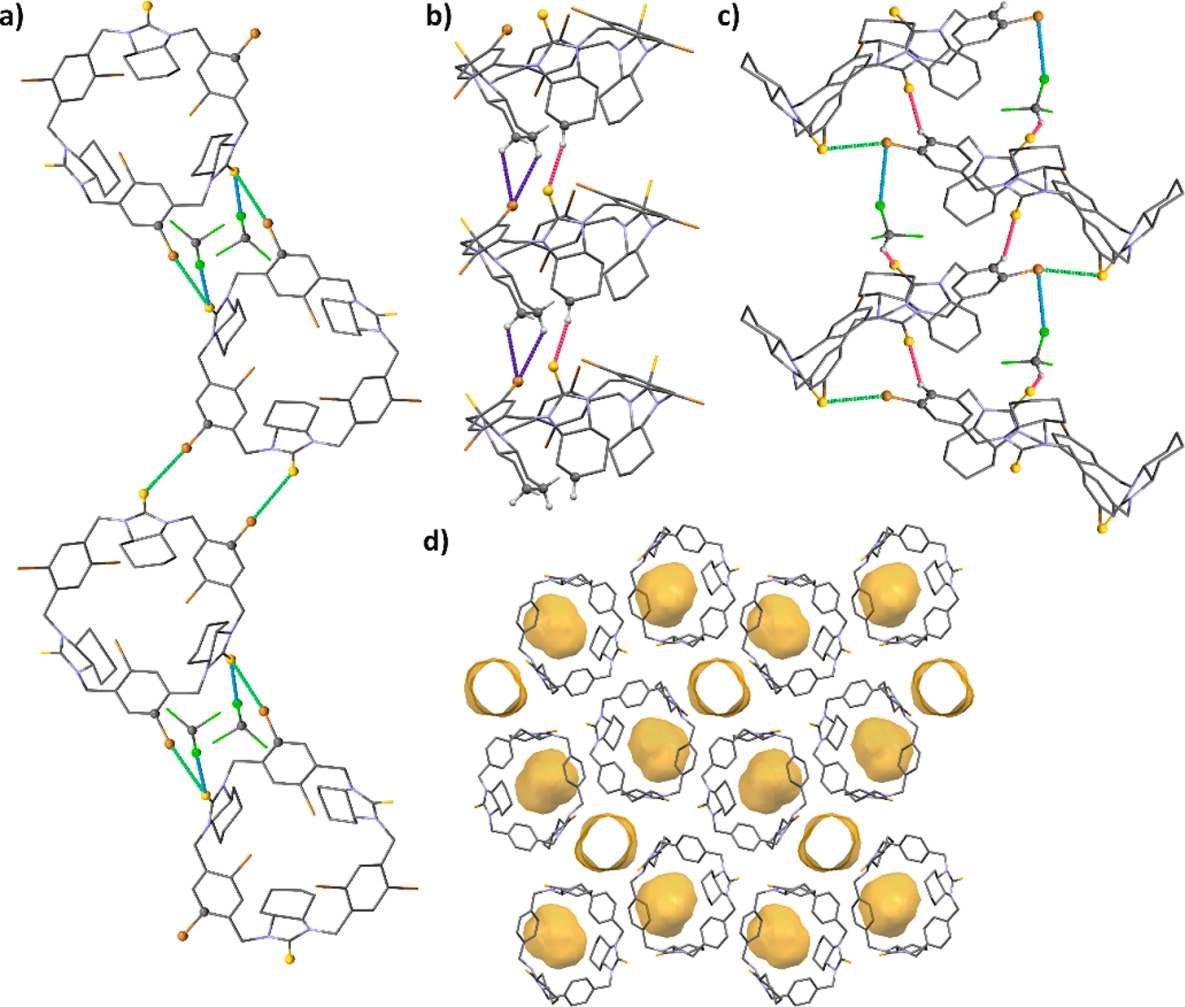
Supramolecular architecture in the crystals: (a) a halogen-bonded chain in the crystal of **3**; (b) a hydrogen-bonded columnar assembly of **3**; and (c) a halogen- and hydrogen-bonded chain in the crystal of **8b**. Halogen bonds have been marked as blue and green dashed lines; hydrogen bonds have been marked as dark-blue and pink dashed lines. (d) Structural voids and channels formed by host macrocyclic molecules in the crystals of **1b_II**. View along [100] direction. Probe radius: 1.5 Å.

Structures of both *M* and *P* diastereoisomers are stabilized by sets of C–Br···S interactions between the host molecules (the mean value of distances between Br and S atoms: *d*_Br···S_ = 3.46(7) Å, estimated for four observations) and by S···Cl–C (*d*_Cl···S_ = 3.363(6) Å, 2 observations) interactions specific to the host–guest systems (Figure 8a). Despite the halogen bonds, the structure is stabilized by a set of hydrogen bonds of the, e.g., C–H···S (*d*_H···S_ = 2.75(14) Å, 11 observations) and C–H···Br (*d*_H···Br_ = 2.91(15) Å, 17 observations) types and formed between macrocycles (Figure 8b) and by C–H···S (*d*_H···S_ = 2.85(1) Å, 2 observations) and C–H···Cl (*d*_H···Cl_ = 2.83(10) Å, 6 observations) interactions between the host and guest (solvent) molecules.

Folded molecules of **8b** have co-created unexpected supramolecular architecture in the solvated crystals. Although at first glance the structure seems to be highly disordered, it can be presented as ordered upon in-depth structural analysis.

Noncovalent interactions are responsible for the ordered supramolecular assembly of **8b** and solvent molecules in the crystalline phase. The host molecule system is stabilized by C–Br···S (*d*_Br···S_ = 3.381(3) Å, 1 observation) and C–H···S (*d*_H···S_ = 2.77(17) Å, 6 observations) interactions, while host–guest arrangement is stabilized by C–H···Cl (*d*_H···Cl_ = 2.85 Å, 1 observation), S···H–C (*d*_H···S_ = 2.79 Å, 1 observation), and Br···Cl–C (*d*_Cl···Br_ = 3.414(4) Å, 1 observation) interactions. Therefore, one can say that seemingly disordered molecules form an ordered supramolecular network stabilized by hydrogen and halogen bonds (Figure 8c).

Similarly to the previously studied chiral DACH-based polyazamacrocycles of (iso)trianglimine, (iso)trianglamine, resorcinsalen, or calixsalen structures,^8^ all of the investigated macrocycles have formed solvates in the solid state. As mentioned earlier, **1b** has formed highly solvated crystals with ethyl acetate and crystallize in the *P*2_1_ space group (crystal form **1b_I**).^53^ In the crystals of **1b_I**, the volume of a unit cell occupied by structural channels and filled by solvent molecules has taken up to 27%.

In isostructural inclusion crystals of **1a**, sets of structural voids are connected by narrow channels and filled with solvent, namely, tetrahydrofuran (**1a_I**) or chloroform (**1a_II**) molecules. Crystals of **1b_II** (Figure 8d) and **3** contain isolated voids and channels created between macrocyclic host molecules and filled with guest chloroform molecules. In the above-mentioned crystal forms, the contribution of structural voids and channels in the corresponding unit cell volume ranges from 15% to 20% (probe radius: 1.5 Å). In comparison, in the crystal phase formed by macrocycles **8b** there are also structural voids with entrapped chloroform molecules and the contribution of voids to the unit cell volume does not exceed 10%.

### Compound Sources

(1*R*,2*R*)-Diaminocyclohexane, 1,1′- carbonyldiimidazole, and 1,1′-thiocarbonyldiimidazole were purchased from commercial suppliers. The imine precursors of macrocyclic amines **1**-**9** were synthesized according to the published procedures^11,13,14,65−67^ and used as received for further steps. The *N*-benzyl amines **10**–**12** were synthesized according to the published procedure.^75−78^

## Conclusions

In contrast to the highly symmetrical and rigid bridged trianglamine,^42^ characterized by a perpendicular arrangement of aromatic linkers to the mean macrocycle plane, the macrocyclic ureas and thioureas may adapt three main conformations. These conformations might be conveniently distinguished by taking into account mutual orientation of C=X bonds.

The most symmetrical (of *D*_3_ or *D*_2_ symmetry) flattened conformers with a coplanar arrangement of all C=X bonds are over 20 kcal mol^–1^ higher in energy than cone and partial cone conformers. The *C*_3_- or *C*_2_-symmetrical cone conformers are characterized by parallel orientation of C=X bonds, whereas in the case of the partial cone conformer, one C=X bond is situated antiparallelly to the remaining two.

In general, urea derivatives exhibit a tendency to adapt a more symmetrical cone conformation. Higher diversity is observed for the thiourea derivatives. While nonsubstituted **1b** exists in the solution and in the solid state as the partial cone conformer, the trianglamines substituted at C2 and C5, or those having expanded macrocycle cavity, as well as isotrianglamine derivatives, predominantly adapt a cone conformation.

DFT calculations conducted for model compounds **1a**, **1b**, **3**, **4**, **9a**, and **9b** provide consistent results and correspond well to the results of NMR measurements. In each of the cases, the preference is visible for *C*_2_- or *C*_3_-symmetrical structures over these of higher *D*_2_-, *D*_3_-, and lower *C*_1_ symmetry.

The analysis of the ECD data led to formulating some generalities. First, there are visible significant differences between ECD spectra of urea and thiourea derivatives of the same macrocycle. Second, contrary to ureas, the ECD data obtained for thiourea derivatives is consistent and shows a sequence of the Cotton effects of the same pattern. Third, the solvent effect is more visible for ureas than for their thiourea counterparts. Fourthly, ECD spectra of thiourea derivatives reflect the closest neighborhood of the chromophore, whereas the observed Cotton effects in ECD spectra of the macrocyclic urea derivatives originated from interactions between aromatic chromophores. The exceptions are **4a** and **9**, where the aromatic linkers dynamically adjust the conformation to the chiral neighborhood. Due to the different sterical requirements of urea and thiourea moieties, the complementarity between systems is not visible. In other words, structural data extracted from ECD spectra of urea-derived macrocycles is of no use in analyzing the conformation of the thioureas and *vice versa*.

The structural diversity of urea and thiourea macrocycles reflects in their ability to form host–guest complexes in the solid state and has had no counterpart among other macrocyclic derivatives of a similar type known so far. For example, the methylene-bridged trianglamine, the closest in the substitution mode, preserves high *D*_3_ symmetry in the solution and in the solid state.^42^ The compound is characterized by a rigid structure and perpendicular orientation of aromatic rings to the mean macrocycle plane, which makes the macrocycle cavity open to guest molecules. In the case of urea and thiourea derivatives, the folding of diamine and/or aromatic parts blocks the molecular cavity. On the other hand, the less symmetrical macrocycles show propensity to form highly solvated crystals, which is of potential utility in further applications of these compounds as molecular sieves and selectors.

An interesting feature of the macrocycle **3** is the tendency toward supramolecular self-sorting in the solid state. The macrocycle exists in the solution as the 1:1 mixture of cone-type conformational diastereoisomers, which differ in the orientation of C–Br bonds (clockwise and counter clockwise). In the solid state, helical conformers form homohelical layers. Incorporating bromine atom(s) into the aromatic linker in the macrocycle skeleton increases the role of halogen bonds in the formation and stabilization of supramolecular systems. Recently, in the field of crystal engineering, an increasing interest in designing the structures capable of forming halogen bonds has been observed.^96−99^

**Figure 9:**
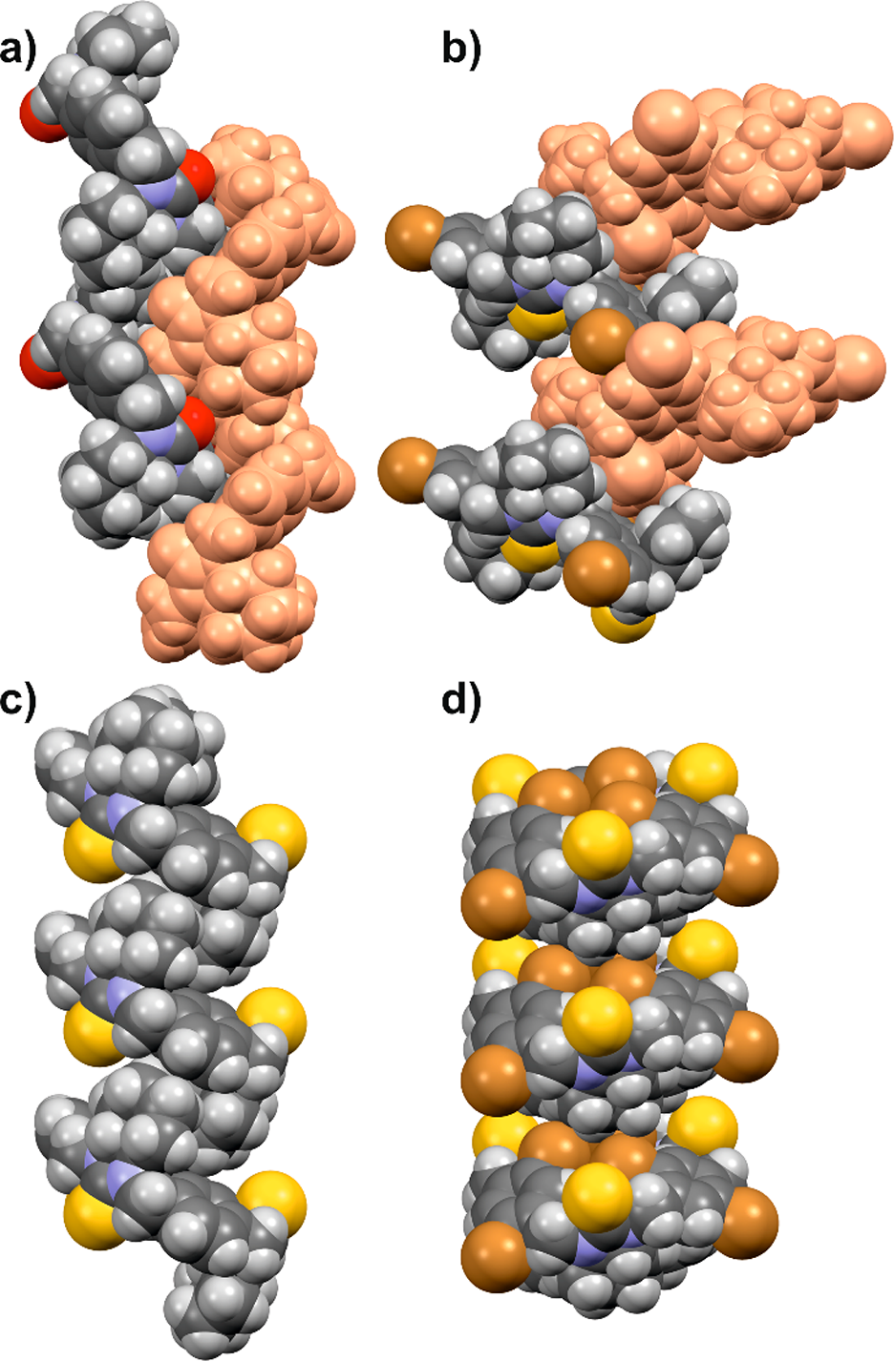
Various columnar systems of host macrocyclic molecules in the crystals: a zipper motif formed by two columnar stacks (distinguished in different colors) in (a) **1a_II** and (b) **8b**; a single columnar stack in (c) **1b_II** and (d) **3**. Macrocycles are drawn as van der Waals spheres.

In the crystals, host macrocyclic molecules have formed various columnar systems (see Figure 9).

In the crystal of **1b_I** macrocycles form a zipper motif created by two columnar stacks.^53^ Amines in the crystals of **1a** (**I** and **II**) and **8b** have adapted the same pattern, while in the crystals of **1b_II** and **3** macrocycles are arranged in single columnar stacks. In all cases, noncovalent intermolecular interactions have a significant impact on stabilizing the supramolecular structures. The current work in our laboratory is focusing on the applications of some of the compounds under study as ligands and/or gelators.

## Data Availability

The data underlying this study are available in the published article and its online Supporting Information.

## Supporting Information Available

detailed procedures for the synthesis of all compounds, and their characteristics, supplementary figures and tables, computational methods, details of X-ray diffraction analyses, and copies of ECD spectra. Energetic and selected structural data (Tables S1–S17) and selected and measured halogen bonds parameters (Tables S18 and S19). Experimental and calculated CD spectra along with calculated structures of individual low-energy conformers of model compounds **1a**, **1b**, **3b**, **4**, **5a**, **5b**, **9a** and **9b** (Figures S2–S106). X-ray determined structures and calculated contribution of noncovalent interactions in the crystals of **1a_I**, **1a_II**, **1b_II**, **3b** and **8b** based on Hirshfeld surface analysis (Figures S107–S130). Copies of ^1^H and ^13^C NMR spectra and Cartesian coordinates for all calculated structures (PDF)
FAIR data, including the primary NMR FID files, for compounds **1**–**12** (ZIP)
