# Supplementary material for: Unravelling Structural Dynamics, Supramolecular Behavior, and Chiroptical Properties of Enantiomerically Pure Macrocyclic Tertiary Ureas and Thioureas
Source: J Org Chem. 2022 Dec 8;88(1):285–99. doi: 10.1021/acs.joc.2c02319 (PMC9830626; doi:10.1021/acs.joc.2c02319)
Supplement: Supplementary file 1 — jo2c02319_si_001.pdf [file jo2c02319_si_001.pdf]

Supplementary Information for:

Unravelling structural dynamics, supramolecular behaviour and chiroptical properties of enantiomerically pure macrocyclic tertiary ureas and thioureas

Natalia Prusinowska,† Joanna Szymkowiak,‡ Marcin Kwit*†

† Faculty of Chemistry, Adam Mickiewicz University, Uniwersytetu Poznańskiego 8, 61 614 Poznań, Poland. E-mail: marcin.kwit@amu.edu.pl

‡ Faculty of Science, Department of Chemistry University of British Columbia, 2036 Main Mall, Vancouver, British Columbia, BC Canada V6T 1Z1

Table of contents

Experimental section ....SI 15

General procedure for synthesis of macrocyclic urea derivatives. ....SI 16

General procedure for synthesis of thiourea-derived macrocycles. ....SI 17

General procedure for synthesis of urea- and thiourea derivatives 10-12. ....SI 19

Comment on temperature-dependent ^{1}H NMR spectra of 2b ....SI 20

Computational details ....SI 21

Crystallographic details ....SI 22

Table S1. Total and free energies (*E*, *ΔG*, in Hartree), relative energies (*ΔE*, *ΔΔG* in kcal mol^{-1}), percentage populations and number of imaginary frequencies (#ImFreq) calculated at the B3LYP/6-311G(d,p) level for individual conformers of 1a. ....SI 24

Table S2. Total and free energies (*E*, *ΔG*, in Hartree), relative energies (*ΔE*, *ΔΔG* in kcal mol^{-1}), percentage populations and number of imaginary frequencies (#ImFreq) calculated at the IEFPCM(ACN)/B3LYP/6-311G(d,p) level for individual conformers of 1a. ....SI 24

Table S3. Total and free energies (*E*, *ΔG*, in Hartree), relative energies (*ΔE*, *ΔΔG* in kcal mol^{-1}), percentage populations and number of imaginary frequencies (#ImFreq) calculated at the B3LYP/6-311G(d,p) level for individual conformers of 1b. ....SI 24

Table S4. Total and free energies (*E*, *ΔG*, in Hartree), relative energies (*ΔE*, *ΔΔG* in kcal mol^{-1}), percentage populations and number of imaginary frequencies (#ImFreq) calculated at the B3LYP/6-311G(d,p) level for individual conformers of 3. ....SI 25

Table S5. Total and free energies (*E*, *ΔG*, in Hartree), relative energies (*ΔE*, *ΔΔG* in kcal mol^{-1}), percentage populations and number of imaginary frequencies (#ImFreq) calculated at the IEFPCM(CH_2Cl_2)/B3LYP/6-311G(d,p) level for individual conformers of 3. ....SI 25

Table S6. Total and free energies (*E*, *ΔG*, in Hartree), relative energies (*ΔE*, *ΔΔG* in kcal mol^{-1}), percentage populations and number of imaginary frequencies (#ImFreq) calculated at the B3LYP/6-311G(d,p) level for individual conformers of 4. ....SI 25

Table S7. Total and free energies (*E*, *ΔG*, in Hartree), relative energies (*ΔE*, *ΔΔG* in kcal mol^{-1}), percentage populations and number of imaginary frequencies (#ImFreq) calculated at the IEFPCM(ACN)/B3LYP/6-311G(d,p) level for individual conformers of 4. ....SI 26

Table S8. Total and free energies (*E*, *ΔG*, in Hartree), relative energies (*ΔE*, *ΔΔG* in kcal mol^{-1}), percentage populations and number of imaginary frequencies (#ImFreq) calculated at the B3LYP/6-311G(d,p) level for individual conformers of 5a. ....SI 26

Table S9. Total and free energies (*E*, *ΔG*, in Hartree), relative energies (*ΔE*, *ΔΔG* in kcal mol^{-1}), percentage populations and number of imaginary frequencies (#ImFreq) calculated at the IEFPCM(ACN)/B3LYP/6-311G(d,p) level for individual conformers of 5a. ....SI 26

Table S10. Total and free energies (*E*, *ΔG*, in Hartree), relative energies (*ΔE*, *ΔΔG* in kcal mol^{-1}), percentage populations and number of imaginary frequencies (#ImFreq) calculated at the B3LYP/6-311G(d,p) level for individual conformers of 5b. ....SI 27

Table S11. Total and free energies (E, ΔG, in Hartree), relative energies (ΔE, $\Delta\Delta G$ in kcal mol^{-1}), percentage populations and number of imaginary frequencies (#ImFreq) calculated at the B3LYP/6-311G(d,p) level for individual conformers of 9a. .... SI 27

Table S12. Total and free energies (E, ΔG, in Hartree), relative energies (ΔE, $\Delta\Delta G$ in kcal mol^{-1}), percentage populations and number of imaginary frequencies (#ImFreq) calculated at the IEFPCM(ACN)/B3LYP/6-311G(d,p) level for individual conformers of 9a. .... SI 27

Table S13. Total and free energies (E, ΔG, in Hartree), relative energies (ΔE, $\Delta\Delta G$ in kcal mol^{-1}), percentage populations and number of imaginary frequencies (#ImFreq) calculated at the B3LYP/6-311G(d,p) level for individual conformers of 9b. .... SI 28

Table S14. Total and free energies (E, ΔG, in Hartree), relative energies (ΔE, $\Delta\Delta G$ in kcal mol^{-1}), percentage populations and number of imaginary frequencies (#ImFreq) calculated at the IEFPCM(CH_2Cl_2)/B3LYP/6-311G(d,p) level for individual conformers of 9b. .... SI 28

Table S15. Relative free energies ($\Delta\Delta G°$, in kcal mol^{-1}), percentage populations, sequences of torsion angles $\beta,\alpha,\alpha',\beta'$, pseudotorsion angles γ and helicities found in calculated and X-ray determined the structures of 1a, 1b, 3, 5a, 5b, 8b, 9a and 9b. The symbols in parentheses indicate symmetry of the given calculated conformer. .... SI 29

Table S16. Experimental details. .... SI 32

Table S17. Calculated angles (φ) of inclination of the planes of aromatic linkers to the plane of the macrocycle rim. Plane of aromatic linker has been designated by all aromatic carbon atoms, and macrocycles planes – by all six nitrogen atoms from macrocyclic rim. .... SI 33

Hydrogen and halogen bonds parameters .... SI 34

Table S18. Selected hydrogen bonds parameters for 1a_I, 1a_II, 1b_II, 3, 8b. .... SI 34

Table S19. Selected halogen bonds parameters for 1b_II, 3 and 8b. .... SI 36

Figure S1. Parts of the temperature-dependent ^{1}H NMR spectra (600 MHz, $CDCl_3$) of 2b. Asterisk indicates trace solvent peaks. .... SI 37

Figure S2. UV (upper panel) and ECD (lower panel) spectra of 1a measured in cyclohexane (solid black lines) and acetonitrile (blue dashed lines). .... SI 38

Figure S3. UV (upper panel) and ECD (lower panel) spectra of 1b measured in cyclohexane (solid black lines) and acetonitrile (blue dashed lines). .... SI 39

Figure S4. UV (upper panel) and ECD (lower panel) spectra of 2a measured in cyclohexane (solid black lines) and acetonitrile (blue dashed lines). .... SI 40

Figure S5. UV (upper panel) and ECD (lower panel) spectra of 2b measured in cyclohexane (solid black lines) and acetonitrile (blue dashed lines). .... SI 41

Figure S6. UV (upper panel) and ECD (lower panel) spectra of 3 measured in dichloromethane ~~acetonitrile~~ (blue dashed lines). .... SI 42

Figure S7. UV (upper panel) and ECD (lower panel) spectra of 4 measured in cyclohexane (solid black lines) and acetonitrile (blue dashed lines). .... SI 43

Figure S8. UV (upper panel) and ECD (lower panel) spectra of 5a measured in cyclohexane (solid black lines) and acetonitrile (blue dashed lines). .... SI 44

Figure S9. UV (upper panel) and ECD (lower panel) spectra of 5b measured in cyclohexane (solid black lines) and acetonitrile (blue dashed lines). ....SI 45

Figure S10. UV (upper panel) and ECD (lower panel) spectra of 6a measured in cyclohexane (solid black lines) and acetonitrile (blue dashed lines). ....SI 46

Figure S11. UV (upper panel) and ECD (lower panel) spectra of 6b measured in cyclohexane (solid black lines) and acetonitrile (blue dashed lines). ....SI 47

Figure S12. UV (upper panel) and ECD (lower panel) spectra of 7a measured in cyclohexane (solid black lines) and acetonitrile (blue dashed lines). ....SI 48

Figure S13. UV (upper panel) and ECD (lower panel) spectra of 7b measured in cyclohexane (solid black lines) and acetonitrile (blue dashed lines). ....SI 49

Figure S14. UV (upper panel) and ECD (lower panel) spectra of 8a measured in cyclohexane (solid black lines) and acetonitrile (blue dashed lines). ....SI 50

Figure S15. UV (upper panel) and ECD (lower panel) spectra of 8b measured in cyclohexane (solid black lines) and dichloromethane (blue dashed lines). ....SI 51

Figure S16. UV (upper panel) and ECD (lower panel) spectra of 9a measured in cyclohexane (solid black lines) and acetonitrile (blue dashed lines). ....SI 52

Figure S17. UV (upper panel) and ECD (lower panel) spectra of 9b measured in dichloromethane (blue dashed lines). ....SI 53

Figure S18. UV (upper panel) and ECD (lower panel) spectra of 10a measured in cyclohexane (solid black lines) and acetonitrile (blue dashed lines). ....SI 54

Figure S19. UV (upper panel) and ECD (lower panel) spectra of 10b measured in cyclohexane (solid black lines) and acetonitrile (blue dashed lines). ....SI 55

Figure S20. UV (upper panel) and ECD (lower panel) spectra of 11a measured in cyclohexane (solid black lines) and acetonitrile (blue dashed lines). ....SI 56

Figure S21. UV (upper panel) and ECD (lower panel) spectra of 11b measured in cyclohexane (solid black lines) and acetonitrile (blue dashed lines). ....SI 57

Figure S22. UV (upper panel) and ECD (lower panel) spectra of 12a measured in cyclohexane (solid black lines) and acetonitrile (blue dashed lines). ....SI 58

Figure S23. UV (upper panel) and ECD (lower panel) spectra of 12b measured in cyclohexane (solid black lines) and acetonitrile (blue dashed lines). ....SI 59

Figure S24. Structures of individual low-energy conformers of 1a calculated at the B3LYP/6-311G(d,p) level. ....SI 60

Figure S25. Structures of individual low-energy conformers of 1a calculated at the IEFPCM(ACN)/B3LYP/6-311G(d,p) level. ....SI 61

Figure S26. Structures of individual low-energy conformers of 1b calculated at the B3LYP/6-311G(d,p) level. ....SI 62

Figure S27. Structures of individual low-energy conformers of 3 calculated at the B3LYP/6-311G(d,p) level. ....SI 63

Figure S28. Structures of individual low-energy conformers of 3 calculated at the IEFPCM(CH_2Cl_2)/B3LYP/6-311G(d,p) level. ....SI 64

Figure S29. Structures of individual low-energy conformers of 4 calculated at the B3LYP/6-311G(d,p) level. ....SI 65

Figure S30. Structures of individual low-energy conformers of 4 calculated at the IEFPCM(ACN)/B3LYP/6-311G(d,p) level. ....SI 66

Figure S31. Structures of individual low-energy conformers of 5a calculated at the B3LYP/6-311G(d,p) level. ....SI 67

Figure S32. Structures of individual low-energy conformers of 5a calculated at the IEFPCM(ACN)/B3LYP/6-311G(d,p) level. ....SI 68

Figure S33. Structures of individual low-energy conformers of 5b calculated at the B3LYP/6-311G(d,p) level. ....SI 69

Figure S34. Structures of individual low-energy conformers of 9a calculated at the B3LYP/6-311G(d,p) level. ....SI 70

Figure S35. Structures of individual low-energy conformers of 9a calculated at the IEFPCM(ACN)/B3LYP/6-311G(d,p) level. ....SI 71

Figure S36. Structures of individual low-energy conformers of 9b calculated at the B3LYP/6-311G(d,p) level. ....SI 72

Figure S37. Structures of individual low-energy conformers of 9b calculated at the IEFPCM(CH_2Cl_2)/B3LYP/6-311G(d,p) level. ....SI 73

Figure S38. Overlaid of the structures of individual low-energy conformers of 1a calculated at the DFT/6-311G(d,p) level of theory: *in vacuo* (black color) and with the use of acetonitrile solvent model (green color). ....SI 74

Figure S39. Overlaid of the structures of individual low-energy conformers of 3 calculated at the DFT/6-311G(d,p) level of theory: *in vacuo* (black color) and with the use of dichloromethane solvent model (blue color). ....SI 74

Figure S40. Overlaid of the structures of individual low-energy conformers of 4 calculated at the DFT/6-311G(d,p) level of theory: *in vacuo* (black color) and with the use of acetonitrile solvent model (green color). ....SI 75

Figure S41. Overlaid of the structures of individual low-energy conformers of 5a calculated at the DFT/6-311G(d,p) level of theory: *in vacuo* (black color) and with the use of acetonitrile solvent model (green color). ....SI 76

Figure S42. Overlaid of the structures of individual low-energy conformers of 9a calculated at the DFT/6-311G(d,p) level of theory: *in vacuo* (black color) and with the use of acetonitrile solvent model (green color). ....SI 76

Figure S43. Overlaid of the structures of individual low-energy conformers of 9b calculated at the DFT/6-311G(d,p) level of theory: *in vacuo* (black color) and with the use of dichloromethane solvent model (blue color). ....SI 77

Figure S44. Calculated at the TD-CAM-B3LYP/6-311++G(d,p) level UV (upper panel) and ECD (lower panel) spectra of model compounds 13a (left) and 13b (right). Wavelengths were not corrected. Vertical bars represent oscillator or rotator strengths, respectively. .... SI 78

Figure S45. Calculated at the TD-CAM-B3LYP/6-311++G(d,p) level UV (upper panel) and ECD (lower panel) spectra of model compounds 14a (left) and 14b (right). Wavelengths were not corrected. Vertical bars represent oscillator or rotator strengths, respectively. .... SI 79

Figure S46. Calculated at the TD-CAM-B3LYP/6-311++G(d,p) level UV (upper panels) and ECD (lower panels) spectra of individual conformers of model compound 15. Wavelengths were not corrected. Vertical bars represent oscillator or rotator strengths, respectively. The values at top right corner of each UV spectrum indicated the biphenyl moiety twist angle. .... SI 81

Figure S47. Values of the first three low-energy Cotton effects of model compound 15, as a function of torsion angle ω, calculated at the TD-CAM-B3LYP/6-311++G(d,p) level of theory. .... SI 82

Figure S48. UV (upper panel) and ECD (lower panel) spectra of 1a measured in cyclohexane (solid black lines) and calculated at the TD-CAM-B3LYP/6-311++G(d,p) level. The calculated ECD spectra were Boltzmann-averaged based on ΔE (red lines) and $\Delta\Delta$G values (blue lines). Wavelengths were corrected to match the experimental UV maxima. The insert shows the comparison between the ECD spectra calculated for the lowest energy conformer of a given compound (dashed blue lines) and the $\Delta\Delta$G-based and Boltzmann averaged (solid blue lines). .... SI 83

Figure S49. UV (upper panel) and ECD (lower panel) spectra of 1a measured in cyclohexane (solid black lines) and calculated at the TD-M06-2X/6-311++G(d,p) level. The calculated ECD spectra were Boltzmann-averaged based on ΔE (red lines) and $\Delta\Delta$G values (blue lines). Wavelengths were corrected to match the experimental UV maxima. The insert shows the comparison between the ECD spectra calculated for the lowest energy conformer of a given compound (dashed blue lines) and the $\Delta\Delta$G-based and Boltzmann averaged (solid blue lines). .... SI 84

Figure S50. UV (upper panel) and ECD (lower panel) spectra of 1a measured in acetonitrile (solid black lines) and calculated at the IEFPCM/TD-CAM-B3LYP/6-311++G(d,p) level. The calculated ECD spectra were Boltzmann-averaged based on ΔE (red lines) and $\Delta\Delta$G values (blue lines). Wavelengths were corrected to match the experimental UV maxima. The insert shows the comparison between the ECD spectra calculated for the lowest energy conformer of a given compound (dashed blue lines) and the $\Delta\Delta$G-based and Boltzmann averaged (solid blue lines). .... SI 85

Figure S51. UV (upper panel) and ECD (lower panel) spectra of 1a measured in acetonitrile (solid black lines) and calculated at the IEFPCM/TD-M06-2X/6-311++G(d,p) level. The calculated ECD spectra were Boltzmann-averaged based on ΔE (red lines) and $\Delta\Delta$G values (blue lines). Wavelengths were corrected to match the experimental UV maxima. The insert shows the comparison between the ECD spectra calculated for the lowest energy conformer of a given compound (dashed blue lines) and the $\Delta\Delta$G-based and Boltzmann averaged (solid blue lines). .... SI 86

Figure S52. UV (upper panel) and ECD (lower panel) spectra of 1b measured in cyclohexane (solid black lines) and calculated at the TD-CAM-B3LYP/6-311++G(d,p) level. The calculated ECD spectra were Boltzmann-averaged based on ΔE (red lines) and $\Delta\Delta$G values (blue lines). Wavelengths were corrected to match the experimental UV maxima. The insert shows the comparison between the ECD spectra calculated for the lowest energy conformer of a given compound (dashed blue lines) and the $\Delta\Delta$G-based and Boltzmann averaged (solid blue lines). .... SI 87

Figure S53. UV (upper panel) and ECD (lower panel) spectra of 1b measured in cyclohexane (solid black lines) and calculated at the TD-M06-2X/6-311++G(d,p) level. The calculated ECD spectra were Boltzmann-averaged based on ΔE (red lines) and $\Delta\Delta$G values (blue lines). Wavelengths were corrected to match the experimental UV maxima. The insert shows the comparison between the ECD spectra calculated for the lowest energy conformer of a given compound (dashed blue lines) and the $\Delta\Delta$G-based and Boltzmann averaged (solid blue lines). .... SI 88

Figure S54. UV (upper panel) and ECD (lower panel) spectra of 3 measured in dichloromethane (solid black lines) and calculated at the TD-CAM-B3LYP/6-311++G(d,p) level. The calculated ECD spectra were Boltzmann-averaged based on ΔE (red lines) and $\Delta\Delta$G values (blue lines). Wavelengths were corrected to match the experimental UV maxima. The insert shows the comparison between the ECD spectra calculated for the lowest energy conformer of a given compound (dashed blue lines) and the $\Delta\Delta$G-based and Boltzmann averaged (solid blue lines). .... SI 89

Figure S55. UV (upper panel) and ECD (lower panel) spectra of 3 measured in dichloromethane (solid black lines) and calculated at the TD-M06-2X/6-311++G(d,p) level. The calculated ECD spectra were Boltzmann-averaged based on ΔE (red lines) and $\Delta\Delta$G values (blue lines). Wavelengths were corrected to match the experimental UV maxima. The insert shows the comparison between the ECD spectra calculated for the lowest energy conformer of a given compound (dashed blue lines) and the $\Delta\Delta$G-based and Boltzmann averaged (solid blue lines). .... SI 90

Figure S56. UV (upper panel) and ECD (lower panel) spectra of 3 measured in dichloromethane (solid black lines) and calculated at the IEFPCM/TD-CAM-B3LYP/6-311++G(d,p) level. The calculated ECD spectra were Boltzmann-averaged based on ΔE (red lines) and $\Delta\Delta$G values (blue lines). Wavelengths were corrected to match the experimental UV maxima. The insert shows the comparison between the ECD spectra calculated for the lowest energy conformer of a given compound (dashed blue lines) and the $\Delta\Delta$G-based and Boltzmann averaged (solid blue lines). .... SI 91

Figure S57. UV (upper panel) and ECD (lower panel) spectra of 3 measured in dichloromethane (solid black lines) and calculated at the IEFPCM/TD-M06-2X/6-311++G(d,p) level. The calculated ECD spectra were Boltzmann-averaged based on ΔE (red lines) and $\Delta\Delta$G values (blue lines). Wavelengths were corrected to match the experimental UV maxima. The insert shows the comparison between the ECD spectra calculated for the lowest energy conformer of a given compound (dashed blue lines) and the $\Delta\Delta$G-based and Boltzmann averaged (solid blue lines). .... SI 92

Figure S58. UV (upper panel) and ECD (lower panel) spectra of 4 measured in cyclohexane (solid black lines) and calculated at the TD-CAM-B3LYP/6-311++G(d,p) level. The calculated ECD spectra were Boltzmann-averaged based on ΔE (red lines) and $\Delta\Delta$G values (blue lines). Wavelengths were corrected to match the experimental UV maxima. The insert shows the comparison between the ECD spectra calculated for the lowest energy conformer of a given compound (dashed blue lines) and the $\Delta\Delta$G-based and Boltzmann averaged (solid blue lines). .... SI 93

Figure S59. UV (upper panel) and ECD (lower panel) spectra of 4 measured in cyclohexane (solid black lines) and calculated at the TD-M06-2X/6-311++G(d,p) level. The calculated ECD spectra were Boltzmann-averaged based on ΔE (red lines) and $\Delta\Delta$G values (blue lines). Wavelengths were corrected to match the experimental UV maxima. The insert shows the comparison between the ECD spectra calculated for the lowest energy conformer of a given compound (dashed blue lines) and the $\Delta\Delta$G-based and Boltzmann averaged (solid blue lines). .... SI 94

Figure S60. UV (upper panel) and ECD (lower panel) spectra of 4 measured in acetonitrile (solid black lines) and calculated at the IEFPCM/TD-CAM-B3LYP/6-311++G(d,p) level. The calculated ECD spectra were Boltzmann-averaged based on ΔE (red lines) and $\Delta\Delta$G values (blue lines). Wavelengths were corrected to match the experimental UV maxima. The insert shows the comparison between the ECD spectra calculated for the lowest energy conformer of a given compound (dashed blue lines) and the $\Delta\Delta$G-based and Boltzmann averaged (solid blue lines). ....SI 95

Figure S61. UV (upper panel) and ECD (lower panel) spectra of 4 measured in acetonitrile (solid black lines) and calculated at the IEFPCM/TD-M06-2X/6-311++G(d,p) level. The calculated ECD spectra were Boltzmann-averaged based on ΔE (red lines) and $\Delta\Delta$G values (blue lines). Wavelengths were corrected to match the experimental UV maxima. The insert shows the comparison between the ECD spectra calculated for the lowest energy conformer of a given compound (dashed blue lines) and the $\Delta\Delta$G-based and Boltzmann averaged (solid blue lines). ....SI 96

Figure S62. UV (upper panel) and ECD (lower panel) spectra of 5a measured in cyclohexane (solid black lines) and calculated at the TD-CAM-B3LYP/6-311++G(d,p) level. The calculated ECD spectra were Boltzmann-averaged based on ΔE (red lines) and $\Delta\Delta$G values (blue lines). Wavelengths were corrected to match the experimental UV maxima. The insert shows the comparison between the ECD spectra calculated for the lowest energy conformer of a given compound (dashed blue lines) and the $\Delta\Delta$G-based and Boltzmann averaged (solid blue lines). ....SI 97

Figure S63. UV (upper panel) and ECD (lower panel) spectra of 5a measured in cyclohexane (solid black lines) and calculated at the TD-M06-2X/6-311++G(d,p) level. The calculated ECD spectra were Boltzmann-averaged based on ΔE (red lines) and $\Delta\Delta$G values (blue lines). Wavelengths were corrected to match the experimental UV maxima. The insert shows the comparison between the ECD spectra calculated for the lowest energy conformer of a given compound (dashed blue lines) and the $\Delta\Delta$G-based and Boltzmann averaged (solid blue lines). ....SI 98

Figure S64. UV (upper panel) and ECD (lower panel) spectra of 5a measured in acetonitrile (solid black lines) and calculated at the IEFPCM/TD-CAM-B3LYP/6-311++G(d,p) level. The calculated ECD spectra were Boltzmann-averaged based on ΔE (red lines) and $\Delta\Delta$G values (blue lines). Wavelengths were corrected to match the experimental UV maxima. The insert shows the comparison between the ECD spectra calculated for the lowest energy conformer of a given compound (dashed blue lines) and the $\Delta\Delta$G-based and Boltzmann averaged (solid blue lines). ....SI 99

Figure S65. UV (upper panel) and ECD (lower panel) spectra of 5a measured in acetonitrile (solid black lines) and calculated at the IEFPCM/TD-M06-2X/6-311++G(d,p) level. The calculated ECD spectra were Boltzmann-averaged based on ΔE (red lines) and $\Delta\Delta$G values (blue lines). Wavelengths were corrected to match the experimental UV maxima. The insert shows the comparison between the ECD spectra calculated for the lowest energy conformer of a given compound (dashed blue lines) and the $\Delta\Delta$G-based and Boltzmann averaged (solid blue lines). ....SI 100

Figure S66. UV (upper panel) and ECD (lower panel) spectra of 5b measured in cyclohexane (solid black lines) and calculated at the TD-CAM-B3LYP/6-311++G(d,p) level. The calculated ECD spectra were Boltzmann-averaged based on ΔE (red lines) and $\Delta\Delta$G values (blue lines). Wavelengths were corrected to match the experimental UV maxima. The insert shows the comparison between the ECD spectra calculated for the lowest energy conformer of a given compound (dashed blue lines) and the $\Delta\Delta$G-based and Boltzmann averaged (solid blue lines). ....SI 101

Figure S67. UV (upper panel) and ECD (lower panel) spectra of 5b measured in cyclohexane (solid black lines) and calculated at the TD-M06-2X/6-311++G(d,p) level. The calculated ECD spectra were Boltzmann-averaged based on ΔE (red lines) and $\Delta\Delta$G values (blue lines). Wavelengths were corrected to match the experimental UV maxima. The insert shows the comparison between the ECD spectra calculated for the lowest energy conformer of a given compound (dashed blue lines) and the $\Delta\Delta$G-based and Boltzmann averaged (solid blue lines). ....SI 102

Figure S68. UV (upper panel) and ECD (lower panel) spectra of 9a measured in cyclohexane (solid black lines) and calculated at the TD-CAM-B3LYP/6-311++G(d,p) level. The calculated ECD spectra were Boltzmann-averaged based on ΔE (red lines) and $\Delta\Delta$G values (blue lines). Wavelengths were corrected to match the experimental UV maxima. The insert shows the comparison between the ECD spectra calculated for the lowest energy conformer of a given compound (dashed blue lines) and the $\Delta\Delta$G-based and Boltzmann averaged (solid blue lines). ....SI 103

Figure S69. UV (upper panel) and ECD (lower panel) spectra of 9a measured in cyclohexane (solid black lines) and calculated at the TD-M06-2X/6-311++G(d,p) level. The calculated ECD spectra were Boltzmann-averaged based on ΔE (red lines) and $\Delta\Delta$G values (blue lines). Wavelengths were corrected to match the experimental UV maxima. The insert shows the comparison between the ECD spectra calculated for the lowest energy conformer of a given compound (dashed blue lines) and the $\Delta\Delta$G-based and Boltzmann averaged (solid blue lines). ....SI 104

Figure S70. UV (upper panel) and ECD (lower panel) spectra of 9a measured in acetonitrile (solid black lines) and calculated at the IEFPCM/TD-CAM-B3LYP/6-311++G(d,p) level. The calculated ECD spectra were Boltzmann-averaged based on ΔE (red lines) and $\Delta\Delta$G values (blue lines). Wavelengths were corrected to match the experimental UV maxima. The insert shows the comparison between the ECD spectra calculated for the lowest energy conformer of a given compound (dashed blue lines) and the $\Delta\Delta$G-based and Boltzmann averaged (solid blue lines). ....SI 105

Figure S71. UV (upper panel) and ECD (lower panel) spectra of 9a measured in acetonitrile (solid black lines) and calculated at the IEFPCM/TD-M06-2X/6-311++G(d,p) level. The calculated ECD spectra were Boltzmann-averaged based on ΔE (red lines) and $\Delta\Delta$G values (blue lines). Wavelengths were corrected to match the experimental UV maxima. The insert shows the comparison between the ECD spectra calculated for the lowest energy conformer of a given compound (dashed blue lines) and the $\Delta\Delta$G-based and Boltzmann averaged (solid blue lines). ....SI 106

Figure S72. UV (upper panel) and ECD (lower panel) spectra of 9b measured in dichloromethane (solid black lines) and calculated at the TD-CAM-B3LYP/6-311++G(d,p) level. The calculated ECD spectra were Boltzmann-averaged based on ΔE (red lines) and $\Delta\Delta$G values (blue lines). Wavelengths were corrected to match the experimental UV maxima. The insert shows the comparison between the ECD spectra calculated for the lowest energy conformer of a given compound (dashed blue lines) and the $\Delta\Delta$G-based and Boltzmann averaged (solid blue lines). ....SI 107

Figure S73. UV (upper panel) and ECD (lower panel) spectra of 9b measured in dichloromethane (solid black lines) and calculated at the TD-M06-2X/6-311++G(d,p) level. The calculated ECD spectra were Boltzmann-averaged based on ΔE (red lines) and $\Delta\Delta$G values (blue lines). Wavelengths were corrected to match the experimental UV maxima. The insert shows the comparison between the ECD spectra calculated for the lowest energy conformer of a given compound (dashed blue lines) and the $\Delta\Delta$G-based and Boltzmann averaged (solid blue lines). ....SI 108

Figure S74. UV (upper panel) and ECD (lower panel) spectra of 9b measured in dichloromethane (solid black lines) and calculated at the IEFPCM/TD-CAM-B3LYP/6-311++G(d,p) level. The calculated ECD spectra were Boltzmann-averaged based on ΔE (red lines) and $\Delta\Delta G$ values (blue lines). Wavelengths were corrected to match the experimental UV maxima. The insert shows the comparison between the ECD spectra calculated for the lowest energy conformer of a given compound (dashed blue lines) and the $\Delta\Delta G$-based and Boltzmann averaged (solid blue lines). ....SI 109

Figure S75. UV (upper panel) and ECD (lower panel) spectra of 9b measured in dichloromethane (solid black lines) and calculated at the IEFPCM/TD-M06-2X/6-311++G(d,p) level. The calculated ECD spectra were Boltzmann-averaged based on ΔE (red lines) and $\Delta\Delta G$ values (blue lines). Wavelengths were corrected to match the experimental UV maxima. The insert shows the comparison between the ECD spectra calculated for the lowest energy conformer of a given compound (dashed blue lines) and the $\Delta\Delta G$-based and Boltzmann averaged (solid blue lines)..SI 110

Figure S76. UV (upper panel) and ECD (lower panel) spectra of individual conformers of 1a calculated at the TD-CAM-B3LYP/6-311++G(d,p) level. Wavelengths were not corrected, vertical bars represent rotator strengths. ....SI 111

Figure S77. UV (upper panel) and ECD (lower panel) spectra of individual conformers of 1a calculated at the TD-M06-2X/6-311++G(d,p) level. Wavelengths were not corrected, vertical bars represent rotator strengths. ....SI 111

Figure S78. UV (upper panel) and ECD (lower panel) spectra of individual conformers of 1a calculated at the IEFPCM/TD-CAM-B3LYP/6-311++G(d,p) level. Wavelengths were not corrected, vertical bars represent rotator strengths. ....SI 112

Figure S79. UV (upper panel) and ECD (lower panel) spectra of individual conformers of 1a calculated at the IEFPCM/TD-M06-2X/6-311++G(d,p) level. Wavelengths were not corrected, vertical bars represent rotator strengths. ....SI 112

Figure S80. UV (upper panel) and ECD (lower panel) spectra of individual conformers of 1b calculated at the TD-CAM-B3LYP/6-311++G(d,p) level. Wavelengths were not corrected, vertical bars represent rotator strengths. ....SI 113

Figure S81. UV (upper panel) and ECD (lower panel) spectra of individual conformers of 1b calculated at the TD-M06-2X/6-311++G(d,p) level. Wavelengths were not corrected, vertical bars represent rotator strengths. ....SI 113

Figure S82. UV (upper panel) and ECD (lower panel) spectra of individual conformers of 3 calculated at the TD-CAM-B3LYP/6-311++G(d,p) level. Wavelengths were not corrected, vertical bars represent rotator strengths. ....SI 114

Figure S83. UV (upper panel) and ECD (lower panel) spectra of individual conformers of 3 calculated at the TD-M06-2X/6-311++G(d,p) level. Wavelengths were not corrected, vertical bars represent rotator strengths..SI 114

Figure S84. UV (upper panel) and ECD (lower panel) spectra of individual conformers of 3 calculated at the IEFPCM/TD-CAM-B3LYP/6-311++G(d,p) level. Wavelengths were not corrected, vertical bars represent rotator strengths. ....SI 115

Figure S85. UV (upper panel) and ECD (lower panel) spectra of individual conformers of 3 calculated at the IEFPCM/TD-M06-2X/6-311++G(d,p) level. Wavelengths were not corrected, vertical bars represent rotator strengths. ....SI 115

Figure S86. UV (upper panel) and ECD (lower panel) spectra of individual conformers of 4 calculated at the TD-CAM-B3LYP/6-311++G(d,p) level. Wavelengths were not corrected, vertical bars represent rotator strengths. ....SI 116

Figure S87. UV (upper panel) and ECD (lower panel) spectra of individual conformers of 4 calculated at the TD-M06-2X/6-311++G(d,p) level. Wavelengths were not corrected, vertical bars represent rotator strengths. ....SI 117

Figure S88. UV (upper panel) and ECD (lower panel) spectra of individual conformers of 4 calculated at the IEFPCM/TD-CAM-B3LYP/6-311++G(d,p) level. Wavelengths were not corrected, vertical bars represent rotator strengths. ....SI 118

Figure S89. UV (upper panel) and ECD (lower panel) spectra of individual conformers of 4 calculated at the IEFPCM/TD-M06-2X/6-311++G(d,p) level. Wavelengths were not corrected, vertical bars represent rotator strengths. ....SI 119

Figure S90. UV (upper panel) and ECD (lower panel) spectra of individual conformers of 5a calculated at the TD-CAM-B3LYP/6-311++G(d,p) level. Wavelengths were not corrected, vertical bars represent rotator strengths. ....SI 120

Figure S91. UV (upper panel) and ECD (lower panel) spectra of individual conformers of 5a calculated at the TD-M06-2X/6-311++G(d,p) level. Wavelengths were not corrected, vertical bars represent rotator strengths. ....SI 120

Figure S92. UV (upper panel) and ECD (lower panel) spectra of individual conformers of 5a calculated at the IEFPCM/TD-CAM-B3LYP/6-311++G(d,p) level. Wavelengths were not corrected, vertical bars represent rotator strengths. ....SI 121

Figure S93. UV (upper panel) and ECD (lower panel) spectra of individual conformers of 5a calculated at the IEFPCM/TD-M06-2X/6-311++G(d,p) level. Wavelengths were not corrected, vertical bars represent rotator strengths. ....SI 121

Figure S94. UV (upper panel) and ECD (lower panel) spectra of individual conformers of 5b calculated at the TD-CAM-B3LYP/6-311++G(d,p) level. Wavelengths were not corrected, vertical bars represent rotator strengths. ....SI 122

Figure S95. UV (upper panel) and ECD (lower panel) spectra of individual conformers of 5b calculated at the TD-M06-2X/6-311++G(d,p) level. Wavelengths were not corrected, vertical bars represent rotator strengths. ....SI 122

Figure S96. UV (upper panel) and ECD (lower panel) spectra of individual conformers of 9a calculated at the TD-CAM-B3LYP/6-311++G(d,p) level. Wavelengths were not corrected, vertical bars represent rotator strengths. 123

Figure S97. UV (upper panel) and ECD (lower panel) spectra of individual conformers of 9a calculated at the TD-M06-2X/6-311++G(d,p) level. Wavelengths were not corrected, vertical bars represent rotator strengths. ....SI 123

Figure S98. UV (upper panel) and ECD (lower panel) spectra of individual conformers of 9a calculated at the IEFPCM/TD-CAM-B3LYP/6-311++G(d,p) level. Wavelengths were not corrected, vertical bars represent rotator strengths. ....SI 124

Figure S99. UV (upper panel) and ECD (lower panel) spectra of individual conformers of 9a calculated at the IEFPCM/TD-M06-2X/6-311++G(d,p) level. Wavelengths were not corrected, vertical bars represent rotator strengths. ....SI 124

Figure S100. UV (upper panel) and ECD (lower panel) spectra of individual conformers of 9b calculated at the TD-CAM-B3LYP/6-311++G(d,p) level. Wavelengths were not corrected, vertical bars represent rotator strengths. ....SI 125

Figure S101. UV (upper panel) and ECD (lower panel) spectra of individual conformers of 9b calculated at the TD-M06-2X/6-311++G(d,p) level. Wavelengths were not corrected, vertical bars represent rotator strengths. ....SI 125

Figure S102. UV (upper panel) and ECD (lower panel) spectra of individual conformers of 9b calculated at the IEFPCM/TD-CAM-B3LYP/6-311++G(d,p) level. Wavelengths were not corrected, vertical bars represent rotator strengths. ....SI 126

Figure S103. UV (upper panel) and ECD (lower panel) spectra of individual conformers of 9b calculated at the IEFPCM/TD-M06-2X/6-311++G(d,p) level. Wavelengths were not corrected, vertical bars represent rotator strengths. ....SI 126

Figure S104. UV (upper panel) and ECD (lower panel) spectra of individual conformers of 4 (left column), and the model compounds A (middle column) and B (right column) calculated at the TD-CAM-B3LYP/6-311++G(d,p) level. Wavelengths were not corrected, vertical bars represent rotator strengths. ....SI 127

Figure S105. Simulated UV (upper panel) and ECD (lower panel) spectra of 4, as the function of the amounts of the most abundant low-energy conformers 1 and 4 of 4. The spectra were calculated at the TD-CAM-B3LYP/6-311++G(d,p) level. Wavelengths were not corrected. ....SI 128

Figure S106. UV (upper panel) and ECD (lower panel) spectra of the lowest energy conformer no. 1 of 9 (left column), and the model compounds A (middle column) and B (right column), calculated at the TD-CAM-B3LYP/6-311++G(d,p) level. Wavelengths were not corrected, vertical bars represent rotator strengths. ....SI 128

Structures of urea-derived macrocycle 1a in the crystal phase ....SI 129

Figure S107. Structures of macrocycle 1a as present in the crystal form: (a) I and (b) II. Displacement ellipsoids are drawn at the 50% probability level. For clarity, only selected heteroatoms are labeled. Hydrogen atoms are represented in arbitrary radii. ....SI 129

Figure S108. Overlaid molecular structures of macrocycle 1a from 1a_I (orange) and 1a_II (purple) in the crystal forms. Hydrogen atoms are omitted for clarity. ....SI 130

Figure S109. Packing diagrams of host molecules of 1a_I (a) and 1a_II (b) displayed as sticks and guest molecules displayed as spacefills. Asymmetry independent solvent molecules are distinguished by different colors. Solvent molecules are represented in the highest occupation rate. Hydrogen atoms are omitted for clarity. Views along [100] and [010] directions (upper and lower panel, respectively). ....SI 130

Figure S110. Supramolecular assembly in the crystal of 1a_II. Selected hydrogen bonds are marked in blue dash lines. .... SI 131

Structures of thiourea-derived macrocycle 1b in the crystal phase .... SI 132

Figure S111. Structure of macrocycle 1b as present in the crystal form II. Displacement ellipsoids are drawn at the 50% probability level. For clarity, only selected heteroatoms are labeled. Hydrogen atoms are represented in arbitrary radii. .... SI 132

Figure S112. Overlaid molecular structures of macrocycle 1b from 1b_I (red) [10] and 1b_II (blue) in the crystal phase. Hydrogen atoms are omitted for clarity. .... SI 133

Figure S113. Packing diagram of host molecules of 1b_II displayed as sticks and guest molecules displayed as spacefills. Modelled, but disordered chloroform molecules are presented in one arbitrarily selected position. For illustration, disordered solvent molecules, which their unresolved electron density was treated with PLATON/SQUEEZE, were presented in blue color. Hydrogen atoms molecules are omitted for clarity. View along [100] direction. .... SI 133

Figure S114. Supramolecular assembly in the crystal 1b_II. Selected hydrogen bonds are marked in pink (C-H···S) and blue (C-H···C) dash lines. Halogen bonds are marked in dark-blue (C-Cl···S) lines. ..... SI 134

Structure of thiourea-derived macrocycle 3 in the crystal phase .... SI 135

Figure S115. Structures of asymmetry independent molecules of macrocycle 3 in the crystal. Displacement ellipsoids are drawn at the 50% probability level. For clarity, only selected heteroatoms are labeled. Hydrogen atoms are represented in arbitrary radii. .... SI 135

Figure S116. (a) Homochiral isomers of 3 with different helical arrangement of bromine atoms (views on the top of molecules). Helical arrangement of bromine atoms is distinguished by red and blue arrows. Short dipole–dipole contacts between bromine atoms are marked in green, and between carbon and bromine atoms – in orange dashed lines. (b) Overlaid molecular structures of diastereoisomers of 3. Isomers are distinguished in blue and red colors. Hydrogen atoms are omitted for clarity. .... SI 136

Figure S117. (a) Two diastereoisomers of 3 – the arrows indicate bromine atoms' ”twisting” directions (views on the top of molecules). (b) Packing diagrams of host molecules displayed as sticks. Isomers have been marked with different colors. Views along [100], [010] and [001] directions. Hydrogen atoms are omitted for clarity. .... SI 137

Figure S118. Packing diagrams of host molecules of 3b displayed as sticks and guest molecules displayed as spacefills. For illustration, disordered solvent molecules, which their unresolved electron density was treated with PLATON/SQUEEZE, were presented in blue color. Hydrogen atoms are omitted for clarity. Views along (a) [100] and (b) [010] directions. .... SI 138

Figure S119. Supramolecular chain created in crystal 3. Halogen bonds are marked in blue (C-Cl···S) and green (C-Br···S). .... SI 139

Figure S120. Supramolecular columnar assembly created in the crystal of 3. Hydrogen bonds are marked in dark-blue (C-H···Br) and pink (C-H···S). .... SI 140

Structure of thiourea-derived macrocycle 8b in the crystal phase .... SI 141

Figure S121. Structure of macrocycle 8b in the crystal. Displacement ellipsoids are drawn at the 50% probability level. For clarity, only selected heteroatoms are labeled. Hydrogen atoms are represented

in arbitrary radii. The ratio of site occupation factors for two components of the disordered cyclohexane group, is 50% : 50%. ....SI 141

Figure S122. Arrangement of molecule 8b in the crystal and measured distances between thiourea groups: S1···C16 and S2···C01 (marked in red dashed lines). ....SI 142

Figure S123. Packing diagram of host molecules of 8b displayed as sticks and guest molecules displayed as spacefills. Hydrogen atoms are omitted for clarity. View along [010] direction. ....SI 142

Figure S124. Supramolecular chain created around 2_1 screw axis in the crystal of 8b. Selected hydrogen bonds are marked in pink (C-H···S); and halogen bonds – in blue (C-Cl···Br) and green (C-Br···S) lines. View along [010] direction. ....SI 143

Figure S125. Various of columnar systems of host macrocyclic molecules in the crystals: a zipper motif created by two columnar stacks (distinguished in different colors) in (a) 1a_II and (b) 8b; a single columnar stack in (c) 1b_II and (d) 3. Macrocycles are drawn as van der Waals spheres. ....SI 144

Figure S126. Structure of bridged trianglimine in the crystal.[31] Macrocycle is drawn with van der Waals spheres. ....SI 144

Figure S127. Various of structural voids and channels created by host macrocyclic molecules in crystals: 1a_I, 1a_II, 1b_I,[10] 1b_II, 3, and 8b. Estimated percentages of unit cell volume occupied by voids in unit cell are reported in parentheses. Probe radius: 1.5 Å. Host molecules are represented in sticks style; hydrogen atoms and solvent molecules are omitted for clarity. ....SI 145

Hirshfeld surface analysis ....SI 146

Figure S128. Percentage contributions of various intermolecular contacts to the molecular Hirshfeld surface of macrocycles 1a_I, 1a_II, 1b_II, 3 and 8b. ....SI 146

Figure S129. Results of Hirshfeld surface analysis (normalized Hirshfeld surfaces d_{norm} and selected fingerprint plots) for 1a_I, 1a_II, 1b_II and 8b. ....SI 147

Figure S130. Results of Hirshfeld surface analysis (normalized Hirsfeld surfaces d_{norm} and selected fingerprint plots) for 3. ....SI 148

Figure S131. Overlaid molecular structures of urea and thiourea derivatives 1a, 1b, 3 and 8b, found in the crystal phase (green color) and fully optimized at the B3LYP/6-311G(d,p) level. The optimization procedures were utilized starting geometries found in the respective crystals. ....SI 149

Copies of ^{1}H and ^{13}C NMR spectra ....SI 150

Cartesian coordinates..SI 174

References ....SI 439

Experimental section

All commercially available reagents were obtained from commercial suppliers and, unless specified otherwise, used in reactions without further purification. The anhydrous dichloromethane and chloroform were distilled over calcium hydride under an inert atmosphere. Flash column chromatography was performed on Merck Kieselgel type 60 (250-400 mesh). Merck Kieselgel type $60F_{254}$ analytical plates were used for TLC analysis.

^{1}H and ^{13}C NMR spectra were recorded on a Bruker 400 MHz or Bruker 600 MHz at ambient or at low temperature. All NMR spectra are reported in parts per million (ppm) downfield of TMS and were measured relative to the signals for residual $CDCl_3$ (7.27 ppm and 77.0 ppm, respectively for ^{1}H and ^{13}C NMR spectra). All ^{13}C NMR spectra were obtained with ^{1}H decoupling. Mass spectra were recorded on AB Sciex TripleTOF® 5600+ System. Melting points were measured by using open glass capillaries in a Büchi Melting Point B-545 apparatus.

A Jasco P-2000 polarimeter was used for optical rotation measurements (at 20 °C). UV and CD spectra were recorded on a Jasco J-810 spectropolarimeter at room temperature in cyclohexane and acetonitrile. In selected cases, dichloromethane has been used as the solvent.

The UV and CD measurements have been done with the use of a quartz cell of optical lengths 0.1 cm. The concentration of analytes ranged from 1.0 to 2.0×10^{-4} mol L^{-1}. Background spectra of the pure solvents were recorded from 400 to 185 (225 nm in the case of dichloromethane) nm with the scan speed of 100 nm min^{-1}. The ECD spectra of analytes were measured with 8 accumulations.

The starting macro- and acyclic imines were obtained according to the previously published procedures.[1-10] The general synthetic route is shown in **Scheme S1**.

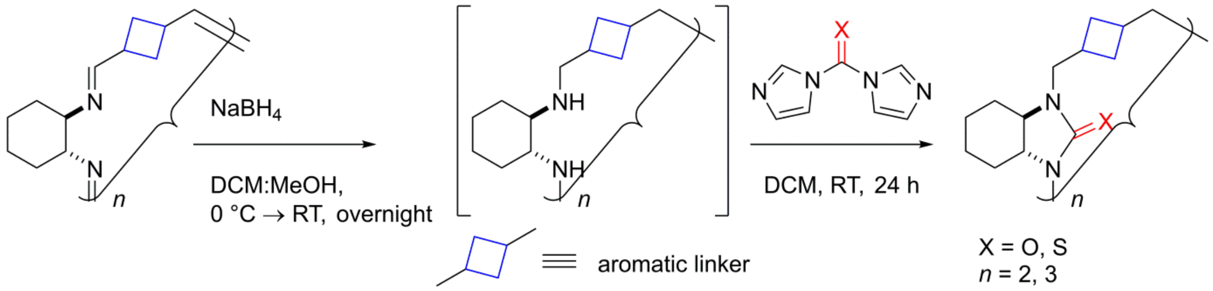

Scheme S1. General synthetic route.

General procedure for synthesis of macrocyclic urea derivatives.

To the solution of respective imine (1 mmol) in 25 mL of dichloromethane-methanol (4:1) mixture cooled to 0 °C, a solid sodium borohydride (228 mg, 6 mmol, 6 eq) was added in small portions during 15 minutes. The reaction was allowed to warm to room temperature and was being stirred overnight. The solvents were removed by evaporation and the solid residue was extracted with dichloromethane (3 × 20 mL) and water. The organic extracts were washed with brine and dried over sodium sulphate. After evaporation of the solvent, the quantitatively obtained crude product was used as received for further reactions. Respective macrocyclic amine (1 eq) and 1,1′-carbonyldiimidazole (1 eq per one diaminocyclohexane unit) were dissolved in dichloromethane and stirred for 24 h, at room temperature. Then, the mixture was washed with water and brine and dried over anhydrous sodium sulfate. After evaporation of the solvent, the crude product was purified by column chromatography on silica gel to provide the product.

Macrocycle 1a – colorless crystalline solid, (known compound) all spectra were in agreement to that previously reported.[10]

Macrocycle 2a: eluent CH_2Cl_2 to CH_2Cl_2:MeOH (96:4), yield 94 mg (57%), colorless amorphous solid,. Mp.209-210 °C; $[\alpha]_D^{20}$ –28° (*c* 1.29, $CHCl_3$); ^{1}H NMR (300 MHz, $CDCl_3$) δ 6.88 (s, 6H), 4.48 (d, *J* = 14.7 Hz, 6H), 4.22 (d, *J* = 14.7 Hz, 6H), 3.58 (s, 18H), 2.54 (d, *J* = 7.6 Hz, 6H), 1.94 (d, *J* = 10.7 Hz, 6H), 1.70 (d, *J* = 7.2 Hz, 6H), 1.19-1. 01 (m, 12H) ppm; ^{13}C{^{1}H} NMR (75 MHz, $CDCl_3$) δ 163.6, 151.5, 126.1, 114.0, 62.2, 55.9, 42.0, 28.8, 24.4 ppm; ATR-IR 2934 (CH_{2asym}), 2858 (CH_{2sym}), 1694 (C=O), 1506, 1439 (CH_{2def}), 1210 (Ar-O-C_{asym}), 1040 (Ar-O-C $_{sym}$), 774 (Ar-H_{def}) cm^{-1}; HRMS (ESI) m/z: [M + H]$^+$ calcd for $C_{51}H_{67}N_6O_9$, 907.4964; found, 907.4953.

Macrocycle 4: eluent CH_2Cl_2 to CH_2Cl_2:MeOH (99:1), yield 54 mg (48%), colorless amorphous solid,. Mp. 233-234 °C (decomposition); $[\alpha]_D^{20}$ +42° (*c* 2.68, $CHCl_3$); ^{1}H NMR (300 MHz, $CDCl_3$) δ 7.58 (d, *J* = 8.1 Hz, 12H), 7.37 (d, *J* = 8.1 Hz, 12H), 4.47 (q, *J* = 8.5, *J* = 15.4 Hz, 12H), 2.75 (bs, 6H), 1.87-1.84 (m, 6H), 1.70 (m, 6H), 1.23-1.21 (m, 12H) ppm; ^{13}C{^{1}H} NMR (75 MHz, $CDCl_3$) δ 163.7, 139.6, 137.2, 128.4, 127.0, 61.9, 46.6, 28.5, 24.1 ppm; ATR-IR 2933 (CH_{2asym}), 2862 (CH_{2sym}), 1698 (C=O), 1500, 1436 (CH_{2def}) cm^{-1}; HRMS (ESI) m/z: [M + H]$^+$ calcd for $C_{63}H_{67}N_6O_3$, 955.5269; found, 955.5286.

Macrocycle 5a – yield 163 mg (70%),colorless crystalline solid, (known compound) all spectra were in agreement to that previously reported.[10]

Macrocycle 6a: eluent CH_2Cl_2 to CH_2Cl_2:MeOH (95:5), yield 21 mg (64%), colorless amorphous solid. Mp. 165-166 °C; $[\alpha]_D^{20}$ +3.5° (*c* 1.12, $CHCl_3$), ^{1}H NMR (300 MHz, $CDCl_3$) δ 7.15 (s, 6H), 7.11 (s, 3H), 4.76 (d, *J* = 15.7 Hz, 6H), 4.12 (d, *J* = 15.7 Hz, 6H), 2.87 (d, *J* = 7.4 Hz, 6H), 1.72-1.65 (m, 13H), 1.30 (s, 27H), 1.22-1.19 (m, 11H) ppm; ^{13}C{^{1}H} NMR (75 MHz, $CDCl_3$) δ 164.0, 151.1, 138.4, 123.3, 122.7, 63.4, 47.7, 34.6, 31.4, 28.8, 24.2 ppm; ATR-IR 2935 (CH_{2asym}), 2864 (CH_{2sym}), 1699 (C=O), 1603 (C=C_{ar}), 1435 (CH_{2def})

cm^{-1}; HRMS (ESI) m/z: $[M + H]^+$ calcd for $C_{57}H_{78}N_6O_3$, 895.6208; found, 895.6215; $[M + H_2O]^+$ calcd for $C_{57}H_{80}N_6O_4$, 912.6241; found, 912.6477.

Macrocycle 7a: eluent CH_2Cl_2 to CH_2Cl_2:MeOH (95:5), yield 25 mg (11%). Mp.159-167 °C; $[\alpha]_D^{20}$ –6.3° (*c* 0.99, $CHCl_3$); ^{1}H NMR (600 MHz, $CDCl_3$) *δ* 6.88 (s, 3H), 6.71 (s, 6H), 4.68 (d, *J* = 15.6 Hz, 6H), 4.10 (d, *J* = 15.6 Hz, 6H), 3.78 (s, 9H), 2.85-2.83 (m, 6H), 1.75 (d, *J* = 9.5 Hz, 7H), 1.67 (d, *J* = 6.8 Hz, 7H),1.21-1.18 (m, 10H) ppm; ^{13}C{^{1}H} NMR (151 MHz, $CDCl_3$) *δ* 163.7, 159.7, 140.3, 118.8, 111.4, 63.1, 55.3, 47.3, 28.8, 24.1 ppm; ATR-IR 2931 (CH_{2asym}), 2855 (CH_{2sym}), 1694 (C=O), 1596 (C=C_{ar}), 1435 (CH_{2def}), 1253 (C-O-C_{def}), 1146 (C-O-C_{def}) cm^{-1}; HRMS (ESI) m/z: $[M + Na]^+$ calcd for $C_{48}H_{61}N_6O_3$, 839.4467; found, 839.4462.

Macrocycle 8a: eluent CH_2Cl_2 to CH_2Cl_2:MeOH (96:4), yield 93 mg (60%), colorless amorphous solid. Mp.192-194 °C; $[\alpha]_D^{20}$ +11.5° (*c* 1.32, $CHCl_3$); ^{1}H NMR (300 MHz, $CDCl_3$) *δ* 7.30 (s, 6H), 7.22 (s, 3H), 4.71 (d, *J* = 16.0Hz, 6H), 4.06 (d, *J* = 16.0 Hz, 6H), 2.88 (d, *J* = 7.6 Hz, 6H), 1.82-1.68 (m, 12H), 1.33-1.17 (m, 12H) ppm; ^{13}C{^{1}H} NMR (75 MHz, $CDCl_3$) *δ* 163.7, 141.3, 128.5, 124.8, 122.3, 63.3, 46.7, 28.7, 24.1 ppm; ATR-IR 2933 (CH_{2asym}), 2862 (CH_{2sym}), 1698 (C=O), 1605 (C=C_{ar}), 1573, 1430 (CH_{2def}) cm^{-1}; HRMS (ESI) m/z: $[M + H]^+$ calcd for $C_{45}H_{52}N_6O_3Br_3$, 963.1625, 965.1605; found, 963.1637, 965.1624.

Macrocycle 9a: eluent CH_2Cl_2 to CH_2Cl_2:MeOH (97:3), yield 125 mg (56%), colorless amorphous solid. Mp. does not melt up to 305 °C; $[\alpha]_D^{20}$ +69° (*c* 1.08, $CHCl_3$); ^{1}H NMR (300 MHz, $CDCl_3$) *δ* 7.19 (d, *J* = 7.9 Hz, 8H), 7.07 (d, *J* = 7.9 Hz, 8H), 4.39-4.20 (m, 8H), 3.91 (bs, 4H), 2.71 (s, 4H), 1.97 (d, *J* = 6.8 Hz, 4H), 1.75 (bs, 4H), 1.26 (m, 8H) ppm; ^{13}C{^{1}H} NMR (75 MHz, $CDCl_3$) *δ* 163.4, 139.7, 135.8, 129.1, 128.7, 61.9, 46.4, 41.2, 28.4, 24.2 ppm; ATR-IR 2929 (CH_{2asym}), 2859 (CH_{2sym}), 1693 (C=O), 1512 (C=C_{ar}), 1434 (CH_{2def}) cm^{-1}; HRMS (ESI) m/z: $[M + H]^+$ calcd for $C_{44}H_{49}N_4O_2$, 665.3850; found, 665.3867.

General procedure for synthesis of thiourea-derived macrocycles.

Macrocyclic amine (1 eq) and 1,1′-thiocarbonyldiimidazole (1 equivalent per one cyclohexanediamine unit) were dissolved in dichloromethane and then was being stirred at room temperature for 24 h. Silica gel was added to the reaction mixture in one portion and all the volatiles were removed *in vacuo*. The residue was applied at the top of chromatographic column and the product was purified with silica gel column chromatography to give the respective product.

Macrocycle 1b – colorless crystalline solid, (known compound) all spectra were in agreement to that previously reported.[10]

Macrocycle 2b: eluent CH_2Cl_2 to CH_2Cl_2:MeOH (99.5:0.5), yield 64 mg (40%), colorless amorphous solid. Mp. 213-216 °C; $[\alpha]_D^{20}$ +144° (*c* 0.84, $CHCl_3$); ^{1}H NMR (600 MHz, $CDCl_3$, 0 °C) *δ* 7.32 (s, 2H), 7.18 (s, 2H), 6.85 (s, 2H), 5.56 (s, 2H), 5.47 (s, 2H), 5.17 (bs, 2H), 4.63 (bs, 2H), 4.34 (d, *J* = 13 Hz, 2H), 4.01 (d, *J* = 13 Hz, 2H), 3.63 (s, 13H), 3.55 (s, 5H), 2.96 (s, 2H), 2.76 (s, 2H), 2.45 (s, 2H), 2.20 (s, 2H), 1.94 (s, 4H), 1.69 (s, 7H), 1.33-1.18 (m, 6H), 0.95 (s, 5H) ppm; ^{13}C{^{1}H} NMR (150 MHz, $CDCl_3$, 0 °C) *δ* 186.9, 186.3, 151.4, 150.8, 126.1, 125.7, 123.9, 114.8, 114.2, 113.1, 66.6, 65.3, 63.5, 56.0, 55.6, 46.2, 45.1, 43.9, 29.3, 28.8,

28.2, 24.4 ppm; ATR-IR 2934 (CH_{2asym}), 2856 (CH_{2sym}), 2839 (Me-O), 1506, 1437 (CH_{2def}), 1206 (C=S), 1039 (Ar-O-C_{sym}) cm^{-1}; HRMS (ESI) m/z: $[M + H]^+$ calcd for $C_{51}H_{67}N_6O_6S_3$, 955.4279; found, 955.4257.

Macrocycle 3: eluent CH_2Cl_2, yield 31 mg (15%); colorless crystalline solid, $[\alpha]_D^{20}$ +26.1° (*c* 0.236, $CHCl_3$); ^1H NMR (600 MHz, $CDCl_3$, –30 °C) δ 7.54 (s, 3H), 7.45 (s, 3H), 6.10 (d, *J* = 14.6 Hz, 3H), 5.93 (d, *J* = 15.9 Hz, 3H), 4.53 (d, *J* = 15.8 Hz, 3H), 3.86 (d, *J* = 14.7 Hz, 3H), 3.03 (t, *J* = 12.0 Hz, 3H), 2.43 (t, *J* = 12.4 Hz, 3H), 1.76 (s, 2H), 1.68 (d, *J* = 12.2 Hz, 2H), 1.60 (d, *J* = 11.4 Hz, 2H), 1.47-1.28 (m, 12H), 1.15 (d, J = =11.2 Hz, 2H), 0.45 (q, *J* = 12.6 Hz, 2H), 0.26 (q, *J* = 12.4 Hz, 2H) ppm; $^{13}C\{^1H\}$ NMR (150 MHz, $CDCl_3$, –30 °C) δ 186.1, 137.3, 135.9, 134.3, 133.8, 123.6, 121.2, 65.5, 61.8, 52.4, 46.8, 28.9, 26.7, 24.3, 23.9, 23.7 ppm; ATR-IR 2933 (CH_{2asym}), 2856 (CH_{2sym}), 1433 (CH_{2def}), 1233 (C=S), 649 (C_{ar}-Br) cm^{-1}; HRMS (ESI) m/z: $[M + H]^+$ calcd for $C_{45}H_{49}N_6S_3Br_6$, 1246.8235, 1248.8214; found, 1246.8223, 1248.8205.

Macrocycle 5b – yield 169 mg (66%); colorless crystalline solid, (known compound) all spectra were in agreement to that previously reported.[10]

Macrocycle 6b: eluent CH_2Cl_2, yield 164 mg (77%), colorless amorphous solid. Mp. 244-248 °C; $[\alpha]_D^{20}$ +97° (*c* 2.52, $CHCl_3$); ^1H NMR (300 MHz, $CDCl_3$) δ 7.15 (s, 6H), 7.09 (s, 3H), 5.49 (d, *J* = 16 Hz, 6H), 4.47 (d, *J* = 15.9 Hz, 6H), 3.21 (bs 6H), 1.87-1.85 (m, 6H), 1.72-1.71 (m, 6H), 1.37 (s, 27H), 1.28-1.26 (m, 12H) ppm; $^{13}C\{^1H\}$ NMR (75 MHz, $CDCl_3$) δ 188.4, 151.5, 137.4, 122.0, 121.9, 66.5, 50.9, 34.7, 31.5, 29.0, 24.2 ppm; ATR-IR 2938 (CH_{2asym}), 2862 (CH_{2sym}), 1603 (C=C_{ar}), 1439 (CH_{2def}) 1222 (C=S) cm^{-1}; HRMS (ESI) m/z: $[M + H]^+$ calcd for $C_{57}H_{79}N_6S_3$, 943.5523; found, 943.5532.

Macrocycle 7b: eluent CH_2Cl_2, yield 88 mg (20%), colorless amorphous solid. Mp. 181-187 °C; $[\alpha]_D^{20}$ +106° (*c* 0.89, $CHCl_3$); ^1H NMR (600 MHz, $CDCl_3$) δ 6.89 (s, 3H), 6.70 (s, 6H), 5.45 (bs, 6H), 4.43 (bs, 6H), 3.79 (s, 9H), 3.19-3.18 (m, 6H), 1.88 (d, *J* = 7.7 Hz, 6H), 1.73 (d, *J* = 7.7 Hz, 6H), 1.31-1.27 (m, 12H) ppm; $^{13}C\{^1H\}$ NMR (151 MHz, $CDCl_3$) δ 188.4, 160.0, 139.4, 117.2, 110.5, 66.4, 55.3, 50.6, 28.9, 24.1 ppm; ATR-IR 2934 (CH_{2asym}), 2857 (CH_{2sym}), 1596 (C=C_{ar}), 1436 (CH_{2def}),1253 (C-O-C_{def}), 1221 (C=S), 1149 (C-O-C_{def}) cm^{-1}; HRMS (ESI) m/z: $[M + Na]^+$ calcd for $C_{48}H_{60}N_6O_3S_3Na$, 887.3781; found, 887.3793.

Macrocycle 8b: eluent CH_2Cl_2, yield 88 mg (53%), colorless crystalline solid. Mp. 244-249 °C; $[\alpha]_D^{20}$ +93° (*c* 1.41, $CHCl_3$); ^1H NMR (300 MHz, $CDCl_3$) δ 7.31 (s, 6H), 7.24 (s, 3H), 5.45 (d, *J* = 15.5 Hz, 6H), 4.42 (d, *J* = 12.9 Hz, 6H), 3.20 (bs, 6H), 1.89-1.87 (m, 6H), 1.77-1.76 (m, 6H), 1.29-1.27 (m, 12H) ppm; $^{13}C\{^1H\}$ NMR (75 MHz, $CDCl_3$) δ 188.6, 140.3, 127.9, 123.6, 122.6, 66.4, 50.0, 28.8, 24.0 ppm; ATR-IR 2935 (CH_{2asym}), 2858 (CH_{2sym}), 1606 (C=C_{ar}), 1574, 1435 (CH_{2def}) 1221 (C=S) cm^{-1}; HRMS (ESI) m/z: $[M + H]^+$ calcd for $C_{45}H_{52}N_6S_3Br_3$, 1011.0940, 1013.0919; found, 1011.0954, 1013.0939.

Macrocycle 9b: eluent CH_2Cl_2, yield 103 mg (57%), colorless amorphous solid. Mp. does not melt up to 305 °C; $[\alpha]_D^{20}$ +90° (*c* 0.79, $CHCl_3$); ^1H NMR (300 MHz, $CDCl_3$) δ 7.34 (d, *J* = 7.9 Hz, 8H), 7.07 (d, *J* = 7.9 Hz, 8H), 4.84 (bs, 8H), 3.93 (s, 4H), 2.92 (d, *J* = 7.6 Hz, 4H), 2.13 (d, *J* = 9.3 Hz, 4H), 1.81 (d, *J* = 6.9 Hz, 4H), 1.30-1.26 (m, 8H) ppm; $^{13}C\{^1H\}$ NMR (75 MHz, $CDCl_3$) δ 186.5, 139.8, 135.0, 129.1, 128.9, 49.3,

41.1, 29.7, 28.6, 24.2 ppm; ATR-IR 2932 (CH_{2asym}), 2853 (CH_{2sym}), 1511 (C=C_{ar}), 1439 (CH_{2def}) 1220 (C=S) cm^{-1}; HRMS (ESI) m/z: $[M + H]^+$ calcd for $C_{44}H_{49}N_4S_2$, 697.3393; found, 697.3402.

General procedure for synthesis of urea- and thiourea derivatives 10-12.

Diamine (1 eq) and 1,1′-carbonyldiimidazole or 1,1′-thiocarbonyldiimidazole (1 eq) were dissolved in dichloromethane and then was being stirred at room temperature for 24 h. Silica gel was added to the reaction mixture in one portion and all the volatiles were removed *in vacuo*. The residue was applied at the top of chromatographic column and the product was purified with silica gel column chromatography to give the respective product.

Urea derivative 10a: eluent CH_2Cl_2 to CH_2Cl_2:MeOH (95:5), yield 21 mg (64%), colorless amorphous solid. Mp. 195-197 °C (decomposition); $[\alpha]_D^{20}$ +13° (*c* 1.11, $CHCl_3$); ^1H NMR (300 MHz, $CDCl_3$) δ 7.33-7.21 (m, 10H), 4.47 (d, *J* = 15.2 Hz, 2H), 4.36 (d, *J* = 15.1 Hz, 2H), 2.70-2.67 (m, 2H), 1.81 (d, *J* = 7.8 Hz, 2H), 1.68 (d, *J* = 5.9 Hz, 2H), 1.18 (d, *J* = 6.8 Hz, 4H) ppm; $^{13}C\{^1H\}$ NMR (75 MHz, $CDCl_3$) δ 163.6, 137.8, 128.4, 128.3, 127.2, 61.7, 46.9, 28.4, 24.1 ppm; ATR-IR 2935 (CH_{2asym}), 2863 (CH_{2sym}), 1693 (C=O), 1436 (CH_{2def}) cm^{-1}; HRMS (ESI) m/z: $[M + H]^+$ calcd for $C_{21}H_{25}N_2O$, 321.1961; found, 321.1960.

Thiourea derivative 10b: eluent CH_2Cl_2, yield 229 mg (92%), colorless amorphous solid. Mp. 146-147 °C; $[\alpha]_D^{20}$ +180° (*c* 1.44, $CHCl_3$); ^1H NMR (300 MHz, $CDCl_3$) δ 7.39-7.23 (m, 10H), 5.07 (d, *J* = 15.2 Hz, 2H), 4.84 (d, *J* = 15.2 Hz, 2H), 2.91-2.81 (m, 2H), 1.94 (d, *J* = 8.9 Hz, 2H), 1.71 (d, *J* = 7.5 Hz, 2H), 1.31-1.08 (m, 4H) ppm; $^{13}C\{^1H\}$ NMR (75 MHz, $CDCl_3$) δ 187.3, 137.0, 128.5, 128.0, 127.4, 64.6, 49.9, 28.5, 24.1 ppm; ATR-IR 2938 ($CH_{2\ asym}$), 2861 (CH_{2sym}), 1437 (CH_{2def}) 1225 (C=S) cm^{-1}; HRMS (ESI) m/z: $[M + H]^+$ calcd for $C_{21}H_{25}N_2S$, 337.1733; found, 337.1729.

Urea derivative 11a: eluent CH_2Cl_2 to CH_2Cl_2:MeOH (95:5), yield 428 mg (99%), colorless oil; $[\alpha]_D^{20}$ +33° (*c* 1.58, $CHCl_3$); ^1H NMR (300 MHz, $CDCl_3$) δ 7.39 (d, *J* = 7.4 Hz, 2H), 7.22 (t, *J* = 7.7 Hz, 2H), 6.91 (t, *J* = 7.4 Hz, 2H), 6.84 (d, *J* = 8.2 Hz, 2H), 4.44 (q, *J* = 15.6 Hz, 4H), 3.81 (s, 6H), 2.70 (bs, 2H), 1.93-1.91 (m, 2H), 1.71-1.70 (m, 2H), 1.22-1.19 (m, 4H) ppm; $^{13}C\{^1H\}$ NMR (75 MHz, $CDCl_3$) δ 164.2, 157.2, 130.0, 128.1, 126.2, 120.5, 110.0, 62.2, 55.2, 40.9, 28.7, 24.4 ppm; ATR-IR 2937 (CH_{2asym}), 2864 (CH_{3sym}), 2836 (CH_{2sym}), 1697 (C=O), 1437 (CH_{2def}), 1237 (C_{ar}-O), 1172 (C_{alkil}-O), 1027 (C_{alkil}-O), 750 (C_{ar}-H) cm^{-1}; HRMS (ESI) m/z: $[M + H]^+$ calcd for $C_{23}H_{29}N_2O_3$, 381.2173; found, 381.2177.

Thiourea derivative 11b: eluent CH_2Cl_2, yield 417 mg (90%), colorless oil; $[\alpha]_D^{20}$ +144° (*c* 1.73, $CHCl_3$); ^1H NMR (300 MHz, $CDCl_3$) δ 7.43 (d, *J* = 7.4 Hz, 2H), 7.23 (t, *J* = 8.8 Hz, 2H), 6.92 (t, *J* = 7.4 Hz, 2H), 6.86 (d, *J* = 8.2 Hz, 2H), 5.06 (d, *J* = 16.0 Hz, 2H), 4.88 (d, *J* = 16.0 Hz, 2H), 3.83 (s, 6H), 2.96-2.93 (m, 2H), 1.96 (d, *J* = 9.3 Hz, 2H), 1.72 (d, *J* = 6.7 Hz, 2H), 1.29-1.15 (m, 4H) ppm; $^{13}C\{^1H\}$ NMR (75 MHz, $CDCl_3$) δ 187.9, 156.9, 129.0, 128.1, 125.3, 120.5, 110.2, 65.3, 55.3, 43.8, 28.6, 24.3 ppm; ATR-IR 2956 (CH_{3asym}), 2932 (CH_{2asym}), 2873 (CH_{3sym}), 2833 (CH_{2sym}), 1438 (CH_{2def}), 1237 (C_{ar}-O), 1221 (C=S), 1171 (C_{alkil}-O), 1022 (C_{alkil}-O), 751 (C_{ar}-H) cm^{-1}; HRMS (ESI) m/z: $[M + H]^+$ calcd for $C_{23}H_{29}N_2O_2S$, 397.1944; found, 397.1936.

Urea derivative 12a: eluent CH_2Cl_2 to CH_2Cl_2:MeOH (99:1), yield 100 mg (22%), pale yellow oil; $[\alpha]_D^{20}$ +26° (*c* 1.14, $CHCl_3$), ^{1}H NMR (300 MHz, $CDCl_3$) δ 7.53 (dd, *J* = 7.9, 0.8 Hz, 2H), 7.45 (d, *J* = 7.7 Hz, 2H), 7.29 (td, *J* = 7.5, 0.8 Hz, 2H), 7.12 (td, *J* = 7.8, 1.4 Hz, 2H), 4.76 (d, *J* = 16.3 Hz, 2H), 4.35 (d, *J* = 16.3 Hz, 2H), 3.00-2.79 (m, 2H), 1.84 (d, *J* = 7.8 Hz, 2H), 1.72 (d, *J* = 5.9 Hz, 2H), 1.19-1.28 (m, 4H) ppm; ^{13}C{^{1}H} NMR (75 MHz, $CDCl_3$) δ 163.8, 137.3, 132.7, 129.5, 128.6, 127.4, 123.0, 63.1, 47.1, 28.8, 24.2 ppm; ATR-IR 3062 (C_{ar}-H), 2936 (CH_{2asym}), 2864 (CH_{2sym}), 1699 (C=O), 1427 (CH_{2def}), 1254 (C_{ar}-O), 1110 (C_{alkil}-O), 1021 (C_{alkil}-O), 746 (C_{ar}-H) cm^{-1}; HRMS (ESI) m/z: $[M + H]^+$ calcd for $C_{21}H_{23}N_2OBr_2$, 477.0172, 479.0151; found, 477.0179, 479.0164.

Thiourea derivative 12b: eluent CH_2Cl_2, yield 416 mg (89%), colorless foam; $[\alpha]_D^{20}$ +101° (*c* 2.39, $CHCl_3$); ^{1}H NMR (300 MHz, $CDCl_3$) δ 7.55 (dd, *J* = 7.9 0.8Hz, 2H), 7.41 (dd, *J* = 7.6, 0.9Hz, 2H), 7.30 (td, *J* = 7.5, 0.8 Hz, 2H), 7.13 (td, *J* = 7.8, 1.4 Hz, 2H), 5.32 (d, *J* = 16.4 Hz, 2H), 4.82 (d, *J* = 16.4 Hz, 2H), 3.15-2.91 (m, 2H), 1.90 (d, *J* = 7.8 Hz, 2H), 1.75 (d, *J* = 5.8 Hz, 2H), 1.26-1.21 (m, 4H) ppm; ^{13}C{^{1}H} NMR (75 MHz, $CDCl_3$) δ 188.2, 136.2, 132.8, 128.8, 128.7, 127.5, 122.7, 65.8, 50.1, 28.6, 24.2 ppm; ATR-IR 3053 (C_{ar}-H), 2935 (CH_{2asym}), 2854 (CH_{2sym}), 1430 (CH_{2def}), 1228 (C=S), 749 (C_{ar}-H) 653 (C_{ar}-Br) cm^{-1}; HRMS (ESI) m/z: $[M + H]^+$ calcd for $C_{21}H_{23}N_2SBr_2$, 492.9943, 494.9923; found, 492.9938, 494.9920.

Comment on temperature-dependent ^{1}H NMR spectra of 2b

Even at –60 °C, we have not observed the full separation of signals. What is more, as the measurement temperature has decreased, the complexity of the spectra has increased. Probably, the observed picture has consisted of changes in the macrocycle structure (macrocyclic ring flip) and restricted rotation of aryl groups including hindered rotation of the methoxy substituents as well (see **Figure S1**).

Computational details

The ground-state quantum-chemical calculations were performed at the DFT level with the use of B3LYP hybrid functional. The possible structures were pre-optimized at the molecular mechanic level (MM3 force field as implemented in Scigress software),[11] then optimized at the B3LYP/6-31(d) level and re-optimized with the use of the same hybrid functional and enhanced triple-ζ basis set, 6-311G(d,p). To estimate solvent effect the IEFPCM model of dichloromethane, chloroform or acetonitrile was employed.[12-17] For optimized structures, frequency calculations were carried out at the B3LYP/6-311G(d,p) level of theory to confirm that the conformations are stable (no imaginary frequencies were found). If necessary, the same IEFPCM solvent model was used for calculations of frequency calculations. The total and free energy values were used to obtain the Boltzmann population at 298.15 K.

Structure of the model compounds **13**, **14** were based on the respective lowest-energy conformers of their macrocyclic counterparts. Structures of the model compound **15** were generated by systematical change of biphenyl twist angle by 10°. This have given 36 individual structures, which were optimized at the B3LYP/6-311G(d,p) level of theory. Additionally, structures of compounds **1a**, **1b**, **3** and **8b** were optimized at the B3LYP/6-311G(d,p) level starting from X-ray data.

Additionally, for compounds **1a**, **1b**, **3**, **4**, **5a**, **5b**, **9a**, **9b** and for model compounds **13**-**15**, ECD spectra were calculated at the TD-DFT/6-311++G(d,p) level for all stable geometries, according to the previously described procedure.[18] For saving the PCU time in all 'real' compounds used for ECD calculations, the cyclohexane moieties were replaced by the ethane units, and the remaining part of the structure was unchanged. We employed two different hybrid functionals to calculate ECD spectra: CAM-B3LYP[19] and M06-2X.[20,21] Rotatory strengths were calculated using both length and velocity representations. In the present study, the differences between the length and velocity calculated values of rotatory strengths were quite small and, for this reason, only the velocity representations were further used. ECD spectra were simulated by overlapping Gaussian functions for each transition,[22] according to the procedure previously described.[18] It worth noting that all of employed functionals produced similar results, thus we restrict discussion to the results obtained with the use of CAM-B3LYP hybrid functional only. As the energies of electronic transitions were overestimated, the simulated and Boltzmann averaged spectra were wavelength-corrected to match the experimental ones. The calculated energies were scaled (the scaling factors were ranging from 1.05 to 1.10) taking into account the UV maxima. By the same scaling factors were wavelength-corrected ECD spectra.[18]

All the above-mentioned quantum-chemical calculations were performed with the use of Gaussian 09 package.[17]

Crystallographic details

Single crystals suitable for X-ray analysis were obtained by slow evaporation of tetrahydrofuran solution (**1a_I**) or by slow diffusion of diethyl ether vapors to chloroform solutions (**1a_II**, **2b_II**, **3**, **8b**). All crystals subjected to X-ray analysis were mounted on loops by crystal protection grease. Reflection intensities for **1a_I** were measured on a New Xcalibur diffractometer equipped with a Mo $K\alpha$ radiation source (λ = 0.71073 Å), a graphite monochromator and an EosS2 detector, while for **1a_II**, **2b_II**, **3**, **8b** were measured on an Oxford Diffraction SuperNova Atlas diffractometer equipped with a Cu $K\alpha$ radiation source (λ = 1.54184 Å) and an Atlas CCD detector. In all experiments, the diffraction data were collected at 130 K and the temperature was controlled with an Oxford Instruments Cryosystem cold nitrogen-gas blower.

Data collection, reduction and analysis were carried out with CrysAlisPro software.[23] All crystal structures were solved by direct methods using SHELXT-2018 program,[24] and refined by full matrix least squares method on F2 using SHELXL-2018 program.[25]

Unless otherwise specified below, non-hydrogen atoms were refined using anisotropic thermal parameters. Hydrogen atoms bonded to carbon atoms were placed in idealized positions and refined using the riding model, and their isotropic displacement parameters were set equal to 1.2Ueq(C). Non-hydrogen atoms in disordered solvent molecules in crystals: **1a_I** – tetrahydrofuran and water, **1a_II** – chloroform (one of two symmetrically independent molecules) – were refined using isotropic parameters.

In crystal **1b_II**, two and half chloroform molecules have been identified on subsequent difference electron density maps but their disorder has not been precisely modelled. Instead, the electron density corresponding to these included solvent molecules was taken into account using the SQUEEZE/PLATON procedure.[26] The estimated electron count is 161 in an accessible voids volume of 482 Å^3.

In the crystal of **3**, three chloroform molecules have been clearly identified on subsequent difference electron density maps but their disorder has not been precisely modelled. Instead, the electron density corresponding to these included solvent molecules was taken into account using the SQUEEZE/PLATON procedure.[26] The estimated electron count is 174 in an accessible void volume of 507 Å^3.

In case of bromine-derived macrocycles, large residual peaks and/or holes are located near halogen atoms, and this effect is caused by absorption and known in literature.[27]

Absolute structures of the compounds were specified by the synthetic procedure – from the known absolute configuration of *trans*-(*R*,*R*)-1,2-diaminocyclohexane, which was used as a starting material in the syntheses; and for measurements with a Cu Kα radiation source also confirmed using Flack parameter.[28]

Graphical images were produced using ORTEP-3 and Mercury programs.[29] Hirshfeld surface analysis was prepared using CrystalExplorer software.[30]

Crystallographic data and refinement details are collected in **Table S10**.

CCDC 2172822–2172826 contain the supplementary crystallographic data for this paper. These data can be obtained free of charge from The Cambridge Crystallographic Data Centre *via* www.ccdc.cam.ac.uk/data%5Frequest/cif.

Table S1. Total and free energies (*E*, *ΔG*, in Hartree), relative energies (*ΔE*, *ΔΔG* in kcal mol^{-1}), percentage populations and number of imaginary frequencies (#ImFreq) calculated at the B3LYP/6-311G(d,p) level for individual conformers of **1a**.

Conformer no[a]	*E*	*ΔG*	*ΔE*	Pop.	*ΔΔG*	Pop	#ImFreq
1[b]	-2302.13911	-2301.292987	27.93	–	24.36	–	0
2[c]	-2302.18361	-2301.331805	0.00	84.5	0.00	83.63	0
3[d]	-2302.18201	-2301.330266	1.00	15.5	0.97	16.37	0

[a] Conformers are numbered according to their appearance during conformational search; [b] D_3 symmetry; [c] C_3 symmetry; [d] C_1 symmetry.

Table S2. Total and free energies (*E*, *ΔG*, in Hartree), relative energies (*ΔE*, *ΔΔG* in kcal mol^{-1}), percentage populations and number of imaginary frequencies (#ImFreq) calculated at the IEFPCM(ACN)/B3LYP/6-311G(d,p) level for individual conformers of **1a**.

Conformer no[a]	*E*	*ΔG*	*ΔE*	Pop.	*ΔΔG*	Pop	#ImFreq
1[b]	-2302.18207	-2301.333880	17.00	–	16.47	–	0
2[c]	-2302.20916	-2301.360120	0.00	93.11	0.00	90.14	0
3[d]	-2302.2067	-2301.358032	1.54	6.89	1.31	9.86	0

[a] Conformers are numbered according to their appearance during conformational search; [b] D_3 symmetry; [c] C_3 symmetry; [d] C_1 symmetry.

Table S3. Total and free energies (*E*, *ΔG*, in Hartree), relative energies (*ΔE*, *ΔΔG* in kcal mol^{-1}), percentage populations and number of imaginary frequencies (#ImFreq) calculated at the B3LYP/6-311G(d,p) level for individual conformers of **1b**.

Conformer no[a]	*E*	*ΔG*	*ΔE*	Pop.	*ΔΔG*	Pop	#ImFreq
1[b]	-3270.98428	-3270.146516	41.70	–	38.30	–	0
2[c]	-3271.05074	-3270.207558	0.00	88.11	0.00	71.82	0
3[d]	-3271.04885	-3270.206675	1.19	11.89	0.55	28.18	0

[a] Conformers are numbered according to their appearance during conformational search; [b] D_3 symmetry; [c] C_3 symmetry; [d] C_1 symmetry.

Table S4. Total and free energies (*E*, *ΔG*, in Hartree), relative energies (*ΔE*, *ΔΔG* in kcal mol^{-1}), percentage populations and number of imaginary frequencies (#ImFreq) calculated at the B3LYP/6-311G(d,p) level for individual conformers of **3**.

Conformer no[a]	*E*	*ΔG*	*ΔE*	Pop.	*ΔΔG*	Pop	#ImFreq
1	-18712.2722	-18711.507836	1.21	10.65	0.43	26.98	0
2	-18712.2741	-18711.508523	0.00	81.87	0.00	55.88	0
3	-18712.2714	-18711.507151	1.71	4.59	0.86	13.06	0
4	-18712.271	-18711.506055	1.98	2.89	1.55	4.09	0

[a] Conformers are numbered according to their appearance during conformational search.

Table S5. Total and free energies (*E*, *ΔG*, in Hartree), relative energies (*ΔE*, *ΔΔG* in kcal mol^{-1}), percentage populations and number of imaginary frequencies (#ImFreq) calculated at the IEFPCM(CH_2Cl_2)/B3LYP/6-311G(d,p) level for individual conformers of **3**.

Conformer no[a]	*E*	*ΔG*	*ΔE*	Pop.	*ΔΔG*	Pop	#ImFreq
1	-18712.29867	-18711.53389	0.00	67.86	0.00	84.99	0
2	-18712.29756	-18711.53192	0.70	20.96	1.24	10.48	0
3	-18712.29645	-18711.53112	1.39	6.44	1.74	4.52	0
4	-18712.29616	-18711.52973	1.57	4.75	2.61	–	0

[a] Conformers are numbered according to their appearance during conformational search.

Table S6. Total and free energies (*E*, *ΔG*, in Hartree), relative energies (*ΔE*, *ΔΔG* in kcal mol^{-1}), percentage populations and number of imaginary frequencies (#ImFreq) calculated at the B3LYP/6-311G(d,p) level for individual conformers of **4**.

Conformer no[a]	*E*	*ΔG*	*ΔE*	Pop.	*ΔΔG*	Pop	#ImFreq
1[b,c]	-2995.50982	-2994.44323	3.71	–	0.02	46.69	0
2[d,e]	-2995.51208	-2994.43802	2.3	–	3.29	–	0
3[d,f]	-2995.51318	-2994.43853	1.6	3.64	2.97	–	0
4[b,g]	-2995.51574	-2994.44326	0	54.44	0	48.2	0
5[d,c]	-2995.51226	-2994.4388	2.18	–	2.8	–	0
6[d,e]	-2995.51541	-2994.44115	0.21	38.5	1.33	5.11	0
7[d,f]	-2995.51312	-2994.43968	1.64	3.42	2.25	–	0
8[d,g]	-2995.51177	-2994.43876	2.49	–	2.82	–	0

[a] Conformers are numbered according to their appearance during conformational search; [b] C_3 symmetry; [c] *MMM* helicity; [d] C_1 symmetry; [e] *MMP* helicity; [f] *MPP* helicity; [g] *PPP* helicity.

Table S7. Total and free energies (E, ΔG, in Hartree), relative energies (ΔE, $\Delta\Delta G$ in kcal mol^{-1}), percentage populations and number of imaginary frequencies (#ImFreq) calculated at the IEFPCM(ACN)/B3LYP/6-311G(d,p) level for individual conformers of **4**.

Conformer no[a]	*E*	*ΔG*	*ΔE*	Pop.	*ΔΔG*	Pop	#ImFreq
1[b,c]	-2995.54047	-2994.46912	2.97	–	2.86	–	0
2[d,e]	-2995.54212	-2994.47013	1.94	2.24	2.23	–	0
3[d,f]	-2995.54325	-2994.4707	1.23	7.46	1.87	3.47	0
4[b,g]	-2995.54521	-2994.47368	0	59.4	0	81.7	0
5[d,c]	-2995.54195	-2994.46886	2.05	–	3.03	–	0
6[d,e]	-2995.54434	-2994.47115	0.55	23.58	1.59	5.57	0
7[d,f]	-2995.54276	-2994.46918	1.53	4.45	2.83	–	0
8[d,g]	-2995.54235	-2994.47163	1.8	2.86	1.29	9.27	0

[a] Conformers are numbered according to their appearance during conformational search; [b] C_3 symmetry; [c] *MMM* helicity; [d] C_1 symmetry; [e] *MMP* helicity; [f] *MPP* helicity; [g] *PPP* helicity.

Table S8. Total and free energies (E, ΔG, in Hartree), relative energies (ΔE, $\Delta\Delta G$ in kcal mol^{-1}), percentage populations and number of imaginary frequencies (#ImFreq) calculated at the B3LYP/6-311G(d,p) level for individual conformers of **5a**.

Conformer no[a]	*E*	*ΔG*	*ΔE*	Pop.	*ΔΔG*	Pop	#ImFreq
1	-2302.17906	-2301.327298	0.16	43.12	0.71	23.06	0
3	-2302.17933	-2301.328435	0.00	56.88	0.00	76.94	0

[a] Conformers are numbered according to their appearance during conformational search.

Table S9. Total and free energies (E, ΔG, in Hartree), relative energies (ΔE, $\Delta\Delta G$ in kcal mol^{-1}), percentage populations and number of imaginary frequencies (#ImFreq) calculated at the IEFPCM(ACN)/B3LYP/6-311G(d,p) level for individual conformers of **5a**.

Conformer no[a]	*E*	*ΔG*	*ΔE*	Pop.	*ΔΔG*	Pop	#ImFreq
1	-2302.20449	-2301.353213	0.00	66.87	0.00	45.53	0
2	-2302.2029	-2301.351931	1.00	12.35	0.80	11.7	0
3	-2302.20339	-2301.353154	0.69	20.78	0.04	42.77	0

[a] Conformers are numbered according to their appearance during conformational search.

Table S10. Total and free energies (E, ΔG, in Hartree), relative energies (ΔE, $\Delta\Delta G$ in kcal mol^{-1}), percentage populations and number of imaginary frequencies (#ImFreq) calculated at the B3LYP/6-311G(d,p) level for individual conformers of **5b**.

Conformer no[a]	*E*	*ΔG*	*ΔE*	Pop.	*ΔΔG*	Pop	#ImFreq
1	-3271.0422	-3270.199713	0.00	51.47	0.00	56.27	0
2	-3271.04191	-3270.198394	0.18	37.94	0.83	13.91	0
3	-3271.0407	-3270.199114	0.94	10.59	0.38	29.83	0

[a] Conformers are numbered according to their appearance during conformational search.

Table S11. Total and free energies (E, ΔG, in Hartree), relative energies (ΔE, $\Delta\Delta G$ in kcal mol^{-1}), percentage populations and number of imaginary frequencies (#ImFreq) calculated at the B3LYP/6-311G(d,p) level for individual conformers of **9a**.

Conformer no[a]	*E*	*ΔG*	*ΔE*	Pop.	*ΔΔG*	Pop	#ImFreq
1[b]	-2075.62528	-2074.86643	15.43	-	13.14	-	0
2	-2075.63786	-2074.87712	7.53	-	6.43	-	0
3[c]	-2075.64986	-2074.88737	0	92.94	0	57.91	0
4	-2075.64743	-2074.88707	1.53	7.06	0.19	42.09	0

[a] Conformers are numbered according to their appearance during conformational search; [b] D_2 symmetry; [c] C_2 symmetry.

Table S12. Total and free energies (E, ΔG, in Hartree), relative energies (ΔE, $\Delta\Delta G$ in kcal mol^{-1}), percentage populations and number of imaginary frequencies (#ImFreq) calculated at the IEFPCM(ACN)/B3LYP/6-311G(d,p) level for individual conformers of **9a**.

Conformer no[a]	*E*	*ΔG*	*ΔE*	Pop.	*ΔΔG*	Pop	#ImFreq
1[b]	-2075.65671	-2074.89997	8.91	-	6.26	-	0
2	-2075.66191	-2074.90215	5.65	-	4.9	-	0
3[c]	-2075.67092	-2074.90995	0	96.5	0	86.98	0
4	-2075.66779	-2074.90816	1.96	3.5	1.12	13.02	0

[a] Conformers are numbered according to their appearance during conformational search; [b] D_2 symmetry; [c] C_2 symmetry.

Table S13. Total and free energies (E, ΔG, in Hartree), relative energies (ΔE, $\Delta\Delta G$ in kcal mol^{-1}), percentage populations and number of imaginary frequencies (#ImFreq) calculated at the B3LYP/6-311G(d,p) level for individual conformers of **9b**.

Conformer no[a]	*E*	*ΔG*	*ΔE*	Pop.	*ΔΔG*	Pop	#ImFreq
1[b]	-2721.52656	-2720.77363	21.21		18.17		0
2	-2721.54425	-2720.78933	10.11		8.32		0
3[c]	-2721.56037	-2720.80259	0	72.56	0	70.01	0
4	-2721.55945	-2720.80179	0.58	27.44	0.5	29.99	0

[a] Conformers are numbered according to their appearance during conformational search; [b] D_2 symmetry; [c] C_2 symmetry.

Table S14. Total and free energies (E, ΔG, in Hartree), relative energies (ΔE, $\Delta\Delta G$ in kcal mol^{-1}), percentage populations and number of imaginary frequencies (#ImFreq) calculated at the IEFPCM(CH_2Cl_2)/B3LYP/6-311G(d,p) level for individual conformers of **9b**.

Conformer no[a]	*E*	*ΔG*	*ΔE*	Pop.	*ΔΔG*	Pop	#ImFreq
1[b]	-2721.55478	-2720.79303	16.60		20.45		0
2	-2721.56696	-2720.81	8.96		7.90		0
3[c]	-2721.58123	-2720.83	0.00	88.51	0.00	100	0
4	-2721.57931	-2720.82	1.21	11.49	2.46		0

[a] Conformers are numbered according to their appearance during conformational search; [b] D_2 symmetry; [c] C_2 symmetry.

Table S15. Relative free energies (*ΔΔG*°, in kcal mol^{-1}), percentage populations, sequences of torsion angles *β*,*α*,*α*',*β*', pseudotorsion angles *γ* and helicities found in calculated and X-ray determined the structures of **1a**, **1b**, **3**, **5a**, **5b**, **8b**, **9a** and **9b**. The symbols in parentheses indicate symmetry of the given calculated conformer.

Comp.		*ΔΔG*°	Pop.	*β*,*α*,*α*',*β*'[a]	*γ*[a]	Helicity
**1a**[b]	Conf. 1 (D_3)	24.4[b]	-	G^{-},T,T,G^{-}	S,S,S	*n.a.*
	Conf. 2 (C_3)	0.0[b]	84[b]	A^{+},G^{+},G^{-},G^{-}	S,S,S	*n.a.*
	Conf. 3 (C_1)	1.0[b]	16[b]	A^{+},A^{-},A^{+},G^{+}<br>G^{+},A^{+},G^{-},A^{-}<br>G^{+},A^{+}, G^{-},G^{-}	T^{-},S^{-},A^{-}	*n.a.*
**1a**[c]	Mol_I	*n.a.*	*n.a.*	S^{-},A^{+},A^{+},S^{+}<br>G^{-},G^{-},A^{+},S^{+}<br>G^{+},A^{+},G^{-},G^{-}	S^{-},T^{-},T^{+}	*n.a.*
	Mol_II	*n.a.*	*n.a.*	G^{-},G^{-},A^{+},G^{+}<br>S^{+},A^{+},G^{-},G^{-}<br>G^{+},A^{+},G^{-},S^{-}	T^{-},S^{+},T^{+}	*n.a.*
**1b**[b]	Conf. 1 (D_3)	38.3[b]	-	G^{-},T,T,G^{-}	S,S,S	*n.a.*
	Conf. 2 (C_3)	0.0[b]	72[b]	A^{+},G^{+},G^{-},G^{-}	S,S,S	*n.a.*
	Conf. 3 (C_1)	0.5[b]	28[b]	A^{+},A^{-},A^{+},G^{+}<br>G^{+},A^{+},G^{-},A^{-}<br>G^{+},A^{+},G^{-},G^{-}	T^{-},S^{-},A^{-}	*n.a.*
**1b**[c]	Mol_I[d]			G^{+},A^{+},G^{-},G^{-}<br>G^{-},G^{-},G^{+},A^{+}<br>G^{+},A^{+},G^{-},G^{-}	S^{+},T^{-},T^{+}	*n.a.*
	Mol_II			G^{-},G^{-},A^{+},S^{+}<br>G^{+},A^{+},G^{-},G^{-}<br>S^{-},G^{-},A^{+},G^{+}	T^{-},T^{+},S^{+}	*n.a.*
**3**[b]	Conf. 1 (C_3)	0.43[b]	27[b]	$G^{+},A^{+},A^{+}G^{-}$	S^{+},S^{+},S^{+}	*n.a.*
	Conf. 2 (C_1)	0.00[b]	56[b]	T^{-},A^{+},G^{-},A^{+}<br>G^{+},A^{+},G^{-},A^{+}<br>A^{+},A^{-},A^{+},G^{+}	T^{-},S^{-},A^{-}	*n.a.*
	Conf. 3 (C_1)	0.86[b]	13[b]	G^{+},A^{+},G^{-},A^{+}<br>G^{+},A^{+},G^{-},A^{+}<br>G^{+},A^{-},A^{+},T^{-}	S^{-},T^{-},T^{-}	*n.a.*
	Conf. 4 (C_1)	1.55[b]	4[b]	G^{+},A^{+},G^{-},A^{+}<br>A^{+},A^{+},S^{-},G^{-}<br>A^{-},A^{+},G^{-},A^{+}	S^{-},S^{+},S^{-}	*n.a.*
**3**[c]	Mol_I	*n.a.*	*n.a.*	A^{-},A^{+},S^{-},G^{-}<br>A^{-},A^{+},S^{-},G^{-}<br>A^{-},A^{+},S^{-},A^{-}	S^{-},S^{-},S^{-}	*n.a.*
	Mol_II	*n.a.*	*n.a.*	G^{+},G^{+},G^{-},A^{+}<br>G^{+},G^{+},G^{-},A^{+}<br>G^{+},G^{+},G^{-},A^{+}	S^{+},S^{+},S^{+}	*n.a.*

**4**[b]	Conf. 1 (C_3)	0.02[b]	47[b]	G^-,G^-,A^+,A^+	S^-,S^-,S^-	*MMM*
	Conf. 2 (C_1)	3.29[b]	-	G^-,G^-,A^+,G^+<br>G^-,G^-,A^+,A^+<br>G^-,G^-,A^+,G^+	S^+,S^-,S^-	*MMP*
	Conf. 3 (C_1)	2.97[b]	-	G^-,G^-,A^+,G^+<br>A^-,G^-,A^+,G^+<br>A^-,G^-,A^+,G^+	S^+,S^-,S^-	*MPP*
	Conf. 4 (C_3)	0[b]	48[b]	A^-,G^-,A^+,G^+	S^+,S^+,S^+	*PPP*
	Conf. 5 (C_1)	2.80[b]	-	A^-,G^-,A^+,G^+<br>G^-,G^-,A^+,G^+<br>G^+,A^+,G^-,A^-	S^-,T,T^-	*MMM*
	Conf. 6 (C_1)	1.33[b]	5[b]	A^-,G^-,A^+,G^+<br>G^+,A^+,G^-,A^-<br>A^-,G^-,A^+,G^+	T^-,T,S^+	*MMP*
	Conf. 7 (C_1)	2.25[b]	-	A^-,G^-,A^+,G^+<br>A^-,G^-,A^+,S^+<br>S^+,A^+,G^-,A^-	S,T^-,T^-	*MPP*
	Conf. 8 (C_1)	2.82[b]	-	S^+,A^+,G^-,G^-<br>G^-,G^-,A^+,G^+<br>A^-,G^-,A^+,S^+	T,S,T^-	*PPP*
**5a**[b]	Conf. 1 (C_1)	0.71[b]<br>0.00[i]	23[b]<br>45[i]	A^-,A^+,A^+,G^+<br>A^-,A^-,A^+,T<br>A^-,G^-,A^+,G^+	G^-,A^+,G^+	*n.a.*
	Conf. 2[e] (C_1)	2.40[b]<br>0.80[i]	–<br>12[e]	S^-,G^-,A^+,A^-<br>T^+,A^+,G^-,A^-<br>G^+,A^+,A^+,A^-	A^-,G^-,T^-	*n.a.*
	Conf. 3 (C_1)	0.00[b]<br>0.04[i]	77[b]<br>43[i]	S^+,A^+,A^+,A^-<br>G^-,G^-,A^+,A^-<br>A^+,A^-,A^+,G^+	S^+,A^+,T^+	*n.a.*
**5b**	Conf. 1 (C_1)	0.00[b]	56[b]	G^+,A^+,A^+,A^-<br>S^-,G^-,A^+,A^-<br>A^+,G^-,A^+,G^-	T^-,S^+,A^+	*n.a.*
	Conf. 2 (C_1)	0.83[b]	14[b]	S^+,A^+,A^-,A^-<br>G^+,A^+,A^+,A^-<br>G^+,A^+,G^-,A^-	G^-,G^+,A^+	*n.a.*
	Conf. 3 (C_1)	0.38[b]	30[b]	S^+,A^+,G^-,A^-<br>G^+,A^+,A^+,A^-<br>S^-,A^-,A^+,A^-	G^-,S^-,A^-	*n.a.*
**8b**[c]		*n.a.*	*n.a.*	G^+,A^+,A^+,T^-<br>S^-,G^-,A^+,S^+<br>T^+,A^+,A^+,S^+	T^+,S^-,T^-	*n.a.*
**9a**[b]	Conf. 1 (D_2)	13.14[b]	-	G^-,A^+,A^+,G^-	S	*PP*
	Conf. 2 (C_1)	6.43[b]	-	G^-,A^+,A^+,G^+<br>G^+,A^+,G^-,A^-	A^+	*MM*

	Conf. 3 (C_2)	0.00[b]	58[b]	A^-,G^-,A^+,G^+	S^+	*PP*
	Conf. 4 (C_2)	0.19[b]	42[b]	A^+,A^+,G^-,A^-	*T*	*PP*
**9b**[b]	Conf. 1 (D_2)	18.17[b]	-	G^-,A^+,A^+,G^-	*S*	*PP*
	Conf. 2 (C_1)	8.32[b]	-	G^-,A^+,A^+,G^+<br>G^+,A^+,G^-,A^-	A^+	*MM*
	Conf. 3 (C_2)	0.00[b]	70[b]	A^-,G^-,A^+,G^+	S^+	*PP*
	Conf. 4 (C_2)	0.50[b]	30[b]	A^+,A^+,G^-,A^-	*T*	*PP*

[a] sequences of the torsion angles refer to the structure calculated in the gas phase: *S* – *synclinal*, *G* – *gauche*, *A* – *anticlinal*; *T* – *trans*; [b] calculated at the B3LYP/6-311G(d,p) level; [c] X-ray determined structure; [d] data taken from ref. 10; [e] calculated at the IEFPCM(MeCN)/B3LYP/6-311G(d,p) level.

Table S16. Experimental details.

	**1a_I**	**1a_II**	**1b_II**	**3**	**8b**
**Crystal data**					
Chemical formula	**$(C_{45}H_{54}N_6O_3)$<br>$\cdot 1.8(C_4H_8O)\cdot 0.2(H_2O)$**	**$(C_{45}H_{54}N_6O_3)$<br>$\cdot 2(CHCl_3)$**	**$(C_{45}H_{54}N_6O_6)$<br>$\cdot 1.625(CHCl_3)$**	**$(C_{45}H_{48}Br_6N_6S_3)$<br>$\cdot 2.5(CHCl_3)$**	**$(C_{45}H_{51}Br_3N_6S_3)$<br>$\cdot(CHCl_3)$**
M_r	860.33	965.67	969.09	1546.95	1131.19
Crystal system, space group	Orthorhombic, $P2_12_12_1$	Orthorhombic, $P2_12_12_1$	Orthorhombic, $P2_12_12_1$	Triclinic, $P1$	Orthorhombic, $P2_12_12_1$
a(Å)	12.6729 (9)	12.7006 (2)	9.0579 (1)	8.9964 (2)	13.0616 (2)
b (Å)	14.9048 (10)	14.7451 (2)	19.5328 (2)	13.3846 (4)	15.7448 (2)
c (Å)	25.0178 (18)	25.2415 (3)	27.1022 (3)	25.4282 (4)	23.9319 (4)
γ (°)	90	90	90	85.711 (2)	90
β (°)	90	90	90	82.225 (1)	90
γ (°)	90	90	90	71.680 (2)	90
V (Å^3)	4725.5 (6)	4727.02 (11)	4795.09 (9)	2878.29 (12)	4921.65 (13)
Z	4	4	4	2	4
Radiation type	Mo $K\alpha$	Cu $K\alpha$	Cu $K\alpha$	Cu $K\alpha$	Cu $K\alpha$
μ (mm^{-1})	0.08	3.69	4.22	9.58	6.03
Crystal size (mm)	0.50 × 0.10 × 0.06	0.25 × 0.10 × 0.05	0.50 × 0.08 × 0.05	0.50 × 0.20 × 0.10	0.30 × 0.20 × 0.05
**Data collection**					
Temperature (K)	130	130	130	130	130
Absorption correction	–	Multi-scan	Multi-scan	Multi-scan	Multi-scan
T_{min}, T_{max}	–	0.677, 1.000	0.714, 0.714	0.385, 1.000	0.748, 1.000
No. of measured, independent and observed [$I > 2\sigma(I)$] reflections	33447, 8318, 4917	25310, 8345, 7588	27786, 9883, 9328	57316, 19352, 19054	26774, 8657, 8424
R_{int}	0.183	0.043	0.047	0.038	0.040
(sin $\Theta/\lambda)_{max}$ (Å^{-1})	0.595	0.595	0.631	0.595	0.595
**Refinement**					
$R[F^2 > 2\sigma(F^2)]$, $wR(F^2)$, S	0.093, 0.202, 1.07	0.063, 0.174, 1.05	0.045, 0.123, 1.03	0.051, 0.152, 1.07	0.062, 0.172, 1.05
$\Delta\rho_{max}$, $\Delta\rho_{min}$ (e Å^{-3})	0.34, -0.35	0.60, -0.70	0.76, -0.60	0.78, -1.04	1.81, -0.97
Absolute structure parameter	-1.9 (10)	-0.006 (7)	0.031 (8)	0.10 (2)	-0.036 (12)

Table S17. Calculated angles (φ) of inclination of the planes of aromatic linkers to the plane of the macrocycle rim. Plane of aromatic linker has been designated by all aromatic carbon atoms, and macrocycles planes – by all six nitrogen atoms from macrocyclic rim.

			**φ (°)**	
Bridged trianglimine [31]		90	89	83
**1a_I**		22	13	8
**1a_II**		22	16	11
**1b_I** [10]	molecule 1	20	14	8
	molecule 2	19	14	6
**1b_II**		19	15	10
**3**	molecule 1	33	32	29
	molecule 2	33	32	31
**8b**		27	27	14

Hydrogen and halogen bonds parameters

Table S18. Selected hydrogen bonds parameters for **1a_I**, **1a_II**, **1b_II**, **3**, **8b**.

D*—H···*A	***D*—H (Å)**	**H···*A* (Å)**	***D*···*A* (Å)**	***D*—H···*A* (°)**
**1a_I**				
C03—H03A···O3^{i}	0.99	2.49	3.207 (9)	129.3
C1R2^{b}—H1R3^{b}···O3^{ii}	0.99	2.64	3.48 (2)	142.9
**1a_II**				
C03—H03A···O3^{iii}	0.99	2.48	3.156 (7)	125.3
C03—H03B···Cl2A^{a}	0.99	2.86	3.846 (7)	174.6
C08—H08B···Cl2A^{aiv}	0.99	2.96	3.670 (7)	129.6
C08—H08B···Cl2B^{biv}	0.99	2.80	3.437 (10)	122.5
C45—H45B···O3^{iii}	0.99	2.63	3.587 (8)	161.9
C1RA^{a}—H1RA^{a}···O1^{v}	1.00	2.43	3.250 (9)	138.4
C1RB^{b}—H1RB^{b}···O3^{iii}	1.00	2.51	3.422 (16)	152.1
**1b_II**				
C08—H08B···S1	0.99	2.66	3.176 (4)	112.7
C15—H15B···S2	0.99	2.67	3.166 (4)	111.3
C17—H17···Cl1	1.00	2.91	3.902 (4)	170.9
C30—H30B···S1^{vi}	0.99	2.97	3.941 (4)	165.7
C36—H36B···Cl2^{vii}	0.99	2.93	3.670 (5)	132.5
C45—H45B···S2^{viii}	0.99	2.81	3.766 (4)	163.0
**3**				
C1R—H1R···S3^{ix}	1.00	2.84	3.730 (11)	148.2
C02—H02···Br1	1.00	3.13	3.744 (9)	121.1
C2R—H2R···S6^{x}	1.00	2.85	3.734 (12)	148.2
C08—H08A···S1	0.99	2.65	3.165 (10)	112.5
C08—H08A···Br4^{ix}	0.99	2.91	3.744 (10)	142.3
C08—H08B···S3^{ii}	0.99	2.83	3.563 (9)	131.1
C11—H11···Br3	0.95	3.08	3.977 (11)	158.9
C15—H15A···Br2	0.99	2.71	3.215 (11)	112.1
C15—H15B···S2	0.99	2.72	3.204 (12)	110.6
C15—H15B···Br8	0.99	2.98	3.681 (11)	129.1
C17—H17···Br3	1.00	2.93	3.589 (11)	124.4
C20—H20B···Br3^{vii}	0.99	2.91	3.650 (10)	132.3
C23—H23A···S2	0.99	2.62	3.141 (13)	112.6
C26—H26···Br5	0.95	3.12	4.047 (11)	167.1

C30—H30B···S3	0.99	2.67	3.167 (13)	111.5
C37—H37···Cl2^{x}	1.00	2.98	3.751 (11)	134.4
C38—H38A···S3	0.99	2.63	3.131 (10)	111.3
C38—H38B···Cl1^{x}	0.99	2.73	3.542 (10)	139.6
C45—H45A···Br6	0.99	2.74	3.238 (10)	111.6
C45—H45B···S1	0.99	2.67	3.172 (11)	111.5
C45—H45B···Cl6^{xi}	0.99	2.77	3.466 (11)	128.2
C49—H49B···Br11^{xiv}	0.99	2.98	3.670 (10)	128.2
C50—H50A···Br11^{xiv}	0.99	2.94	3.580 (10)	123.0
C53—H53A···Br2	0.99	2.89	3.664 (11)	135.2
C53—H53B···Br8	0.99	2.72	3.222 (12)	112.0
C60—H60A···S6^{xii}	0.99	2.98	3.776 (10)	138.0
C60—H60B···S5	0.99	2.65	3.144 (10)	111.0
C60—H60B···Br12^{x}	0.99	3.01	3.826 (10)	140.0
C63—H63A···Cl1^{xiii}	0.99	2.97	3.612 (10)	123.5
C63—H63B···Cl6^{xiv}	0.99	2.92	3.724 (11)	138.5
C68—H68A···Br6^{xiii}	0.99	3.03	3.656 (10)	122.1
C68—H68A···Cl3^{xv}	0.99	2.84	3.552 (10)	129.9
C68—H68B···Br10	0.99	2.68	3.194 (10)	112.8
C75—H75A···Cl5^{ix}	0.99	2.76	3.656 (11)	150.8
C79—H79B···S6^{xiv}	0.99	2.88	3.770 (10)	151.1
C83—H83B···Br12	0.99	2.70	3.192 (10)	110.9
**8b**				
C1R—H1R···S2^{iii}	1.00	2.79	3.579 (12)	136.0
C03A^{a}—H03A^{a}···S3^{i}	0.99	3.00	3.96 (3)	161.7
C12—H12···S1^{xvi}	0.95	2.55	3.401 (9)	149.8
C23—H23B···S2	0.99	2.61	3.148 (9)	114.0
C36—H36A···Cl1^{xii}	0.99	2.85	3.641 (10)	137.4
C38—H38A···S2^{xvii}	0.99	2.83	3.766 (10)	158.2
C38—H38B···S3	0.99	2.64	3.168 (12)	113.5
C45—H45A···S3^{i}	0.99	2.93	3.880 (10)	161.8

Symmetry code(s): (i) x-1/2, -y+3/2, -z+1; (ii) x, y-1, z; (iii) x+1/2, -y+1/2, -z+1; (iv) x-1/2, -y-1/2, -z+1; (v) x+1/2, -y-1/2, -z+1; (vi) -x+3/2, -y+1, z-1/2; (vii) x-1, y, z; (viii) -x+1, y+1/2, -z+1/2; (ix) x+1, y-1, z; (x) x-1, y+1, z; (xi) x+2, y, z-1; (xii) x, y+1, z; (xiii) x-1, y, z+1; (xiv) x+1, y, z; (xv) x-2, y, z+1; (xvi) x-1/2, -y+1/2, -z+1; (xvii) x+1/2, -y+3/2, -z+1.

Table S19. Selected halogen bonds parameters for **1b_II**, **3** and **8b**.

D*—X···*A	***D*—X (Å)**	**X···*A* (Å)**	***D*···*A* (Å)**	***D*—X···*A* (°)**
**1b_II**				
C1R—Cl3···S1	1.779 (5)	3.397 (2)	5.165 (5)	172.3 (2)
**3**				
C1R—Cl2···S1	1.743 (12)	3.367 (3)	5.11 (1)	176.0 (4)
C2R—Cl4···S5	1.760 (11)	3.358 (3)	5.12 (1)	178.0 (4)
C14—Br2···S4	1.896 (10)	3.554 (3)	5.41 (1)	165.1 (3)
C43—Br6···S5^{i}	1.897 (9)	3.475 (3)	5.36 (1)	171.5 (3)
C55—Br8···S2	1.913 (11)	3.394 (3)	5.28 (1)	167.1 (4)
C74—Br10^{i}···S1	1.908 (10)	3.416 (3)	5.31 (1)	172.4 (3)
**8b**				
C1R—Cl1···Br1	1.752 (13)	3.414 (4)	5.08 (1)	157.8 (5)
C11—Br1···S3^{ii}	1.918 (8)	3.381 (3)	5.236 (8)	161.5 (3)

Symmetry code(s): (i) 1+x, y, -1+z; (ii) -1/2+x, ½-y, 1-z.

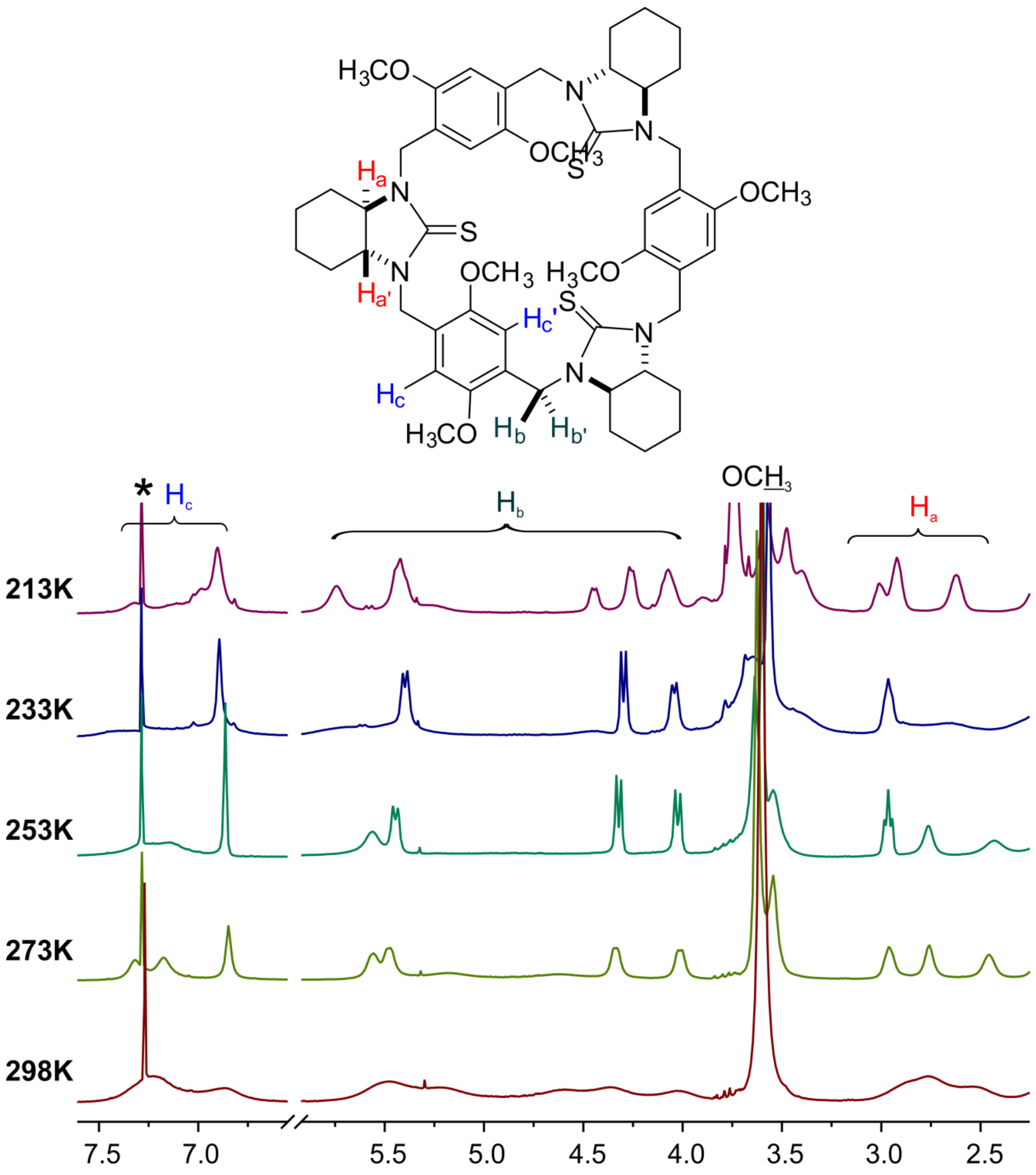

Figure S1. Parts of the temperature-dependent ^{1}H NMR spectra (600 MHz, $CDCl_3$) of **2b**. Asterisk indicates trace solvent peaks.

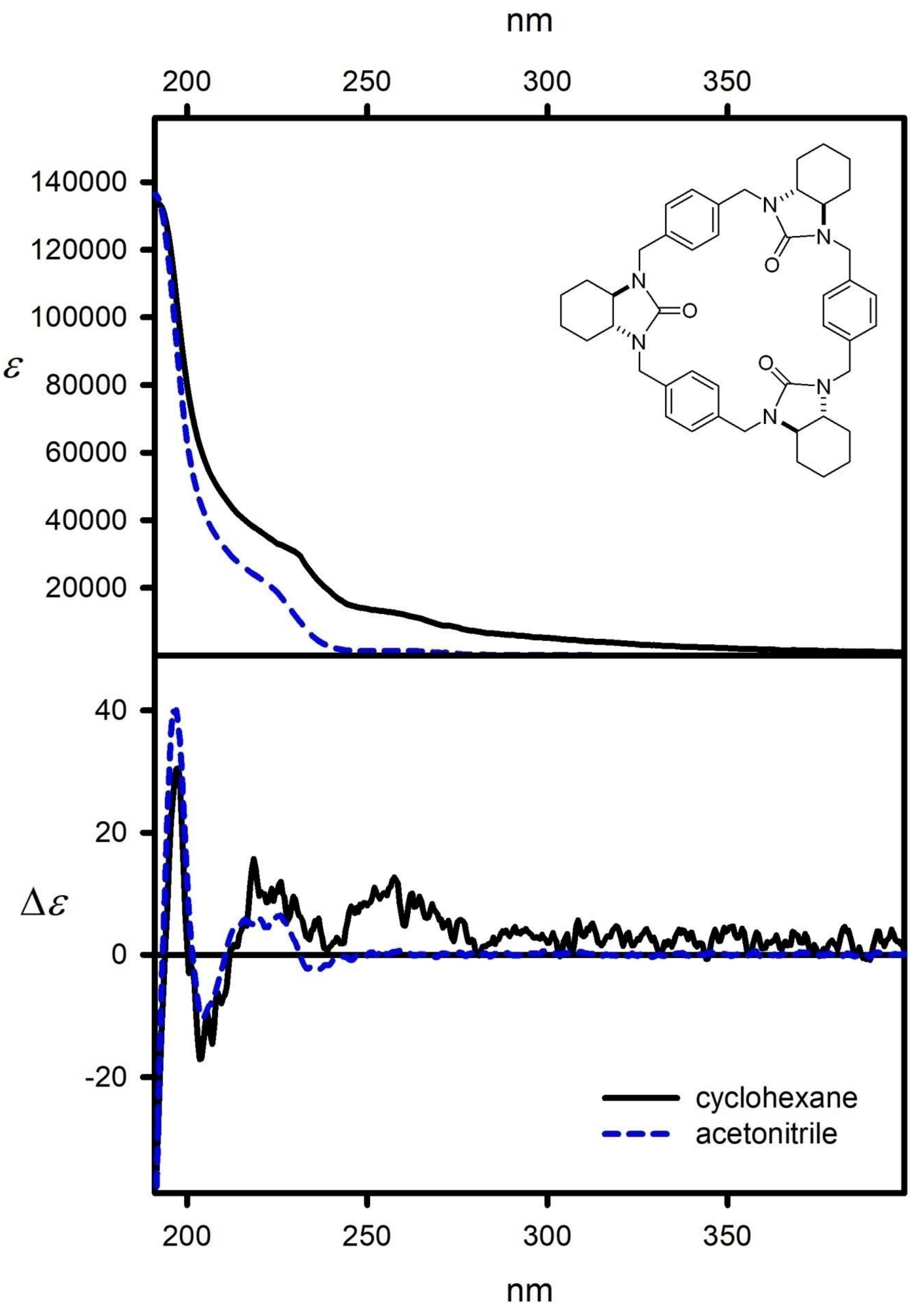

Figure S2. UV (upper panel) and ECD (lower panel) spectra of **1a** measured in cyclohexane (solid black lines) and acetonitrile (blue dashed lines).

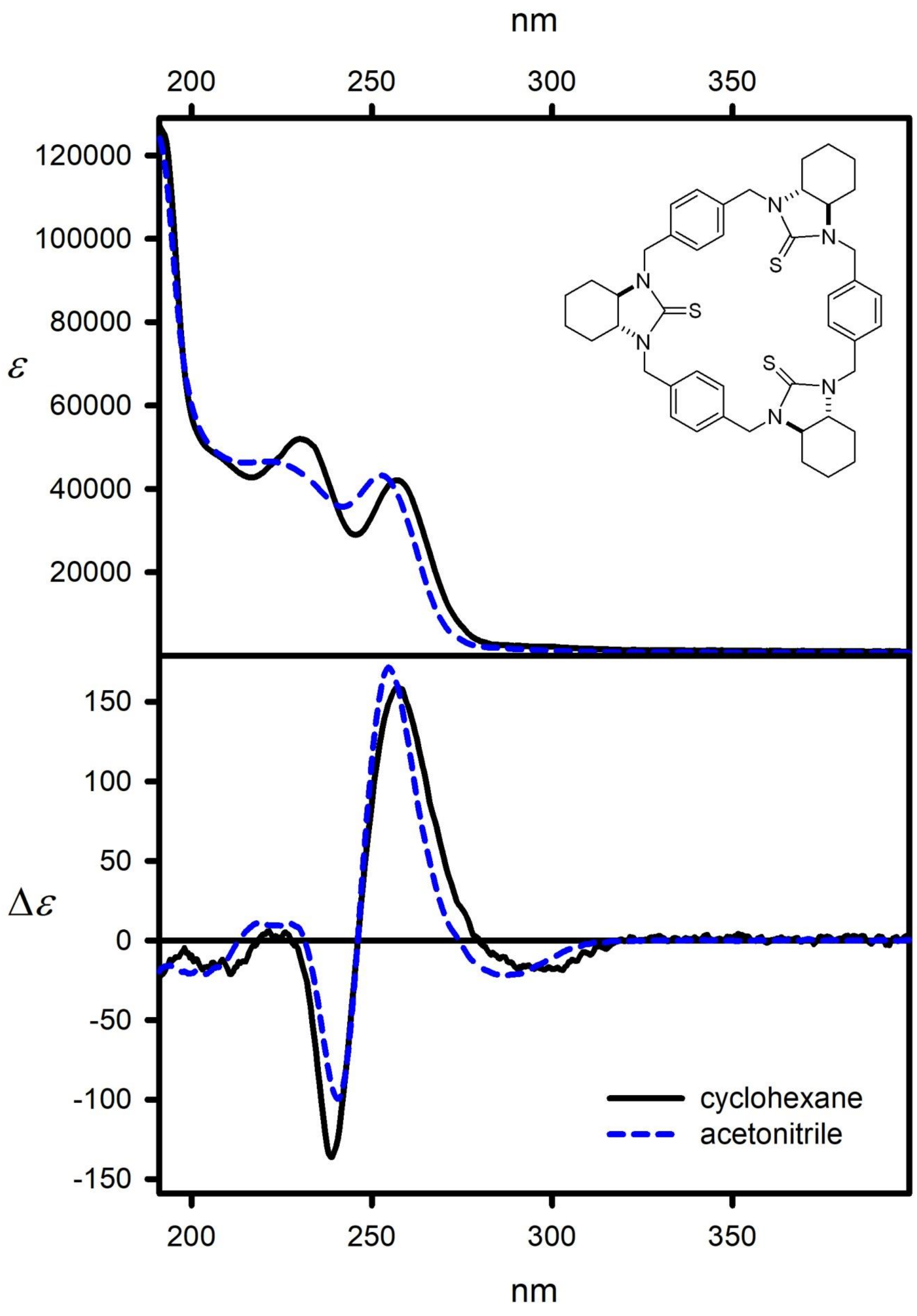

Figure S3. UV (upper panel) and ECD (lower panel) spectra of **1b** measured in cyclohexane (solid black lines) and acetonitrile (blue dashed lines).

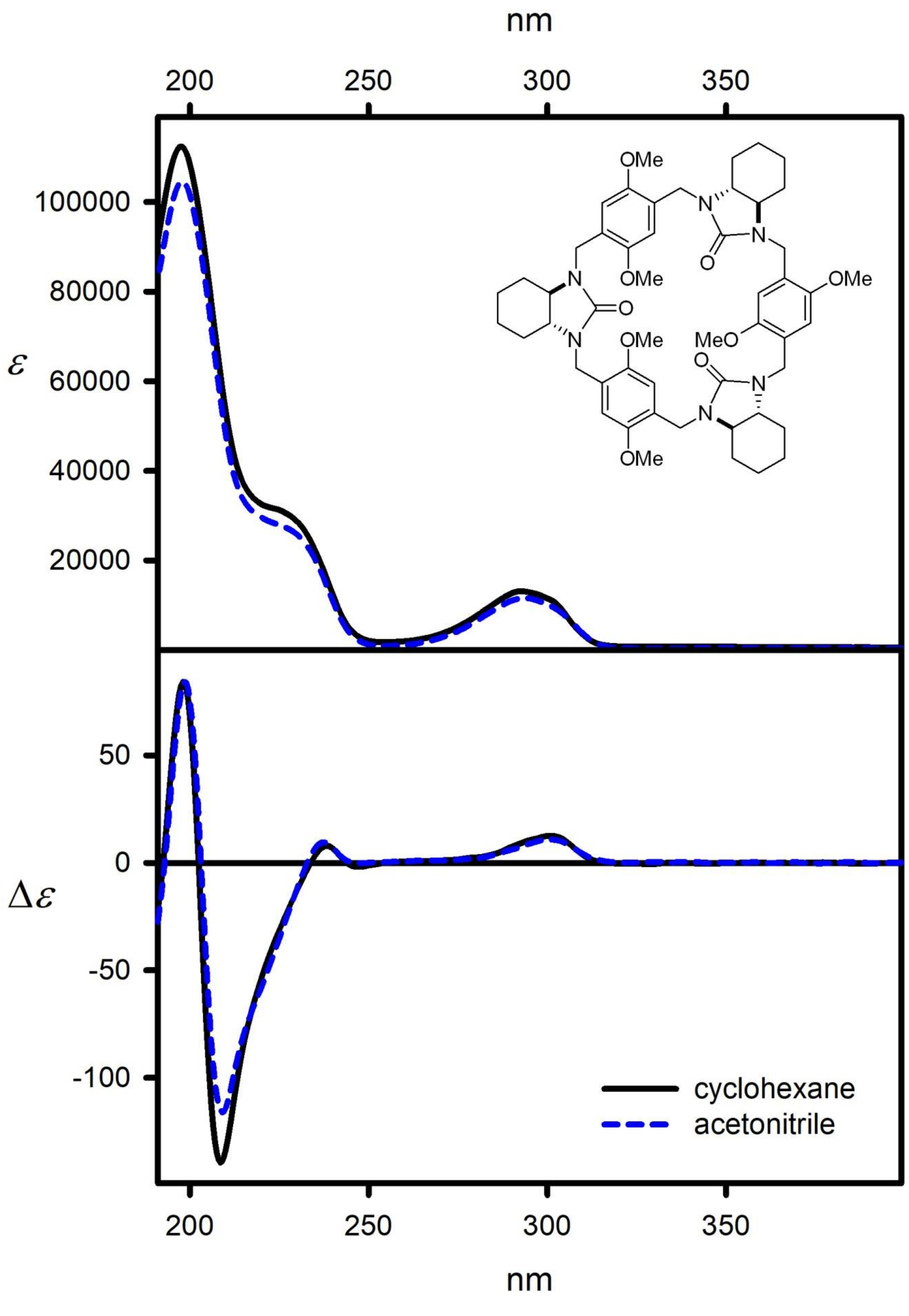

Figure S4. UV (upper panel) and ECD (lower panel) spectra of **2a** measured in cyclohexane (solid black lines) and acetonitrile (blue dashed lines).

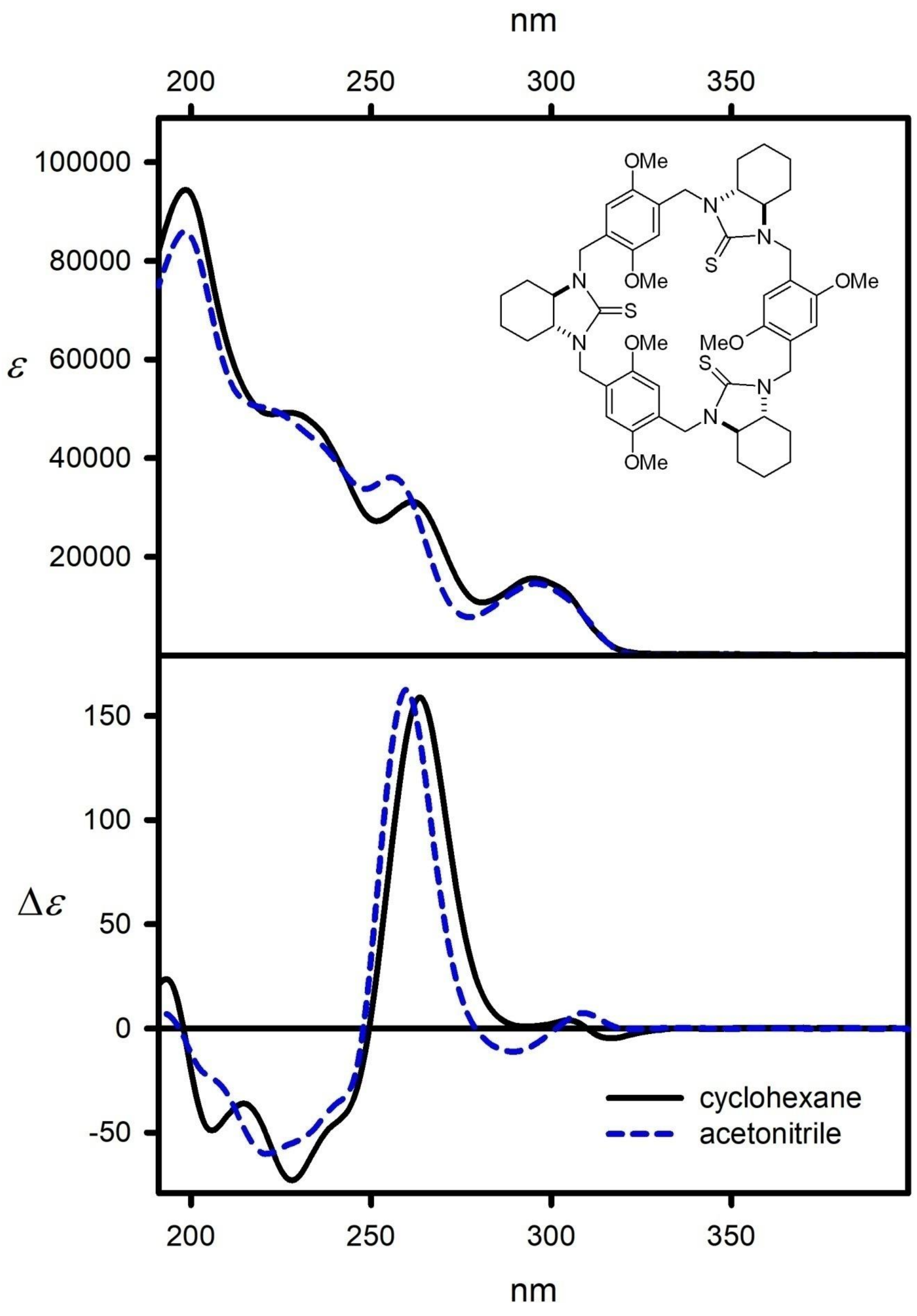

Figure S5. UV (upper panel) and ECD (lower panel) spectra of **2b** measured in cyclohexane (solid black lines) and acetonitrile (blue dashed lines).

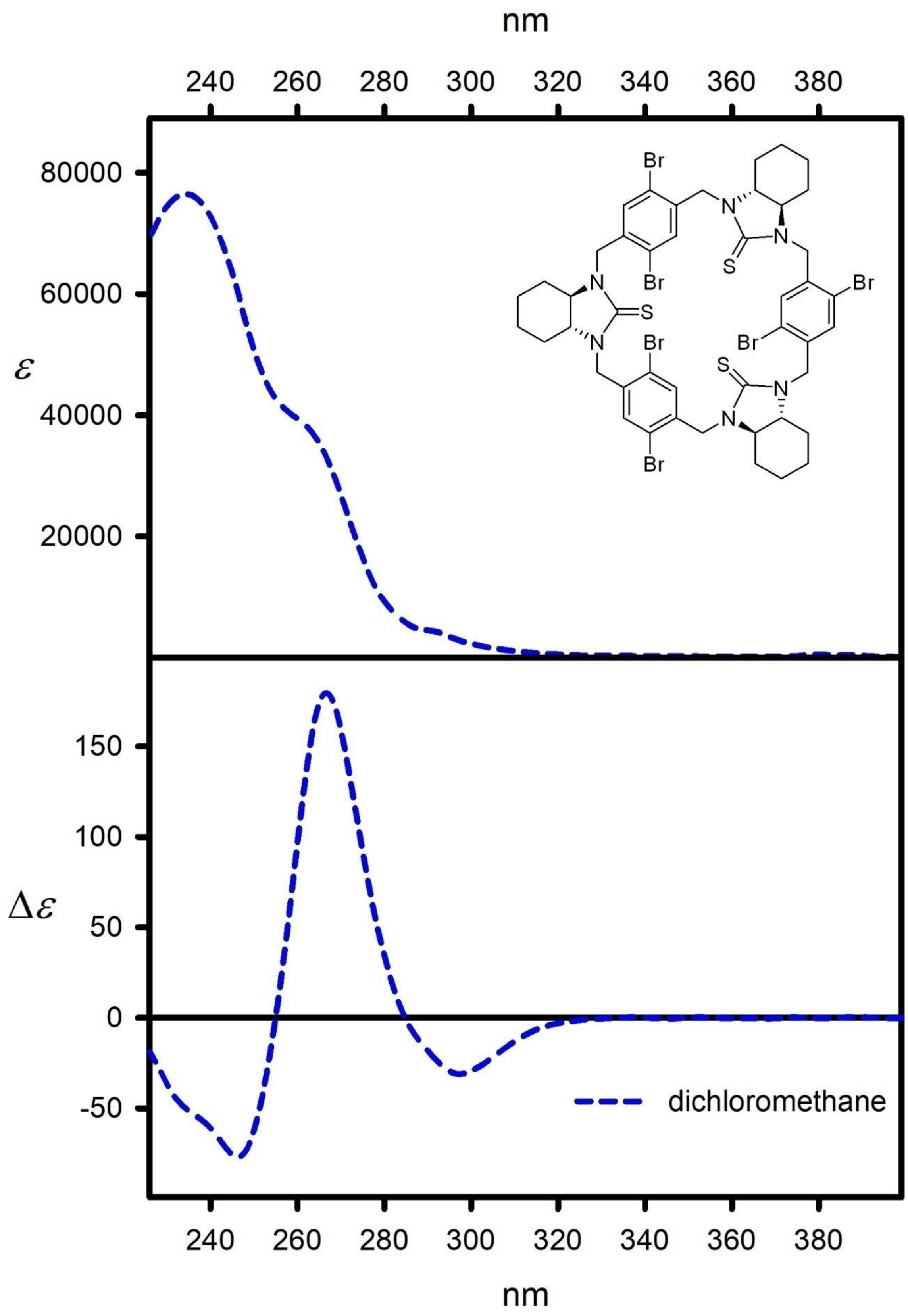

Figure S6. UV (upper panel) and ECD (lower panel) spectra of **3** measured in dichloromethane ~~acetonitrile~~ (blue dashed lines).

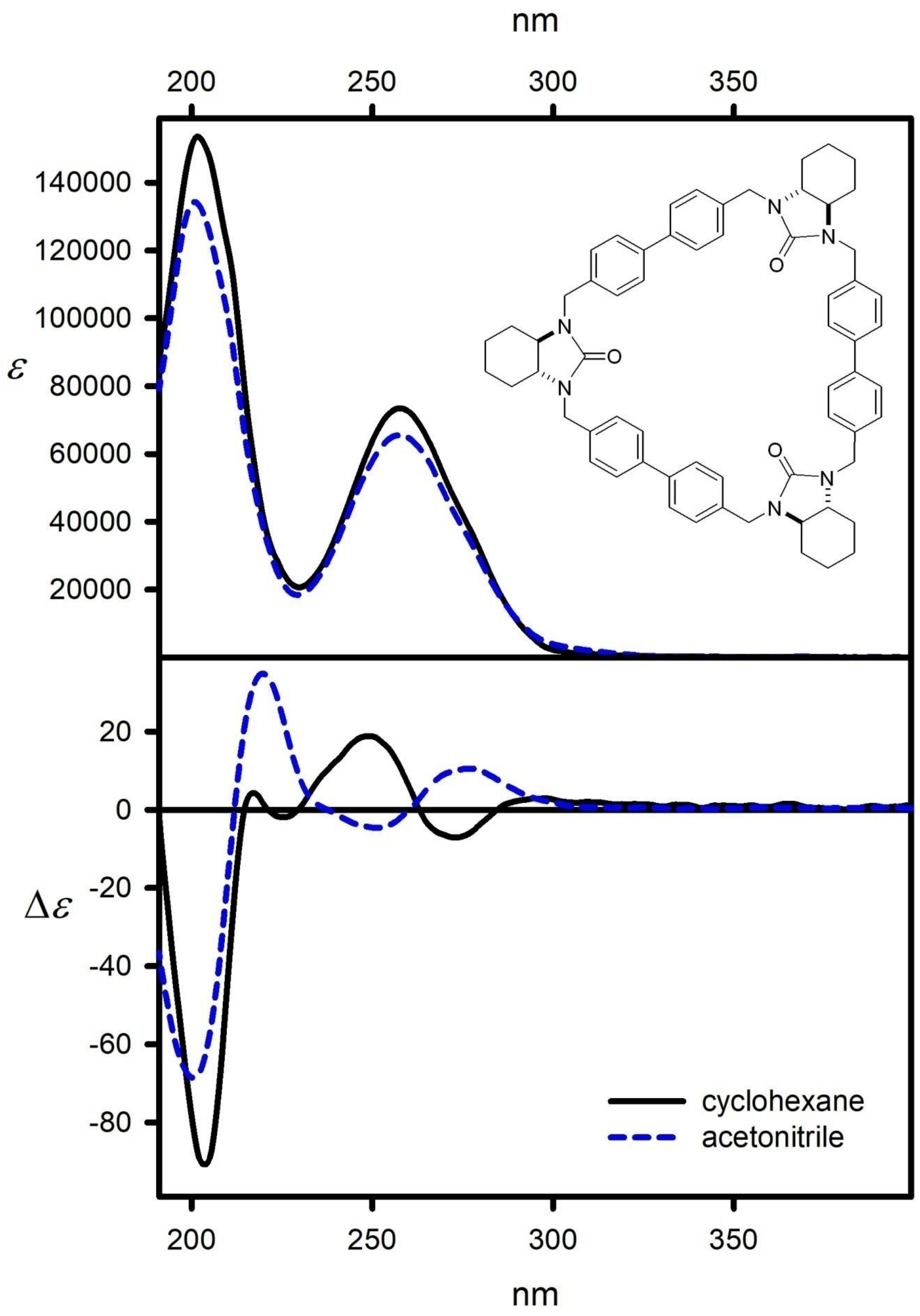

Figure S7. UV (upper panel) and ECD (lower panel) spectra of **4** measured in cyclohexane (solid black lines) and acetonitrile (blue dashed lines).

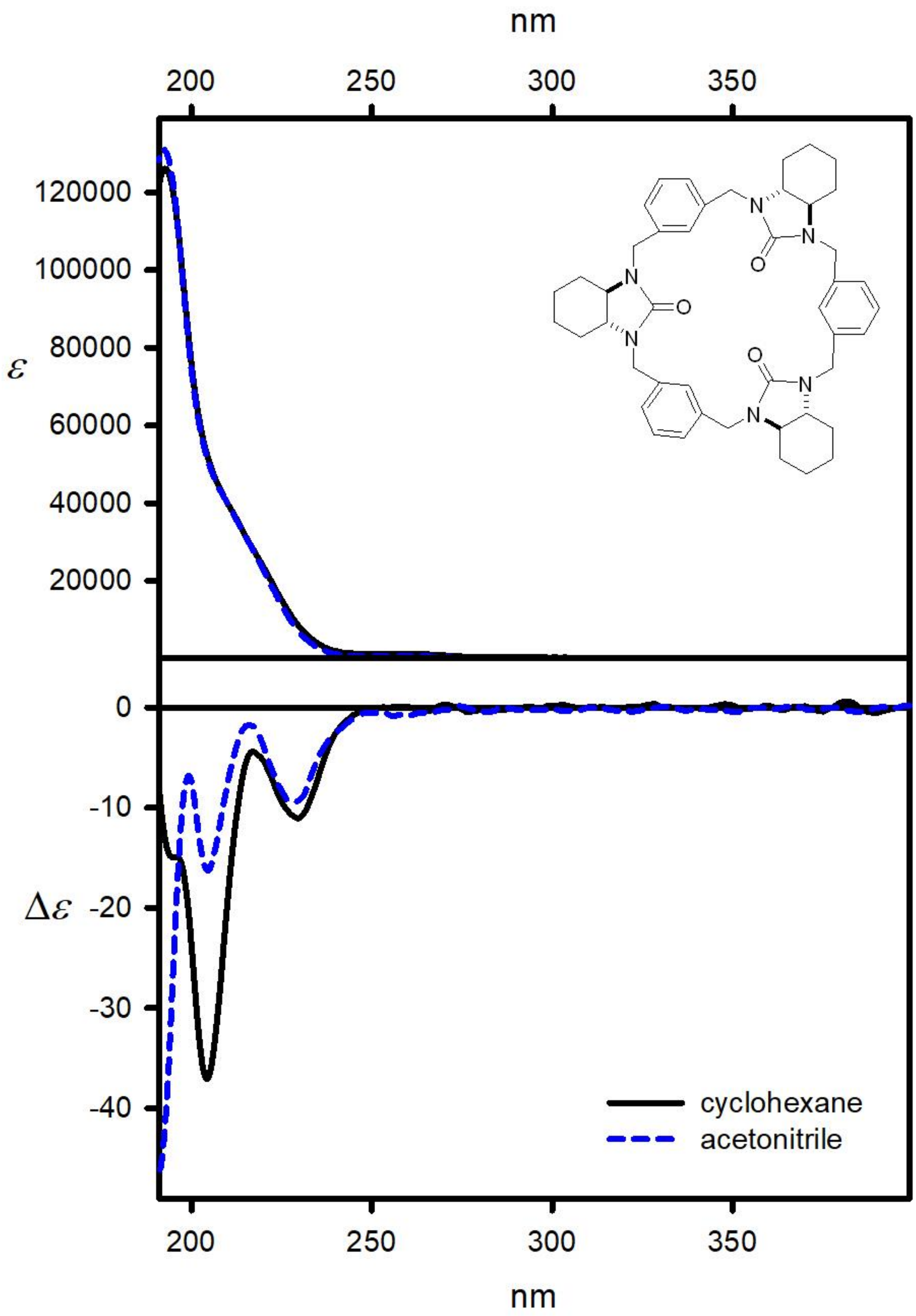

Figure S8. UV (upper panel) and ECD (lower panel) spectra of **5a** measured in cyclohexane (solid black lines) and acetonitrile (blue dashed lines).

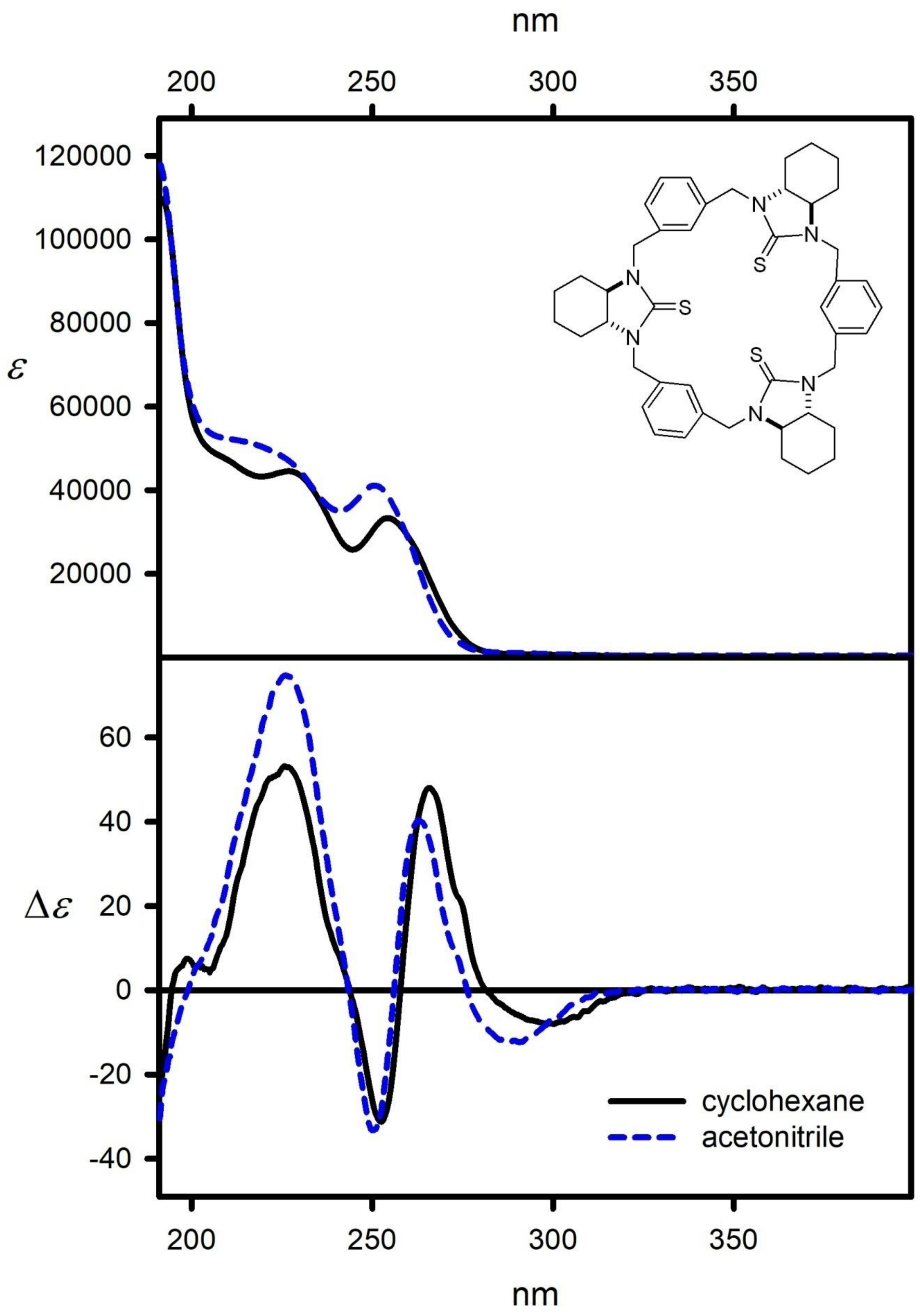

Figure S9. UV (upper panel) and ECD (lower panel) spectra of **5b** measured in cyclohexane (solid black lines) and acetonitrile (blue dashed lines).

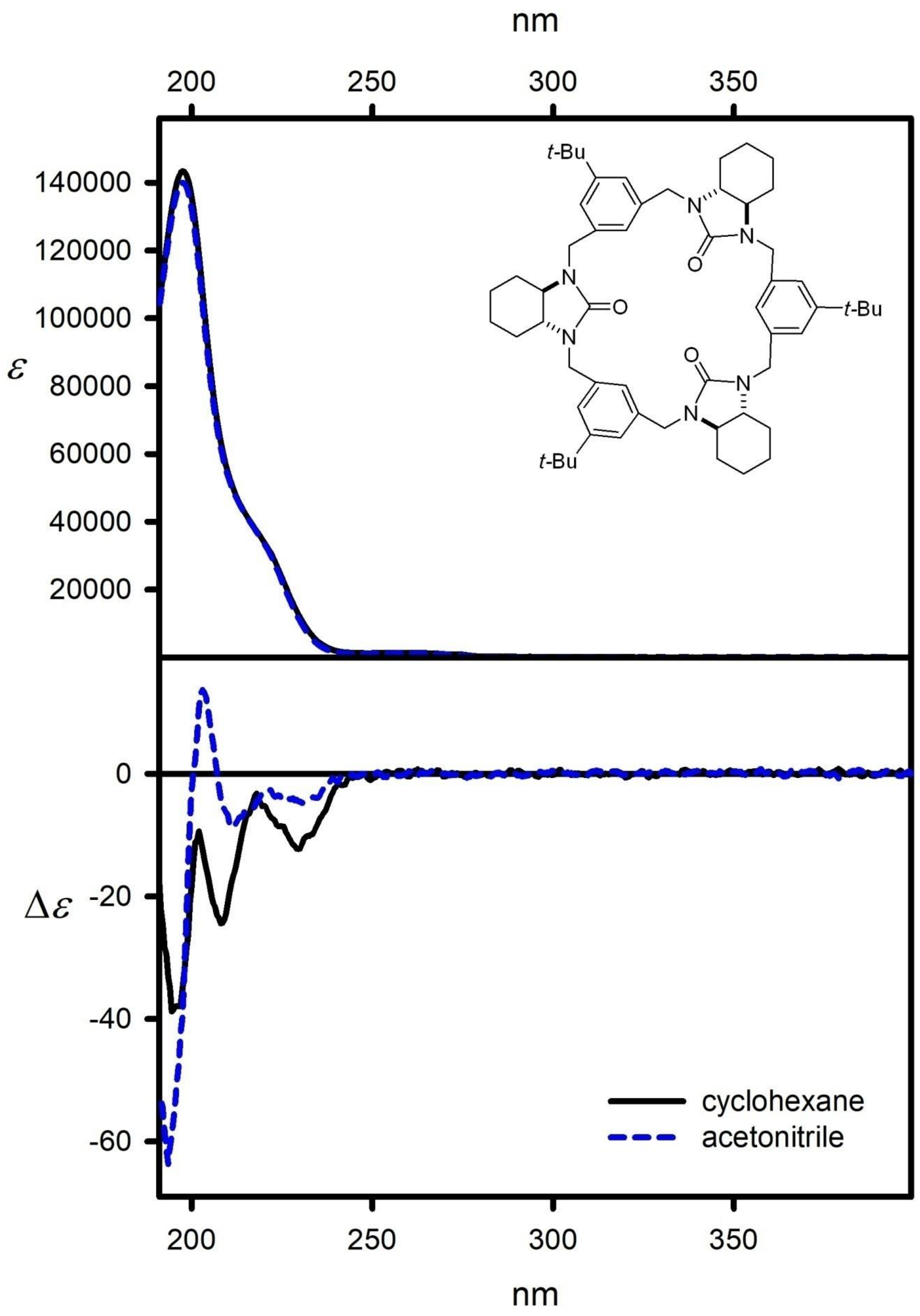

Figure S10. UV (upper panel) and ECD (lower panel) spectra of **6a** measured in cyclohexane (solid black lines) and acetonitrile (blue dashed lines).

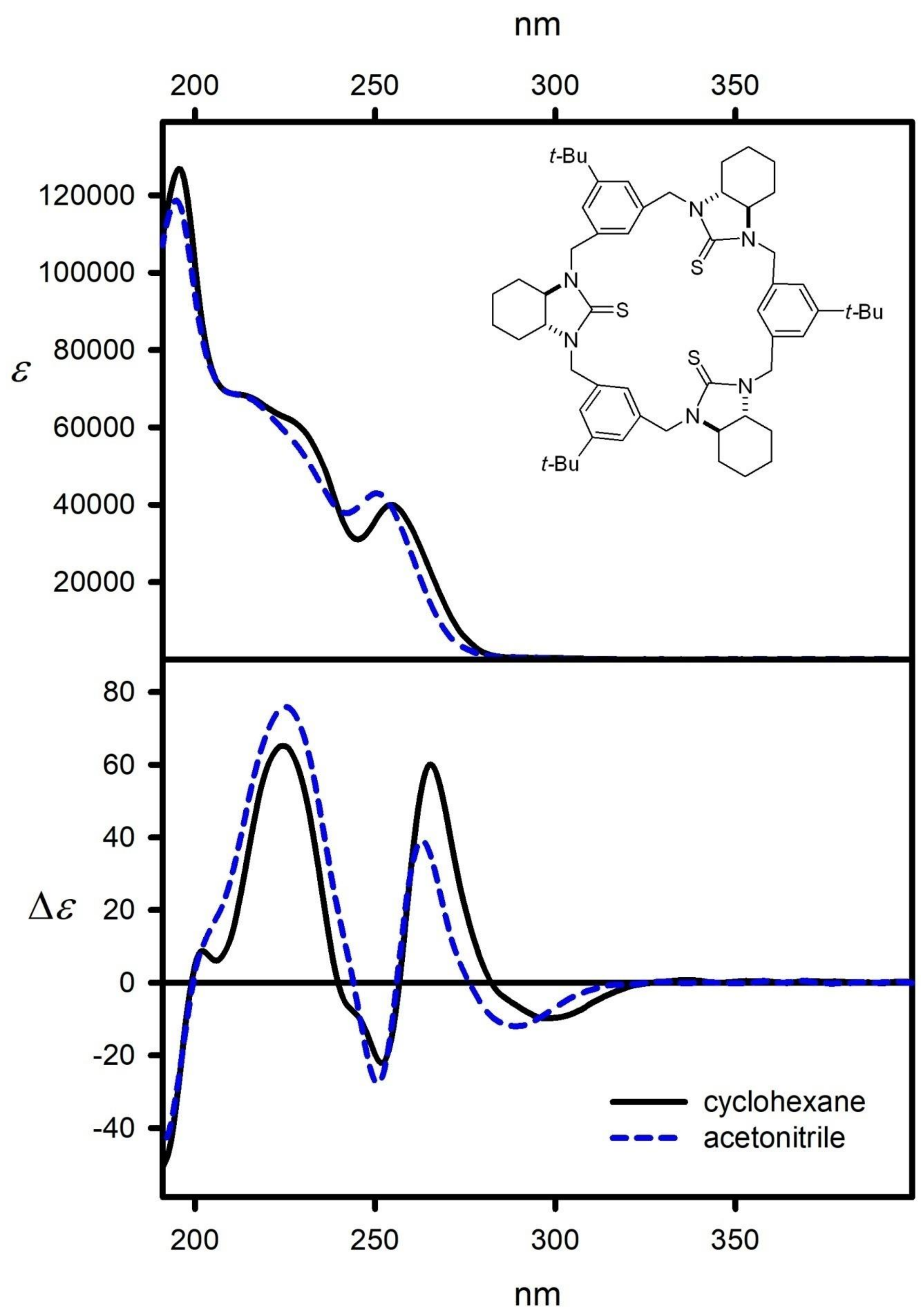

Figure S11. UV (upper panel) and ECD (lower panel) spectra of **6b** measured in cyclohexane (solid black lines) and acetonitrile (blue dashed lines).

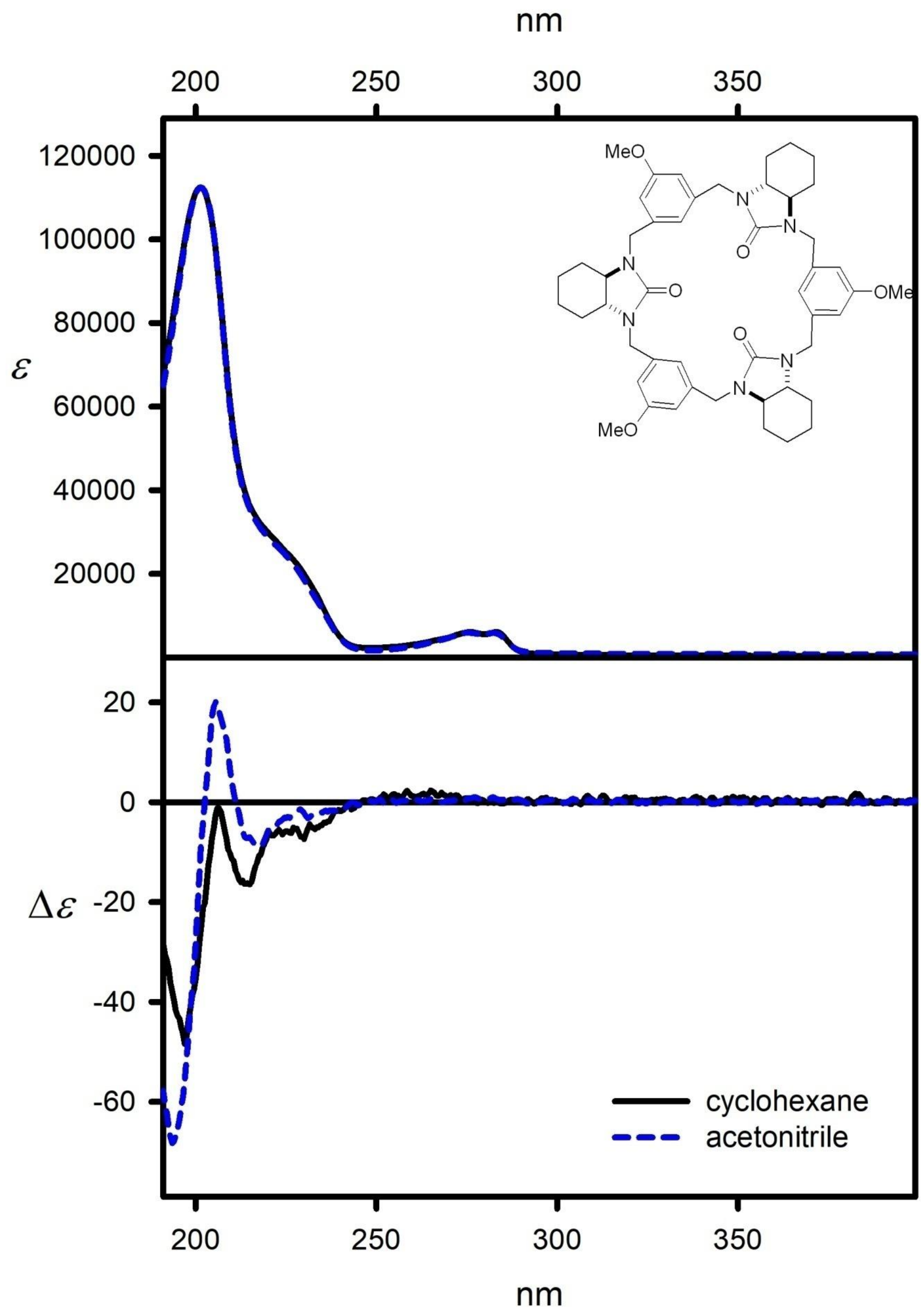

Figure S12. UV (upper panel) and ECD (lower panel) spectra of **7a** measured in cyclohexane (solid black lines) and acetonitrile (blue dashed lines).

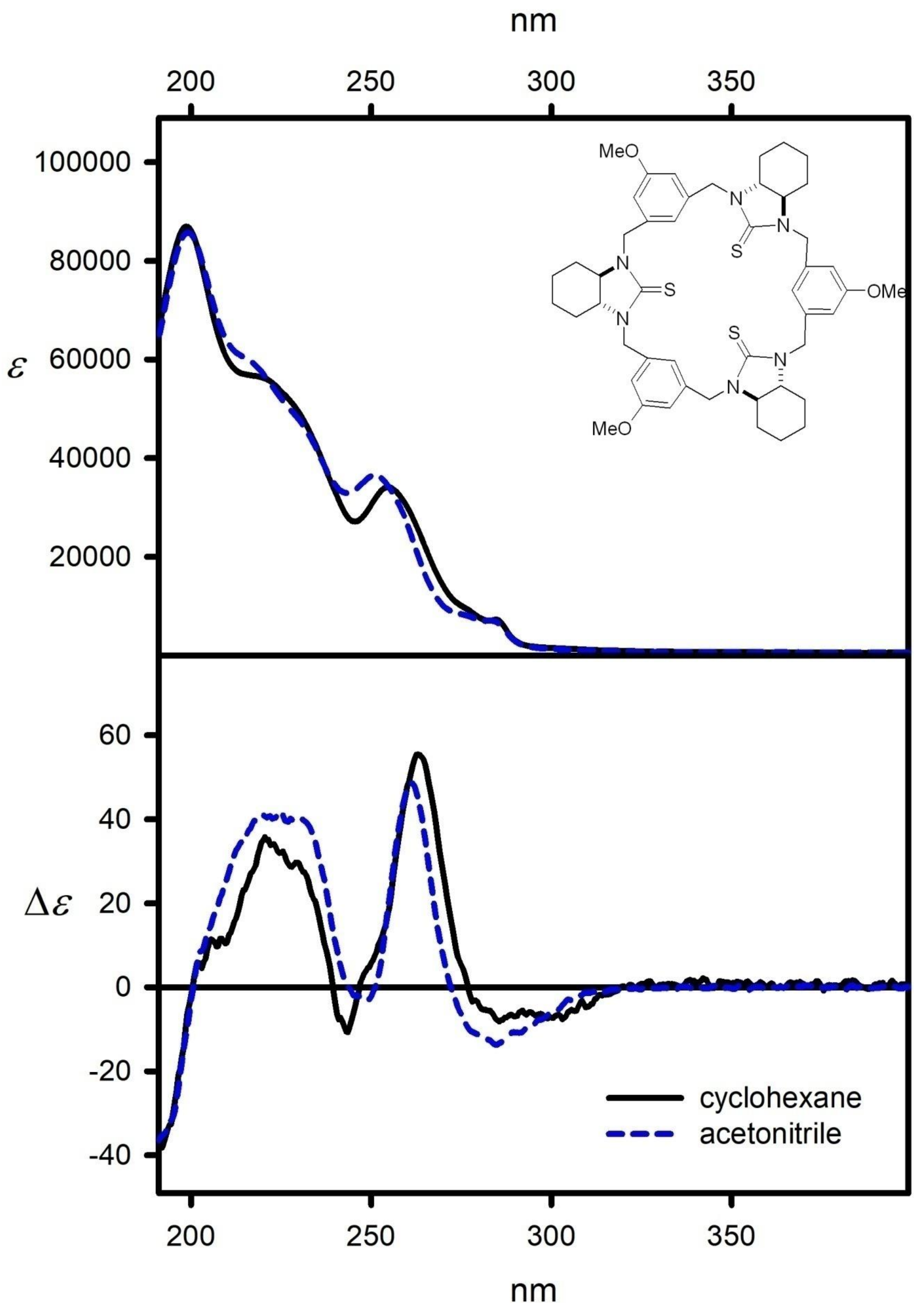

Figure S13. UV (upper panel) and ECD (lower panel) spectra of **7b** measured in cyclohexane (solid black lines) and acetonitrile (blue dashed lines).

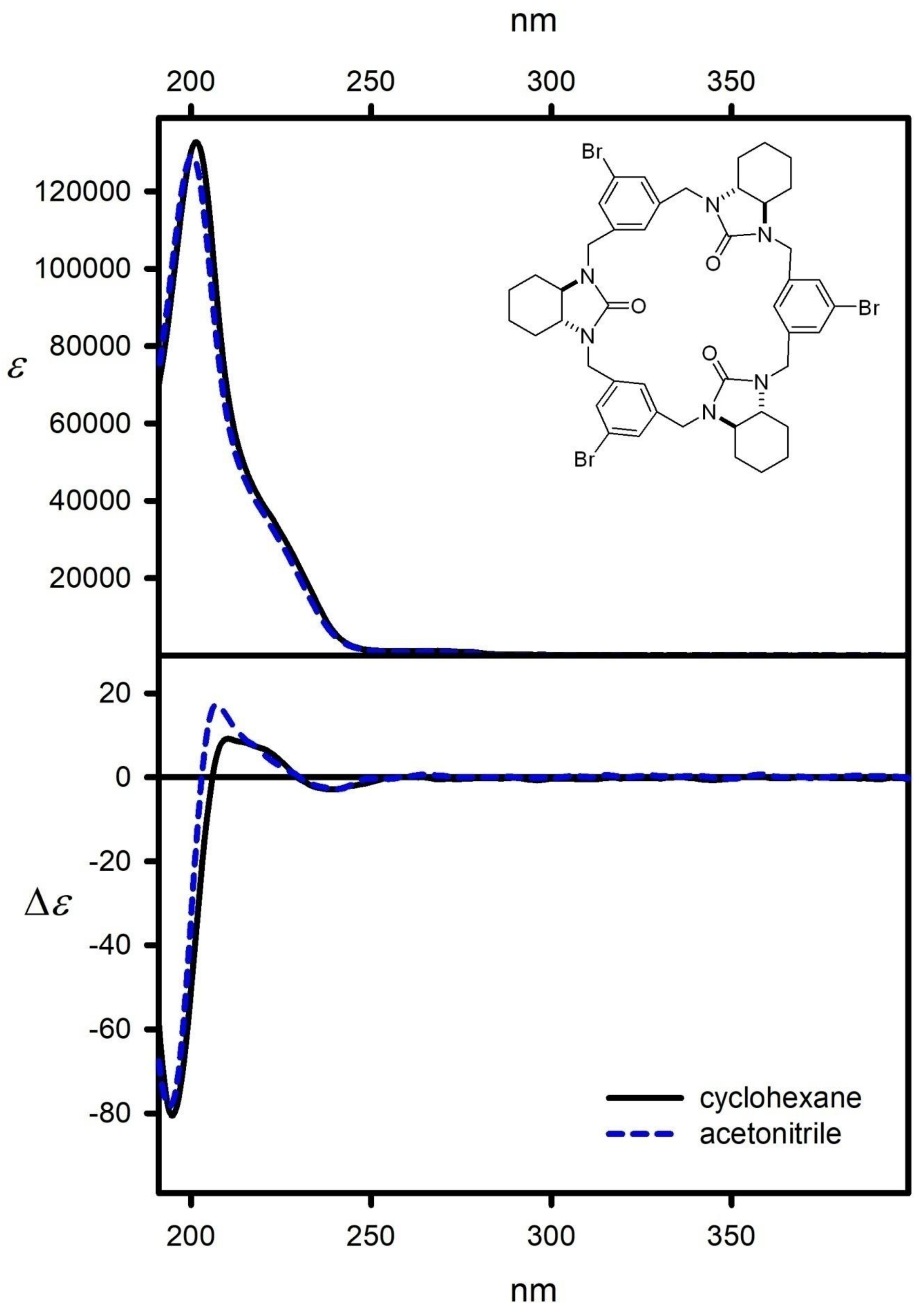

Figure S14. UV (upper panel) and ECD (lower panel) spectra of **8a** measured in cyclohexane (solid black lines) and acetonitrile (blue dashed lines).

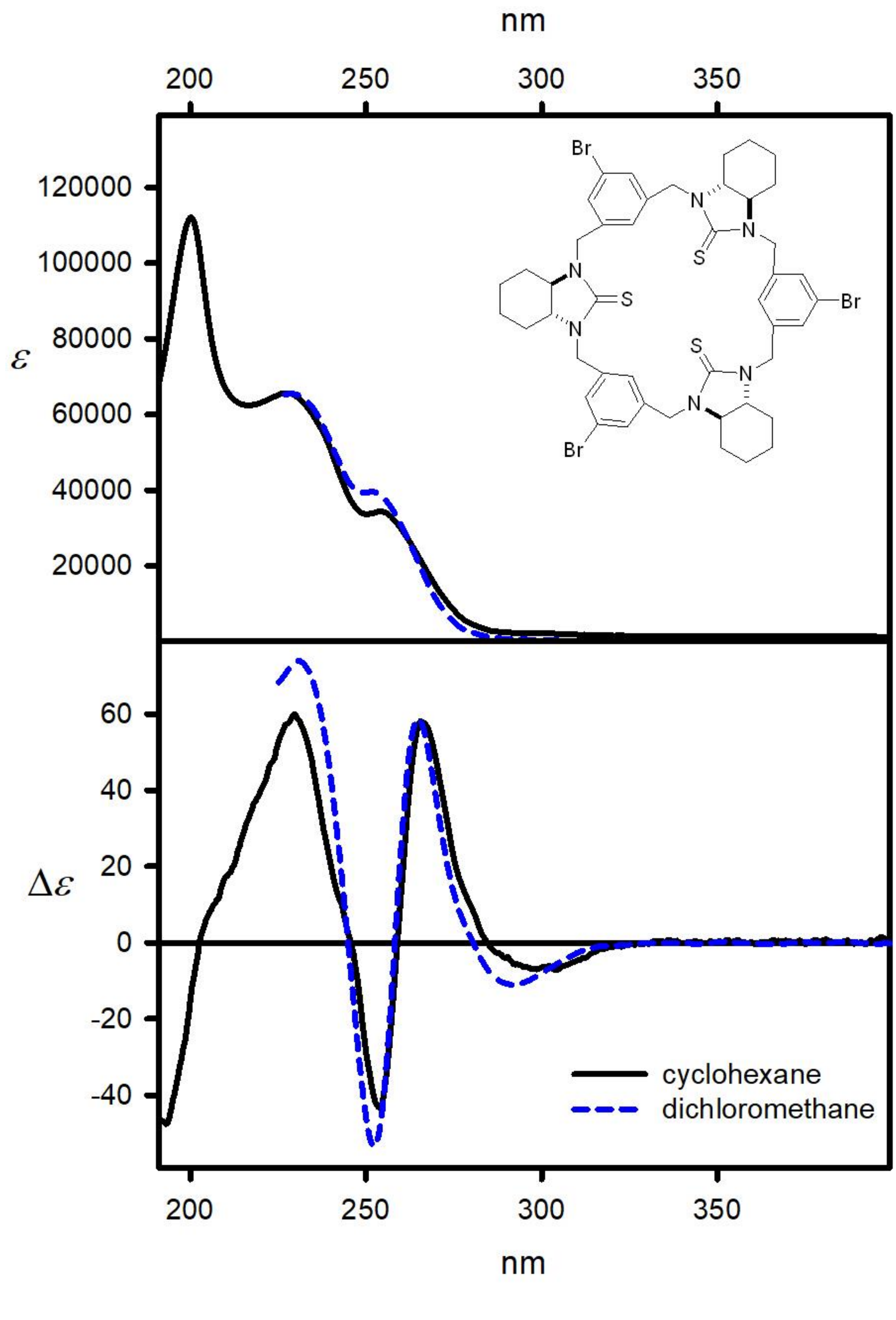

Figure S15. UV (upper panel) and ECD (lower panel) spectra of **8b** measured in cyclohexane (solid black lines) and dichloromethane (blue dashed lines).

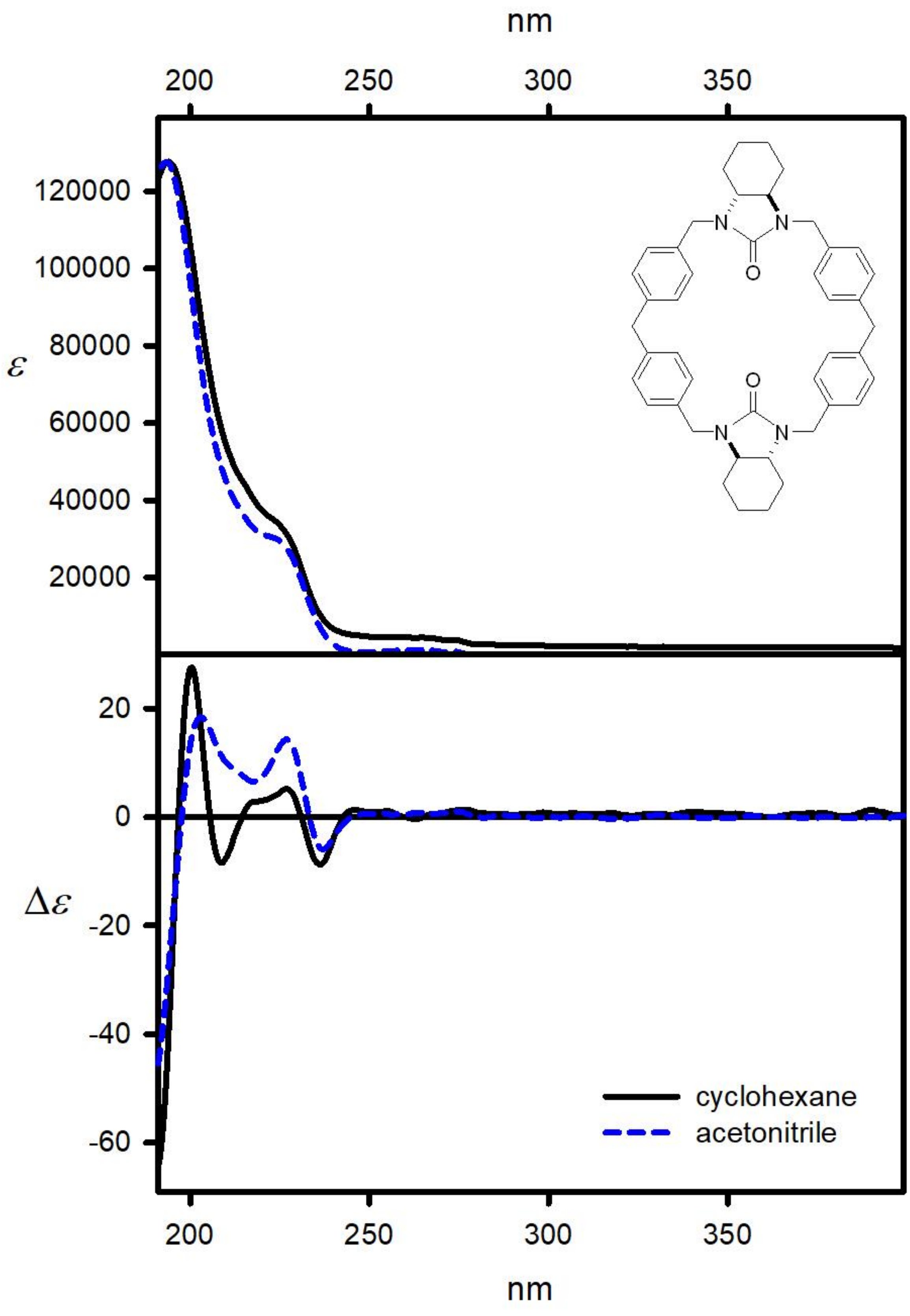

Figure S16. UV (upper panel) and ECD (lower panel) spectra of **9a** measured in cyclohexane (solid black lines) and acetonitrile (blue dashed lines).

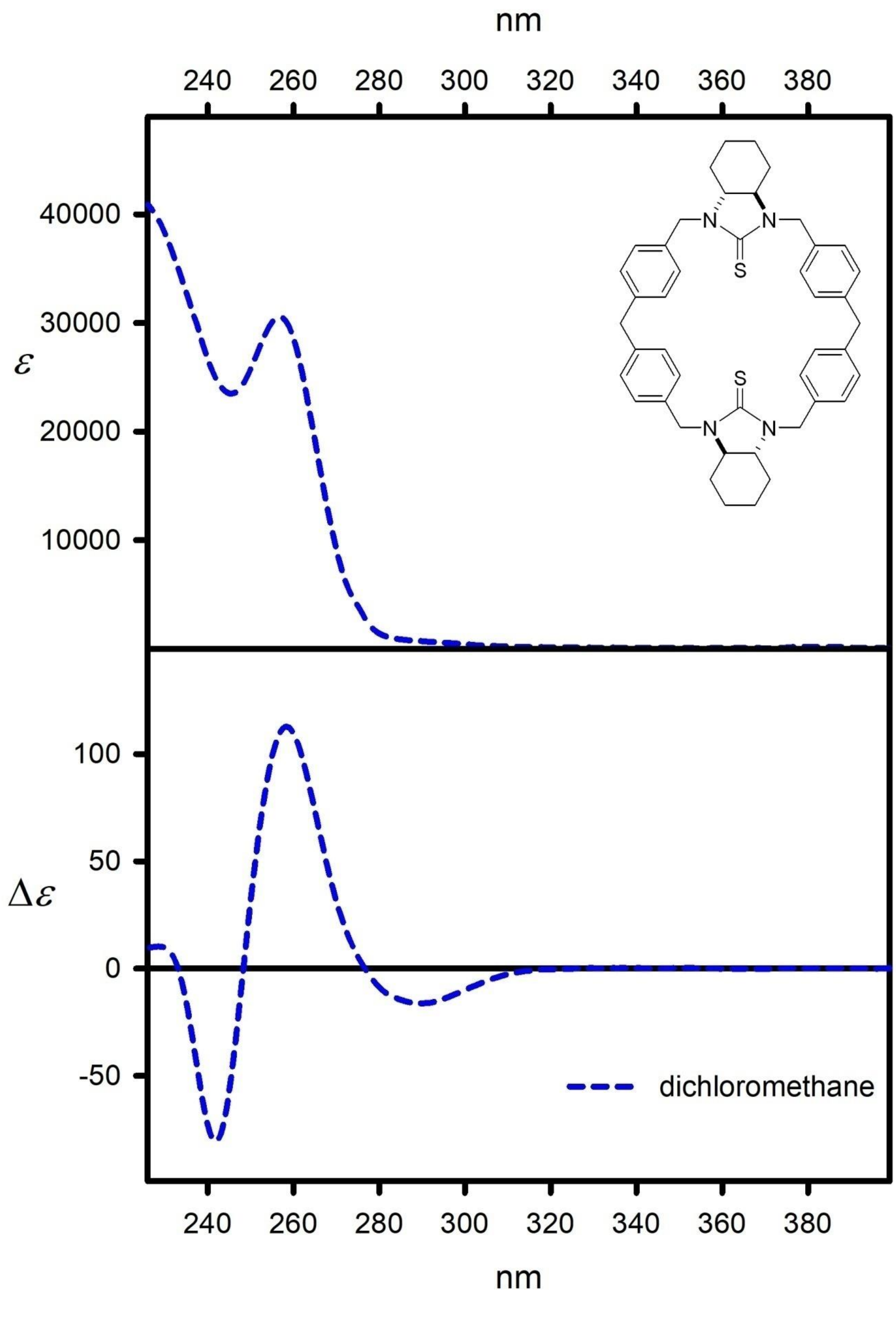

Figure S17. UV (upper panel) and ECD (lower panel) spectra of **9b** measured in dichloromethane (blue dashed lines).

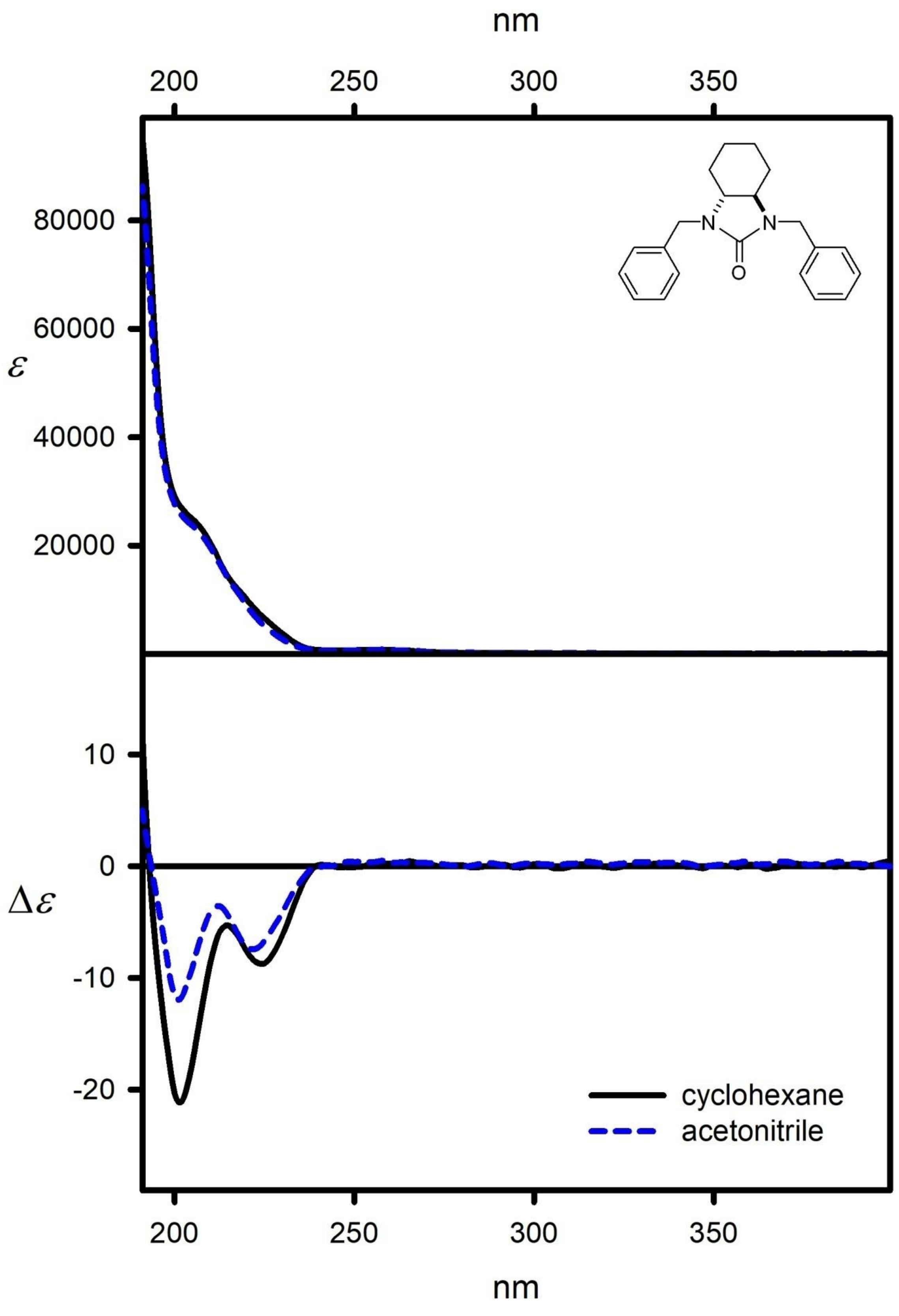

Figure S18. UV (upper panel) and ECD (lower panel) spectra of **10a** measured in cyclohexane (solid black lines) and acetonitrile (blue dashed lines).

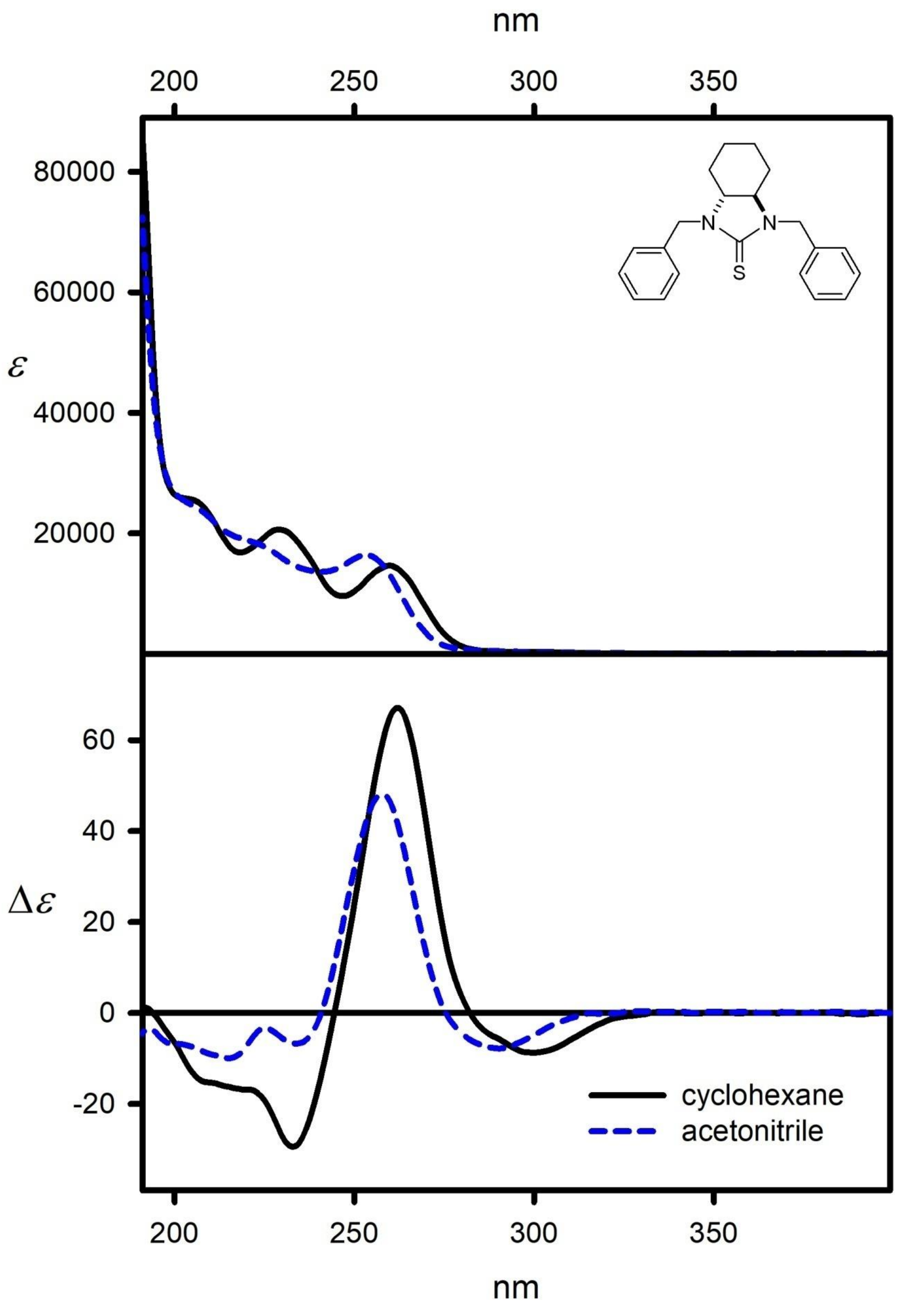

Figure S19. UV (upper panel) and ECD (lower panel) spectra of **10b** measured in cyclohexane (solid black lines) and acetonitrile (blue dashed lines).

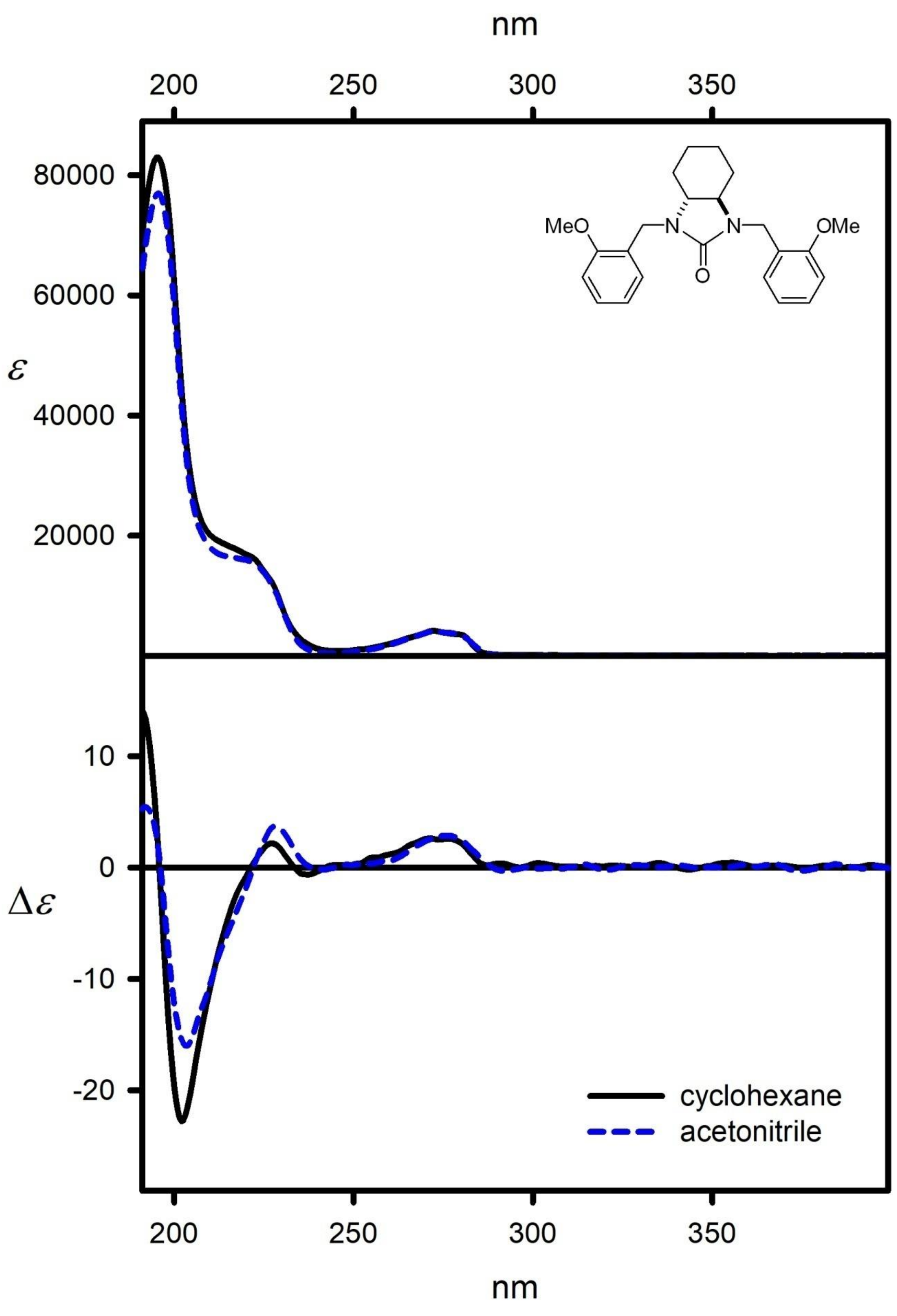

Figure S20. UV (upper panel) and ECD (lower panel) spectra of **11a** measured in cyclohexane (solid black lines) and acetonitrile (blue dashed lines).

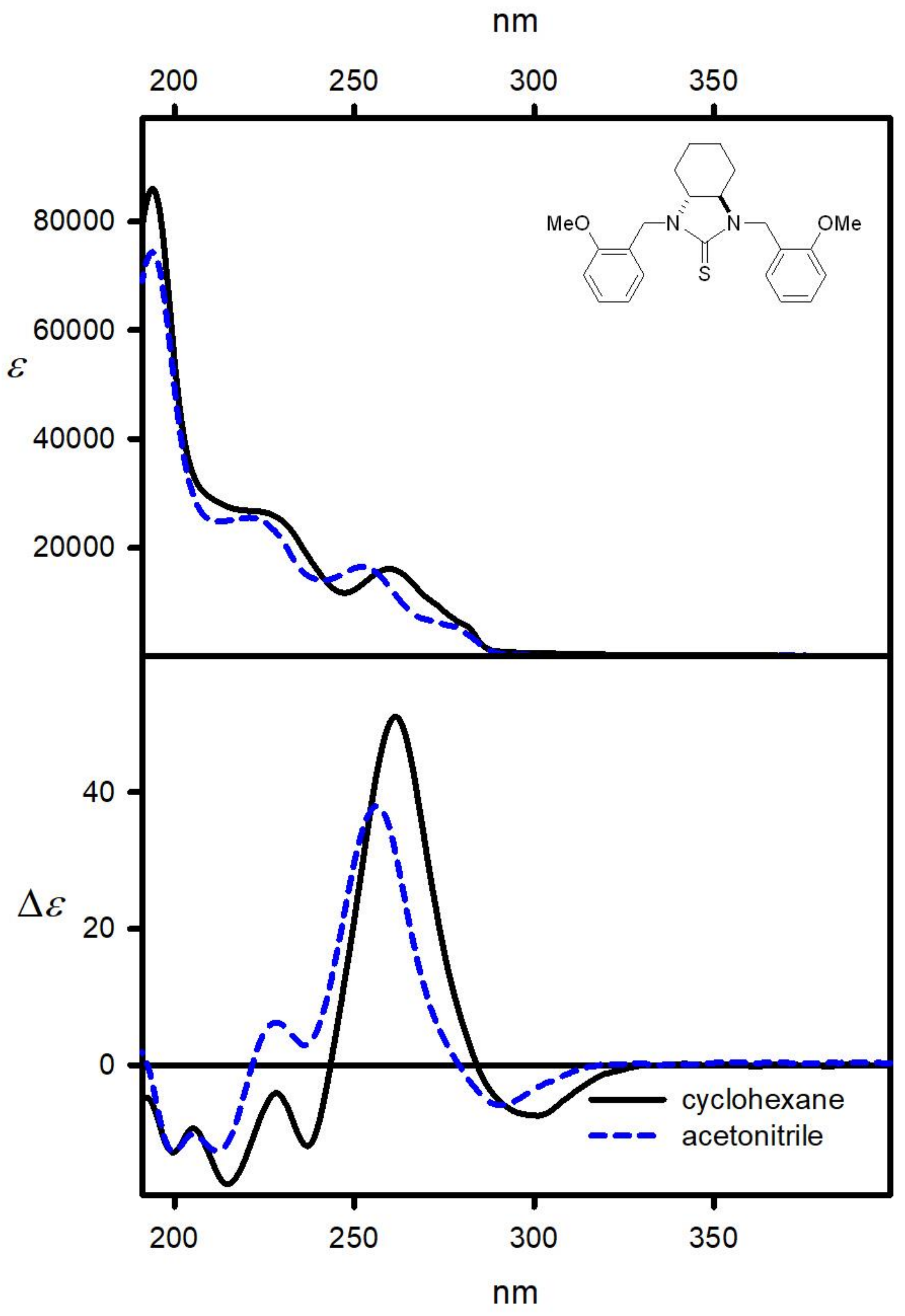

Figure S21. UV (upper panel) and ECD (lower panel) spectra of **11b** measured in cyclohexane (solid black lines) and acetonitrile (blue dashed lines).

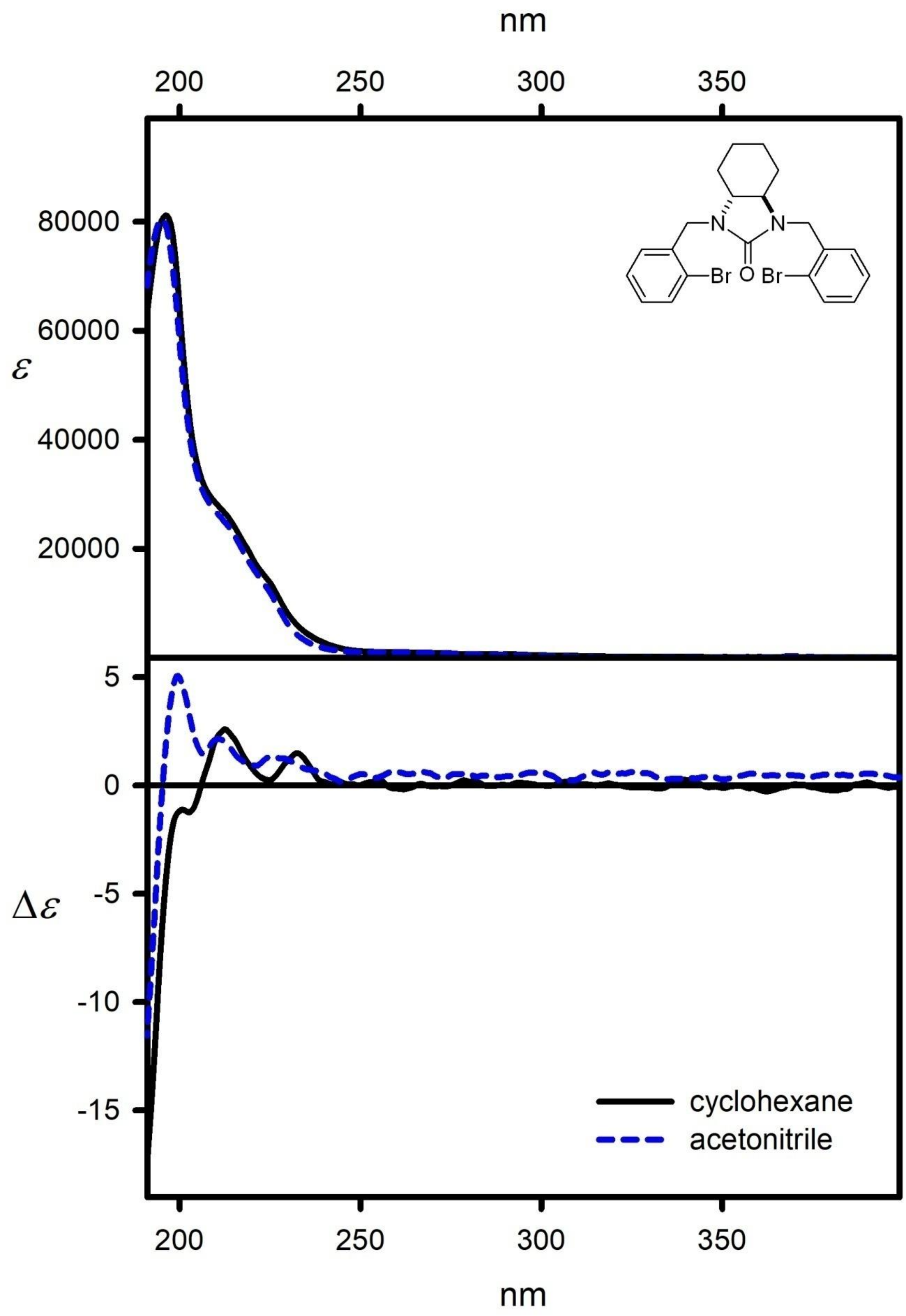

Figure S22. UV (upper panel) and ECD (lower panel) spectra of **12a** measured in cyclohexane (solid black lines) and acetonitrile (blue dashed lines).

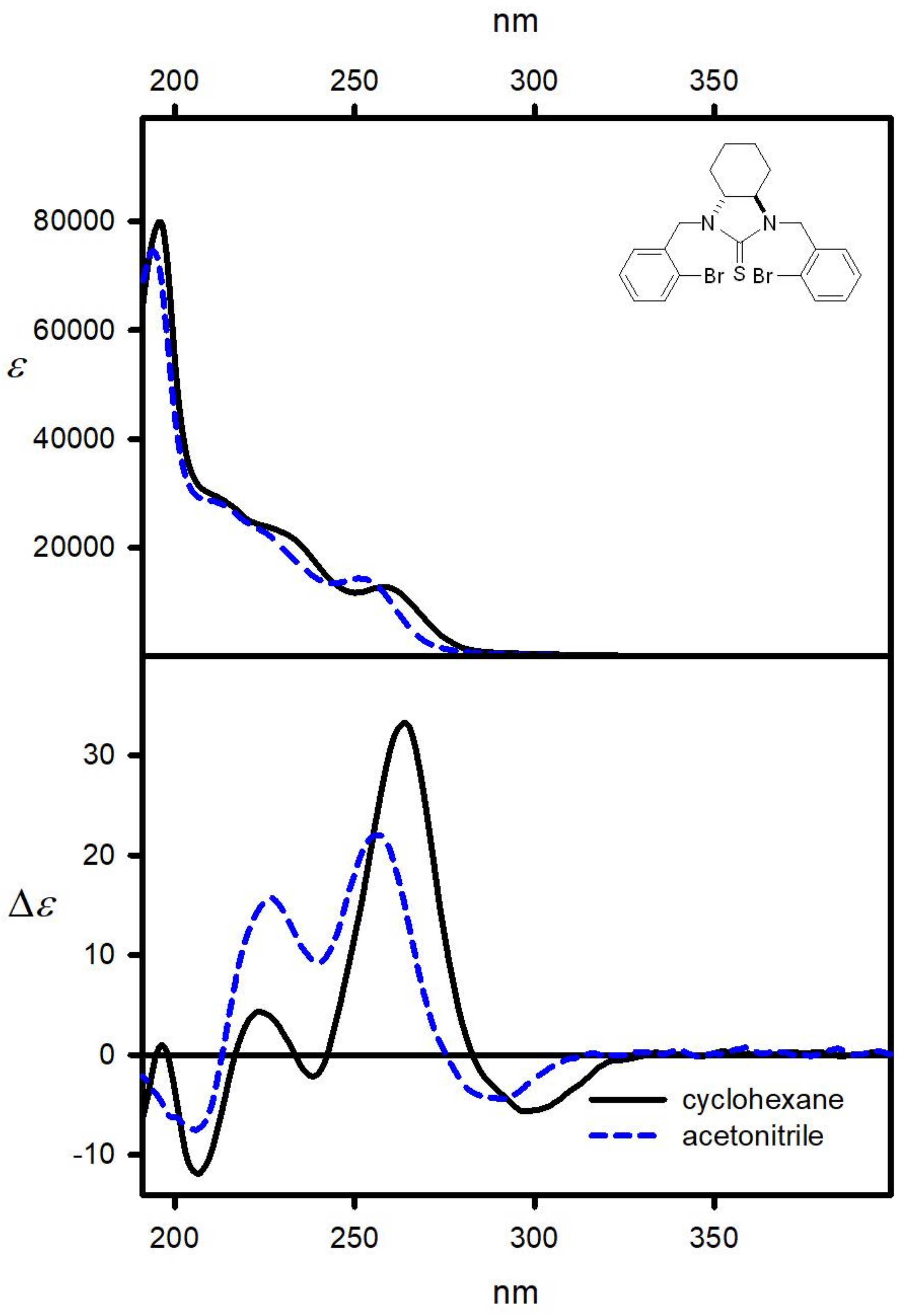

Figure S23. UV (upper panel) and ECD (lower panel) spectra of **12b** measured in cyclohexane (solid black lines) and acetonitrile (blue dashed lines).

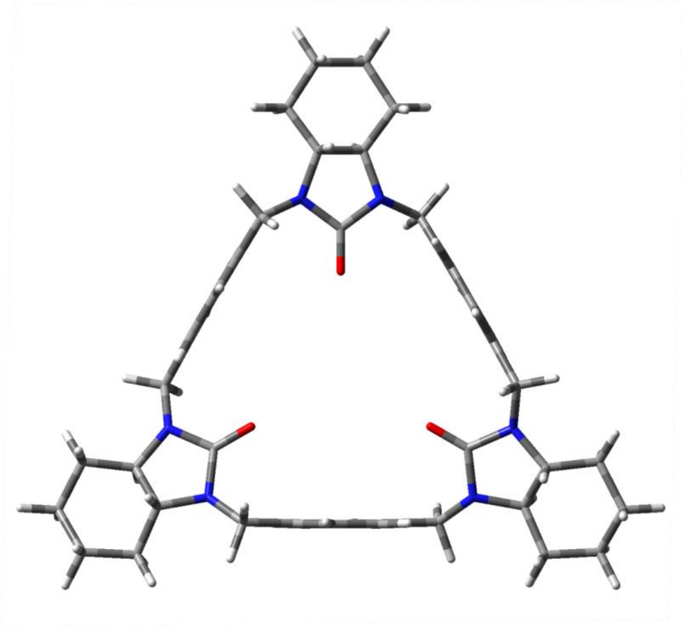

conf. 1

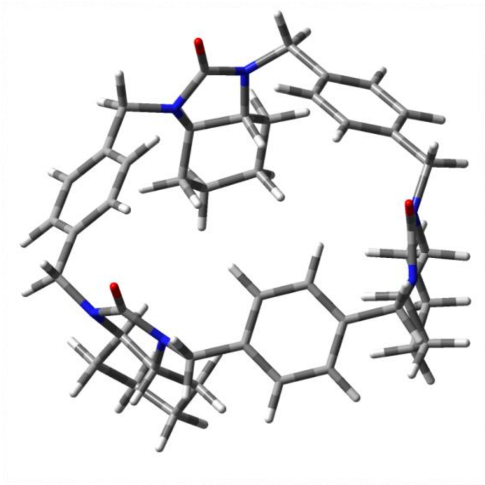

conf. 2

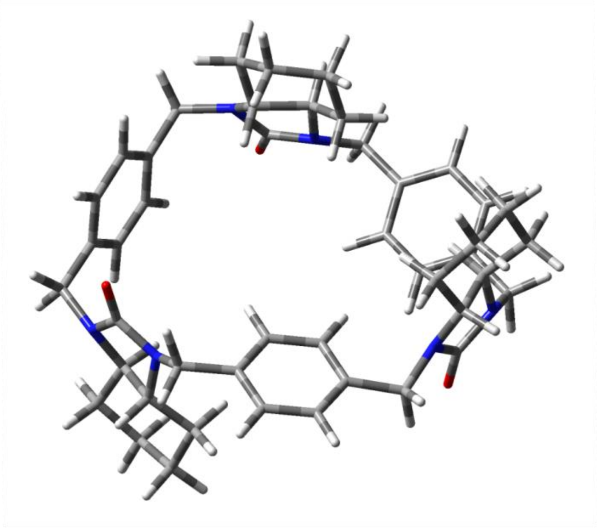

conf. 3

Figure S24. Structures of individual low-energy conformers of **1a** calculated at the B3LYP/6-311G(d,p) level.

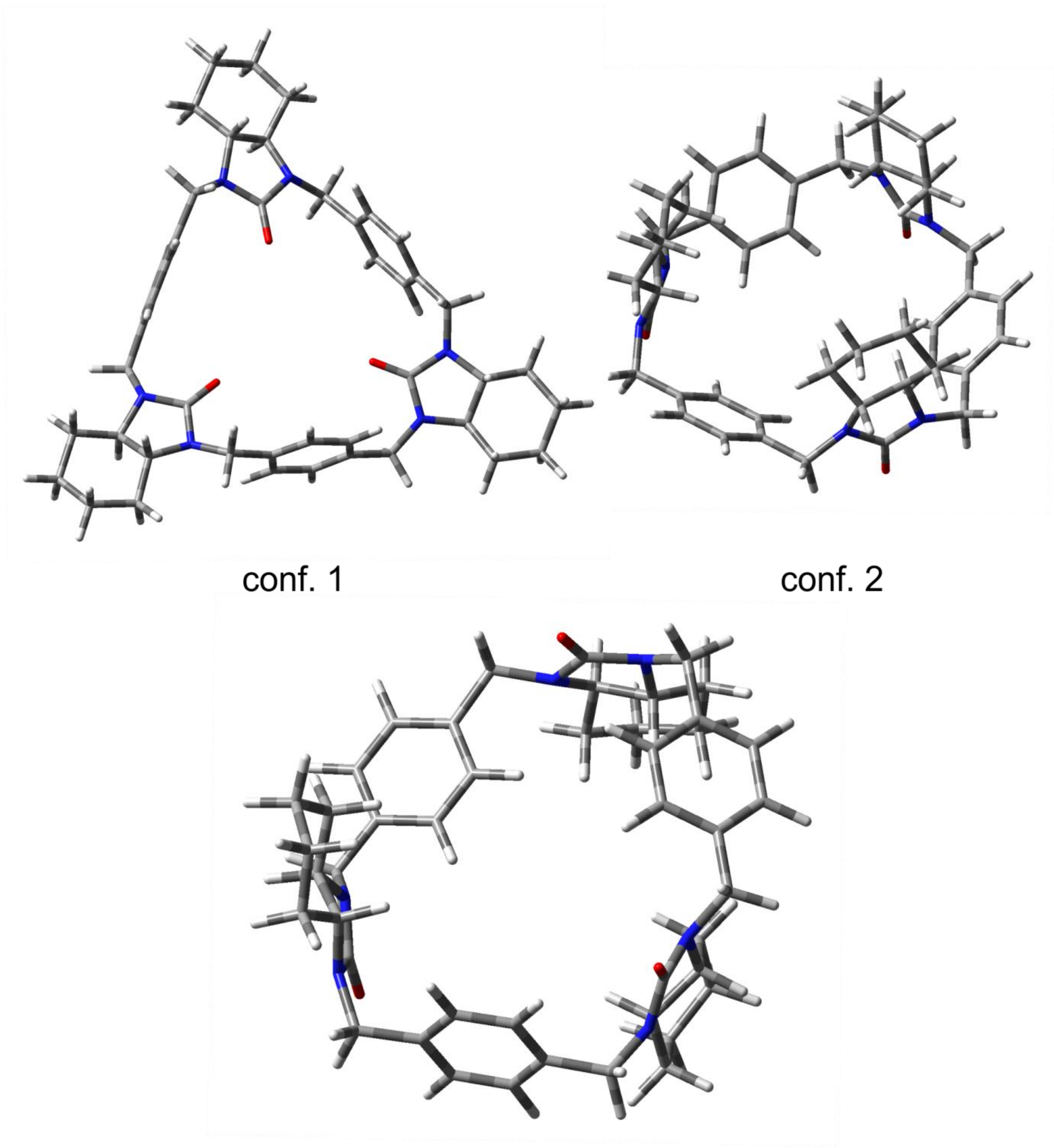

Figure S25. Structures of individual low-energy conformers of **1a** calculated at the IEFPCM(ACN)/B3LYP/6-311G(d,p) level.

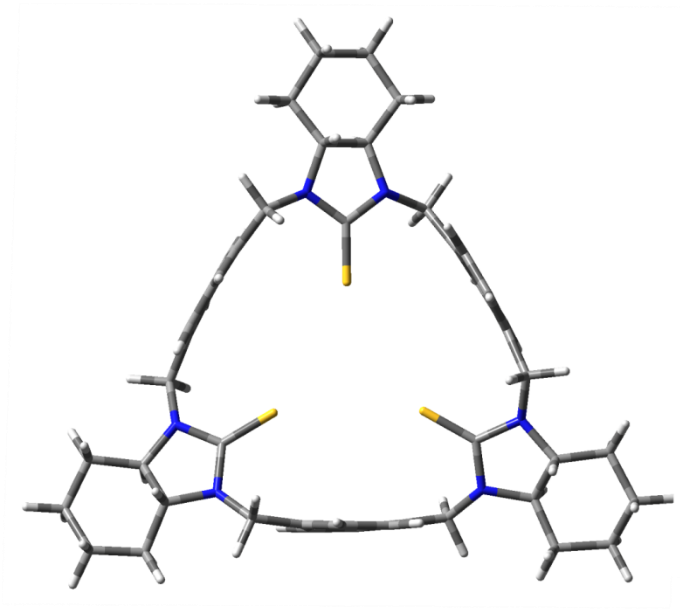

conf. 1

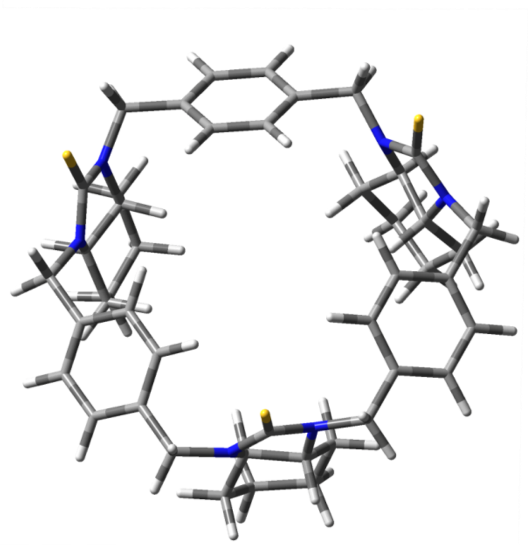

conf. 2

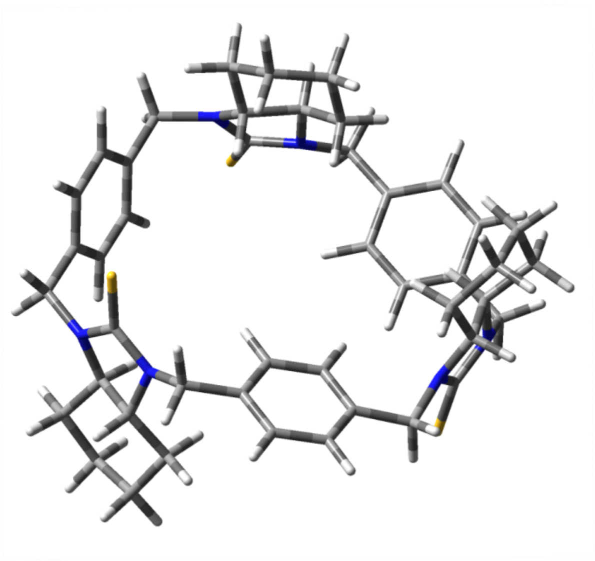

conf. 3

Figure S26. Structures of individual low-energy conformers of **1b** calculated at the B3LYP/6-311G(d,p) level.

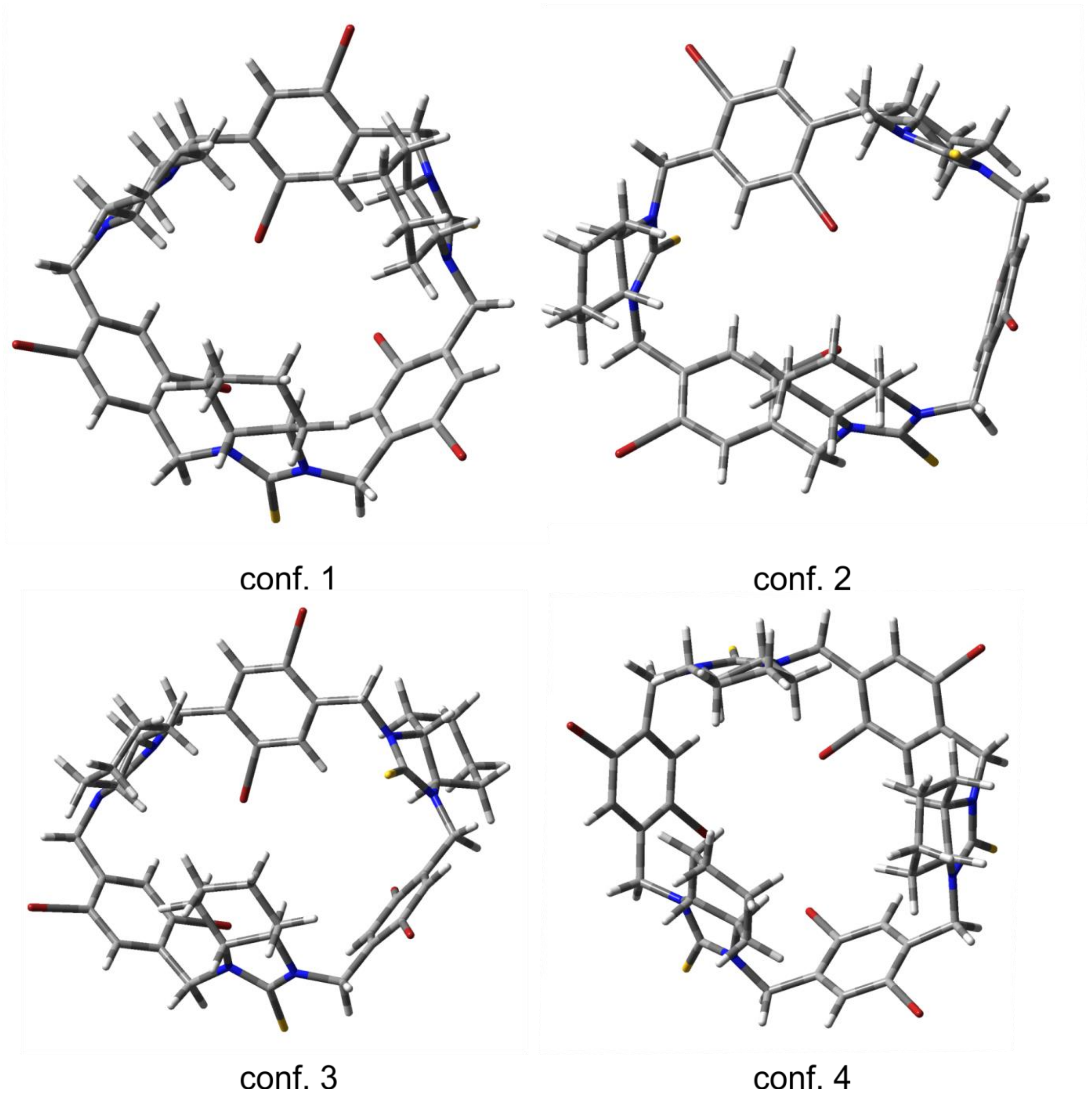

Figure S27. Structures of individual low-energy conformers of **3** calculated at the B3LYP/6-311G(d,p) level.

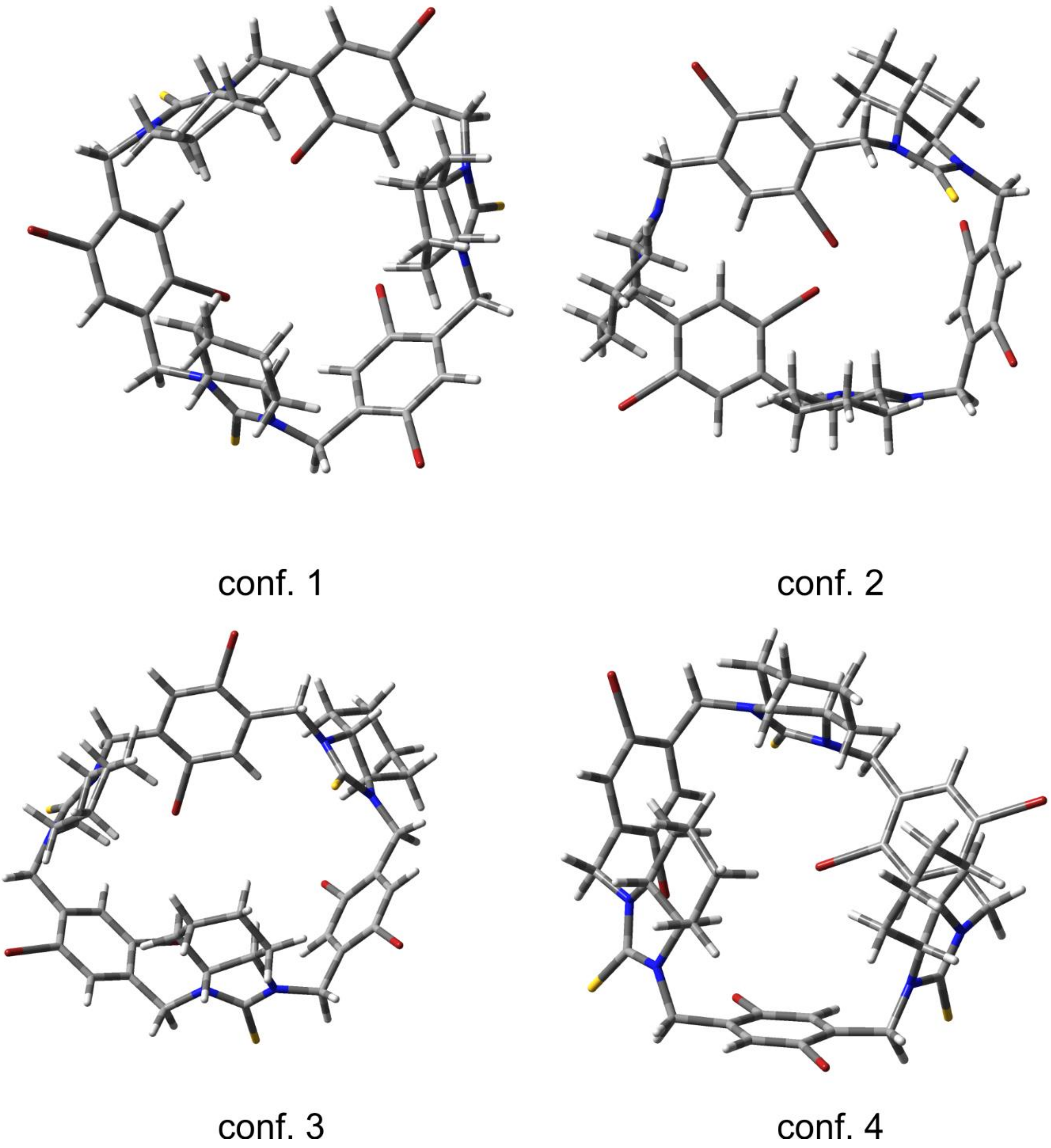

Figure S28. Structures of individual low-energy conformers of **3** calculated at the IEFPCM(CH_2Cl_2)/B3LYP/6-311G(d,p) level.

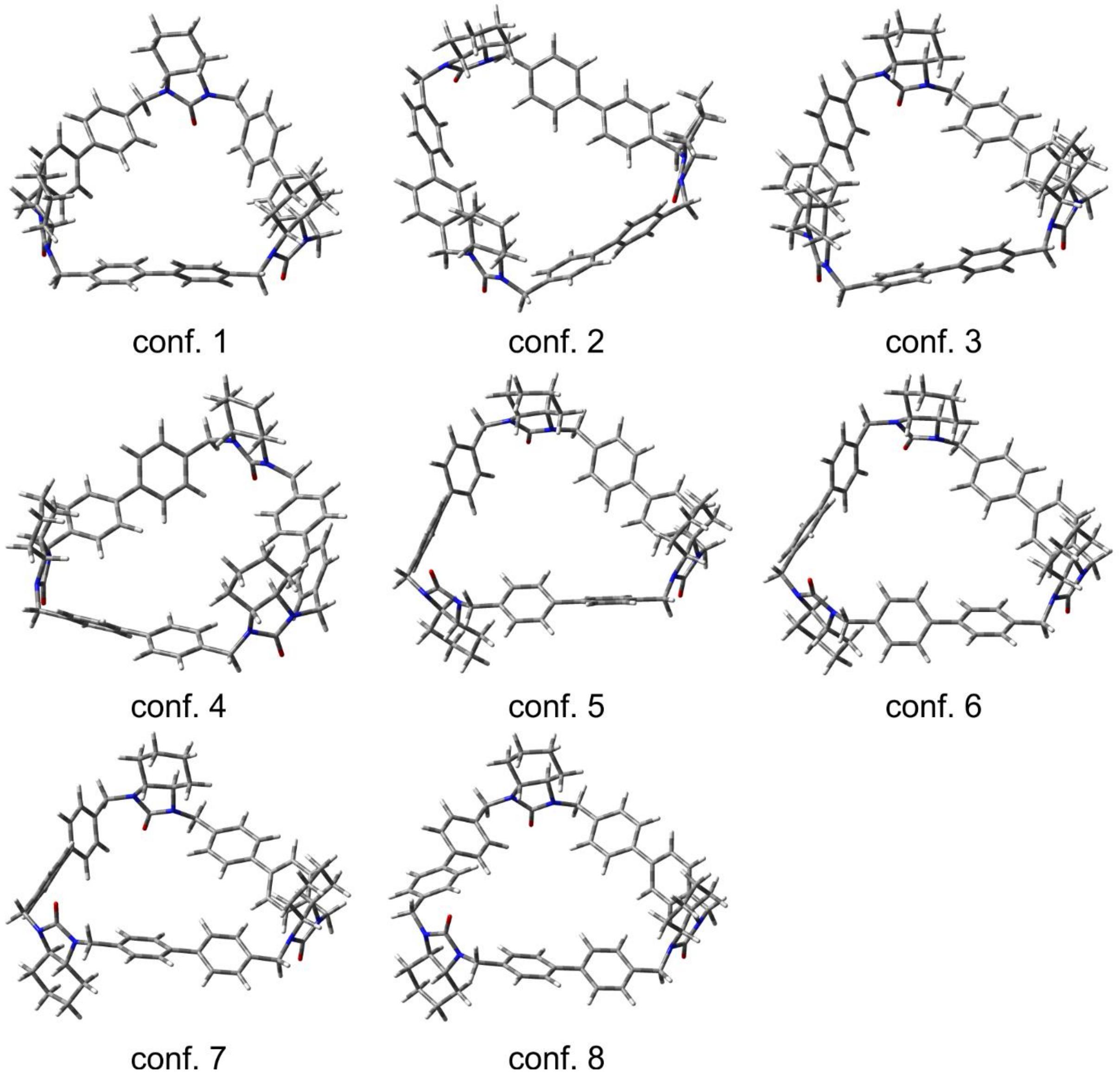

Figure S29. Structures of individual low-energy conformers of **4** calculated at the B3LYP/6-311G(d,p) level.

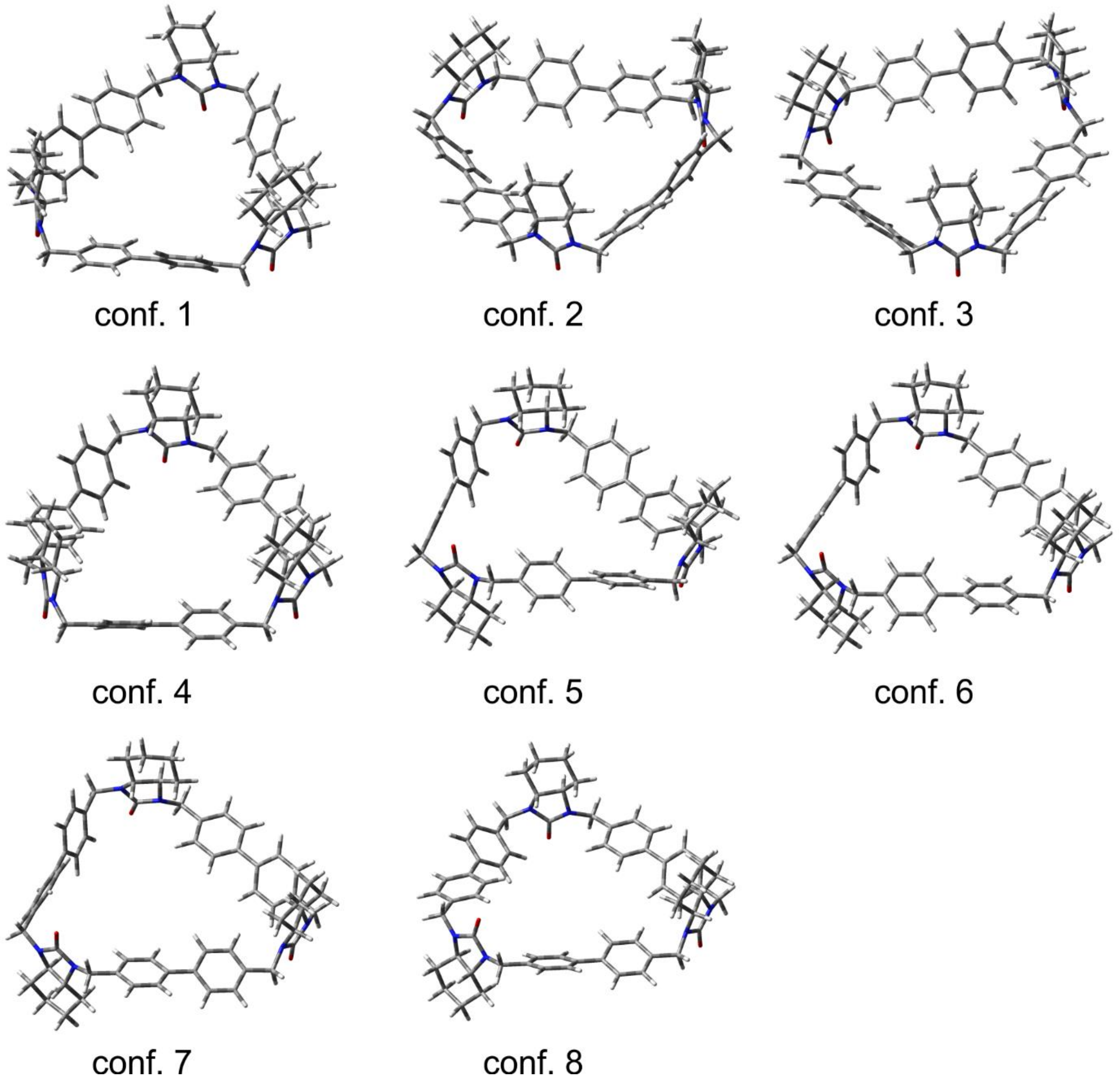

Figure S30. Structures of individual low-energy conformers of **4** calculated at the IEFPCM(ACN)/B3LYP/6-311G(d,p) level.

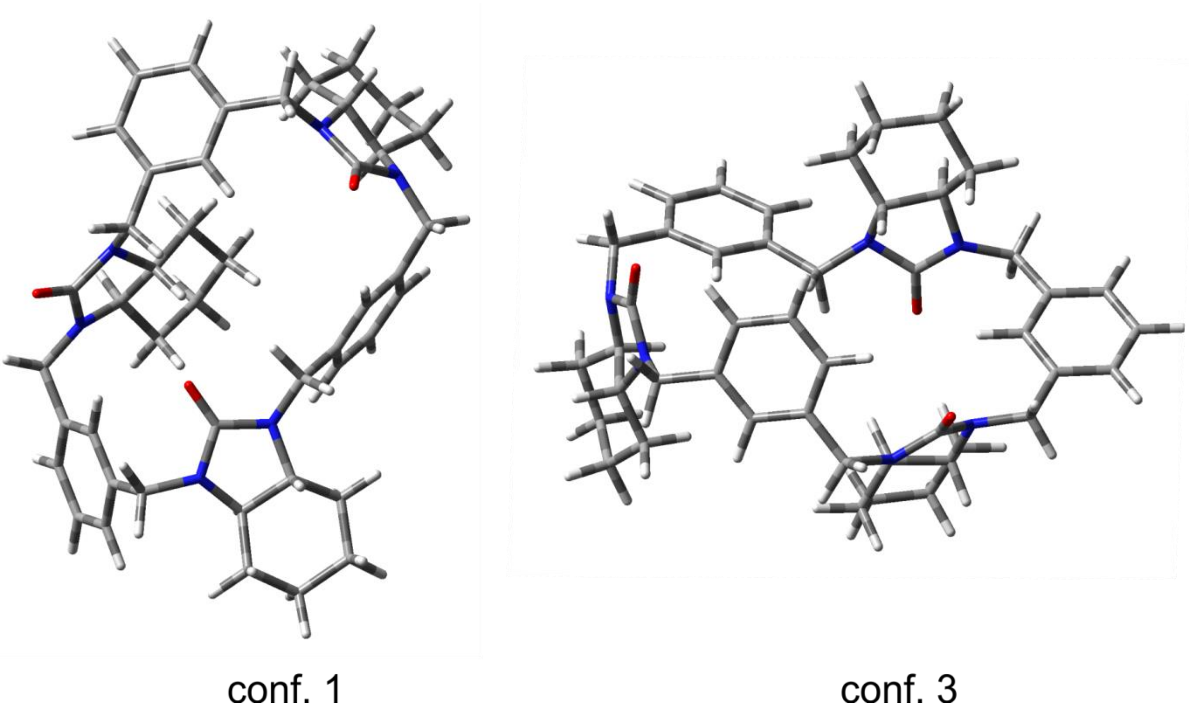

Figure S31. Structures of individual low-energy conformers of **5a** calculated at the B3LYP/6-311G(d,p) level.

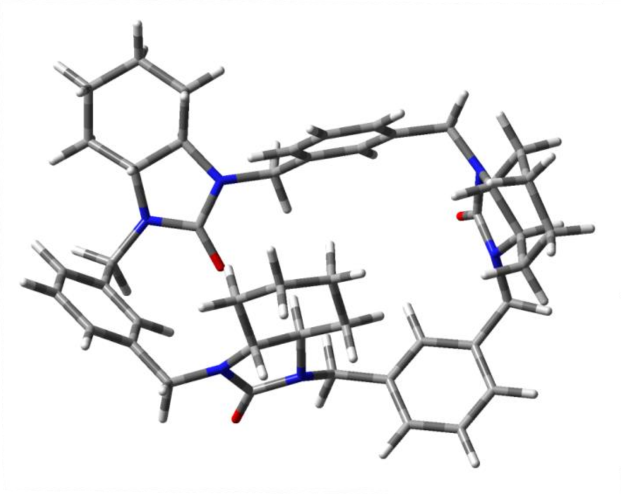

conf. 1

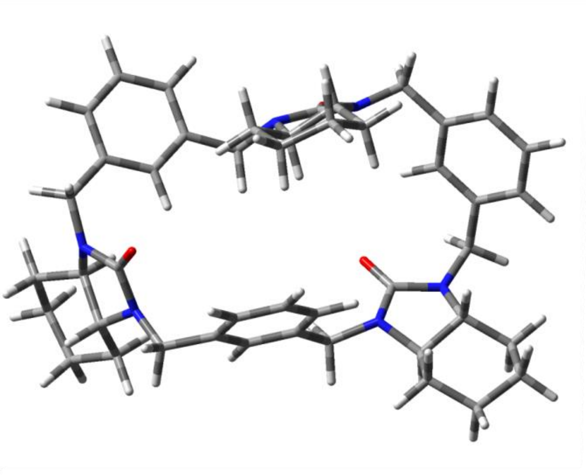

conf. 2

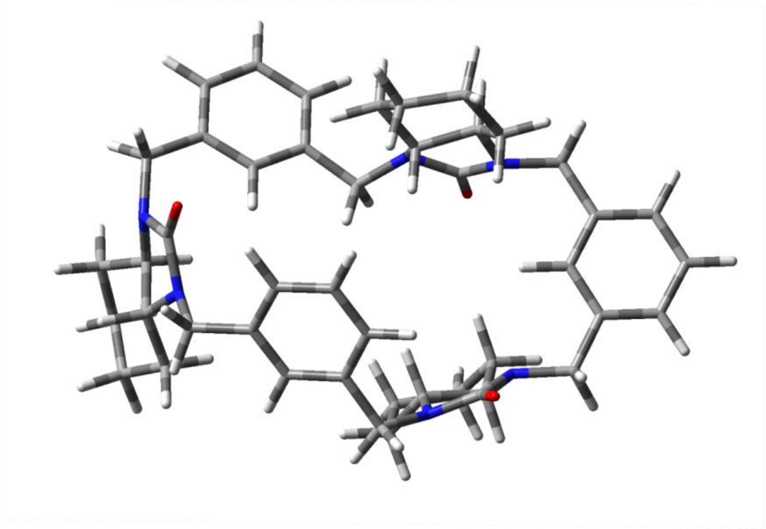

conf. 3

Figure S32. Structures of individual low-energy conformers of **5a** calculated at the IEFPCM(ACN)/B3LYP/6-311G(d,p) level.

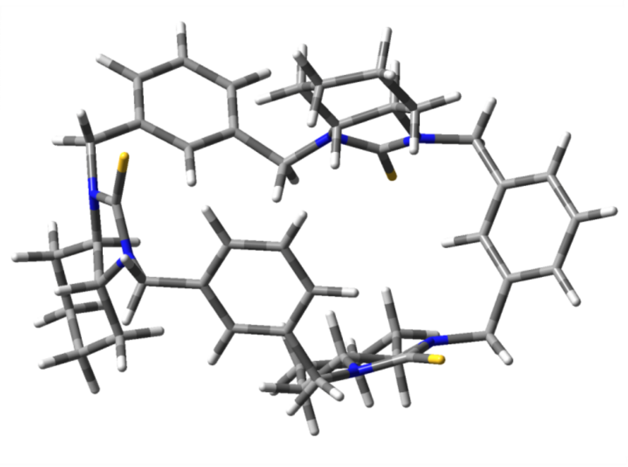

conf. 1

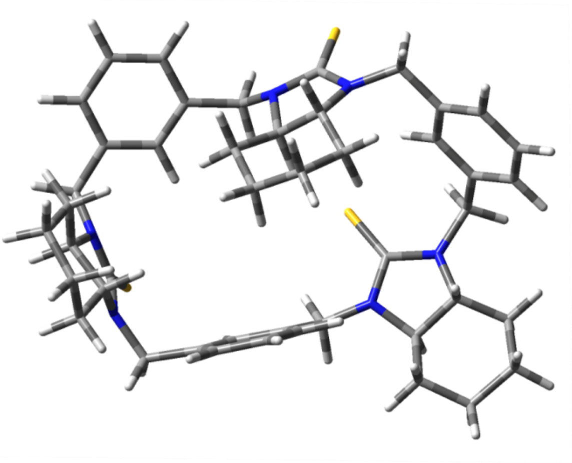

conf. 2

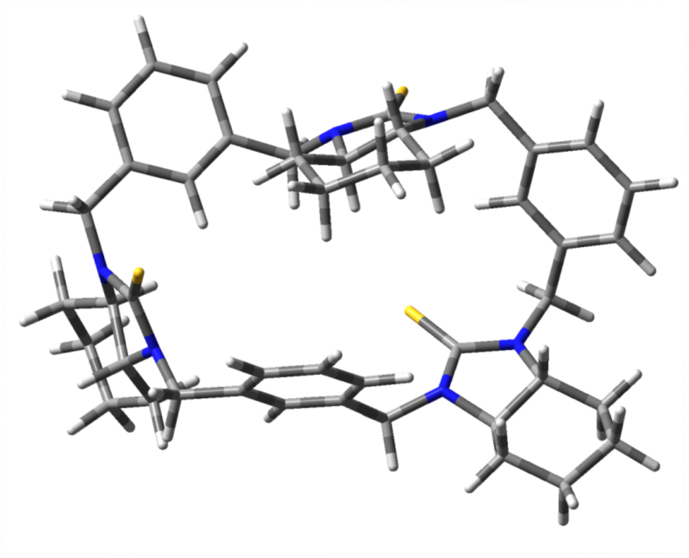

conf. 3

Figure S33. Structures of individual low-energy conformers of **5b** calculated at the B3LYP/6-311G(d,p) level.

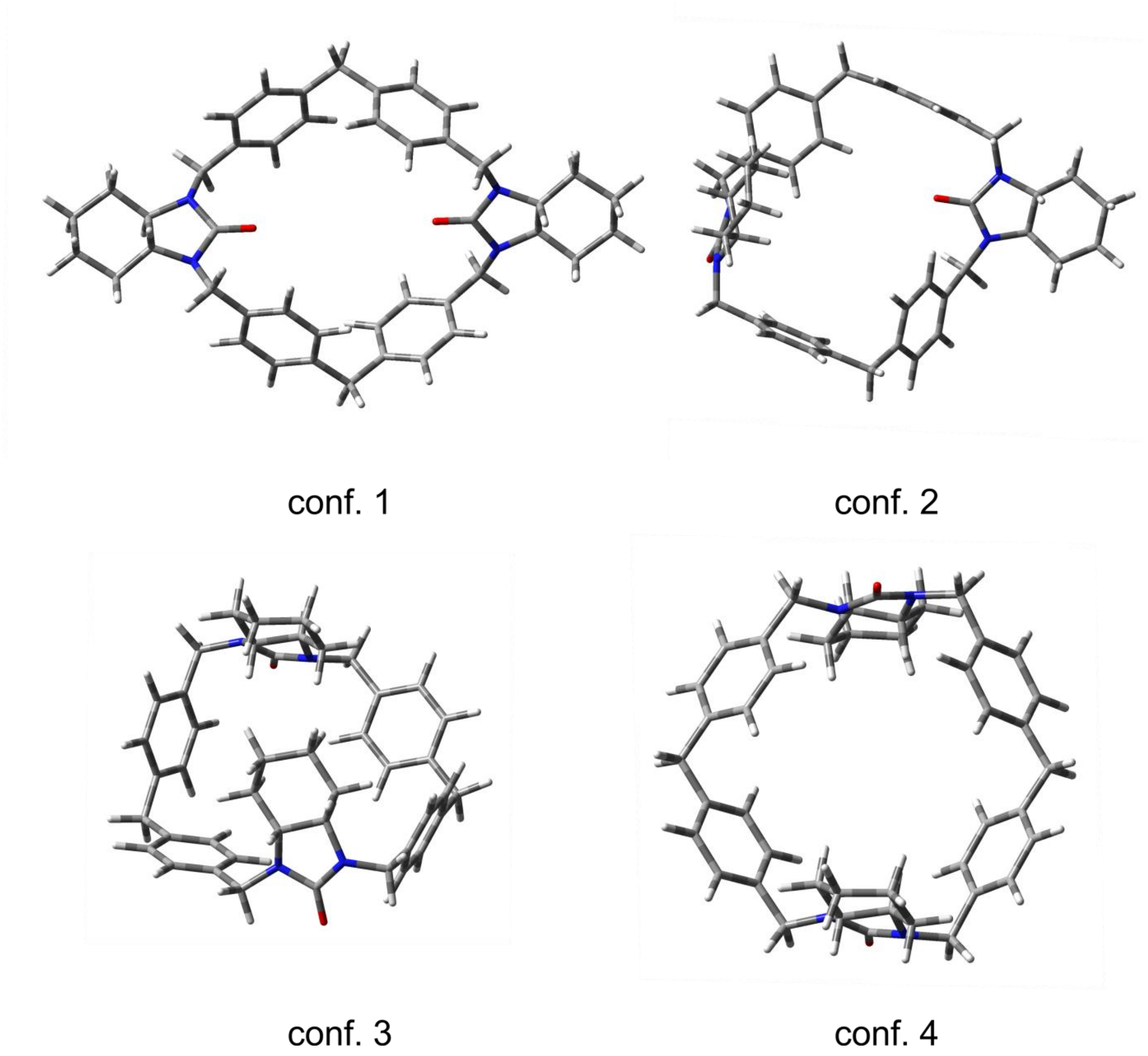

Figure S34. Structures of individual low-energy conformers of **9a** calculated at the B3LYP/6-311G(d,p) level.

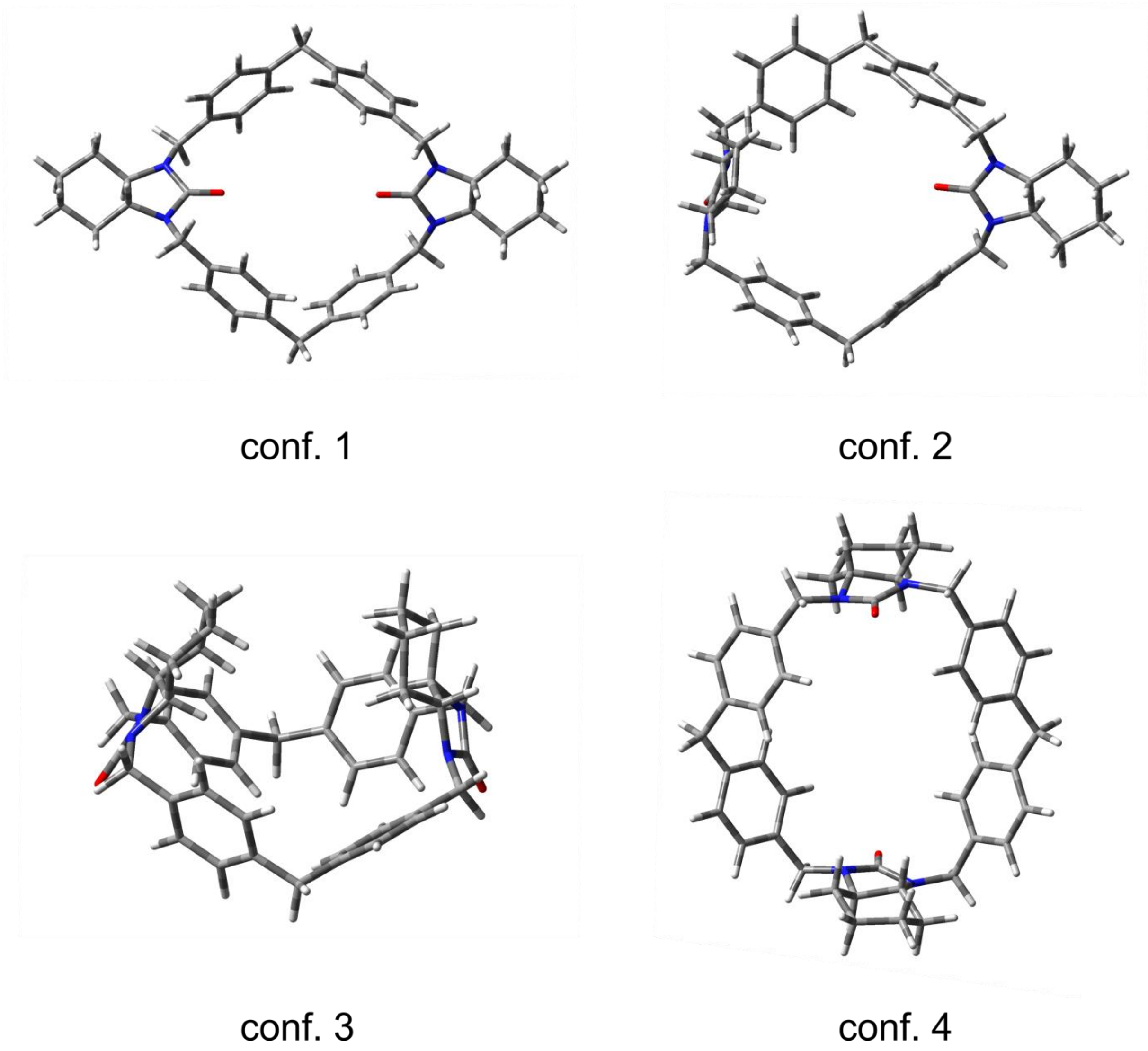

Figure S35. Structures of individual low-energy conformers of **9a** calculated at the IEFPCM(ACN)/B3LYP/6-311G(d,p) level.

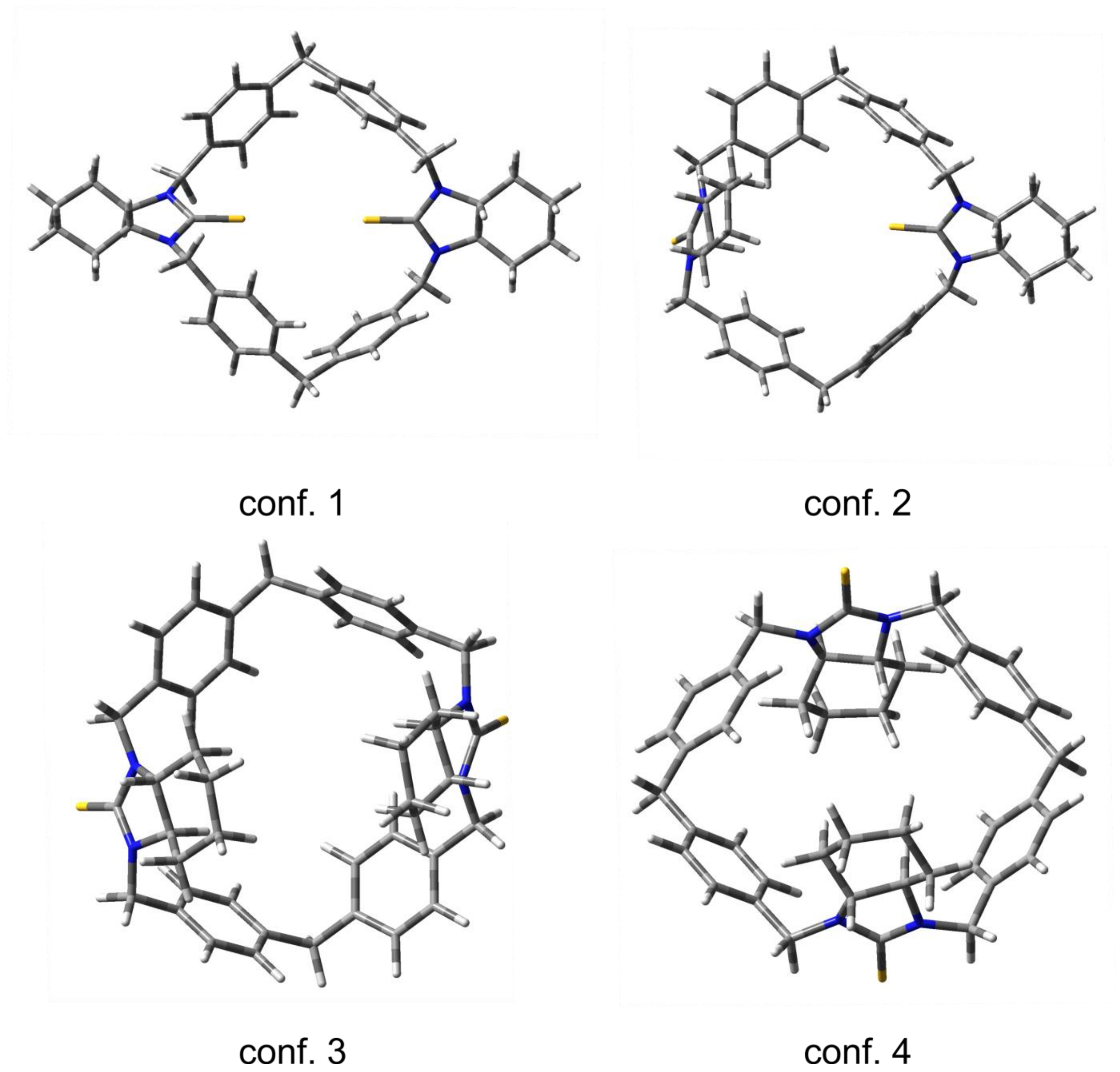

Figure S36. Structures of individual low-energy conformers of **9b** calculated at the B3LYP/6-311G(d,p) level.

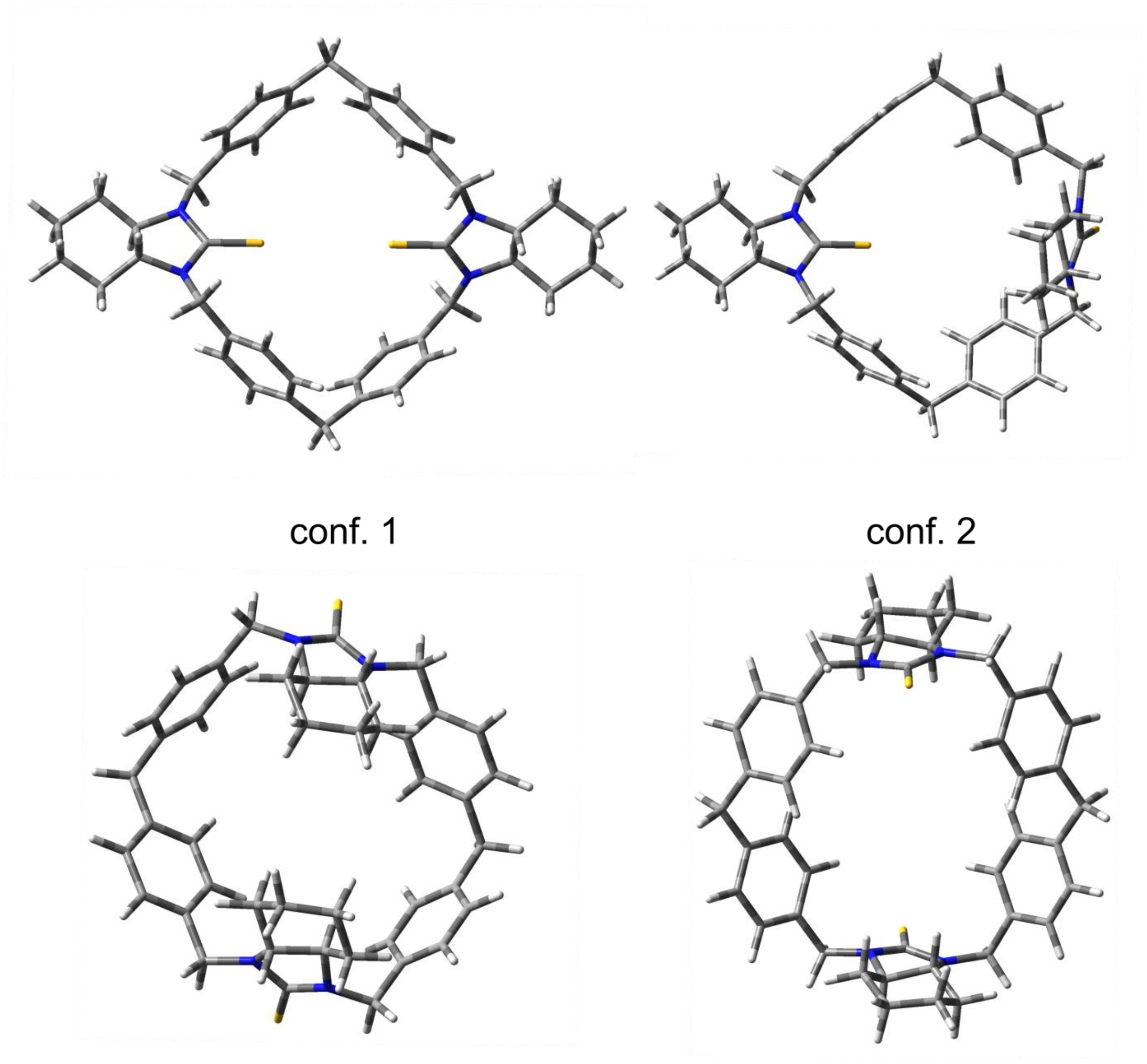

conf. 1 conf. 2

conf. 3 conf. 4

Figure S37. Structures of individual low-energy conformers of **9b** calculated at the IEFPCM(CH_2Cl_2)/B3LYP/6-311G(d,p) level.

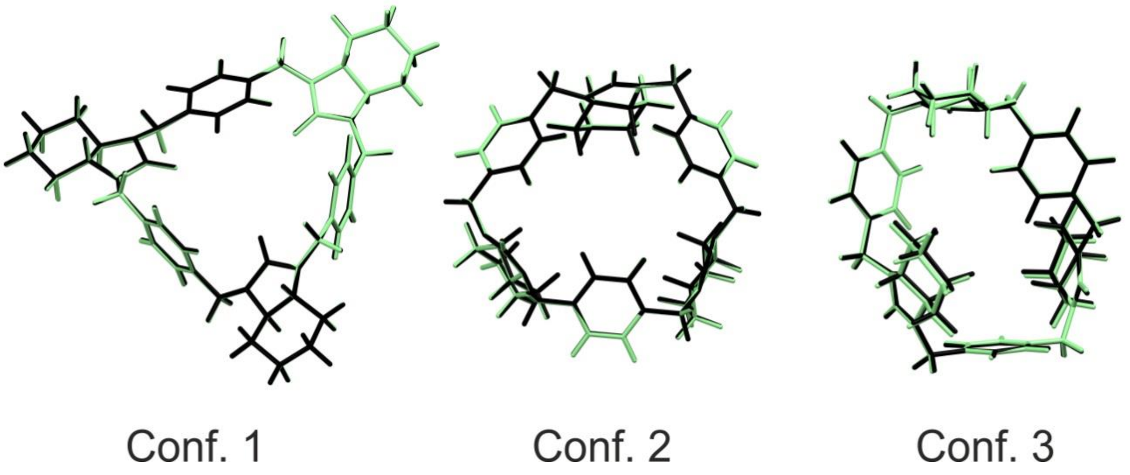

Figure S38. Overlaid of the structures of individual low-energy conformers of **1a** calculated at the DFT/6-311G(d,p) level of theory: *in vacuo* (black color) and with the use of acetonitrile solvent model (green color).

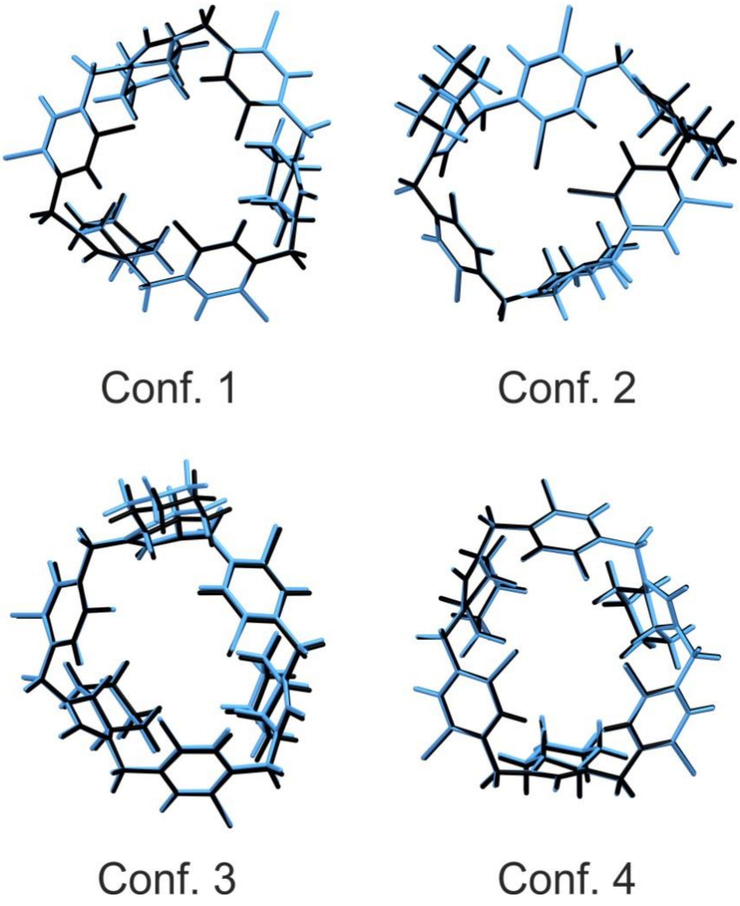

Figure S39. Overlaid of the structures of individual low-energy conformers of **3** calculated at the DFT/6-311G(d,p) level of theory: *in vacuo* (black color) and with the use of dichloromethane solvent model (blue color).

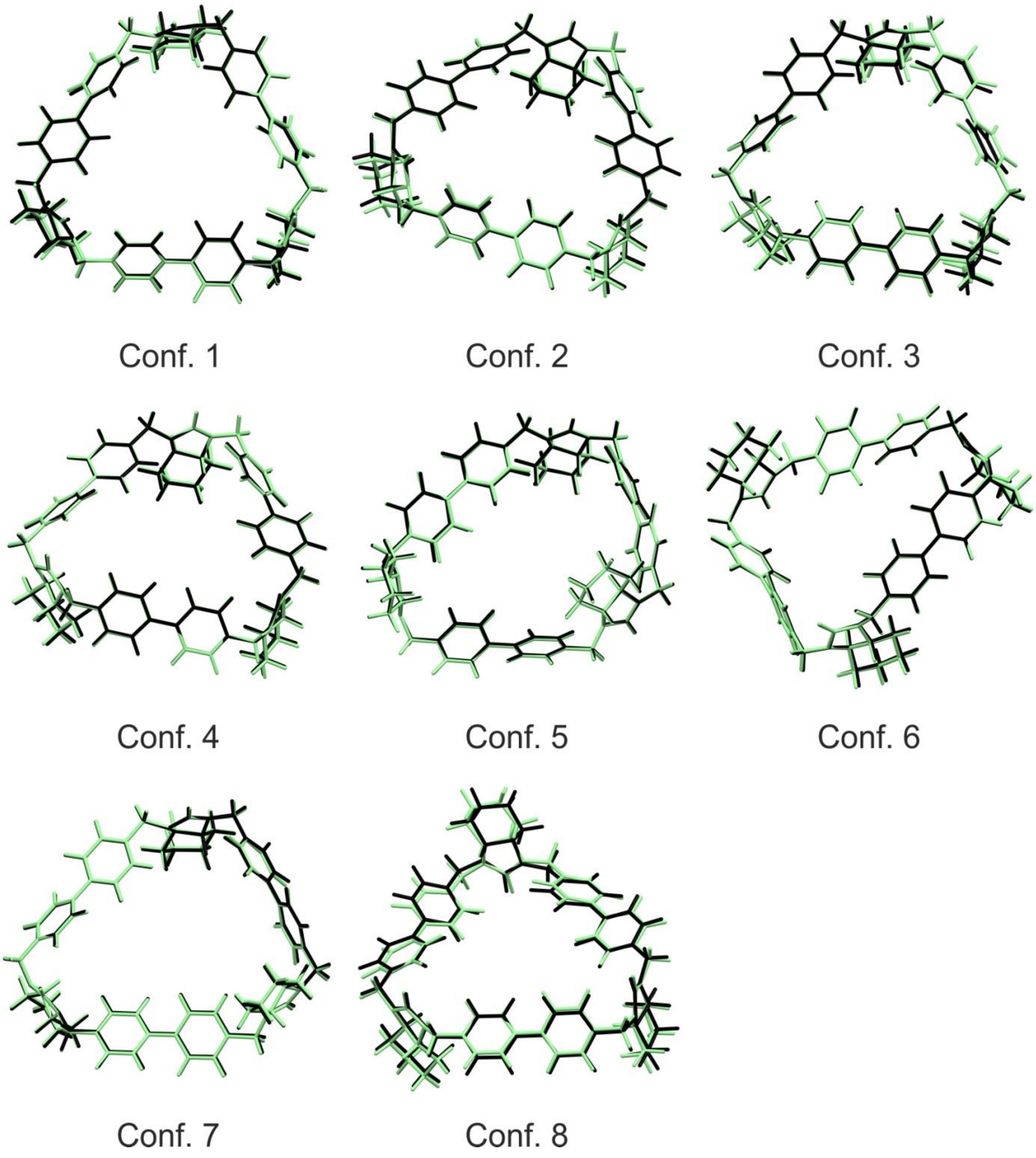

Figure S40. Overlaid of the structures of individual low-energy conformers of **4** calculated at the DFT/6-311G(d,p) level of theory: *in vacuo* (black color) and with the use of acetonitrile solvent model (green color).

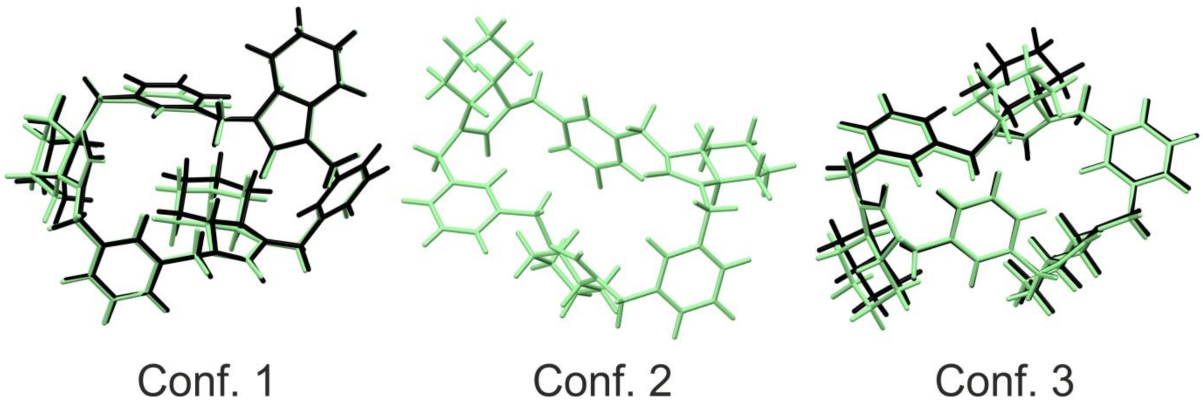

Figure S41. Overlaid of the structures of individual low-energy conformers of **5a** calculated at the DFT/6-311G(d,p) level of theory: *in vacuo* (black color) and with the use of acetonitrile solvent model (green color).

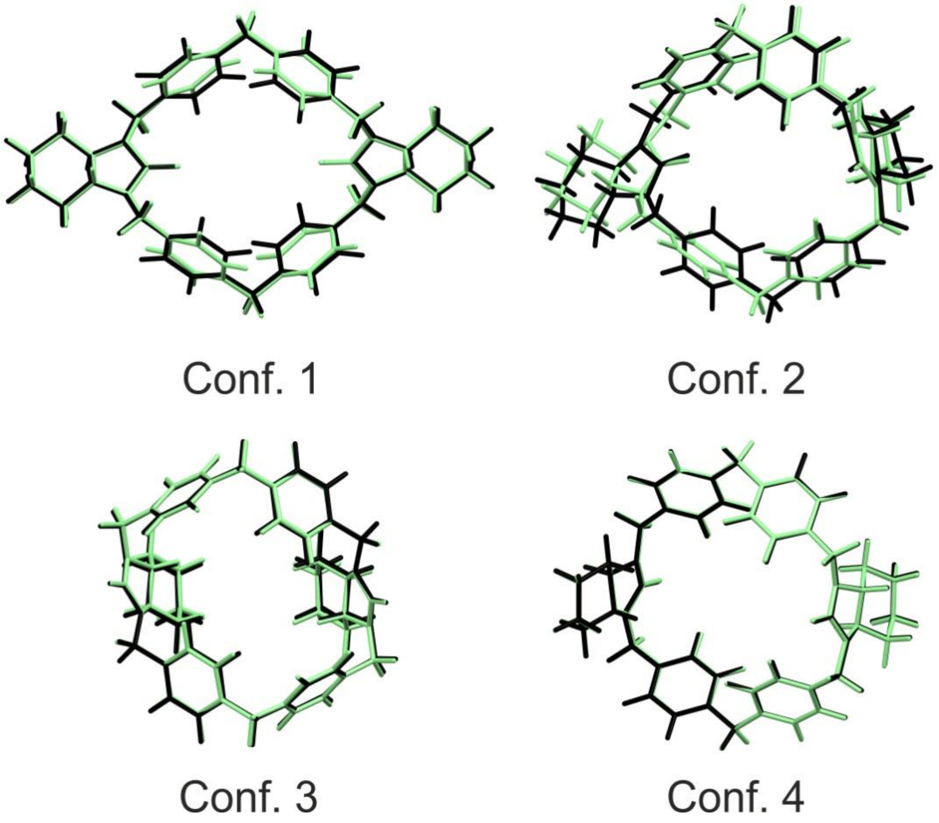

Figure S42. Overlaid of the structures of individual low-energy conformers of **9a** calculated at the DFT/6-311G(d,p) level of theory: *in vacuo* (black color) and with the use of acetonitrile solvent model (green color).

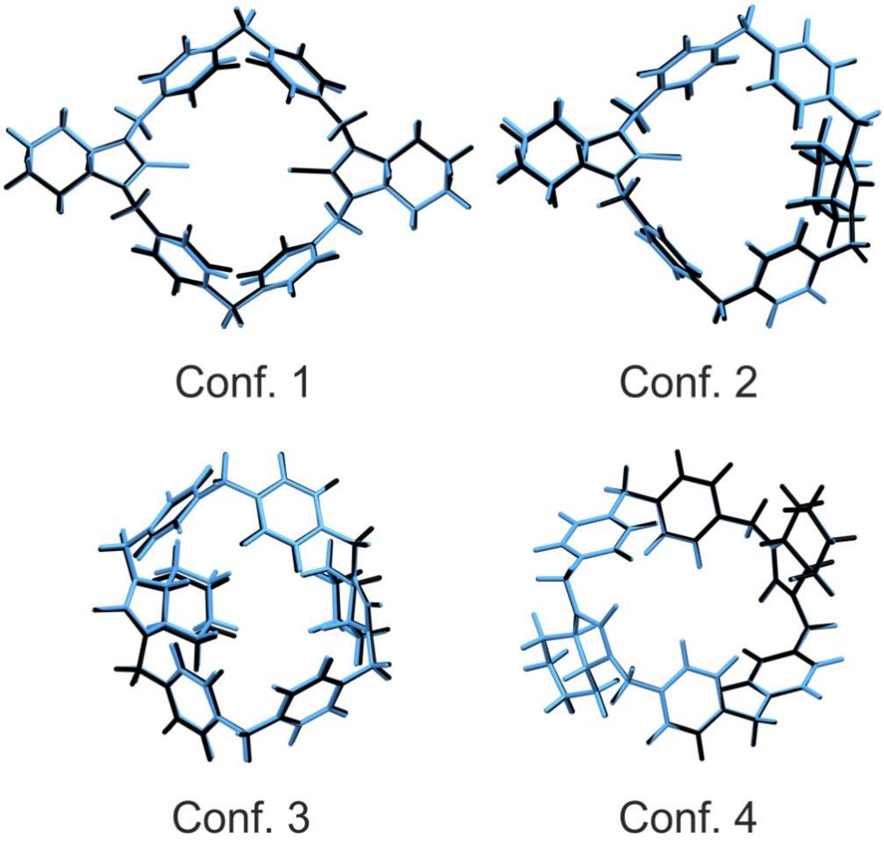

Figure S43. Overlaid of the structures of individual low-energy conformers of **9b** calculated at the DFT/6-311G(d,p) level of theory: *in vacuo* (black color) and with the use of dichloromethane solvent model (blue color).

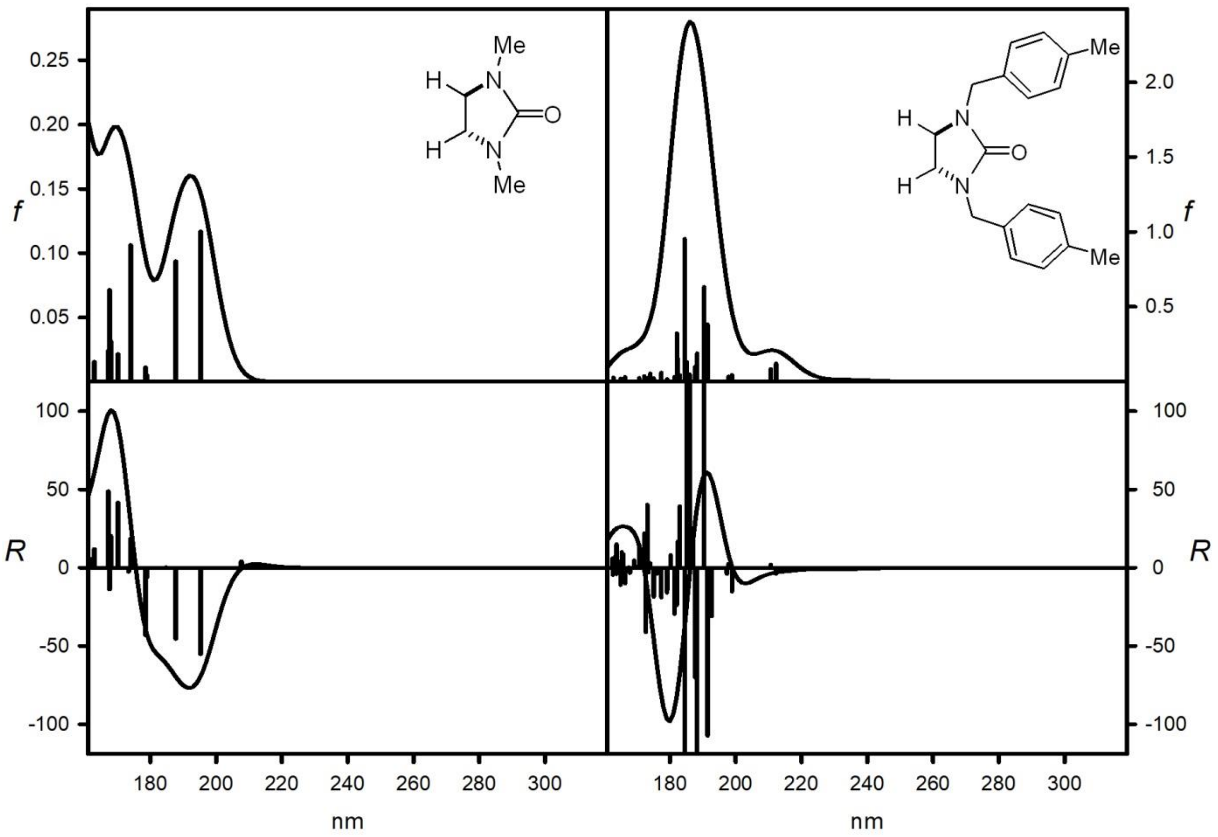

Figure S44. Calculated at the TD-CAM-B3LYP/6-311++G(d,p) level UV (upper panel) and ECD (lower panel) spectra of model compounds **13a** (left) and **13b** (right). Wavelengths were not corrected. Vertical bars represent oscillator or rotator strengths, respectively.

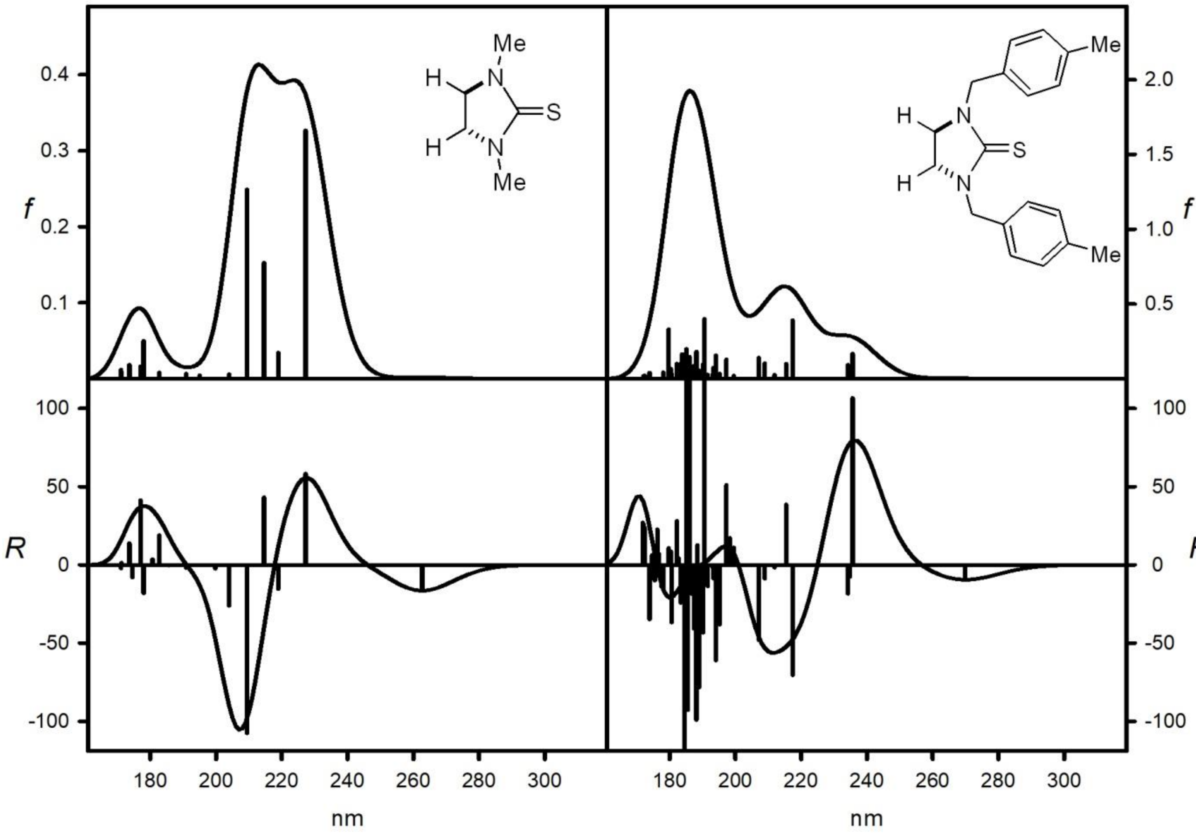

Figure S45. Calculated at the TD-CAM-B3LYP/6-311++G(d,p) level UV (upper panel) and ECD (lower panel) spectra of model compounds **14a** (left) and **14b** (right). Wavelengths were not corrected. Vertical bars represent oscillator or rotator strengths, respectively.

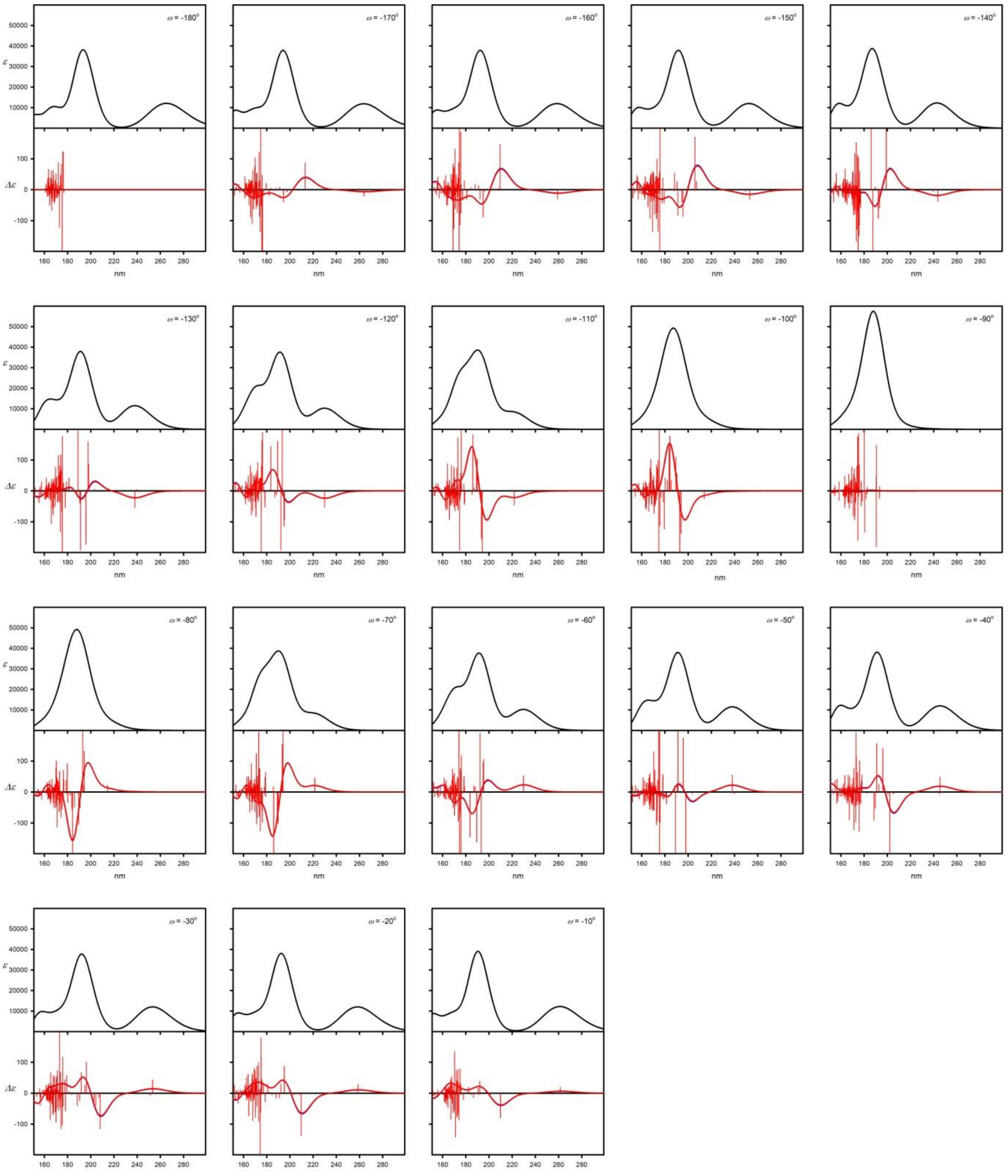

ω = -180°
ω = -170°
ω = -160°
ω = -150°
ω = -140°
ω = -130°
ω = -120°
ω = -110°
ω = -100°
ω = -90°
ω = -80°
ω = -70°
ω = -60°
ω = -50°
ω = -40°
ω = -30°
ω = -20°
ω = -10°
ε
10000
20000
30000
40000
50000
$\Delta\varepsilon$
100
0
-100
160
180
200
220
240
260
280
nm

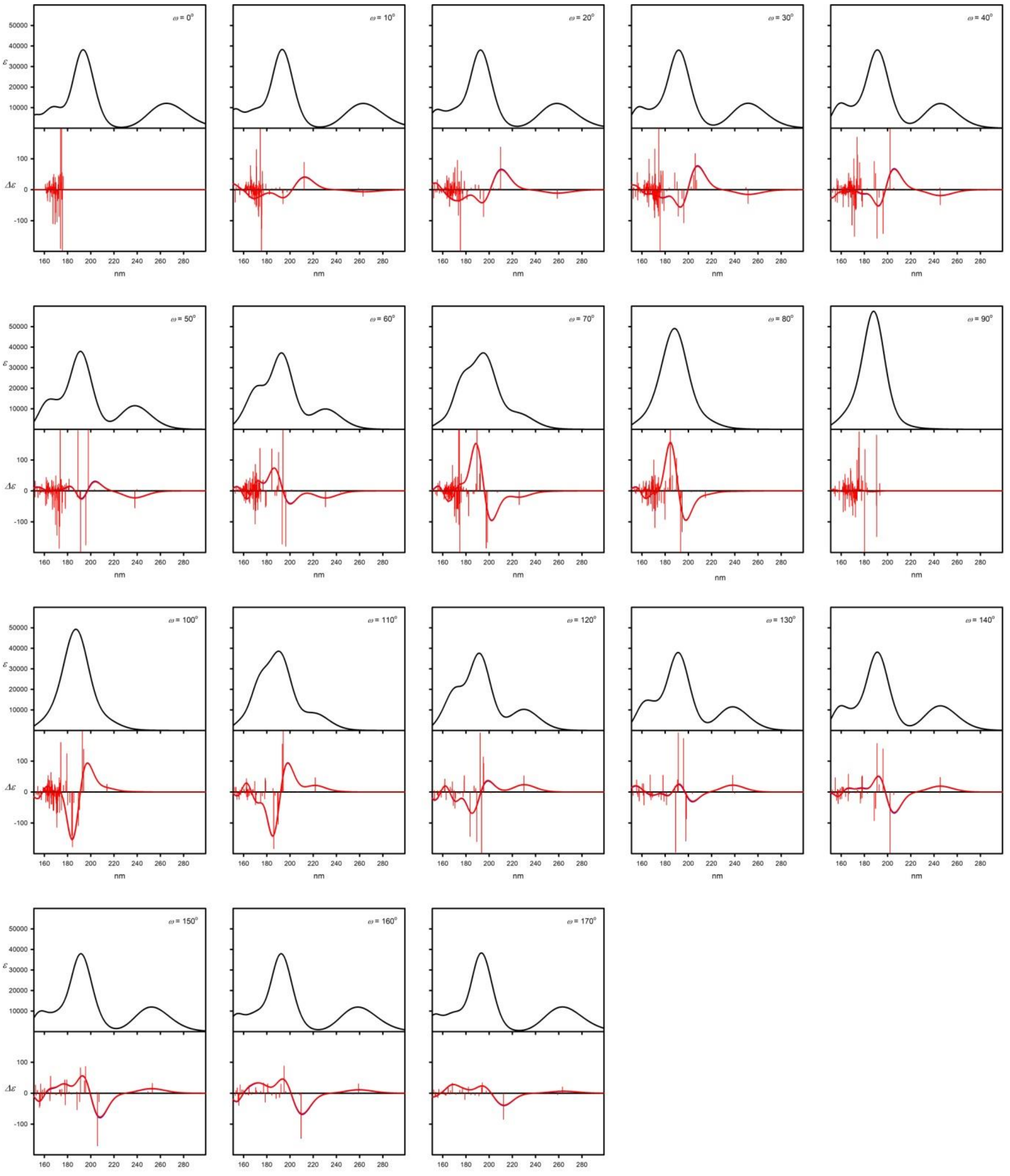

Figure S46. Calculated at the TD-CAM-B3LYP/6-311++G(d,p) level UV (upper panels) and ECD (lower panels) spectra of individual conformers of model compound **15**. Wavelengths were not corrected. Vertical bars represent oscillator or rotator strengths, respectively. The values at top right corner of each UV spectrum indicated the biphenyl moiety twist angle.

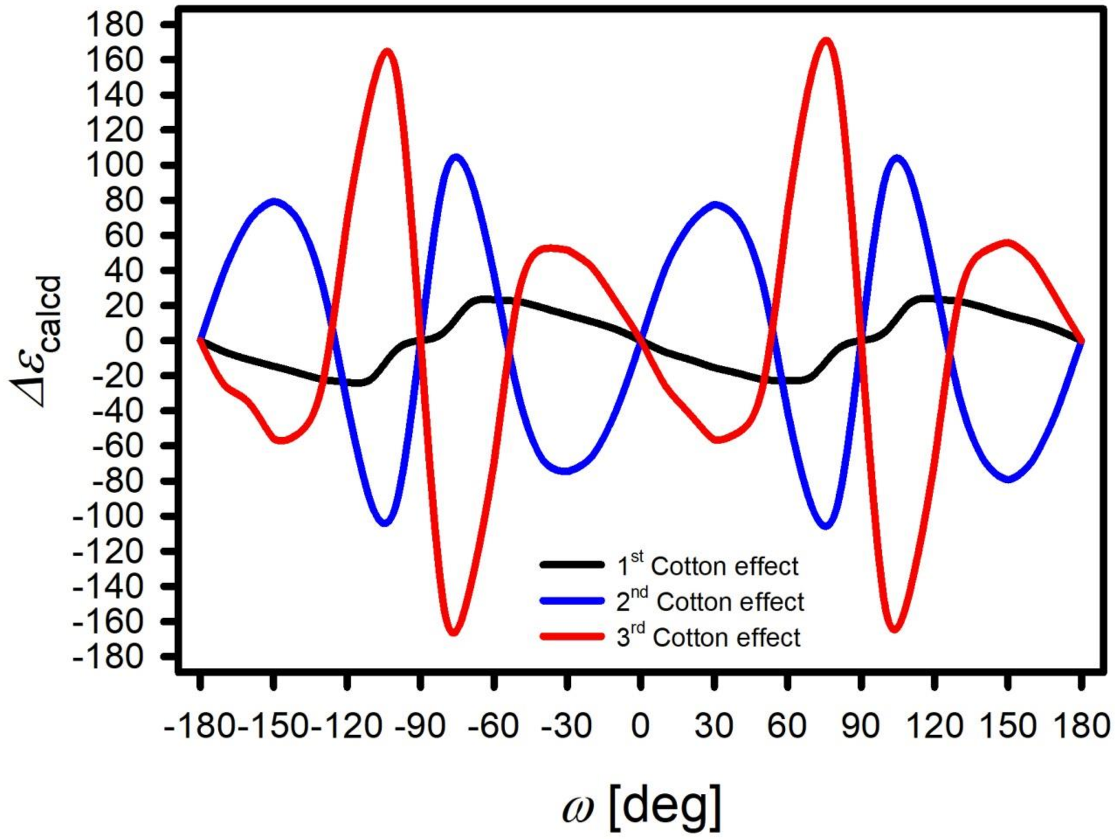

Figure S47. Values of the first three low-energy Cotton effects of model compound **15**, as a function of torsion angle ω, calculated at the TD-CAM-B3LYP/6-311++G(d,p) level of theory.

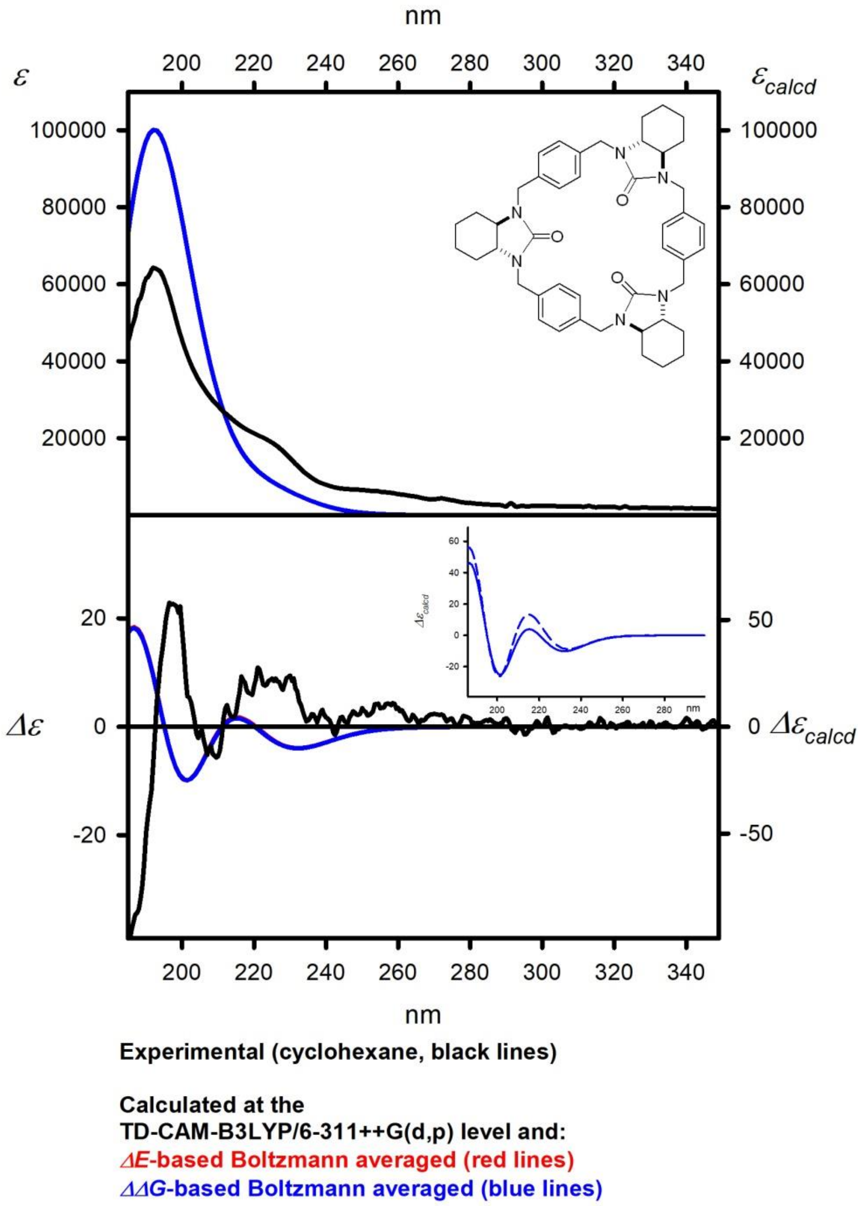

Figure S48. UV (upper panel) and ECD (lower panel) spectra of **1a** measured in cyclohexane (solid black lines) and calculated at the TD-CAM-B3LYP/6-311++G(d,p) level. The calculated ECD spectra were Boltzmann-averaged based on *Δ*E (red lines) and *ΔΔ*G values (blue lines). Wavelengths were corrected to match the experimental UV maxima. The insert shows the comparison between the ECD spectra calculated for the lowest energy conformer of a given compound (dashed blue lines) and the *ΔΔ*G-based and Boltzmann averaged (solid blue lines).

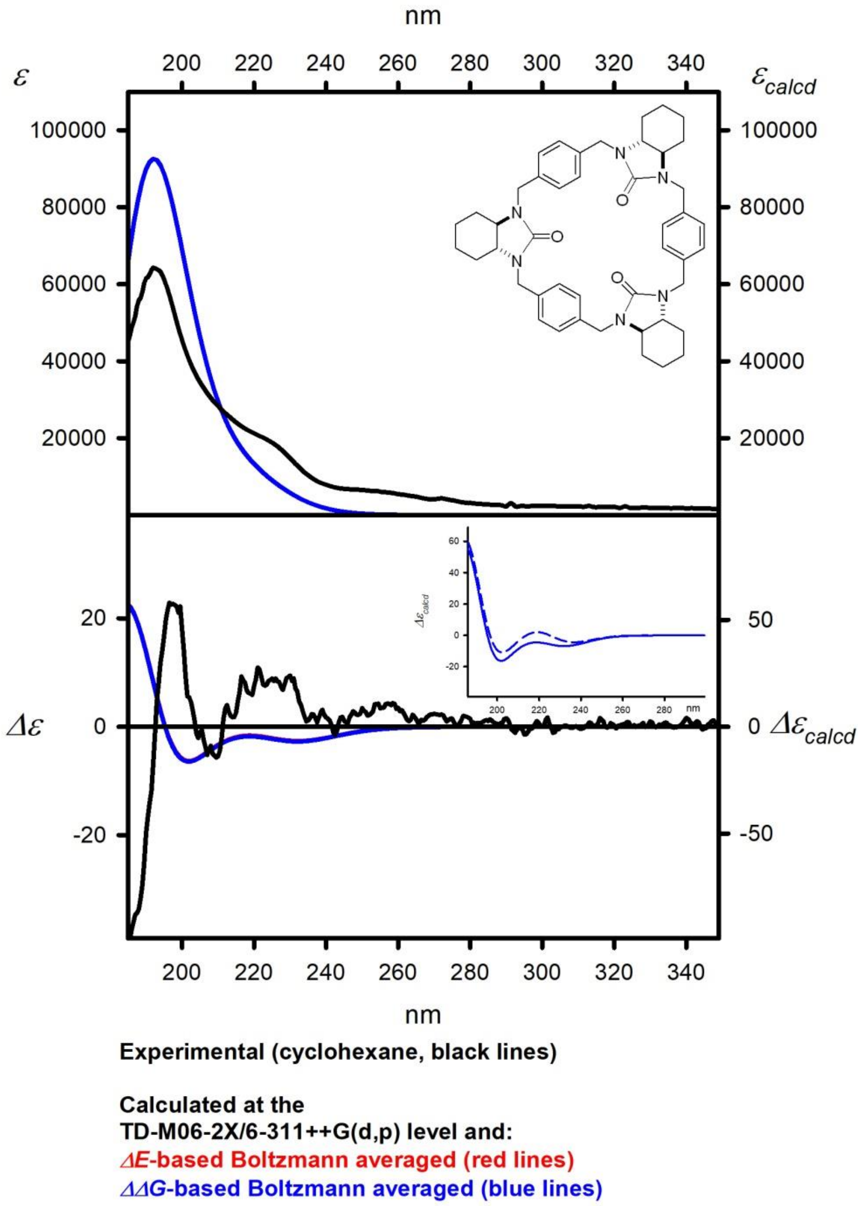

Figure S49. UV (upper panel) and ECD (lower panel) spectra of **1a** measured in cyclohexane (solid black lines) and calculated at the TD-M06-2X/6-311++G(d,p) level. The calculated ECD spectra were Boltzmann-averaged based on *Δ*E (red lines) and *ΔΔ*G values (blue lines). Wavelengths were corrected to match the experimental UV maxima. The insert shows the comparison between the ECD spectra calculated for the lowest energy conformer of a given compound (dashed blue lines) and the *ΔΔ*G-based and Boltzmann averaged (solid blue lines).

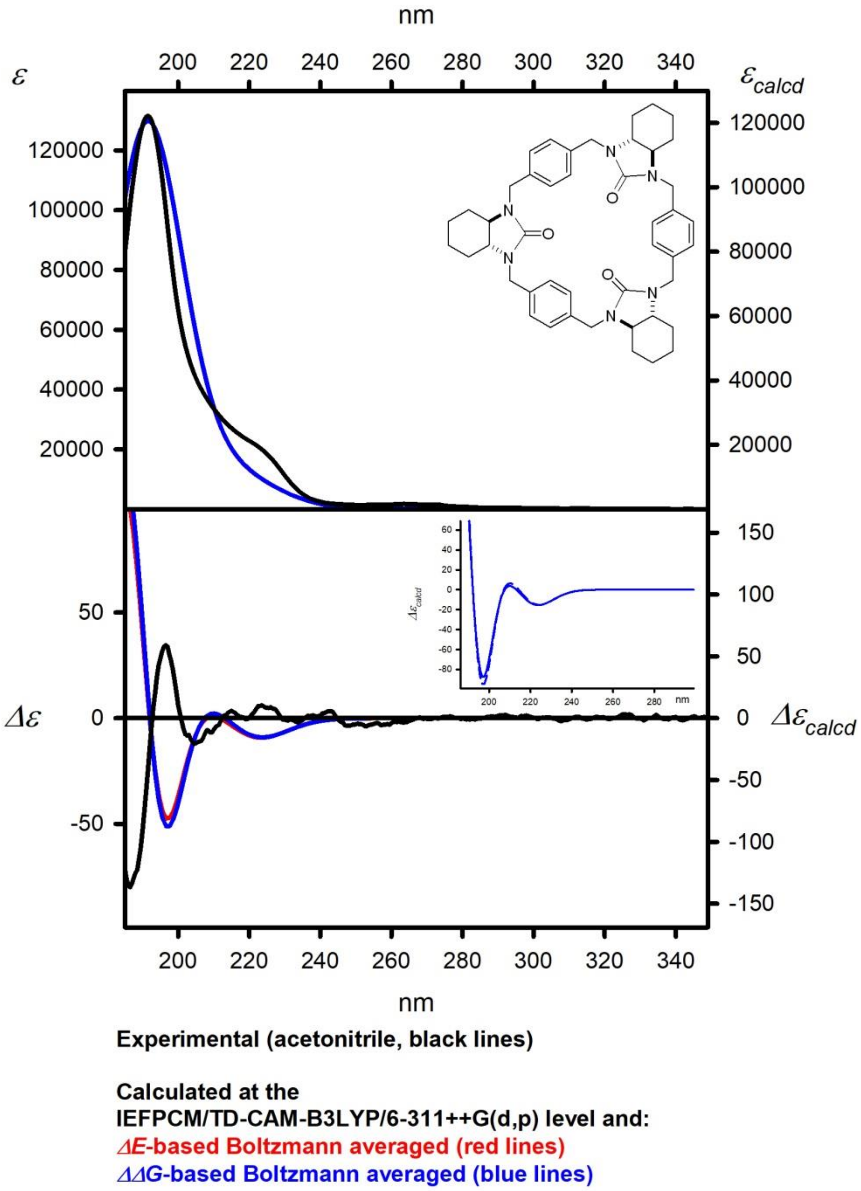

Figure S50. UV (upper panel) and ECD (lower panel) spectra of **1a** measured in acetonitrile (solid black lines) and calculated at the IEFPCM/TD-CAM-B3LYP/6-311++G(d,p) level. The calculated ECD spectra were Boltzmann-averaged based on *Δ*E (red lines) and *ΔΔ*G values (blue lines). Wavelengths were corrected to match the experimental UV maxima. The insert shows the comparison between the ECD spectra calculated for the lowest energy conformer of a given compound (dashed blue lines) and the *ΔΔ*G-based and Boltzmann averaged (solid blue lines).

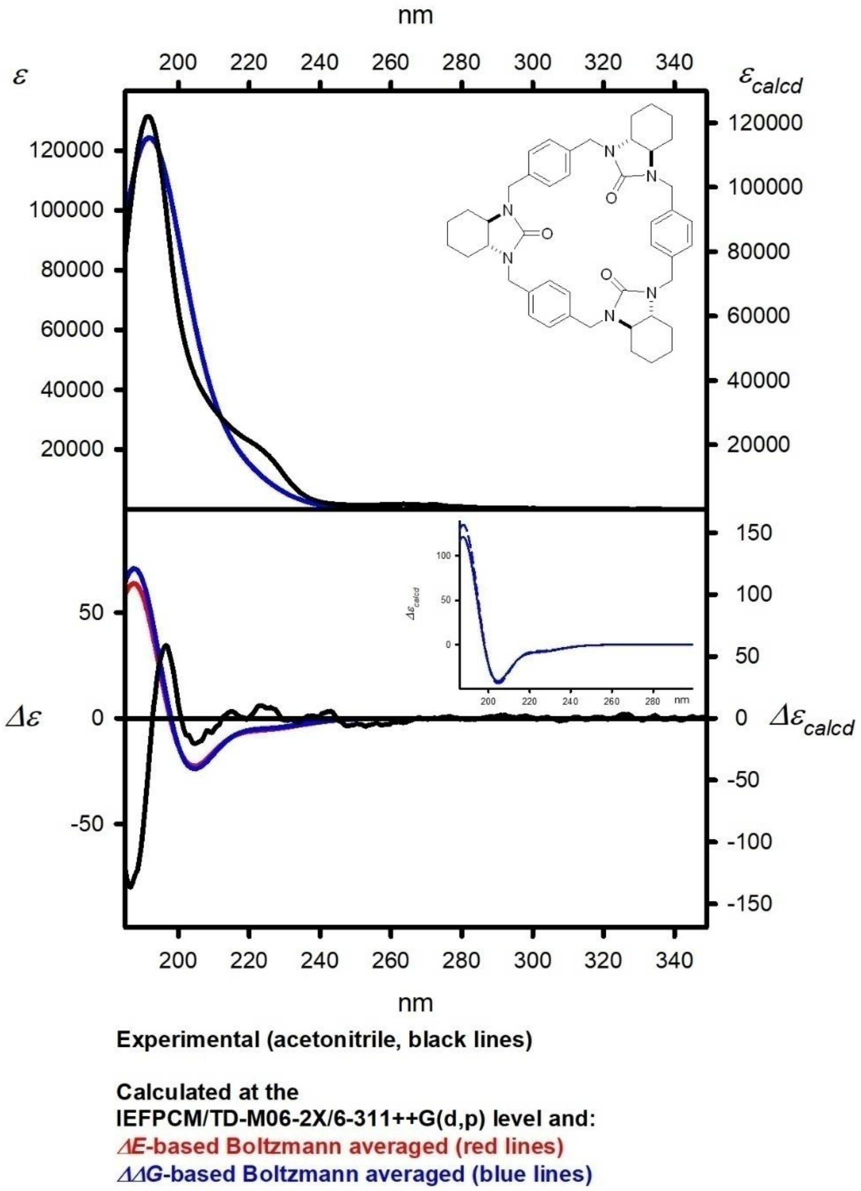

Figure S51. UV (upper panel) and ECD (lower panel) spectra of **1a** measured in acetonitrile (solid black lines) and calculated at the IEFPCM/TD-M06-2X/6-311++G(d,p) level. The calculated ECD spectra were Boltzmann-averaged based on *Δ*E (red lines) and *ΔΔ*G values (blue lines). Wavelengths were corrected to match the experimental UV maxima. The insert shows the comparison between the ECD spectra calculated for the lowest energy conformer of a given compound (dashed blue lines) and the *ΔΔ*G-based and Boltzmann averaged (solid blue lines).

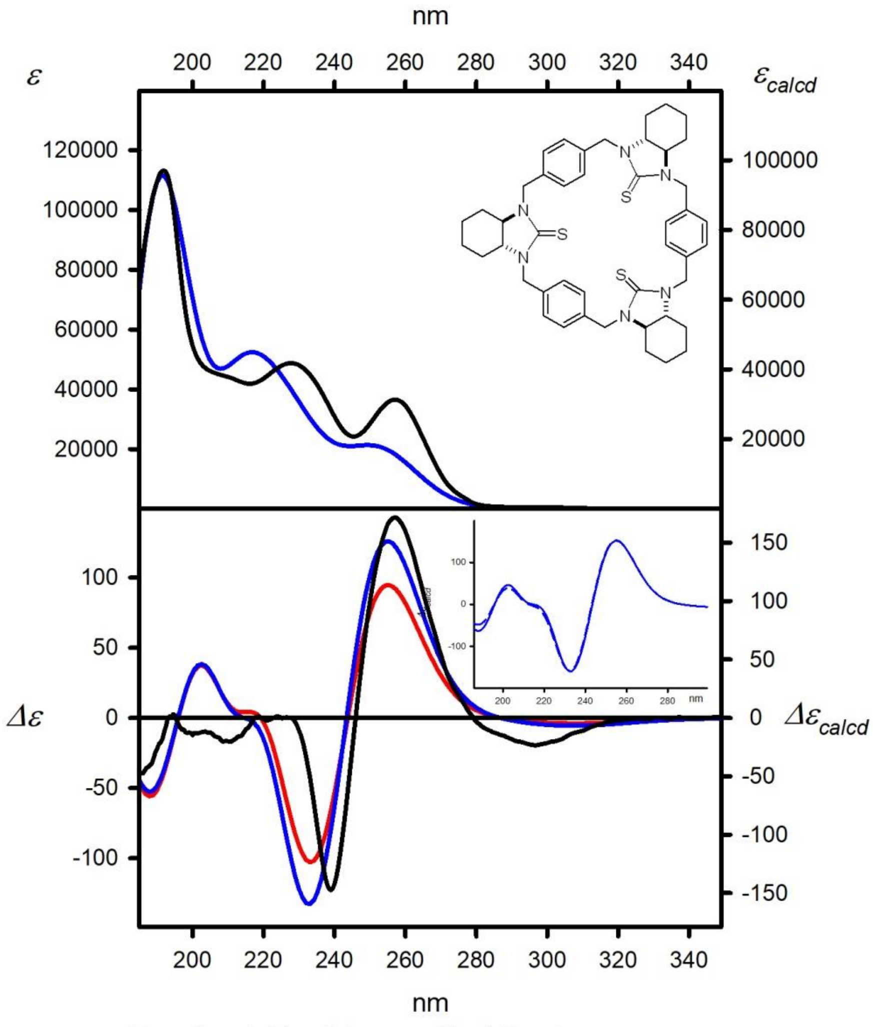

Figure S52. UV (upper panel) and ECD (lower panel) spectra of **1b** measured in cyclohexane (solid black lines) and calculated at the TD-CAM-B3LYP/6-311++G(d,p) level. The calculated ECD spectra were Boltzmann-averaged based on *Δ*E (red lines) and *ΔΔ*G values (blue lines). Wavelengths were corrected to match the experimental UV maxima. The insert shows the comparison between the ECD spectra calculated for the lowest energy conformer of a given compound (dashed blue lines) and the *ΔΔ*G-based and Boltzmann averaged (solid blue lines).

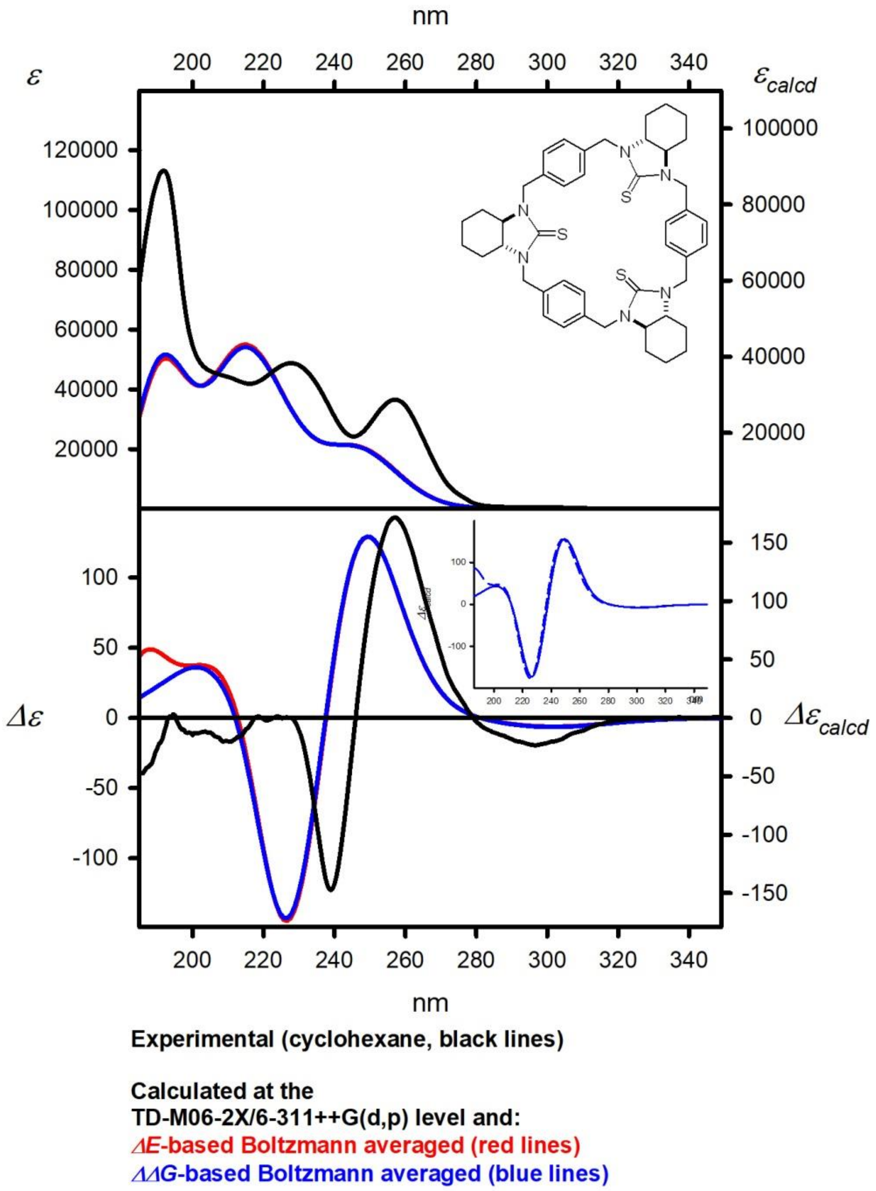

Figure S53. UV (upper panel) and ECD (lower panel) spectra of **1b** measured in cyclohexane (solid black lines) and calculated at the TD-M06-2X/6-311++G(d,p) level. The calculated ECD spectra were Boltzmann-averaged based on *Δ*E (red lines) and *ΔΔ*G values (blue lines). Wavelengths were corrected to match the experimental UV maxima. The insert shows the comparison between the ECD spectra calculated for the lowest energy conformer of a given compound (dashed blue lines) and the *ΔΔ*G-based and Boltzmann averaged (solid blue lines).

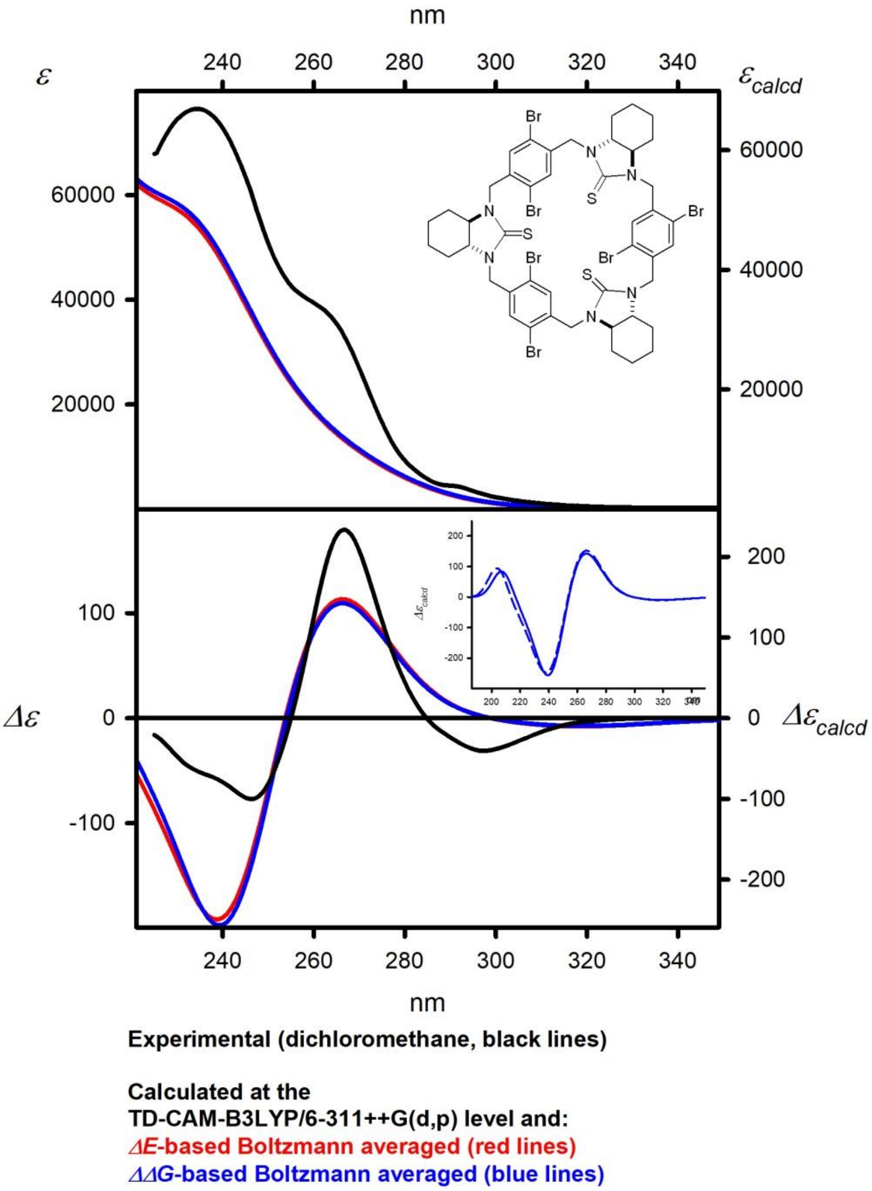

Figure S54. UV (upper panel) and ECD (lower panel) spectra of **3** measured in dichloromethane (solid black lines) and calculated at the TD-CAM-B3LYP/6-311++G(d,p) level. The calculated ECD spectra were Boltzmann-averaged based on *Δ*E (red lines) and *ΔΔ*G values (blue lines). Wavelengths were corrected to match the experimental UV maxima. The insert shows the comparison between the ECD spectra calculated for the lowest energy conformer of a given compound (dashed blue lines) and the *ΔΔ*G-based and Boltzmann averaged (solid blue lines).

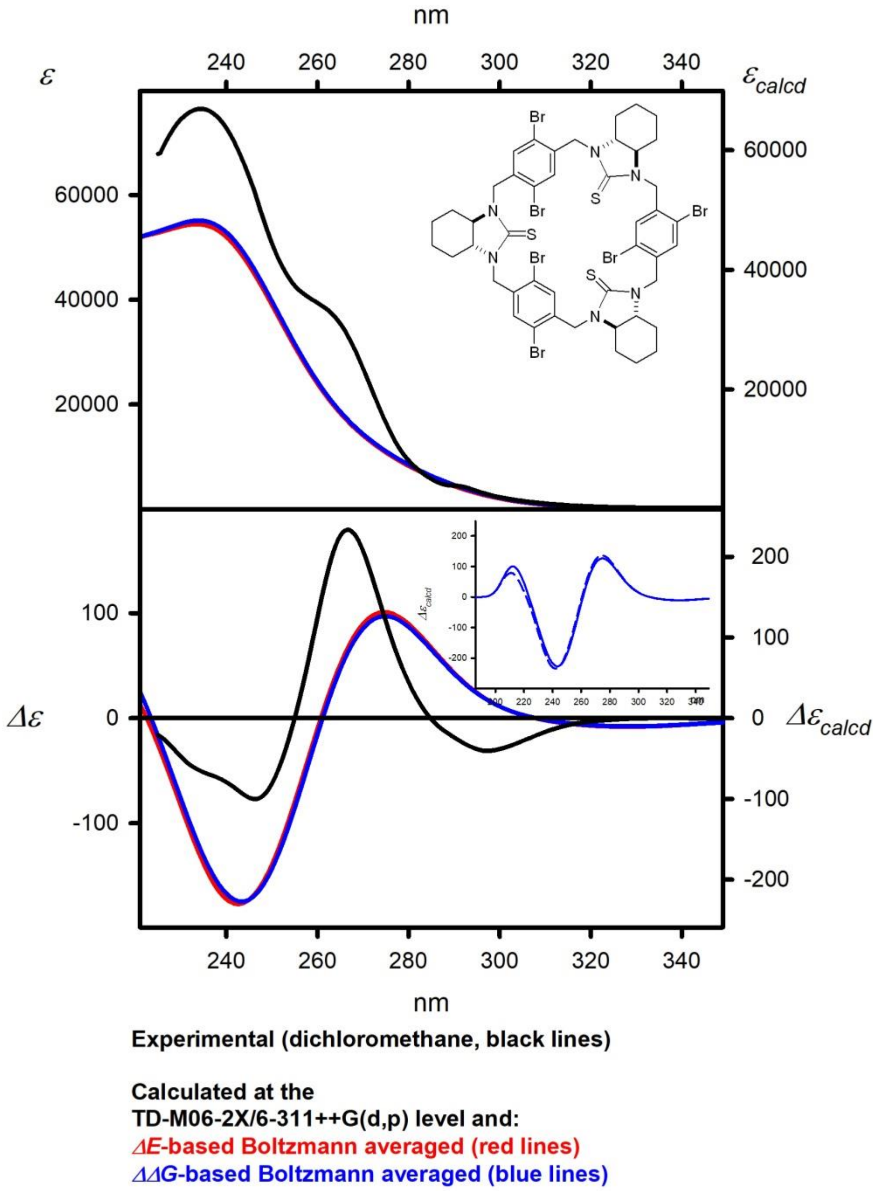

Figure S55. UV (upper panel) and ECD (lower panel) spectra of **3** measured in dichloromethane (solid black lines) and calculated at the TD-M06-2X/6-311++G(d,p) level. The calculated ECD spectra were Boltzmann-averaged based on *Δ*E (red lines) and *ΔΔ*G values (blue lines). Wavelengths were corrected to match the experimental UV maxima. The insert shows the comparison between the ECD spectra calculated for the lowest energy conformer of a given compound (dashed blue lines) and the *ΔΔ*G-based and Boltzmann averaged (solid blue lines).

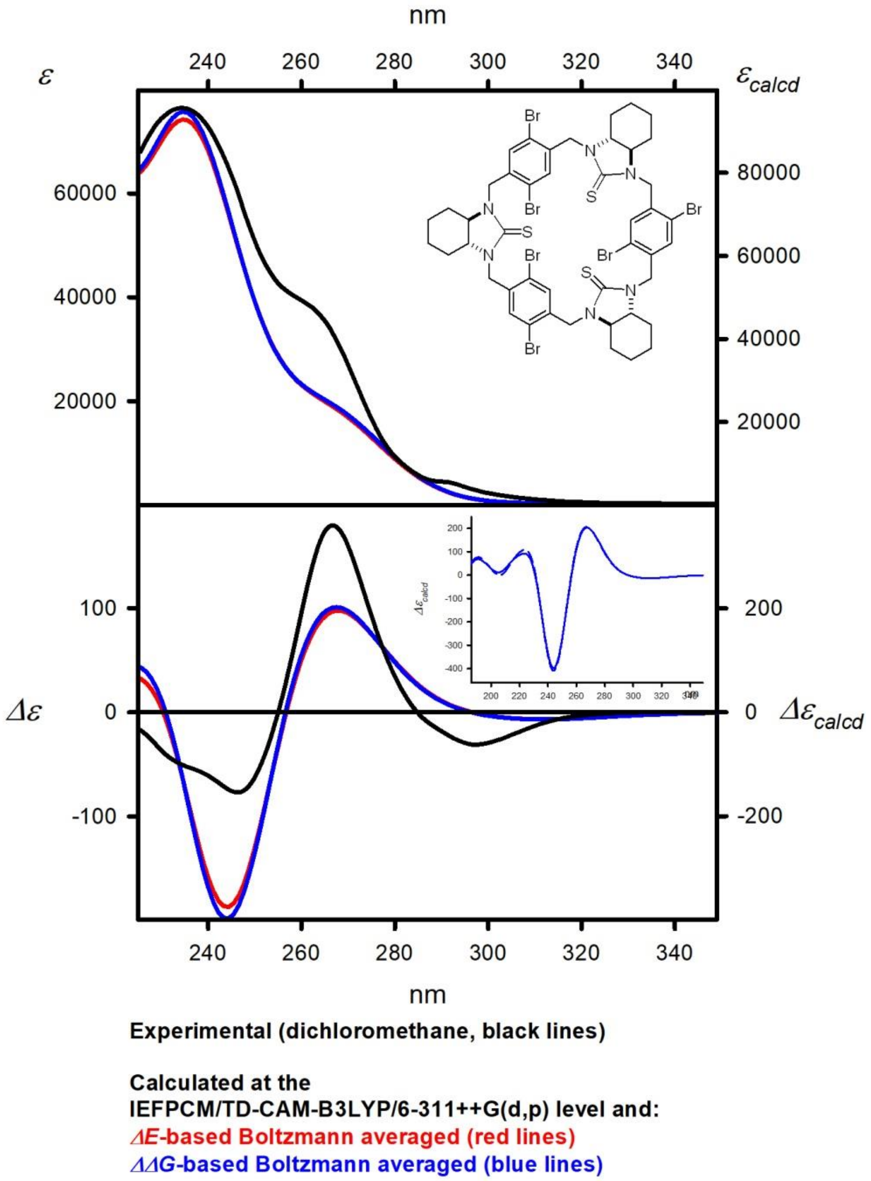

Figure S56. UV (upper panel) and ECD (lower panel) spectra of **3** measured in dichloromethane (solid black lines) and calculated at the IEFPCM/TD-CAM-B3LYP/6-311++G(d,p) level. The calculated ECD spectra were Boltzmann-averaged based on ΔE (red lines) and $\Delta\Delta G$ values (blue lines). Wavelengths were corrected to match the experimental UV maxima. The insert shows the comparison between the ECD spectra calculated for the lowest energy conformer of a given compound (dashed blue lines) and the $\Delta\Delta G$-based and Boltzmann averaged (solid blue lines).

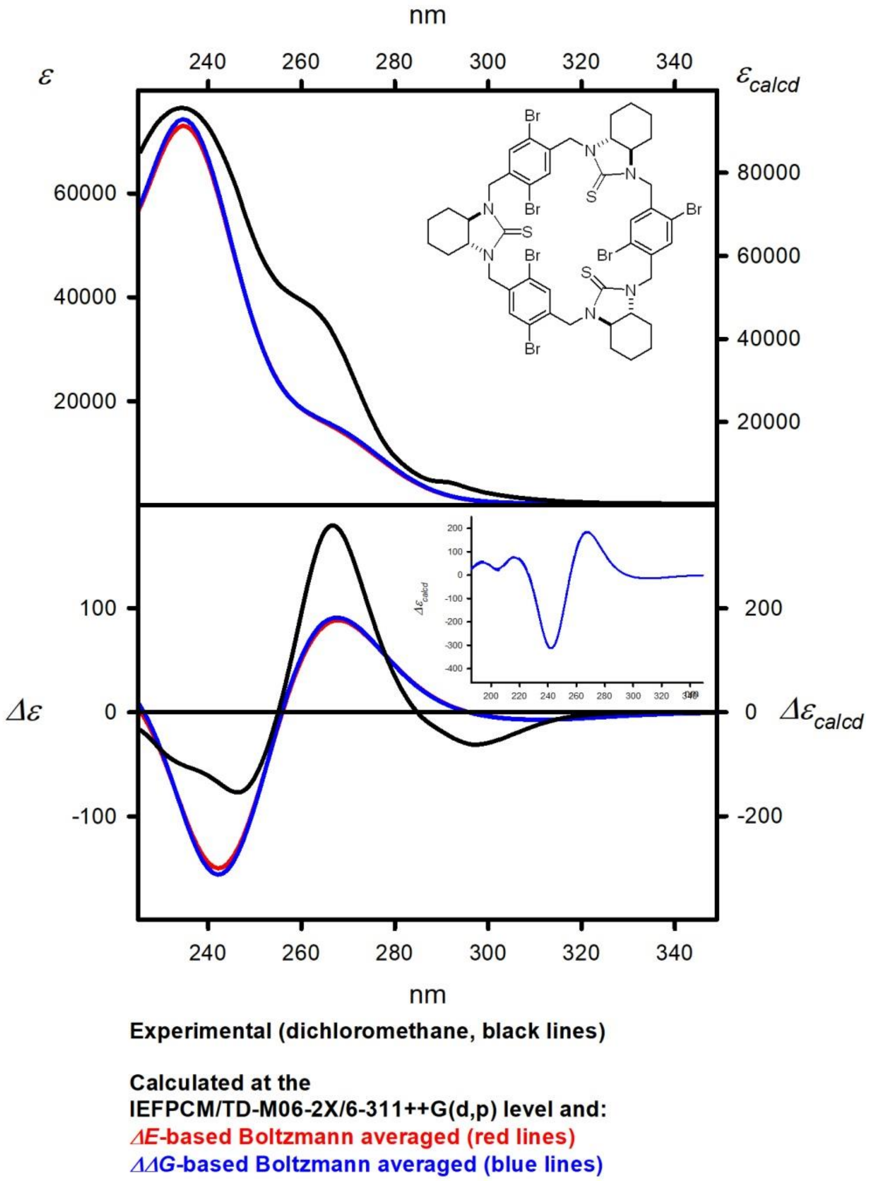

Figure S57. UV (upper panel) and ECD (lower panel) spectra of **3** measured in dichloromethane (solid black lines) and calculated at the IEFPCM/TD-M06-2X/6-311++G(d,p) level. The calculated ECD spectra were Boltzmann-averaged based on *Δ*E (red lines) and *ΔΔ*G values (blue lines). Wavelengths were corrected to match the experimental UV maxima. The insert shows the comparison between the ECD spectra calculated for the lowest energy conformer of a given compound (dashed blue lines) and the *ΔΔ*G-based and Boltzmann averaged (solid blue lines).

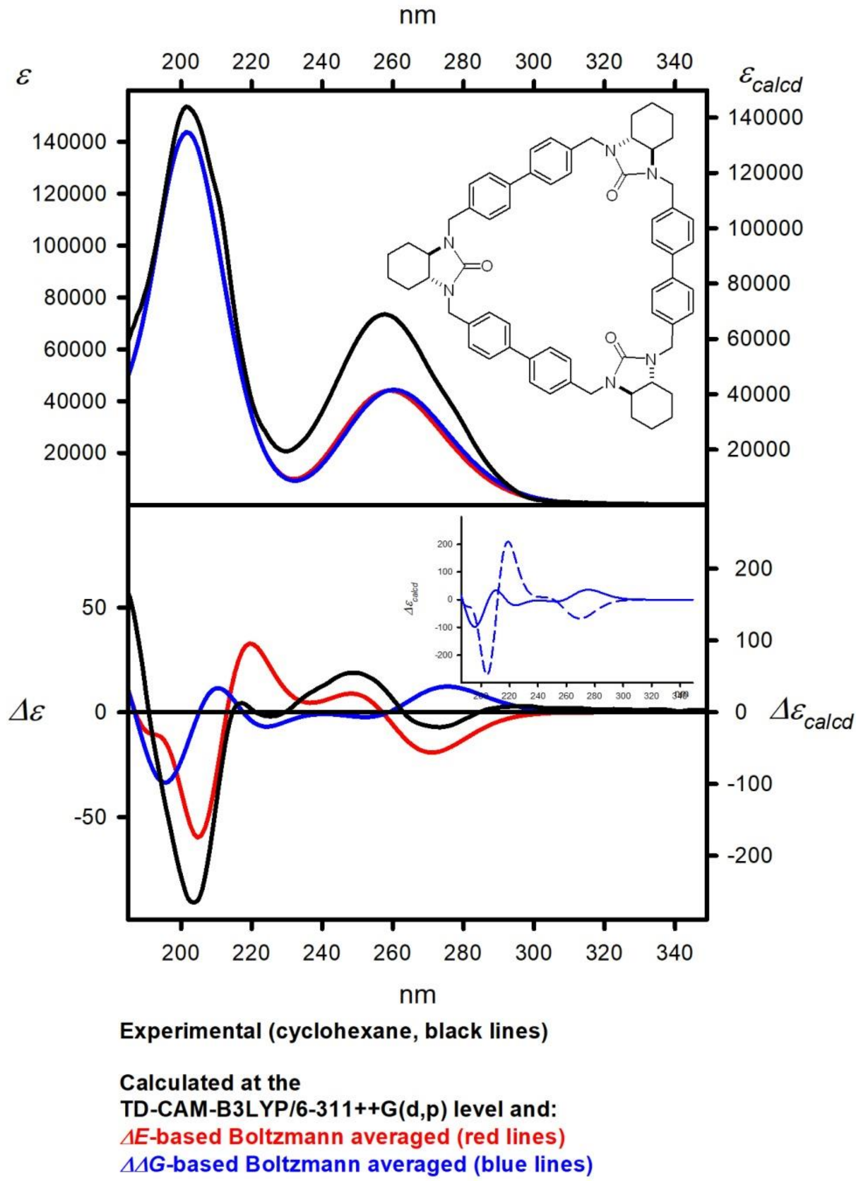

Figure S58. UV (upper panel) and ECD (lower panel) spectra of **4** measured in cyclohexane (solid black lines) and calculated at the TD-CAM-B3LYP/6-311++G(d,p) level. The calculated ECD spectra were Boltzmann-averaged based on *Δ*E (red lines) and *ΔΔ*G values (blue lines). Wavelengths were corrected to match the experimental UV maxima. The insert shows the comparison between the ECD spectra calculated for the lowest energy conformer of a given compound (dashed blue lines) and the *ΔΔ*G-based and Boltzmann averaged (solid blue lines).

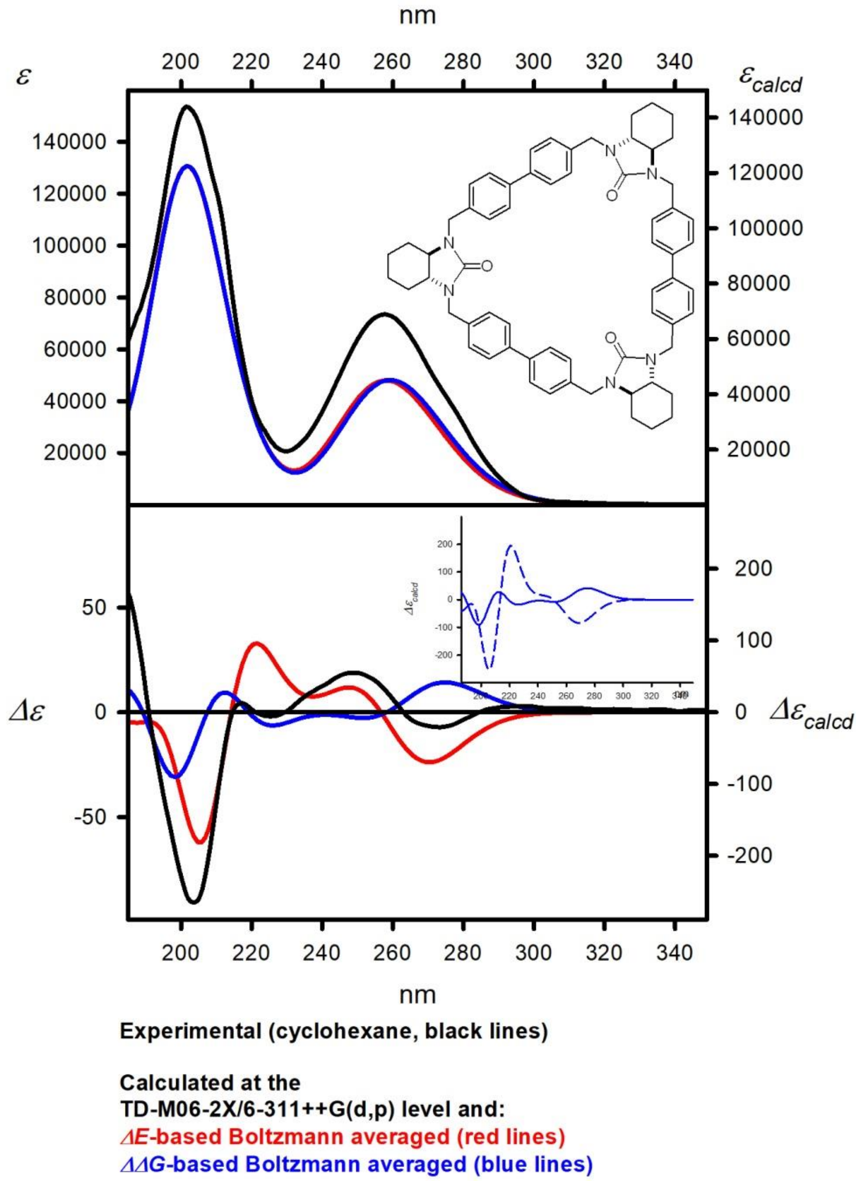

Figure S59. UV (upper panel) and ECD (lower panel) spectra of **4** measured in cyclohexane (solid black lines) and calculated at the TD-M06-2X/6-311++G(d,p) level. The calculated ECD spectra were Boltzmann-averaged based on *Δ*E (red lines) and *ΔΔ*G values (blue lines). Wavelengths were corrected to match the experimental UV maxima. The insert shows the comparison between the ECD spectra calculated for the lowest energy conformer of a given compound (dashed blue lines) and the *ΔΔ*G-based and Boltzmann averaged (solid blue lines).

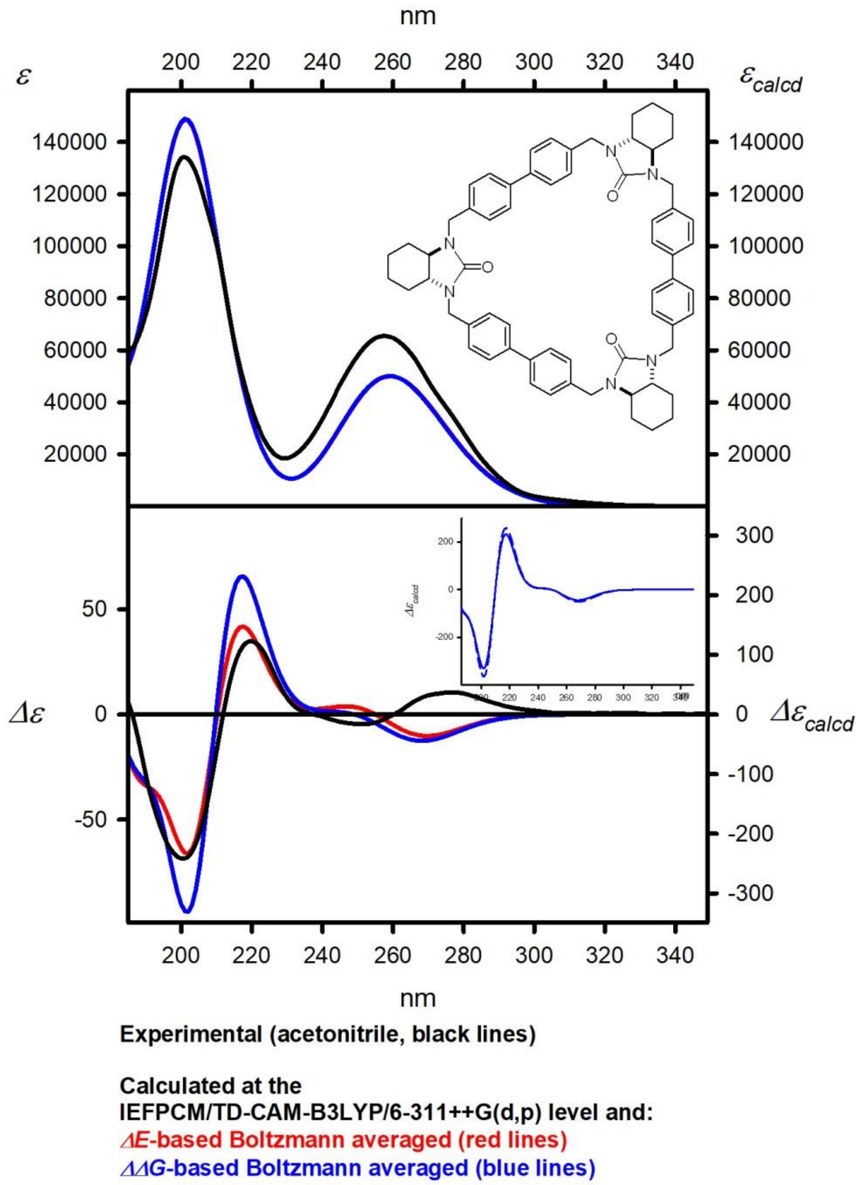

Figure S60. UV (upper panel) and ECD (lower panel) spectra of **4** measured in acetonitrile (solid black lines) and calculated at the IEFPCM/TD-CAM-B3LYP/6-311++G(d,p) level. The calculated ECD spectra were Boltzmann-averaged based on *Δ*E (red lines) and *ΔΔ*G values (blue lines). Wavelengths were corrected to match the experimental UV maxima. The insert shows the comparison between the ECD spectra calculated for the lowest energy conformer of a given compound (dashed blue lines) and the *ΔΔ*G-based and Boltzmann averaged (solid blue lines).

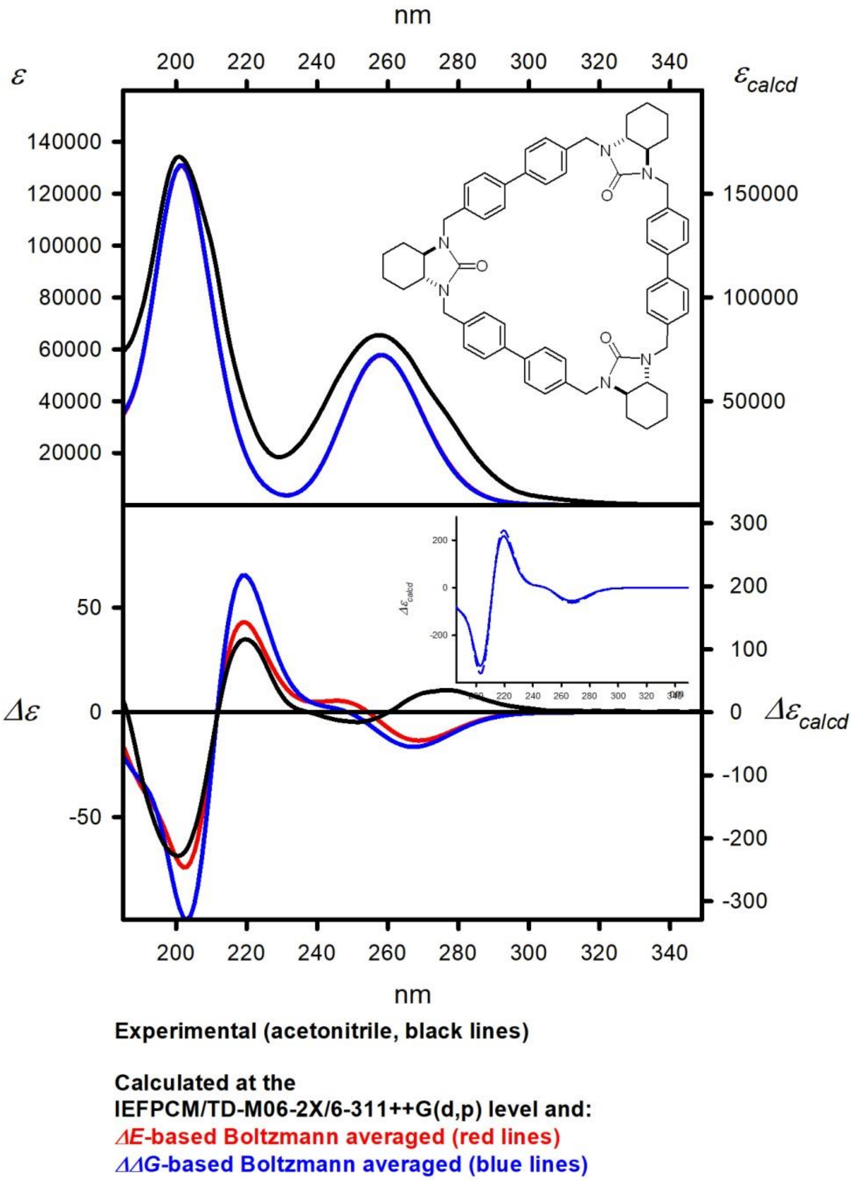

Figure S61. UV (upper panel) and ECD (lower panel) spectra of **4** measured in acetonitrile (solid black lines) and calculated at the IEFPCM/TD-M06-2X/6-311++G(d,p) level. The calculated ECD spectra were Boltzmann-averaged based on *Δ*E (red lines) and *ΔΔ*G values (blue lines). Wavelengths were corrected to match the experimental UV maxima. The insert shows the comparison between the ECD spectra calculated for the lowest energy conformer of a given compound (dashed blue lines) and the *ΔΔ*G-based and Boltzmann averaged (solid blue lines).

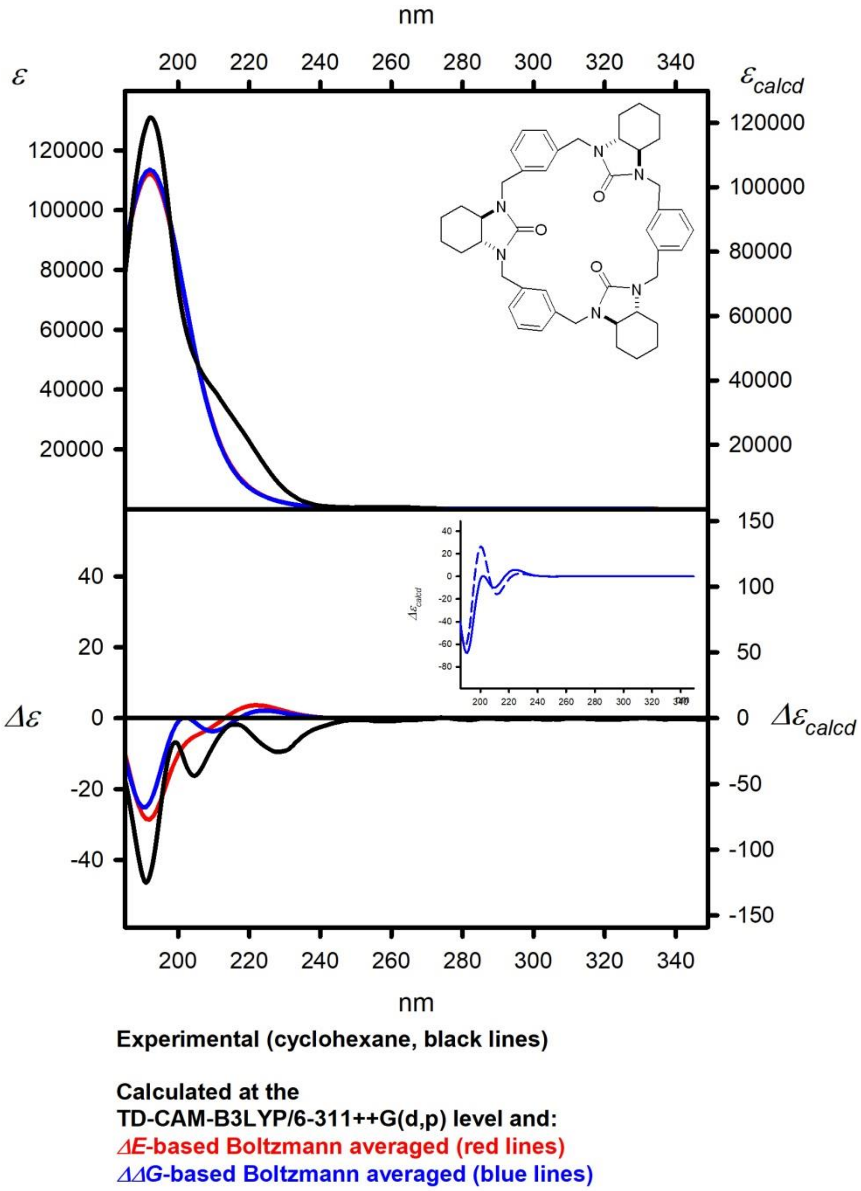

Figure S62. UV (upper panel) and ECD (lower panel) spectra of **5a** measured in cyclohexane (solid black lines) and calculated at the TD-CAM-B3LYP/6-311++G(d,p) level. The calculated ECD spectra were Boltzmann-averaged based on *Δ*E (red lines) and *ΔΔ*G values (blue lines). Wavelengths were corrected to match the experimental UV maxima. The insert shows the comparison between the ECD spectra calculated for the lowest energy conformer of a given compound (dashed blue lines) and the *ΔΔ*G-based and Boltzmann averaged (solid blue lines).

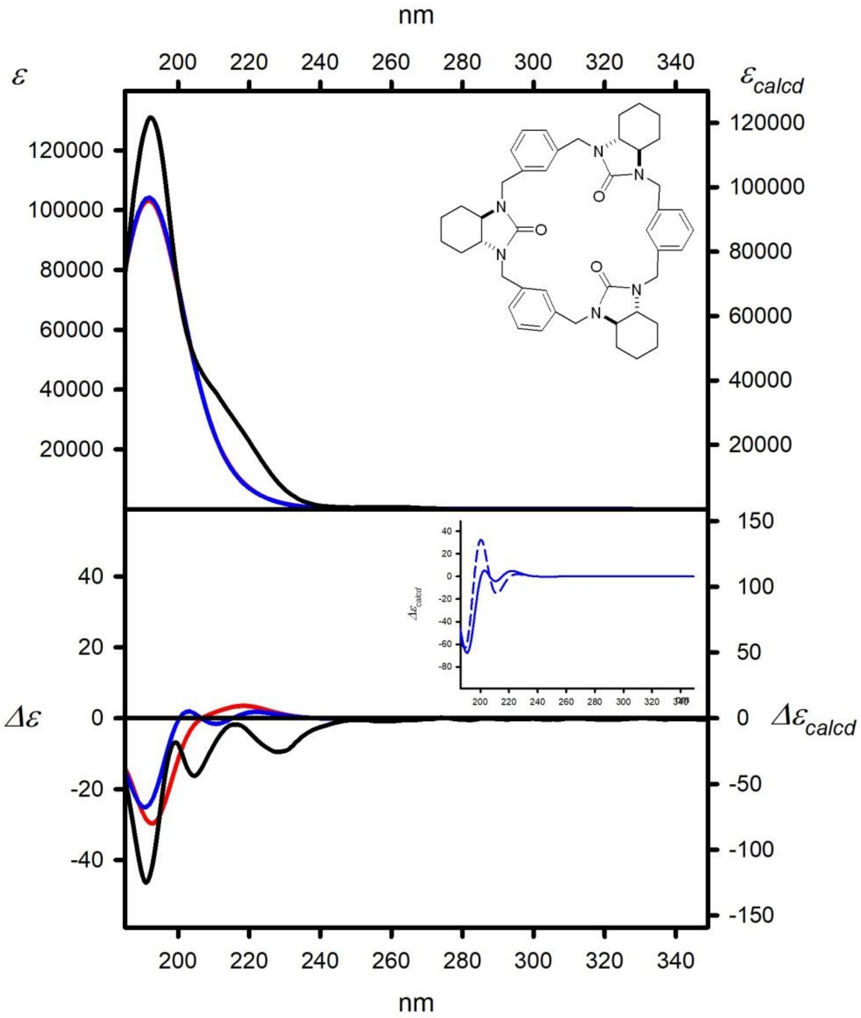

Experimental (cyclohexane, black lines)

Calculated at the
TD-M06-2X/6-311++G(d,p) level and:
***ΔE*-based Boltzmann averaged (red lines)**
***ΔΔG*-based Boltzmann averaged (blue lines)**

Figure S63. UV (upper panel) and ECD (lower panel) spectra of **5a** measured in cyclohexane (solid black lines) and calculated at the TD-M06-2X/6-311++G(d,p) level. The calculated ECD spectra were Boltzmann-averaged based on *Δ*E (red lines) and *ΔΔ*G values (blue lines). Wavelengths were corrected to match the experimental UV maxima. The insert shows the comparison between the ECD spectra calculated for the lowest energy conformer of a given compound (dashed blue lines) and the *ΔΔ*G-based and Boltzmann averaged (solid blue lines).

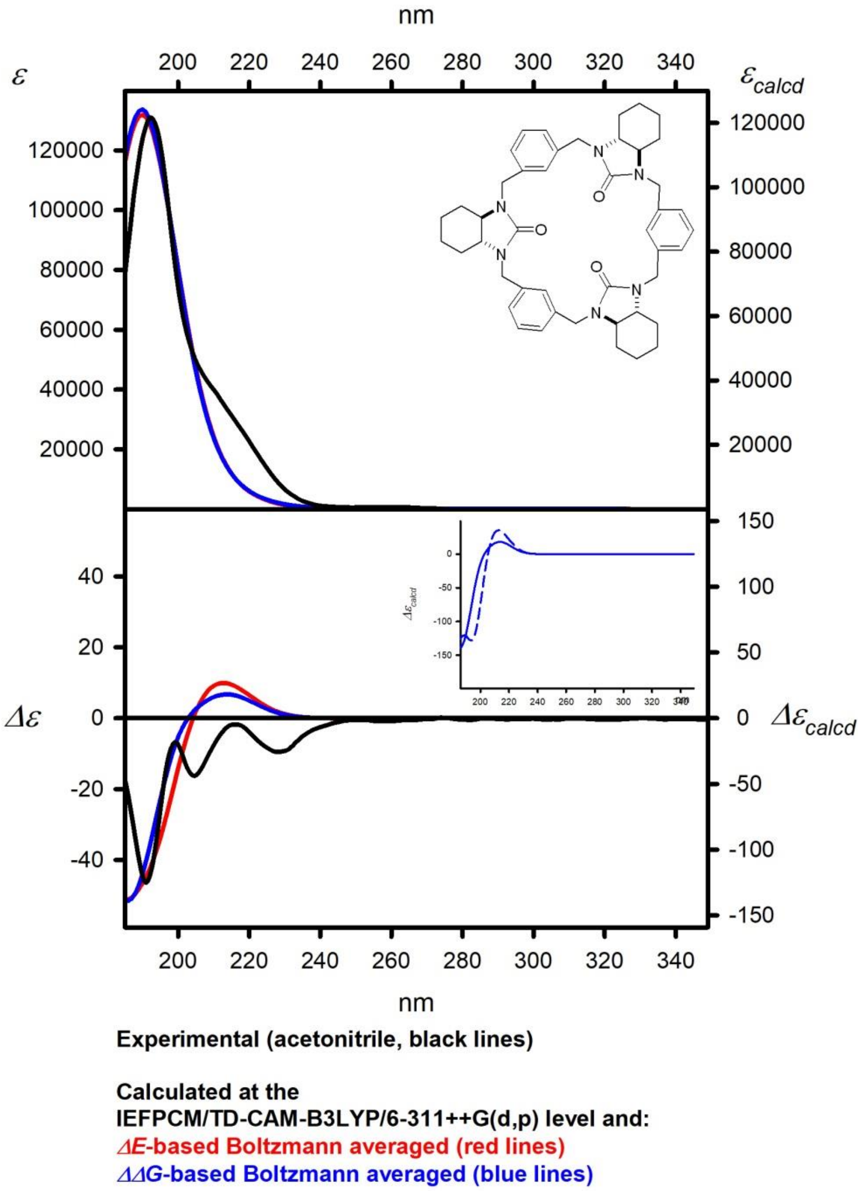

Figure S64. UV (upper panel) and ECD (lower panel) spectra of **5a** measured in acetonitrile (solid black lines) and calculated at the IEFPCM/TD-CAM-B3LYP/6-311++G(d,p) level. The calculated ECD spectra were Boltzmann-averaged based on *Δ*E (red lines) and *ΔΔ*G values (blue lines). Wavelengths were corrected to match the experimental UV maxima. The insert shows the comparison between the ECD spectra calculated for the lowest energy conformer of a given compound (dashed blue lines) and the *ΔΔ*G-based and Boltzmann averaged (solid blue lines).

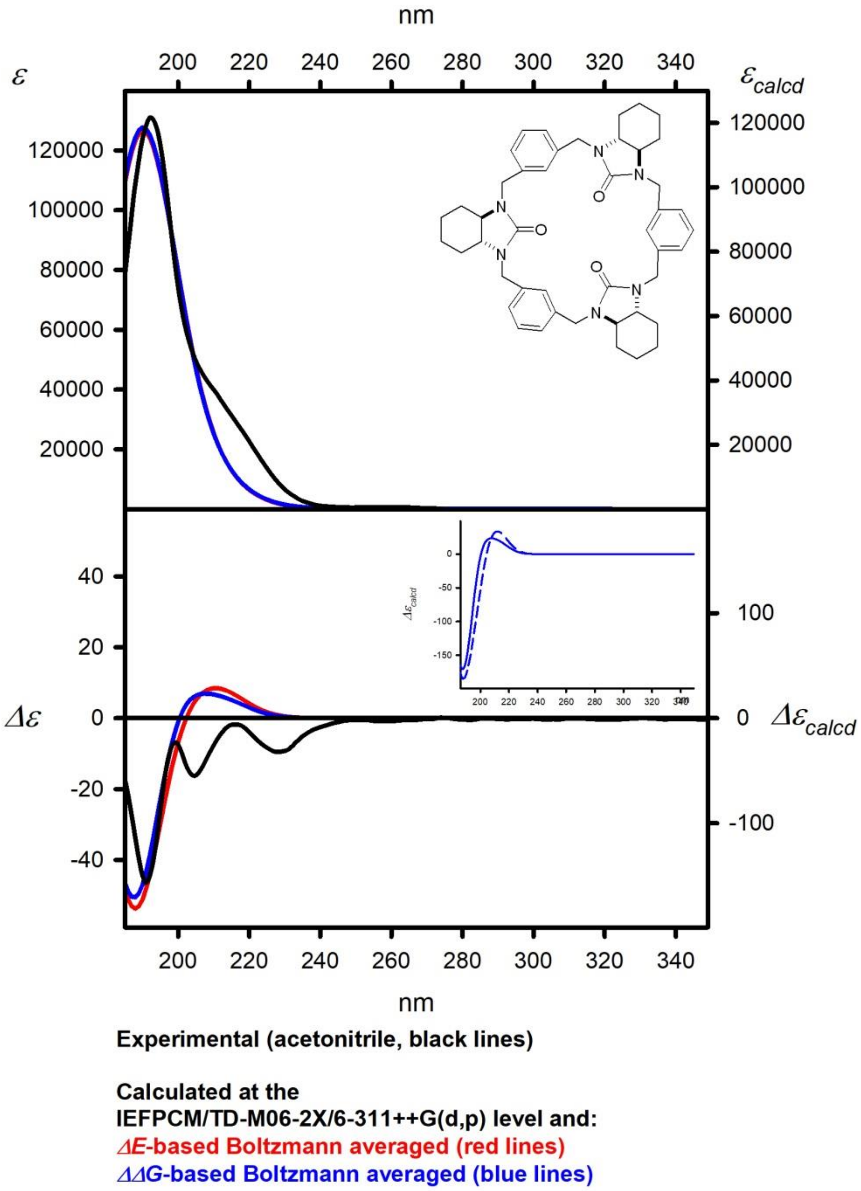

Figure S65. UV (upper panel) and ECD (lower panel) spectra of **5a** measured in acetonitrile (solid black lines) and calculated at the IEFPCM/TD-M06-2X/6-311++G(d,p) level. The calculated ECD spectra were Boltzmann-averaged based on *Δ*E (red lines) and *ΔΔ*G values (blue lines). Wavelengths were corrected to match the experimental UV maxima. The insert shows the comparison between the ECD spectra calculated for the lowest energy conformer of a given compound (dashed blue lines) and the *ΔΔ*G-based and Boltzmann averaged (solid blue lines).

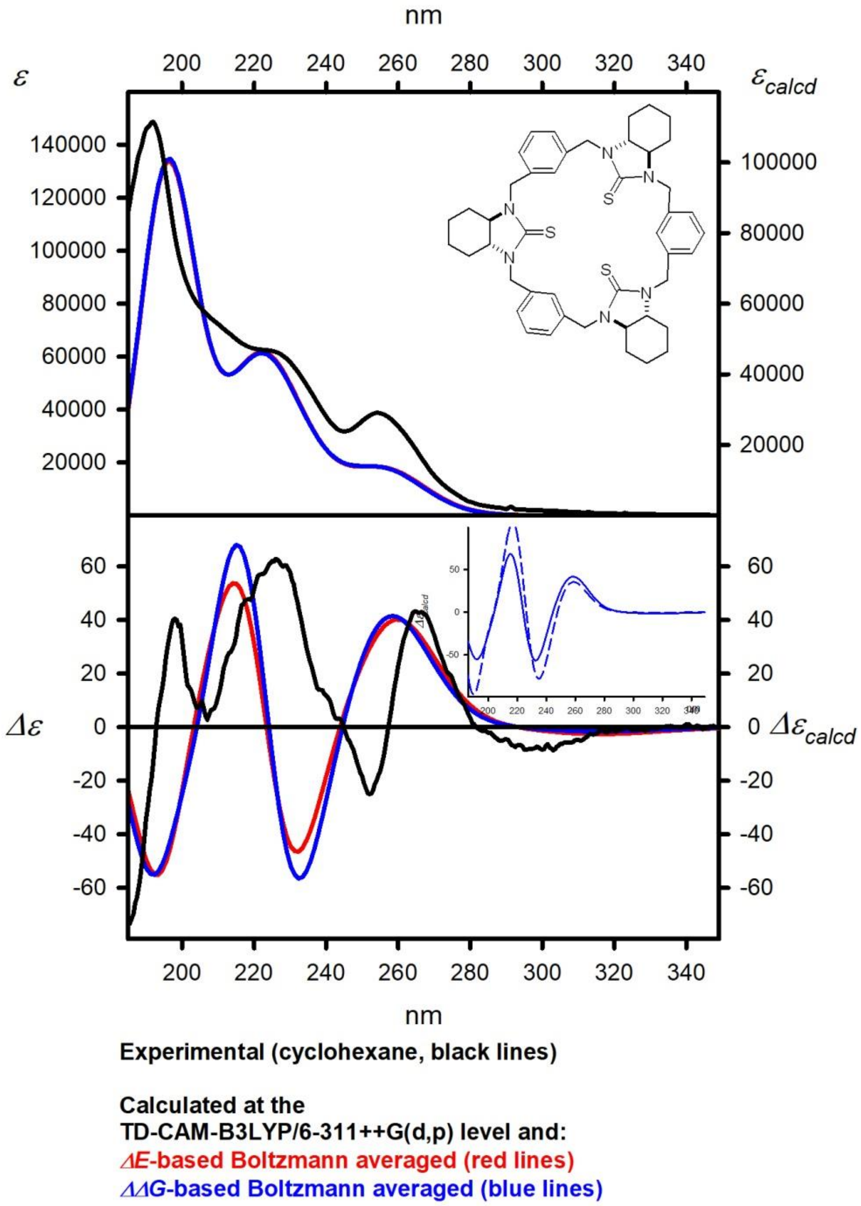

Figure S66. UV (upper panel) and ECD (lower panel) spectra of **5b** measured in cyclohexane (solid black lines) and calculated at the TD-CAM-B3LYP/6-311++G(d,p) level. The calculated ECD spectra were Boltzmann-averaged based on ΔE (red lines) and $\Delta\Delta$G values (blue lines). Wavelengths were corrected to match the experimental UV maxima. The insert shows the comparison between the ECD spectra calculated for the lowest energy conformer of a given compound (dashed blue lines) and the $\Delta\Delta$G-based and Boltzmann averaged (solid blue lines).

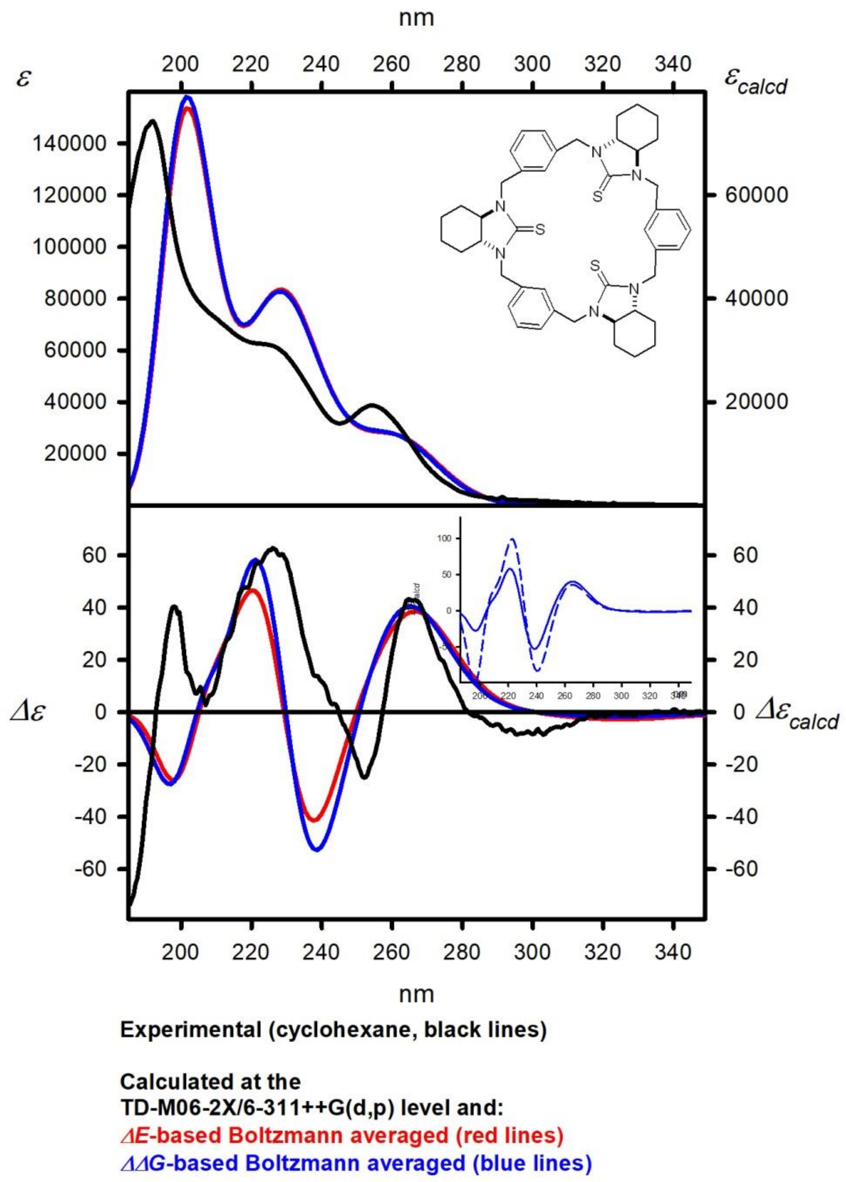

Figure S67. UV (upper panel) and ECD (lower panel) spectra of **5b** measured in cyclohexane (solid black lines) and calculated at the TD-M06-2X/6-311++G(d,p) level. The calculated ECD spectra were Boltzmann-averaged based on *Δ*E (red lines) and *ΔΔ*G values (blue lines). Wavelengths were corrected to match the experimental UV maxima. The insert shows the comparison between the ECD spectra calculated for the lowest energy conformer of a given compound (dashed blue lines) and the *ΔΔ*G-based and Boltzmann averaged (solid blue lines).

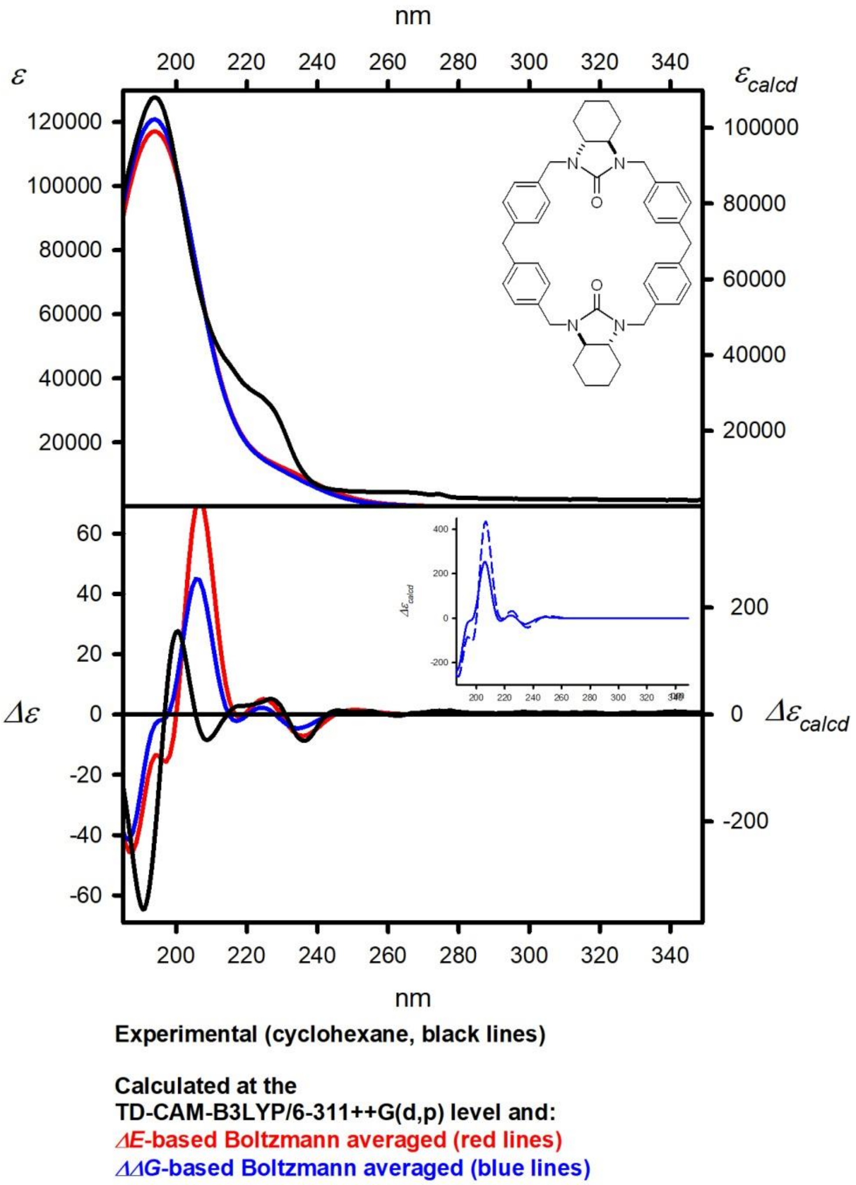

Figure S68. UV (upper panel) and ECD (lower panel) spectra of **9a** measured in cyclohexane (solid black lines) and calculated at the TD-CAM-B3LYP/6-311++G(d,p) level. The calculated ECD spectra were Boltzmann-averaged based on *Δ*E (red lines) and *ΔΔ*G values (blue lines). Wavelengths were corrected to match the experimental UV maxima. The insert shows the comparison between the ECD spectra calculated for the lowest energy conformer of a given compound (dashed blue lines) and the *ΔΔ*G-based and Boltzmann averaged (solid blue lines).

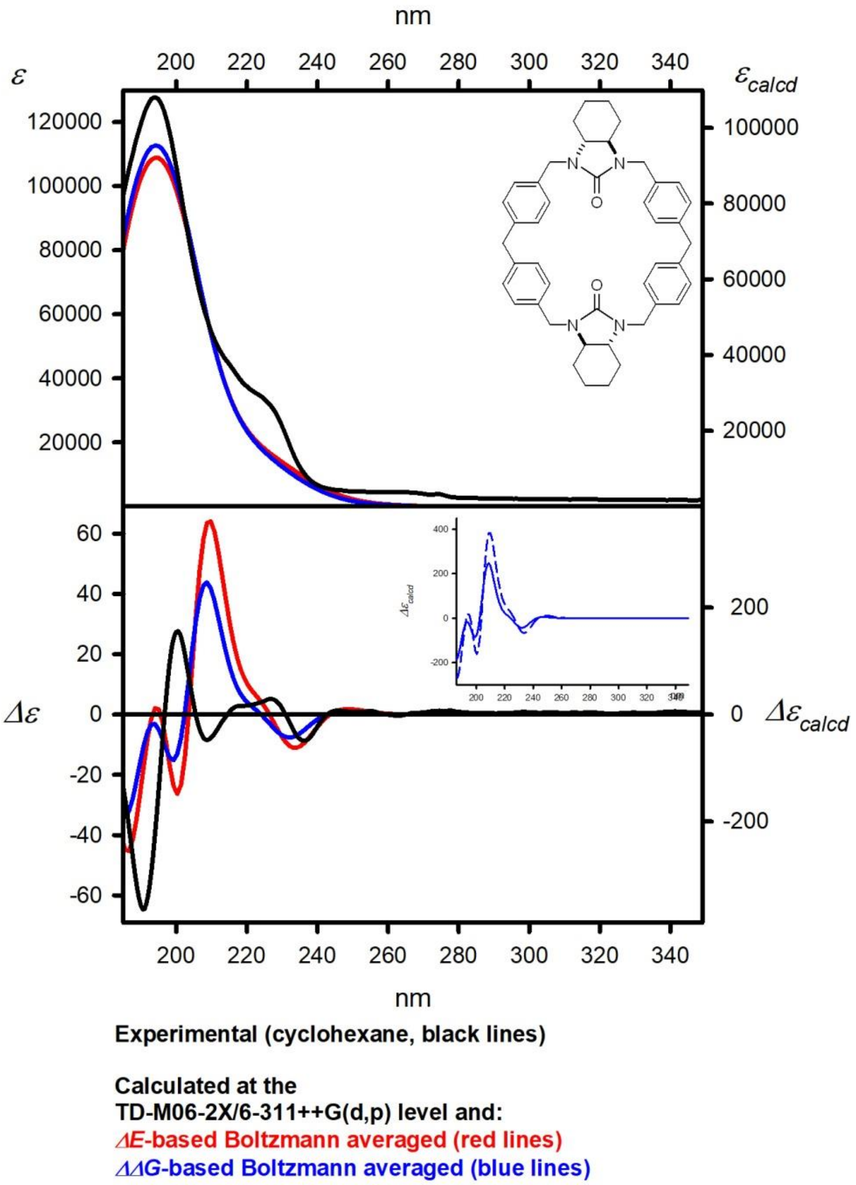

Figure S69. UV (upper panel) and ECD (lower panel) spectra of **9a** measured in cyclohexane (solid black lines) and calculated at the TD-M06-2X/6-311++G(d,p) level. The calculated ECD spectra were Boltzmann-averaged based on *Δ*E (red lines) and *ΔΔ*G values (blue lines). Wavelengths were corrected to match the experimental UV maxima. The insert shows the comparison between the ECD spectra calculated for the lowest energy conformer of a given compound (dashed blue lines) and the *ΔΔ*G-based and Boltzmann averaged (solid blue lines).

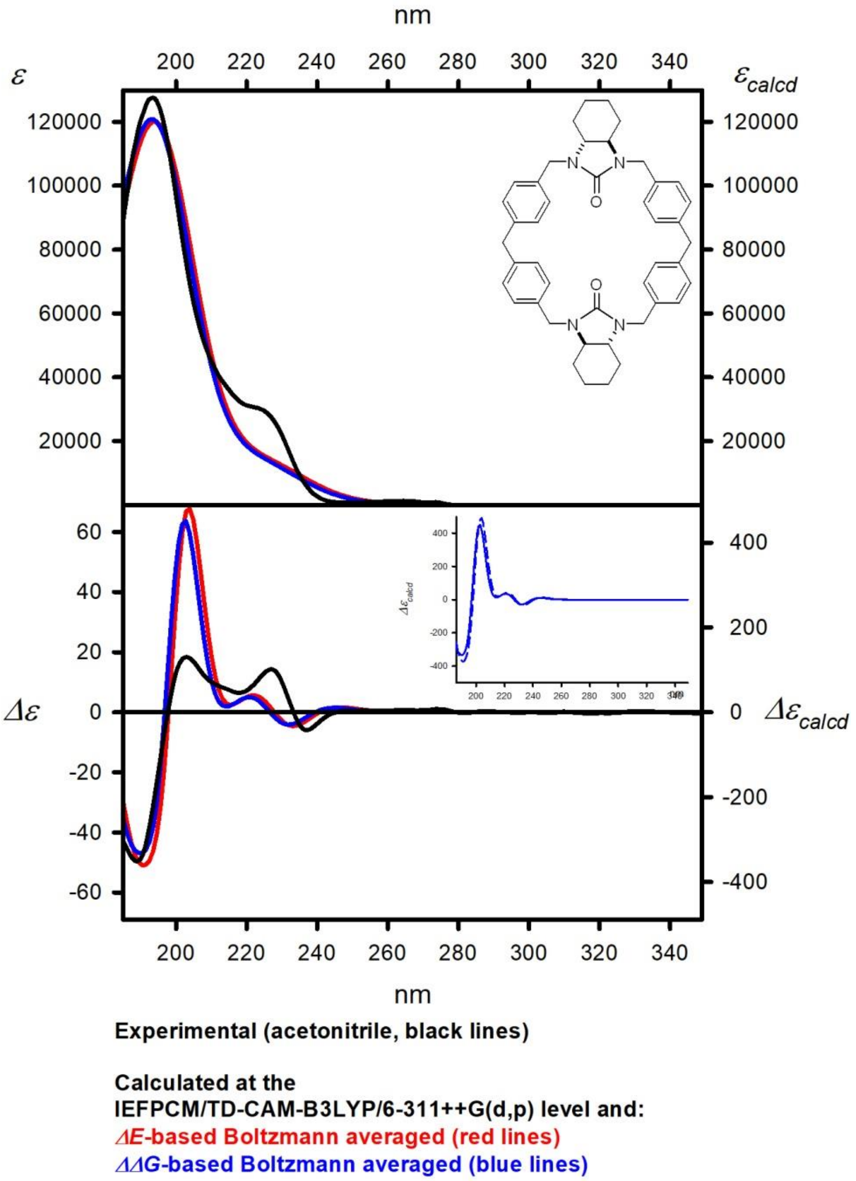

Figure S70. UV (upper panel) and ECD (lower panel) spectra of **9a** measured in acetonitrile (solid black lines) and calculated at the IEFPCM/TD-CAM-B3LYP/6-311++G(d,p) level. The calculated ECD spectra were Boltzmann-averaged based on *Δ*E (red lines) and *ΔΔ*G values (blue lines). Wavelengths were corrected to match the experimental UV maxima. The insert shows the comparison between the ECD spectra calculated for the lowest energy conformer of a given compound (dashed blue lines) and the *ΔΔ*G-based and Boltzmann averaged (solid blue lines).

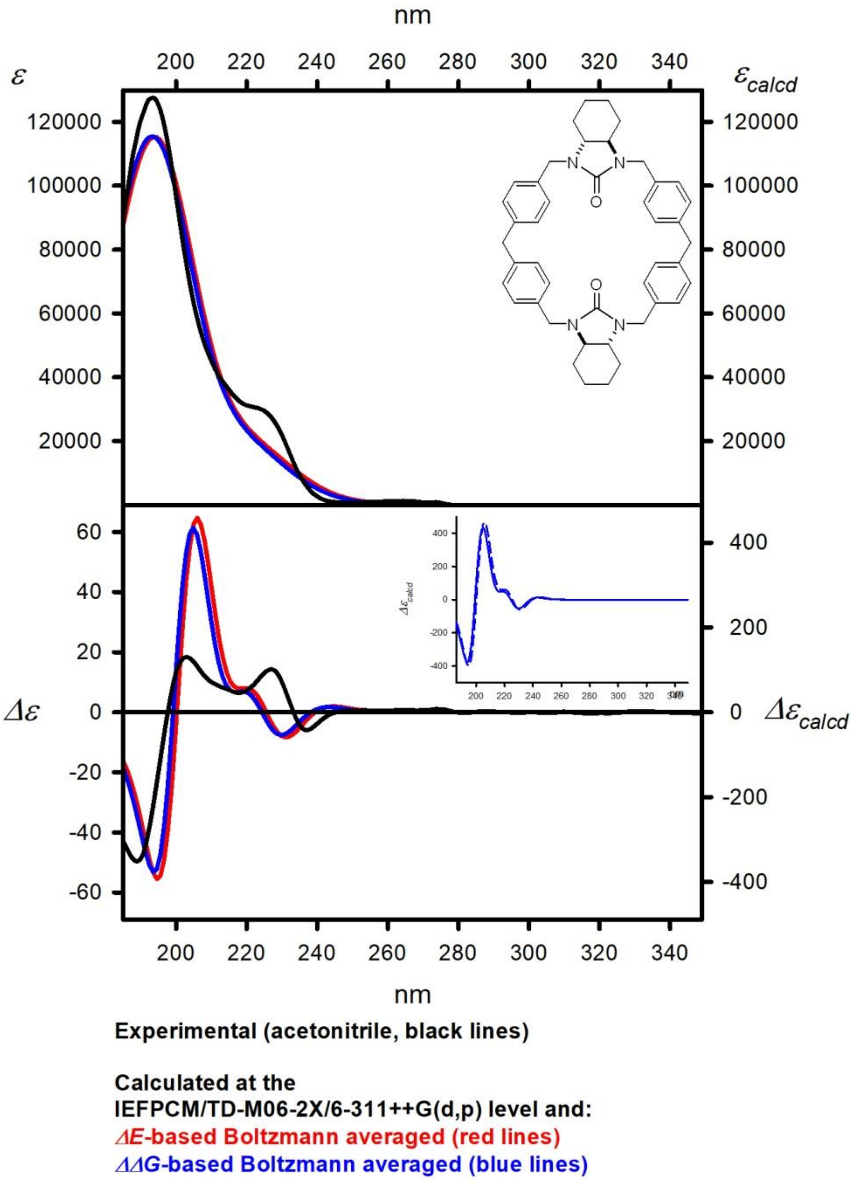

Figure S71. UV (upper panel) and ECD (lower panel) spectra of **9a** measured in acetonitrile (solid black lines) and calculated at the IEFPCM/TD-M06-2X/6-311++G(d,p) level. The calculated ECD spectra were Boltzmann-averaged based on *Δ*E (red lines) and *ΔΔ*G values (blue lines). Wavelengths were corrected to match the experimental UV maxima. The insert shows the comparison between the ECD spectra calculated for the lowest energy conformer of a given compound (dashed blue lines) and the *ΔΔ*G-based and Boltzmann averaged (solid blue lines).

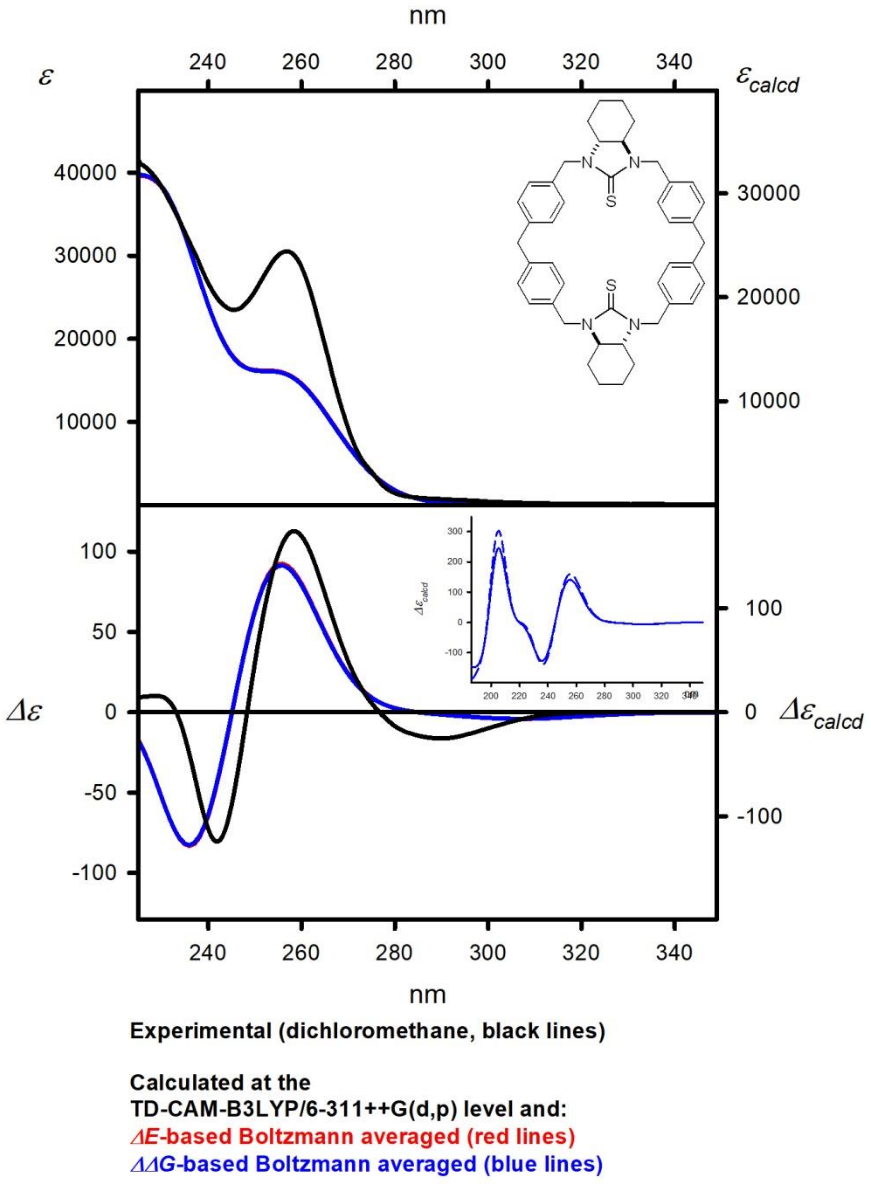

Figure S72. UV (upper panel) and ECD (lower panel) spectra of **9b** measured in dichloromethane (solid black lines) and calculated at the TD-CAM-B3LYP/6-311++G(d,p) level. The calculated ECD spectra were Boltzmann-averaged based on *Δ*E (red lines) and *ΔΔ*G values (blue lines). Wavelengths were corrected to match the experimental UV maxima. The insert shows the comparison between the ECD spectra calculated for the lowest energy conformer of a given compound (dashed blue lines) and the *ΔΔ*G-based and Boltzmann averaged (solid blue lines).

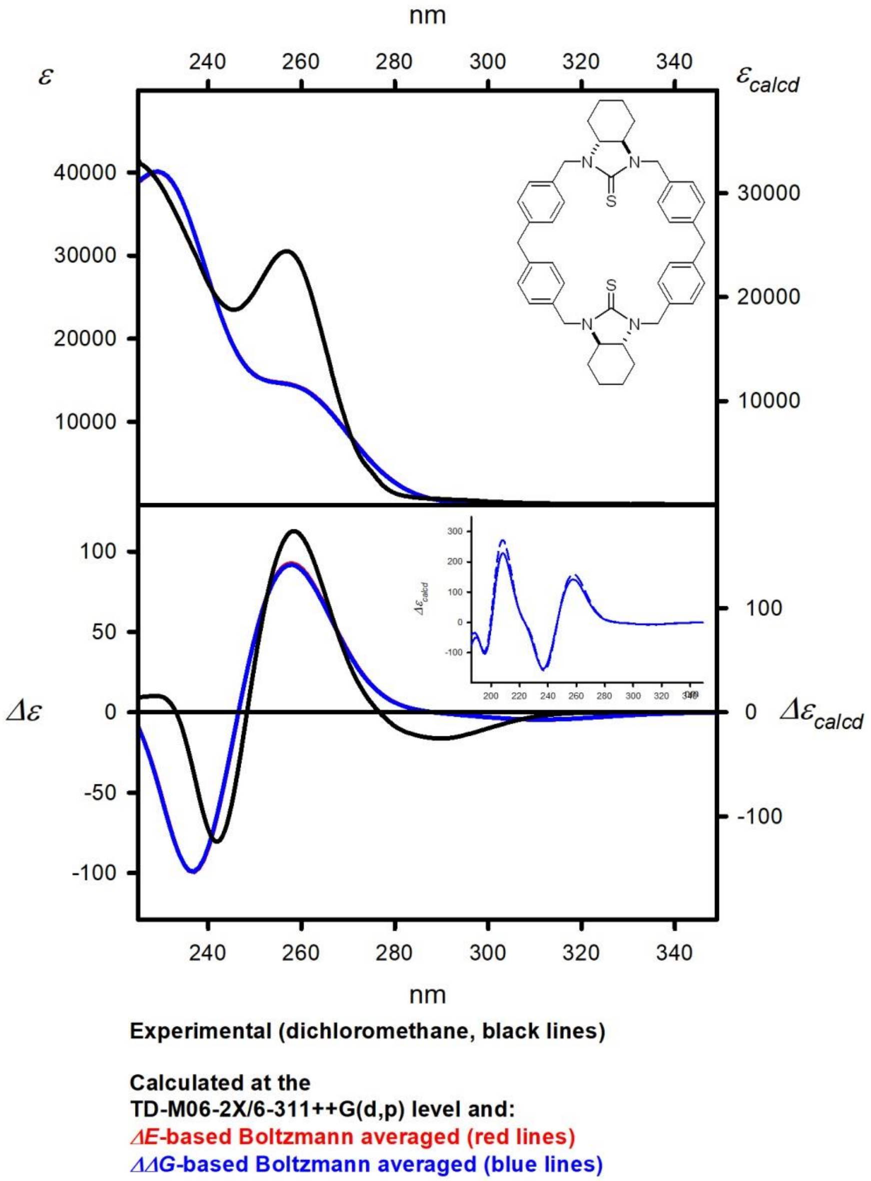

Figure S73. UV (upper panel) and ECD (lower panel) spectra of **9b** measured in dichloromethane (solid black lines) and calculated at the TD-M06-2X/6-311++G(d,p) level. The calculated ECD spectra were Boltzmann-averaged based on *Δ*E (red lines) and *ΔΔ*G values (blue lines). Wavelengths were corrected to match the experimental UV maxima. The insert shows the comparison between the ECD spectra calculated for the lowest energy conformer of a given compound (dashed blue lines) and the *ΔΔ*G-based and Boltzmann averaged (solid blue lines).

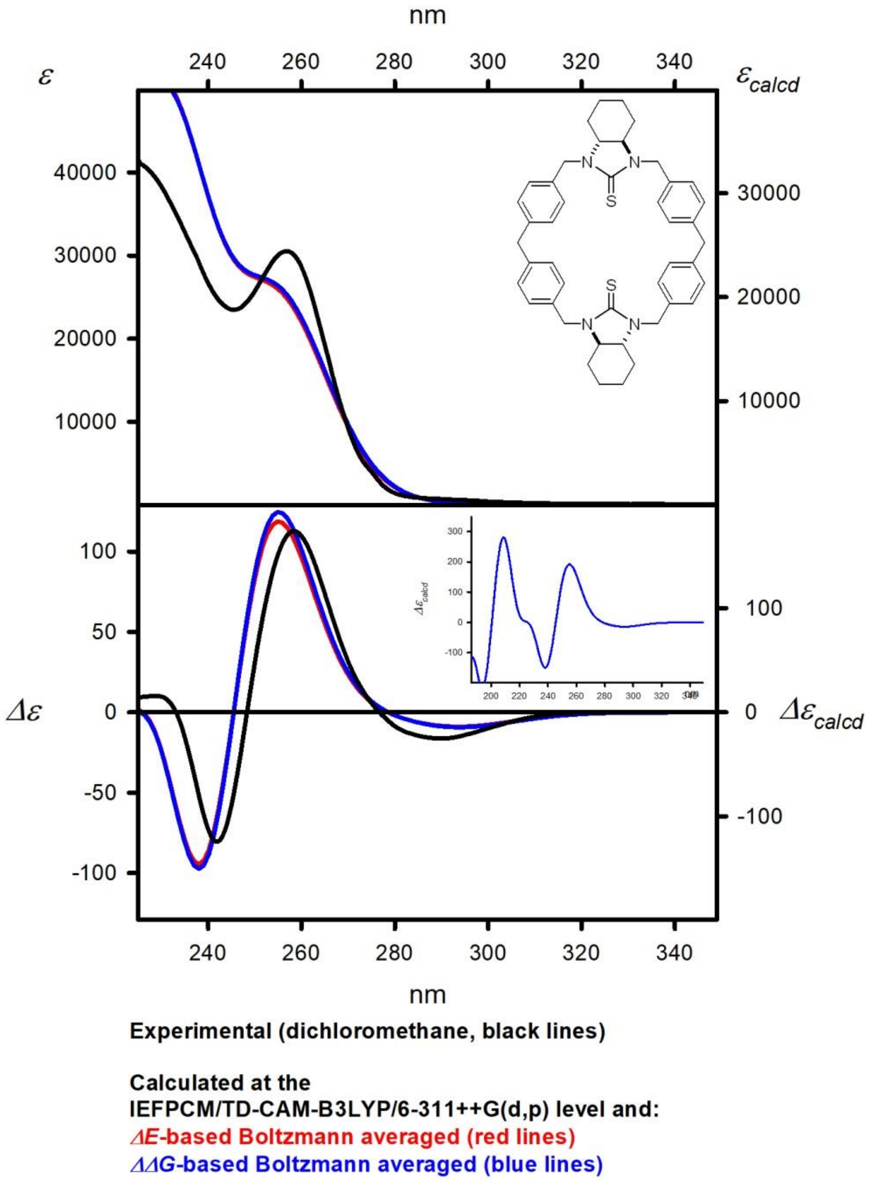

Figure S74. UV (upper panel) and ECD (lower panel) spectra of **9b** measured in dichloromethane (solid black lines) and calculated at the IEFPCM/TD-CAM-B3LYP/6-311++G(d,p) level. The calculated ECD spectra were Boltzmann-averaged based on *Δ*E (red lines) and *ΔΔ*G values (blue lines). Wavelengths were corrected to match the experimental UV maxima. The insert shows the comparison between the ECD spectra calculated for the lowest energy conformer of a given compound (dashed blue lines) and the *ΔΔ*G-based and Boltzmann averaged (solid blue lines).

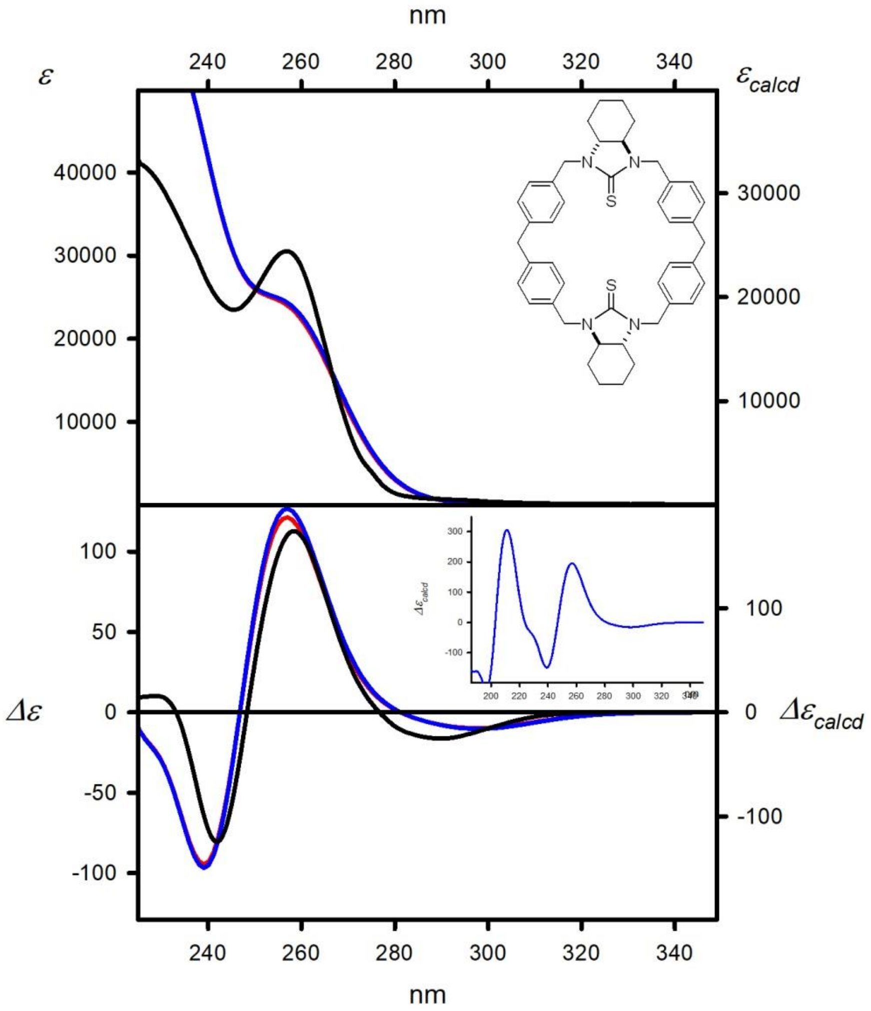

Experimental (dichloromethane, black lines)

Calculated at the
IEFPCM/TD-M06-2X/6-311++G(d,p) level and:
***ΔE*-based Boltzmann averaged (red lines)**
***ΔΔG*-based Boltzmann averaged (blue lines)**

Figure S75. UV (upper panel) and ECD (lower panel) spectra of **9b** measured in dichloromethane (solid black lines) and calculated at the IEFPCM/TD-M06-2X/6-311++G(d,p) level. The calculated ECD spectra were Boltzmann-averaged based on *Δ*E (red lines) and *ΔΔ*G values (blue lines). Wavelengths were corrected to match the experimental UV maxima. The insert shows the comparison between the ECD spectra calculated for the lowest energy conformer of a given compound (dashed blue lines) and the *ΔΔ*G-based and Boltzmann averaged (solid blue lines).

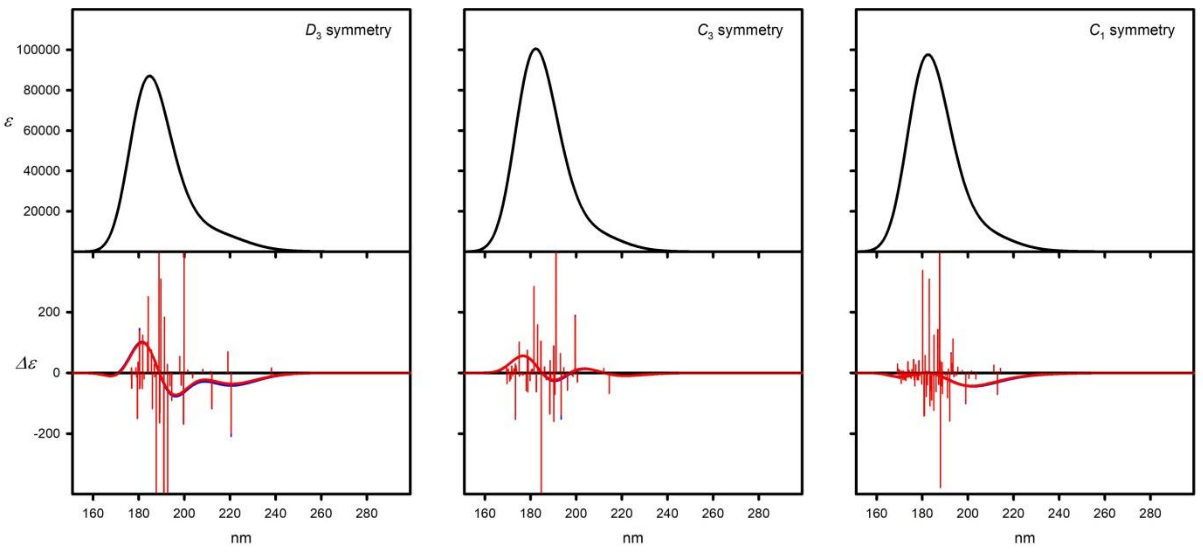

Figure S76. UV (upper panel) and ECD (lower panel) spectra of individual conformers of **1a** calculated at the TD-CAM-B3LYP/6-311++G(d,p) level. Wavelengths were not corrected, vertical bars represent rotator strengths.

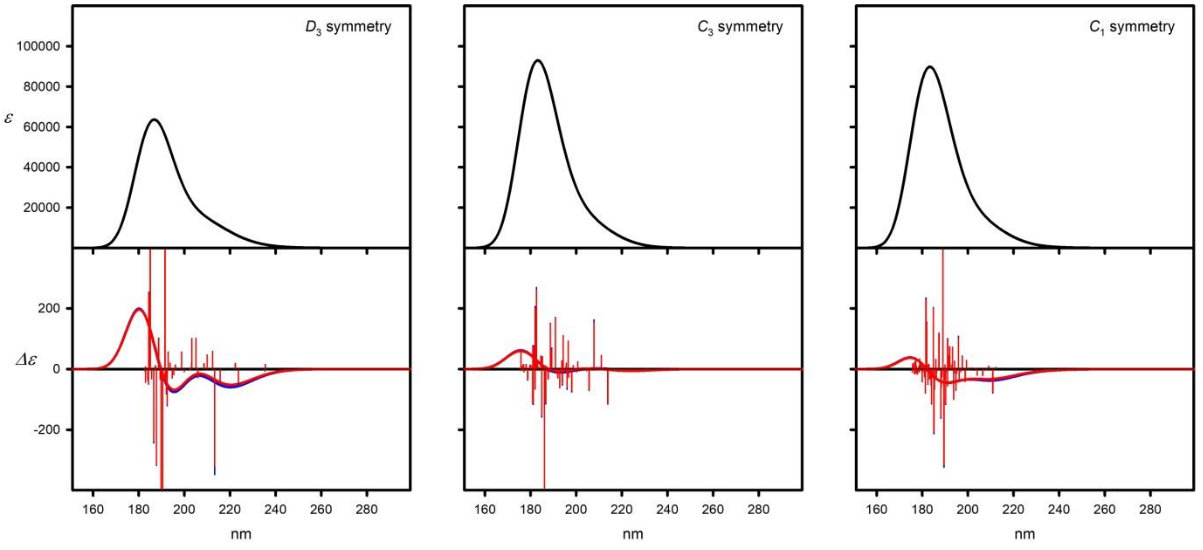

Figure S77. UV (upper panel) and ECD (lower panel) spectra of individual conformers of **1a** calculated at the TD-M06-2X/6-311++G(d,p) level. Wavelengths were not corrected, vertical bars represent rotator strengths.

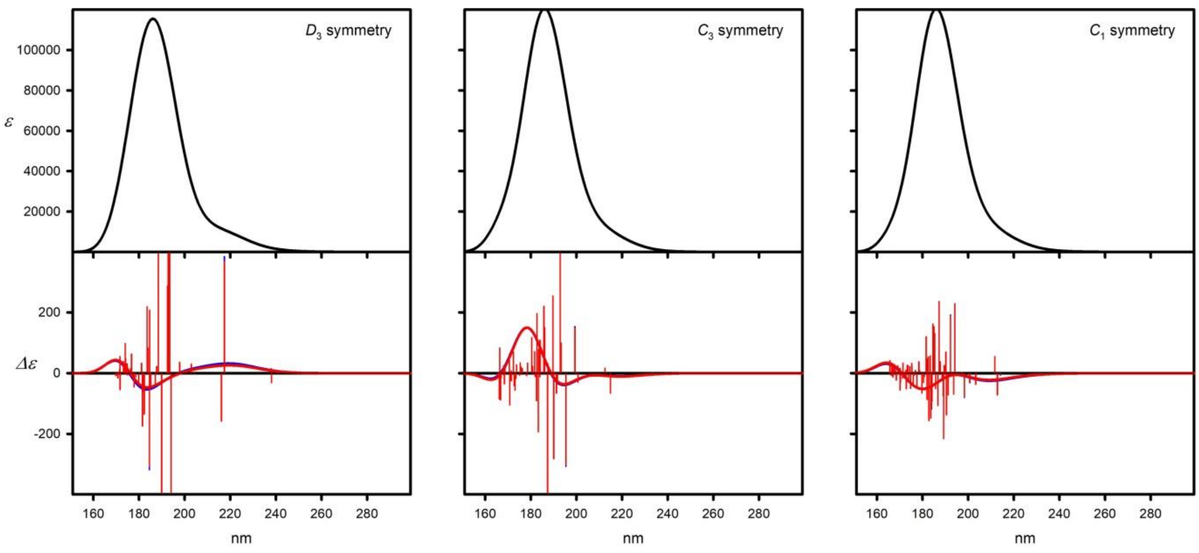

Figure S78. UV (upper panel) and ECD (lower panel) spectra of individual conformers of **1a** calculated at the IEFPCM/TD-CAM-B3LYP/6-311++G(d,p) level. Wavelengths were not corrected, vertical bars represent rotator strengths.

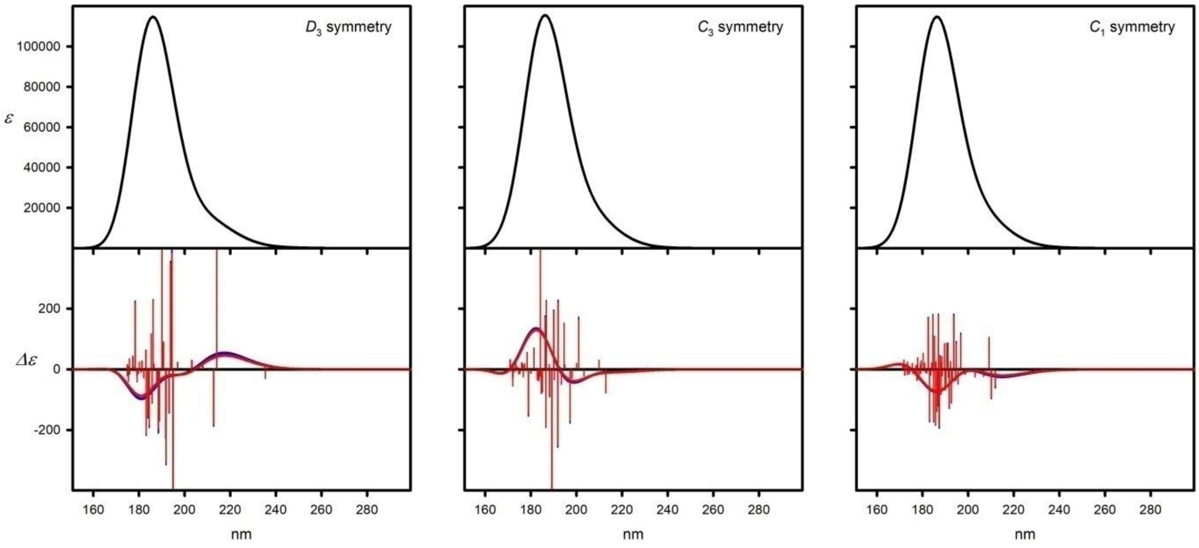

Figure S79. UV (upper panel) and ECD (lower panel) spectra of individual conformers of **1a** calculated at the IEFPCM/TD-M06-2X/6-311++G(d,p) level. Wavelengths were not corrected, vertical bars represent rotator strengths.

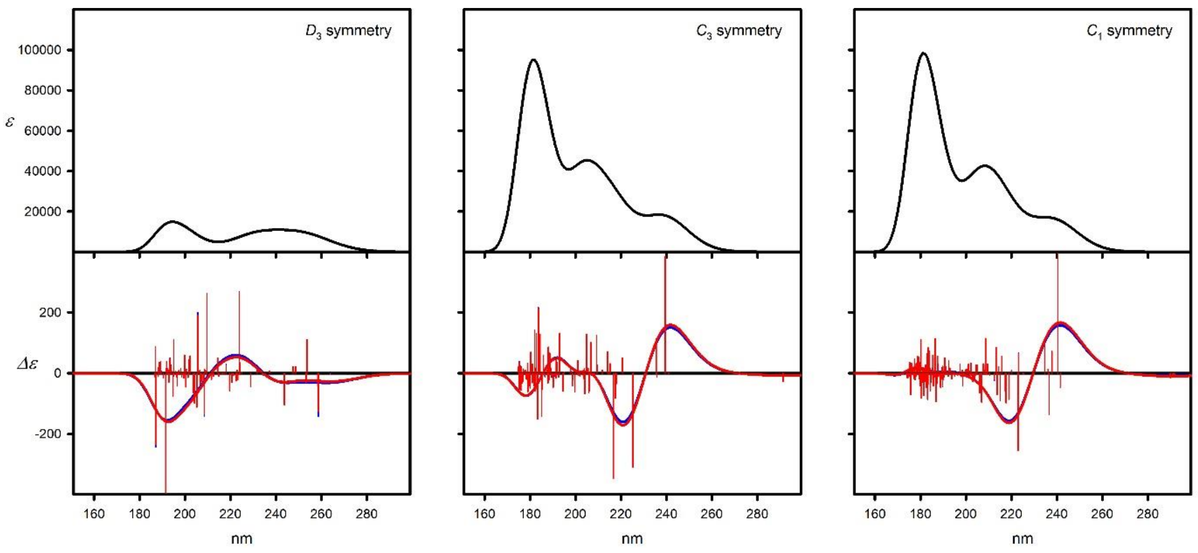

Figure S80. UV (upper panel) and ECD (lower panel) spectra of individual conformers of **1b** calculated at the TD-CAM-B3LYP/6-311++G(d,p) level. Wavelengths were not corrected, vertical bars represent rotator strengths.

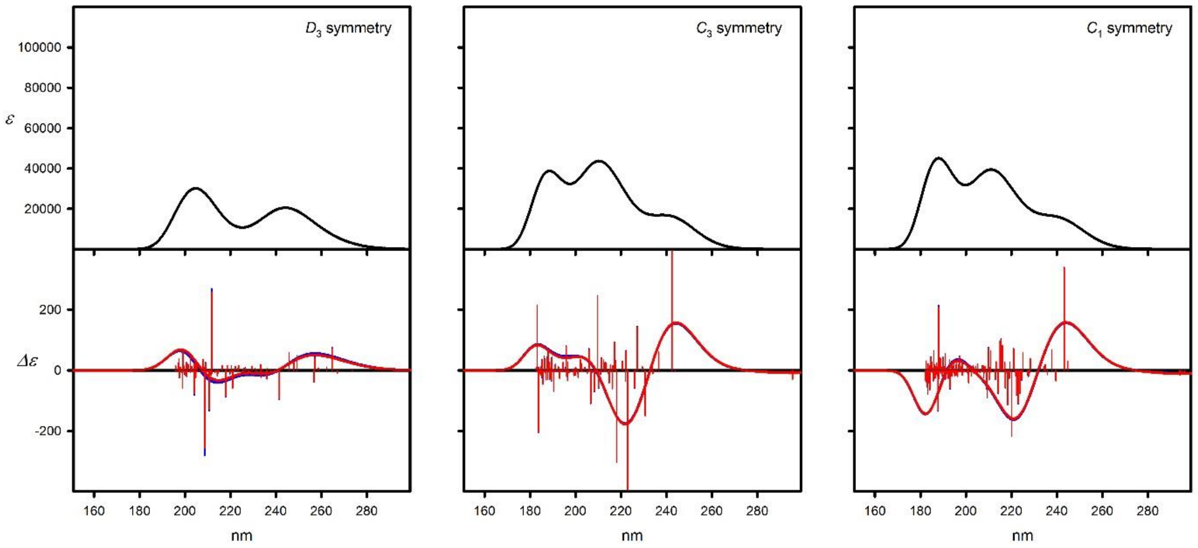

Figure S81. UV (upper panel) and ECD (lower panel) spectra of individual conformers of **1b** calculated at the TD-M06-2X/6-311++G(d,p) level. Wavelengths were not corrected, vertical bars represent rotator strengths.

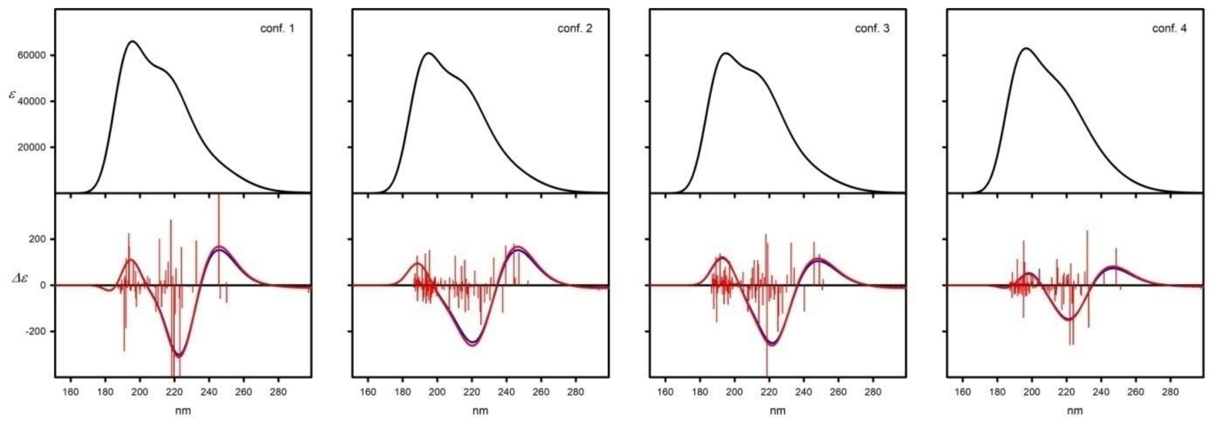

Figure S82. UV (upper panel) and ECD (lower panel) spectra of individual conformers of **3** calculated at the TD-CAM-B3LYP/6-311++G(d,p) level. Wavelengths were not corrected, vertical bars represent rotator strengths.

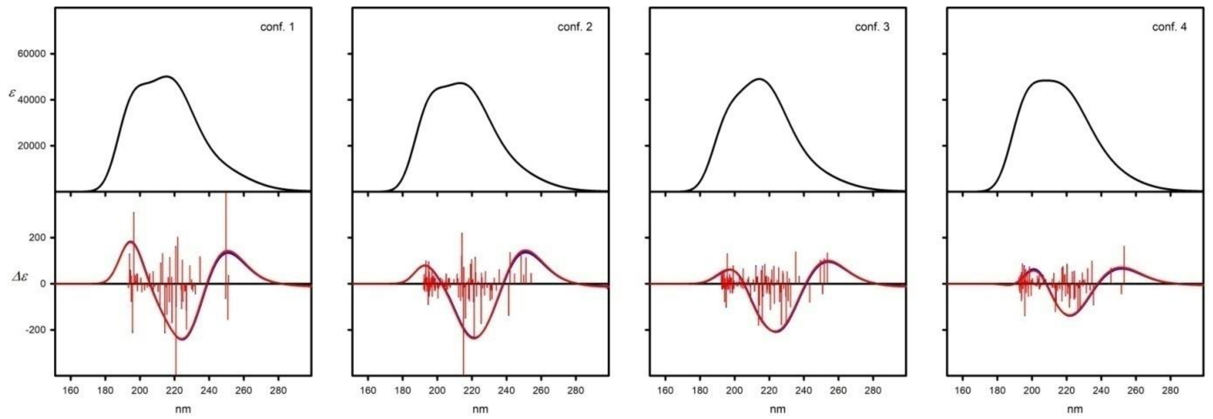

Figure S83. UV (upper panel) and ECD (lower panel) spectra of individual conformers of **3** calculated at the TD-M06-2X/6-311++G(d,p) level. Wavelengths were not corrected, vertical bars represent rotator strengths.

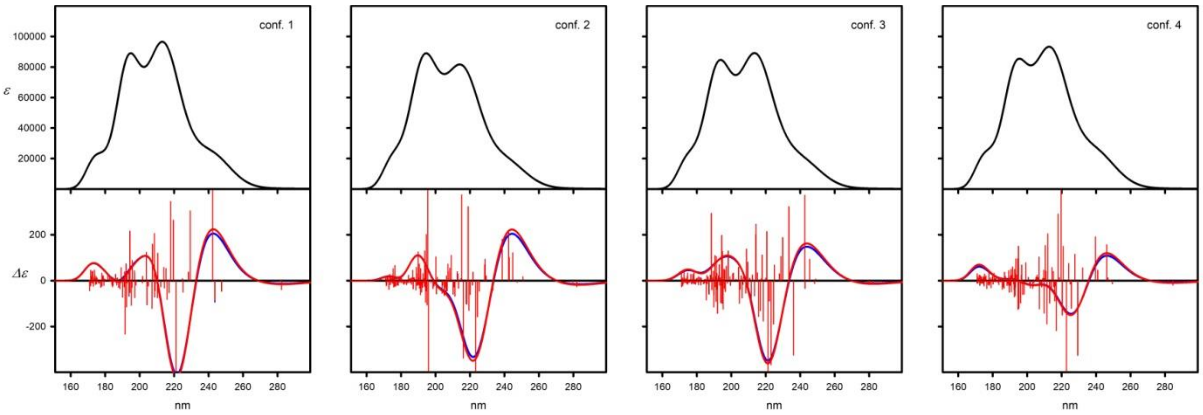

Figure S84. UV (upper panel) and ECD (lower panel) spectra of individual conformers of **3** calculated at the IEFPCM/TD-CAM-B3LYP/6-311++G(d,p) level. Wavelengths were not corrected, vertical bars represent rotator strengths.

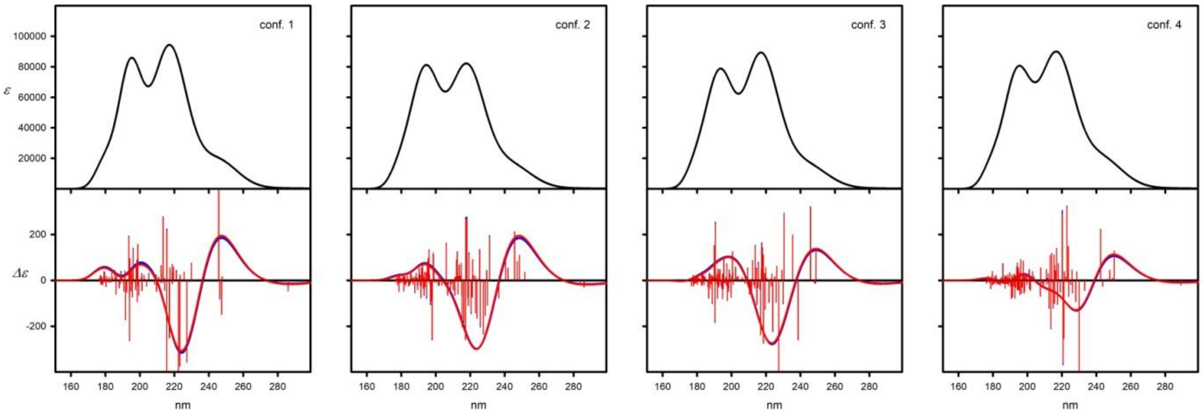

Figure S85. UV (upper panel) and ECD (lower panel) spectra of individual conformers of **3** calculated at the IEFPCM/TD-M06-2X/6-311++G(d,p) level. Wavelengths were not corrected, vertical bars represent rotator strengths.

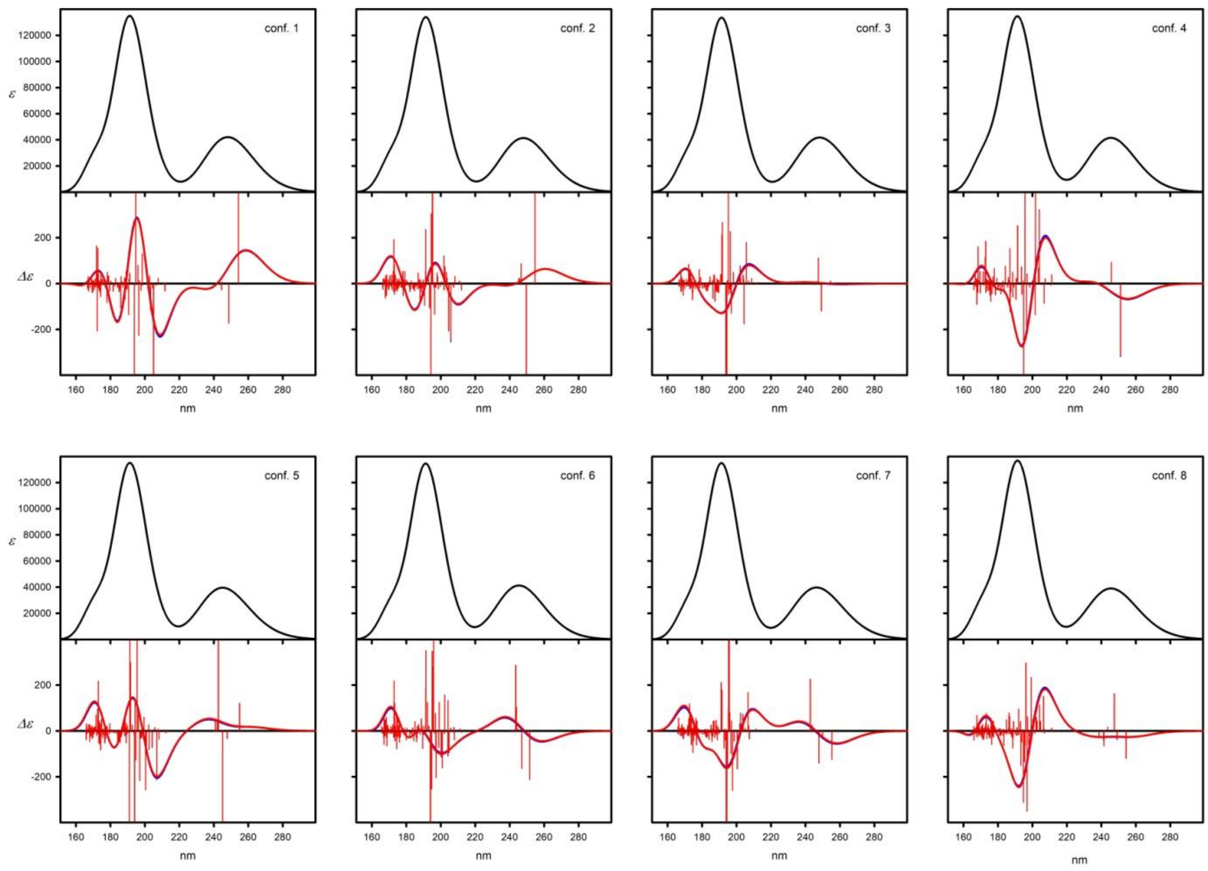

Figure S86. UV (upper panel) and ECD (lower panel) spectra of individual conformers of **4** calculated at the TD-CAM-B3LYP/6-311++G(d,p) level. Wavelengths were not corrected, vertical bars represent rotator strengths.

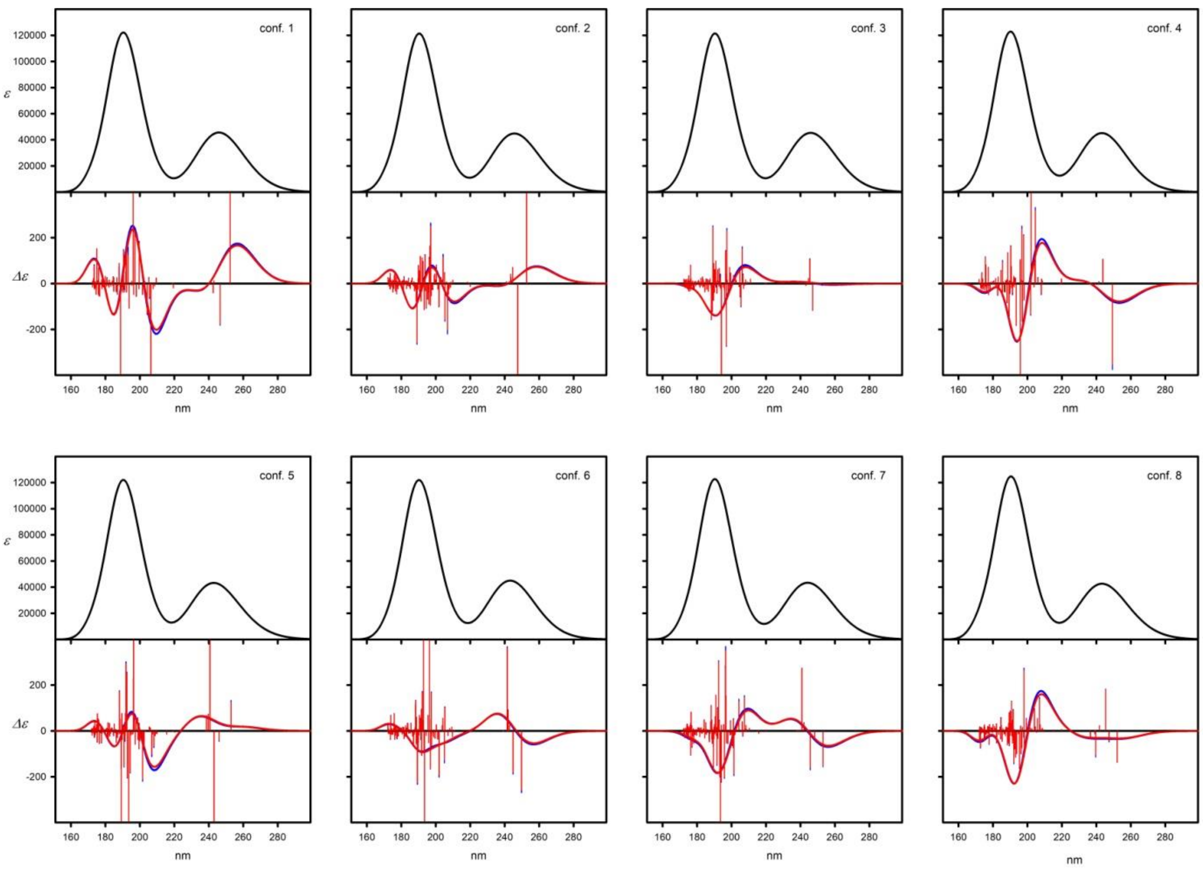

Figure S87. UV (upper panel) and ECD (lower panel) spectra of individual conformers of **4** calculated at the TD-M06-2X/6-311++G(d,p) level. Wavelengths were not corrected, vertical bars represent rotator strengths.

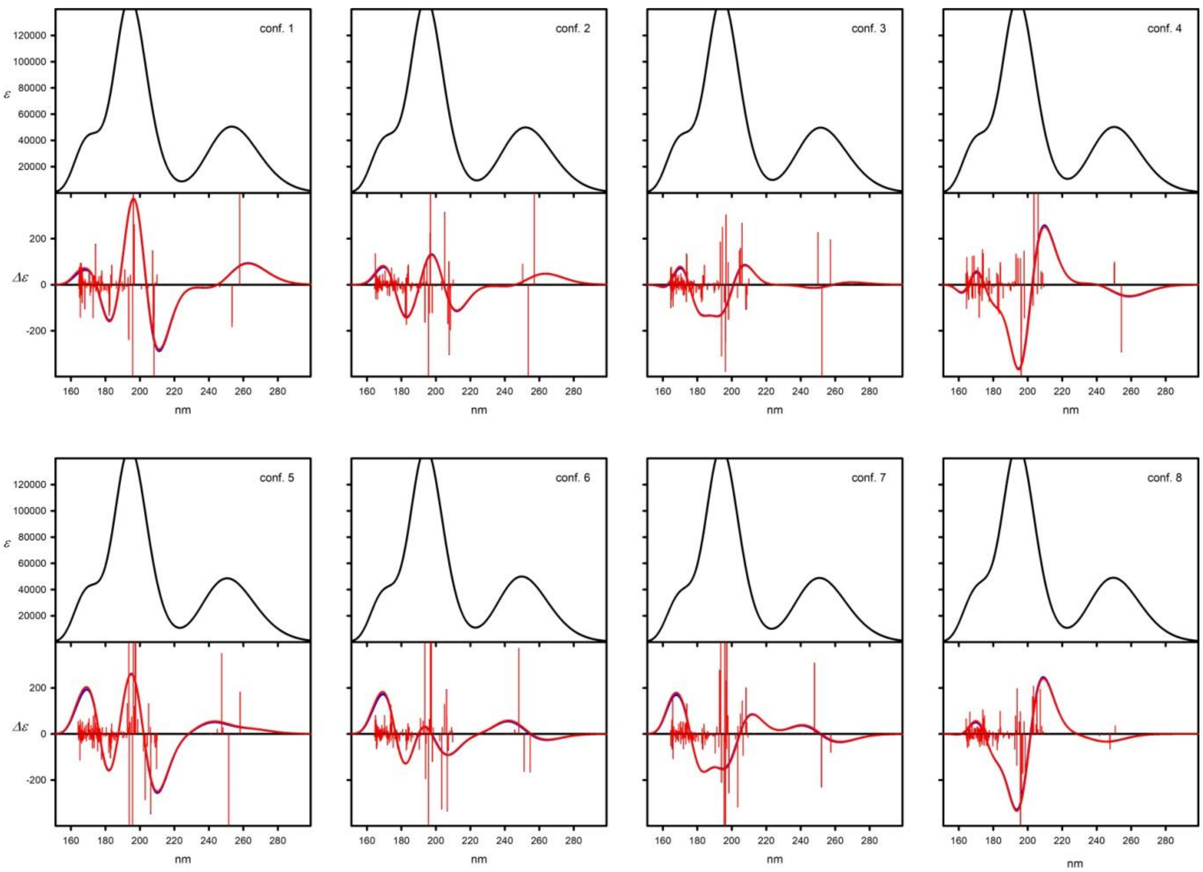

Figure S88. UV (upper panel) and ECD (lower panel) spectra of individual conformers of **4** calculated at the IEFPCM/TD-CAM-B3LYP/6-311++G(d,p) level. Wavelengths were not corrected, vertical bars represent rotator strengths.

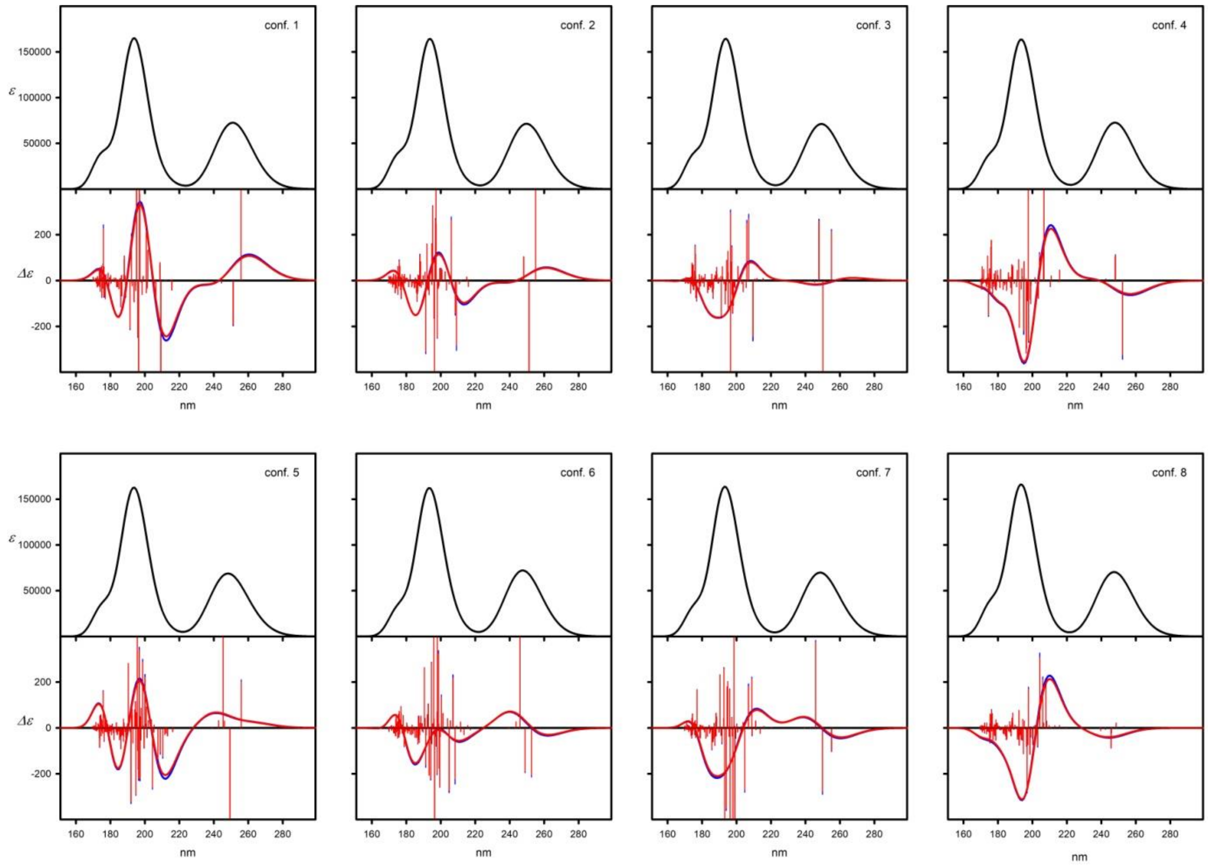

Figure S89. UV (upper panel) and ECD (lower panel) spectra of individual conformers of **4** calculated at the IEFPCM/TD-M06-2X/6-311++G(d,p) level. Wavelengths were not corrected, vertical bars represent rotator strengths.

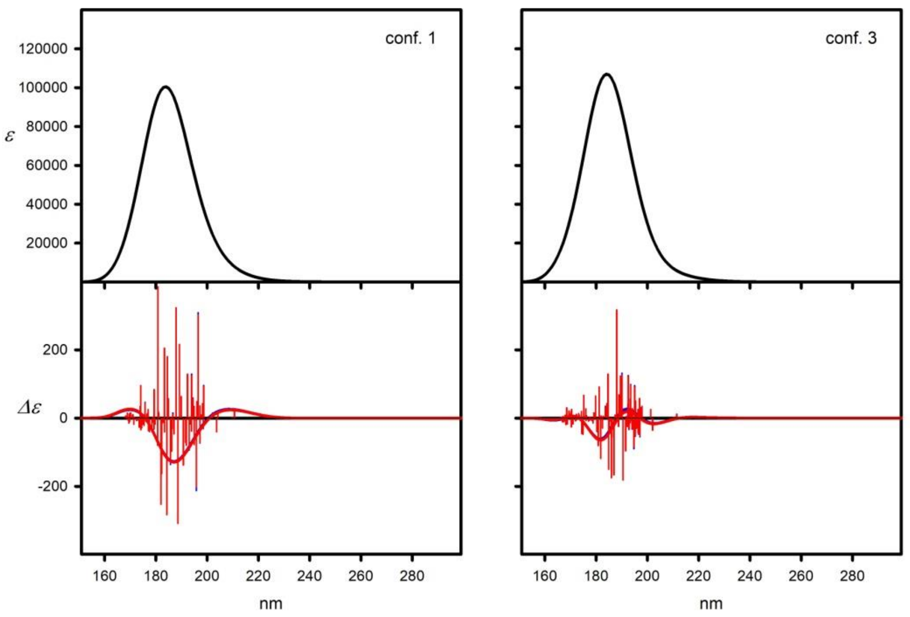

Figure S90. UV (upper panel) and ECD (lower panel) spectra of individual conformers of **5a** calculated at the TD-CAM-B3LYP/6-311++G(d,p) level. Wavelengths were not corrected, vertical bars represent rotator strengths.

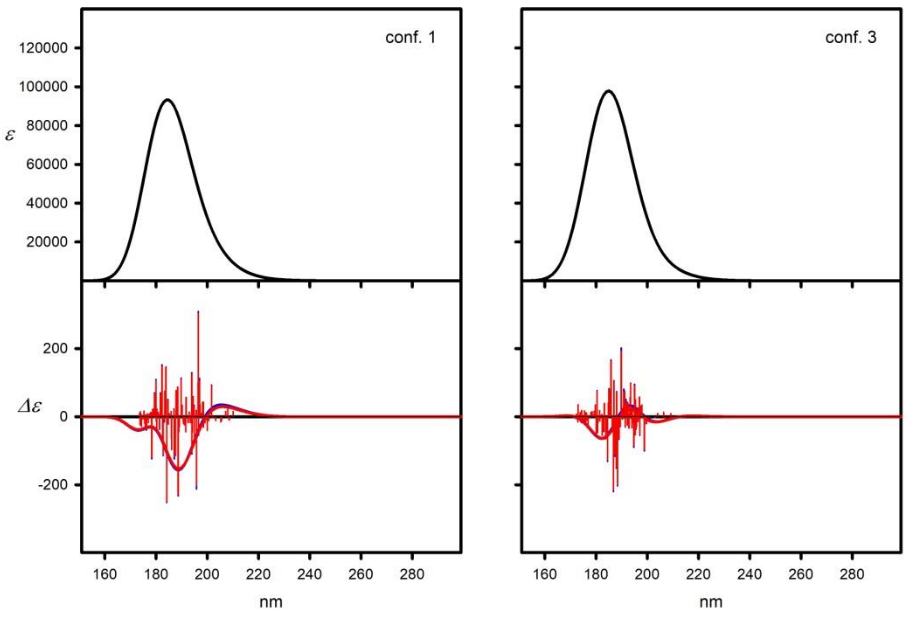

Figure S91. UV (upper panel) and ECD (lower panel) spectra of individual conformers of **5a** calculated at the TD-M06-2X/6-311++G(d,p) level. Wavelengths were not corrected, vertical bars represent rotator strengths.

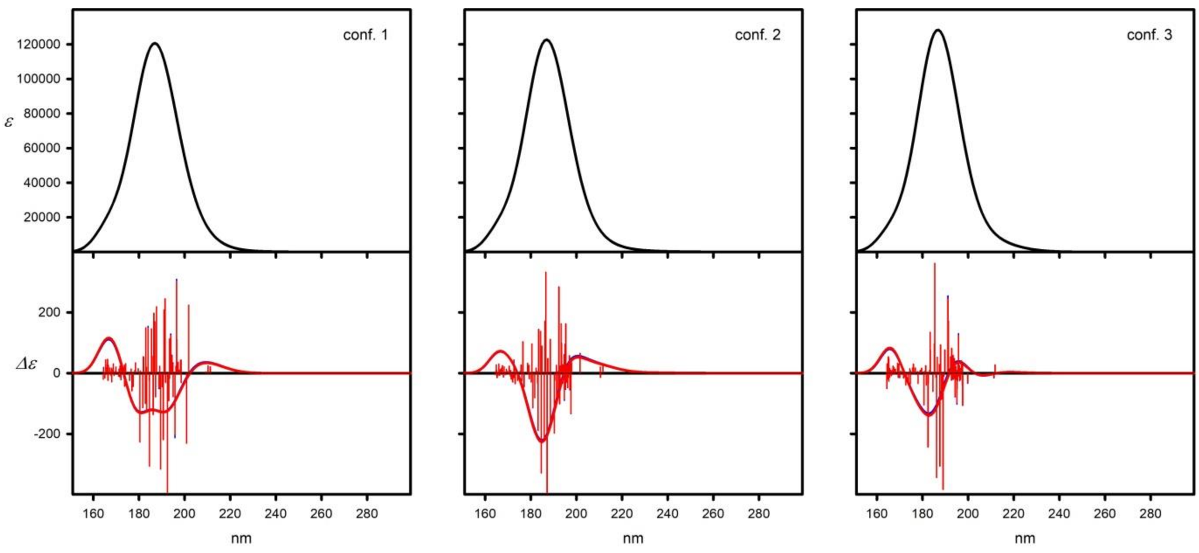

Figure S92. UV (upper panel) and ECD (lower panel) spectra of individual conformers of **5a** calculated at the IEFPCM/TD-CAM-B3LYP/6-311++G(d,p) level. Wavelengths were not corrected, vertical bars represent rotator strengths.

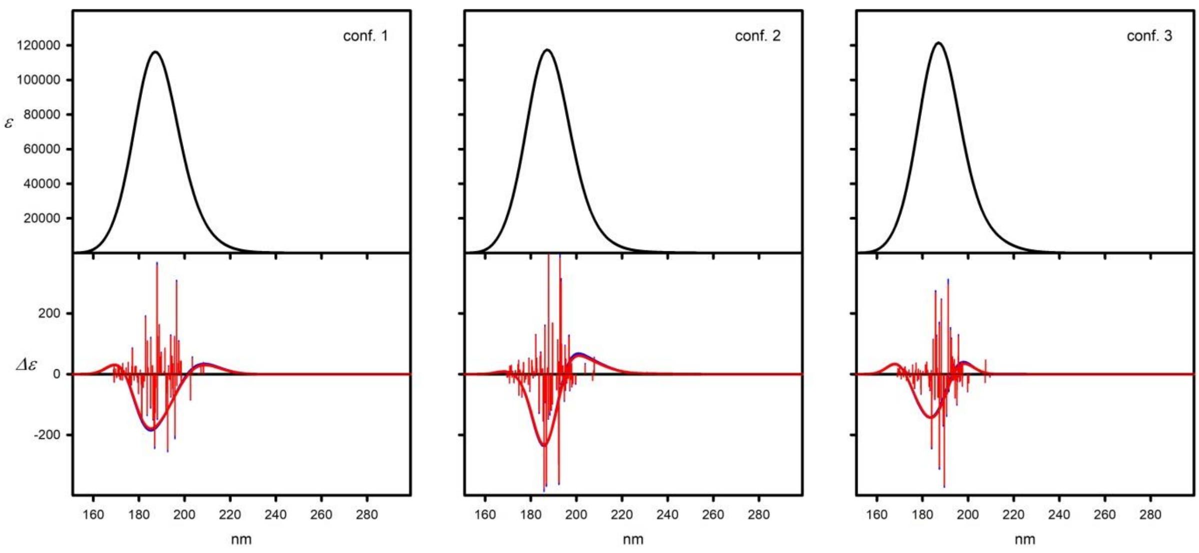

Figure S93. UV (upper panel) and ECD (lower panel) spectra of individual conformers of **5a** calculated at the IEFPCM/TD-M06-2X/6-311++G(d,p) level. Wavelengths were not corrected, vertical bars represent rotator strengths.

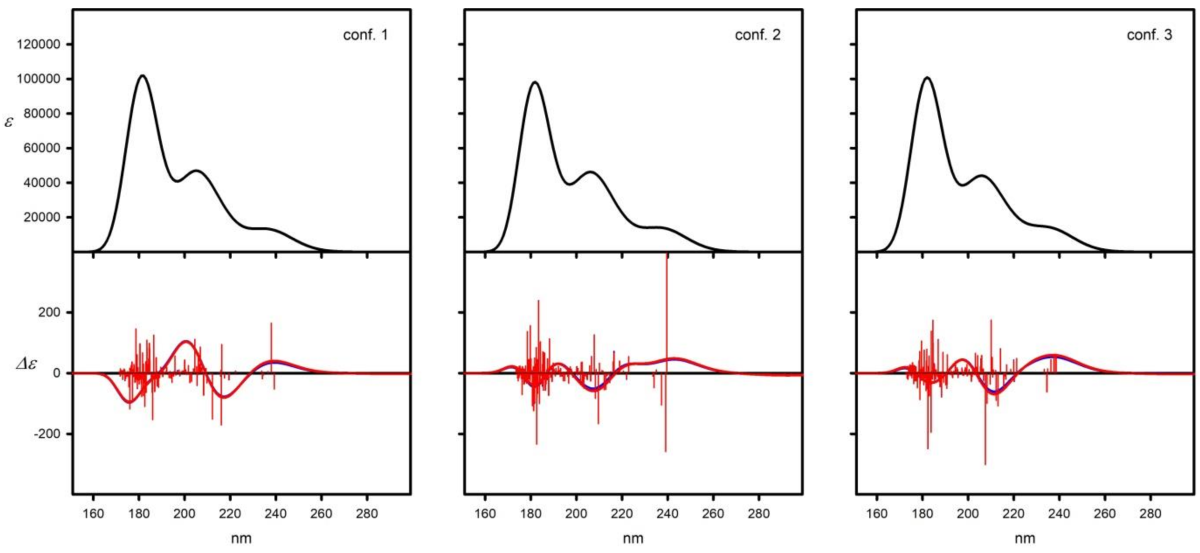

Figure S94. UV (upper panel) and ECD (lower panel) spectra of individual conformers of **5b** calculated at the TD-CAM-B3LYP/6-311++G(d,p) level. Wavelengths were not corrected, vertical bars represent rotator strengths.

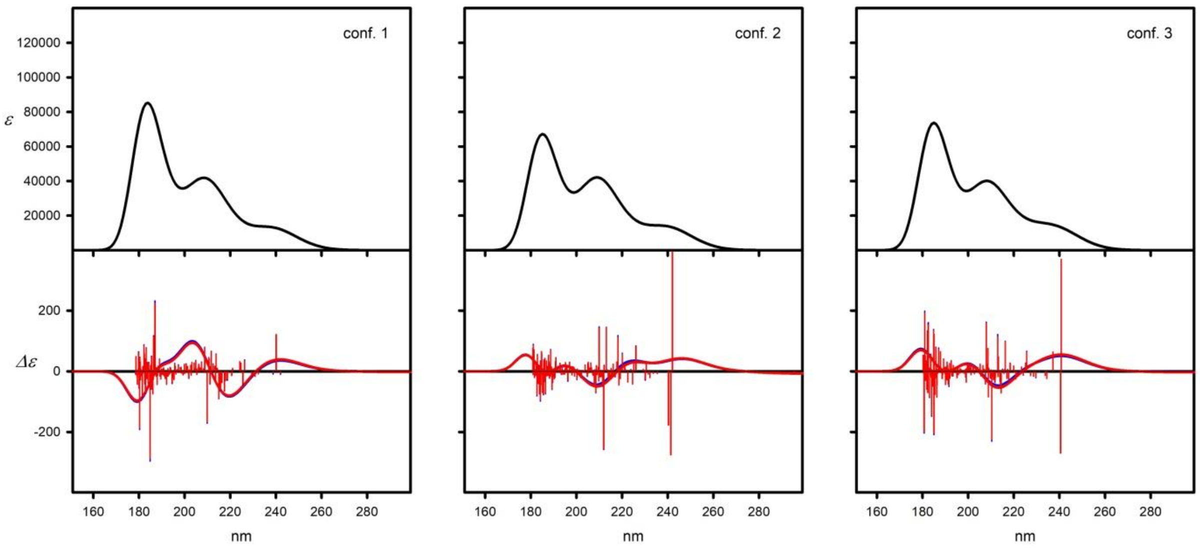

Figure S95. UV (upper panel) and ECD (lower panel) spectra of individual conformers of **5b** calculated at the TD-M06-2X/6-311++G(d,p) level. Wavelengths were not corrected, vertical bars represent rotator strengths.

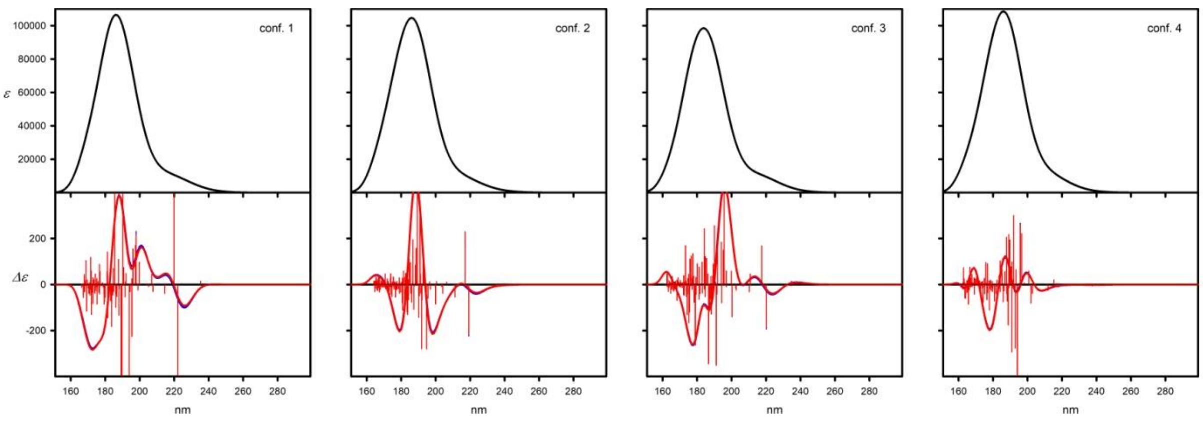

Figure S96. UV (upper panel) and ECD (lower panel) spectra of individual conformers of **9a** calculated at the TD-CAM-B3LYP/6-311++G(d,p) level. Wavelengths were not corrected, vertical bars represent rotator strengths.

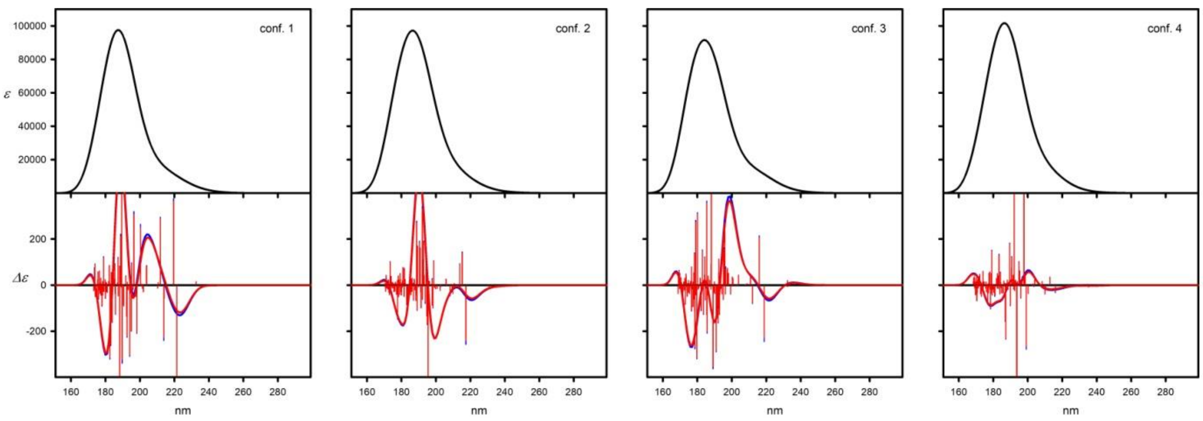

Figure S97. UV (upper panel) and ECD (lower panel) spectra of individual conformers of **9a** calculated at the TD-M06-2X/6-311++G(d,p) level. Wavelengths were not corrected, vertical bars represent rotator strengths.

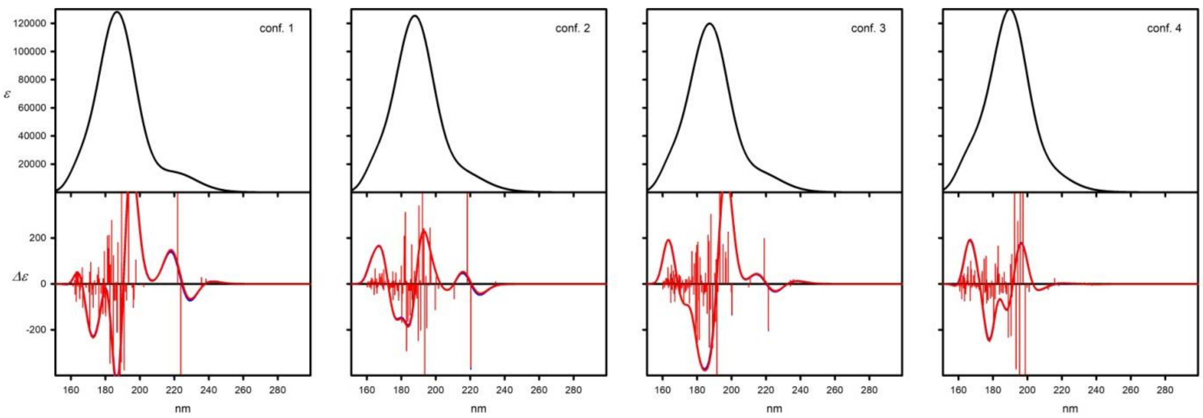

Figure S98. UV (upper panel) and ECD (lower panel) spectra of individual conformers of **9a** calculated at the IEFPCM/TD-CAM-B3LYP/6-311++G(d,p) level. Wavelengths were not corrected, vertical bars represent rotator strengths.

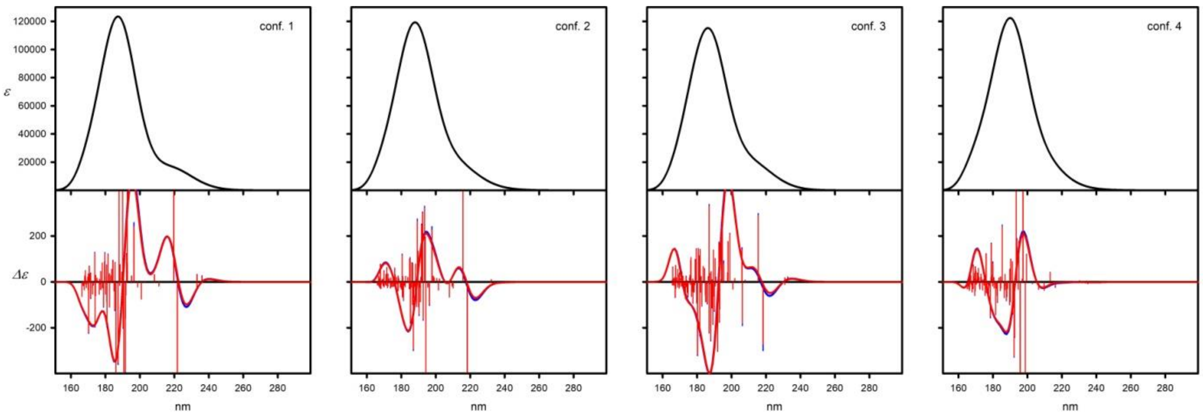

Figure S99. UV (upper panel) and ECD (lower panel) spectra of individual conformers of **9a** calculated at the IEFPCM/TD-M06-2X/6-311++G(d,p) level. Wavelengths were not corrected, vertical bars represent rotator strengths.

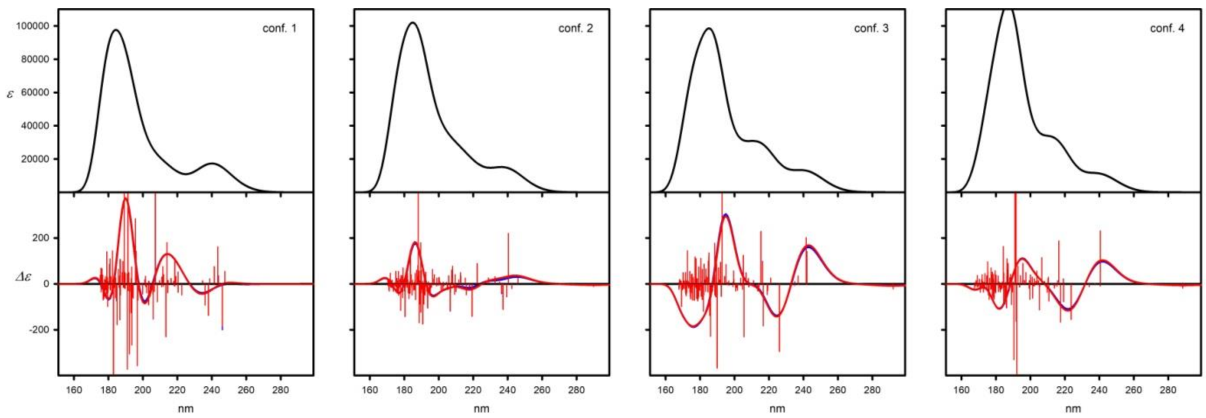

Figure S100. UV (upper panel) and ECD (lower panel) spectra of individual conformers of **9b** calculated at the TD-CAM-B3LYP/6-311++G(d,p) level. Wavelengths were not corrected, vertical bars represent rotator strengths.

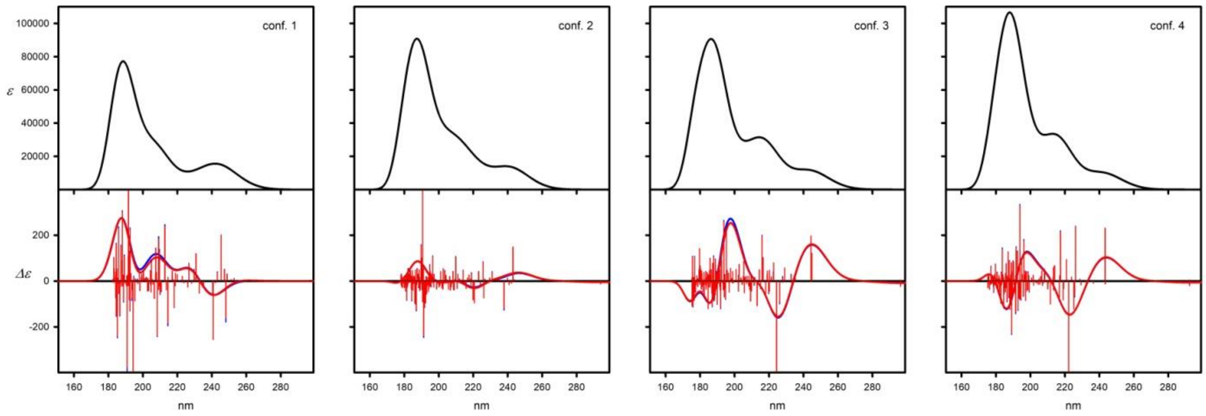

Figure S101. UV (upper panel) and ECD (lower panel) spectra of individual conformers of **9b** calculated at the TD-M06-2X/6-311++G(d,p) level. Wavelengths were not corrected, vertical bars represent rotator strengths.

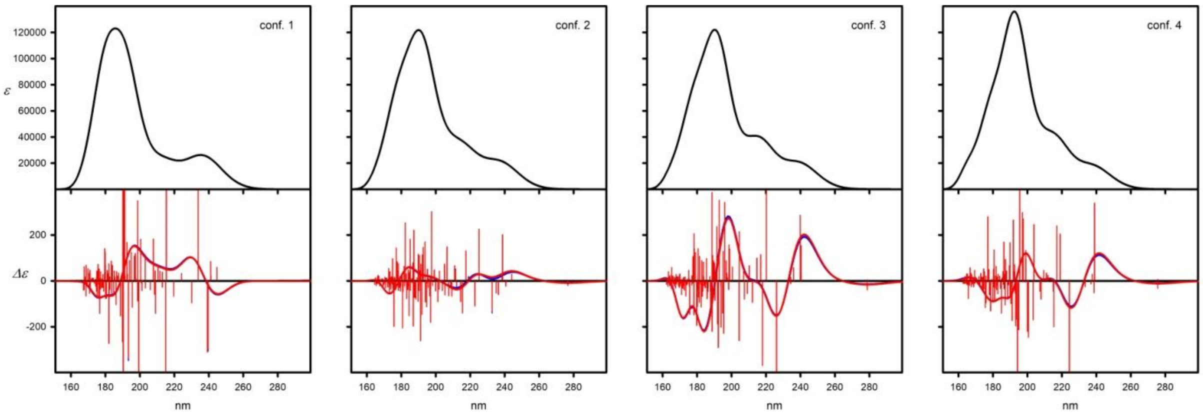

Figure S102. UV (upper panel) and ECD (lower panel) spectra of individual conformers of **9b** calculated at the IEFPCM/TD-CAM-B3LYP/6-311++G(d,p) level. Wavelengths were not corrected, vertical bars represent rotator strengths.

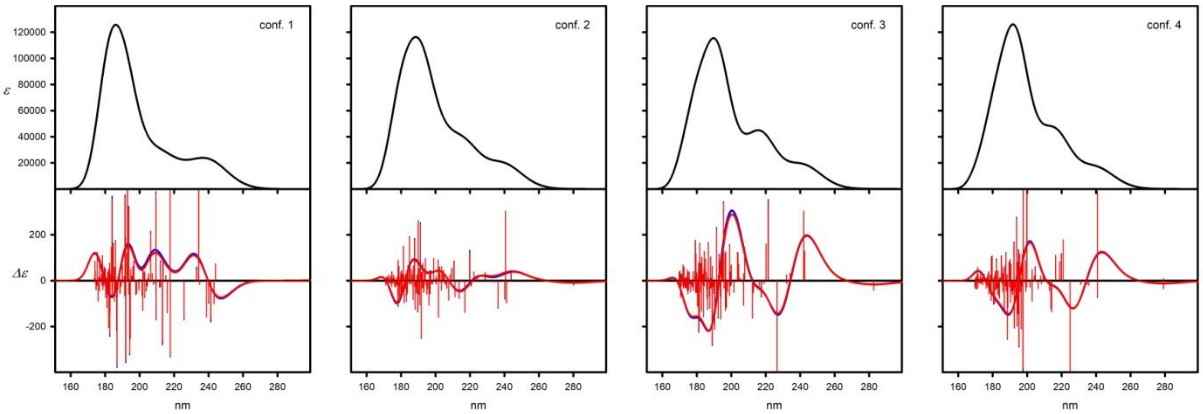

Figure S103. UV (upper panel) and ECD (lower panel) spectra of individual conformers of **9b** calculated at the IEFPCM/TD-M06-2X/6-311++G(d,p) level. Wavelengths were not corrected, vertical bars represent rotator strengths.

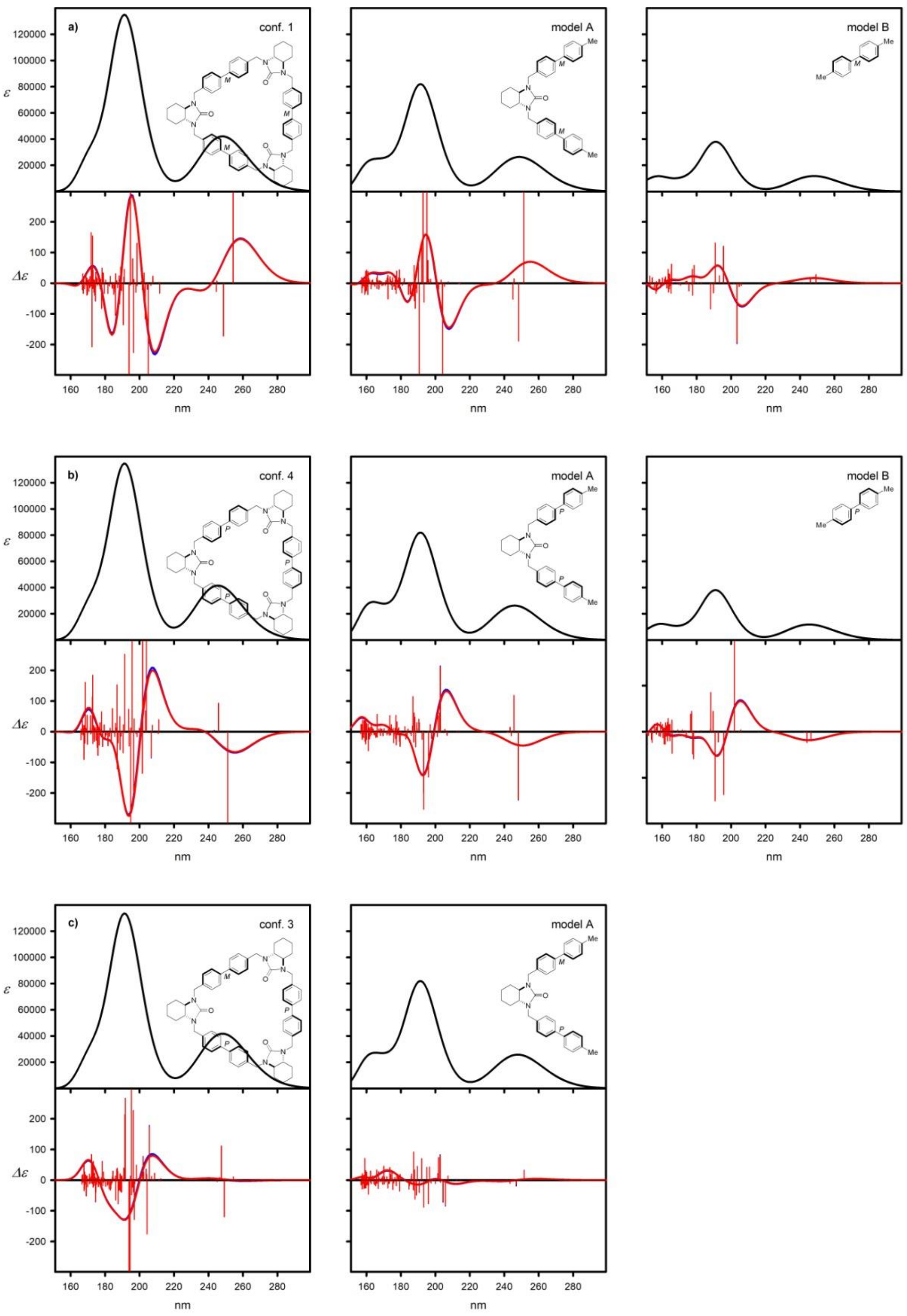

Figure S104. UV (upper panel) and ECD (lower panel) spectra of individual conformers of **4** (left column), and the model compounds A (middle column) and B (right column) calculated at the TD-CAM-B3LYP/6-311++G(d,p) level. Wavelengths were not corrected, vertical bars represent rotator strengths.

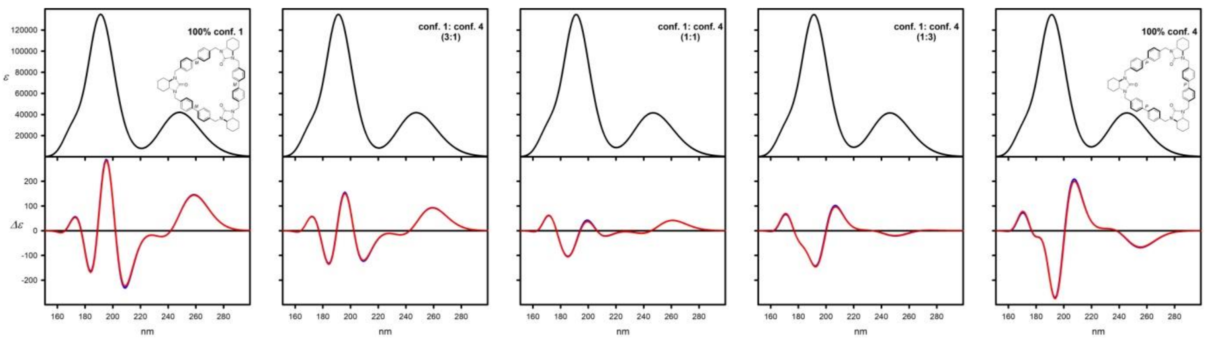

Figure S105. Simulated UV (upper panel) and ECD (lower panel) spectra of **4**, as the function of the amounts of the most abundant low-energy conformers 1 and 4 of **4**. The spectra were calculated at the TD-CAM-B3LYP/6-311++G(d,p) level. Wavelengths were not corrected.

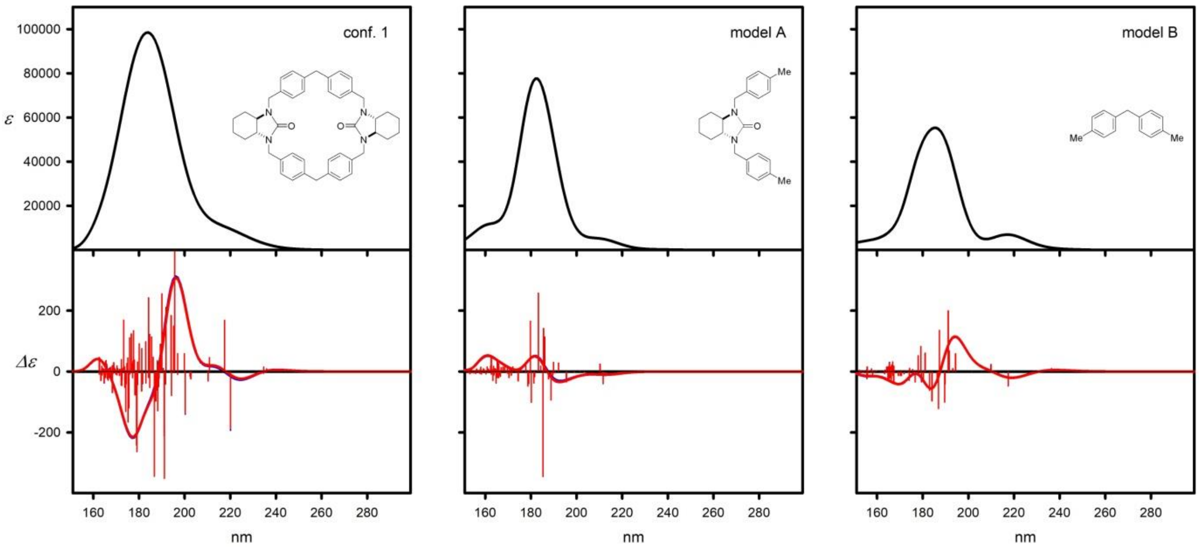

Figure S106. UV (upper panel) and ECD (lower panel) spectra of the lowest energy conformer no. 1 of **9** (left column), and the model compounds A (middle column) and B (right column), calculated at the TD-CAM-B3LYP/6-311++G(d,p) level. Wavelengths were not corrected, vertical bars represent rotator strengths.

Structures of urea-derived macrocycle 1a in the crystal phase

Colorless crystals suitable for single-crystal X-ray diffraction were obtained by slow evaporation of tetrahydrofuran solution (**1a_I**) and by slow diffusion of diethyl ether vapors to chloroform solution (**1a_II**).

Compound **1a** crystallizes in orthorhombic system in $P2_12_12_1$ space group with 4 macrocyclic molecules and: 1.8 molecules of THF and 0.2 molecules of water in form I (**1a_I**); 8 molecules of $CHCl_3$ in form II (**1a_II**) in unit cell. The both forms are isostructural.

In form I and II, disordered solvent molecules fill voids connected to each other by system of narrow channels. One guest molecule occupied the space above macrocycle ring and is stabilized by two cyclohexane groups, while the second solvent molecule's position is located between host molecules.

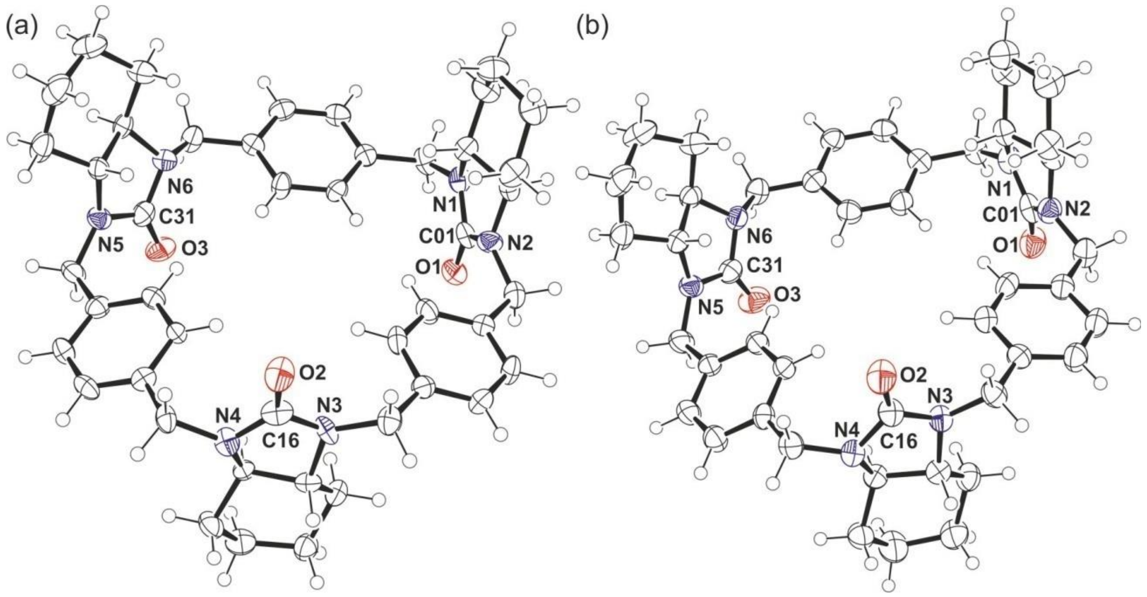

Figure S107. Structures of macrocycle **1a** as present in the crystal form: (a) I and (b) II. Displacement ellipsoids are drawn at the 50% probability level. For clarity, only selected heteroatoms are labeled. Hydrogen atoms are represented in arbitrary radii.

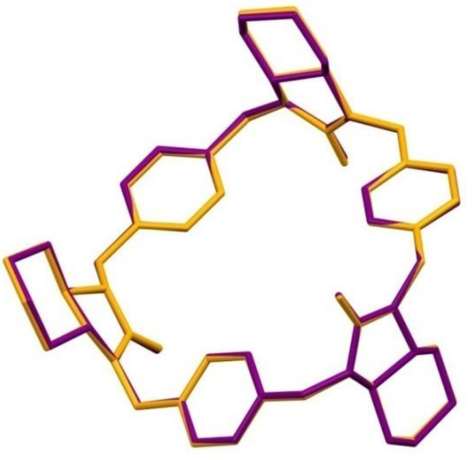

Figure S108. Overlaid molecular structures of macrocycle **1a** from **1a_**I (orange) and **1a_II** (purple) in the crystal forms. Hydrogen atoms are omitted for clarity.

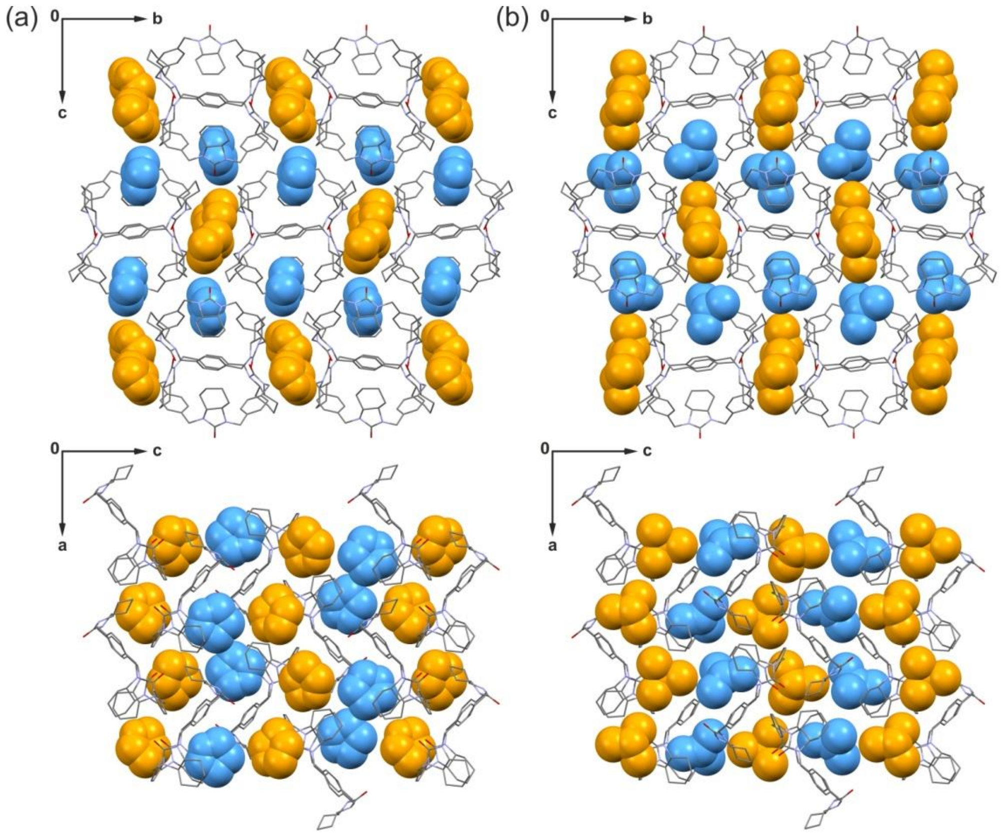

Figure S109. Packing diagrams of host molecules of **1a_I** (a) and **1a_II** (b) displayed as sticks and guest molecules displayed as spacefills. Asymmetry independent solvent molecules are distinguished by different colors. Solvent molecules are represented in the highest occupation rate. Hydrogen atoms are omitted for clarity. Views along [100] and [010] directions (upper and lower panel, respectively).

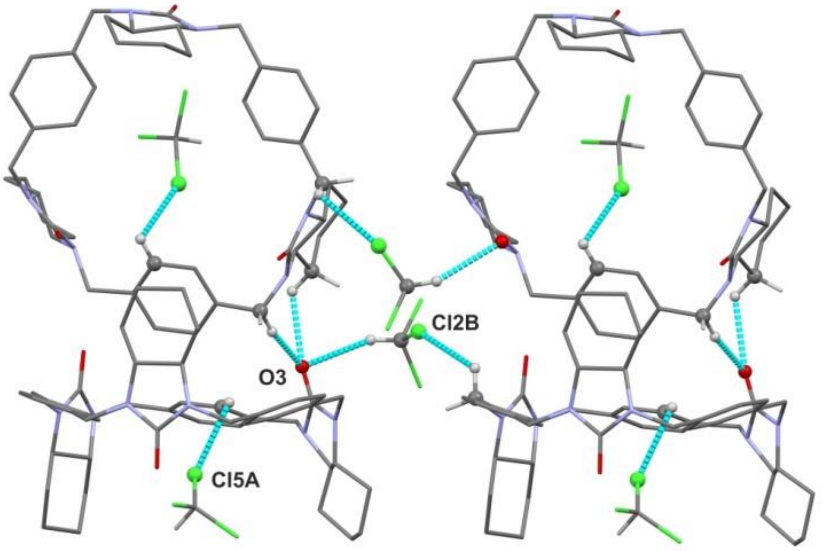

Figure S110. Supramolecular assembly in the crystal of **1a_II**. Selected hydrogen bonds are marked in blue dash lines.

Structures of thiourea-derived macrocycle 1b in the crystal phase

In our previous study, we reported the first crystal structure of compound **1b**, and here, it's identified as form I – **1b_I**.[10]

In this study, colorless crystal suitable for single-crystal X-ray diffraction were obtained by slow diffusion of diethyl ether vapors to chloroform solution (**1b_II**). In form II (**1b_II**), compound **1b** crystallizes in orthorhombic system in $P2_12_12_1$ space group with 4 macrocyclic molecules, 4 ordered and 2.5 highly disordered chloroform molecules in unit cell.

Ordered chloroform molecules fill voids between host molecules and are involved in intermolecular interactions that stabilize the host–guest structure, while disordered molecules fill structural channels created around 2_1 screw axes, along [100] direction. Solvent molecules, located in structural channel, were highly disordered and could not be resolved to yield a satisfactory model, therefore their unresolved electron density was treated with PLATON/SQUEEZE.[26] The estimated electron count is 161 in an accessible void volume of 482 Å^3 and can indicate to squeezed of 2.5 molecule of chloroform per unit cell.

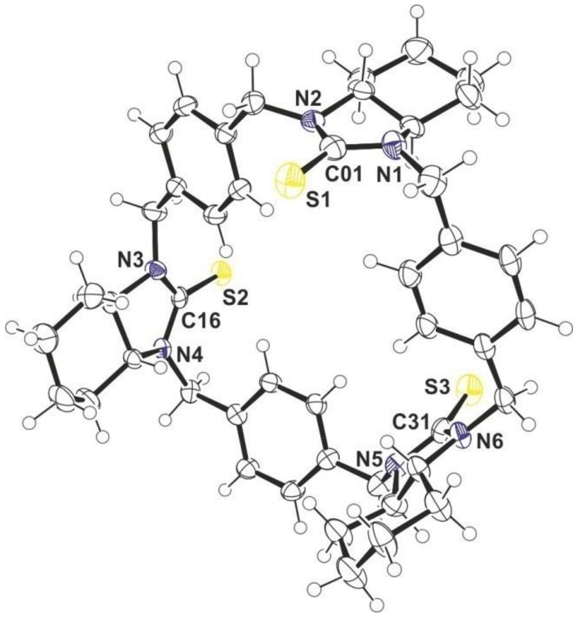

Figure S111. Structure of macrocycle **1b** as present in the crystal form II. Displacement ellipsoids are drawn at the 50% probability level. For clarity, only selected heteroatoms are labeled. Hydrogen atoms are represented in arbitrary radii.

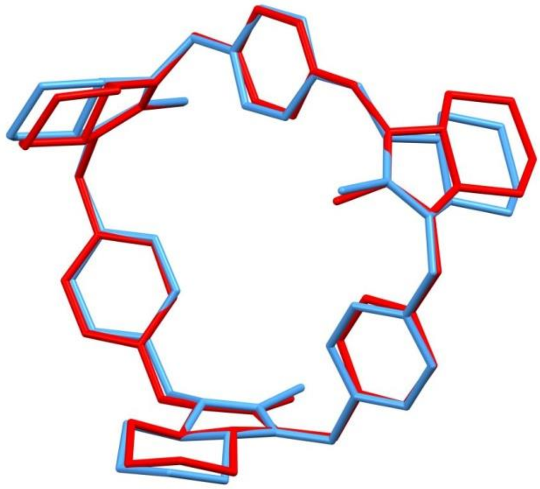

Figure S112. Overlaid molecular structures of macrocycle **1b** from **1b_I** (red) [10] and **1b_II** (blue) in the crystal phase. Hydrogen atoms are omitted for clarity.

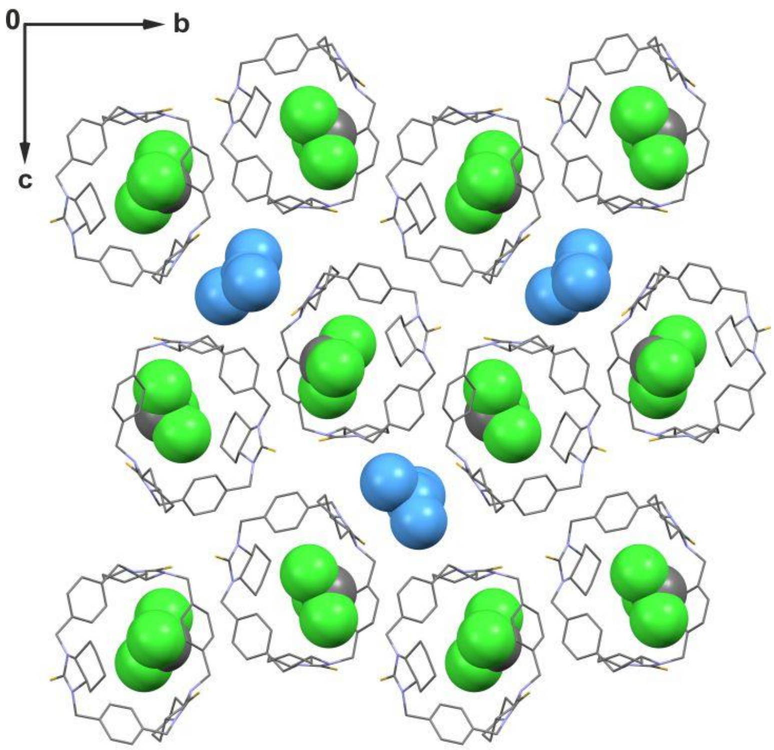

Figure S113. Packing diagram of host molecules of **1b_II** displayed as sticks and guest molecules displayed as spacefills. Modelled, but disordered chloroform molecules are presented in one arbitrarily selected position. For illustration, disordered solvent molecules, which their unresolved electron density was treated with PLATON/SQUEEZE, were presented in blue color. Hydrogen atoms molecules are omitted for clarity. View along [100] direction.

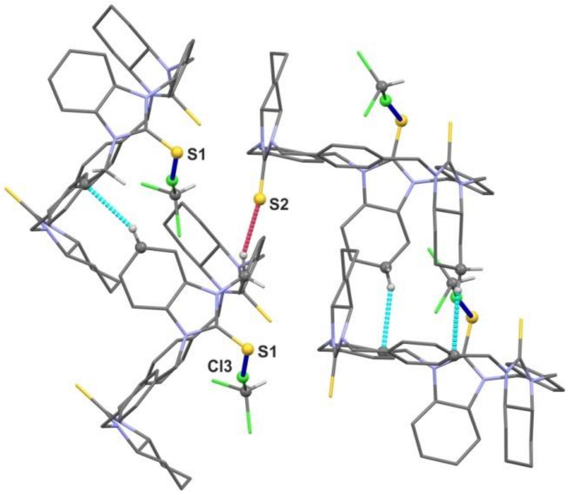

Figure S114. Supramolecular assembly in the crystal **1b_II**. Selected hydrogen bonds are marked in pink (C-H···S) and blue (C-H···C) dash lines. Halogen bonds are marked in dark-blue (C-Cl···S) lines.

Structure of thiourea-derived macrocycle 3 in the crystal phase

Colorless single crystal of **3** suitable for X-ray analysis was obtained by slow diffusion of diethyl ether vapors to chloroform solution. Compound **3** crystallizes in triclinic system in P_1 space group with 2 macrocyclic molecules and 5 chloroform molecules in unit cell.

Chloroform molecules fill voids created between host molecules. More precisely, two ordered solvent molecules are involved in intermolecular interactions that stabilize the host–guest structure. The remaining guest molecules, located in structural channels, were highly disordered and could not be resolved to yield a satisfactory model, therefore their unresolved electron density was treated with PLATON/SQUEEZE.[26] The estimated electron count is 174 in an accessible void volume of 507 Å^3 and can indicate to squeezed of 3 molecule of chloroform per unit cell.

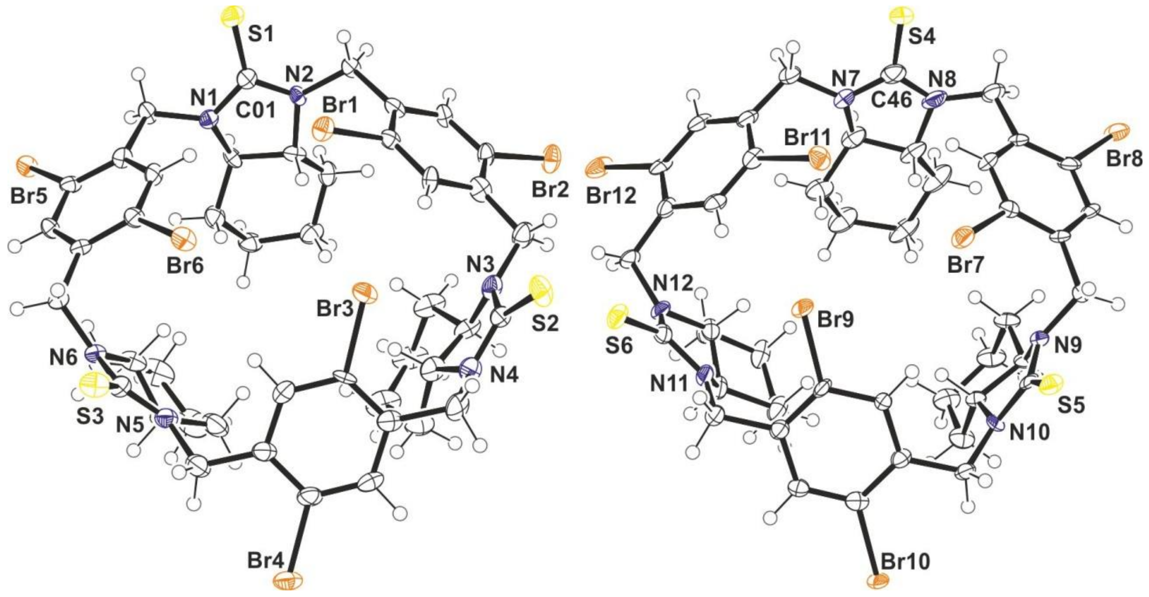

Figure S115. Structures of asymmetry independent molecules of macrocycle **3** in the crystal. Displacement ellipsoids are drawn at the 50% probability level. For clarity, only selected heteroatoms are labeled. Hydrogen atoms are represented in arbitrary radii.

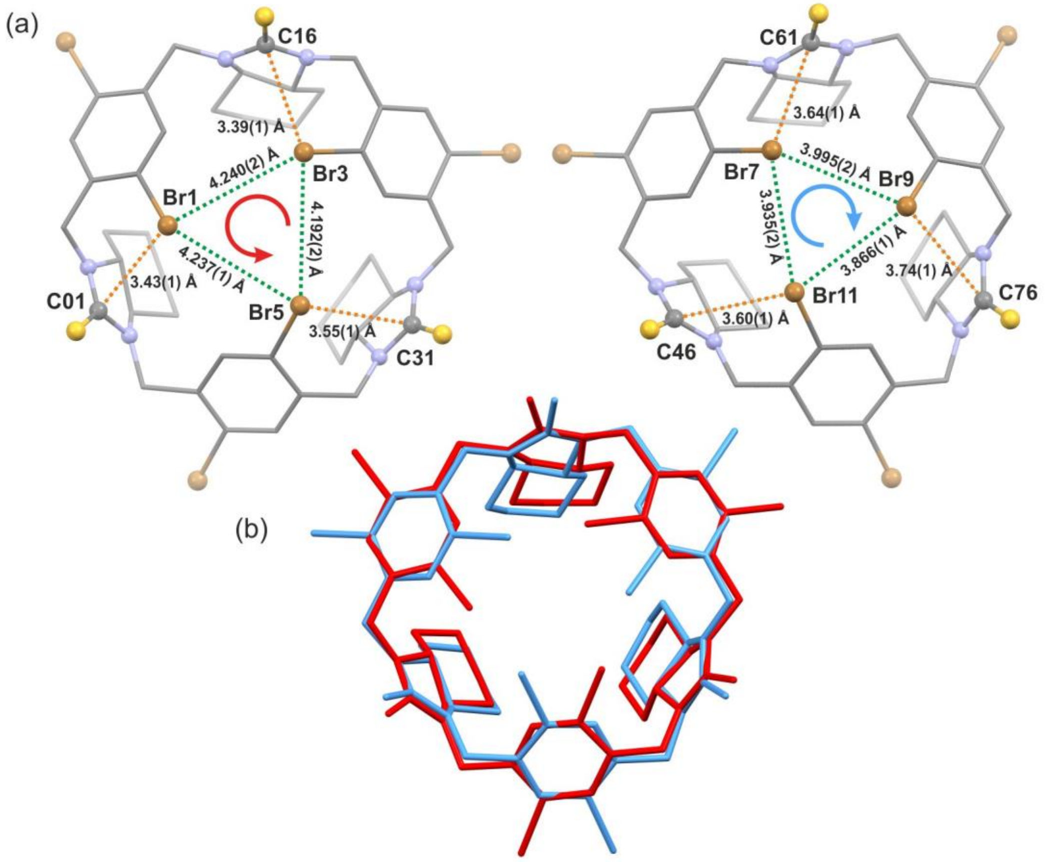

Figure S116. (a) Homochiral isomers of **3** with different helical arrangement of bromine atoms (views on the top of molecules). Helical arrangement of bromine atoms is distinguished by red and blue arrows. Short dipole–dipole contacts between bromine atoms are marked in green, and between carbon and bromine atoms – in orange dashed lines. (b) Overlaid molecular structures of diastereoisomers of **3**. Isomers are distinguished in blue and red colors. Hydrogen atoms are omitted for clarity.

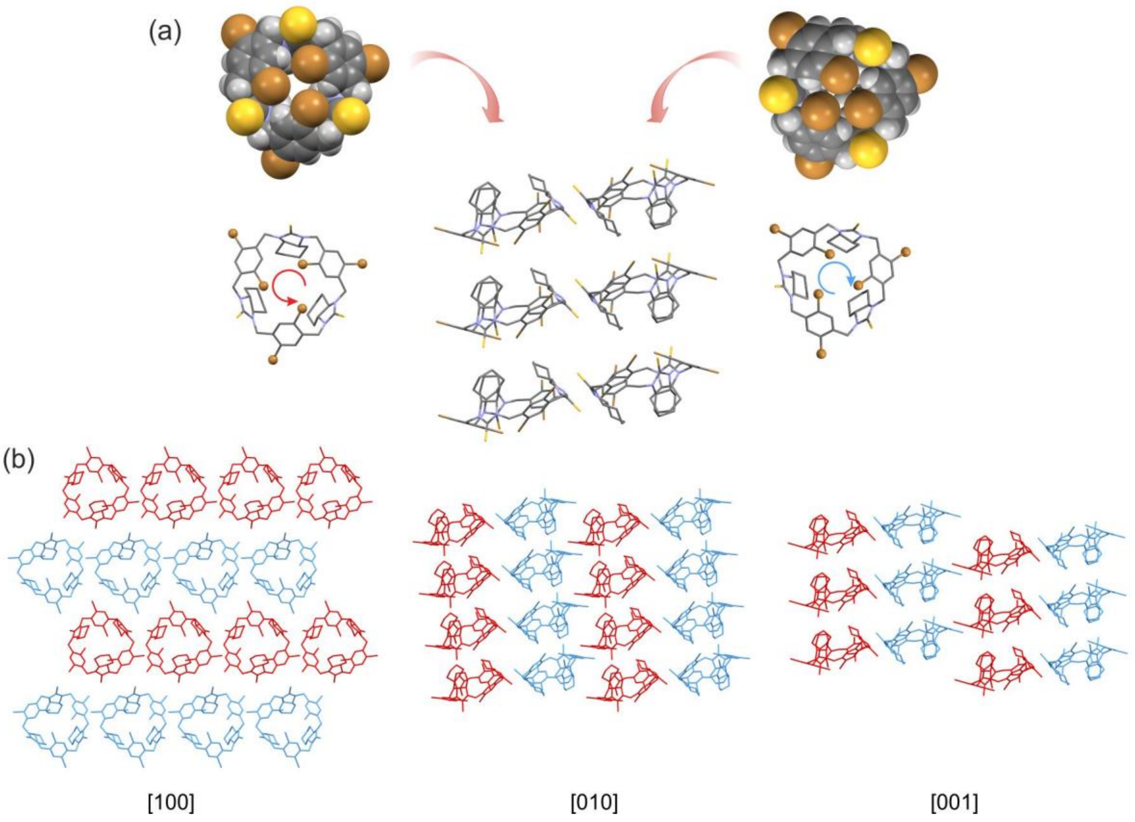

Figure S117. (a) Two diastereoisomers of **3** – the arrows indicate bromine atoms' "twisting" directions (views on the top of molecules). (b) Packing diagrams of host molecules displayed as sticks. Isomers have been marked with different colors. Views along [100], [010] and [001] directions. Hydrogen atoms are omitted for clarity.

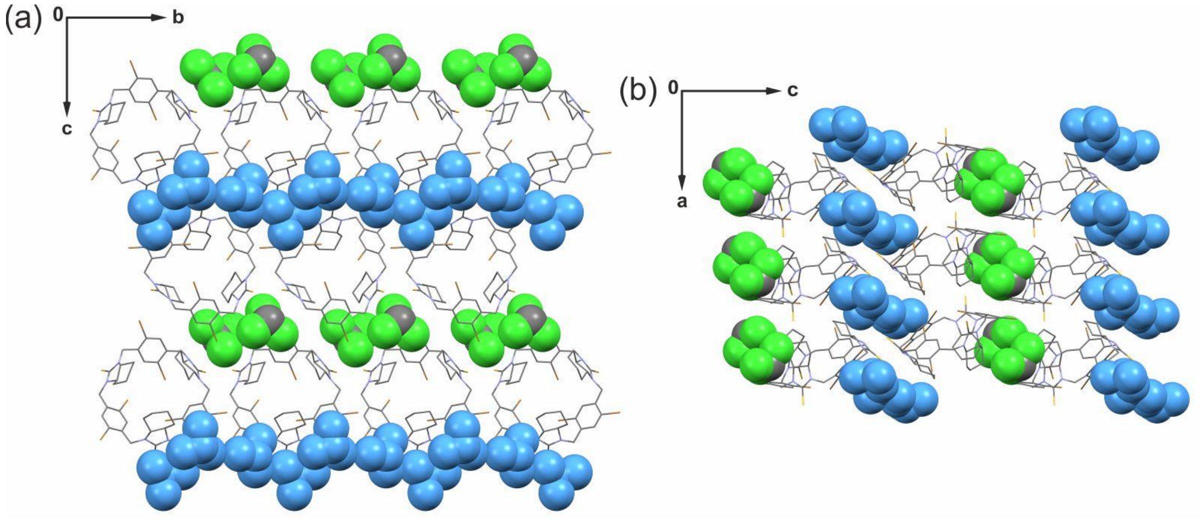

Figure S118. Packing diagrams of host molecules of 3b displayed as sticks and guest molecules displayed as spacefills. For illustration, disordered solvent molecules, which their unresolved electron density was treated with PLATON/SQUEEZE, were presented in blue color. Hydrogen atoms are omitted for clarity. Views along (a) [100] and (b) [010] directions.

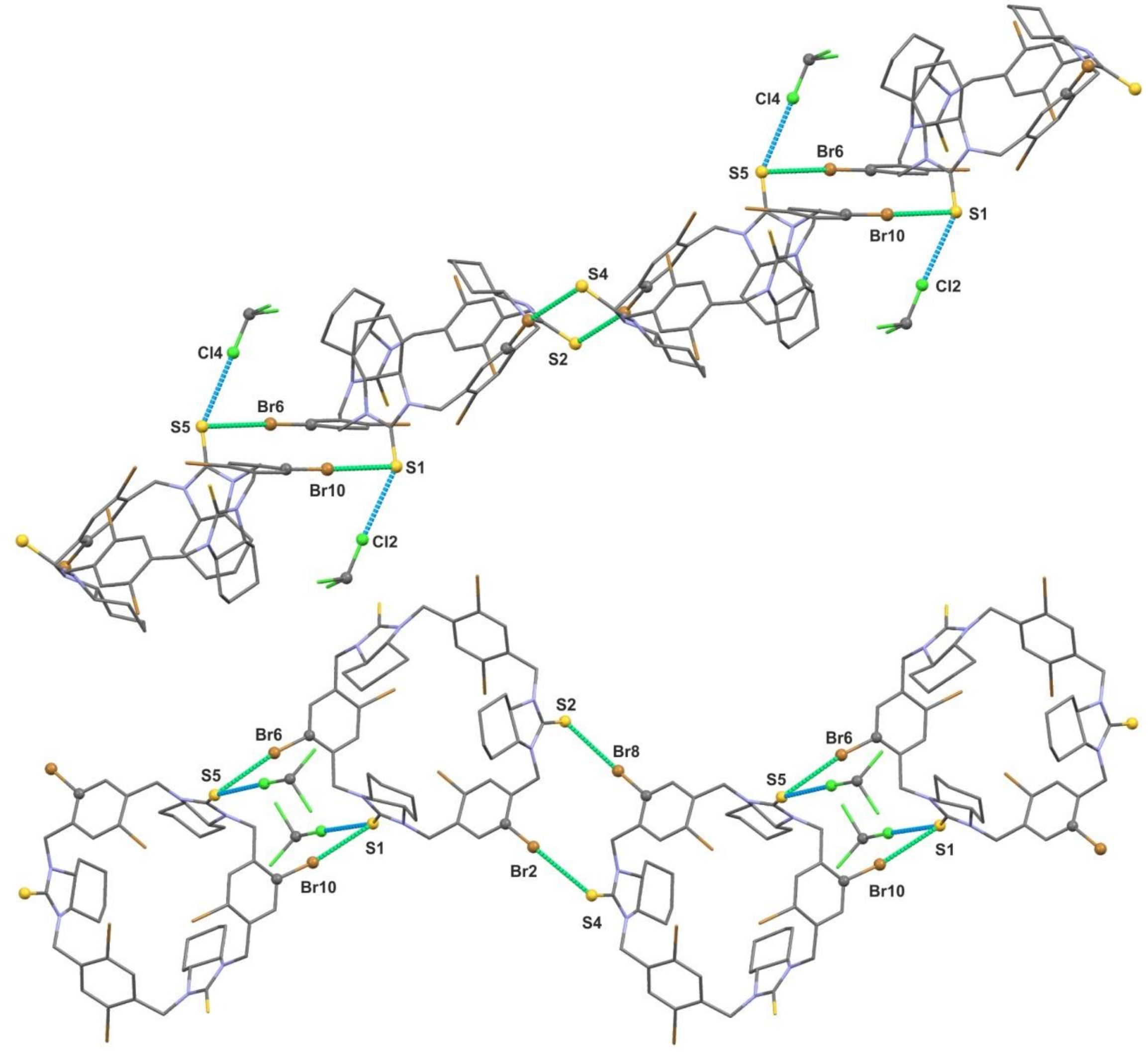

Figure S119. Supramolecular chain created in crystal **3**. Halogen bonds are marked in blue (C-Cl···S) and green (C-Br···S).

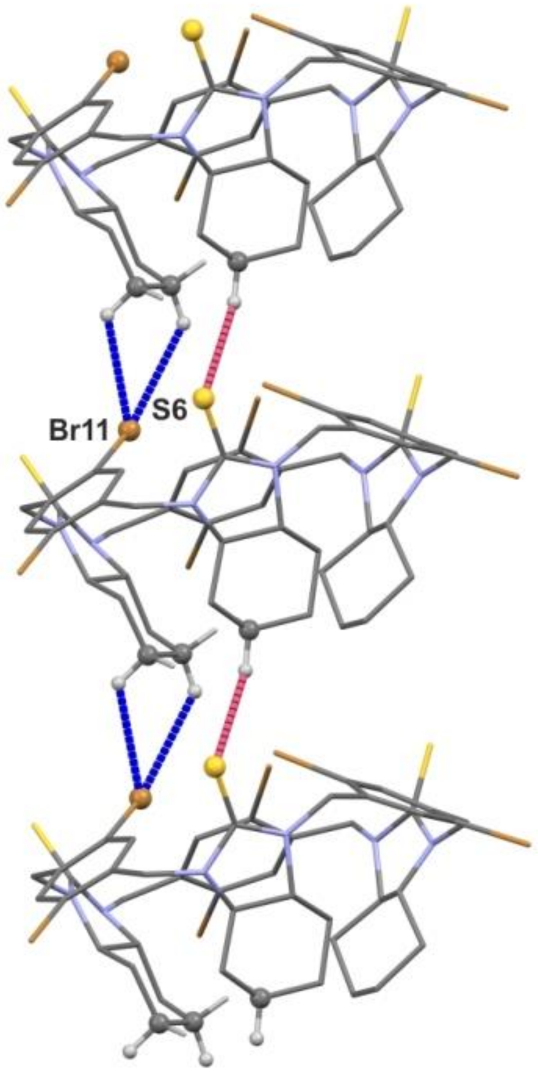

Figure S120. Supramolecular columnar assembly created in the crystal of **3**. Hydrogen bonds are marked in dark-blue (C-H···Br) and pink (C-H···S).

Structure of thiourea-derived macrocycle 8b in the crystal phase

Colorless single crystal of **8b** suitable for X-ray analysis was obtained by slow diffusion of diethyl ether vapors to chloroform solution.

Compound **8b** crystallizes in orthorhombic system in $P2_12_12_1$ space group with 4 macrocyclic molecules and 4 chloroform molecules in unit cell.

Chloroform molecules fill voids between host macrocyclic molecules, and are involved in intermolecular interactions that stabilize the host–guest structure.

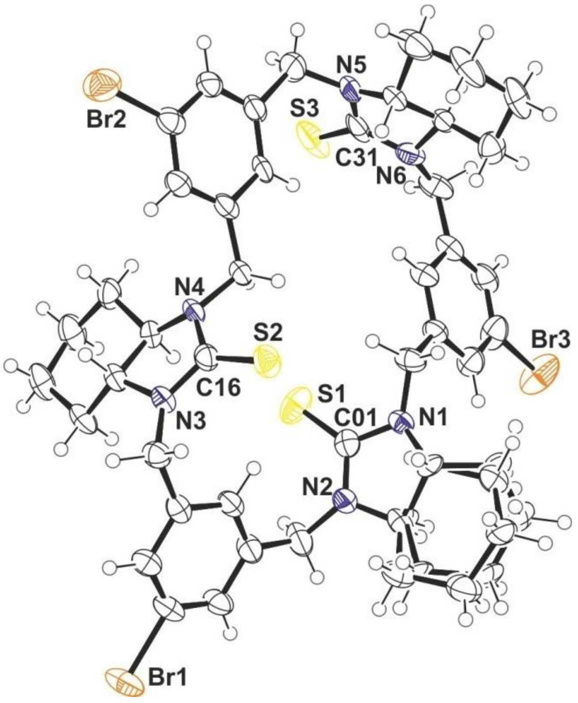

Figure S121. Structure of macrocycle **8b** in the crystal. Displacement ellipsoids are drawn at the 50% probability level. For clarity, only selected heteroatoms are labeled. Hydrogen atoms are represented in arbitrary radii. The ratio of site occupation factors for two components of the disordered cyclohexane group, is 50% : 50%.

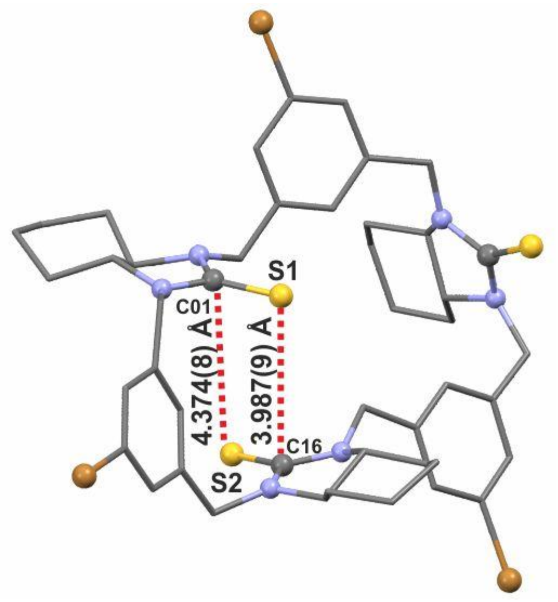

Figure S122. Arrangement of molecule **8b** in the crystal and measured distances between thiourea groups: S1···C16 and S2···C01 (marked in red dashed lines).

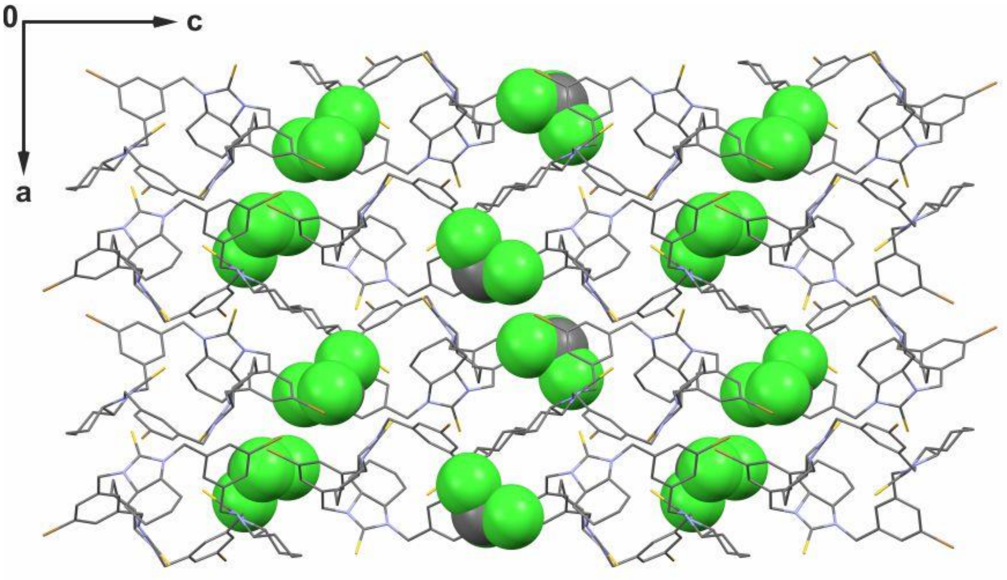

Figure S123. Packing diagram of host molecules of **8b** displayed as sticks and guest molecules displayed as spacefills. Hydrogen atoms are omitted for clarity. View along [010] direction.

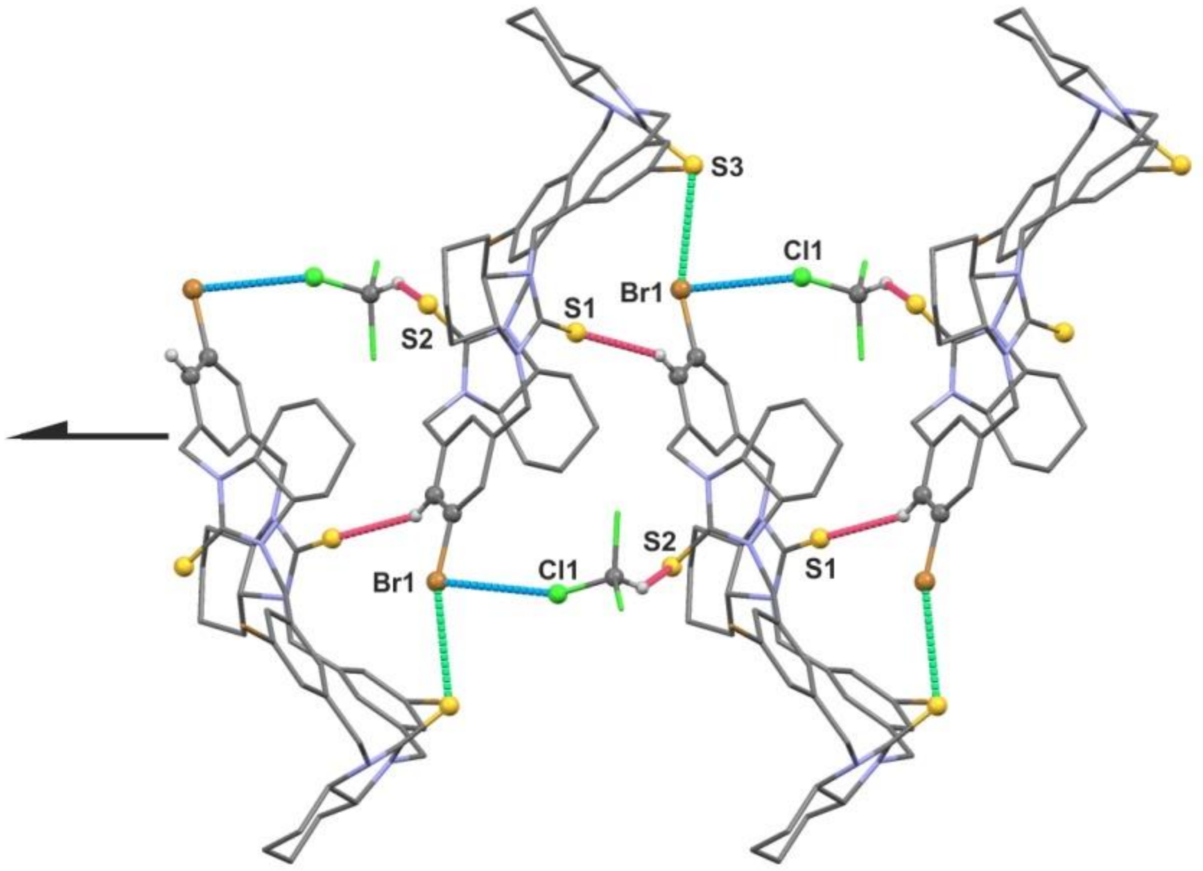

Figure S124. Supramolecular chain created around 2_1 screw axis in the crystal of **8b**. Selected hydrogen bonds are marked in pink (C-H···S); and halogen bonds – in blue (C-Cl···Br) and green (C-Br···S) lines. View along [010] direction.

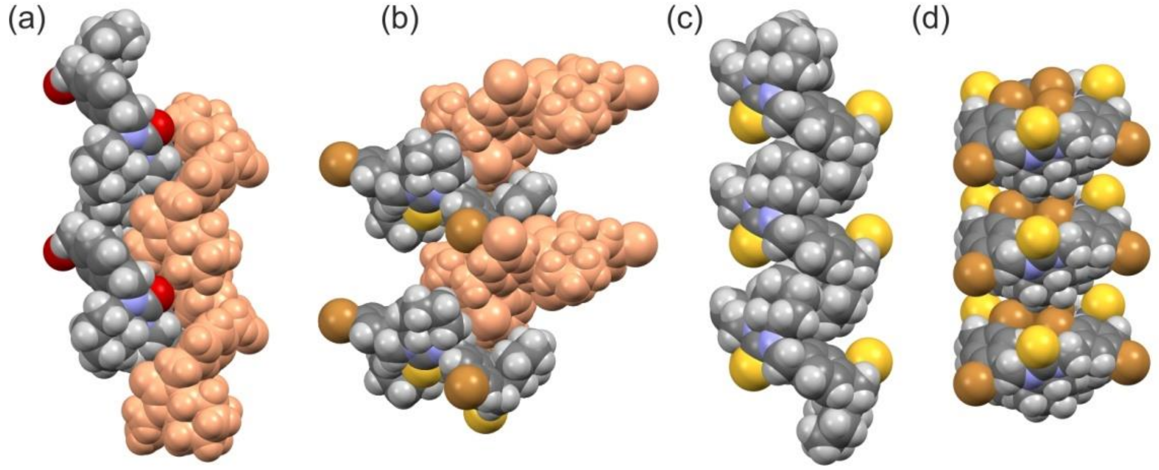

Figure S125. Various of columnar systems of host macrocyclic molecules in the crystals: a zipper motif created by two columnar stacks (distinguished in different colors) in (a) **1a_II** and (b) **8b**; a single columnar stack in (c) **1b_II** and (d) **3**. Macrocycles are drawn as van der Waals spheres.

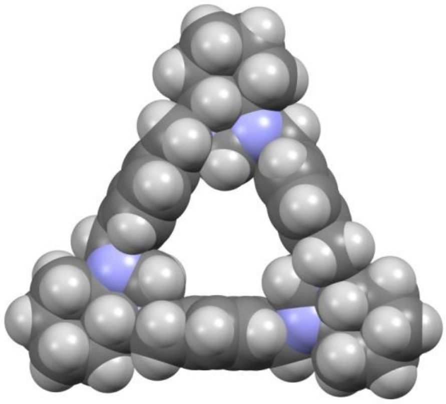

Figure S126. Structure of bridged trianglimine in the crystal.[31] Macrocycle is drawn with van der Waals spheres.

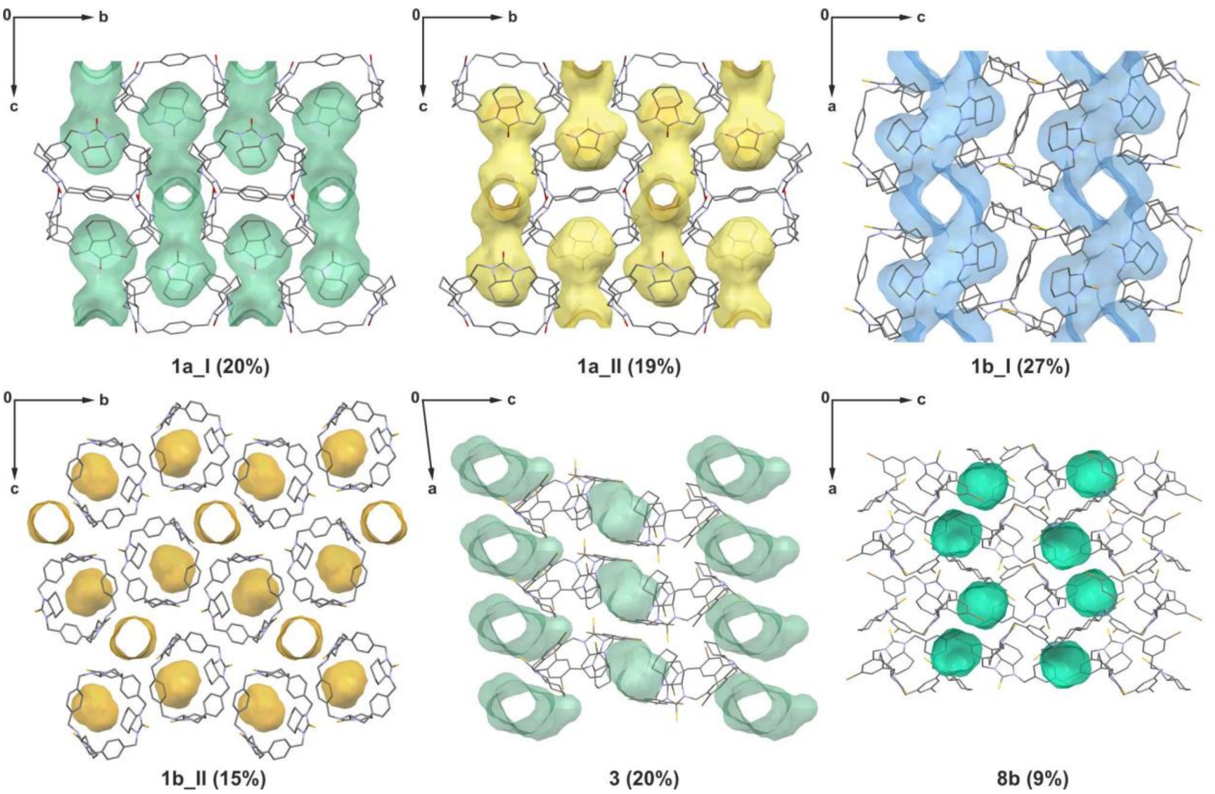

Figure S127. Various of structural voids and channels created by host macrocyclic molecules in crystals: **1a_I**, **1a_II**, **1b_I**,[10] **1b_II**, **3**, and **8b**. Estimated percentages of unit cell volume occupied by voids in unit cell are reported in parentheses. Probe radius: 1.5 Å. Host molecules are represented in sticks style; hydrogen atoms and solvent molecules are omitted for clarity.

Hirshfeld surface analysis

Hirshfeld surface analysis was carried out using CrystalExplorer program.[30] Contact distances based on BR_{vdW} [32] were mapped on the Hirshfeld surfaces (d_{norm}). In the color scale, negative values of d_{norm} were visualized in red, indicating contacts shorter than ΣBR_{vdW}. Values represented in white denote intermolecular distances close to contacts with d_{norm} equal to zero. Contacts longer than ΣBR_{vdW} with positive d_{norm} values were shown in blue.

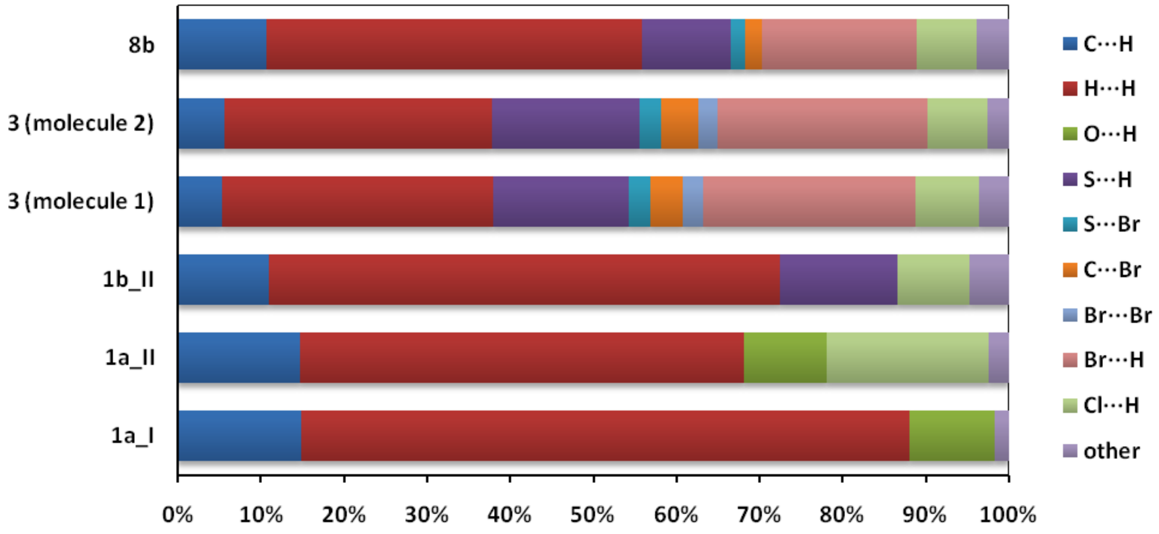

Figure S128. Percentage contributions of various intermolecular contacts to the molecular Hirshfeld surface of macrocycles **1a_I**, **1a_II**, **1b_II**, **3** and **8b**.

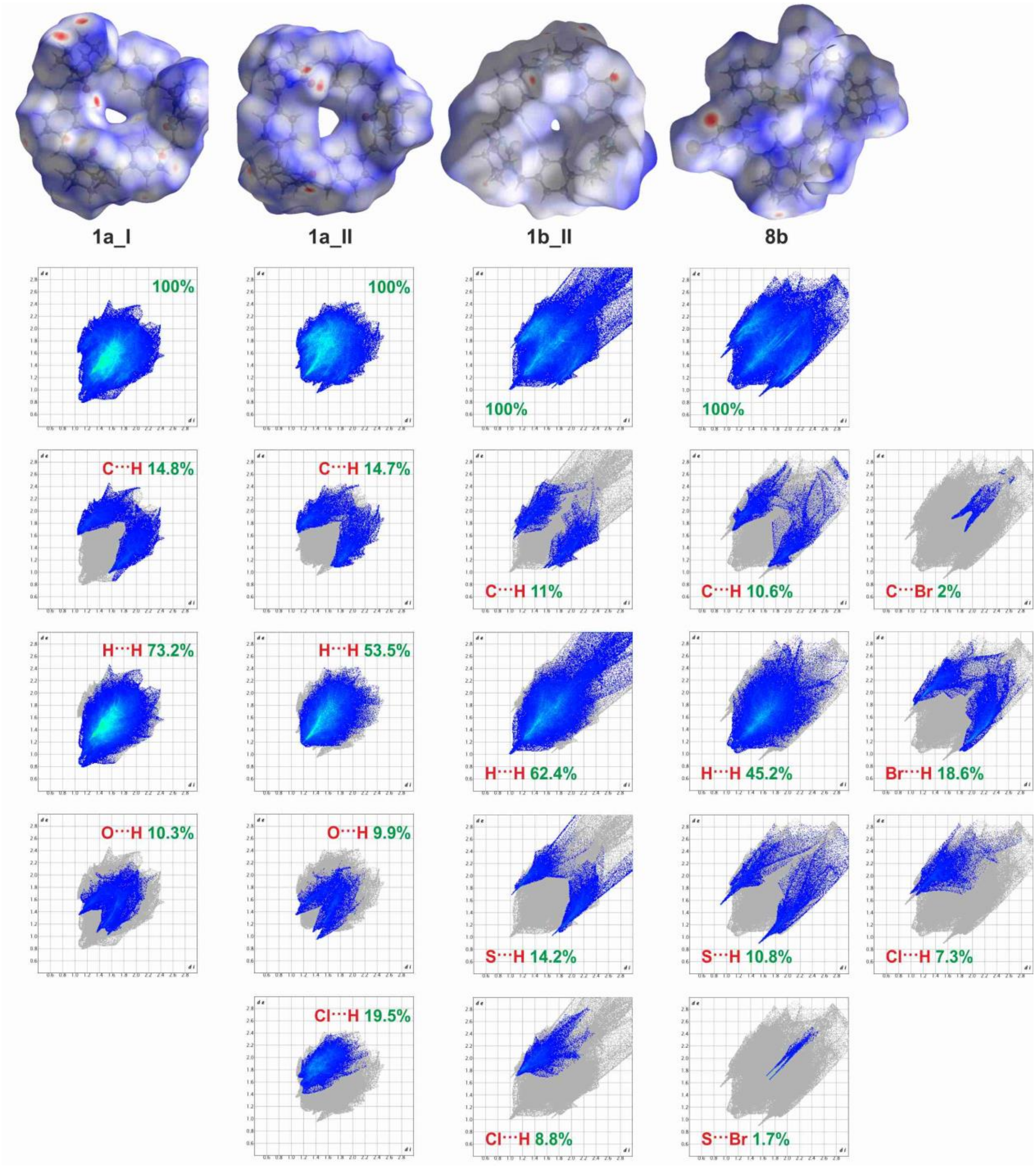

Figure S129. Results of Hirshfeld surface analysis (normalized Hirshfeld surfaces d_{norm} and selected fingerprint plots) for **1a_I**, **1a_II**, **1b_II** and **8b**.

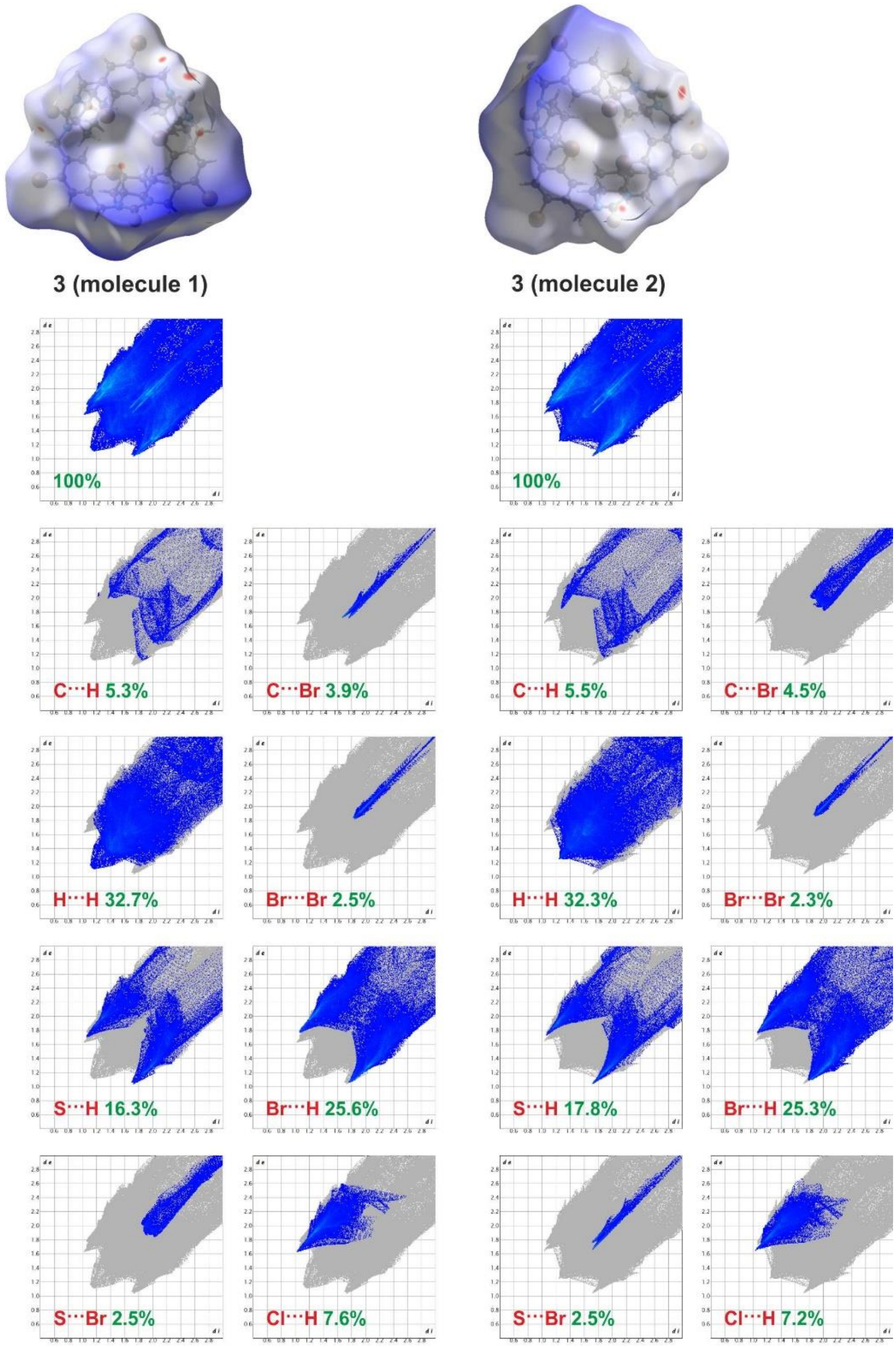

Figure S130. Results of Hirshfeld surface analysis (normalized Hirsfeld surfaces d_{norm} and selected fingerprint plots) for **3**.

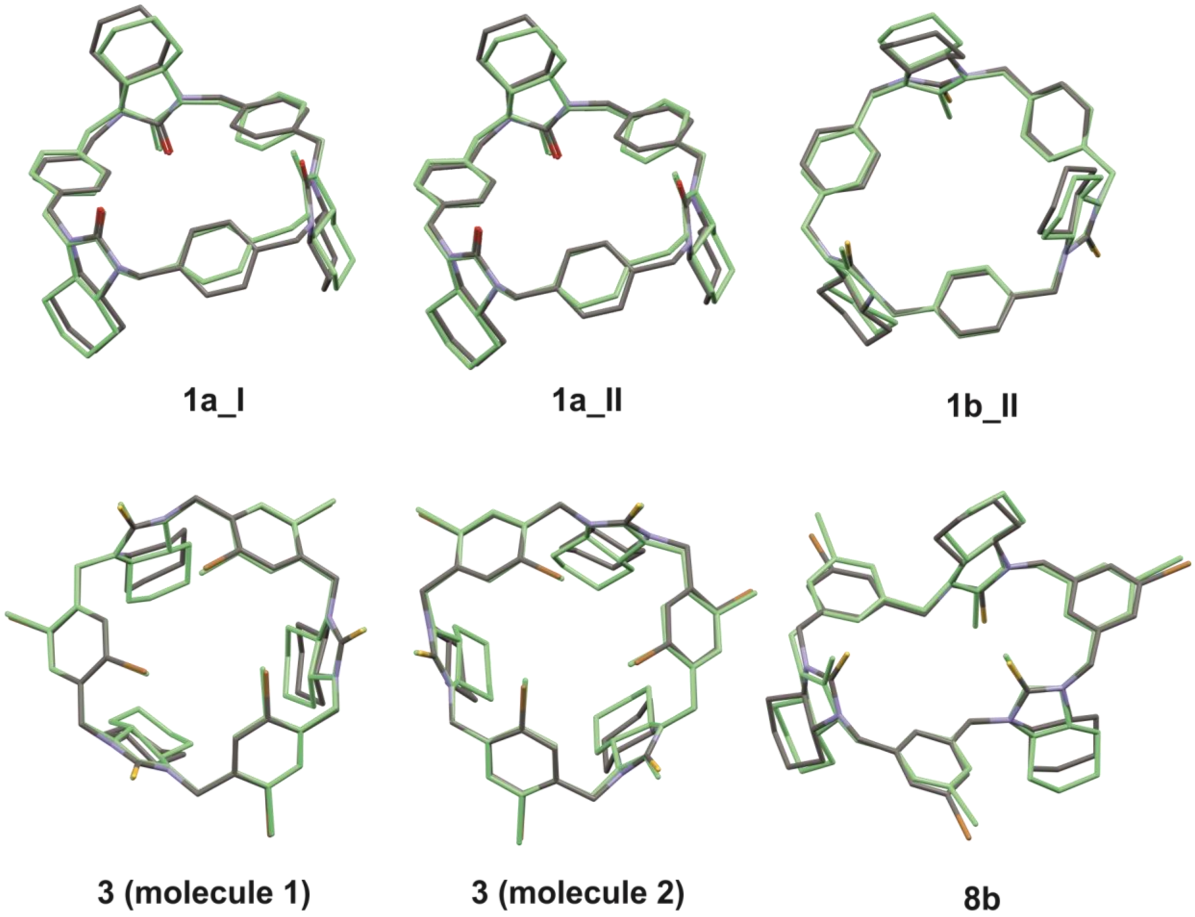

Figure S131. Overlaid molecular structures of urea and thiourea derivatives **1a**, **1b**, **3** and **8b**, found in the crystal phase (green color) and fully optimized at the B3LYP/6-311G(d,p) level. The optimization procedures were utilized starting geometries found in the respective crystals.

Copies of ^{1}H and ^{13}C NMR spectra

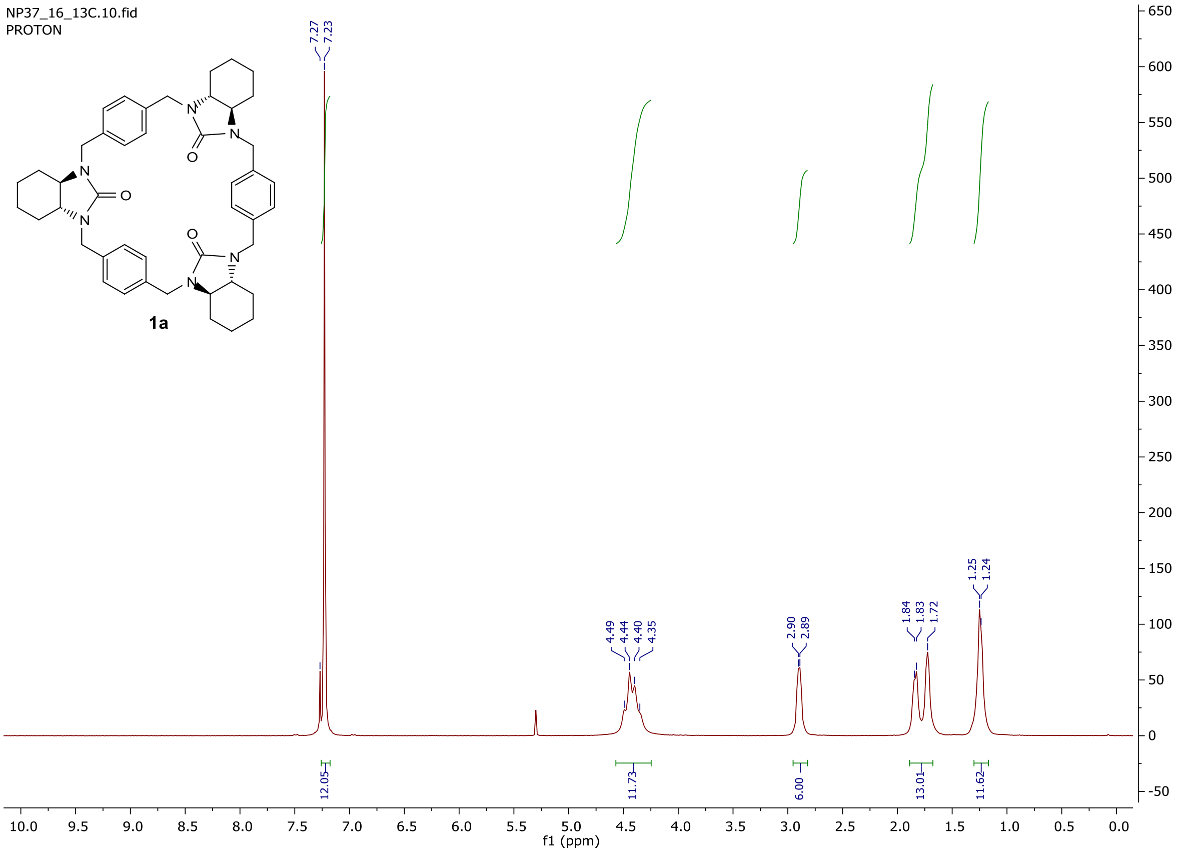

Copy of ^{1}H NMR spectrum ($CDCl_3$, 300 MHz, RT) of **1a**.

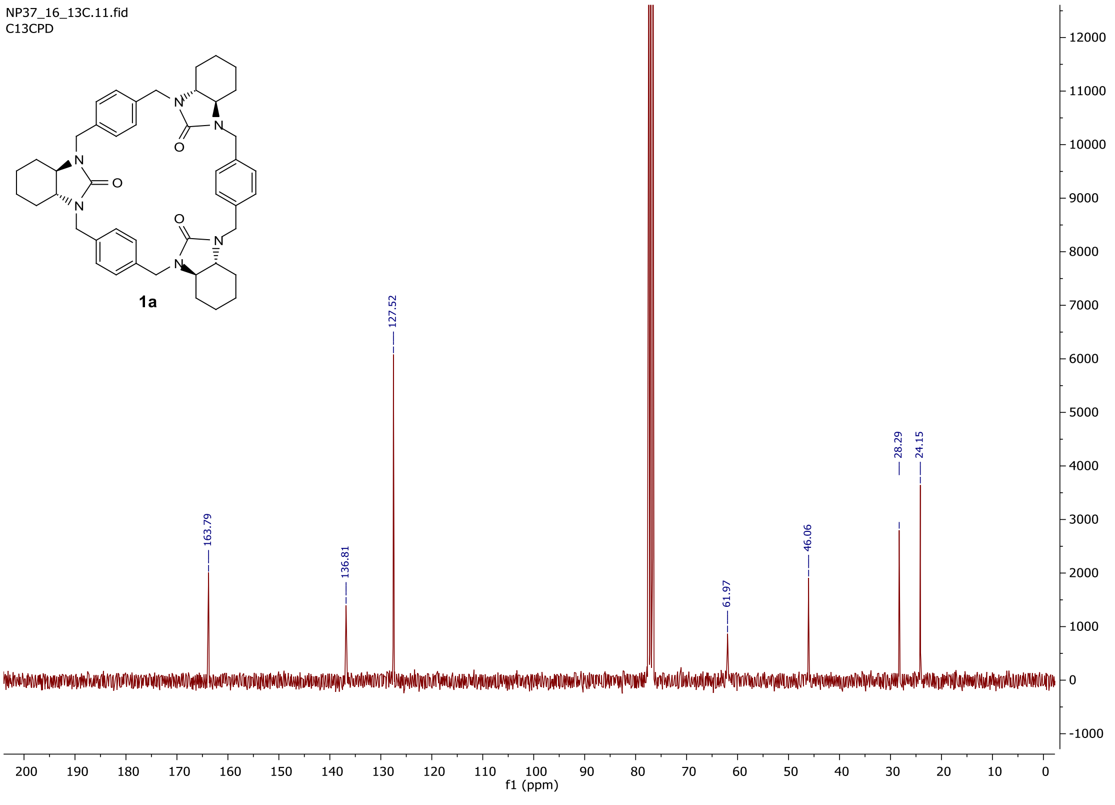

Copy of $^{13}C\{^{1}H\}$ NMR spectrum ($CDCl_3$, 300 MHz, RT) of **1a**.

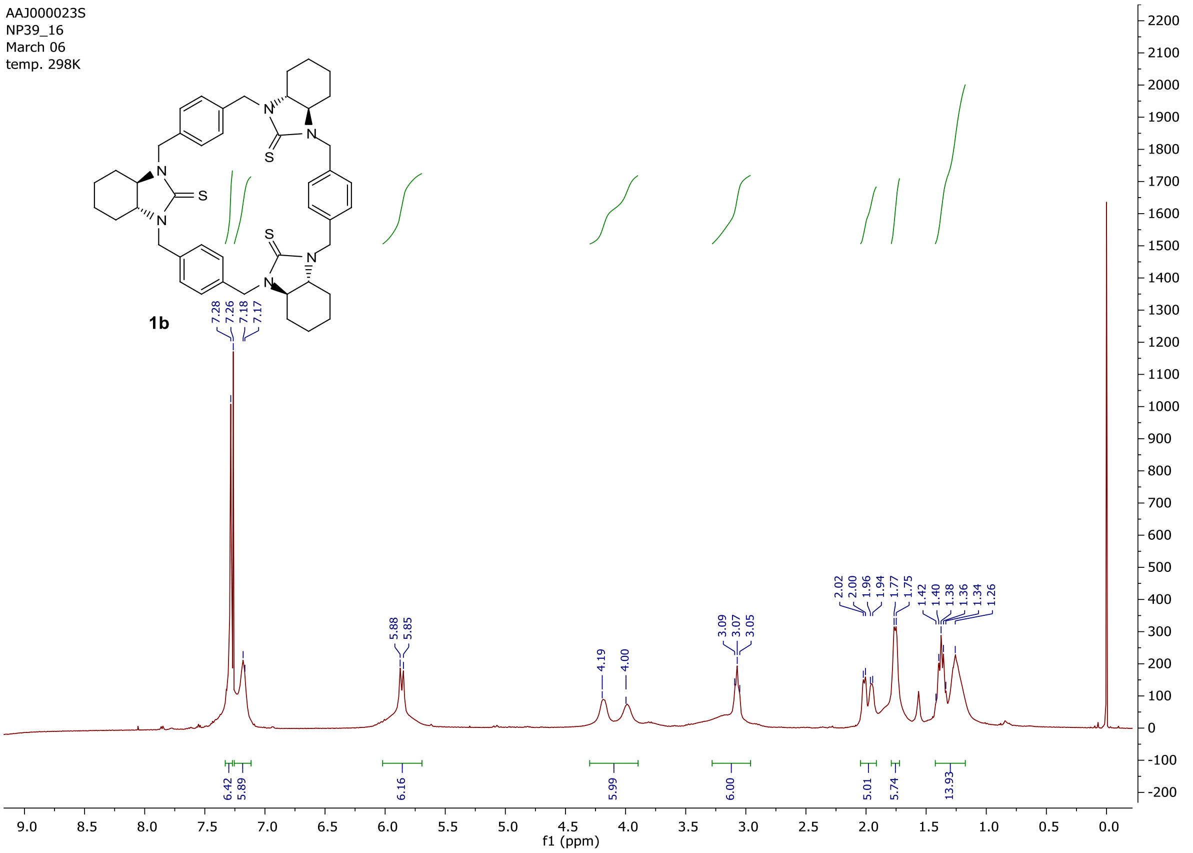

Copy of ^{1}H NMR spectrum ($CDCl_3$, 600 MHz, RT) of **1b**.

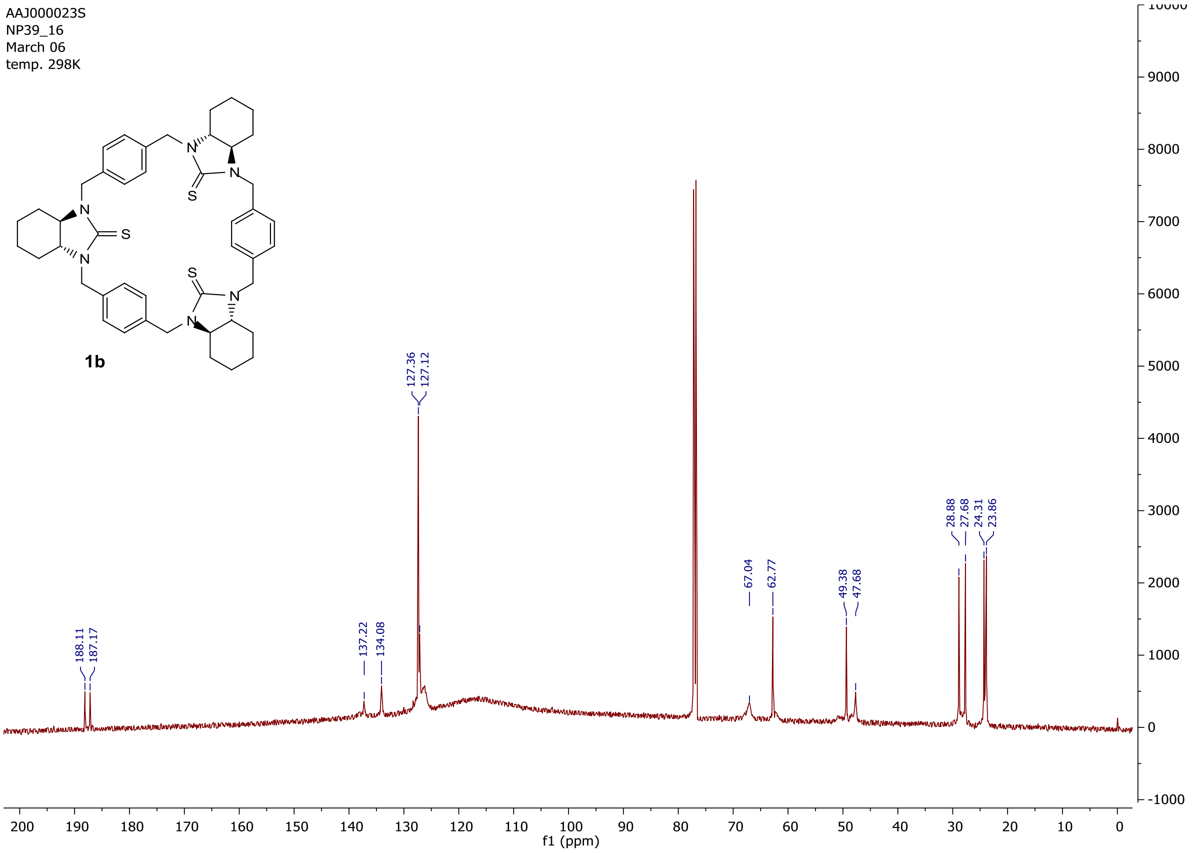

Copy of $^{13}C\{^{1}H\}$ NMR spectrum ($CDCl_3$, 600 MHz, RT) of **1b**.

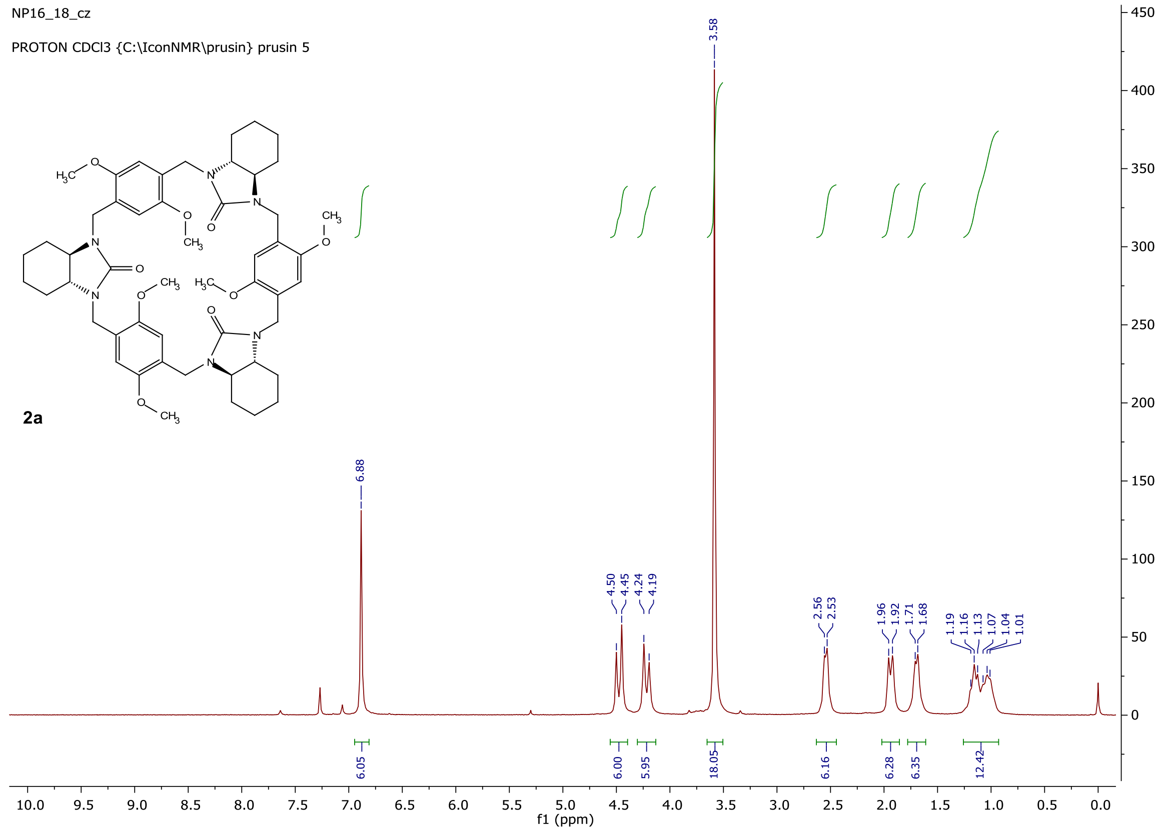

Copy of ^{1}H NMR spectrum ($CDCl_3$, 300 MHz, RT) of **2a**.

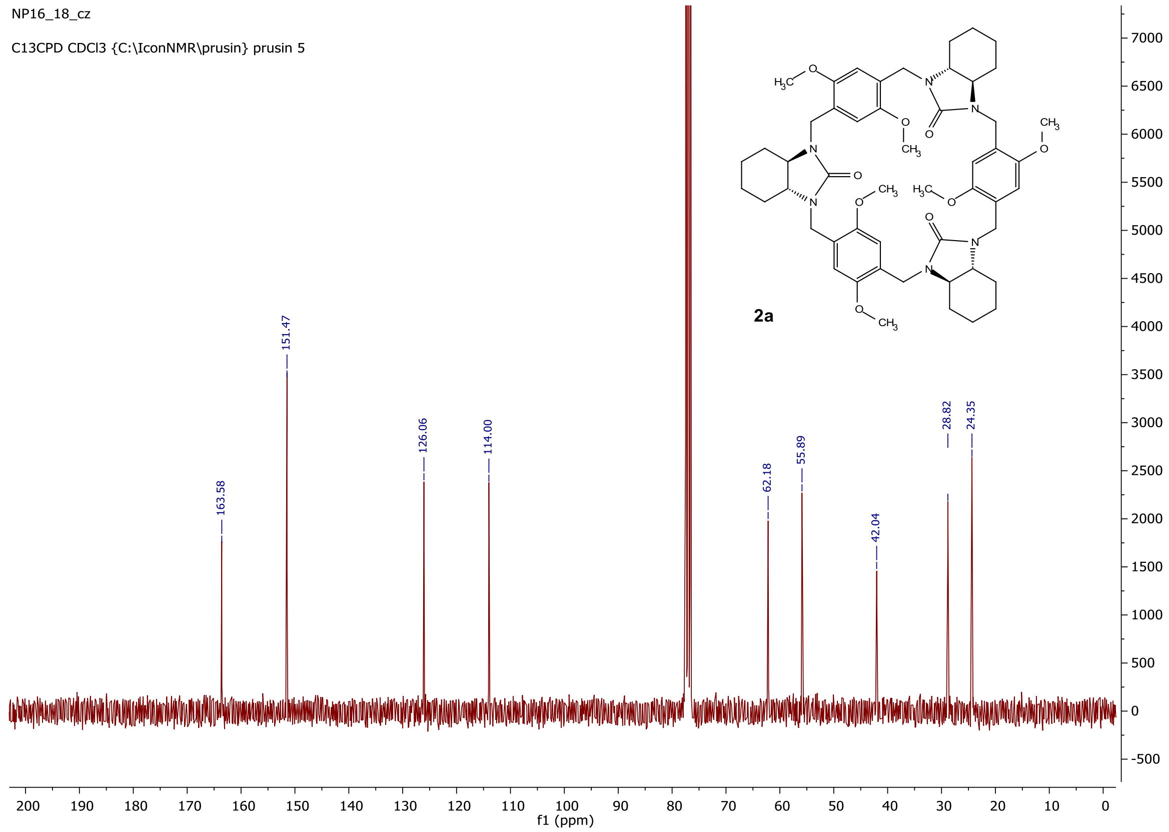

Copy of $^{13}C\{^{1}H\}$ NMR spectrum ($CDCl_3$, 300 MHz, RT) of **2a**.

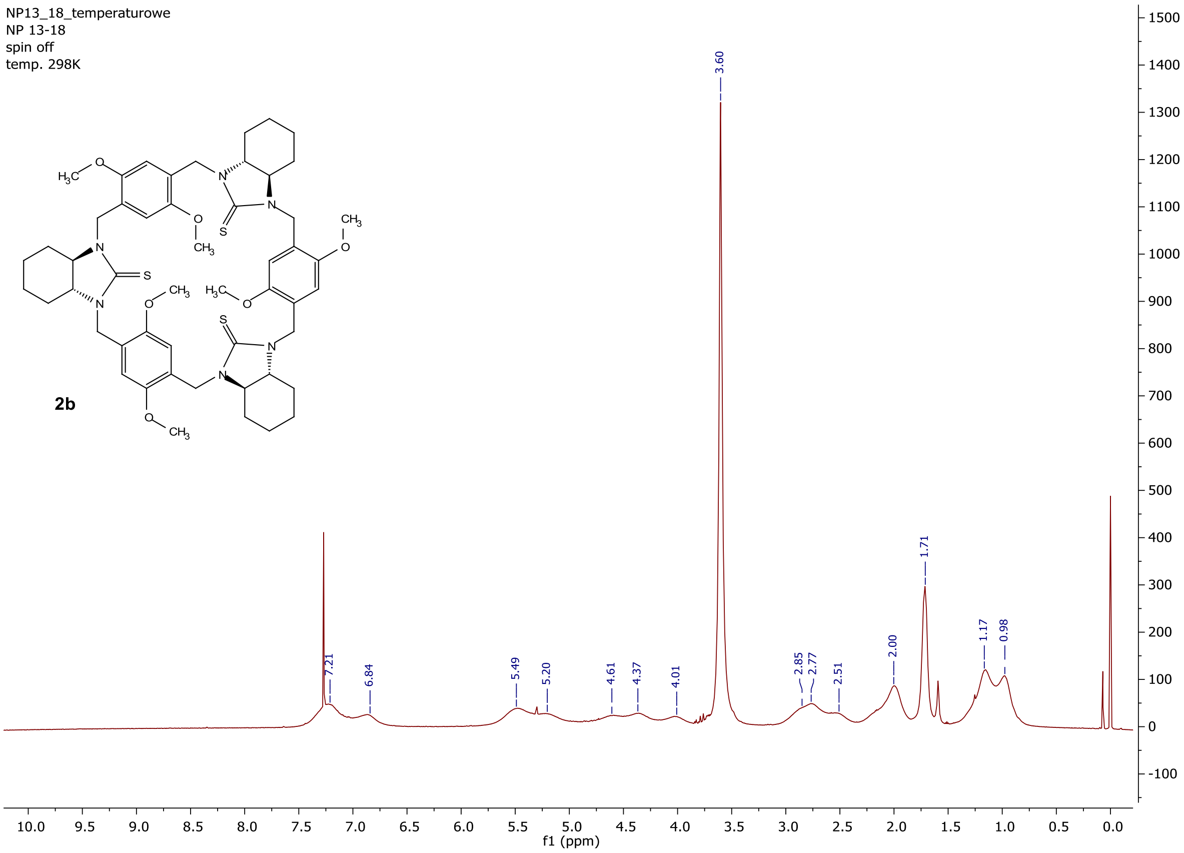

Copy of ^{1}H NMR spectrum ($CDCl_3$, 600 MHz, RT) of **2b**.

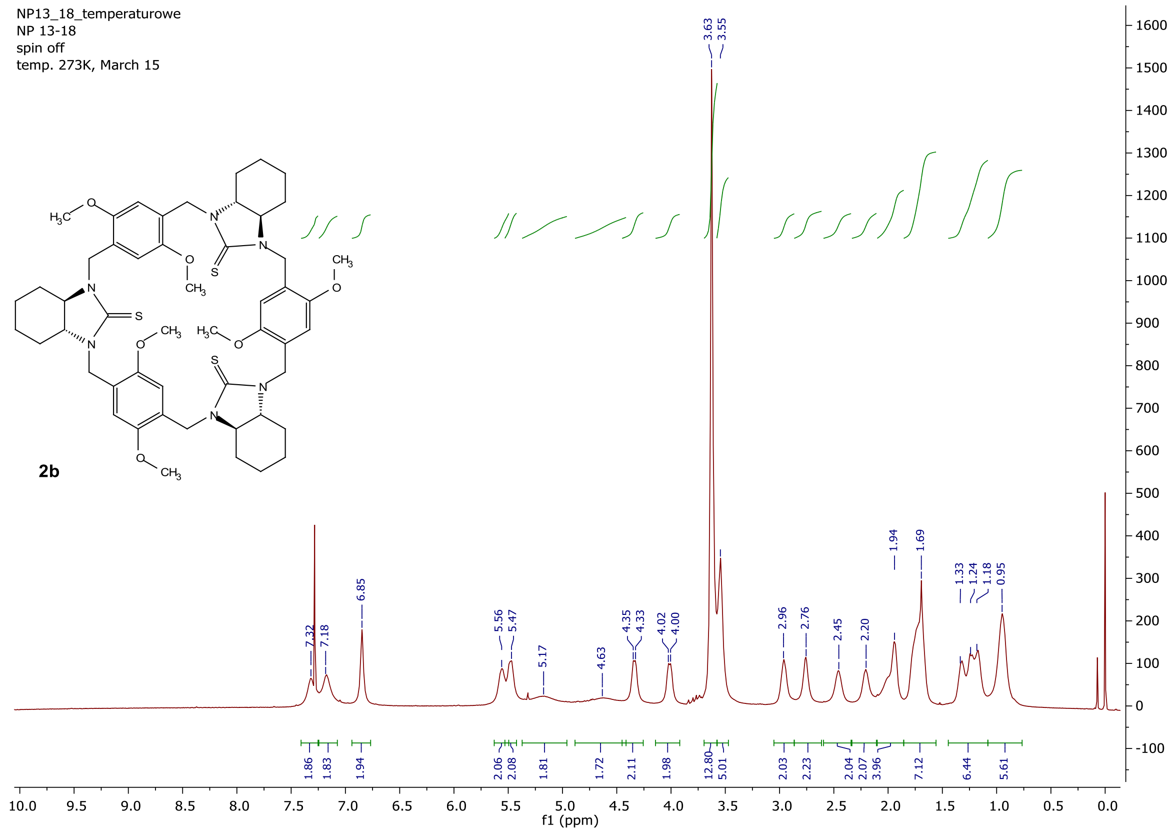

Copy of ^{1}H NMR spectrum ($CDCl_3$, 600 MHz, 0 °C) of **2b**.

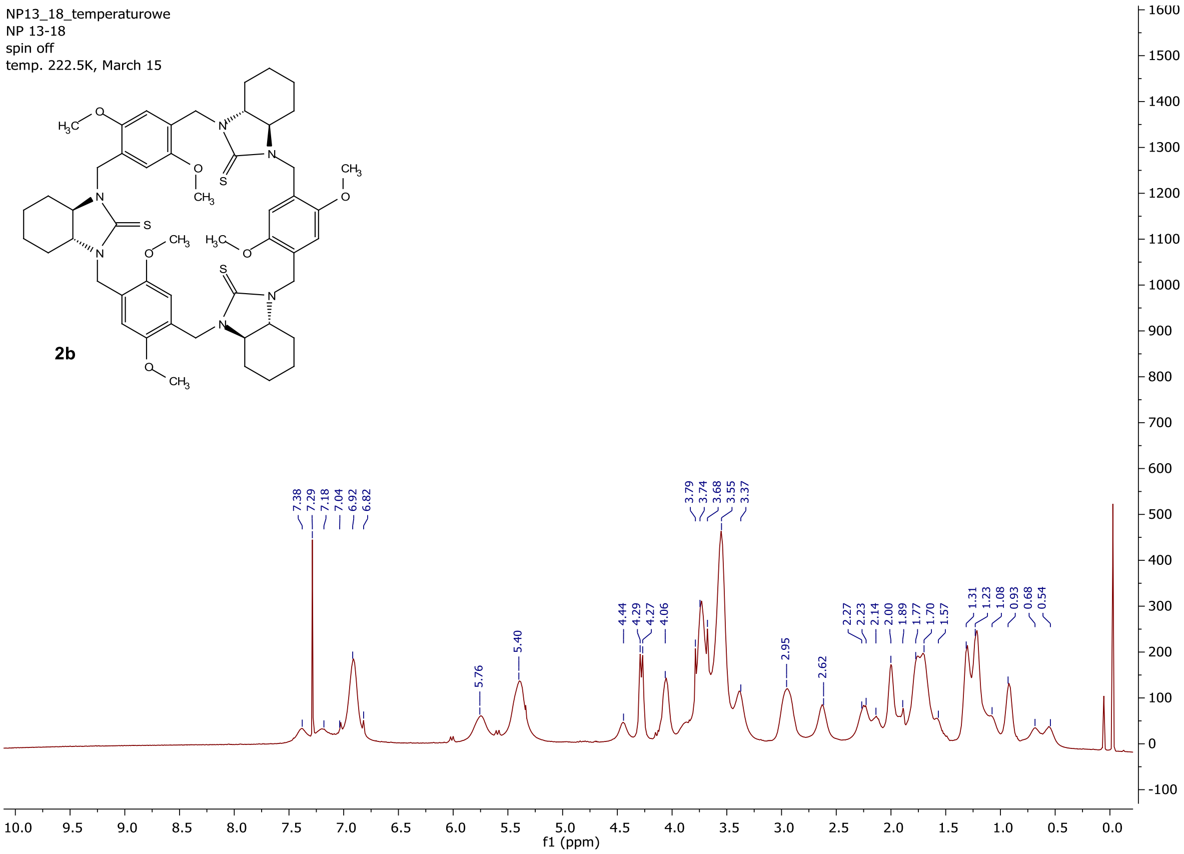

Copy of ^{1}H NMR spectrum ($CDCl_3$, 600 MHz, –50 °C) of **2b**.

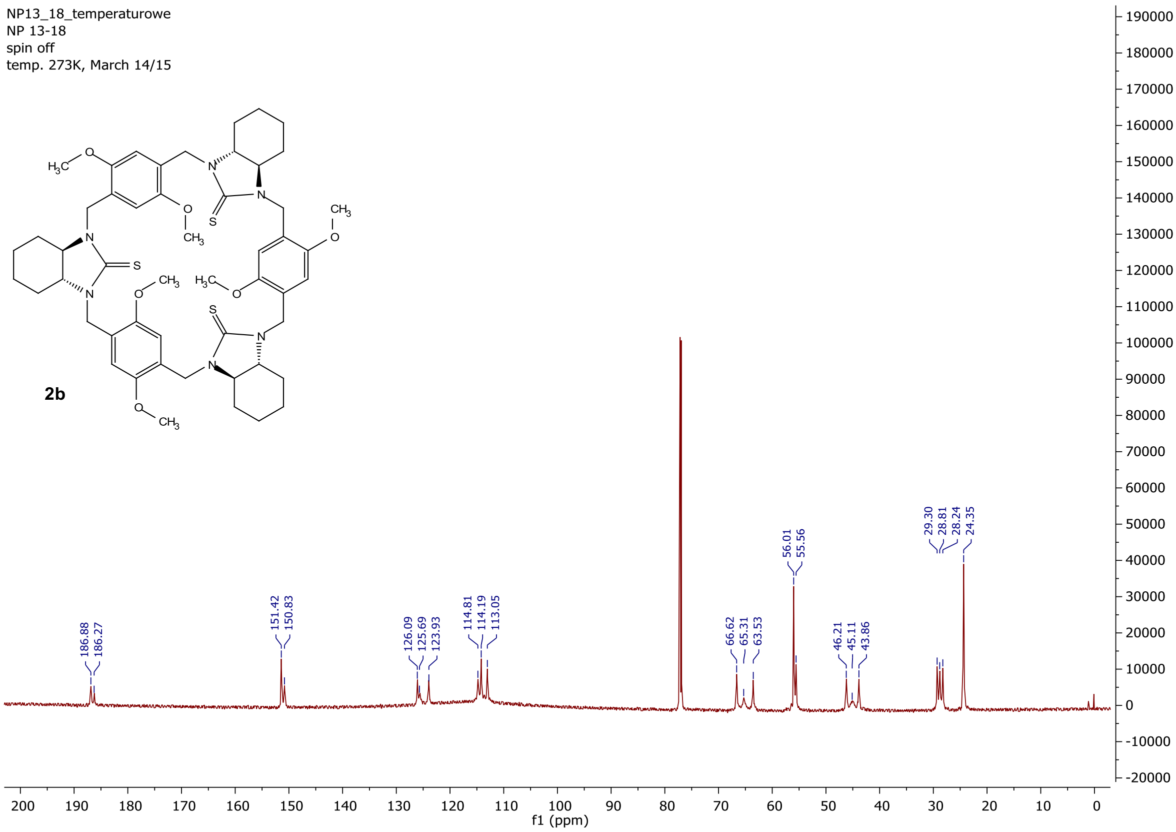

Copy of $^{13}C\{^{1}H\}$ NMR spectrum ($CDCl_3$, 600 MHz, 0 °C) of **2b**.

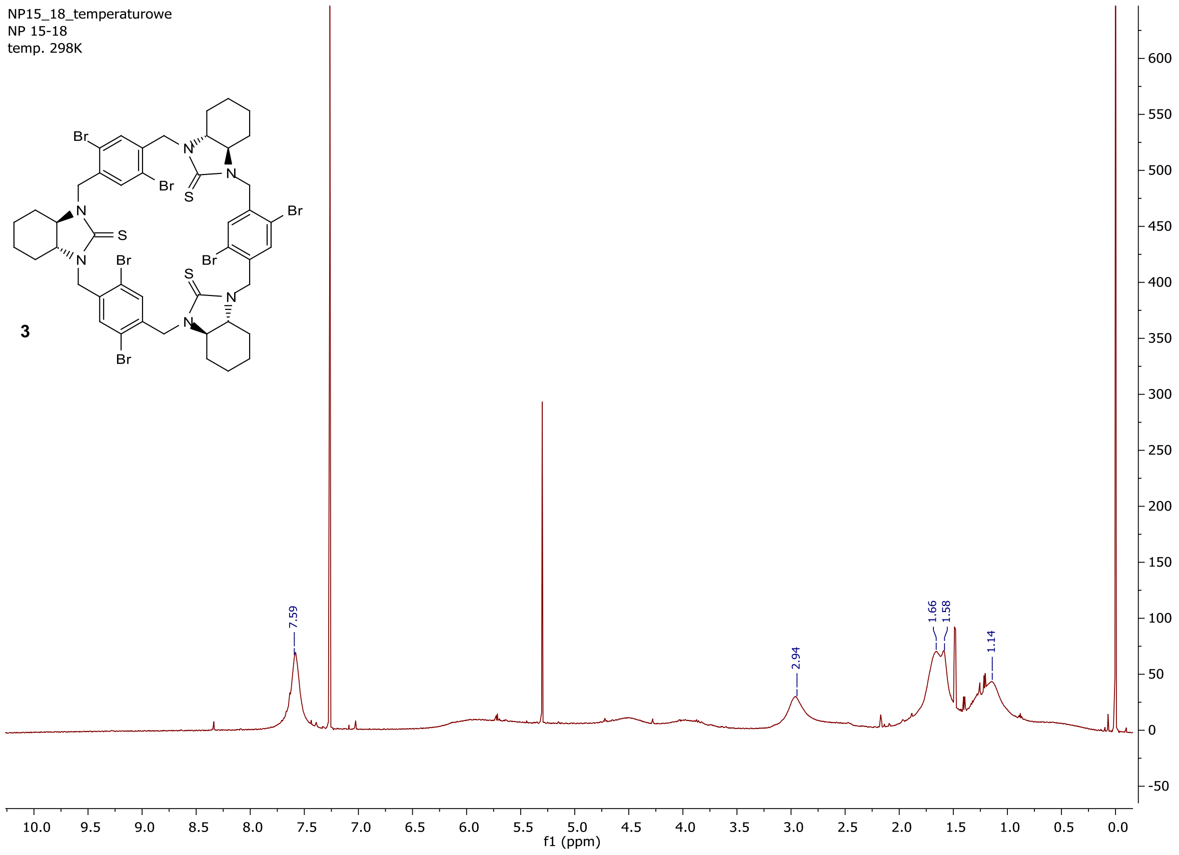

Copy of ^{1}H NMR spectrum ($CDCl_3$, 600 MHz, RT) of **3**.

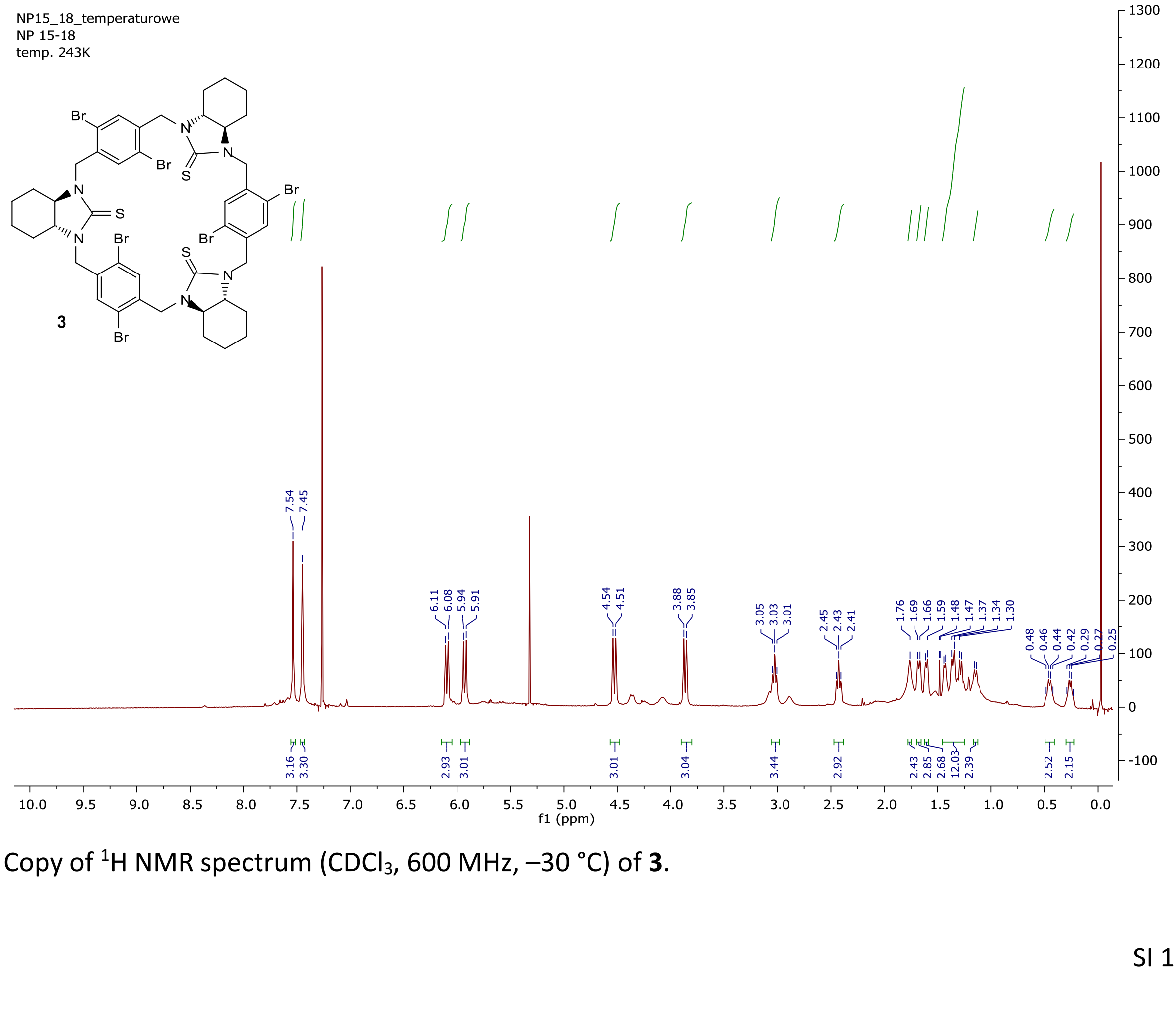

Copy of ^{1}H NMR spectrum ($CDCl_3$, 600 MHz, –30 °C) of **3**.

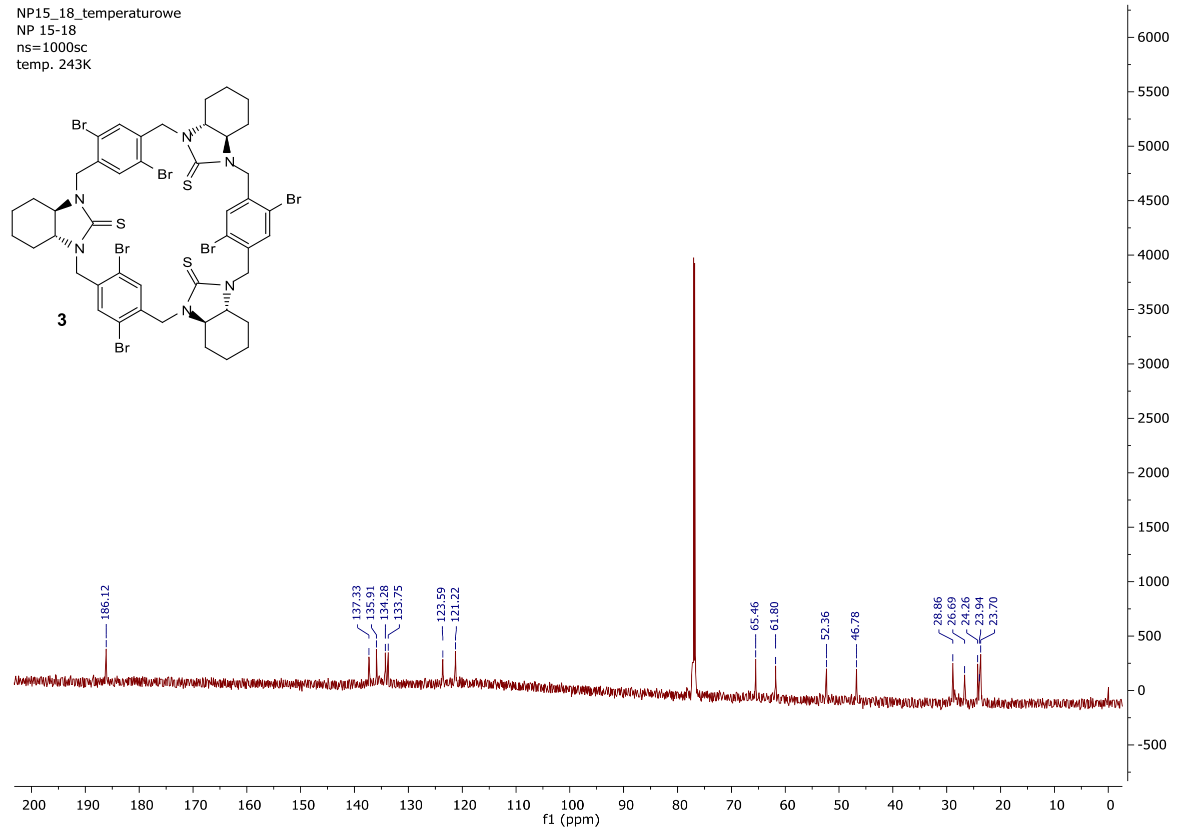

Copy of $^{13}C\{^{1}H\}$ NMR spectrum ($CDCl_3$, 600 MHz, –30 °C) of **3**.

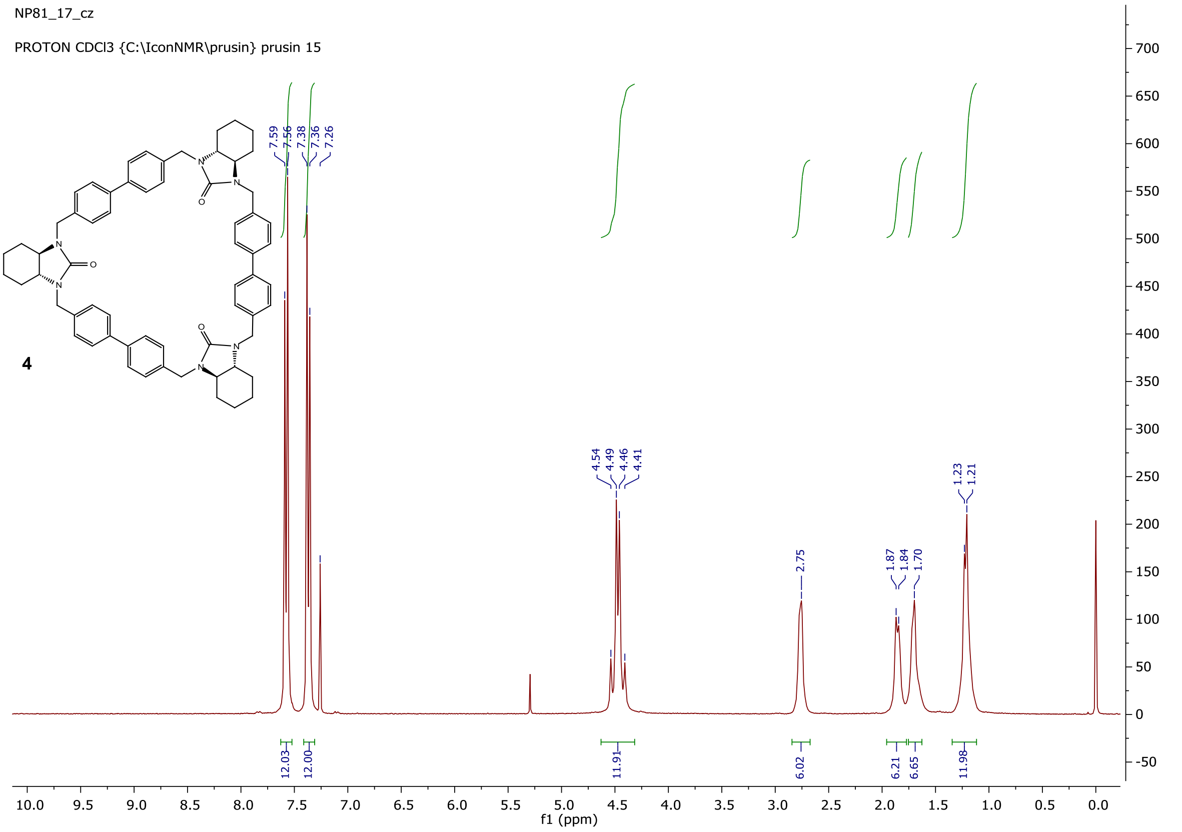

Copy of ^{1}H NMR spectrum ($CDCl_3$, 300 MHz, RT) of **4**.

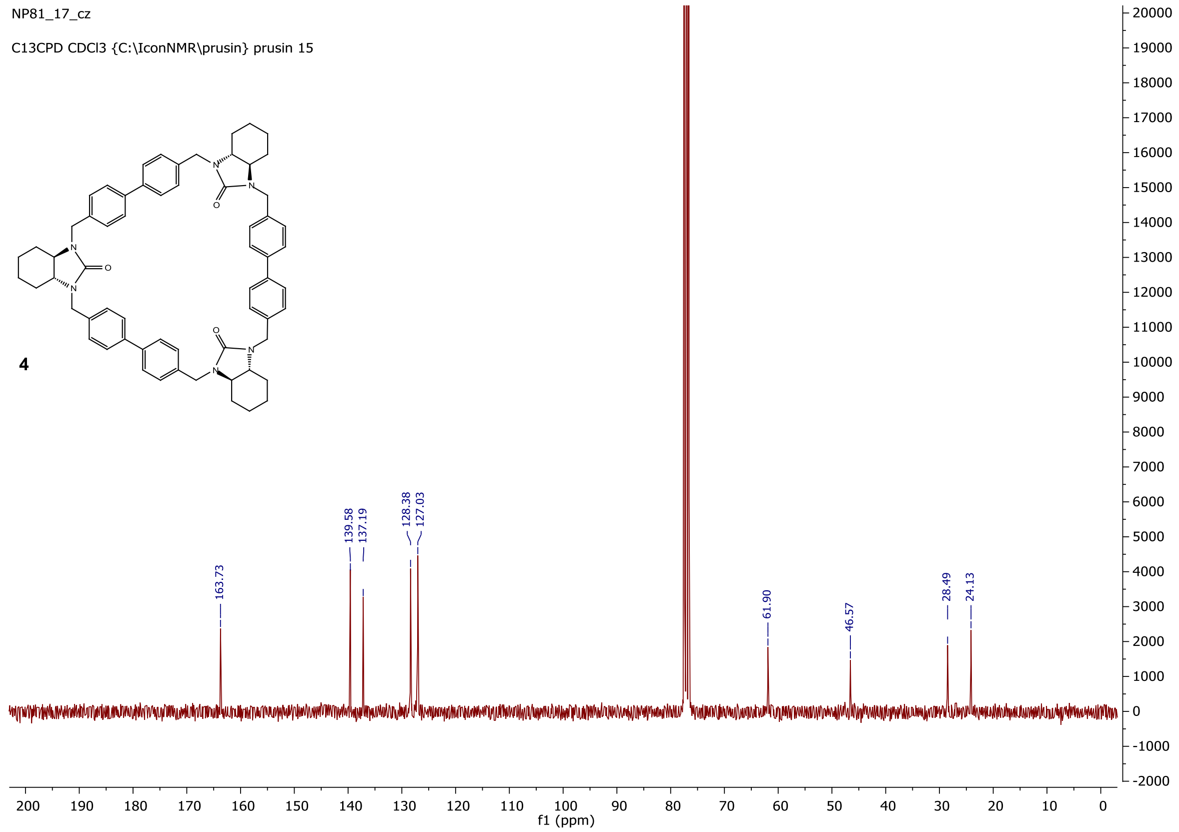

Copy of $^{13}C\{^{1}H\}$ NMR spectrum ($CDCl_3$, 300 MHz, RT) of **4**.

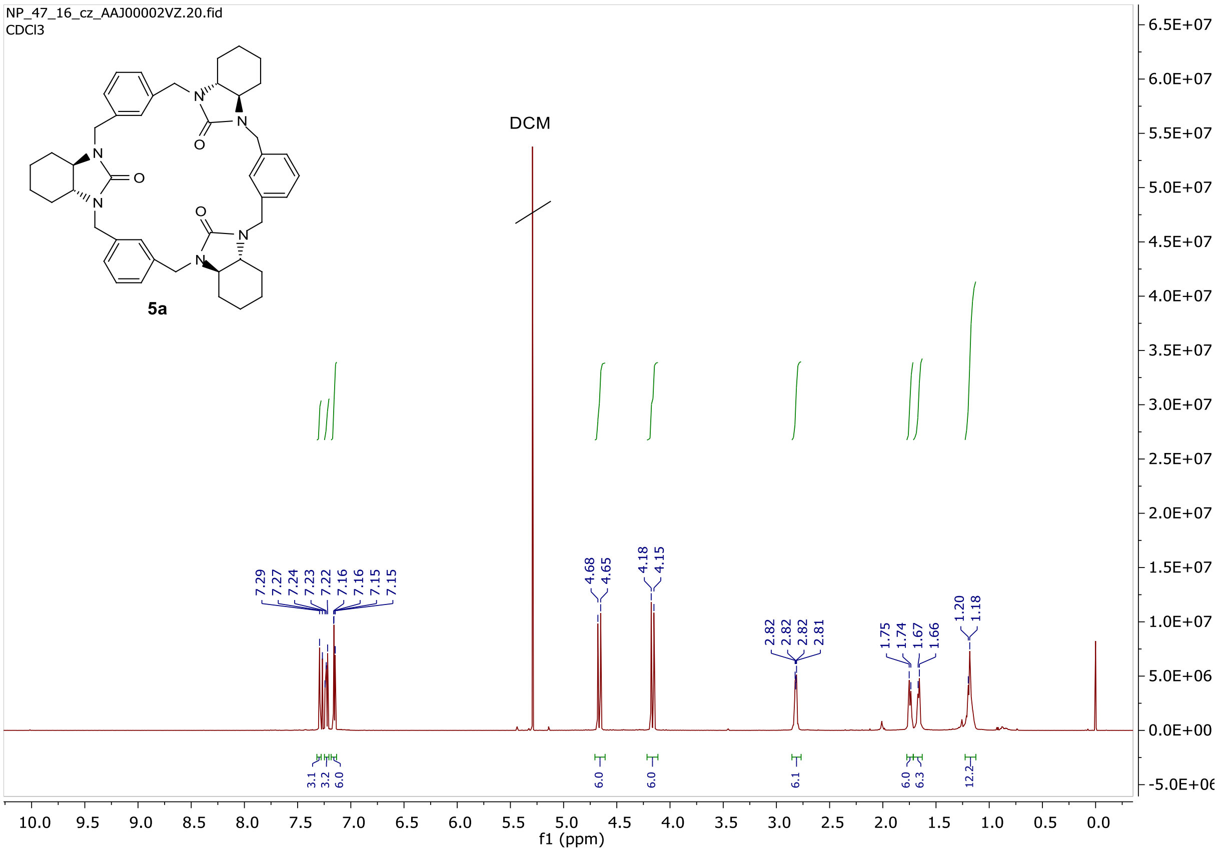

Copy of ^{1}H NMR spectrum ($CDCl_3$, 600 MHz, RT) of **5a**.

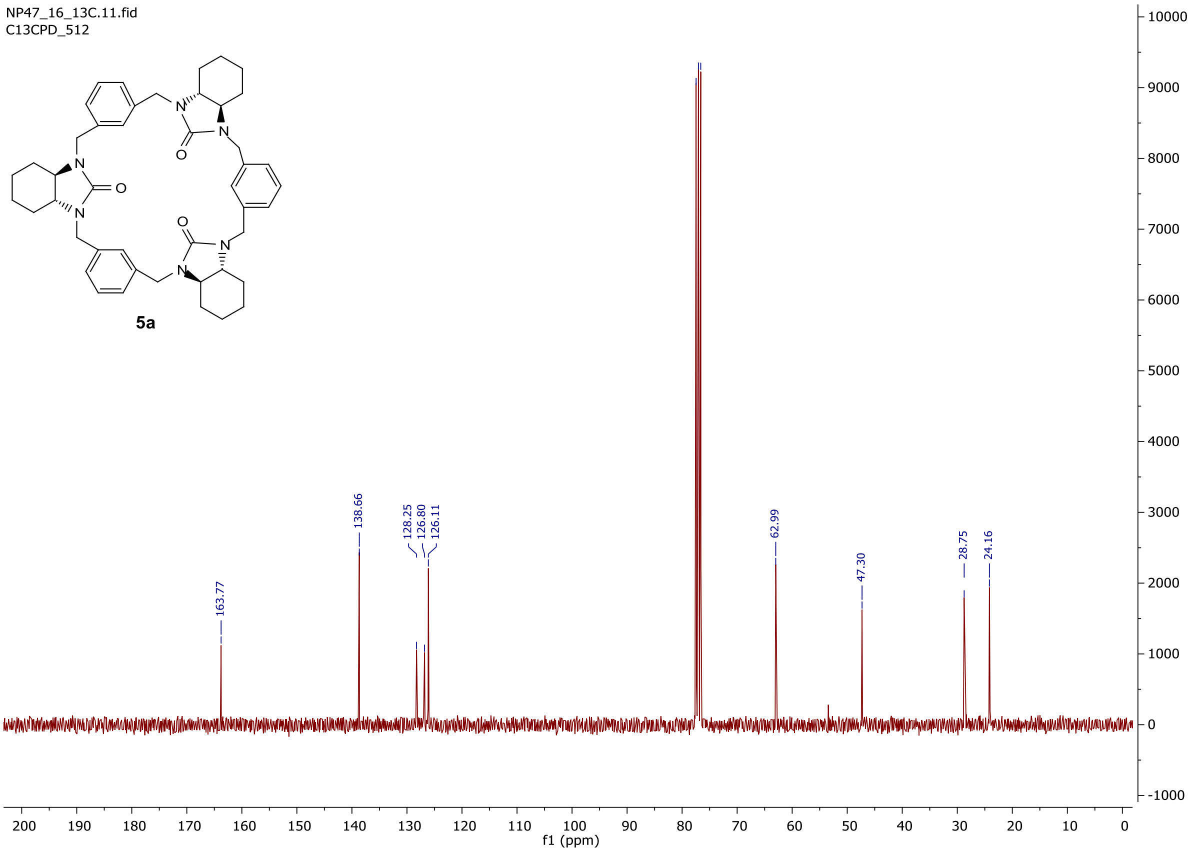

Copy of $^{13}C\{^{1}H\}$ NMR spectrum ($CDCl_3$, 300 MHz, RT) of **5a**.

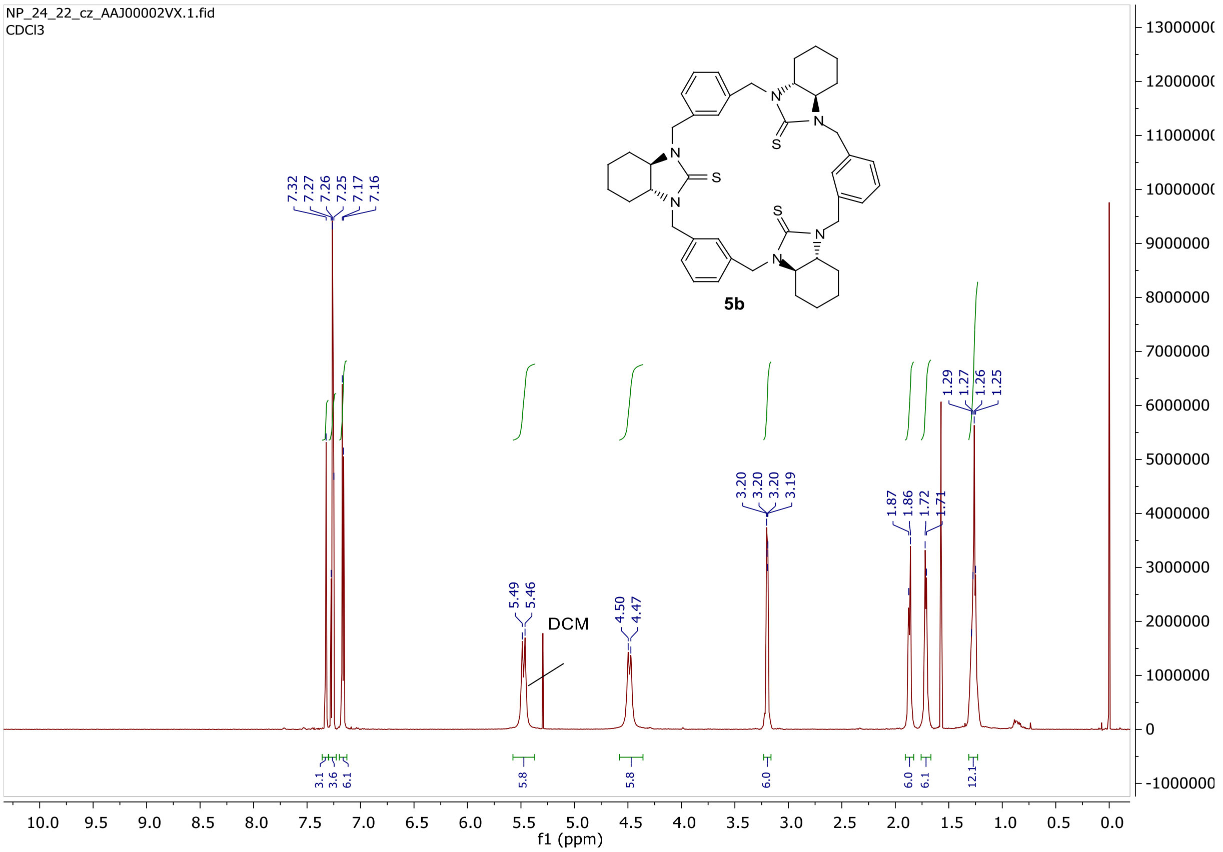

Copy of ^{1}H NMR spectrum ($CDCl_3$, 600 MHz, RT) of **5b**.

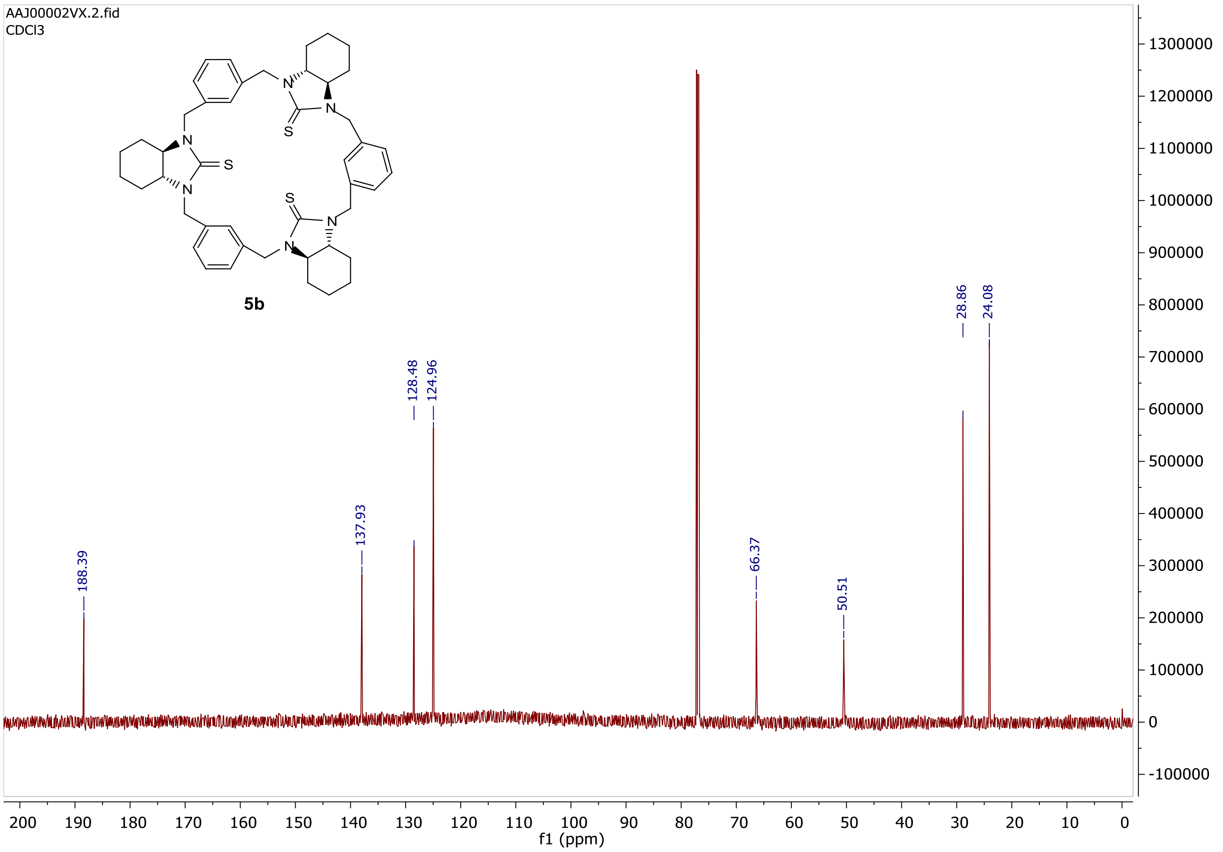

Copy of $^{13}C\{^{1}H\}$ NMR spectrum ($CDCl_3$, 125 MHz, RT) of **5b**.

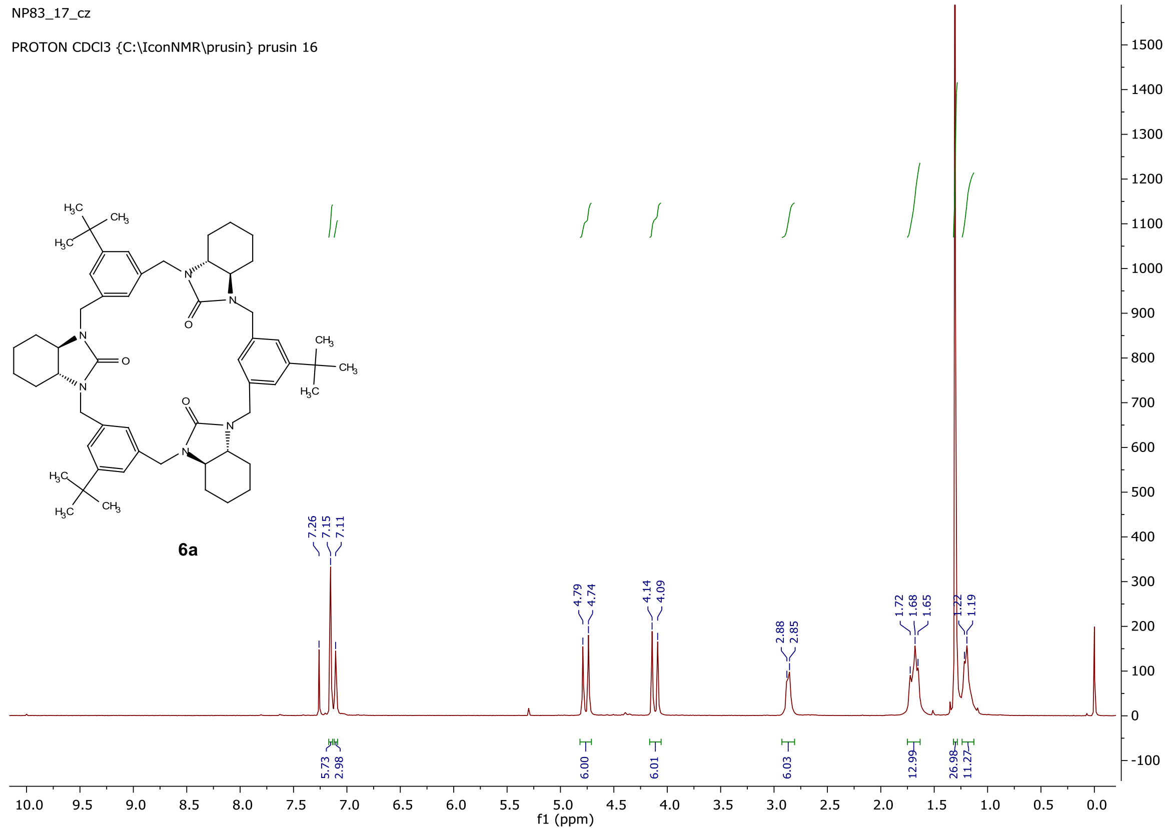

Copy of ^{1}H NMR spectrum ($CDCl_3$, 300 MHz, RT) of **6a**.

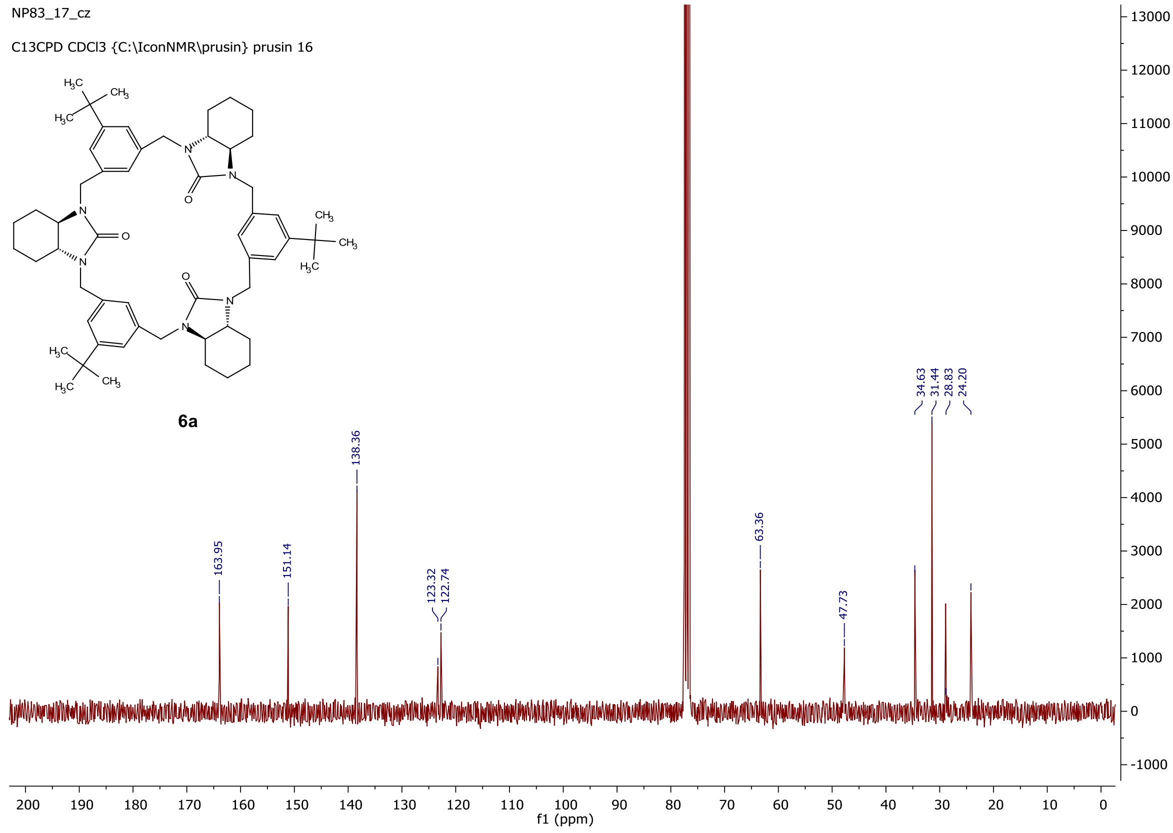

Copy of $^{13}C\{^{1}H\}$ NMR spectrum ($CDCl_3$, 300 MHz, RT) of **6a**.

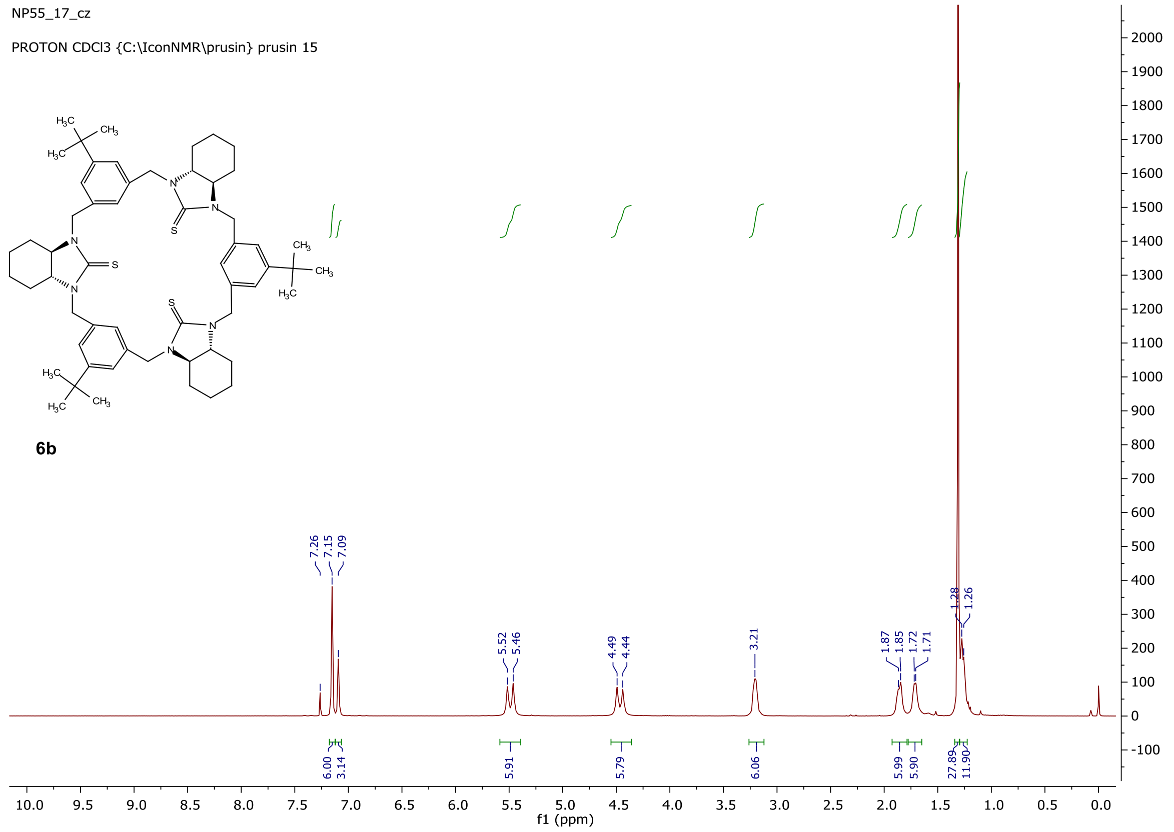

Copy of ^{1}H NMR spectrum ($CDCl_3$, 300 MHz, RT) of **6b**.

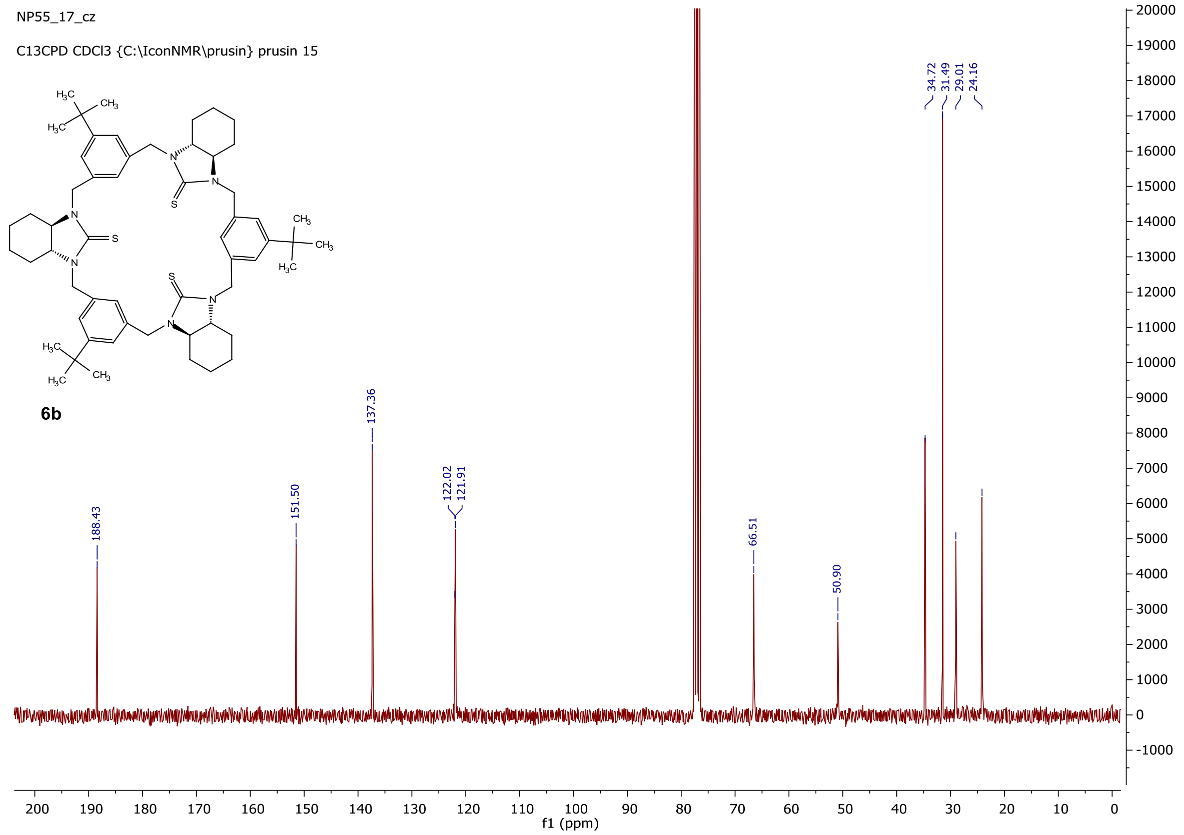

Copy of $^{13}C\{^{1}H\}$ NMR spectrum ($CDCl_3$, 300 MHz, RT) of **6b**.

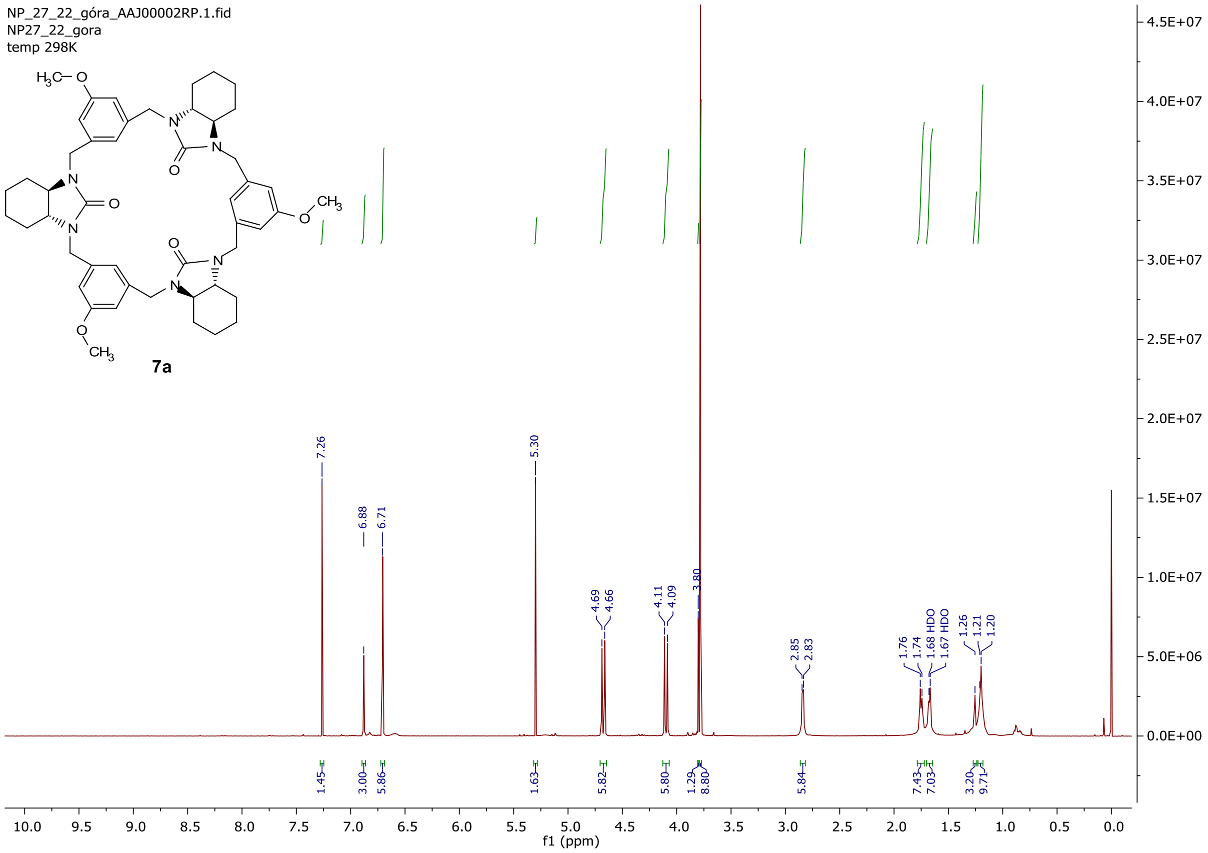

Copy of ^{1}H NMR spectrum ($CDCl_3$, 600 MHz, RT) of **7a**.

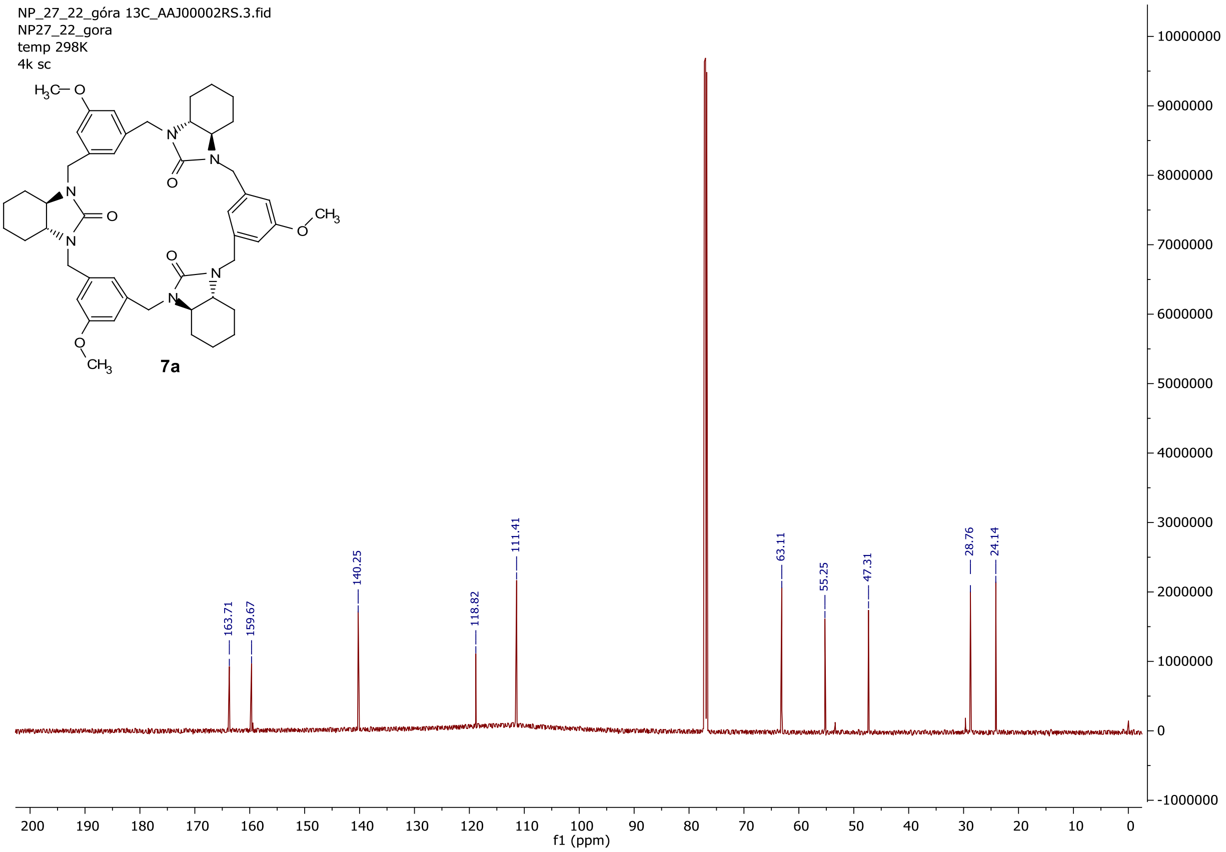

Copy of $^{13}C\{^{1}H\}$ NMR spectrum ($CDCl_3$, 600 MHz, RT) of **7a**.

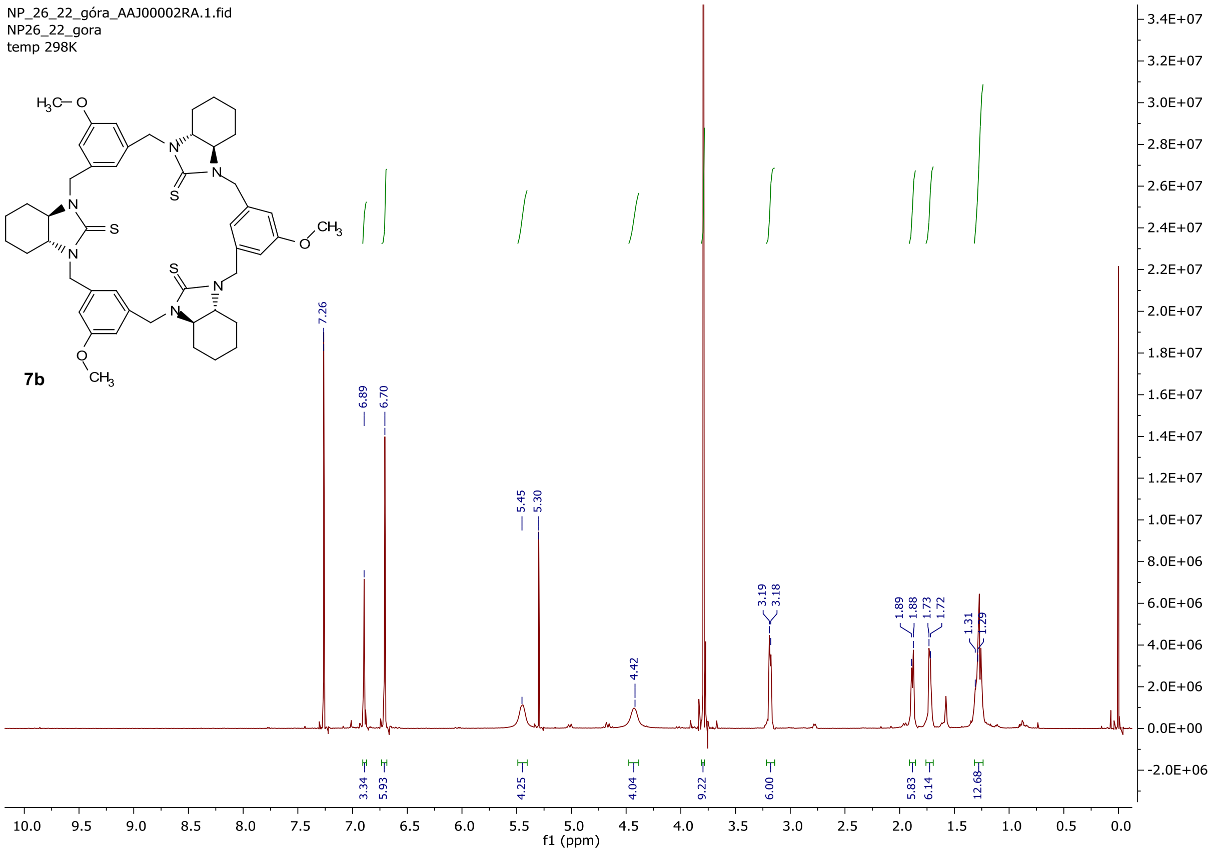

Copy of ^{1}H NMR spectrum ($CDCl_3$, 600 MHz, RT) of **7b**.

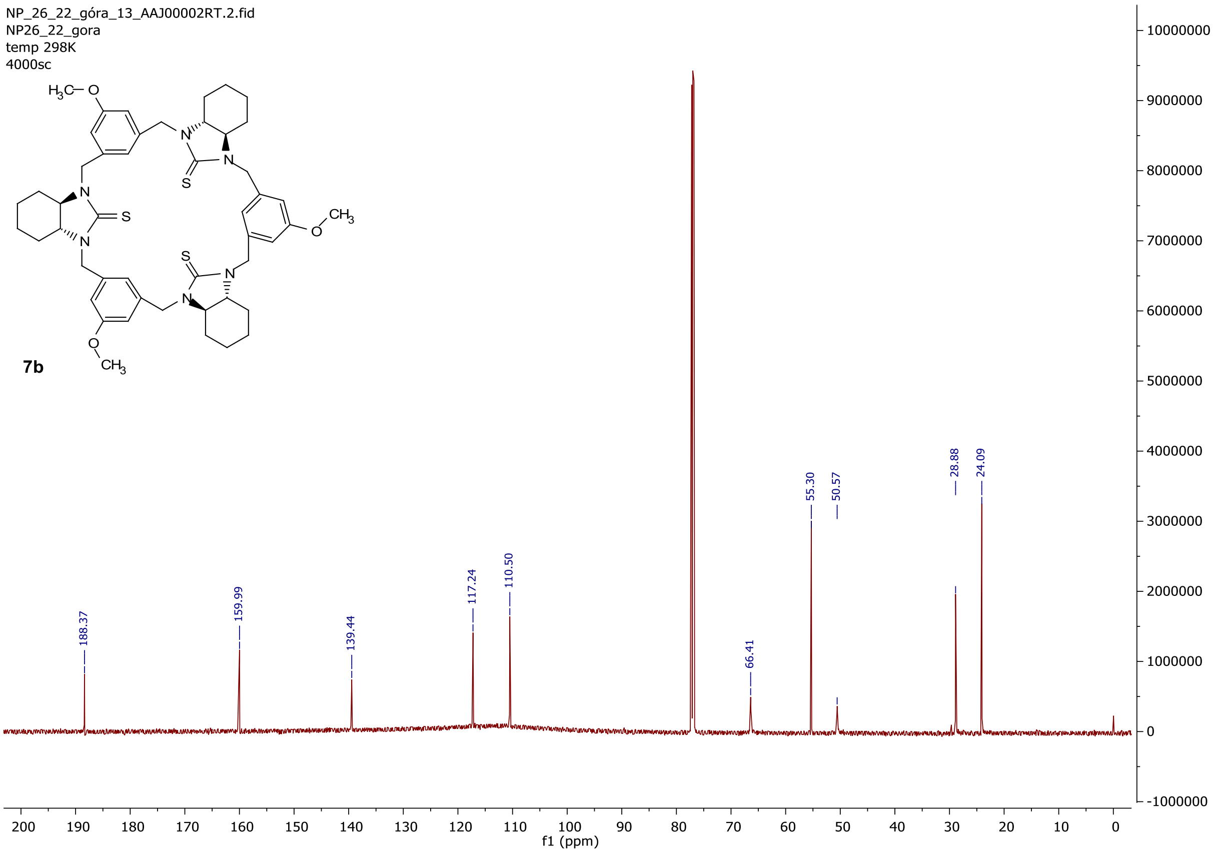

Copy of $^{13}C\{^{1}H\}$ NMR spectrum ($CDCl_3$, 600 MHz, RT) of **7b**.

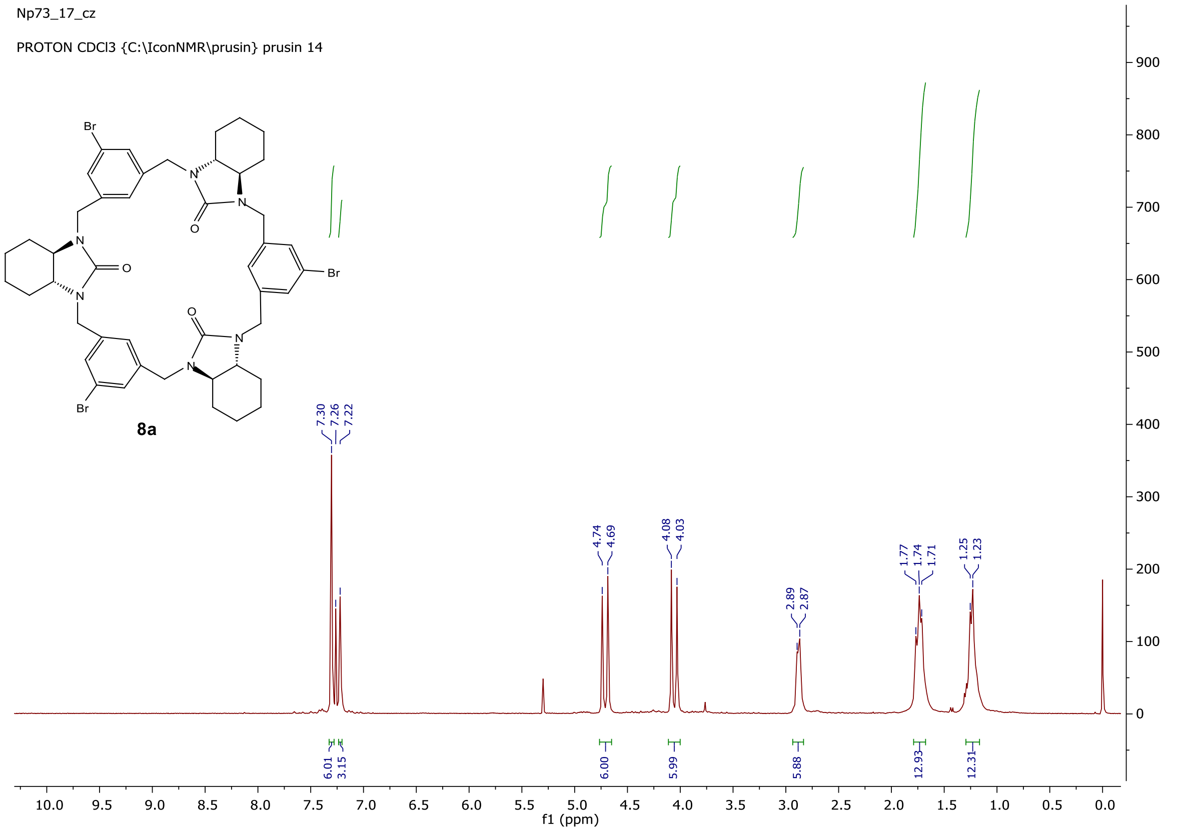

Copy of ^{1}H NMR spectrum ($CDCl_3$, 300 MHz, RT) of **8a**.

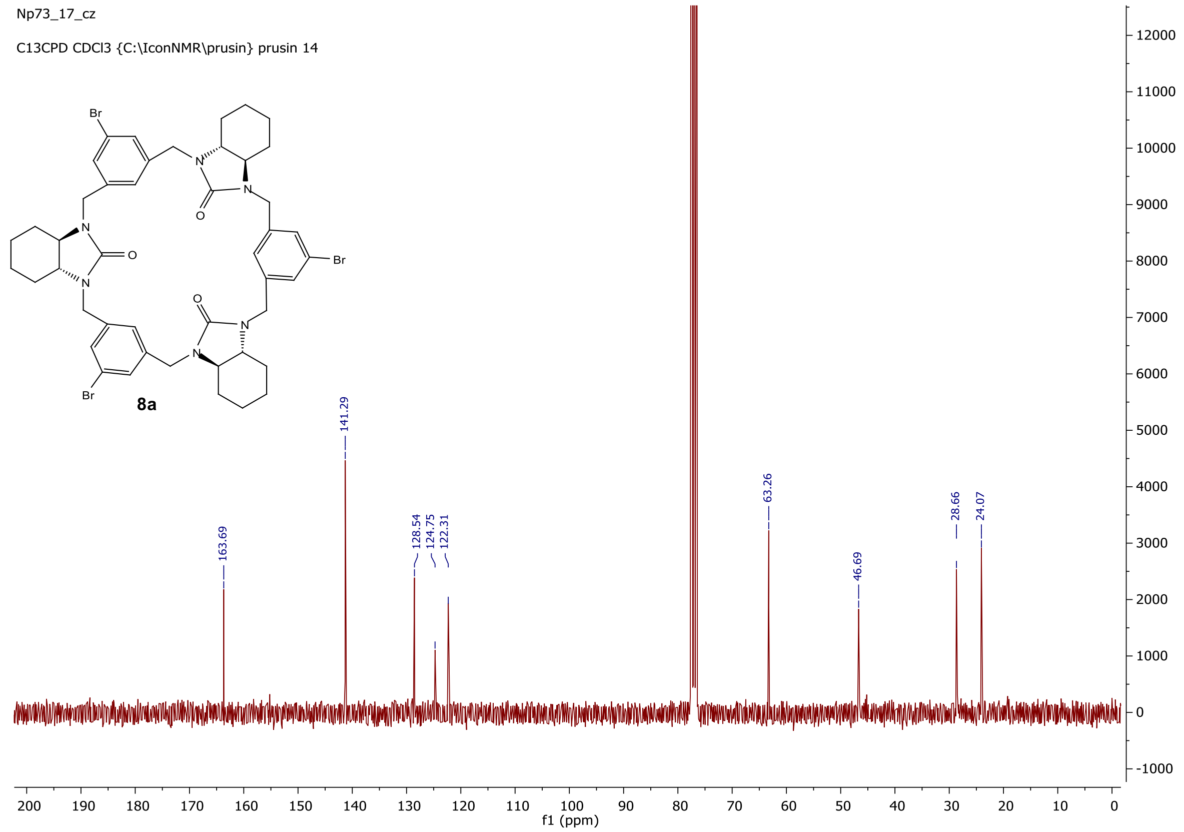

Copy of $^{13}C\{^{1}H\}$ NMR spectrum ($CDCl_3$, 300 MHz, RT) of **8a**.

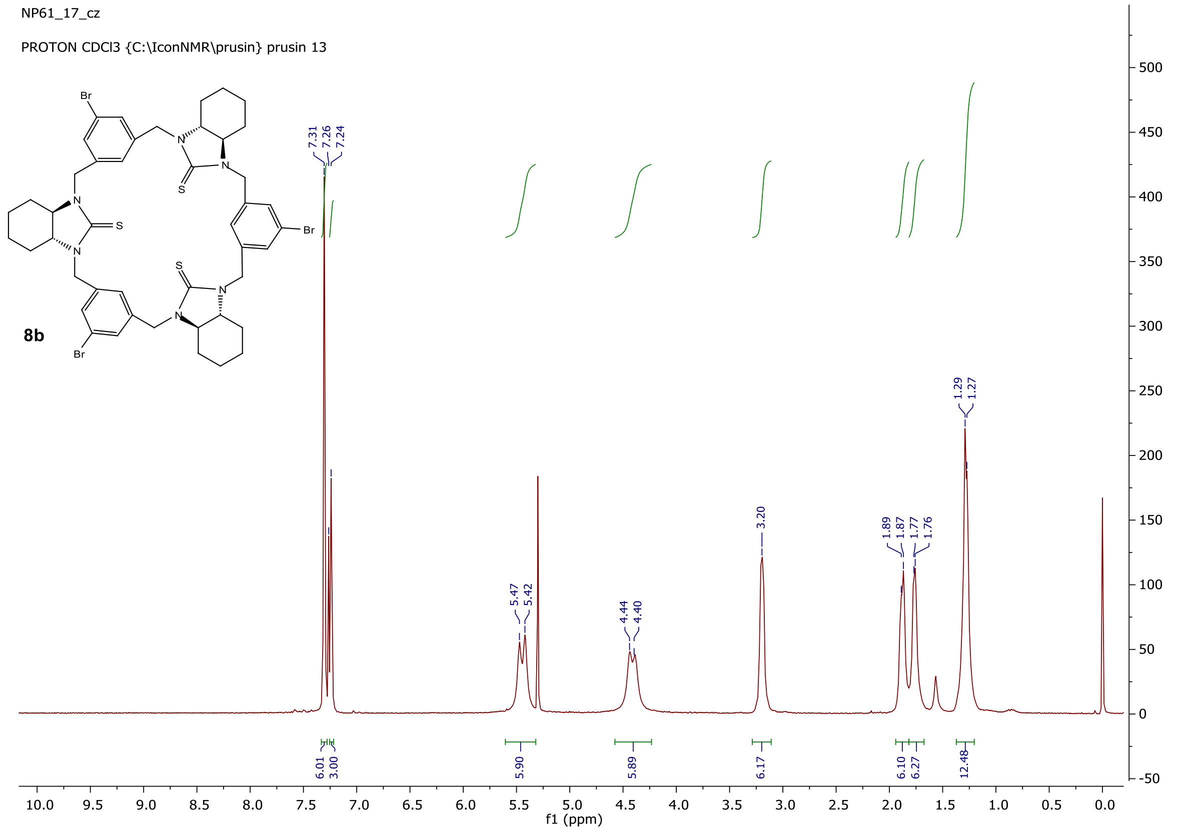

Copy of ^{1}H NMR spectrum ($CDCl_3$, 300 MHz, RT) of **8b**.

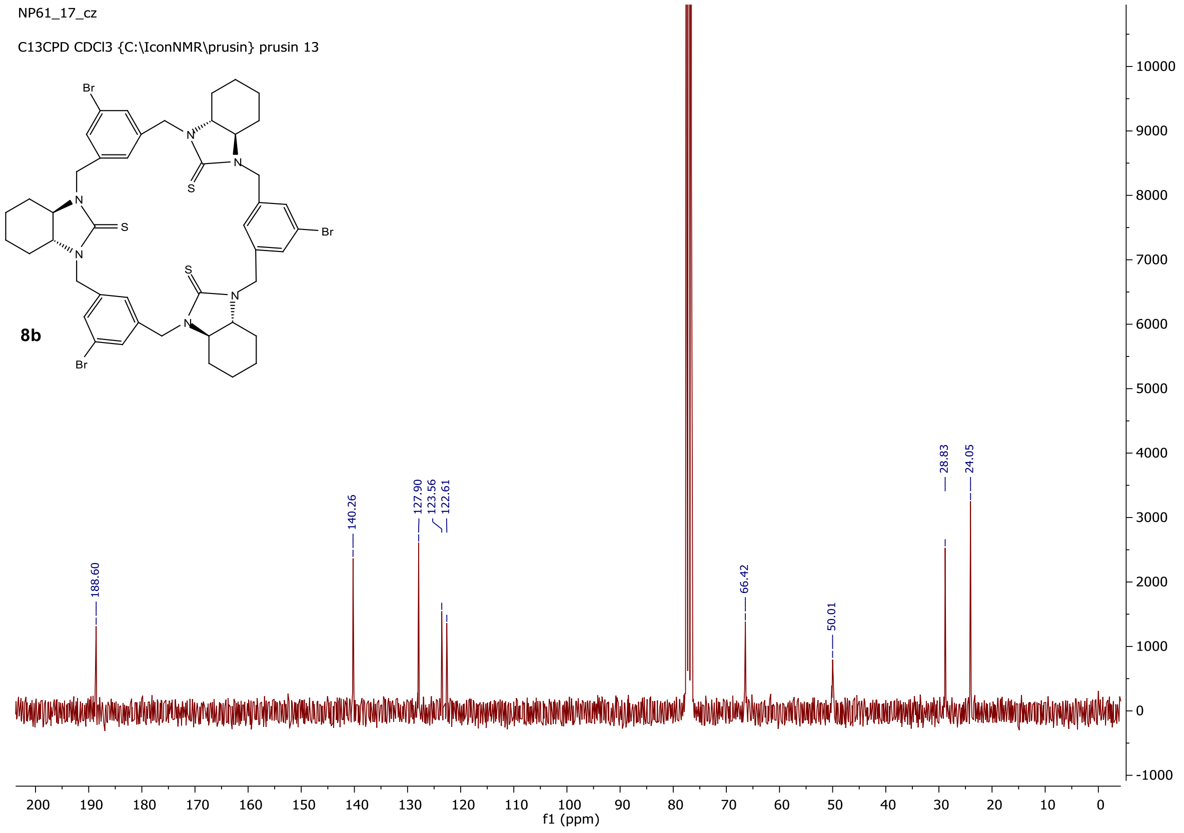

Copy of $^{13}C\{^{1}H\}$ NMR spectrum ($CDCl_3$, 300 MHz, RT) of **8b**.

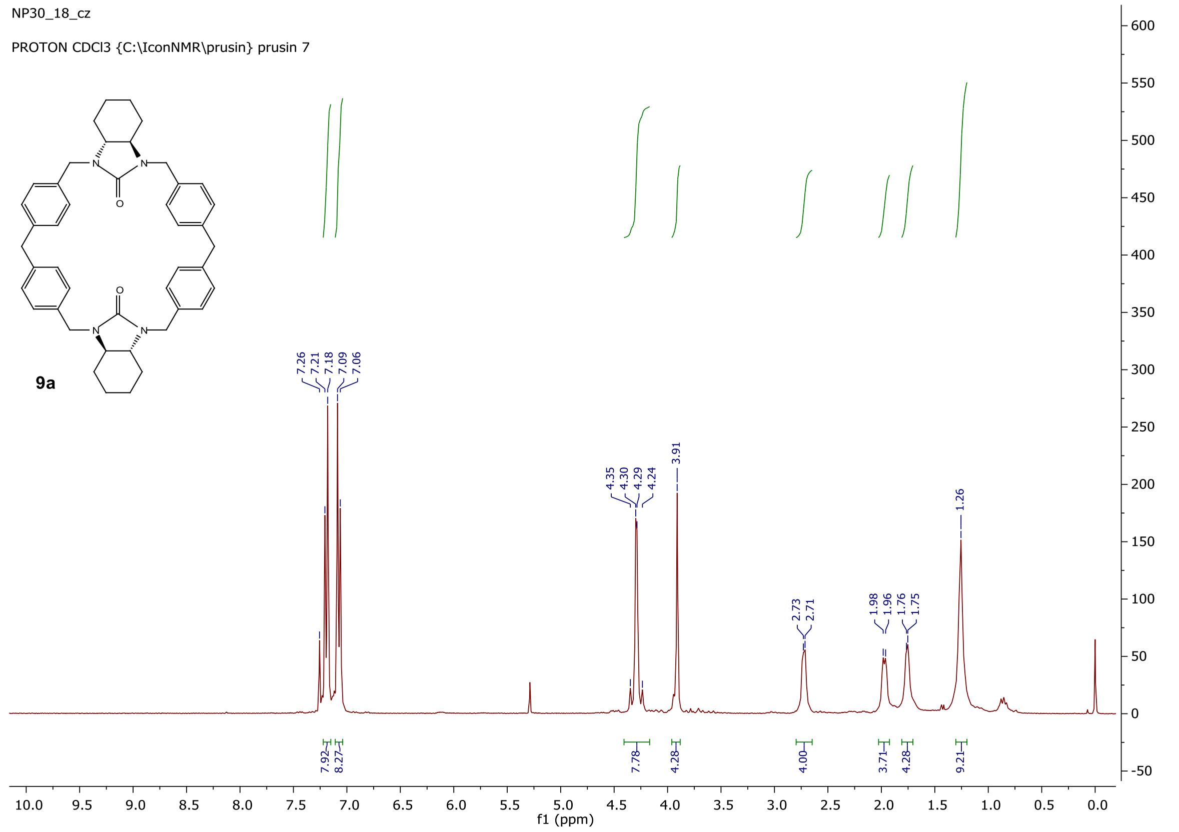

Copy of ^{1}H NMR spectrum ($CDCl_3$, 300 MHz, RT) of **9a**.

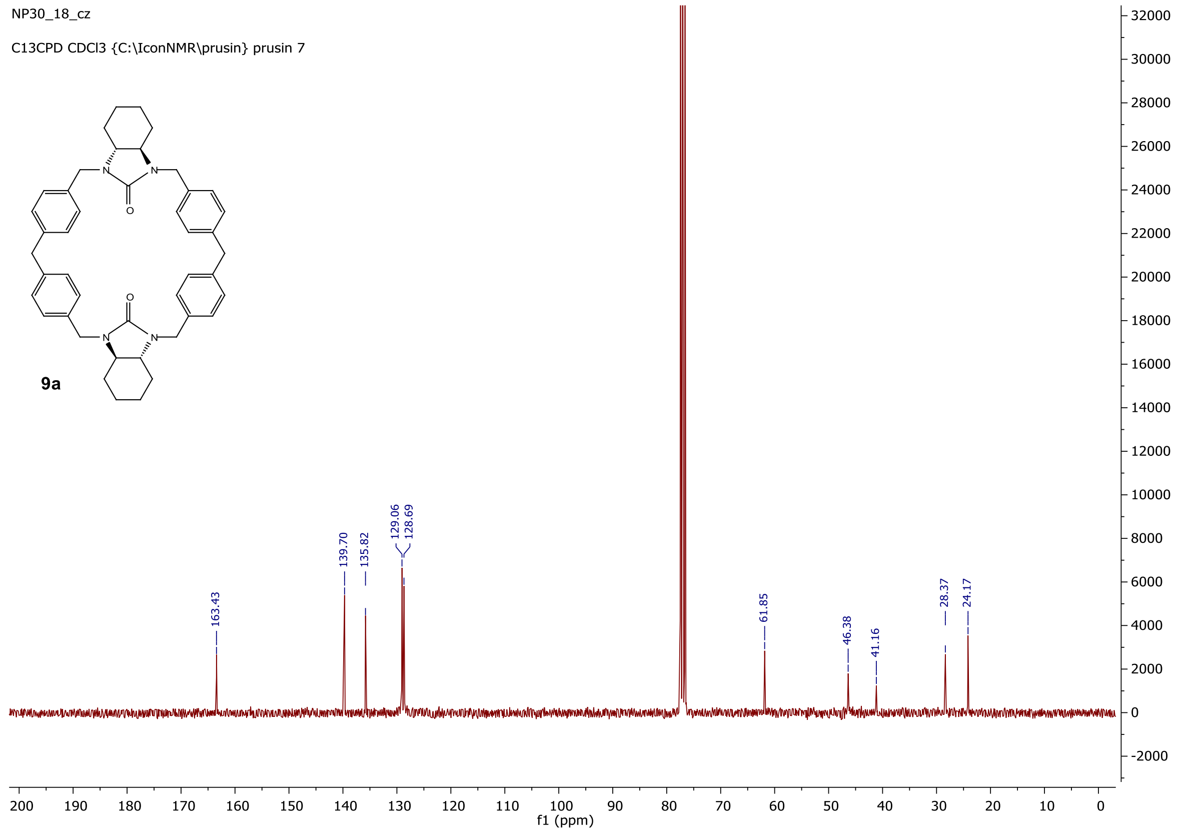

Copy of $^{13}C\{^{1}H\}$ NMR spectrum ($CDCl_3$, 300 MHz, RT) of **9a**.

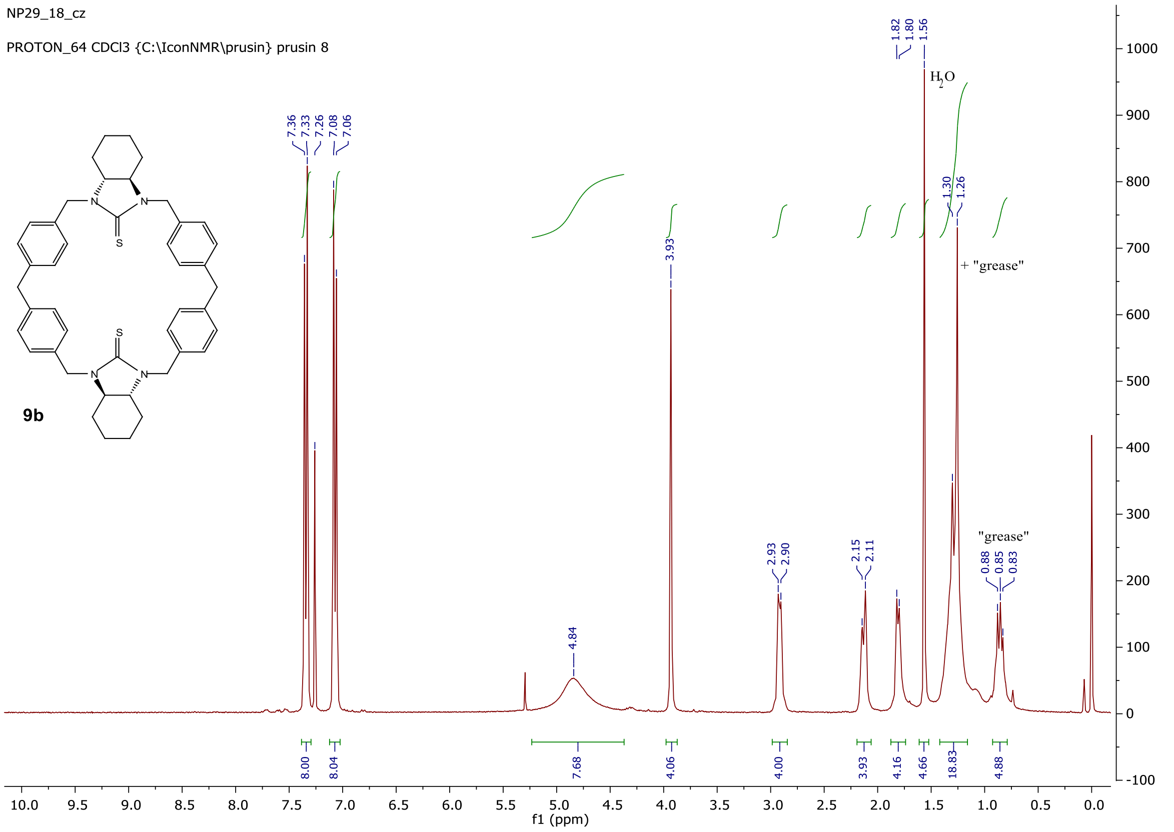

Copy of ^{1}H NMR spectrum ($CDCl_3$, 300 MHz, RT) of **9b**.

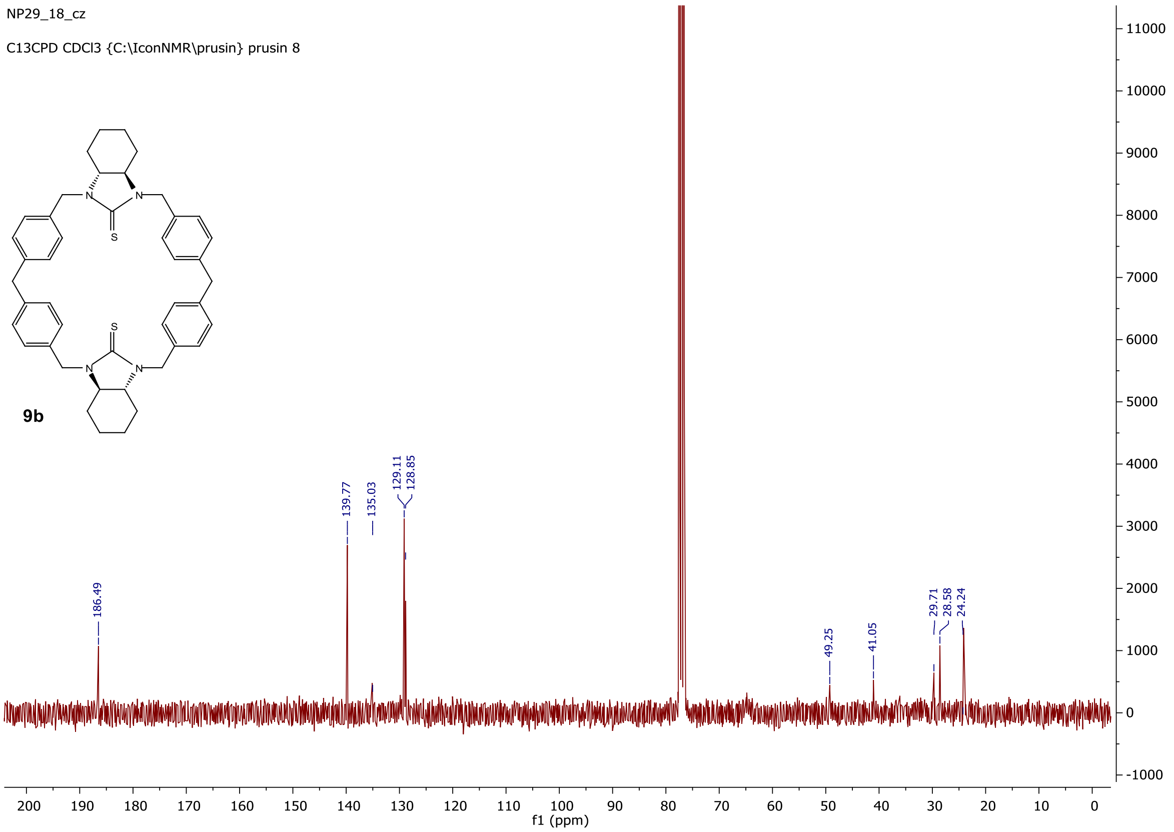

Copy of $^{13}C\{^{1}H\}$ NMR spectrum ($CDCl_3$, 300 MHz, RT) of **9b**.

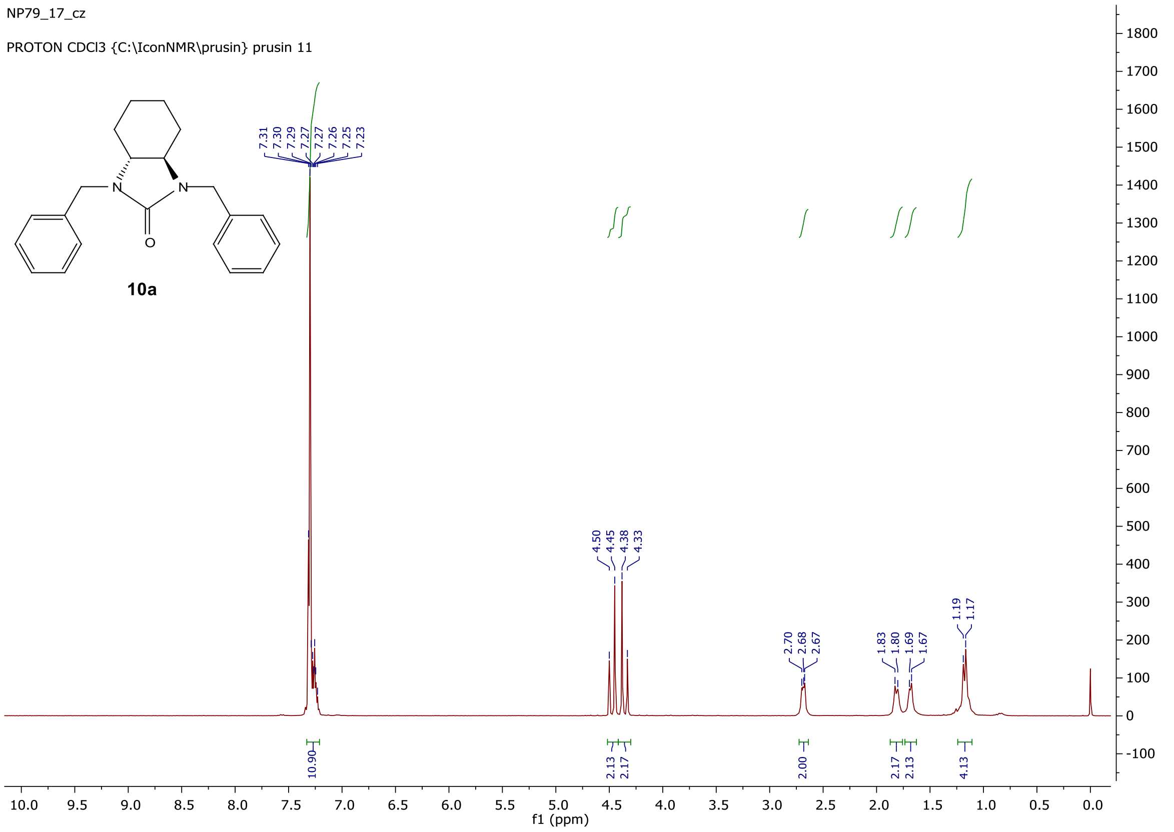

Copy of ^{1}H NMR spectrum ($CDCl_3$, 300 MHz, RT) of **10a**.

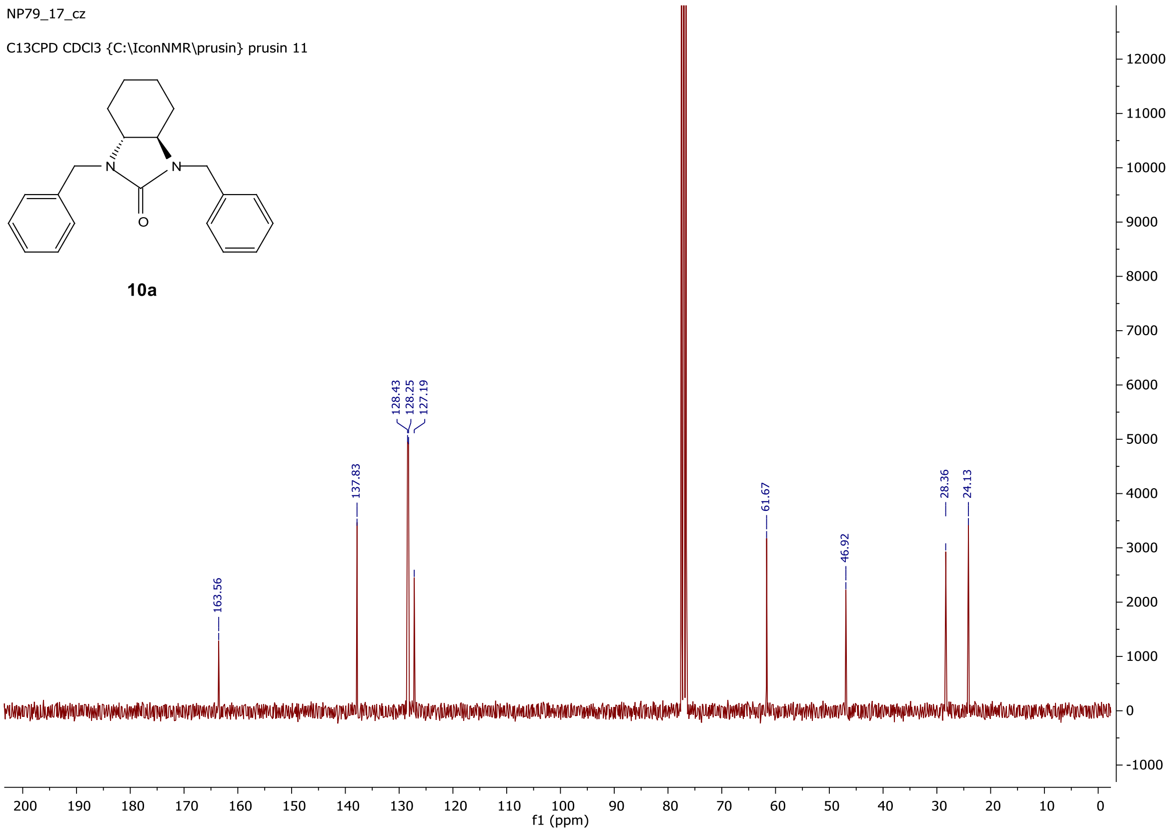

Copy of $^{13}C\{^{1}H\}$ NMR spectrum ($CDCl_3$, 300 MHz, RT) of **10a**.

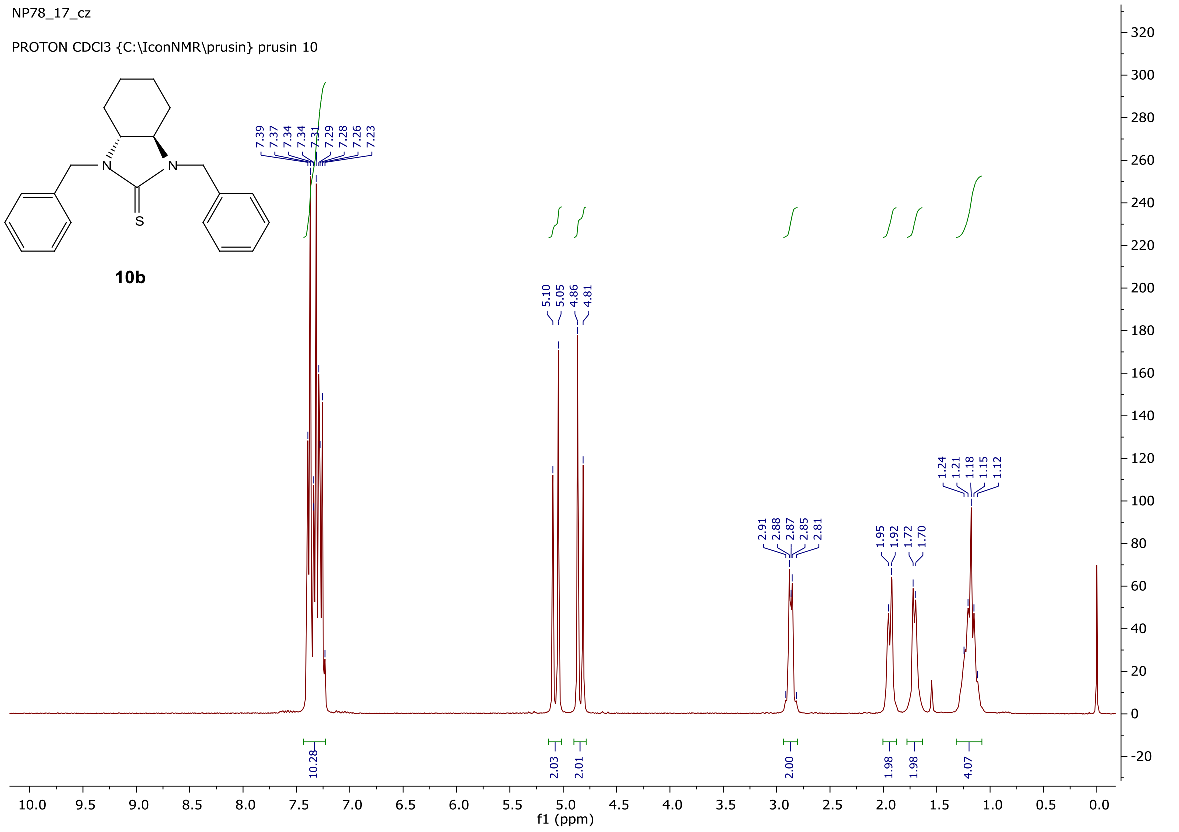

Copy of ^{1}H NMR spectrum ($CDCl_3$, 300 MHz, RT) of **10b**.

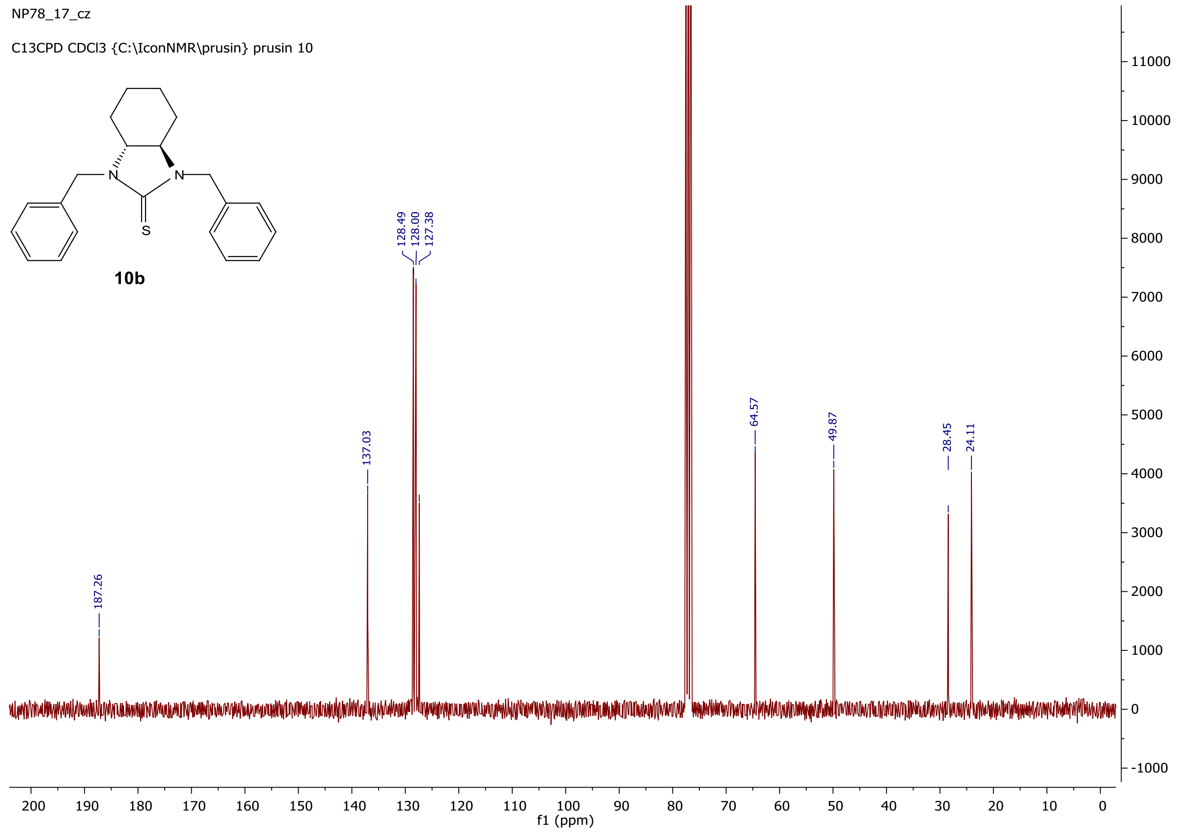

Copy of $^{13}C\{^{1}H\}$ NMR spectrum ($CDCl_3$, 300 MHz, RT) of **10b**.

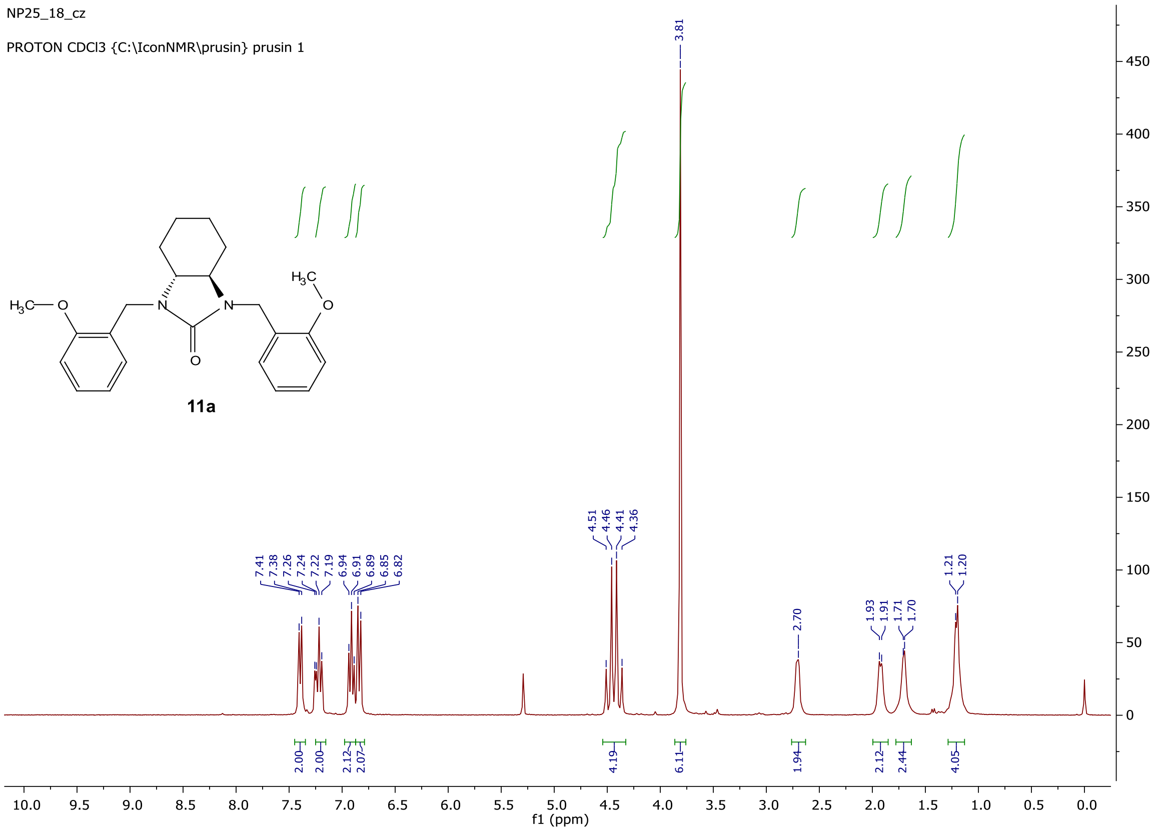

Copy of ^{1}H NMR spectrum ($CDCl_3$, 300 MHz, RT) of **11a**.

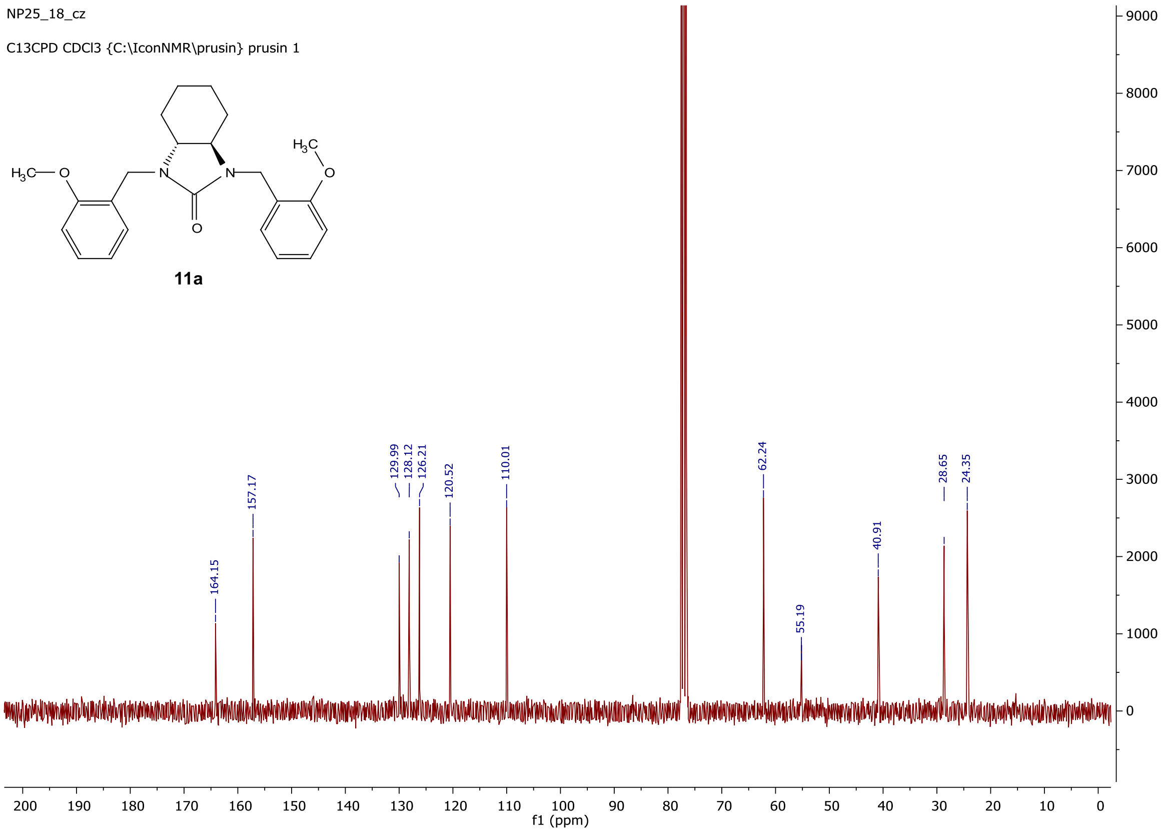

Copy of $^{13}C\{^{1}H\}$ NMR spectrum ($CDCl_3$, 300 MHz, RT) of **11a**.

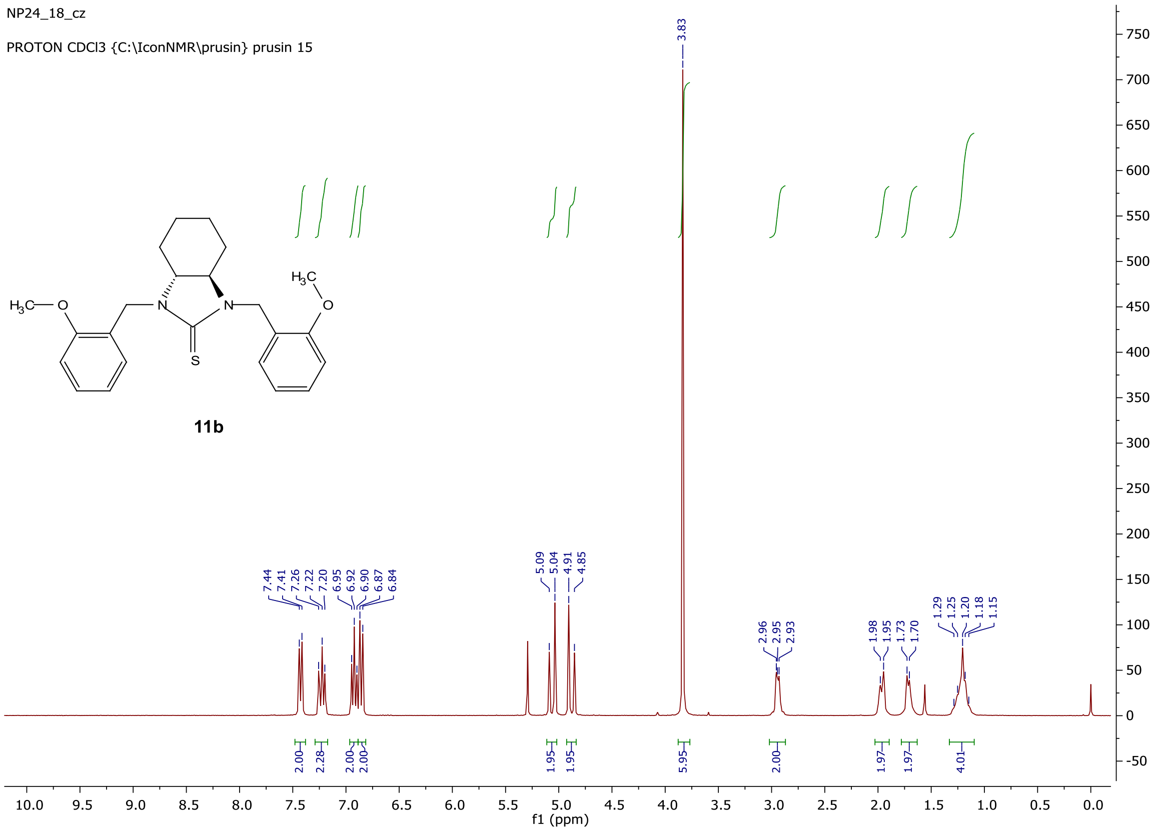

Copy of ^{1}H NMR spectrum ($CDCl_3$, 300 MHz, RT) of **11b**.

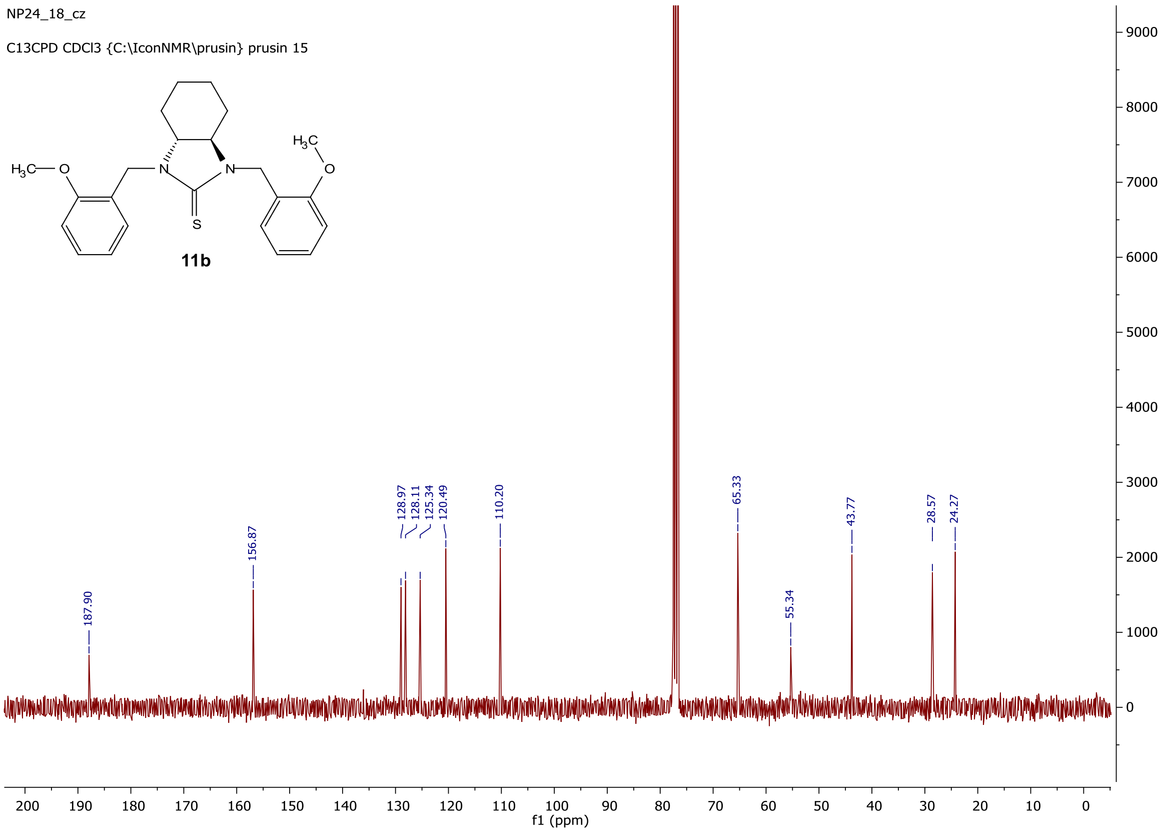

Copy of $^{13}C\{^{1}H\}$ NMR spectrum ($CDCl_3$, 300 MHz, RT) of **11b**.

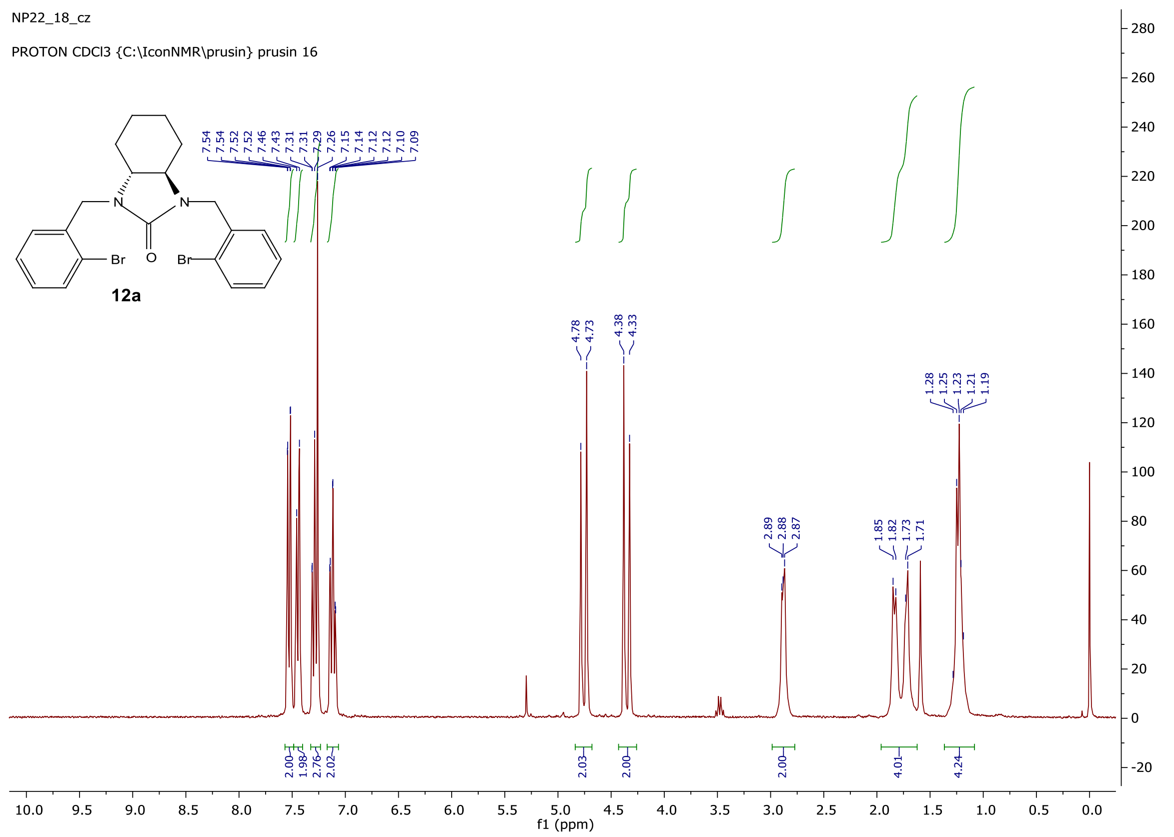

Copy of ^{1}H NMR spectrum ($CDCl_3$,300 MHz, RT) of **12a**.

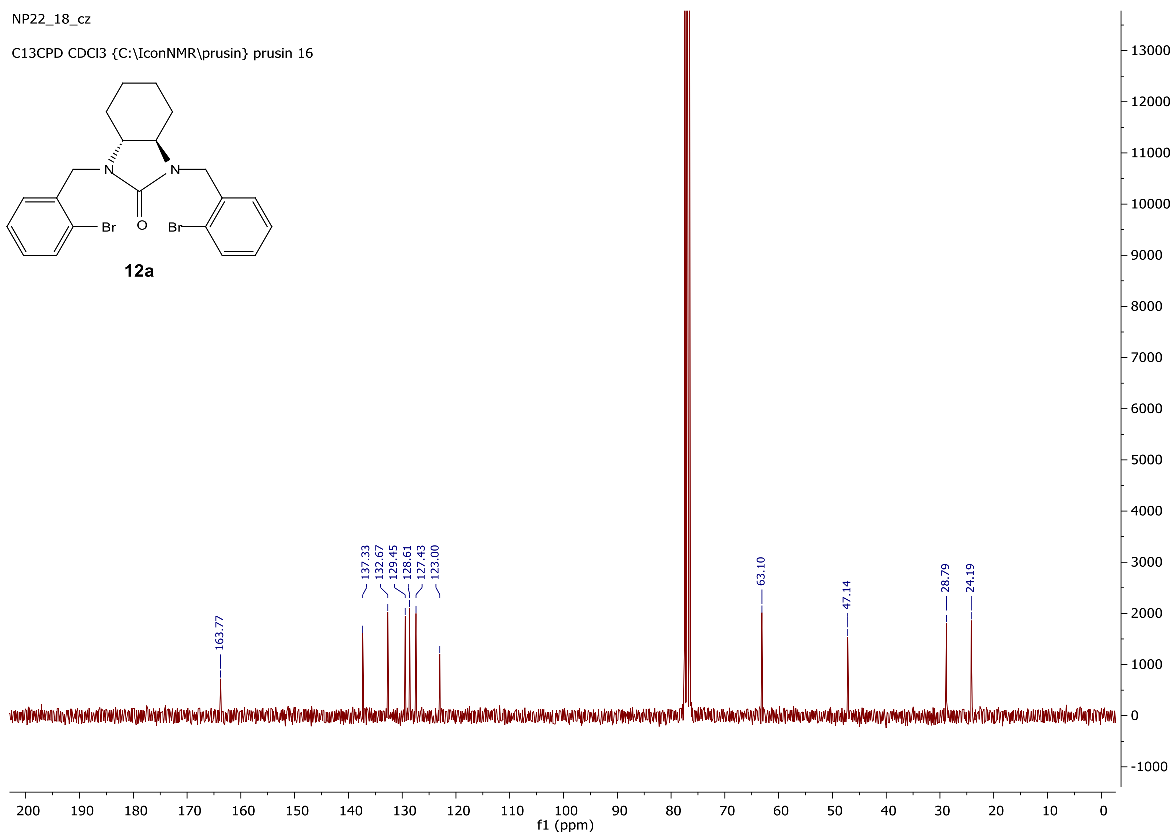

Copy of $^{13}C\{^{1}H\}$ NMR spectrum ($CDCl_3$, 300 MHz, RT) of **12a**.

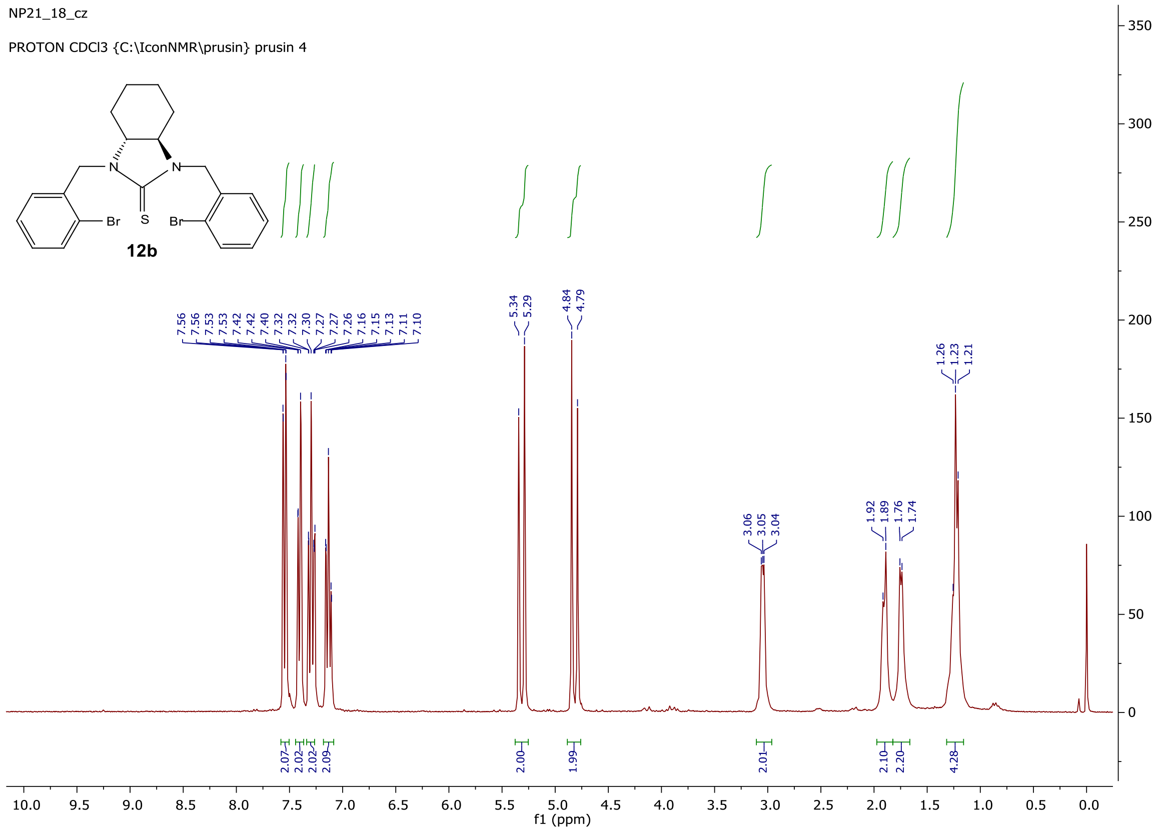

Copy of ^{1}H NMR spectrum ($CDCl_3$, 300 MHz, RT) of **12b**.

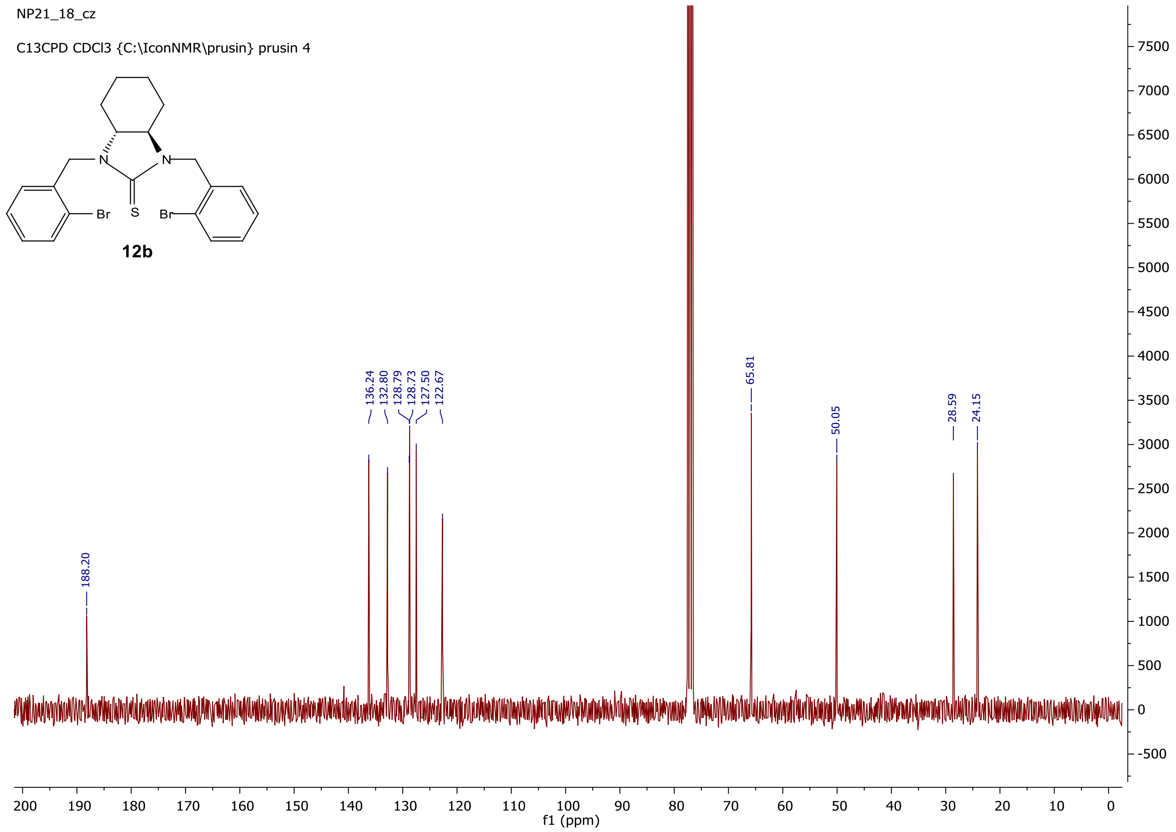

Copy of $^{13}C\{^{1}H\}$ NMR spectrum ($CDCl_3$, 300 MHz, RT) of **12b**.

Cartesian coordinates

1a (*in vacuo*)

Conf. 1[a]	C	-5.73620000	-5.05510000	0.10340000
	C	-7.24600000	-2.44020000	-0.10340000
	C	-5.76900000	-2.60870000	-0.43910000
	C	-5.14380000	-3.69170000	0.43910000
	H	-5.52780000	-5.30280000	-0.94310000
	H	-5.29750000	-5.84690000	0.71770000
	H	-7.71230000	-1.66430000	-0.71770000
	H	-5.69630000	-2.94200000	-1.49070000
	N	-4.83890000	-1.50370000	-0.22050000
	H	-5.39600000	-3.46210000	1.49070000
	N	-3.72180000	-3.43880000	0.22050000
	H	-7.35630000	-2.13580000	0.94310000
	C	1.06780000	-4.19700000	0.88790000
	C	0.24040000	-4.18510000	-1.35950000
	C	1.32560000	-4.16980000	-0.48160000
	C	-1.06780000	-4.19700000	-0.88790000
	C	-1.32560000	-4.16980000	0.48160000
	C	-0.24040000	-4.18510000	1.35950000
	H	1.89450000	-4.16900000	1.58760000
	H	0.41760000	-4.14850000	-2.43010000
	H	-1.89450000	-4.16900000	-1.58760000
	H	-0.41760000	-4.14850000	2.43010000
	C	3.74460000	1.88440000	1.35950000
	C	4.27390000	0.93690000	0.48160000

C	4.16860000	1.17370000	-0.88790000
H	3.80150000	1.71260000	2.43010000
H	4.55770000	0.44390000	-1.58760000
C	-4.16860000	1.17370000	0.88790000
C	-3.74460000	1.88440000	-1.35950000
C	-4.27390000	0.93690000	-0.48160000
H	-4.55770000	0.44390000	1.58760000
C	-7.25800000	-4.97870000	0.35960000
C	-7.94060000	-3.79620000	-0.35960000
H	-7.42560000	-4.88480000	1.43940000
H	-7.73350000	-5.91560000	0.05530000
H	-7.94310000	-3.98830000	-1.43940000
H	-8.98980000	-3.73960000	-0.05530000
C	3.50420000	2.30070000	-1.35950000
C	3.10080000	3.02330000	0.88790000
C	2.94830000	3.23290000	-0.48160000
H	3.38390000	2.43590000	-2.43010000
H	2.66330000	3.72520000	1.58760000
C	-1.50970000	7.49530000	0.10340000
C	1.50970000	7.49530000	-0.10340000
C	0.62530000	6.30050000	-0.43910000
C	-0.62530000	6.30050000	0.43910000
H	-1.82850000	7.43860000	-0.94310000
H	-2.41490000	7.51120000	0.71770000
H	2.41490000	7.51120000	-0.71770000
H	0.30030000	6.40410000	-1.49070000
N	1.11720000	4.94250000	-0.22050000

H	-0.30030000	6.40410000	1.49070000
N	-1.11720000	4.94250000	0.22050000
H	1.82850000	7.43860000	0.94310000
C	-3.50420000	2.30070000	1.35950000
C	-2.94830000	3.23290000	0.48160000
C	-3.10080000	3.02330000	-0.88790000
H	-3.38390000	2.43590000	2.43010000
H	-2.66330000	3.72520000	-1.58760000
C	-0.68270000	8.77490000	0.35960000
C	0.68270000	8.77490000	-0.35960000
H	-0.51760000	8.87310000	1.43940000
H	-1.25630000	9.65520000	0.05530000
H	0.51760000	8.87310000	-1.43940000
H	1.25630000	9.65520000	-0.05530000
H	-3.80150000	1.71260000	-2.43010000
C	7.24600000	-2.44020000	0.10340000
C	5.73620000	-5.05510000	-0.10340000
C	5.14380000	-3.69170000	-0.43910000
C	5.76900000	-2.60870000	0.43910000
H	7.35630000	-2.13580000	-0.94310000
H	7.71230000	-1.66430000	0.71770000
H	5.29750000	-5.84690000	-0.71770000
H	5.39600000	-3.46210000	-1.49070000
N	3.72180000	-3.43880000	-0.22050000
H	5.69630000	-2.94200000	1.49070000
N	4.83890000	-1.50370000	0.22050000
H	5.52780000	-5.30280000	0.94310000

C	7.94060000	-3.79620000	0.35960000
C	7.25800000	-4.97870000	-0.35960000
H	7.94310000	-3.98830000	1.43940000
H	8.98980000	-3.73960000	0.05530000
H	7.42560000	-4.88480000	-1.43940000
H	7.73350000	-5.91560000	-0.05530000
C	-3.56390000	-2.05760000	0.00000000
C	-2.73460000	-4.13810000	1.04420000
H	-2.70090000	-3.70240000	2.05330000
H	-3.09850000	-5.16490000	1.15520000
C	-4.95100000	-0.29920000	-1.04420000
H	-4.55690000	-0.48780000	-2.05330000
H	-6.02220000	-0.10100000	-1.15520000
C	0.00000000	4.11520000	0.00000000
C	-2.21640000	4.43730000	1.04420000
H	-1.85600000	4.19030000	2.05330000
H	-2.92370000	5.26590000	1.15520000
C	2.21640000	4.43730000	-1.04420000
H	1.85600000	4.19030000	-2.05330000
H	2.92370000	5.26590000	-1.15520000
C	3.56390000	-2.05760000	0.00000000
C	2.73460000	-4.13810000	-1.04420000
H	3.09850000	-5.16490000	-1.15520000
H	2.70090000	-3.70240000	-2.05330000
C	4.95100000	-0.29920000	1.04420000
H	6.02220000	-0.10100000	1.15520000
H	4.55690000	-0.48780000	2.05330000

	O	0.00000000	2.90840000	0.00000000
	O	2.51870000	-1.45420000	0.00000000
	O	-2.51870000	-1.45420000	0.00000000
Conf. 2[b]	C	2.05440000	3.51490000	-1.94180000
	C	4.80720000	2.24390000	-2.04520000
	C	3.92930000	2.30970000	-0.80240000
	C	3.08150000	3.58300000	-0.81880000
	H	1.39200000	2.65620000	-1.79010000
	H	1.42360000	4.40740000	-1.96330000
	H	5.42670000	1.34260000	-2.04880000
	H	3.24770000	1.44360000	-0.82430000
	N	4.53630000	2.40270000	0.52240000
	H	3.74890000	4.43950000	-1.02610000
	N	2.65960000	3.65290000	0.57710000
	H	5.48190000	3.10670000	-2.07460000
	C	-1.81490000	5.24960000	0.33890000
	C	-1.56150000	3.36310000	1.80350000
	C	-2.39830000	4.21170000	1.06390000
	C	-0.18510000	3.54410000	1.79860000
	C	0.40060000	4.58530000	1.06720000
	C	-0.43140000	5.43820000	0.34560000
	H	-2.44450000	5.92470000	-0.23280000
	H	-2.00130000	2.56990000	2.39820000
	H	0.44970000	2.88260000	2.37820000
	H	0.00000000	6.25940000	-0.21900000
	C	-4.49390000	-3.09270000	0.34560000
	C	-4.17130000	-1.94570000	1.06720000

C	-2.97670000	-1.93230000	1.79860000
H	-5.42080000	-3.12970000	-0.21900000
H	-2.72130000	-1.05180000	2.37820000
C	5.45370000	-1.05310000	0.33890000
C	3.69330000	-0.32920000	1.80350000
C	4.84660000	-0.02890000	1.06390000
H	6.35320000	-0.84530000	-0.23280000
C	2.83770000	3.38770000	-3.26800000
C	3.86570000	2.23550000	-3.27110000
H	3.36180000	4.33310000	-3.45400000
H	2.14020000	3.25550000	-4.10030000
H	3.32960000	1.27900000	-3.28840000
H	4.45460000	2.27460000	-4.19210000
C	-2.13170000	-3.03390000	1.80350000
C	-3.63890000	-4.19650000	0.33890000
C	-2.44830000	-4.18290000	1.06390000
H	-1.22490000	-3.01820000	2.39820000
H	-3.90870000	-5.07930000	-0.23280000
C	2.01680000	-3.53660000	-1.94180000
C	-0.46030000	-5.28510000	-2.04520000
C	0.03560000	-4.55770000	-0.80240000
C	1.56220000	-4.46010000	-0.81880000
H	1.60440000	-2.53370000	-1.79010000
H	3.10510000	-3.43660000	-1.96330000
H	-1.55060000	-5.37090000	-2.04880000
H	-0.37370000	-3.53440000	-0.82430000
N	-0.18730000	-5.12990000	0.52240000

H	1.97020000	-5.46640000	-1.02610000
N	1.83370000	-4.12980000	0.57710000
H	-0.05050000	-6.30080000	-2.07460000
C	4.92530000	-2.34550000	0.34560000
C	3.77060000	-2.63960000	1.06720000
C	3.16180000	-1.61180000	1.79860000
H	5.42080000	-3.12970000	-0.21900000
H	2.27160000	-1.83080000	2.37820000
C	1.51500000	-4.15130000	-3.26800000
C	0.00310000	-4.46560000	-3.27110000
H	2.07170000	-5.07800000	-3.45400000
H	1.74920000	-3.48120000	-4.10030000
H	-0.55710000	-3.52300000	-3.28840000
H	-0.25740000	-4.99510000	-4.19210000
H	3.22630000	0.44820000	2.39820000
C	-4.07120000	0.02170000	-1.94180000
C	-4.34690000	3.04120000	-2.04520000
C	-3.96490000	2.24800000	-0.80240000
C	-4.64370000	0.87720000	-0.81880000
H	-2.99640000	-0.12260000	-1.79010000
H	-4.52870000	-0.97080000	-1.96330000
H	-3.87610000	4.02830000	-2.04880000
H	-2.87400000	2.09080000	-0.82430000
N	-4.34900000	2.72720000	0.52240000
H	-5.71920000	1.02690000	-1.02610000
N	-4.49330000	0.47690000	0.57710000
H	-5.43140000	3.19410000	-2.07460000

C	-4.35260000	0.76360000	-3.26800000
C	-3.86890000	2.23010000	-3.27110000
H	-5.43350000	0.74490000	-3.45400000
H	-3.88940000	0.22570000	-4.10030000
H	-2.77250000	2.24400000	-3.28840000
H	-4.19710000	2.72050000	-4.19210000
C	3.65460000	3.07280000	1.36620000
C	1.90330000	4.78520000	1.10170000
H	2.23230000	4.91790000	2.13560000
H	2.17110000	5.69440000	0.54770000
C	5.42320000	1.37700000	1.06590000
H	6.36050000	1.39120000	0.50300000
H	5.64640000	1.69260000	2.08730000
C	0.83380000	-4.70130000	1.36620000
C	3.19240000	-4.04090000	1.10170000
H	3.14290000	-4.39220000	2.13560000
H	3.84600000	-4.72740000	0.54770000
C	-1.51910000	-5.38510000	1.06590000
H	-1.97550000	-6.20390000	0.50300000
H	-1.35730000	-5.73620000	2.08730000
C	-4.48840000	1.62860000	1.36620000
C	-3.90410000	4.00810000	1.06590000
H	-4.28900000	4.04360000	2.08730000
H	-4.38500000	4.81280000	0.50300000
C	-5.09580000	-0.74420000	1.10170000
H	-6.01710000	-0.96700000	0.54770000
H	-5.37520000	-0.52570000	2.13560000

	O	0.85440000	-4.80100000	2.58010000
	O	-4.58500000	1.66060000	2.58010000
	O	3.73060000	3.14040000	2.58010000
Conf. 3[c]	C	6.44780000	-0.44470000	0.73840000
	C	4.37100000	-2.02580000	2.28580000
	C	4.73920000	-2.27230000	0.82880000
	C	5.13950000	-0.95890000	0.15270000
	H	7.24690000	-1.17460000	0.56790000
	H	6.75580000	0.49280000	0.26640000
	H	4.06310000	-2.94980000	2.78350000
	H	5.61230000	-2.94930000	0.81460000
	N	3.74810000	-2.77120000	-0.11850000
	H	4.35680000	-0.21260000	0.36700000
	N	5.06340000	-1.33740000	-1.25740000
	H	3.52890000	-1.32920000	2.34940000
	C	1.97540000	1.66000000	-2.31520000
	C	3.79550000	3.16840000	-1.90710000
	C	2.42480000	2.95880000	-2.04730000
	C	4.69600000	2.10630000	-2.00820000
	C	4.24850000	0.81020000	-2.26060000
	C	2.87030000	0.60370000	-2.42700000
	H	0.91230000	1.48320000	-2.43810000
	H	4.16760000	4.16910000	-1.70870000
	H	5.75900000	2.29320000	-1.88830000
	H	2.50760000	-0.39330000	-2.65100000
	C	-4.13190000	2.87520000	1.37130000
	C	-3.75520000	3.37710000	0.12260000

C	-4.09540000	2.64170000	-1.01810000
H	-3.89650000	3.43770000	2.26960000
H	-3.80590000	3.01550000	-1.99410000
C	0.96250000	-4.96210000	1.16810000
C	0.92320000	-2.65700000	0.51190000
C	1.62080000	-3.86220000	0.61390000
H	1.48370000	-5.91040000	1.26290000
C	6.21680000	-0.21740000	2.25000000
C	5.62550000	-1.44480000	2.97700000
H	5.53450000	0.63230000	2.37200000
H	7.15620000	0.07210000	2.72970000
H	6.38930000	-2.23020000	3.02910000
H	5.38920000	-1.17710000	4.01070000
C	-4.80880000	1.45090000	-0.90620000
C	-4.82110000	1.67120000	1.48460000
C	-5.17980000	0.94570000	0.34550000
H	-5.09560000	0.92010000	-1.80870000
H	-5.08190000	1.28330000	2.46210000
C	-2.88770000	-3.82960000	-1.39590000
C	-5.56360000	-2.52290000	-1.95750000
C	-4.61650000	-2.10150000	-0.84100000
C	-3.83490000	-3.30950000	-0.32120000
H	-2.17330000	-3.05060000	-1.67940000
H	-2.30540000	-4.68280000	-1.03950000
H	-6.13480000	-1.67310000	-2.34400000
H	-3.89050000	-1.38410000	-1.25860000
N	-5.15730000	-1.57430000	0.40950000

H	-4.55800000	-4.11430000	-0.09450000
N	-3.32650000	-2.77530000	0.94140000
H	-6.28660000	-3.25270000	-1.57660000
C	-0.36770000	-4.86850000	1.57350000
C	-1.07220000	-3.66960000	1.44760000
C	-0.40020000	-2.56120000	0.92810000
H	-0.86520000	-5.74390000	1.98030000
H	-0.93280000	-1.62300000	0.82300000
C	-3.75510000	-4.24730000	-2.60390000
C	-4.70650000	-3.13400000	-3.08930000
H	-4.34760000	-5.12670000	-2.32270000
H	-3.11090000	-4.56200000	-3.42970000
H	-4.11340000	-2.33380000	-3.54800000
H	-5.35730000	-3.52420000	-3.87710000
H	1.41990000	-1.79240000	0.08930000
C	-0.81260000	4.68440000	2.44140000
C	1.64780000	3.35280000	1.27840000
C	0.65590000	4.18730000	0.47600000
C	-0.75110000	4.04370000	1.06000000
H	-0.56880000	5.75040000	2.37140000
H	-1.81240000	4.60730000	2.87760000
H	2.65800000	3.42880000	0.86950000
H	0.95680000	5.24710000	0.56800000
N	0.40390000	3.90260000	-0.93610000
H	-0.97130000	2.96980000	1.17390000
N	-1.55050000	4.58470000	-0.03410000
H	1.36280000	2.29620000	1.23980000

C	0.20460000	3.94300000	3.33720000
C	1.62180000	3.88010000	2.73090000
H	-0.15880000	2.92190000	3.50260000
H	0.24760000	4.41790000	4.32160000
H	2.06170000	4.88500000	2.74510000
H	2.26100000	3.25680000	3.36300000
C	4.08480000	-2.32430000	-1.39180000
C	5.21940000	-0.35720000	-2.33470000
H	6.24790000	0.01200000	-2.32130000
H	5.07760000	-0.91170000	-3.26410000
C	3.02520000	-4.01830000	0.04970000
H	2.95480000	-4.47340000	-0.94340000
H	3.60890000	-4.70000000	0.68070000
C	-4.26490000	-1.86740000	1.43750000
C	-2.52880000	-3.56670000	1.86380000
H	-2.61870000	-3.07280000	2.83550000
H	-2.95310000	-4.57550000	1.97270000
C	-5.95870000	-0.35160000	0.48450000
H	-6.72580000	-0.41850000	-0.29070000
H	-6.46790000	-0.36730000	1.44910000
C	-0.89930000	4.32900000	-1.23360000
C	1.44090000	4.10700000	-1.94190000
H	1.98620000	5.03990000	-1.73620000
H	0.91410000	4.24040000	-2.89020000
C	-3.00780000	4.69450000	0.00280000
H	-3.29310000	5.20280000	-0.91900000
H	-3.27370000	5.34750000	0.83820000

	O	-4.29850000	-1.41330000	2.56680000
	O	-1.36940000	4.45830000	-2.34950000
	O	3.60400000	-2.72200000	-2.43800000

1a (ACN)

Conf. 1[a]	C	-7.63607100	0.33506900	-0.09306200
	C	-7.16431800	-2.65182900	0.10033600
	C	-6.12675200	-1.58958300	0.43925400
	C	-6.32311800	-0.35609900	-0.43721100
	H	-7.62719500	0.65305200	0.95504100
	H	-7.79524600	1.22647100	-0.70601500
	H	-7.03763900	-3.54870700	0.71298000
	H	-6.27547700	-1.29048400	1.49094500
	N	-4.70027100	-1.85760700	0.22613700
	H	-6.37638500	-0.68660200	-1.48863000
	N	-5.04728000	0.33779400	-0.22832300
	H	-7.06362300	-2.95181800	-0.94829400
	C	-1.82042200	3.96004900	-0.87753900
	C	-2.45281200	3.41506100	1.36896100
	C	-1.59301700	4.08485500	0.49367600
	C	-3.48225600	2.60799700	0.89185500
	C	-3.66549300	2.42839100	-0.47986000
	C	-2.83456200	3.13383400	-1.35473700
	H	-1.16767600	4.46996300	-1.57620000
	H	-2.29719400	3.49895200	2.44017600
	H	-4.11388900	2.07172600	1.59022400
	H	-2.95993300	3.01208300	-2.42629900
	C	4.13803300	0.88058900	-1.35564500

C	3.93550400	1.95487300	-0.48463700
C	3.99683500	1.71105000	0.88804500
H	4.09738500	1.04636700	-2.42782800
H	3.84242800	2.52766000	1.58349000
C	-2.51778000	-3.54747900	-0.89767300
C	-1.73524200	-3.83943800	1.34859100
C	-2.74392200	-3.42580300	0.47405700
H	-3.28489300	-3.23350400	-1.59581000
C	-8.77090400	-0.68225000	-0.34908100
C	-8.55564100	-2.03328300	0.36437500
H	-8.84613900	-0.85499300	-1.42917300
H	-9.72702400	-0.25324900	-0.03690500
H	-8.67341400	-1.89071700	1.44509300
H	-9.33522900	-2.73614700	0.05795000
C	4.18641900	0.41844000	1.36989100
C	4.35236500	-0.40811800	-0.87358500
C	4.34415500	-0.66316300	0.49833500
H	4.17838200	0.24506200	2.44164100
H	4.47286700	-1.23014700	-1.56930500
C	3.52191000	-6.77103000	-0.11459600
C	5.87652000	-4.87788500	0.12053200
C	4.43557400	-4.50931900	0.44922800
C	3.47130800	-5.28484600	-0.44379600
H	3.23219900	-6.93272200	0.92925500
H	2.83334500	-7.34580900	-0.74000400
H	6.58583400	-4.32855000	0.74572800
H	4.24028800	-4.79606400	1.49668800

N	3.95979900	-3.13705500	0.24225000
H	3.79297800	-5.15587700	-1.49138600
N	2.23232800	-4.52657100	-0.23727800
H	6.09652900	-4.63011300	-0.92347300
C	-1.29195100	-4.00387300	-1.37537300
C	-0.26717200	-4.37572600	-0.50032200
C	-0.51895700	-4.32018100	0.87095100
H	-1.12047800	-4.04150400	-2.44680700
H	0.25944200	-4.60402100	1.56931500
C	4.97113000	-7.24664800	-0.36280400
C	6.02964000	-6.39559700	0.36948300
H	5.16797700	-7.21433000	-1.44094800
H	5.07195100	-8.29277000	-0.06099800
H	5.95477900	-6.58032300	1.44766000
H	7.02991500	-6.71909000	0.06867900
H	-1.88957300	-3.75730800	2.42011200
C	4.10577900	6.43621600	-0.11362100
C	1.28494600	7.52230400	0.10295400
C	1.68900000	6.09237800	0.43782900
C	2.84780000	5.64589700	-0.44844500
H	4.38434300	6.26987900	0.93254600
H	4.95248300	6.12718700	-0.73274600
H	0.45082300	7.86129000	0.72355500
H	2.03110800	6.07090000	1.48664500
N	0.74165200	4.99137700	0.23097300
H	2.58044100	5.85721900	-1.49792400
N	2.81261500	4.19436200	-0.23824000

H	0.96431600	7.58488700	-0.94250700
C	3.79122400	7.92783800	-0.36824100
C	2.51888300	8.41857400	0.35404500
H	3.67139800	8.07851200	-1.44766600
H	4.64361200	8.54089500	-0.06288800
H	2.71004800	8.45225600	1.43318900
H	2.29757000	9.44442700	0.04688700
C	-4.06885000	-0.63265800	-0.00109500
C	-4.73208500	1.51019000	-1.04693100
H	-4.43388300	1.20202000	-2.05825300
H	-5.66623000	2.06885100	-1.15428700
C	-4.03984100	-2.87833000	1.04219200
H	-3.85041900	-2.49580800	2.05428900
H	-4.75760300	-3.69683300	1.14801700
C	2.58500700	-3.19669300	0.00296900
C	1.06451700	-4.83223200	-1.06592900
H	1.18770700	-4.41008900	-2.07260700
H	1.04866100	-5.91957600	-1.18358600
C	4.50764400	-2.05957300	1.06828800
H	4.06801200	-2.08717100	2.07454400
H	5.57328400	-2.27499200	1.18742100
C	1.48342800	3.83227600	-0.00825300
C	-0.46360600	4.92506600	1.05956800
H	-0.80975500	5.95552800	1.18017000
H	-0.21698900	4.55827900	2.06513300
C	3.67166800	3.33550000	-1.05566400
H	4.62247800	3.86576600	-1.16108200

	H	3.25781600	3.23064300	-2.06780200
	O	1.82050900	-2.24645100	0.00345800
	O	1.04461200	2.69422500	-0.01053900
	O	-2.86431000	-0.44096300	0.00077300
Conf. 2[b]	C	-1.48547400	-3.82356800	-1.95029300
	C	-4.40499900	-2.99560200	-2.03577200
	C	-3.51581800	-2.91689700	-0.80237600
	C	-2.48313000	-4.04305000	-0.82129700
	H	-0.96382000	-2.87084300	-1.81127900
	H	-0.72833300	-4.61144000	-1.97401600
	H	-5.15784800	-2.20283200	-2.03881300
	H	-2.97836900	-1.95565500	-0.83226700
	N	-4.08796500	-3.09335600	0.53369800
	H	-3.00782000	-4.99432900	-1.01569700
	N	-2.04673700	-4.04247100	0.57769700
	H	-4.93470000	-3.95428100	-2.05616800
	C	2.63736200	-4.91416900	0.36701900
	C	2.06521600	-3.05528100	1.77589000
	C	3.03768400	-3.77620400	1.06697500
	C	0.73555600	-3.45797100	1.76861500
	C	0.33423200	-4.60014300	1.06360900
	C	1.30228800	-5.32486300	0.36981900
	H	3.37214200	-5.48887200	-0.18786500
	H	2.35846800	-2.18066400	2.34625900
	H	-0.00205900	-2.88673600	2.32198500
	H	1.01513500	-6.21483900	-0.18099200
	C	3.96157100	3.79079400	0.37243500

C	3.81766300	2.58951700	1.06523100
C	2.62762100	2.36536300	1.76959700
H	4.87616400	3.98784400	-0.17770300
H	2.50178500	1.44045400	2.32223100
C	-5.57548600	0.17330300	0.36685000
C	-3.68073900	-0.26067600	1.77752800
C	-4.79063900	-0.74248900	1.06761100
H	-6.44005500	-0.17568000	-0.18892000
C	-2.29056900	-3.82657400	-3.27006900
C	-3.48408200	-2.84730100	-3.26885500
H	-2.66225700	-4.84298300	-3.44612800
H	-1.62758000	-3.59094200	-4.10711400
H	-3.10276900	-1.81958400	-3.29434200
H	-4.06842700	-2.98309800	-4.18306300
C	1.61363800	3.31499300	1.77653600
C	2.93792700	4.74138400	0.36957100
C	1.75167700	4.51801800	1.06841600
H	0.70931900	3.13074600	2.34615900
H	3.06839300	5.66543400	-0.18474500
C	-2.56938800	3.19879900	-1.95128500
C	-0.39197900	5.31241400	-2.03548000
C	-0.76856800	4.50217100	-0.80265600
C	-2.26024000	4.17158800	-0.82151600
H	-2.00477200	2.27062700	-1.81365500
H	-3.63024000	2.93683200	-1.97482400
H	0.67116300	5.56736000	-2.03863600
H	-0.20529300	3.55574800	-0.83322200

N	-0.63514300	5.08508800	0.53386600
H	-2.82122300	5.10196000	-1.01539100
N	-2.47806000	3.79276900	0.57727800
H	-0.95664400	6.25096700	-2.05477700
C	-5.26344100	1.53485500	0.36951900
C	-4.15221600	2.01081000	1.06403800
C	-3.36449300	1.09213000	1.77007200
H	-5.89006900	2.22841900	-0.18209400
H	-2.50120200	1.44550900	2.32371800
C	-2.17021300	3.89906600	-3.27044200
C	-0.72521100	4.44234200	-3.26945600
H	-2.86436700	4.72971000	-3.44492100
H	-2.29851900	3.20819500	-4.10827200
H	-0.02636900	3.59791800	-3.29647600
H	-0.55091200	5.01729300	-4.18310400
H	-3.07037500	-0.95175500	2.34862700
C	4.05580500	0.62614300	-1.95009900
C	4.79882100	-2.31601500	-2.03605400
C	4.28491600	-1.58580600	-0.80287200
C	4.74393400	-0.12838000	-0.82091900
H	2.96972700	0.60106000	-1.81222200
H	4.35931400	1.67584800	-1.97302000
H	4.48904400	-3.36449600	-2.03985600
H	3.18378200	-1.60081300	-0.83380300
N	4.72283000	-1.99329100	0.53348500
H	5.83013000	-0.10708900	-1.01488900
N	4.52447900	0.24898000	0.57811300

H	5.89390100	-2.29501300	-2.05544400
C	4.46217400	-0.06882800	-3.26987000
C	4.21094000	-1.59222100	-3.26946100
H	5.52840400	0.11744000	-3.44495100
H	3.92731000	0.38796700	-4.10710600
H	3.13032100	-1.77569800	-3.29606700
H	4.62158500	-2.03005000	-4.18344300
C	-3.11362500	-3.62395200	1.36484600
C	-1.11583200	-5.04470100	1.08940500
H	-1.41756100	-5.25191700	2.11902000
H	-1.23021900	-5.97322500	0.51807300
C	-5.13172200	-2.22237500	1.06953200
H	-6.04860200	-2.38687600	0.49901000
H	-5.31512100	-2.56607900	2.08977800
C	-1.58172800	4.50644200	1.36475100
C	-3.81143400	3.48869400	1.08966500
H	-3.83908900	3.85334900	2.11929200
H	-4.55847100	4.05271400	0.51915500
C	0.64054100	5.55302900	1.07072600
H	0.95670600	6.43001700	0.50144300
H	0.43370300	5.88227300	2.09122300
C	4.69476200	-0.88446400	1.36488800
C	4.48999200	-3.33257200	1.06909900
H	4.87935900	-3.31952200	2.08926400
H	5.09046900	-4.04468300	0.49849000
C	4.92731100	1.55577400	1.09129100
H	5.78959500	1.92072000	0.52136500

	H	5.25627100	1.39723400	2.12118000
	O	-1.62368600	4.60762600	2.58753600
	O	4.80300600	-0.89872600	2.58768800
	O	-3.18039300	-3.71125500	2.58746300
Conf. 3[c]	C	6.45420100	-0.57277900	0.72588800
	C	4.38094600	-2.13601000	2.29878900
	C	4.72923600	-2.38122300	0.83748300
	C	5.13077900	-1.07025900	0.16091900
	H	7.24131500	-1.31260600	0.54416100
	H	6.76622600	0.36217100	0.25271200
	H	4.07366200	-3.05956800	2.79735200
	H	5.59188600	-3.06809600	0.80854600
	N	3.72052800	-2.87312500	-0.10249800
	H	4.35956200	-0.31676000	0.38738600
	N	5.02312700	-1.44335100	-1.25263900
	H	3.54995100	-1.42727600	2.37603700
	C	2.00166000	1.63506900	-2.29427400
	C	3.86724500	3.10257200	-1.93991500
	C	2.48914200	2.92707000	-2.06046800
	C	4.73831300	2.01473400	-2.03165500
	C	4.25328100	0.72561600	-2.25286400
	C	2.86779600	0.55278600	-2.39335500
	H	0.93319300	1.47989300	-2.39933100
	H	4.26855600	4.09511200	-1.76140100
	H	5.80636500	2.17587600	-1.92506800
	H	2.47166700	-0.43805100	-2.58495600
	C	-4.06540900	3.00490100	1.37575300

C	-3.67568600	3.44712200	0.10830800
C	-4.01607800	2.66251800	-1.00013700
H	-3.83409900	3.60489000	2.24988100
H	-3.72693200	2.99177500	-1.99199800
C	0.89146900	-5.06763700	1.10101000
C	0.89738400	-2.74264400	0.51201300
C	1.57737600	-3.95935300	0.59530000
H	1.39801900	-6.02499700	1.17862200
C	6.24610900	-0.34805900	2.24152200
C	5.65227800	-1.57093800	2.97336000
H	5.57552600	0.50874900	2.37557100
H	7.19647700	-0.07235300	2.70676200
H	6.40657800	-2.36566500	3.01201800
H	5.43136600	-1.30253500	4.01004000
C	-4.73636900	1.48075800	-0.84337600
C	-4.76722800	1.81172900	1.53415100
C	-5.12014000	1.03404500	0.42770300
H	-5.01956000	0.91464900	-1.72436700
H	-5.05328600	1.48387700	2.52697700
C	-3.10813700	-3.86006100	-1.42330700
C	-5.74267800	-2.41701200	-1.84803500
C	-4.71658400	-2.04784100	-0.78497200
C	-3.97415600	-3.29487900	-0.30512100
H	-2.37004600	-3.11715300	-1.74223900
H	-2.55964100	-4.74612200	-1.09420300
H	-6.28933700	-1.53917100	-2.20403800
H	-3.97823100	-1.36871500	-1.24092000

N	-5.15658100	-1.48939300	0.49588100
H	-4.72115300	-4.06224200	-0.03809700
N	-3.37499500	-2.79098900	0.93419200
H	-6.47791600	-3.11432300	-1.43196600
C	-0.44410400	-4.96631600	1.48650700
C	-1.12876800	-3.75194300	1.38697400
C	-0.43422400	-2.64017300	0.90605500
H	-0.96061700	-5.84545400	1.85921100
H	-0.94832500	-1.68986000	0.81882600
C	-4.05613800	-4.22640100	-2.58770800
C	-4.97293200	-3.06380600	-3.02159000
H	-4.67688100	-5.07583600	-2.27857700
H	-3.47002000	-4.56759400	-3.44551200
H	-4.36484500	-2.29097200	-3.50659000
H	-5.68139300	-3.41832900	-3.77534000
H	1.41417600	-1.87035700	0.13105300
C	-0.73676700	4.88239100	2.36902700
C	1.74134800	3.51793800	1.27699500
C	0.74224900	4.30226900	0.43577400
C	-0.66303300	4.17245900	1.02326400
H	-0.50263900	5.94544000	2.24614700
H	-1.73597900	4.81598200	2.80728900
H	2.75147500	3.58865700	0.86727500
H	1.03390900	5.36610600	0.47216200
N	0.49394000	3.94900600	-0.96640000
H	-0.87306700	3.10384500	1.18956400
N	-1.46819800	4.64454800	-0.10340900

H	1.46728500	2.45780900	1.29481800
C	0.28799700	4.19723600	3.30096700
C	1.70693300	4.11919900	2.70073000
H	-0.06495000	3.18212200	3.51784600
H	0.32433400	4.72389500	4.25859200
H	2.13574900	5.12768100	2.66264900
H	2.35104400	3.53403600	3.36293600
C	4.04156000	-2.42115300	-1.37083300
C	5.19757700	-0.46435000	-2.32801300
H	6.23247000	-0.11652000	-2.31280900
H	5.04753700	-1.00892800	-3.26188400
C	3.00020400	-4.12117000	0.07926600
H	2.96550300	-4.61904900	-0.89438500
H	3.56911700	-4.77146400	0.75233600
C	-4.23314600	-1.83603200	1.46975300
C	-2.58275200	-3.63964000	1.81178500
H	-2.65057700	-3.19576200	2.80864700
H	-3.02806400	-4.64121400	1.87291800
C	-5.91959600	-0.24564800	0.61489300
H	-6.72992400	-0.29449700	-0.11559300
H	-6.37709300	-0.24370400	1.60489100
C	-0.80649700	4.34953500	-1.28096100
C	1.54056900	4.10690000	-1.97310800
H	2.11109300	5.02393700	-1.77592000
H	1.02607000	4.24148200	-2.92741700
C	-2.92573900	4.75800600	-0.06625600
H	-3.21953000	5.23110600	-1.00376100

	H	-3.18611000	5.44220100	0.74433000
	O	-4.18462200	-1.38149800	2.60670700
	O	-1.27505800	4.43185800	-2.41078700
	O	3.54448200	-2.81912600	-2.41923200

1a ($CHCl_3$)

Conf. 1[a]	C	-5.75839500	-5.01923600	0.08234400
	C	-7.27048000	-2.40174900	-0.10583300
	C	-5.79482600	-2.57107900	-0.44467700
	C	-5.16998500	-3.65707500	0.42758400
	H	-5.55210400	-5.25976100	-0.96622500
	H	-5.31992700	-5.81386400	0.69272100
	H	-7.73727100	-1.62317000	-0.71586600
	H	-5.72232700	-2.89435400	-1.49804700
	N	-4.85858300	-1.46644500	-0.22365400
	H	-5.42004200	-3.43510900	1.48008700
	N	-3.74385600	-3.39771500	0.21528000
	H	-7.38049000	-2.10644100	0.94324500
	C	1.03974000	-4.20462900	0.89025300
	C	0.21737300	-4.18643500	-1.35993600
	C	1.30192500	-4.17397200	-0.47938000
	C	-1.09285200	-4.19101900	-0.89031400
	C	-1.35463800	-4.15758200	0.47931900
	C	-0.27033300	-4.18388500	1.35987400
	H	1.86500800	-4.18734200	1.59233900
	H	0.39719000	-4.15630700	-2.43024100
	H	-1.91783800	-4.16317500	-1.59239600
	H	-0.44977100	-4.15188600	2.43018900

C	3.79317500	1.88091500	1.33962200
C	4.31631000	0.92440000	0.46616200
C	4.21196200	1.15746900	-0.90521500
H	3.85679600	1.71837200	2.41121900
H	4.60318600	0.42638600	-1.60286200
C	-4.19675700	1.21028900	0.90533400
C	-3.76884600	1.92816800	-1.33958000
C	-4.30405200	0.97844500	-0.46602900
H	-4.59720500	0.48427600	1.60304200
C	-7.28026000	-4.94366400	0.34010900
C	-7.96369000	-3.75715200	-0.37121800
H	-7.44619400	-4.85658800	1.42059000
H	-7.75468600	-5.87863400	0.02901600
H	-7.96679900	-3.94117700	-1.45222500
H	-9.01199000	-3.70208200	-0.06444800
C	3.54438800	2.28136300	-1.38304300
C	3.14723500	3.01735800	0.86168600
C	2.98447800	3.21659900	-0.50915600
H	3.42503300	2.41183700	-2.45427700
H	2.71497800	3.72469000	1.55943800
C	-1.46155700	7.48455300	0.13237800
C	1.55577700	7.46565800	-0.13232400
C	0.65536500	6.27843400	-0.45086000
C	-0.57609700	6.28610900	0.45078100
H	-1.79972300	7.43189800	-0.90822200
H	-2.35320000	7.50643500	0.76552700
H	2.44761200	7.47644600	-0.76548900

H	0.31377800	6.38032400	-1.49594200
N	1.14102900	4.91217700	-0.24231900
H	-0.23324100	6.38359800	1.49586500
N	-1.07884600	4.92607100	0.24211300
H	1.89328200	7.40863600	0.90826100
C	-3.51501200	2.32567900	1.38307100
C	-2.94337200	3.25369700	0.50909800
C	-3.10860400	3.05640500	-0.86173400
H	-3.39402100	2.45473800	2.45429200
H	-2.66745000	3.75816500	-1.55954300
C	-0.61988400	8.75780200	0.37331400
C	0.73013000	8.74939200	-0.37308100
H	-0.43329900	8.85371500	1.44970700
H	-1.19417500	9.64116400	0.07997900
H	0.54476600	8.84778800	-1.44946100
H	1.31543500	9.62545400	-0.07962200
H	-3.83452400	1.76634800	-2.41116200
C	7.23949700	-2.49305600	0.10582600
C	5.69434300	-5.09112900	-0.08257200
C	5.12323600	-3.72156600	-0.42760300
C	5.76183900	-2.64370800	0.44474900
H	7.35317500	-2.19903700	-0.94322300
H	7.71615500	-1.72052700	0.71592300
H	5.24578400	-5.88005100	-0.69301900
H	5.37603400	-3.50265500	-1.48009100
N	3.70052500	-3.44418300	-0.21518700
H	5.68531700	-2.96615200	1.49808900

N	4.83964600	-1.52729100	0.22389100
H	5.48508200	-5.32914900	0.96598000
C	7.91549300	-3.85716700	0.37099700
C	7.21702800	-5.03484100	-0.34042600
H	7.91634400	-4.04134900	1.45198200
H	8.96438700	-3.81534500	0.06416200
H	7.38399100	-4.94975300	-1.42091000
H	7.67957600	-5.97579100	-0.02947300
C	-3.59073600	-2.02172400	-0.00425100
C	-2.76376600	-4.10713900	1.04021200
H	-2.72702000	-3.67274000	2.04885400
H	-3.14033200	-5.12849200	1.15225700
C	-4.98353800	-0.25362200	-1.03417500
H	-4.60248700	-0.43068400	-2.04960800
H	-6.05601000	-0.05895500	-1.13101600
C	0.02592500	4.09819600	-0.00019700
C	-2.17702100	4.43486700	1.07594400
H	-1.80937500	4.17057000	2.07714200
H	-2.86306300	5.27736900	1.20630400
C	2.23304500	4.40728900	-1.07609200
H	1.86219700	4.14763600	-2.07732500
H	2.92960800	5.24112400	-1.20638700
C	3.56486100	-2.06643100	0.00459300
C	2.71157200	-4.14102800	-1.04026300
H	3.07528900	-5.16700000	-1.15255900
H	2.68029000	-3.70596500	-2.04880500
C	4.98009000	-0.31615600	1.03439200

	H	6.05495900	-0.13514300	1.13115000
	H	4.59687700	-0.48831100	2.04985300
	O	0.01828100	2.88397200	-0.00032300
	O	2.52139600	-1.44565100	0.00295300
	O	-2.53936200	-1.41413800	-0.00234700
Conf. 2[b]	C	3.93086200	-1.24054300	-1.94581300
	C	3.26576800	-4.20050900	-2.02974600
	C	3.13774000	-3.31754300	-0.79601900
	C	4.20309300	-2.22134900	-0.81286600
	H	2.95045300	-0.77246400	-1.80950200
	H	4.67404600	-0.43939900	-1.96966100
	H	2.51518300	-4.99573000	-2.03142800
	H	2.14769100	-2.83473600	-0.82615000
	N	3.34755400	-3.88000800	0.53794500
	H	5.18291300	-2.69311600	-1.00457700
	N	4.17128400	-1.78461300	0.58400200
	H	4.25182600	-4.67736500	-2.05045000
	C	4.73427900	2.93667000	0.34208000
	C	2.93769500	2.26559900	1.78774800
	C	3.58455700	3.27257500	1.05658700
	C	3.42306900	0.96415400	1.78707700
	C	4.57630900	0.62696400	1.06642700
	C	5.22791600	1.63018500	0.35140200
	H	5.25467400	3.70055300	-0.22719100
	H	2.05717200	2.51157100	2.37097500
	H	2.91083700	0.19818800	2.35954000
	H	6.12653600	1.39341700	-0.20976400

C	-4.02592100	3.70936000	0.37812400
C	-2.81808100	3.63913500	1.06983600
C	-2.52358700	2.46724400	1.77875100
H	-4.27853800	4.60996200	-0.17273700
H	-1.59369600	2.40098200	2.33317700
C	0.18062300	-5.55904600	0.35513100
C	0.48803300	-3.64895400	1.77789700
C	1.04140400	-4.72191400	1.06415900
H	0.58623000	-6.39847000	-0.20122800
C	3.98132400	-2.04679000	-3.26411700
C	3.06713100	-3.28997800	-3.26312000
H	5.01643000	-2.36503500	-3.43707100
H	3.71369400	-1.39898300	-4.10360400
H	2.02045700	-2.96428200	-3.28775900
H	3.23334600	-3.86526700	-4.17817700
C	-3.41117400	1.39889500	1.79062700
C	-4.91426600	2.63179100	0.38043200
C	-4.62041500	1.46349600	1.08321900
H	-3.17565900	0.50923000	2.36423700
H	-5.84626900	2.70577000	-0.17135600
C	-3.04917300	-2.74586500	-1.95997800
C	-5.29786600	-0.70935700	-2.02352100
C	-4.46077800	-1.03941500	-0.79514300
C	-4.03407500	-2.50701900	-0.82306000
H	-2.15898900	-2.12323900	-1.82240800
H	-2.71742800	-3.78699200	-1.99070600
H	-5.62175600	0.33510200	-2.01814000

H	-3.55242900	-0.41647600	-0.82600000
N	-5.04725200	-0.95045100	0.54175900
H	-4.92813400	-3.12508000	-1.01757800
N	-3.63619900	-2.70613800	0.57148100
H	-6.19809500	-1.33337100	-2.04334100
C	-1.19770000	-5.33433600	0.35377200
C	-1.74583500	-4.26055300	1.05298500
C	-0.88194400	-3.42037200	1.76739400
H	-1.84844500	-6.00168100	-0.20251500
H	-1.29331100	-2.58766200	2.32761900
C	-3.77951500	-2.38496600	-3.27430300
C	-4.41362800	-0.97749600	-3.26252600
H	-4.56527300	-3.12929000	-3.45056400
H	-3.08571800	-2.46422200	-4.11595200
H	-3.61551100	-0.22617000	-3.28771600
H	-5.00099700	-0.83483100	-4.17393100
H	1.13728300	-3.00161400	2.35713100
C	-0.88297900	3.96588700	-1.94557300
C	2.00224200	4.90211800	-2.05584900
C	1.31451600	4.35622200	-0.81185200
C	-0.17137000	4.71594400	-0.82715100
H	-0.78647400	2.88579900	-1.79377600
H	-1.95104600	4.19738000	-1.96761000
H	3.06956200	4.66442400	-2.05996300
H	1.40462200	3.25825400	-0.82946400
N	1.69800000	4.83845400	0.51485100
H	-0.26457800	5.79628500	-1.03547900

N	-0.52656500	4.48871300	0.57441100
H	1.90729300	5.99301900	-2.09000900
C	-0.22132000	4.39992700	-3.27383200
C	1.31518600	4.25002200	-3.27750400
H	-0.47786000	5.44928300	-3.46294800
H	-0.64506200	3.82528500	-4.10228700
H	1.57039100	3.18375800	-3.28920600
H	1.72060700	4.67470500	-4.20016800
C	3.81633300	-2.87525200	1.37299800
C	5.10940400	-0.79261000	1.10158100
H	5.32314300	-1.07799700	2.13445100
H	6.04958900	-0.85216200	0.54028200
C	2.54032400	-4.97309600	1.07402500
H	2.76242000	-5.88037400	0.50704500
H	2.89098100	-5.13089300	2.09600600
C	-4.40306700	-1.86080700	1.36853500
C	-3.24284700	-4.01705400	1.07973800
H	-3.60304600	-4.07047900	2.11009600
H	-3.75760800	-4.79889500	0.50835600
C	-5.58888000	0.29186800	1.08800300
H	-6.48675600	0.55735300	0.52473300
H	-5.89761100	0.06015100	2.10946400
C	0.59714900	4.74443900	1.35510800
C	3.04997600	4.69554400	1.05072100
H	3.01160200	5.09019900	2.06807400
H	3.72205200	5.33644000	0.47485500
C	-1.85298400	4.80897700	1.09367200

	H	-2.27306200	5.65023300	0.52897700
	H	-1.70788900	5.14297800	2.12402700
	O	-4.49118800	-1.91506200	2.58827400
	O	0.60861100	4.86279800	2.57308300
	O	3.90098900	-2.93259600	2.59288000
Conf. 3[c]	C	6.44543600	-0.52496400	0.74232100
	C	4.35653600	-2.09213400	2.28910900
	C	4.72326900	-2.33821800	0.83216400
	C	5.13123100	-1.02673200	0.15957300
	H	7.23727600	-1.26214000	0.57030200
	H	6.76077500	0.41074800	0.27244500
	H	4.04315600	-3.01510900	2.78498100
	H	5.58884400	-3.02281300	0.81546700
	N	3.72838600	-2.83149200	-0.11930900
	H	4.35476000	-0.27540800	0.37596800
	N	5.04568700	-1.40060300	-1.25398200
	H	3.52241300	-1.38603100	2.35481900
	C	2.00633600	1.65390300	-2.29399400
	C	3.85635800	3.13429500	-1.91453400
	C	2.48065900	2.94726000	-2.04234700
	C	4.73779700	2.05563700	-2.01385600
	C	4.26560000	0.76473500	-2.25032000
	C	2.88264500	0.58099800	-2.40138900
	H	0.93979300	1.49153400	-2.40610600
	H	4.24761200	4.12907000	-1.72502900
	H	5.80423500	2.22525100	-1.90193000
	H	2.49759600	-0.41117200	-2.60827900

C	-4.10421900	2.96347300	1.36158800
C	-3.70593400	3.42601400	0.10421800
C	-4.03793700	2.65953900	-1.01893200
H	-3.87822500	3.54960500	2.24662200
H	-3.74043400	3.00393600	-2.00315600
C	0.91744100	-5.00990200	1.13943800
C	0.89991700	-2.70130000	0.49162700
C	1.58870900	-3.91147200	0.59582700
H	1.43125600	-5.96194100	1.23480700
C	6.21800200	-0.29992200	2.25494600
C	5.61775500	-1.52391200	2.97959700
H	5.54354100	0.55523200	2.38039100
H	7.16137400	-0.02110300	2.73266500
H	6.37391300	-2.31655000	3.02810700
H	5.38385500	-1.25618700	4.01366900
C	-4.75850100	1.47522200	-0.88455600
C	-4.80452200	1.76728200	1.49764800
C	-5.14926000	1.00769400	0.37642600
H	-5.03538700	0.92198800	-1.77592000
H	-5.09087500	1.41850000	2.48297900
C	-3.01744500	-3.85178500	-1.40498700
C	-5.67130000	-2.47038300	-1.89909300
C	-4.67820500	-2.07002900	-0.81556200
C	-3.91942000	-3.29721500	-0.30964900
H	-2.28877200	-3.09588300	-1.71396200
H	-2.45466800	-4.72174700	-1.05775100
H	-6.22894400	-1.60750100	-2.27487700

H	-3.94453600	-1.37788100	-1.25979600
N	-5.16187300	-1.51467000	0.44946500
H	-4.65702000	-4.07861400	-0.05493900
N	-3.36011000	-2.77154500	0.93787900
H	-6.40147400	-3.17876100	-1.49266300
C	-0.41456800	-4.90770600	1.53732700
C	-1.10893900	-3.70180000	1.41332700
C	-0.42672100	-2.59730600	0.89890800
H	-0.92068000	-5.78078700	1.93799400
H	-0.94941400	-1.65366500	0.79323800
C	-3.93112100	-4.24945000	-2.58588600
C	-4.86342900	-3.11132400	-3.05027100
H	-4.53996700	-5.10998700	-2.28299500
H	-3.31919400	-4.58525600	-3.42761900
H	-4.26128800	-2.33039500	-3.52977700
H	-5.54770000	-3.48727200	-3.81606700
H	1.40553800	-1.83659900	0.07980300
C	-0.77481800	4.76415200	2.40929600
C	1.69603300	3.41371400	1.28724900
C	0.71023800	4.23580500	0.46590200
C	-0.70030600	4.09942600	1.04044800
H	-0.52994000	5.82853400	2.32354600
H	-1.77756200	4.69327000	2.83893700
H	2.70942300	3.48585500	0.88585700
H	1.01101500	5.29571800	0.54175900
N	0.46845700	3.93252900	-0.94741600
H	-0.92083900	3.02783600	1.16952200

N	-1.49336700	4.61838700	-0.07253900
H	1.41102800	2.35665800	1.26611600
C	0.23654300	4.03794300	3.32436100
C	1.65845600	3.96608800	2.73049300
H	-0.12753100	3.01960800	3.50396900
H	0.27171800	4.53067500	4.30004000
H	2.09773600	4.97100200	2.73025700
H	2.29243100	3.35252900	3.37679500
C	4.06658700	-2.38208600	-1.38624400
C	5.21879400	-0.41776700	-2.32640100
H	6.25162800	-0.06317200	-2.30671700
H	5.07465200	-0.96239300	-3.26110400
C	3.00122300	-4.07637400	0.05397600
H	2.94565600	-4.55143900	-0.93015500
H	3.57521200	-4.74613600	0.70444400
C	-4.25313000	-1.83346900	1.44931300
C	-2.56266900	-3.58955300	1.83859700
H	-2.63549000	-3.11720000	2.82203400
H	-2.99929500	-4.59362100	1.92872800
C	-5.94206200	-0.27900500	0.53986000
H	-6.72706000	-0.33814700	-0.21718200
H	-6.43129700	-0.28056500	1.51467600
C	-0.82793700	4.35531400	-1.25776200
C	1.52020000	4.11630100	-1.94331000
H	2.08173800	5.03598300	-1.72882500
H	1.00967500	4.26161500	-2.89827200
C	-2.95054900	4.73697000	-0.04254300

	H	-3.23402100	5.22756400	-0.97427600
	H	-3.21439600	5.40787200	0.77844800
	O	-4.24281800	-1.37222600	2.58200200
	O	-1.28809500	4.47913300	-2.38459400
	O	3.58606800	-2.78079300	-2.43891800

1b (*in vacuo*)

Conf. 1[a]	C	-1.50548300	7.42552900	0.15743600
	C	1.50548300	7.42552900	-0.15743600
	C	0.60612900	6.23333700	-0.46029600
	C	-0.60612900	6.23333700	0.46029600
	H	-1.86191100	7.37008200	-0.87681400
	H	-2.38696300	7.44294800	0.80445700
	H	2.38696300	7.44294800	-0.80445700
	H	0.24499300	6.33304900	-1.50027100
	N	1.09748500	4.86622100	-0.26568500
	H	-0.24499300	6.33304900	1.50027100
	N	-1.09748500	4.86622100	0.26568500
	H	1.86191100	7.37008200	0.87681400
	C	-4.32960600	1.24931000	0.86991900
	C	-3.88515500	1.99254000	-1.36291800
	C	-4.38552600	1.01355500	-0.50227900
	C	-3.24673700	3.12489400	-0.86991900
	C	-3.07052700	3.29120000	0.50227900
	C	-3.66816800	2.36837300	1.36291800
	H	-4.71367600	0.50432000	1.55685600
	H	-3.92228600	1.83193600	-2.43610400
	H	-2.79359200	3.83000300	-1.55685600

H	-3.54764600	2.48083100	2.43610400
C	-0.21698700	-4.36091300	1.36291800
C	-1.31499900	-4.30475500	0.50227900
C	-1.08286900	-4.37420300	-0.86991900
H	-0.37463900	-4.31276700	2.43610400
H	-1.92008400	-4.33432300	-1.55685600
C	3.24673700	3.12489400	0.86991900
C	3.66816800	2.36837300	-1.36291800
C	3.07052700	3.29120000	-0.50227900
H	2.79359200	3.83000300	1.55685600
C	-0.66895200	8.70503500	0.38406900
C	0.66895200	8.70503500	-0.38406900
H	-0.46513800	8.80259300	1.45720300
H	-1.25375900	9.58488100	0.10082600
H	0.46513800	8.80259300	-1.45720300
H	1.25375900	9.58488100	-0.10082600
C	0.21698700	-4.36091300	-1.36291800
C	1.08286900	-4.37420300	0.86991900
C	1.31499900	-4.30475500	-0.50227900
H	0.37463900	-4.31276700	-2.43610400
H	1.92008400	-4.33432300	1.55685600
C	7.18343800	-2.40897800	0.15743600
C	5.67795500	-5.01655100	-0.15743600
C	5.09516400	-3.64159100	-0.46029600
C	5.70129200	-2.59174500	0.46029600
H	7.31363400	-2.07257800	-0.87681400
H	7.63926400	-1.65430300	0.80445700

H	5.25230100	-5.78864500	-0.80445700
H	5.36208500	-3.37869500	-1.50027100
N	3.66552800	-3.38356000	-0.26568500
H	5.60707800	-2.95435400	1.50027100
N	4.76301300	-1.48266000	0.26568500
H	5.45172300	-5.29750400	0.87681400
C	3.88515500	1.99254000	1.36291800
C	4.38552600	1.01355500	0.50227900
C	4.32960600	1.24931000	-0.86991900
H	3.92228600	1.83193600	2.43610400
H	4.71367600	0.50432000	-1.55685600
C	7.87325800	-3.77318800	0.38406900
C	7.20430500	-4.93184700	-0.38406900
H	7.85583800	-3.99847500	1.45720300
H	8.92762900	-3.70665400	0.10082600
H	7.39070000	-4.80411800	-1.45720300
H	7.67387100	-5.87822700	-0.10082600
H	3.54764600	2.48083100	-2.43610400
C	-5.67795500	-5.01655100	0.15743600
C	-7.18343800	-2.40897800	-0.15743600
C	-5.70129200	-2.59174500	-0.46029600
C	-5.09516400	-3.64159100	0.46029600
H	-5.45172300	-5.29750400	-0.87681400
H	-5.25230100	-5.78864500	0.80445700
H	-7.63926400	-1.65430300	-0.80445700
H	-5.60707800	-2.95435400	-1.50027100
N	-4.76301300	-1.48266000	-0.26568500

H	-5.36208500	-3.37869500	1.50027100
N	-3.66552800	-3.38356000	0.26568500
H	-7.31363400	-2.07257800	0.87681400
C	-7.20430500	-4.93184700	0.38406900
C	-7.87325800	-3.77318800	-0.38406900
H	-7.39070000	-4.80411800	1.45720300
H	-7.67387100	-5.87822700	0.10082600
H	-7.85583800	-3.99847500	-1.45720300
H	-8.92762900	-3.70665400	-0.10082600
C	0.00000000	4.04739000	0.00000000
S	0.00000000	2.39361300	0.00000000
C	-2.21935200	4.40323900	1.09063200
H	-1.84383300	4.06965000	2.06731800
H	-2.84342800	5.28438800	1.26871600
C	2.21935200	4.40323900	-1.09063200
H	1.84383300	4.06965000	-2.06731800
H	2.84342800	5.28438800	-1.26871600
C	3.50514300	-2.02369500	0.00000000
S	2.07293000	-1.19680600	0.00000000
C	4.92299300	-0.27960400	1.09063200
H	4.44633700	-0.43801800	2.06731800
H	5.99812900	-0.17971300	1.26871600
C	2.70364100	-4.12363500	-1.09063200
H	2.60250300	-3.63163100	-2.06731800
H	3.15470000	-5.10467500	-1.26871600
C	-3.50514300	-2.02369500	0.00000000
S	-2.07293000	-1.19680600	0.00000000

	C	-4.92299300	-0.27960400	-1.09063200
	H	-5.99812900	-0.17971300	-1.26871600
	H	-4.44633700	-0.43801800	-2.06731800
	C	-2.70364100	-4.12363500	1.09063200
	H	-3.15470000	-5.10467500	1.26871600
	H	-2.60250300	-3.63163100	2.06731800
Conf. 2[b]	C	3.02519700	2.70361300	-2.09083000
	C	5.08965200	0.47952500	-2.21977300
	C	4.35048600	0.91238400	-0.96098700
	C	4.05090200	2.40408600	-1.00630200
	H	2.09564800	2.16239500	-1.88735200
	H	2.77761800	3.76712300	-2.13083400
	H	5.31784200	-0.58936400	-2.20226400
	H	3.39464700	0.36537900	-0.92362000
	N	4.99096300	0.80455300	0.35193200
	H	4.98224000	2.93912100	-1.26580800
	N	3.76640000	2.66887800	0.40651900
	H	6.03901200	1.01979300	-2.30394200
	C	0.00000000	5.62893700	0.19275100
	C	-0.34973100	3.62203200	1.46376000
	C	-0.88029600	4.75096000	0.82458700
	C	1.01751800	3.37921600	1.45705300
	C	1.90037600	4.26027000	0.82086200
	C	1.37514700	5.38972300	0.19558500
	H	-0.38800400	6.51228000	-0.30543800
	H	-1.01437400	2.94144900	1.98388900
	H	1.41367800	2.50510200	1.96141000

H	2.04126400	6.09027500	-0.29902300
C	-5.35521100	-1.50394900	0.19558500
C	-4.63969000	-0.48436100	0.82086200
C	-3.43524600	-0.80841200	1.45705300
H	-6.29496500	-1.27735100	-0.29902300
H	-2.87632100	-0.02827000	1.96141000
C	4.87480200	-2.81446800	0.19275100
C	3.31163700	-1.50814000	1.46376000
C	4.55460000	-1.61312100	0.82458700
H	5.83380200	-2.92011900	-0.30543800
C	3.64891500	2.25318000	-3.43211400
C	4.16507900	0.79800800	-3.41801100
H	4.48193200	2.92524100	-3.67115400
H	2.91881500	2.37219600	-4.23772200
H	3.30808800	0.11472400	-3.38374100
H	4.68851700	0.58801300	-4.35499000
C	-2.96190700	-2.11389200	1.46376000
C	-4.87480200	-2.81446800	0.19275100
C	-3.67430400	-3.13783800	0.82458700
H	-2.04018300	-2.34919800	1.98388900
H	-5.44579800	-3.59216200	-0.30543800
C	0.82879900	-3.97170400	-2.09083000
C	-2.12954500	-4.64753000	-2.21977300
C	-1.38509500	-4.22382400	-0.96098700
C	0.05654800	-4.71022700	-1.00630200
H	0.82486500	-2.89608200	-1.88735200
H	1.87361500	-4.28904900	-2.13083400

H	-3.16932600	-4.31070500	-2.20226400
H	-1.38089600	-3.12254000	-0.92362000
N	-1.79871800	-4.72457800	0.35193200
H	0.05423300	-5.78430700	-1.26580800
N	0.42811600	-4.59623800	0.40651900
H	-2.13634000	-5.73983400	-2.30394200
C	3.98006400	-3.88577400	0.19558500
C	2.73931400	-3.77590900	0.82086200
C	2.41772800	-2.57080400	1.45705300
H	4.25370100	-4.81292400	-0.29902300
H	1.46264300	-2.47683300	1.96141000
C	0.12685400	-4.28664300	-3.43211400
C	-1.39144400	-4.00606800	-3.41801100
H	0.29236700	-5.34408800	-3.67115400
H	0.59497400	-3.71386600	-4.23772200
H	-1.55469000	-2.92225100	-3.38374100
H	-1.83502500	-4.35438200	-4.35499000
H	3.05455700	-0.59225100	1.98388900
C	-3.85399600	1.26809100	-2.09083000
C	-2.96010600	4.16800600	-2.21977300
C	-2.96539100	3.31143900	-0.96098700
C	-4.10745100	2.30614100	-1.00630200
H	-2.92051300	0.73368700	-1.88735200
H	-4.65123300	0.52192700	-2.13083400
H	-2.14851700	4.90006900	-2.20226400
H	-2.01375100	2.75716100	-0.92362000
N	-3.19224500	3.92002400	0.35193200

H	-5.03647300	2.84518600	-1.26580800
N	-4.19451700	1.92735900	0.40651900
H	-3.90267300	4.72004200	-2.30394200
C	-3.77576800	2.03346300	-3.43211400
C	-2.77363500	3.20806000	-3.41801100
H	-4.77429900	2.41884700	-3.67115400
H	-3.51379000	1.34167000	-4.23772200
H	-1.75339800	2.80752600	-3.38374100
H	-2.85349300	3.76636900	-4.35499000
C	4.50357700	1.79179100	1.17650200
C	3.39415600	4.00468000	0.86021700
H	3.76137700	4.09871000	1.88535800
H	3.91972200	4.74957300	0.24966400
C	5.54046300	-0.45738300	0.83895200
H	6.41265200	-0.71176000	0.23026200
H	5.89000800	-0.26281000	1.85515500
C	-0.70005200	-4.79610800	1.17650200
C	1.77107600	-4.94176600	0.86021700
H	1.66889900	-5.30680300	1.88535800
H	2.15339000	-5.76936500	0.24966400
C	-3.16633700	-4.56949000	0.83895200
H	-3.82272800	-5.19763900	0.23026200
H	-3.17260400	-4.96949100	1.85515500
C	-3.80352500	3.00431600	1.17650200
C	-2.37412600	5.02687400	0.83895200
H	-2.71740300	5.23230100	1.85515500
H	-2.58992400	5.90939900	0.23026200

	C	-5.16523300	0.93708600	0.86021700
	H	-6.07311100	1.01979200	0.24966400
	H	-5.43027500	1.20809300	1.88535800
	S	4.75561300	1.91357300	2.82984200
	S	-0.72060400	-5.07526800	2.82984200
	S	-4.03500900	3.16169500	2.82984200
Conf. 3[c]	C	6.20612800	-0.23124700	1.17235400
	C	4.22180200	-2.17150600	2.40888600
	C	4.72793100	-2.23673700	0.97451700
	C	5.01331100	-0.83763600	0.44624600
	H	7.09668000	-0.84934100	1.01463400
	H	6.43190700	0.77317500	0.80495600
	H	3.99199000	-3.16528400	2.80240100
	H	5.67272500	-2.80891400	0.97578200
	N	3.89132200	-2.76529300	-0.10601800
	H	4.13539300	-0.20260600	0.64734600
	N	5.07154600	-1.09657900	-0.99598400
	H	3.30104200	-1.58221400	2.45963700
	C	2.12011700	2.04650800	-1.92244900
	C	3.95407100	3.50457600	-1.40413200
	C	2.58469500	3.32561400	-1.59496700
	C	4.83782800	2.43066700	-1.52093000
	C	4.37525400	1.15304500	-1.83585300
	C	2.99944900	0.97809300	-2.04449500
	H	1.05908800	1.89544800	-2.08686200
	H	4.33842500	4.48932100	-1.15581900
	H	5.89974900	2.59385100	-1.36364200

H	2.62315900	-0.00099900	-2.31844400
C	-4.40755600	2.88244700	1.20906800
C	-3.79016800	3.28736600	0.02406300
C	-3.82863100	2.41892400	-1.07377400
H	-4.41280200	3.54795100	2.06654300
H	-3.37215700	2.72337000	-2.00875000
C	1.18744700	-5.15198300	0.96711600
C	1.04385700	-2.83629300	0.36410700
C	1.80372300	-4.00238500	0.46694300
H	1.75652500	-6.07256600	1.05850800
C	5.83594400	-0.16889200	2.67269000
C	5.34486200	-1.51568900	3.24618600
H	5.04868100	0.58310900	2.80336600
H	6.69524700	0.18157500	3.25122300
H	6.19095900	-2.21136900	3.30040700
H	5.00095000	-1.37006300	4.27391800
C	-4.46395700	1.18467400	-0.97642400
C	-5.02323900	1.63743500	1.31154700
C	-5.05904300	0.76760200	0.22111600
H	-4.50803600	0.54933800	-1.85468100
H	-5.47212700	1.33234600	2.24917600
C	-2.78688400	-4.11921000	-1.58898300
C	-5.36793000	-2.61962800	-2.13856300
C	-4.40115200	-2.29666200	-1.00714200
C	-3.71345800	-3.56306000	-0.51582500
H	-2.01833200	-3.38440200	-1.84621500
H	-2.26911000	-5.01879500	-1.24791900

H	-5.87482000	-1.72395000	-2.50920500
H	-3.62451000	-1.61914100	-1.39694400
N	-4.89964000	-1.75543000	0.26208200
H	-4.48882000	-4.32339400	-0.31123800
N	-3.19774600	-3.09914000	0.77721000
H	-6.14143700	-3.30907800	-1.78304200
C	-0.15799600	-5.14060500	1.32773500
C	-0.92043100	-3.97589600	1.21283400
C	-0.29533700	-2.82095200	0.73972300
H	-0.61930500	-6.05133000	1.69800900
H	-0.87110300	-1.90733900	0.64859500
C	-3.66977300	-4.44571700	-2.81521200
C	-4.54061900	-3.25906800	-3.27772400
H	-4.32091700	-5.29166300	-2.56322500
H	-3.03912400	-4.77880200	-3.64385200
H	-3.89237800	-2.48886300	-3.71243400
H	-5.20928600	-3.58524500	-4.07918900
H	1.50410600	-1.93585200	-0.02317500
C	-1.23723100	4.51565600	2.55588000
C	1.46988600	3.52910000	1.60658900
C	0.50178200	4.35539100	0.76963200
C	-0.93676300	4.02717400	1.14436700
H	-1.09668900	5.60069900	2.61632200
H	-2.26712500	4.29997700	2.84855500
H	2.50954200	3.73382300	1.34112100
H	0.68442400	5.42149900	0.99563700
N	0.44470000	4.21927100	-0.69017300

H	-1.06561100	2.93322100	1.12553400
N	-1.64774400	4.59144000	-0.00676400
H	1.29527500	2.46195600	1.43611100
C	-0.26448700	3.77940900	3.50551600
C	1.21499700	3.90140100	3.08586000
H	-0.54421100	2.71995500	3.53272400
H	-0.38816100	4.15655300	4.52455300
H	1.55014300	4.93311000	3.24825700
H	1.83068200	3.27290600	3.73559300
C	4.26736000	-2.17584400	-1.29076000
S	3.82610100	-2.69491900	-2.82407700
C	5.33606200	-0.01920900	-1.95020300
H	6.36143500	0.32783800	-1.79855000
H	5.27985700	-0.47122800	-2.94199500
C	3.24172900	-4.06179800	-0.02303500
H	3.26282000	-4.49397700	-1.02769300
H	3.83416800	-4.71471900	0.62907600
C	-4.07075600	-2.15579900	1.28084600
S	-4.12291100	-1.60943100	2.86565700
C	-2.38165000	-3.95784000	1.62127200
H	-2.48886700	-3.57852300	2.64122700
H	-2.78714100	-4.97864000	1.61040900
C	-5.76155300	-0.57570000	0.34371400
H	-6.51101700	-0.68007800	-0.44516700
H	-6.28218500	-0.62699300	1.30005500
C	-0.83116700	4.55475900	-1.10804900
S	-1.28981000	4.86865200	-2.69261400

	C	1.62488500	4.49433800	-1.50203300
	H	2.14490300	5.37458400	-1.10023100
	H	1.25830400	4.75835000	-2.49728500
	C	-3.10896500	4.64148500	-0.07351400
	H	-3.35374400	5.12950200	-1.01722300
	H	-3.45653300	5.28535600	0.73873000

1b ($CHCl_3$)

Conf. 1[a]	C	-0.43809400	-1.44988100	-7.39169000
	C	0.43809400	1.44988100	-7.39169000
	C	0.56848000	0.50456800	-6.20436000
	C	-0.56848000	-0.50456800	-6.20436000
	H	0.51133100	-1.99271100	-7.33434000
	H	-1.24053000	-2.19205200	-7.40960700
	H	1.24053000	2.19205200	-7.40960700
	H	1.51950200	-0.04615600	-6.30691700
	N	0.48375800	1.01724200	-4.82873400
	H	-1.51950200	0.04615600	-6.30691700
	N	-0.48375800	-1.01724200	-4.82873400
	H	-0.51133100	1.99271100	-7.33434000
	C	-1.77173400	-4.10787000	-1.25997200
	C	0.51362900	-4.12949800	-1.97717700
	C	-0.44245500	-4.44401600	-1.00859600
	C	0.17618300	-3.39119500	-3.10727600
	C	-1.12708800	-2.92926100	-3.28285100
	C	-2.10514000	-3.35143300	-2.37860500
	H	-2.53455500	-4.35219800	-0.52988000
	H	1.55272900	-4.39571500	-1.80989500

H	0.95023300	-3.08934700	-3.80332400
H	-3.13030200	-3.01686800	-2.50496300
C	-1.37669900	0.11008900	4.33699700
C	-0.79824700	-1.16006300	4.27311800
C	0.59012300	-1.25732100	4.35000600
H	-2.45714000	0.20932800	4.29700500
H	1.06172400	-2.23252000	4.31849100
C	-0.17618300	3.39119500	-3.10727600
C	2.10514000	3.35143300	-2.37860500
C	1.12708800	2.92926100	-3.28285100
H	-0.95023300	3.08934700	-3.80332400
C	-0.50190900	-0.58578500	-8.67171800
C	0.50190900	0.58578500	-8.67171800
H	-1.51835300	-0.18710500	-8.77189700
H	-0.32872900	-1.21485800	-9.54906000
H	1.51835300	0.18710500	-8.77189700
H	0.32872900	1.21485800	-9.54906000
C	1.37669900	-0.11008900	4.33699700
C	-0.59012300	1.25732100	4.35000600
C	0.79824700	1.16006300	4.27311800
H	2.45714000	-0.20932800	4.29700500
H	-1.06172400	2.23252000	4.31849100
C	1.50181800	7.03343100	2.41135800
C	1.52044000	5.46539100	5.00379600
C	1.63525700	4.84837200	3.61632900
C	0.85253300	5.66907400	2.60318900
H	2.52542900	6.91101900	2.04144700

H	0.95996800	7.64191200	1.68242400
H	2.06906500	4.88723200	5.75208000
H	2.69899700	4.86054500	3.32189500
N	1.10403000	3.50288700	3.35198000
H	-0.16666400	5.82478200	2.99681900
N	0.77152800	4.71707400	1.48577700
H	0.47063700	5.49390100	5.31459500
C	-0.51362900	4.12949800	-1.97717700
C	0.44245500	4.44401600	-1.00859600
C	1.77173400	4.10787000	-1.25997200
H	-1.55272900	4.39571500	-1.80989500
H	2.53455500	4.35219800	-0.52988000
C	1.49427100	7.74160600	3.78566500
C	2.10820100	6.89259400	4.91796700
H	0.45777400	7.98827900	4.04433800
H	2.02871400	8.69265400	3.71164000
H	3.19049500	6.81407600	4.76114800
H	1.97099900	7.40503400	5.87409000
H	3.13030200	3.01686800	-2.50496300
C	-1.52044000	-5.46539100	5.00379600
C	-1.50181800	-7.03343100	2.41135800
C	-0.85253300	-5.66907400	2.60318900
C	-1.63525700	-4.84837200	3.61632900
H	-0.47063700	-5.49390100	5.31459500
H	-2.06906500	-4.88723200	5.75208000
H	-0.95996800	-7.64191200	1.68242400
H	0.16666400	-5.82478200	2.99681900

N	-0.77152800	-4.71707400	1.48577700
H	-2.69899700	-4.86054500	3.32189500
N	-1.10403000	-3.50288700	3.35198000
H	-2.52542900	-6.91101900	2.04144700
C	-2.10820100	-6.89259400	4.91796700
C	-1.49427100	-7.74160600	3.78566500
H	-3.19049500	-6.81407600	4.76114800
H	-1.97099900	-7.40503400	5.87409000
H	-0.45777400	-7.98827900	4.04433800
H	-2.02871400	-8.69265400	3.71164000
C	0.00000000	0.00000000	-4.01922500
S	0.00000000	0.00000000	-2.34977800
C	-1.51902000	-1.95896800	-4.38307500
H	-2.40340600	-1.39957700	-4.05280300
H	-1.81181000	-2.51951400	-5.27506700
C	1.51902000	1.95896800	-4.38307500
H	2.40340600	1.39957700	-4.05280300
H	1.81181000	2.51951400	-5.27506700
C	0.75945300	3.43497800	2.01055500
S	0.39034600	2.04345000	1.16648600
C	0.00000000	5.08961900	0.29314000
H	-1.06113700	4.86122200	0.45332500
H	0.08481400	6.17668000	0.21166500
C	1.69424100	2.37007400	4.07616200
H	2.61096700	2.04591600	3.56736900
H	1.99391400	2.76515500	5.05060900
C	-0.75945300	-3.43497800	2.01055500

	S	-0.39034600	-2.04345000	1.16648600
	C	0.00000000	-5.08961900	0.29314000
	H	-0.08481400	-6.17668000	0.21166500
	H	1.06113700	-4.86122200	0.45332500
	C	-1.69424100	-2.37007400	4.07616200
	H	-1.99391400	-2.76515500	5.05060900
	H	-2.61096700	-2.04591600	3.56736900
Conf. 2[b]	C	4.22095000	-0.51218600	-2.11274700
	C	3.84774700	-3.52918500	-2.16167600
	C	3.65250300	-2.64386700	-0.93955800
	C	4.60336100	-1.45723700	-0.98274900
	H	3.20185300	-0.14030800	-1.96580500
	H	4.88387700	0.35525100	-2.15604400
	H	3.17843100	-4.39301500	-2.14331400
	H	2.61886600	-2.26454000	-0.95499500
	N	3.92394500	-3.15833200	0.40963500
	H	5.62267600	-1.82958700	-1.18216200
	N	4.56587700	-1.02639900	0.42289800
	H	4.87587400	-3.90521300	-2.19536600
	C	4.22013800	3.76753500	0.18519500
	C	2.47590400	2.67172100	1.43784700
	C	2.96353100	3.82771100	0.80175400
	C	3.22272400	1.48994400	1.44490600
	C	4.48200000	1.42905400	0.82317000
	C	4.97343800	2.58276400	0.19944800
	H	4.61652000	4.64696300	-0.31332800
	H	1.51402200	2.70010700	1.93827900

H	2.83246900	0.60997700	1.94460700
H	5.94448500	2.56057700	-0.28597000
C	-4.71409400	2.99771600	0.23390000
C	-3.46096000	3.14466200	0.82616300
C	-2.88459500	2.03244500	1.45182900
H	-5.18170900	3.84661600	-0.25499100
H	-1.91567500	2.13218400	1.92795700
C	1.14814700	-5.56320400	0.36121200
C	1.03832900	-3.35042700	1.28632100
C	1.80961900	-4.43034100	0.83954000
H	1.72450300	-6.40998400	0.00184700
C	4.32873700	-1.32490700	-3.42493200
C	3.54131000	-2.65191000	-3.39824300
H	5.38697800	-1.54006100	-3.61395800
H	3.98369100	-0.71410800	-4.26355400
H	2.46733300	-2.43182900	-3.40868300
H	3.75005500	-3.21713900	-4.31046800
C	-3.54259600	0.80884800	1.47668000
C	-5.37205500	1.76706400	0.25196800
C	-4.79653400	0.65683600	0.87045500
H	-3.08557700	-0.03498200	1.98129100
H	-6.34309600	1.67342400	-0.22395500
C	-2.52166800	-3.25586000	-2.14360300
C	-4.98491500	-1.47455100	-2.18428900
C	-4.13732600	-1.76649600	-0.95421500
C	-3.55677500	-3.17023100	-1.03145700
H	-1.70991500	-2.54489800	-1.95916600

H	-2.07752400	-4.25238100	-2.20633800
H	-5.41829800	-0.47209800	-2.14564900
H	-3.30670400	-1.04375800	-0.93268800
N	-4.74226800	-1.79653700	0.38447600
H	-4.37103900	-3.87539600	-1.27031700
N	-3.18862500	-3.39196700	0.37432300
H	-5.80988500	-2.19153700	-2.25311700
C	-0.24584500	-5.61997000	0.33385400
C	-1.01311800	-4.54035300	0.77489600
C	-0.35111800	-3.40355500	1.25038300
H	-0.73689500	-6.51103200	-0.04476700
H	-0.93084000	-2.55742500	1.60020100
C	-3.26094400	-2.92149900	-3.46076200
C	-4.04667500	-1.59368700	-3.40856100
H	-3.95462000	-3.73970400	-3.68704100
H	-2.54420400	-2.88739800	-4.28567800
H	-3.33700700	-0.75842700	-3.38197400
H	-4.62657500	-1.47765600	-4.32807800
H	1.52778400	-2.46446100	1.67457200
C	-1.64263700	3.68300300	-2.10170600
C	1.13053000	4.91991000	-2.25008400
C	0.48189000	4.37781500	-0.98462200
C	-1.02562600	4.57398000	-1.03319100
H	-1.43043300	2.63104900	-1.88577200
H	-2.72839000	3.79648700	-2.14460800
H	2.21589700	4.79369200	-2.23103700
H	0.69431900	3.29882000	-0.92656100

N	0.78891900	4.96798700	0.32536000
H	-1.23522600	5.62279000	-1.30449600
N	-1.36766600	4.41714900	0.38853600
H	0.92170200	5.99018100	-2.35252100
C	-1.01299400	4.10831800	-3.44922500
C	0.53059000	4.12520500	-3.43374400
H	-1.37966200	5.10988700	-3.70292400
H	-1.36117400	3.44211600	-4.24320500
H	0.89947400	3.09414000	-3.38039100
H	0.89943800	4.53711900	-4.37692600
C	4.33723600	-2.12753500	1.21004900
C	5.30195500	0.15285000	0.86894800
H	5.62798500	-0.04300900	1.89289100
H	6.19946200	0.26157000	0.25041000
C	3.32805200	-4.39643900	0.89950000
H	3.73536000	-5.22757000	0.31833000
H	3.67064700	-4.51673700	1.92922600
C	-4.05217900	-2.68098200	1.16951600
C	-2.52842300	-4.62375300	0.78968400
H	-2.87603200	-4.84936300	1.80077700
H	-2.86316400	-5.43775000	0.13710400
C	-5.52554400	-0.67612200	0.89981200
H	-6.44614000	-0.60306500	0.31575900
H	-5.80204800	-0.94013400	1.92206000
C	-0.30182600	4.84361400	1.14169100
C	2.16184800	5.11918000	0.79950200
H	2.09488700	5.53129600	1.80802600

	H	2.66278000	5.86023400	0.17168900
	C	-2.75477700	4.48679400	0.83723500
	H	-3.29580300	5.20070300	0.20596300
	H	-2.73917000	4.88982800	1.85231000
	S	4.53859000	-2.19689100	2.88738300
	S	-4.23352200	-2.87439600	2.83913600
	S	-0.33692700	5.16442600	2.80131700
Conf. 3[c]	C	6.18627900	-0.49846700	1.15398800
	C	4.14987700	-2.38251500	2.39896400
	C	4.63764900	-2.44908300	0.95912600
	C	4.96219500	-1.05560200	0.44201300
	H	7.05169300	-1.14646000	0.97903100
	H	6.44197500	0.50098800	0.79340800
	H	3.89504800	-3.37272700	2.78535400
	H	5.55915600	-3.05512900	0.94101700
	N	3.77220200	-2.94253700	-0.12202900
	H	4.10965000	-0.39338500	0.65933500
	N	4.98527500	-1.30038800	-1.00773800
	H	3.25147100	-1.76144000	2.46761500
	C	2.20503900	2.00816700	-1.86405000
	C	4.13215700	3.37027800	-1.42509800
	C	2.74953300	3.26472300	-1.57558800
	C	4.95145200	2.24699200	-1.54697100
	C	4.40923500	0.99179600	-1.82553600
	C	3.02068100	0.88977100	-1.98854800
	H	1.13288000	1.90921300	-1.99060500
	H	4.57710600	4.33474900	-1.20123800

H	6.02397600	2.35267100	-1.41817100
H	2.57889400	-0.07173900	-2.22437100
C	-4.37613200	3.15565400	1.12567900
C	-3.61469600	3.45288800	-0.00583700
C	-3.54834400	2.49934900	-1.02936600
H	-4.45655200	3.88375900	1.92635900
H	-2.97791100	2.71649300	-1.92539400
C	0.98173400	-5.22587300	0.95375400
C	0.91568500	-2.91106800	0.32919900
C	1.63627900	-4.10084300	0.44436800
H	1.52027400	-6.16338200	1.05423700
C	5.83681000	-0.43887300	2.65970400
C	5.30652500	-1.77319500	3.22669600
H	5.07948500	0.33960600	2.80872300
H	6.71682300	-0.12677100	3.22841800
H	6.12757500	-2.49886700	3.26358700
H	4.97894900	-1.62473400	4.25904400
C	-4.22288500	1.28765200	-0.91620000
C	-5.03624100	1.93399400	1.24516100
C	-4.96807600	0.97927900	0.22960600
H	-4.17559600	0.58706400	-1.74178400
H	-5.60658700	1.71883500	2.14154200
C	-3.10471800	-4.15120600	-1.55140200
C	-5.58542900	-2.47210700	-2.05773400
C	-4.54797400	-2.18867700	-0.98101900
C	-3.94216600	-3.48685400	-0.46805200
H	-2.29283100	-3.48885700	-1.86649300

H	-2.64958300	-5.07833400	-1.19518000
H	-6.03467200	-1.55249000	-2.44227100
H	-3.73753000	-1.59248700	-1.42751700
N	-4.93829400	-1.55496100	0.28919800
H	-4.76120200	-4.17681400	-0.20108200
N	-3.34028600	-3.02043400	0.79073800
H	-6.39322000	-3.08799100	-1.64847700
C	-0.36168700	-5.16560900	1.31754600
C	-1.08422800	-3.97583600	1.19399700
C	-0.42330700	-2.84792300	0.70446900
H	-0.85212400	-6.05627900	1.69810900
H	-0.96590800	-1.91542800	0.60192500
C	-4.06031800	-4.44734800	-2.73060600
C	-4.85406700	-3.21123100	-3.20249500
H	-4.76361000	-5.23037800	-2.42320700
H	-3.49020700	-4.85507800	-3.56943400
H	-4.16659800	-2.50790200	-3.68665200
H	-5.57790500	-3.51050300	-3.96509100
H	1.40465100	-2.02689800	-0.06060000
C	-1.07770300	4.60695600	2.52777200
C	1.62866300	3.56403200	1.62804600
C	0.69342600	4.41154000	0.77660000
C	-0.75742000	4.11325900	1.12348400
H	-0.91586000	5.68835300	2.59336200
H	-2.11726200	4.40999400	2.79855600
H	2.67689100	3.75025500	1.38361200
H	0.89706400	5.47231300	1.00243700

N	0.65720300	4.28243400	-0.68908000
H	-0.91043500	3.02336200	1.09799600
N	-1.43628300	4.68992800	-0.04578400
H	1.43465400	2.50062300	1.45661100
C	-0.13667400	3.84962400	3.49319700
C	1.35259800	3.94341900	3.10230300
H	-0.43704900	2.79581800	3.51303400
H	-0.27219900	4.22924100	4.50948200
H	1.70431900	4.96841300	3.26972900
H	1.94314100	3.30212600	3.76214000
C	4.14981800	-2.34837700	-1.29558800
S	3.67228100	-2.84139600	-2.84162700
C	5.30418400	-0.23059400	-1.95339600
H	6.34646600	0.05869600	-1.80128000
H	5.22257800	-0.66559700	-2.95055900
C	3.07482300	-4.21508100	-0.03505200
H	3.08882000	-4.66262500	-1.03218200
H	3.63912100	-4.87849900	0.62891300
C	-4.10815900	-1.99371000	1.28199900
S	-4.05466200	-1.38519900	2.85775700
C	-2.53905200	-3.90595800	1.62260700
H	-2.61229800	-3.52770700	2.64521800
H	-2.98141900	-4.90954500	1.61412700
C	-5.73305400	-0.32909100	0.36924900
H	-6.49292300	-0.40164500	-0.41259200
H	-6.25253500	-0.33996800	1.32744600
C	-0.59739200	4.64371100	-1.12219200

	S	-1.02291900	4.98351400	-2.72496700
	C	1.86494300	4.49244100	-1.47998800
	H	2.43220100	5.32968200	-1.05484000
	H	1.53706700	4.79407900	-2.47734500
	C	-2.89376100	4.78462700	-0.12818300
	H	-3.11849100	5.25759500	-1.08444800
	H	-3.22993000	5.45655500	0.66493900

3 (*in vacuo*)

Conf. 1	C	4.50148700	2.01267900	-0.80710100
	C	3.54696400	2.26901600	1.29333800
	H	2.48364800	2.03824600	1.11842900
	C	3.66959000	2.92541400	2.66204900
	H	3.12940900	3.87465200	2.70348400
	H	4.72160800	3.13711800	2.88247200
	C	3.08063100	1.93519000	3.69048100
	H	3.22163900	2.32405200	4.70275000
	H	1.99720300	1.87270200	3.53193000
	C	3.68882800	0.52169800	3.59142900
	H	4.72635300	0.55482300	3.94500800
	H	3.15797300	-0.15436000	4.26772700
	C	3.67870200	-0.06634600	2.16054600
	H	4.21547700	-1.01578000	2.15383100
	H	2.65646600	-0.27002700	1.82494900
	C	4.33217500	0.96409400	1.24639600
	H	5.35089600	1.15546500	1.62783100
	C	3.66444900	4.29998200	-0.24470400
	H	4.16430100	4.53954300	-1.18601200

H	4.03226800	4.98889000	0.51467900
C	2.15659200	4.44522100	-0.40005100
C	1.47321000	3.54808900	-1.23107400
C	0.09437300	3.59516700	-1.38186400
C	-0.68599200	4.53836000	-0.69758400
C	1.38433000	5.41969500	0.23526000
C	0.00000000	5.46533100	0.08932300
C	-2.19977700	4.61217000	-0.78726700
H	-2.54124900	5.53084800	-0.29274000
H	-2.52148600	4.66455000	-1.82965400
C	-3.99377500	2.89206300	-0.80710100
C	-3.73850700	1.93725300	1.29333800
H	-3.00699700	1.13177900	1.11842900
C	-4.36827800	1.71525200	2.66204900
H	-5.07762800	2.52047400	2.88247200
H	-4.92025200	0.77282200	2.70348400
C	-3.21623900	1.70031000	3.69048100
H	-2.62040900	0.79327700	3.53193000
H	-3.62350700	1.62799600	4.70275000
C	-2.29621800	2.93377000	3.59142900
H	-2.84366700	3.81573000	3.94500800
H	-1.44530700	2.81206500	4.26772700
C	-1.78189300	3.21902200	2.16054600
H	-1.09438200	2.43558000	1.82494900
H	-1.22804700	4.15860100	2.15383100
C	-3.00101700	3.26972700	1.24639600
H	-3.67611000	4.05627900	1.62783100

C	-5.55611800	1.02351500	-0.24470400
H	-6.01351100	1.33661900	-1.18601200
H	-6.33664000	0.99760100	0.51467900
C	-4.92797000	-0.35494700	-0.40005100
C	-3.80934000	-0.49820700	-1.23107400
C	-3.16069200	-1.71585400	-1.38186400
C	-3.58733900	-2.86326600	-0.69758400
C	-4.73311500	-2.73266500	0.08932300
C	-5.38575900	-1.51098300	0.23526000
C	-2.89436800	-4.21114800	-0.78726700
H	-3.51923000	-4.96621000	-0.29274000
H	-2.77887500	-4.51594600	-1.82965400
C	-0.50771200	-4.90474200	-0.80710100
C	0.19154300	-4.20626900	1.29333800
H	0.52334900	-3.17002600	1.11842900
C	0.69868700	-4.64066500	2.66204900
H	1.79084300	-4.64747400	2.70348400
H	0.35602000	-5.65759200	2.88247200
C	0.13560800	-3.63550000	3.69048100
H	0.40186800	-3.95204700	4.70275000
H	0.62320600	-2.66597900	3.53193000
C	-1.39261000	-3.45546800	3.59142900
H	-1.71266600	-2.65770500	4.26772700
H	-1.88268600	-4.37055300	3.94500800
C	-1.89680900	-3.15267600	2.16054600
H	-2.98743000	-3.14282000	2.15383100
H	-1.56208300	-2.16555300	1.82494900

C	-1.33115800	-4.23382100	1.24639600
H	-1.67478500	-5.21174400	1.62783100
C	1.89166900	-5.32349600	-0.24470400
H	2.30437200	-5.98649100	0.51467900
H	1.84920900	-5.87616200	-1.18601200
C	2.77137800	-4.09027300	-0.40005100
C	2.33613000	-3.04988200	-1.23107400
C	3.06631900	-1.87931200	-1.38186400
C	4.27333100	-1.67509400	-0.69758400
C	4.73311500	-2.73266500	0.08932300
C	4.00142900	-3.90871300	0.23526000
C	5.09414500	-0.40102200	-0.78726700
H	6.06047900	-0.56463700	-0.29274000
H	5.30036100	-0.14860400	-1.82965400
N	4.43413500	0.76539200	-0.20862300
N	4.07485200	2.94698100	0.11012400
N	-2.87991700	3.45737800	-0.20862300
N	-4.58958700	2.05543500	0.11012400
N	-1.55421900	-4.22277000	-0.20862300
N	0.51473400	-5.00241600	0.11012400
S	5.03309500	2.34540100	-2.35777800
S	-4.54772400	3.18608700	-2.35777800
S	-0.48537100	-5.53148800	-2.35777800
H	2.04078100	2.80313800	-1.77408900
H	-0.56102000	6.23116900	0.61111900
Br	-0.69618200	2.34124600	-2.58950000
Br	2.18245600	6.76439500	1.36370500

	H	-3.44797900	0.36579900	-1.77408900
	H	-5.11584000	-3.60144200	0.61111900
	Br	-1.67948800	-1.77353400	-2.58950000
	Br	-6.94936600	-1.49213500	1.36370500
	H	1.40719800	-3.16893700	-1.77408900
	H	5.67686000	-2.62972700	0.61111900
	Br	2.37567000	-0.56771200	-2.58950000
	Br	4.76691000	-5.27226000	1.36370500
Conf. 2	C	-6.35990000	-1.20993800	1.42999500
	C	-4.94086600	1.15995400	2.69117600
	C	-5.35157500	1.06252900	1.22876600
	C	-5.27911700	-0.38136300	0.74728100
	H	-7.34855500	-0.80338400	1.19020400
	H	-6.34272200	-2.24992300	1.09883400
	H	-4.96443600	2.19280300	3.04955000
	H	-6.39889000	1.40126400	1.14933100
	N	-4.58639500	1.73743800	0.17464300
	H	-4.29870300	-0.79885400	1.03079100
	N	-5.29238600	-0.18452200	-0.70788600
	H	-3.91892000	0.79166800	2.82626900
	C	-1.64059700	-2.38498600	-1.86077100
	C	-2.87632000	-4.26853500	-1.08844200
	C	-1.63648000	-3.71160300	-1.40793100
	C	-4.05477200	-3.53201100	-1.17503900
	C	-4.05463400	-2.18978900	-1.56290800
	C	-2.81266400	-1.64379900	-1.92048800
	C	5.09678200	-1.44174300	1.28549400

C	4.50390600	-2.26130000	0.32119500
C	4.28044200	-1.68250400	-0.93509000
C	-2.31435500	4.71285200	0.81177100
C	-1.91131200	2.37222600	0.81721200
C	-2.81229700	3.42001300	0.63474800
C	-6.08854700	-1.13939900	2.95100800
C	-5.94549200	0.29911800	3.49205200
H	-5.16783000	-1.69519800	3.16491600
H	-6.88853300	-1.65354100	3.49055400
H	-6.92474100	0.79155100	3.46548200
H	-5.64614000	0.26637300	4.54305500
C	4.58496000	-0.35202800	-1.18494000
C	5.36006900	-0.09755700	1.04538000
C	5.08461700	0.49764500	-0.18980200
C	1.25294900	4.52889100	-1.68475300
C	4.06016800	3.90999700	-2.63974600
C	3.39741400	3.27740600	-1.42197400
C	2.44781200	4.27795400	-0.77286500
H	0.71141100	3.59587200	-1.86769100
H	0.54504600	5.23273700	-1.24356700
H	4.76755800	3.22814000	-3.11540900
H	2.81052000	2.41046400	-1.75457700
N	4.21371300	2.88095000	-0.26456000
H	2.98175100	5.23697200	-0.64681900
N	2.30134900	3.68020100	0.56128100
H	4.61334200	4.80673800	-2.33916800
C	-0.97744300	4.94633000	1.11217900

C	-0.06671100	3.89607900	1.26898600
C	-0.58158300	2.60118000	1.14526300
C	1.80833400	5.11499100	-3.00302800
C	2.93070200	4.26604200	-3.63302100
H	2.19252200	6.12222900	-2.80059300
H	0.99252900	5.23486800	-3.72087400
H	2.50261100	3.33342500	-4.01847200
H	3.34922800	4.79346500	-4.49468600
C	1.98767700	-3.59433000	2.97958900
C	-0.73693800	-3.24919500	1.69783700
C	0.43121300	-3.99647500	1.06357500
C	1.75073000	-3.36369600	1.49257500
H	2.01849600	-4.66958800	3.18915600
H	2.93595700	-3.16635700	3.30856400
H	-1.69727700	-3.67519800	1.40271900
H	0.40916100	-5.03686800	1.43457300
N	0.62044800	-4.05061100	-0.39404400
H	1.69111800	-2.27800300	1.31585400
N	2.66285500	-3.94448900	0.50215700
H	-0.73003500	-2.20203600	1.37964200
C	0.82099200	-2.91959700	3.73413300
C	-0.56747100	-3.36088000	3.23098900
H	0.91642600	-1.83329900	3.62336100
H	0.90610200	-3.12733800	4.80434600
H	-0.74077500	-4.40330500	3.52532200
H	-1.34154800	-2.76901100	3.72777400
C	-4.75315700	1.04737200	-1.00622800

S	-4.37989800	1.63000800	-2.53203300
C	-5.29409900	-1.30685900	-1.64535000
H	-6.19041100	-1.89849800	-1.46676900
H	-5.36908700	-0.87258900	-2.64355900
C	-4.25437100	3.14869400	0.23067200
H	-4.43343300	3.58084700	-0.75762900
H	-4.93424400	3.64532800	0.93019600
C	3.50515800	3.08581300	0.89263500
S	4.02888100	2.73277600	2.44485900
C	1.39013200	4.20750800	1.56410800
H	1.69545500	3.76678300	2.51640800
H	1.51165200	5.29465100	1.65729600
C	5.37164100	1.98791700	-0.35739600
H	5.83767000	2.17274800	-1.32576900
H	6.07918400	2.28951600	0.41578100
C	1.96661300	-4.24057800	-0.64456600
S	2.63147600	-4.77777800	-2.08817900
C	-0.39498300	-4.57490200	-1.29701800
H	-0.69336600	-5.58446500	-0.98423400
H	0.08976700	-4.67189700	-2.27161700
C	4.10630900	-3.71611800	0.53575500
H	4.53368600	-4.33662500	-0.25329900
H	4.48583800	-4.07362800	1.49166300
H	3.87688100	-2.30251600	-1.72538900
H	5.77065900	0.51546900	1.83462400
Br	4.36109100	0.23437700	-3.00737600
Br	5.63058700	-2.12186500	3.00962800

	H	-2.78058100	-0.62067500	-2.27310900
	H	-2.91989800	-5.30071100	-0.76281900
	Br	-5.68608100	-4.47563200	-0.76062000
	Br	-0.03603300	-1.54081900	-2.47440400
	H	-2.26439200	1.35919400	0.69135100
	H	-0.62799600	5.96662500	1.21598900
	Br	0.51276200	1.05654400	1.44559600
	Br	-3.47158800	6.23576500	0.61171400
Conf. 3	C	-5.13094500	2.00146800	2.20159100
	C	-2.33076000	3.15387100	2.39397200
	C	-3.29463800	3.40893300	1.24128400
	C	-4.18350700	2.19069000	1.02416900
	H	-5.76597400	2.88697400	2.31525500
	H	-5.78972100	1.14240400	2.05546200
	H	-1.67682500	4.00810700	2.57554500
	H	-3.93608200	4.26340200	1.52046200
	N	-2.82356600	3.64243800	-0.13260900
	H	-3.53813700	1.30027000	0.95623200
	N	-4.72000600	2.47671700	-0.30889300
	H	-1.68793000	2.29756800	2.16689000
	C	-3.29675400	-1.30502600	-1.81895500
	C	-5.09279000	-2.23260700	-0.55999900
	C	-3.85159400	-2.41707100	-1.17081400
	C	-5.70821100	-0.98387600	-0.51970800
	C	-5.10881300	0.14687000	-1.07869300
	C	-3.90161200	-0.05755200	-1.76267500
	C	3.39386100	-4.03681000	1.24713400

C	2.39393400	-4.22958100	0.28961600
C	2.44756100	-3.40852500	-0.84369300
C	0.25406300	5.07169000	0.88691100
C	0.27992500	3.21264500	-0.59746900
C	-0.42322500	4.29686300	-0.06115000
C	-4.25453700	1.78851000	3.45606300
C	-3.19034100	2.88651400	3.65162800
H	-3.75538900	0.81591500	3.36999700
H	-4.88890100	1.73082300	4.34494200
H	-3.68973300	3.82066300	3.93607500
H	-2.53836600	2.62085400	4.48824500
C	3.43008100	-2.43751800	-0.98864600
C	4.34360400	-3.03137500	1.11968500
C	4.38228700	-2.19110000	0.00529300
C	4.94463700	3.58103800	-1.65688900
C	6.60497200	1.07672400	-2.09693100
C	5.33324100	1.14531400	-1.26429500
C	5.20557900	2.51184200	-0.60572400
H	4.02292600	3.36089800	-2.20442200
H	4.82790800	4.57056000	-1.20638100
H	6.72001200	0.10372800	-2.58176600
H	4.47882200	1.00866300	-1.93948200
N	5.14135700	0.26026200	-0.10078900
H	6.15845400	2.75667500	-0.10359900
N	4.21730600	2.21147500	0.43667300
H	7.48437700	1.23813500	-1.46365200
C	1.54491100	4.75686400	1.29857600

C	2.22797900	3.64905400	0.79165400
C	1.56040900	2.89093800	-0.16866800
C	6.16319400	3.57553700	-2.60943300
C	6.49257600	2.17855700	-3.17798100
H	7.03487500	3.95613600	-2.06371600
H	5.98866000	4.27171700	-3.43410100
H	5.70912900	1.88698300	-3.88690700
H	7.42278700	2.22748400	-3.75048000
C	-0.26691700	-4.06966400	2.93994200
C	-2.54536100	-2.40290300	1.84298400
C	-1.96653100	-3.63377300	1.15185400
C	-0.47926400	-3.74479800	1.46724600
H	-0.75928800	-5.01628400	3.18995800
H	0.79196700	-4.17710400	3.18093700
H	-3.60918800	-2.28248200	1.63819500
H	-2.47688100	-4.52451700	1.55984100
N	-1.94602900	-3.76981100	-0.31526900
H	-0.00434700	-2.77288700	1.25963000
N	-0.05515100	-4.69352500	0.43580700
H	-2.03877000	-1.50159900	1.48281000
C	-0.87225000	-2.90316300	3.75028900
C	-2.33013200	-2.58809400	3.36299800
H	-0.25385900	-2.01234400	3.59022000
H	-0.81668200	-3.12838500	4.81889900
H	-2.97733900	-3.40370900	3.70817600
H	-2.66381100	-1.68996200	3.89078100
C	-3.82714600	3.26587200	-1.00294100

S	-3.96553800	3.70903300	-2.61236200
C	-5.65450100	1.56480000	-0.96539700
H	-6.59245800	1.56552500	-0.41208300
H	-5.84642000	1.97910000	-1.95691500
C	-1.84063300	4.66678000	-0.46329100
H	-1.90775600	4.81435500	-1.54368100
H	-2.11234100	5.61604100	0.01576900
C	4.41440100	0.90989900	0.86045200
S	3.88191000	0.26327700	2.30966500
C	3.61749500	3.24034800	1.26180000
H	3.55359300	2.85843600	2.28535800
H	4.27476800	4.11519500	1.28233900
C	5.50106000	-1.15456400	-0.05873900
H	6.10969800	-1.34187000	-0.94542800
H	6.13936600	-1.31700700	0.81293300
C	-0.88410900	-4.59410400	-0.65354900
S	-0.65938800	-5.36362200	-2.12616000
C	-3.17826600	-3.77324100	-1.10209600
H	-3.88522400	-4.50190100	-0.68327000
H	-2.90212700	-4.12202000	-2.09903600
C	1.28514200	-5.26664500	0.39706500
H	1.30917400	-5.93007100	-0.46955700
H	1.42875500	-5.87251100	1.29090100
H	1.71614000	-3.55785700	-1.62839800
H	5.06418100	-2.87907000	1.91189800
Br	3.45486800	-1.48858800	-2.66164000
Br	3.54357900	-5.16211200	2.80640000

	H	2.05994500	2.02960100	-0.58713400
	H	-0.24229500	5.93453300	1.31488400
	Br	-0.45305000	2.11241400	-1.97903000
	Br	2.36739400	5.88745400	2.62101700
	H	-3.43646400	0.78632600	-2.25586300
	H	-5.57608600	-3.07735700	-0.08406400
	Br	-7.42338900	-0.89341700	0.35809000
	Br	-1.68896500	-1.47711800	-2.84176000
Conf. 4	C	4.80095200	1.19625800	-1.00216200
	C	4.07343900	1.98697200	1.05423200
	H	3.00122500	1.73144700	1.04021100
	C	4.31069200	2.95052800	2.20886900
	H	3.75730600	3.88368900	2.07599000
	H	5.37393400	3.20592000	2.27381900
	C	3.83756400	2.23316200	3.49502600
	H	4.06629500	2.85170500	4.36712300
	H	2.74526900	2.14269200	3.45898500
	C	4.45302800	0.83035100	3.68226700
	H	5.51873500	0.93531000	3.91764000
	H	3.99518200	0.34353800	4.54775900
	C	4.31397700	-0.07922900	2.43918800
	H	4.85977300	-1.01212900	2.59848100
	H	3.26524400	-0.33868300	2.25886400
	C	4.86893100	0.70484700	1.25840100
	H	5.91316700	0.97562000	1.49279600
	C	4.09545200	3.59594900	-0.94018100
	H	4.51504800	3.57574000	-1.94894800

H	4.57012200	4.41373600	-0.39899800
C	2.59244100	3.82728800	-1.01346500
C	1.76498400	2.81144500	-1.50798500
C	0.38859400	2.96871600	-1.60977900
C	-0.24127800	4.15872000	-1.21783000
C	1.96518500	5.01998100	-0.64611800
C	0.58632700	5.18129100	-0.74870900
C	-1.73276100	4.41536600	-1.33067800
H	-1.93020400	5.46508800	-1.07626400
H	-2.06463000	4.26581600	-2.36078100
C	-3.79394900	3.09354900	-0.95517200
C	-3.58939800	2.53216400	1.28660300
H	-3.03052900	1.58244100	1.26066000
C	-4.15742700	2.71368700	2.68852300
H	-4.69195000	3.66784300	2.75364000
H	-4.86632900	1.92311100	2.94274200
C	-2.96412000	2.68431900	3.66918300
H	-2.55593700	1.66653600	3.68885700
H	-3.31412900	2.89459600	4.68357300
C	-1.84149600	3.67266500	3.29473400
H	-2.19441600	4.69614200	3.46932500
H	-0.98624900	3.52666800	3.96070800
C	-1.37809700	3.55941700	1.82388700
H	-0.87457000	2.60403600	1.64310700
H	-0.66054700	4.35089200	1.60308800
C	-2.62449600	3.66427000	0.95282600
H	-3.11973200	4.62363200	1.18639500

C	-5.62261500	1.67726500	-0.00771200
H	-6.12343700	1.94598500	-0.93916600
H	-6.31030200	1.86897400	0.81440700
C	-5.21962300	0.20899200	-0.03461600
C	-4.41833200	-0.24161000	-1.09327100
C	-3.93445400	-1.54034100	-1.14194700
C	-4.19341100	-2.45815000	-0.11397300
C	-5.06251000	-2.03670300	0.89387400
C	-5.56908300	-0.73961000	0.92881500
C	-3.57795300	-3.84368800	-0.04390700
H	-4.08364800	-4.42233800	0.74070100
H	-3.71179700	-4.38162800	-0.98419500
C	-1.27738600	-4.71722900	-0.39891600
C	-0.10357500	-3.62016000	1.28545700
H	0.23618600	-2.67428300	0.83557400
C	0.63718400	-3.81647100	2.60529700
H	1.71243200	-3.92361700	2.45504000
H	0.27952700	-4.72526500	3.10158400
C	0.35673500	-2.57001800	3.47273000
H	0.80502900	-2.69841600	4.46208400
H	0.85995200	-1.71013700	3.01405900
C	-1.14478600	-2.25733600	3.62203200
H	-1.27209900	-1.30435000	4.14392000
H	-1.60525000	-3.01966100	4.26195500
C	-1.91197400	-2.21895000	2.27975500
H	-2.97775400	-2.10634800	2.47795500
H	-1.60260100	-1.36271200	1.67141800

C	-1.60289900	-3.51950100	1.54571000
H	-1.89647800	-4.35390800	2.20822800
C	1.17415500	-5.20331700	-0.29587700
H	1.51833200	-6.03551200	0.32556500
H	0.91972800	-5.61266000	-1.27599800
C	2.29905700	-4.18542300	-0.39908600
C	2.28206100	-3.07182200	-1.25219500
C	3.30201800	-2.13246200	-1.23904000
H	3.25156300	-1.27617600	-1.89985400
C	4.41050800	-2.25983200	-0.39404200
C	4.46796500	-3.40795400	0.39614200
C	3.43417500	-4.34212300	0.40053900
H	3.50486500	-5.20603400	1.05001400
C	5.44258100	-1.14542500	-0.40864600
H	6.24334000	-1.34890700	0.30453900
H	5.88610200	-1.07187700	-1.40454900
N	4.84959000	0.15324200	-0.10160400
N	4.47714000	2.34009400	-0.31027300
N	-2.56264600	3.54155500	-0.51062100
N	-4.47865800	2.57949800	0.12363600
N	-2.13910300	-3.81194900	0.20834900
N	-0.06969300	-4.66315800	0.25909500
S	5.08517300	1.08458300	-2.65037300
S	-4.37079000	3.19148500	-2.52431700
S	-1.65090000	-5.73754300	-1.66788300
Br	0.85207600	-2.79792000	-2.48674700
Br	5.97328800	-3.76612000	1.54653100

	H	2.21853300	1.88761800	-1.84304700
	H	0.14124900	6.12332100	-0.45222500
	Br	-0.60517900	1.50834200	-2.34445300
	Br	2.96663100	6.52571400	0.02245600
	H	-4.17905600	0.45017600	-1.89087000
	H	-5.33210800	-2.73151000	1.68005500
	Br	-2.94458600	-2.04909900	-2.69641100
	Br	-6.77665400	-0.32253400	2.37497800

3 (DCM)

Conf. 1	C	4.90057200	0.55029900	-0.79923700
	C	4.05978600	1.06283300	1.29920500
	H	2.97684800	1.15354500	1.12223400
	C	4.37052000	1.65175900	2.66792900
	H	4.12178400	2.71483900	2.71476000
	H	5.43922100	1.55253900	2.88538100
	C	3.52429700	0.86702400	3.69502000
	H	3.77747700	1.19474000	4.70663500
	H	2.46848800	1.11940900	3.54146300
	C	3.70136300	-0.66054500	3.58839300
	H	4.70665300	-0.92925400	3.93325700
	H	3.00043400	-1.15822000	4.26385000
	C	3.51222800	-1.21391000	2.15581500
	H	3.75493600	-2.27673200	2.14453000
	H	2.47267800	-1.10817000	1.82940000
	C	4.43418100	-0.41181700	1.24617200
	H	5.46483800	-0.52849900	1.61951700
	C	4.78617000	2.98352800	-0.22028400

H	5.35407900	3.08457400	-1.14697900
H	5.32993500	3.51176600	0.56140900
C	3.39576900	3.58300800	-0.38869000
C	2.46829300	2.92600400	-1.20615700
C	1.17294400	3.39692900	-1.36365400
C	0.71993100	4.54384800	-0.69907500
C	2.96349800	4.76210700	0.22265200
C	1.65980500	5.22881400	0.07422000
C	-0.69890800	5.07781000	-0.79208000
H	-0.74444100	6.05525300	-0.29841900
H	-0.98320700	5.22277200	-1.83548600
C	-2.92642600	3.96719400	-0.79154700
C	-2.94936200	2.97496300	1.30351700
H	-2.48993000	1.99081900	1.12330900
C	-3.61271200	2.94568900	2.67327800
H	-4.05794400	3.92106200	2.89626400
H	-4.41085400	2.20052800	2.71802300
C	-2.50831700	2.59748600	3.69629700
H	-2.20334300	1.55640600	3.53806100
H	-2.91578100	2.65069800	4.70923400
C	-1.27055300	3.51022800	3.59007200
H	-1.53551400	4.51480300	3.93972600
H	-0.48911500	3.14591800	4.26227800
C	-0.69970000	3.62708100	2.15688000
H	-0.27649300	2.67361000	1.82467400
H	0.10284300	4.36497800	2.14645300
C	-1.85654500	4.03365200	1.25284900

H	-2.26632700	4.98386600	1.63285500
C	-4.97739500	2.65239200	-0.21495100
H	-5.35054300	3.09779600	-1.13903700
H	-5.70454000	2.85717400	0.56944700
C	-4.80391200	1.14897500	-0.38871800
C	-3.77004800	0.67456800	-1.20494400
C	-3.53142600	-0.68287800	-1.36418800
C	-4.29975000	-1.64876800	-0.70144300
C	-5.36569500	-1.17732800	0.06809600
C	-5.61260100	0.18496200	0.21751800
C	-4.05241400	-3.14458200	-0.79306100
H	-4.87760000	-3.67213800	-0.30127300
H	-4.03372800	-3.46387100	-1.83621500
C	-1.97434600	-4.51479900	-0.79009400
C	-1.10621300	-4.03619600	1.30525000
H	-0.48804400	-3.14332100	1.12378500
C	-0.74645700	-4.59374500	2.67516300
H	0.29958300	-4.90677500	2.72042900
H	-1.36388800	-5.47031800	2.89809700
C	-1.00291000	-3.46468400	3.69820400
H	-0.84259900	-3.84366500	4.71092000
H	-0.25826900	-2.67575500	3.53998500
C	-2.41589900	-2.85720800	3.59303000
H	-2.49568300	-1.99863000	4.26511600
H	-3.14866600	-3.59329700	3.94363500
C	-2.80620300	-2.42411700	2.15983000
H	-3.84913100	-2.10659600	2.14962900

H	-2.19900900	-1.57595900	1.82748400
C	-2.57151000	-3.62660700	1.25463300
H	-3.18475100	-4.46079000	1.63321300
C	0.19063100	-5.63084100	-0.21179700
H	0.37773500	-6.36169800	0.57340700
H	-0.00833700	-6.17796600	-1.13518200
C	1.40540300	-4.72854700	-0.38741400
C	1.29956400	-3.59882900	-1.20775400
C	2.35622900	-2.71461700	-1.37013100
C	3.57689000	-2.89539500	-0.70716500
C	3.70089300	-4.05075300	0.06751100
C	2.64413700	-4.94453000	0.22061200
C	4.74876300	-1.93410000	-0.80287300
H	5.61914400	-2.38522900	-0.31306400
H	5.01302200	-1.75893700	-1.84682300
N	4.47892000	-0.62296000	-0.21682900
N	4.75948100	1.56398800	0.11004200
N	-1.69896400	4.18550400	-0.20954000
N	-3.73349900	3.33584300	0.11624200
N	-2.78123700	-3.56494800	-0.20766000
N	-1.02386700	-4.89605500	0.11831100
S	5.52492100	0.72496100	-2.35946200
S	-3.38958400	4.42394500	-2.35087100
S	-2.13551400	-5.14421300	-2.34948900
H	2.77414600	2.02935000	-1.72910500
H	1.35920100	6.13573200	0.58409800
Br	0.03247100	2.42216300	-2.55635900

	Br	4.14526700	5.81709400	1.32038200
	H	-3.14427400	1.38780700	-1.72548800
	H	-6.00397100	-1.89088000	0.57417800
	Br	-2.11816300	-1.18342800	-2.55847300
	Br	-7.12114900	0.68043600	1.30983900
	H	0.36920300	-3.41556600	-1.72924600
	H	4.63801800	-4.24475800	0.57425300
	Br	2.08476500	-1.24391800	-2.56906200
	Br	2.96844600	-6.49336900	1.32102100
Conf. 2	C	-6.31538600	-1.17834400	1.48135900
	C	-4.89876100	1.20304100	2.72906300
	C	-5.32430900	1.09907500	1.27215900
	C	-5.24481200	-0.34353700	0.79182800
	H	-7.30847600	-0.77900200	1.25000900
	H	-6.29208500	-2.21900600	1.15286000
	H	-4.92891700	2.23614200	3.08492400
	H	-6.37138000	1.43456200	1.20027100
	N	-4.57683100	1.78191900	0.20263100
	H	-4.25977300	-0.75572300	1.06162000
	N	-5.26681800	-0.14586900	-0.66822500
	H	-3.87480200	0.83862400	2.85735900
	C	-1.66722100	-2.38377000	-1.85848000
	C	-2.91862200	-4.26939800	-1.12292100
	C	-1.67355700	-3.71876300	-1.43433800
	C	-4.08904400	-3.51812800	-1.19152300
	C	-4.07486400	-2.16675000	-1.54646000
	C	-2.82894500	-1.62635700	-1.89538300

C	5.09632000	-1.49941600	1.26533300
C	4.47037100	-2.29805200	0.30462100
C	4.22594500	-1.69778400	-0.93695000
C	-2.27707000	4.74697100	0.79319600
C	-1.89640500	2.40327300	0.83615100
C	-2.78884000	3.45611600	0.64082400
C	-6.03045700	-1.10313400	3.00012100
C	-5.89331400	0.33703100	3.53748500
H	-5.10440200	-1.65218200	3.20679500
H	-6.82431100	-1.62042800	3.54511300
H	-6.87504400	0.82386700	3.51661200
H	-5.58378100	0.30714000	4.58529300
C	4.54289700	-0.36864500	-1.17888700
C	5.37410600	-0.15597000	1.03478900
C	5.07717500	0.46125200	-0.18456400
C	1.33660100	4.60915700	-1.66566700
C	4.13890200	3.92954400	-2.60124900
C	3.44381900	3.29766600	-1.40215200
C	2.51499600	4.31210800	-0.74791600
H	0.77272100	3.69430000	-1.87179700
H	0.64684100	5.32716800	-1.21958100
H	4.83072100	3.23552800	-3.08194500
H	2.84012800	2.45036600	-1.75215300
N	4.23545500	2.85638300	-0.23865800
H	3.06890800	5.25363400	-0.59566500
N	2.34137200	3.69680600	0.58061000
H	4.71388700	4.80485000	-2.28075200

C	-0.93873100	4.97567400	1.09028500
C	-0.03754900	3.91935000	1.26448400
C	-0.56380300	2.62834400	1.15534500
C	1.92437000	5.20239500	-2.96709100
C	3.02966600	4.33349800	-3.59947100
H	2.33340500	6.19432500	-2.74178100
H	1.12028300	5.35573900	-3.69153000
H	2.58053000	3.42003700	-4.00622900
H	3.47369300	4.86481500	-4.44548600
C	1.95491000	-3.67268000	2.94472400
C	-0.78011400	-3.33601300	1.67747600
C	0.39378100	-4.06193600	1.03146500
C	1.70750100	-3.42169300	1.46329900
H	1.99776500	-4.74984300	3.13903400
H	2.89941900	-3.23792600	3.27591900
H	-1.73584900	-3.77074300	1.38221200
H	0.38092100	-5.10706600	1.38297400
N	0.58286500	-4.10086900	-0.43244900
H	1.64146900	-2.33529000	1.30014500
N	2.62314600	-3.97827000	0.45605700
H	-0.78616400	-2.28376700	1.37661600
C	0.78311600	-3.02119100	3.71244500
C	-0.60219700	-3.47009200	3.20812900
H	0.86695700	-1.93250000	3.61657000
H	0.87474500	-3.24579500	4.77839000
H	-0.76292300	-4.51858900	3.48564600
H	-1.38052000	-2.89352700	3.71520400

C	-4.74508200	1.08539800	-0.96473800
S	-4.38640400	1.67167200	-2.50922100
C	-5.30491400	-1.26834600	-1.60385700
H	-6.20427400	-1.84661800	-1.40094900
H	-5.39987800	-0.84181600	-2.60309100
C	-4.24016900	3.19226700	0.26476400
H	-4.44441800	3.64135100	-0.70985200
H	-4.90199900	3.67672300	0.98849500
C	3.52025600	3.06825600	0.90629600
S	4.03298700	2.68054500	2.46903000
C	1.41800400	4.22619800	1.57438500
H	1.70444700	3.78607900	2.53136100
H	1.54242500	5.31154400	1.66386400
C	5.38357300	1.94951600	-0.33917600
H	5.85008200	2.13833200	-1.30600300
H	6.10024100	2.23330800	0.43168500
C	1.92306000	-4.27117000	-0.68126000
S	2.59828300	-4.79894800	-2.14118300
C	-0.44229100	-4.59984200	-1.34225700
H	-0.75179500	-5.60726300	-1.03997100
H	0.03292900	-4.68743300	-2.32105700
C	4.06665700	-3.75398600	0.50409600
H	4.50651800	-4.36828900	-0.28213700
H	4.43350000	-4.12098900	1.46090700
H	3.79586100	-2.30041300	-1.72617800
H	5.82010200	0.43848500	1.81890400
Br	4.28701600	0.24227800	-2.98787500

	Br	5.65088900	-2.20845800	2.97136500
	H	-2.78538300	-0.59487000	-2.22025000
	H	-2.97006500	-5.30640900	-0.81550200
	Br	-5.73057200	-4.44852000	-0.79425600
	Br	-0.05525300	-1.53709800	-2.46625300
	H	-2.25880700	1.39076000	0.73513000
	H	-0.57942600	5.99348300	1.17989200
	Br	0.51544800	1.07240100	1.47075200
	Br	-3.42277400	6.27695300	0.56831500
Conf. 3	C	-4.93767700	2.12286400	2.25021200
	C	-2.10659900	3.20350400	2.44182100
	C	-3.08067600	3.51792400	1.31396600
	C	-3.99956000	2.32980700	1.06953800
	H	-5.55067900	3.01746400	2.40302900
	H	-5.61580800	1.28281600	2.08350700
	H	-1.43623800	4.03952800	2.64386100
	H	-3.69329800	4.37984000	1.62595800
	N	-2.62652600	3.79191700	-0.06536300
	H	-3.37968900	1.42704300	0.95845600
	N	-4.54558900	2.67417200	-0.25056900
	H	-1.48722300	2.33985900	2.18041800
	C	-3.33947300	-1.15734100	-1.77238200
	C	-5.23110100	-2.00688300	-0.60444400
	C	-3.98465900	-2.25219800	-1.18324100
	C	-5.76701100	-0.72295100	-0.54464600
	C	-5.07741100	0.38425200	-1.04419700
	C	-3.86187100	0.12502300	-1.69145100

C	3.19755500	-4.26344000	1.15216100
C	2.14393300	-4.34496200	0.23748700
C	2.17444000	-3.44041500	-0.82981200
C	0.49689100	5.05227300	0.98468100
C	0.47253400	3.32506600	-0.64923600
C	-0.20472500	4.37277300	-0.01763700
C	-4.04839100	1.84680700	3.48333300
C	-2.95871000	2.91481200	3.70051000
H	-3.57248000	0.86749500	3.35660200
H	-4.67293100	1.77464000	4.37768500
H	-3.43313400	3.85034900	4.01957500
H	-2.30208500	2.60657000	4.51828300
C	3.18878400	-2.50024500	-0.95855100
C	4.18977400	-3.29755600	1.04013600
C	4.20860700	-2.37614600	-0.01002400
C	5.36807900	3.36613700	-1.63963200
C	6.83297200	0.72387300	-1.97080700
C	5.50174500	0.90265600	-1.25471000
C	5.44357900	2.26948200	-0.58748100
H	4.48374900	3.23017000	-2.26999600
H	5.30117300	4.35789100	-1.18487400
H	6.90308300	-0.25021800	-2.46157400
H	4.70287600	0.84766100	-2.00446500
N	5.11777500	0.02668700	-0.12707600
H	6.36610800	2.42303800	-0.00349100
N	4.34885800	2.05131000	0.37399900
H	7.66193200	0.79842300	-1.25901100

C	1.78088600	4.67162400	1.35715100
C	2.43470100	3.59307200	0.75648500
C	1.74911800	2.93699600	-0.26437400
C	6.66335400	3.25754500	-2.47735500
C	6.91648300	1.84207800	-3.03711400
H	7.51037300	3.55264900	-1.84725400
H	6.62669500	3.97309900	-3.30275300
H	6.17887600	1.62960100	-3.81936000
H	7.89681300	1.81131400	-3.51960100
C	-0.49937900	-4.17614300	2.89020500
C	-2.75368700	-2.43299600	1.85053900
C	-2.19991400	-3.65437400	1.12627100
C	-0.71508500	-3.80546600	1.42984200
H	-1.00732100	-5.11950700	3.11818000
H	0.55983800	-4.30704500	3.12021700
H	-3.81723700	-2.29345000	1.65697700
H	-2.72911400	-4.54452900	1.50503100
N	-2.18509600	-3.75634600	-0.34934900
H	-0.21671000	-2.84237700	1.24140100
N	-0.31671300	-4.73700700	0.36641900
H	-2.23485300	-1.52907200	1.51605100
C	-1.08040700	-3.01993600	3.73374900
C	-2.53440600	-2.66871500	3.36327300
H	-0.44772800	-2.13605900	3.59339900
H	-1.02471700	-3.27634100	4.79498500
H	-3.19375100	-3.48303700	3.68645800
H	-2.84803700	-1.77977600	3.91714300

C	-3.65038700	3.46739300	-0.92069100
S	-3.82021300	3.98585300	-2.52113800
C	-5.54121000	1.82851100	-0.90400200
H	-6.46648900	1.87309500	-0.33188000
H	-5.73338500	2.26370900	-1.88576000
C	-1.61912600	4.80523800	-0.36733300
H	-1.70135000	5.01609000	-1.43465200
H	-1.85365400	5.72978900	0.17211200
C	4.38961300	0.73581200	0.77785000
S	3.67988000	0.12487900	2.18156900
C	3.81516400	3.12301900	1.19294300
H	3.76146000	2.77738700	2.22892400
H	4.51549800	3.96231100	1.16781700
C	5.38603000	-1.40767100	-0.06883800
H	5.98690500	-1.63908100	-0.95005000
H	6.01212500	-1.60839300	0.80349600
C	-1.15226800	-4.59421900	-0.70410100
S	-0.95695400	-5.35477900	-2.20308000
C	-3.40910600	-3.65508900	-1.14305300
H	-4.16701000	-4.33908200	-0.74332800
H	-3.15580900	-3.99625200	-2.14766700
C	1.00445200	-5.35094000	0.32233100
H	1.01886900	-6.00343900	-0.55201000
H	1.12251600	-5.97519700	1.20669600
H	1.39541300	-3.49322800	-1.57979100
H	4.96583500	-3.24552500	1.79230900
Br	3.14693100	-1.40941700	-2.54093200

	Br	3.35400300	-5.49075400	2.63189700
	H	2.22872300	2.10572000	-0.76066200
	H	0.02249600	5.88609700	1.48731300
	Br	-0.30542300	2.36408400	-2.11417500
	Br	2.64138300	5.66694500	2.76243200
	H	-3.31862900	0.95085600	-2.13218200
	H	-5.77958700	-2.83322100	-0.16947900
	Br	-7.50226200	-0.54539800	0.27612600
	Br	-1.70634500	-1.38760800	-2.75095800
Conf. 4	C	4.80198800	1.23354600	-0.94472200
	C	3.99587600	1.98133300	1.09604300
	H	2.92944400	1.71231200	1.04498500
	C	4.18707800	2.93357100	2.26748000
	H	3.62209700	3.85910900	2.13168300
	H	5.24389900	3.20175700	2.36756800
	C	3.68430300	2.19026600	3.52791500
	H	3.88148700	2.79904300	4.41398100
	H	2.59520900	2.08728100	3.45735700
	C	4.31250700	0.79320000	3.71400600
	H	5.36885000	0.90727900	3.98303500
	H	3.83206000	0.28769000	4.55572300
	C	4.22248000	-0.10069300	2.45461000
	H	4.77748500	-1.02720200	2.61832600
	H	3.18281200	-0.36898100	2.24010000
	C	4.80505500	0.71050300	1.30688400
	H	5.83723400	0.98869900	1.57518300
	C	4.06940300	3.62800300	-0.86922700

H	4.51787700	3.64047100	-1.86473900
H	4.52828900	4.42948800	-0.29166800
C	2.56844300	3.86100300	-0.97616700
C	1.74670800	2.84095400	-1.46983800
C	0.37309100	3.00319900	-1.59095700
C	-0.26319200	4.19207800	-1.21107200
C	1.93843200	5.06032200	-0.63483400
C	0.56044500	5.22058500	-0.74690200
C	-1.75605000	4.44437700	-1.32177600
H	-1.95699200	5.48815800	-1.05361700
H	-2.08456900	4.30399600	-2.35298400
C	-3.79962700	3.09232300	-0.93178200
C	-3.56624000	2.51377900	1.29926000
H	-2.99738700	1.57169400	1.25837100
C	-4.12794200	2.67605200	2.70523700
H	-4.67505900	3.62162800	2.78271800
H	-4.82168800	1.87204400	2.95865100
C	-2.92491700	2.65498900	3.67476600
H	-2.50236300	1.64337900	3.68258000
H	-3.27092200	2.85392500	4.69243200
C	-1.82047400	3.66289300	3.29960900
H	-2.18562900	4.67969100	3.48522100
H	-0.95690000	3.52060700	3.95475600
C	-1.36640100	3.56861400	1.82402100
H	-0.84962300	2.62238600	1.63370900
H	-0.66679600	4.37615900	1.60623900
C	-2.62305600	3.66312800	0.96817700

H	-3.12744700	4.61325300	1.20963000
C	-5.61052100	1.65114500	0.02341200
H	-6.13869200	1.92105600	-0.89162500
H	-6.27744100	1.83686400	0.86320600
C	-5.20351800	0.18392100	-0.02177200
C	-4.39834200	-0.24958400	-1.08348300
C	-3.91532000	-1.54714800	-1.14923500
C	-4.17401500	-2.48175200	-0.13772100
C	-5.04592900	-2.07552700	0.87461000
C	-5.55570900	-0.78000100	0.92561800
C	-3.56338700	-3.87116400	-0.08715500
H	-4.07043400	-4.45677400	0.68854900
H	-3.70179200	-4.39056700	-1.03580700
C	-1.24862600	-4.71671000	-0.45145600
C	-0.09259000	-3.66323900	1.26678300
H	0.24144700	-2.70231300	0.84794200
C	0.64589700	-3.90085900	2.58040600
H	1.72266100	-3.99132700	2.43204000
H	0.29473200	-4.82951200	3.04226900
C	0.34797000	-2.68744100	3.48849800
H	0.79255300	-2.84912600	4.47411100
H	0.84482400	-1.80706600	3.06408200
C	-1.15695900	-2.39650400	3.64360300
H	-1.29537300	-1.46221100	4.19447000
H	-1.61231600	-3.18498100	4.25406400
C	-1.92135500	-2.31750500	2.30107400
H	-2.98846300	-2.23040100	2.50342300

H	-1.62069800	-1.43445900	1.72805300
C	-1.59244200	-3.58755300	1.52569000
H	-1.88289400	-4.44694700	2.15309400
C	1.20755600	-5.19713700	-0.35186400
H	1.54977100	-6.03065800	0.26665100
H	0.97170500	-5.59677800	-1.33926000
C	2.32630500	-4.16979800	-0.43228000
C	2.31059800	-3.04112700	-1.26345600
C	3.32073000	-2.09289800	-1.22482500
H	3.26297300	-1.22024400	-1.86270300
C	4.42706500	-2.23214600	-0.37964000
C	4.48959200	-3.39845400	0.38367900
C	3.45924800	-4.33651300	0.36878500
H	3.52904300	-5.21111900	1.00320600
C	5.45372500	-1.11188700	-0.36465200
H	6.23489100	-1.31016800	0.37043100
H	5.92722800	-1.03695800	-1.34557200
N	4.84421400	0.18076100	-0.06729900
N	4.43000200	2.35694500	-0.25861100
N	-2.57936400	3.55893800	-0.50398400
N	-4.46527000	2.55407600	0.13732100
N	-2.12344700	-3.84977100	0.17226300
N	-0.04599400	-4.66932200	0.19694600
S	5.15282500	1.15346200	-2.59727300
S	-4.40431300	3.19811800	-2.50750600
S	-1.61370800	-5.72510100	-1.75607300
Br	0.89201900	-2.75265100	-2.51605900

	Br	5.99742500	-3.77446500	1.52255300
	H	2.20007900	1.90829700	-1.77908500
	H	0.11020900	6.16210900	-0.45827900
	Br	-0.61329300	1.53717000	-2.33807500
	Br	2.93865000	6.57485400	0.01295300
	H	-4.15489900	0.45373500	-1.86921100
	H	-5.31347200	-2.78180000	1.65065600
	Br	-2.91969100	-2.02362600	-2.71634600
	Br	-6.76917200	-0.38306700	2.37043500

4 (*in vacuo*)

Conf. 1	C	-1.00535700	7.56577000	3.33506700
	C	-1.35483100	8.09824900	1.92772600
	C	-0.57813000	7.25871800	0.92389200
	C	0.92579100	7.39517400	1.17060100
	C	1.30025700	6.74669300	2.49560300
	H	-1.08102300	9.15355800	1.82812700
	H	-2.43441900	8.02208800	1.76293400
	H	-0.85704000	6.20251900	1.07871900
	N	-0.64967600	7.56005600	-0.50385600
	N	1.46089200	6.88266300	-0.08834000
	H	1.16388200	8.47292000	1.24250000
	C	0.51357700	7.48462400	3.60421200
	C	0.54186800	7.15268300	-1.09909100
	O	0.75171900	7.05327500	-2.29608400
	C	-1.89226800	7.53752900	-1.26712600
	C	2.89024000	6.92672000	-0.40497600
	C	3.54758700	5.56252400	-0.49413100

C	3.26383300	4.71977300	-1.57360200
C	4.45837100	5.12483600	0.46585900
C	5.07304100	3.88139300	0.35620500
C	4.80041600	3.03342700	-0.72509400
C	3.87893100	3.47928600	-1.68671800
C	5.47532700	1.71852400	-0.85934800
C	4.80579900	0.60428600	-1.38740100
C	6.81131500	1.54918200	-0.47143400
C	7.44364800	0.31603900	-0.59664900
C	6.77395100	-0.78956500	-1.12512100
C	5.44179200	-0.62318800	-1.52397000
C	-2.70368000	6.26015200	-1.12601600
C	-3.99616900	6.28833500	-0.59694100
C	-2.18058700	5.02551600	-1.52373100
C	-2.92588400	3.85902800	-1.38753900
C	-4.22669500	3.88191200	-0.85912400
C	-4.74795700	5.12338700	-0.47113500
C	-5.02852700	2.64133700	-0.72511300
C	-4.95378100	1.61985700	-1.68811200
C	-5.89665100	2.45326800	0.35640500
C	-6.66667100	1.29880200	0.46648900
C	-6.59203600	0.28974400	-0.49406700
C	-5.71879100	0.46575600	-1.57423200
H	-1.60026500	7.68936800	-2.30890800
H	-2.50486400	8.39249300	-0.96666600
H	3.39263200	7.53035500	0.35739700
H	3.00022200	7.44125600	-1.36418900

H	2.56542100	5.05461200	-2.33418100
H	4.69274700	5.76217900	1.31479000
H	5.75936000	3.55605600	1.13106900
H	3.67006300	2.86176900	-2.55274300
H	3.76601400	0.69597600	-1.68144800
H	7.36938600	2.39774200	-0.09062600
H	8.48180200	0.21785200	-0.29547500
C	7.47364800	-2.12896900	-1.26640900
H	4.90289500	-1.46396100	-1.94711000
H	-4.43004600	7.23742600	-0.29668300
H	-1.18240400	4.97895900	-1.94690100
H	-2.48652800	2.91405800	-1.68298500
H	-5.76151000	5.18434000	-0.09113300
H	-4.31204400	1.74731800	-2.55269400
H	-5.96011400	3.21005600	1.13174100
H	-7.33568000	1.18372800	1.31470900
C	-7.44458500	-0.96016500	-0.40520200
H	-5.66036200	-0.30494400	-2.33348400
N	-6.69147300	-2.17690100	-0.08844700
C	-6.46545900	-3.10790200	-1.09920400
N	-6.22229900	-4.34159300	-0.50383200
C	-5.99734800	-4.12952100	0.92271600
C	-6.86741600	-2.89697200	1.16981500
C	-6.33599100	-5.22274800	1.92783100
C	-5.58055200	-5.40875300	-1.26718100
C	-4.06905900	-5.47164600	-1.12467300
C	-3.26172800	-4.40118300	-1.52483100

C	-1.87910300	-4.46355500	-1.38714600
C	-1.24992800	-5.60131700	-0.86021700
C	-2.06352200	-6.67308500	-0.47264100
C	-3.44741100	-6.60505900	-0.59806800
C	0.22778700	-5.67501000	-0.72604500
C	0.82457200	-6.33380300	0.35687700
C	2.20937600	-6.42325200	0.46659600
C	3.04521100	-5.85292700	-0.49439700
C	2.45584900	-5.18566000	-1.57449000
C	1.07329400	-5.09913900	-1.68714300
N	6.87221000	-3.21885800	-0.50446300
C	5.92372800	-4.04444000	-1.09896200
N	5.22980600	-4.70620700	-0.08939300
C	5.94282100	-4.49928900	1.17018200
C	6.57509700	-3.12788000	0.92258700
C	7.05449200	-2.91216000	3.33632700
C	7.69133700	-2.87485200	1.92689300
O	-6.48307200	-2.87425300	-2.29688200
O	5.73172700	-4.17784500	-2.29719000
C	4.55273700	-5.96627400	-0.40390000
H	8.52074000	-2.02740000	-0.96752400
H	7.45899800	-2.45912600	-2.30823700
H	-7.94329000	-1.12204900	-1.36350100
H	-8.21794400	-0.82790000	0.35808200
H	-4.94374400	-3.84429700	1.07915500
C	-6.49237000	-2.24818100	2.49664900
H	-7.38667900	-5.51206400	1.82869200

H	-6.01543800	-6.36479800	-0.96778600
H	-5.85977300	-5.23037900	-2.30842000
H	-3.71991000	-3.51341300	-1.94789000
H	-1.27917300	-3.60965100	-1.68270500
H	-1.60954300	-7.58075200	-0.09130700
H	-4.05318700	-7.45357000	-0.29601900
H	0.20045000	-6.76673300	1.13111000
H	2.64280400	-6.94507400	1.31480100
H	3.09450300	-4.74901400	-2.33365800
H	0.64279800	-4.60877900	-2.55258600
H	6.75451600	-5.24423100	1.24200100
H	5.80088400	-2.35885200	1.07949200
H	7.83278200	-2.81700100	4.09817600
H	8.46831400	-3.64157700	1.82805000
H	8.16542500	-1.90342000	1.76166200
H	4.94327400	-6.31935000	-1.36291100
H	4.82623800	-6.70296100	0.35743900
C	-6.74009200	-3.29836700	3.60427500
C	-6.05007100	-4.65207800	3.33617200
C	5.19326300	-4.49867700	2.49670300
C	6.22473400	-4.18830300	3.60431800
H	-1.43962600	6.56449700	3.44406700
H	-1.47722400	8.19176200	4.09809800
H	2.37359300	6.82009900	2.69038000
H	1.04700600	5.68232400	2.47566400
H	0.91430700	8.50337800	3.69520800
H	0.68780200	7.00078800	4.56850200

	H	-7.92014100	-3.22834600	1.24322700
	H	-5.73159900	-6.11897200	1.76237200
	H	6.40505100	-2.03551100	3.44433500
	H	-7.09401700	-1.35487400	2.69063800
	H	-5.44453200	-1.93511200	2.47617300
	H	-7.81983600	-3.45956800	3.69487100
	H	-6.40654500	-2.90433900	4.56941000
	H	-6.35596900	-5.37374300	4.09790400
	H	-4.96605600	-4.52923900	3.44577000
	H	4.71872400	-5.46660400	2.69009800
	H	4.39721200	-3.74779700	2.47512900
	H	6.90729500	-5.04352900	3.69382700
	H	5.71794100	-4.09569600	4.56810500
Conf. 2	C	7.86856400	1.27981900	2.94294900
	C	8.25412400	1.36732300	1.44877700
	C	7.31826600	0.43733700	0.68756000
	C	7.47868000	-1.00094500	1.18436000
	C	6.96689300	-1.11923300	2.61381300
	H	9.29549100	1.06376200	1.29366800
	H	8.15757000	2.39996400	1.10212000
	H	6.28434400	0.75808200	0.89457300
	N	7.47856400	0.24690900	-0.75103000
	N	6.83484000	-1.73986100	0.09942200
	H	8.55681700	-1.24314100	1.19054200
	C	7.81655900	-0.16527100	3.48426400
	C	7.02347100	-1.02634300	-1.08193100
	O	6.83537200	-1.45236900	-2.20903800

C	7.39260000	1.33122700	-1.72375200
C	6.83784000	-3.20648800	0.04187100
C	5.45575100	-3.82436500	-0.05087100
C	4.79859900	-3.90384100	-1.28290900
C	4.81467800	-4.34353700	1.07524500
C	3.55560400	-4.92850600	0.97847100
C	2.89629100	-5.02301900	-0.25501500
C	3.54478600	-4.49449300	-1.38203700
C	1.56499200	-5.66997100	-0.37049900
C	0.60584500	-5.20256200	-1.28403700
C	1.22486500	-6.77823100	0.41614400
C	-0.02275900	-7.38537100	0.29951400
C	-0.97122000	-6.91836500	-0.61179000
C	-0.63414000	-5.81642000	-1.40792400
C	6.16664900	2.21661500	-1.58187800
C	6.29772000	3.59155000	-1.38064500
C	4.87514000	1.68170200	-1.65236400
C	3.75844900	2.49583200	-1.50868700
C	3.88483400	3.87958000	-1.30319600
C	5.18017100	4.41117500	-1.25208200
C	2.69103200	4.74725300	-1.14257500
C	1.50054300	4.48580500	-1.84118500
C	2.71157800	5.85746400	-0.28865800
C	1.59166800	6.67209000	-0.14205300
C	0.41027200	6.40244500	-0.83227400
C	0.38269000	5.29423600	-1.68551100
H	7.40255600	0.84533500	-2.70234900

H	8.29457300	1.94585400	-1.65234900
H	7.34224200	-3.57180500	0.93987900
H	7.42955100	-3.51823300	-0.82407500
H	5.28284300	-3.49592200	-2.16266400
H	5.30274100	-4.29116100	2.04381600
H	3.07249300	-5.30152300	1.87444400
H	3.07484600	-4.57483200	-2.35550200
H	0.83089600	-4.33805900	-1.89798800
H	1.95195100	-7.18781800	1.10803500
H	-0.25240300	-8.24983000	0.91535800
C	-2.32916400	-7.58592800	-0.74448200
H	-1.35056100	-5.44503800	-2.13256500
H	7.28889900	4.03294700	-1.33760100
H	4.74434200	0.61809500	-1.82030100
H	2.77181600	2.04794000	-1.53771800
H	5.31710300	5.47948000	-1.12988800
H	1.46023200	3.65478500	-2.53596900
H	3.60413900	6.07416700	0.28696300
H	1.63939500	7.52614900	0.52680600
C	-0.81066700	7.29020600	-0.69553500
H	-0.52212700	5.07708500	-2.24302500
N	-1.97231300	6.61164100	-0.13006200
C	-3.17971400	6.62887200	-0.82804700
N	-4.20762200	6.39009200	0.08121300
C	-3.61631300	5.91622500	1.33040800
C	-2.26596600	6.63444700	1.29981100
C	-4.31828200	6.17357200	2.65749300

C	-5.50252700	5.91535600	-0.40754000
C	-5.59773200	4.40746900	-0.56049400
C	-4.85739300	3.74342500	-1.54661600
C	-4.91230700	2.36057200	-1.66549700
C	-5.71198800	1.58690800	-0.80915500
C	-6.46524600	2.25735300	0.16259900
C	-6.40562800	3.64340400	0.28351600
C	-5.74981700	0.10809300	-0.92775000
C	-6.94583900	-0.60561100	-0.78068800
C	-6.96887600	-1.99384300	-0.88675900
C	-5.80234800	-2.71596700	-1.14285700
C	-4.60592600	-2.00831700	-1.28996800
C	-4.57999100	-0.62377800	-1.18488400
N	-3.46204300	-6.74535500	-0.36201200
C	-4.04452100	-5.90563200	-1.30636700
N	-4.76272000	-4.92084400	-0.62275300
C	-4.87365100	-5.32531800	0.77669900
C	-3.57625500	-6.11720900	0.95137800
C	-3.84872300	-6.02111600	3.40515000
C	-3.64760500	-6.97656900	2.20691200
O	-3.30755000	6.81660400	-2.02562000
O	-3.94435000	-6.00458200	-2.51632300
C	-5.83832300	-4.22273300	-1.31156500
H	-2.35363100	-8.49688000	-0.14065500
H	-2.51038200	-7.87169900	-1.78252900
H	-1.11759400	7.64744000	-1.68179300
H	-0.56633500	8.16893400	-0.08445400

H	-3.43012400	4.83275700	1.25085500
C	-1.31157400	5.99849000	2.30302800
H	-4.49298800	7.24739800	2.78731300
H	-6.28058700	6.27043500	0.27312400
H	-5.65425100	6.40624000	-1.37051900
H	-4.24498400	4.32218900	-2.22925500
H	-4.34803200	1.87224500	-2.45193300
H	-7.08086900	1.68742200	0.84955800
H	-6.99480200	4.13572200	1.05143900
H	-7.87095000	-0.06872500	-0.60311600
H	-7.91197500	-2.52069900	-0.77594000
H	-3.68757500	-2.55350600	-1.47525400
H	-3.63520500	-0.10069700	-1.28131800
H	-5.72106800	-6.02885500	0.87078700
H	-2.74960400	-5.39912500	1.07910200
H	-3.98715300	-6.60025000	4.32273200
H	-4.47819400	-7.68692500	2.12883200
H	-2.73137500	-7.55729700	2.34670100
H	-5.73199300	-4.47880200	-2.36974000
H	-6.81216700	-4.60855700	-0.97867700
C	-1.95357000	6.14819600	3.70066000
C	-3.40313300	5.62192700	3.77460300
C	-5.00910100	-4.28553100	1.88189800
C	-5.03455300	-5.04926100	3.22515700
H	6.88440200	1.74534300	3.07355400
H	8.56898900	1.86820400	3.54264600
H	7.05634600	-2.14300300	2.98970000

	H	5.90662200	-0.84875000	2.65428500
	H	8.83727800	-0.56256700	3.54141300
	H	7.43280000	-0.15685100	4.50845800
	H	-2.42589500	7.68414200	1.60733200
	H	-5.29144000	5.67613200	2.69829000
	H	-2.92880300	-5.44040500	3.54274700
	H	-0.33168500	6.48261900	2.28852300
	H	-1.15308800	4.94442100	2.05303300
	H	-1.94563600	7.21026500	3.97514800
	H	-1.34115100	5.63402000	4.44693500
	H	-3.82809000	5.85980700	4.75405000
	H	-3.39021600	4.52791100	3.70162700
	H	-5.92230300	-3.69582000	1.76810300
	H	-4.16742000	-3.58652900	1.84341000
	H	-5.97238200	-5.61501500	3.28702700
	H	-5.05075900	-4.33741600	4.05543100
Conf. 3	C	-4.87338400	-5.35694200	3.34429100
	C	-4.96676300	-6.21629300	2.06265800
	C	-4.80845000	-5.27470900	0.87621300
	C	-5.91921800	-4.22224800	0.88355300
	C	-5.74935300	-3.28482000	2.07203300
	H	-5.93152600	-6.73260900	2.00623400
	H	-4.18492000	-6.98100500	2.07225800
	H	-3.84240100	-4.75508800	0.98425300
	N	-4.92466800	-5.78356000	-0.48804100
	N	-5.83124500	-3.71822100	-0.48429200
	H	-6.88650800	-4.74386200	1.00218200

C	-5.83642000	-4.15022400	3.34965500
C	-5.38565900	-4.75638000	-1.30591600
O	-5.39538100	-4.75531100	-2.52393900
C	-4.03298600	-6.80069500	-1.03956800
C	-6.78434200	-2.75327000	-1.01330300
C	-6.37258700	-1.30646000	-0.82055200
C	-5.06788500	-0.89442700	-1.10673700
C	-7.29570500	-0.34152400	-0.41524600
C	-6.93351600	0.99914500	-0.30868400
C	-5.63091600	1.41860300	-0.60599100
C	-4.70427600	0.44141100	-1.00209200
C	-5.23760100	2.84692400	-0.52020200
C	-4.40226000	3.41897100	-1.49295000
C	-5.68508000	3.66966000	0.52086300
C	-5.30163600	5.00715500	0.59098900
C	-4.46421500	5.56940500	-0.37432100
C	-4.02523500	4.75367500	-1.42536800
C	-2.55336700	-6.47412400	-0.93052200
C	-1.68779800	-7.29184100	-0.20259500
C	-2.01674600	-5.34589800	-1.56306200
C	-0.66592100	-5.04150500	-1.45047200
C	0.20937700	-5.85933200	-0.71687600
C	-0.33088500	-6.99730100	-0.10302100
C	1.65340100	-5.53435300	-0.59908900
C	2.34963100	-4.91107100	-1.64742400
C	2.37332800	-5.84641600	0.56227700
C	3.73103000	-5.55889600	0.66457500

C	4.41612100	-4.93662700	-0.37948300
C	3.70235300	-4.61363500	-1.53819400
H	-4.32173200	-6.90705400	-2.08745300
H	-4.23686700	-7.75254300	-0.54178900
H	-7.77361400	-2.92149500	-0.56615200
H	-6.86923700	-2.96765400	-2.08275000
H	-4.33366400	-1.63265400	-1.40810600
H	-8.31676600	-0.63629500	-0.19204700
H	-7.67821200	1.73166000	-0.01793400
H	-3.68198500	0.73296500	-1.21518100
H	-4.07088900	2.81587900	-2.33070000
H	-6.31799300	3.25403800	1.29715500
H	-5.66101100	5.62026600	1.41216700
C	-4.02683600	7.02195500	-0.29503500
H	-3.39419500	5.17870100	-2.19771100
H	-2.07462200	-8.18369200	0.28141400
H	-2.66529300	-4.70687400	-2.15244100
H	-0.28569300	-4.14620900	-1.92869800
H	0.31891500	-7.67311000	0.44092200
H	1.83222900	-4.68386400	-2.57234000
H	1.86377800	-6.30344000	1.40274600
H	4.26189600	-5.82222700	1.57428700
C	5.89702200	-4.62590600	-0.28660300
H	4.22244300	-4.13590100	-2.36170800
N	6.19778000	-3.22172600	0.00401200
C	6.78250100	-2.44009200	-0.99119600
N	7.44269100	-1.38075800	-0.37526600

C	7.01285800	-1.31680300	1.01898100
C	6.72926400	-2.79438500	1.29562200
C	7.94268500	-0.75189700	2.08530200
C	7.82345400	-0.20269200	-1.15156000
C	6.78173100	0.90122200	-1.18174300
C	5.58169400	0.73807300	-1.88432300
C	4.62283600	1.74374500	-1.89745700
C	4.82203800	2.95102400	-1.20766700
C	6.02796400	3.11055900	-0.51259200
C	6.98905200	2.10405200	-0.50407300
C	3.79262300	4.02038100	-1.20892600
C	4.15096700	5.37566300	-1.21250900
C	3.18314600	6.37462000	-1.18557000
C	1.82274500	6.06043900	-1.16512200
C	1.45849200	4.71182300	-1.17392200
C	2.42397400	3.71198600	-1.19137700
N	-2.60858300	7.21181000	0.01225000
C	-1.67136900	7.12042600	-1.01308600
N	-0.42590200	6.85882400	-0.43678500
C	-0.53186700	7.09843400	1.00107300
C	-1.99522000	6.72321400	1.24487000
C	-1.56060100	6.62528600	3.67629700
C	-2.46016000	7.26058900	2.59202000
O	6.72953500	-2.65834800	-2.18993800
O	-1.89447600	7.24339900	-2.20432700
C	0.77982700	7.16288900	-1.18953200
H	-4.61457400	7.54946400	0.46025300

H	-4.19437900	7.51906200	-1.25217100
H	6.37519800	-4.86797200	-1.23880700
H	6.35956600	-5.24595900	0.48866700
H	6.05909200	-0.76699100	1.07607900
C	5.90023100	-2.94033100	2.56525300
H	8.89358300	-1.29630600	2.08106800
H	8.76172900	0.18873500	-0.74917500
H	8.02190200	-0.56697400	-2.16126400
H	5.41010200	-0.18071000	-2.43484600
H	3.71651700	1.60295500	-2.47521400
H	6.20671000	4.02074300	0.04837600
H	7.91462700	2.25820000	0.04235800
H	5.19810600	5.65309200	-1.25370300
H	3.49336900	7.41535600	-1.18821800
H	0.40770100	4.44790800	-1.14881500
H	2.11086600	2.67432800	-1.16568400
H	-0.42039600	8.18293300	1.18317700
H	-2.06824800	5.62357600	1.27399000
H	-1.80785600	7.04385700	4.65613700
H	-2.38034500	8.35312600	2.60699100
H	-3.50660200	7.00683400	2.78448200
H	0.45304200	7.34030500	-2.21857000
H	1.22724300	8.09917500	-0.82625200
C	6.72752300	-2.33779900	3.72358400
C	7.22502800	-0.90326000	3.44573100
C	0.37455500	6.36876700	1.98446300
C	-0.05147900	6.80347000	3.40474700

	H	-3.84380500	-4.99243900	3.44169800
	H	-5.06376000	-5.97943900	4.22332300
	H	-6.52328200	-2.51275100	2.08764200
	H	-4.78395200	-2.77230400	2.00939600
	H	-6.86559700	-4.51525800	3.45380000
	H	-5.63983200	-3.53200300	4.23024600
	H	7.69588300	-3.30290500	1.46514900
	H	8.16760100	0.30243500	1.90258900
	H	-1.78859200	5.55409300	3.73156900
	H	5.67384300	-3.98886500	2.77857000
	H	4.94390400	-2.41989600	2.44938000
	H	7.59329500	-2.98608100	3.90607900
	H	6.13780300	-2.34705500	4.64474500
	H	7.89018900	-0.58543700	4.25384100
	H	6.36906300	-0.21829900	3.46385600
	H	1.42663800	6.61294700	1.81616200
	H	0.27187700	5.28608200	1.85905600
	H	0.21407800	7.85903700	3.54115400
	H	0.52132900	6.24447700	4.15023600
Conf. 4	C	0.98966900	6.74847600	3.56634800
	C	0.52688800	7.66321000	2.41029400
	C	0.85898500	6.94863100	1.10763100
	C	2.36654200	6.70056200	1.00932300
	C	2.80786200	5.69450500	2.06301700
	H	1.03631000	8.63091300	2.44724000
	H	-0.54713700	7.85460700	2.50052400
	H	0.34964100	5.96974900	1.11619100

N	0.60626300	7.60350100	-0.17348900
N	2.50173800	6.40240500	-0.41509100
H	2.88445700	7.65487500	1.21625200
C	2.46152200	6.29766400	3.44317800
C	1.51833800	7.11534000	-1.10600400
O	1.46679600	7.27508400	-2.31213000
C	-0.73179500	7.99403800	-0.61360900
C	3.79818400	6.19793800	-1.04277000
C	4.31863900	4.77602900	-0.93876100
C	3.47684100	3.68627700	-1.17461000
C	5.66142400	4.52410100	-0.65635600
C	6.15326000	3.22338900	-0.61061800
C	5.31458200	2.12515000	-0.84437200
C	3.96454300	2.38537500	-1.12803300
C	5.82965000	0.73462900	-0.79131000
C	5.40243900	-0.23104800	-1.71489500
C	6.75822900	0.34124900	0.18134900
C	7.22997800	-0.96736900	0.23303800
C	6.79517100	-1.92860600	-0.68226000
C	5.87516100	-1.53653500	-1.66434900
C	-1.72748300	6.84794900	-0.68315500
C	-2.77782300	6.74478400	0.23274600
C	-1.60671500	5.85763400	-1.66411000
C	-2.50054900	4.79355500	-1.71503300
C	-3.55154000	4.68050000	-0.79108600
C	-3.67501500	5.68120400	0.18164800
C	-4.49919900	3.54028900	-0.84439100

C	-4.04924900	2.24055900	-1.12942700
C	-5.86717300	3.71814500	-0.61041800
C	-6.74803900	2.64128200	-0.65572600
C	-6.29596000	1.35050000	-0.93869700
C	-4.92999100	1.16721200	-1.17523900
H	-0.60184700	8.44234800	-1.60023600
H	-1.10383200	8.77025800	0.06008900
H	4.53792400	6.89205700	-0.61723100
H	3.66810800	6.46977000	-2.09475300
H	2.42738700	3.86237800	-1.38338700
H	6.33568500	5.35611800	-0.47535600
H	7.20654200	3.05754200	-0.40986300
H	3.28599800	1.55731300	-1.29333100
H	4.71217700	0.05172600	-2.50111500
H	7.09462900	1.06025400	0.92038800
H	7.94692700	-1.24318700	0.99963800
C	7.28842800	-3.36233800	-0.61289200
H	5.54225900	-2.26265200	-2.39804200
H	-2.89758000	7.50452400	0.99843000
H	-0.81077700	5.93235600	-2.39783300
H	-2.40182000	4.05529500	-2.50265200
H	-4.46618700	5.61497300	0.91988100
H	-2.99034000	2.06738900	-1.29328200
H	-6.25204400	4.71233000	-0.40919200
H	-7.80579500	2.80983100	-0.47543700
C	-7.26743000	0.19027300	-1.04299600
H	-4.55903900	0.17152200	-1.38269000

N	-6.79571300	-1.03548500	-0.41519800
C	-6.92107600	-2.24357600	-1.10611700
N	-6.88824800	-3.27567900	-0.17346500
C	-6.44752100	-2.72998600	1.10645500
C	-6.98585900	-1.30191100	1.00853600
C	-6.89998900	-3.37563500	2.41039900
C	-6.55578600	-4.63161000	-0.61366400
C	-5.06619700	-4.91994500	-0.68181200
C	-4.26931700	-4.32018900	-1.66521000
C	-2.90104300	-4.56246900	-1.71464000
C	-2.27904500	-5.41638700	-0.79217900
C	-3.08319900	-6.02299400	0.18014200
C	-4.45186500	-5.77804000	0.23161900
C	-0.81560900	-5.66577800	-0.84532300
C	-0.28545900	-6.94040900	-0.60994600
C	1.08750700	-7.16466600	-0.65561900
C	1.97825600	-6.12690200	-0.93902700
C	1.45403500	-4.85332000	-1.17549700
C	0.08323400	-4.62643900	-1.12845800
N	6.28233800	-4.32811900	-0.17409600
C	5.40275500	-4.87139800	-1.10587500
N	4.29338900	-5.36759500	-0.41614400
C	4.62080200	-5.39928400	1.00890400
C	5.58774400	-4.21752500	1.10632600
C	5.34918100	-4.23136900	3.56760800
C	6.37337500	-4.28679400	2.40946100
O	-7.03321400	-2.36559800	-2.31292700

O	5.56638700	-4.90825100	-2.31323500
C	3.46763300	-6.38847400	-1.04169400
H	8.14750900	-3.42952300	0.05923100
H	7.61200900	-3.70036000	-1.59956500
H	-7.43606600	-0.05754400	-2.09406500
H	-8.23752800	0.48309700	-0.61654600
H	-5.34519100	-2.68308900	1.11662700
C	-6.33469100	-0.41622700	2.06406200
H	-7.99302100	-3.41712000	2.44780500
H	-7.04335900	-5.34031300	0.05896900
H	-7.01109900	-4.74245900	-1.59974800
H	-4.73145200	-3.66801700	-2.39882200
H	-2.31018800	-4.10655600	-2.50237200
H	-2.63045300	-6.67451700	0.91970700
H	-5.05126100	-6.26019200	0.99909400
H	-0.95458300	-7.77055800	-0.40982200
H	1.46971600	-8.16520400	-0.47534500
H	2.13102300	-4.03342800	-1.38286400
H	-0.29498700	-3.62414300	-1.29317500
H	5.18579500	-6.32570800	1.21575300
H	4.99585200	-3.28722200	1.11696400
H	5.86362200	-4.35791800	4.52394900
H	6.95731700	-5.21367000	2.44716300
H	7.07690300	-3.45378900	2.49925200
H	3.76820000	-6.41229500	-2.09347500
H	3.70064500	-7.37532800	-0.61718900
C	-6.68577700	-1.01834100	3.44324200

	C	-6.34023600	-2.51596700	3.56745300
	C	3.52796800	-5.27805600	2.06411700
	C	4.22324300	-5.28184100	3.44328500
	H	0.34680200	5.86059300	3.58491400
	H	0.84189400	7.25674400	4.52387100
	H	3.87935600	5.49180800	1.99973400
	H	2.29144300	4.74183900	1.91155600
	H	3.11538800	7.16231100	3.62049600
	H	2.68828900	5.57555300	4.23121400
	H	-8.07200900	-1.32941500	1.21697900
	H	-6.53022700	-4.40130000	2.49996200
	H	4.90223200	-3.23057900	3.58518200
	H	-6.69638200	0.61304800	1.99999200
	H	-5.25204200	-0.38711500	1.91206500
	H	-7.75915900	-0.88238200	3.62015900
	H	-6.17264200	-0.45920000	4.23212200
	H	-6.70623800	-2.89800000	4.52367700
	H	-5.24954600	-2.63046000	3.58661700
	H	2.81587200	-6.10671500	1.99945200
	H	2.96049500	-4.35537900	1.91102100
	H	4.64534800	-6.27908500	3.61911500
	H	3.48334800	-5.11590500	4.23081700
Conf. 5	C	-4.30832500	-5.93564000	-3.12897300
	C	-3.64378200	-5.56282800	-1.78444100
	C	-4.75525000	-5.45409300	-0.74761600
	C	-5.75581000	-4.36959700	-1.15431400
	C	-6.50722400	-4.79073200	-2.41057800

H	-3.10919900	-4.61093000	-1.86364900
H	-2.90843100	-6.32153000	-1.50598000
H	-5.28840900	-6.42215700	-0.72498200
N	-4.46669100	-5.05458000	0.62915900
N	-6.48072200	-4.18648900	0.09986600
H	-5.19041700	-3.45147700	-1.38543200
C	-5.46091000	-4.99057700	-3.52958300
C	-5.61391600	-4.45400400	1.15519600
O	-5.81801200	-4.21037500	2.33124800
C	-3.61955000	-5.84736800	1.50653100
C	-7.53133300	-3.18137400	0.27198900
C	-7.05684100	-1.74061400	0.18690700
C	-7.18582700	-0.99872400	-0.99009800
C	-6.47146900	-1.12155800	1.29835200
C	-6.03921200	0.19726000	1.23060500
C	-6.16816800	0.94567300	0.05090700
C	-6.74495100	0.32036600	-1.06185500
C	-5.72146500	2.36073400	-0.00391400
C	-5.00463500	2.86057400	-1.10198100
C	-6.01037400	3.24181000	1.04579700
C	-5.59634600	4.56951700	0.99766000
C	-4.87454400	5.06200600	-0.09171200
C	-4.58525700	4.18559300	-1.14458800
C	-2.13490600	-5.61847100	1.29439900
C	-1.23198100	-6.67981600	1.38041600
C	-1.63078900	-4.34053700	1.03829000
C	-0.26915400	-4.13710000	0.85182000

C	0.64170200	-5.20192500	0.92367300
C	0.13404300	-6.47693900	1.20374600
C	2.08942500	-4.98235200	0.68232100
C	2.51963500	-4.15763000	-0.36956600
C	3.06697600	-5.59043300	1.47861500
C	4.42272000	-5.37251800	1.23804200
C	4.84289800	-4.54836400	0.19460800
C	3.86975600	-3.94808200	-0.61202900
H	-3.84548100	-6.91748100	1.39053600
H	-3.90296200	-5.56798300	2.52550300
H	-7.97428100	-3.36972800	1.25089600
H	-8.30043300	-3.36935400	-0.48098300
H	-7.65661700	-1.44843600	-1.85915700
H	-6.34676600	-1.68914700	2.21338700
H	-5.57844600	0.65153400	2.10074600
H	-6.88094200	0.88183500	-1.97950500
H	-4.75722300	2.19625100	-1.92261100
H	-6.58780300	2.89042300	1.89349400
H	-5.85416600	5.23653000	1.81463000
C	-4.40765800	6.50669100	-0.13006800
H	-4.02512800	4.55063600	-1.99881300
H	-1.60116800	-7.68317300	1.57083800
H	-2.31699200	-3.50379900	0.97709900
H	0.09921400	-3.13449500	0.66484300
H	0.81003200	-7.32387900	1.24351400
H	1.78326900	-3.70216500	-1.02222800
H	2.76509500	-6.21670400	2.31086700

H	5.15998700	-5.84773700	1.87826400
C	6.31179300	-4.31052900	-0.09903100
H	4.18388400	-3.33240400	-1.44765900
N	6.68784200	-2.89890400	-0.07656000
C	6.99459000	-2.24804000	-1.26533300
N	7.71355300	-1.09507500	-0.95430000
C	7.60585500	-0.86096900	0.48459000
C	7.46226300	-2.29393700	1.00229800
C	8.71816900	-0.14012500	1.23450400
C	7.78817500	-0.01225900	-1.93044000
C	6.68462300	1.02767100	-1.82003400
C	5.33652800	0.65245000	-1.79834200
C	4.33194300	1.60545900	-1.69384900
C	4.62750900	2.97678000	-1.63021900
C	5.97834700	3.34756200	-1.67345300
C	6.98511000	2.38932500	-1.75494000
C	3.54721600	3.98870600	-1.51573600
C	2.28492800	3.77487400	-2.09509300
C	1.26681600	4.71050500	-1.97167900
C	1.47025900	5.90654200	-1.27605700
C	2.72585700	6.13227800	-0.71202600
C	3.74454100	5.18941100	-0.82129800
N	-2.98064400	6.69485000	0.12424200
C	-2.07576200	6.66446600	-0.93082500
N	-0.79670800	6.46809200	-0.39864000
C	-0.87274300	6.65737000	1.04862300
C	-2.31040700	6.21439700	1.32865500

C	-1.79968100	6.07741900	3.74181100
C	-2.75252200	6.70675400	2.70079300
O	6.67963500	-2.62111000	-2.38298100
O	-2.34437100	6.78813300	-2.11296900
C	0.35740300	6.92863300	-1.16606600
H	-4.96765700	7.09410300	0.60245900
H	-4.59195700	6.93966300	-1.11456900
H	6.54627300	-4.68878500	-1.09830100
H	6.93311300	-4.85582400	0.61891300
H	6.66295500	-0.32819000	0.68891800
C	6.93054300	-2.28116200	2.42971300
H	9.67056700	-0.66113600	1.08646800
H	8.76178800	0.47753000	-1.84226300
H	7.75247100	-0.49728700	-2.90907700
H	5.07069500	-0.39611200	-1.86526400
H	3.30024000	1.27763000	-1.64340900
H	6.24809200	4.39726600	-1.66080500
H	8.02224800	2.71000200	-1.78296500
H	2.10659600	2.87453100	-2.67132600
H	0.30200400	4.52094300	-2.42995200
H	2.91091700	7.05377800	-0.16821800
H	4.69582900	5.38083500	-0.33841400
H	-0.79979900	7.73990600	1.26149600
H	-2.33681300	5.11254100	1.33964000
H	-2.03023700	6.46460800	4.73849100
H	-2.70779500	7.80082400	2.73894100
H	-3.78306400	6.41351700	2.91897500

	H	-0.02192200	7.17318800	-2.16113600
	H	0.74912800	7.85490000	-0.72309200
	C	7.96071400	-1.51506700	3.29089800
	C	8.32266800	-0.12280500	2.72926500
	C	0.08988700	5.93521200	1.98426900
	C	-0.30690600	6.31277600	3.42942300
	H	-4.69563000	-6.95930100	-3.05532400
	H	-3.55537200	-5.94898100	-3.92202000
	H	-7.23546900	-4.03391100	-2.71667900
	H	-7.06060600	-5.71728000	-2.22173700
	H	-5.04448500	-4.01144100	-3.79504200
	H	-5.94954900	-5.37133100	-4.43104400
	H	8.46504600	-2.75761500	1.02069900
	H	8.84472200	0.88469000	0.87373000
	H	-1.99105900	4.99840100	3.77808600
	H	6.79862900	-3.29473600	2.81936300
	H	5.95095100	-1.79302800	2.45901900
	H	8.87293900	-2.12019600	3.36183400
	H	7.58378300	-1.40826300	4.31205500
	H	9.13334300	0.30946100	3.32288500
	H	7.46200400	0.54550000	2.85083200
	H	1.12680500	6.22249200	1.79517400
	H	0.02298100	4.85371100	1.82720600
	H	-0.07176400	7.37235000	3.58898500
	H	0.30688500	5.75211400	4.14022500
Conf. 6	C	-5.09635300	-5.17453700	-3.29274400
	C	-4.33518600	-5.04724600	-1.95390800

C	-5.38078400	-4.93595600	-0.85105300
C	-6.25792400	-3.70239400	-1.07444300
C	-7.10815300	-3.87803400	-2.32618400
H	-3.69168800	-4.16160600	-1.95103900
H	-3.68737100	-5.91485100	-1.80702600
H	-6.02632900	-5.83146600	-0.90806500
N	-4.98537600	-4.73916700	0.54208300
N	-6.89713000	-3.58378400	0.23368100
H	-5.59548100	-2.83540300	-1.23240400
C	-6.13971500	-4.06022100	-3.51670600
C	-6.01776800	-4.06691300	1.19906800
O	-6.12632800	-3.92704000	2.40472300
C	-4.19258800	-5.71847300	1.27115800
C	-7.80929800	-2.49183100	0.57524800
C	-7.19406500	-1.10503200	0.50041800
C	-7.43056600	-0.26680800	-0.59160800
C	-6.36300000	-0.63715300	1.52610100
C	-5.79084300	0.62782900	1.45524600
C	-6.02201000	1.46981600	0.35682900
C	-6.85279400	0.99733400	-0.66717700
C	-5.41831100	2.82435100	0.28404300
C	-4.86854900	3.30845400	-0.91285300
C	-5.39499600	3.66620000	1.40325800
C	-4.85234700	4.94582500	1.32258100
C	-4.30881400	5.42563800	0.12864700
C	-4.32096800	4.58349100	-0.99021800
C	-2.70052200	-5.58312800	1.03700800

C	-1.91204900	-6.69344100	0.73330000
C	-2.07403100	-4.33791400	1.14562000
C	-0.70638500	-4.20909300	0.94475300
C	0.09074500	-5.31971300	0.62343900
C	-0.54024600	-6.56658000	0.52867900
C	1.54564300	-5.16791900	0.37407000
C	2.04486700	-4.03934300	-0.29556200
C	2.46726700	-6.13766900	0.78948900
C	3.82977100	-5.98432200	0.54330500
C	4.31817600	-4.85999000	-0.12324500
C	3.40324200	-3.88788000	-0.53783400
H	-4.51700800	-6.73671800	1.01376800
H	-4.42334400	-5.56133700	2.32854700
H	-8.15470200	-2.69612000	1.58966500
H	-8.67488400	-2.55228600	-0.08927000
H	-8.09086800	-0.59700700	-1.38788200
H	-6.16243800	-1.27829500	2.37737300
H	-5.14001300	0.96346800	2.25510300
H	-7.07258300	1.63730900	-1.51431200
H	-4.85704600	2.67020400	-1.78940100
H	-5.83599000	3.32903800	2.33461200
H	-4.87461400	5.58773000	2.19814200
C	-3.72382400	6.82413600	0.03461800
H	-3.89129300	4.93610300	-1.92121700
H	-2.37630100	-7.67030600	0.63674400
H	-2.66910700	-3.46451500	1.38779900
H	-0.24305700	-3.23544300	1.05709100

H	0.04022600	-7.44085500	0.25643400
H	1.35436800	-3.28486900	-0.65493400
H	2.11939000	-7.00724800	1.33572700
H	4.52171900	-6.74855200	0.88459000
C	5.79390000	-4.70981400	-0.44215800
H	3.76432900	-3.00951400	-1.06035800
N	6.31784100	-3.38011900	-0.17070200
C	6.98147600	-2.67643500	-1.17363200
N	7.74551700	-1.68004800	-0.56724200
C	7.32823600	-1.56852200	0.82898500
C	6.89540300	-3.00710700	1.11724200
C	8.31647300	-1.09354800	1.88611000
C	8.14939400	-0.51566300	-1.35870400
C	7.09184800	0.57042000	-1.45339200
C	5.93370300	0.37378700	-2.21694200
C	4.94333300	1.34704200	-2.27026100
C	5.07105300	2.55580600	-1.56755200
C	6.23939600	2.75471100	-0.82075100
C	7.23024500	1.77806300	-0.76653300
C	4.00166800	3.58446400	-1.60445200
C	4.30745800	4.95074500	-1.64721900
C	3.29946200	5.91089700	-1.64977800
C	1.95348800	5.54341600	-1.61394400
C	1.64194700	4.18097600	-1.58421400
C	2.64598300	3.22070700	-1.57911900
N	-2.26376300	6.88966800	0.08329900
C	-1.52195400	6.64702200	-1.06607400

N	-0.20572900	6.36444100	-0.67952700
C	-0.06068100	6.74198500	0.72622700
C	-1.47759700	6.48268300	1.24385300
C	-0.64588900	6.59403800	3.56978700
C	-1.68301200	7.17734100	2.58408400
O	6.90057100	-2.89200500	-2.37034700
O	-1.93825600	6.67771800	-2.21046800
C	0.86251200	6.59559200	-1.64235300
H	-4.11652100	7.44717300	0.84201600
H	-4.01327300	7.28609100	-0.91042400
H	5.95853300	-4.88795600	-1.50892100
H	6.37378400	-5.46109400	0.11014200
H	6.43462400	-0.92580900	0.88730700
C	6.06364900	-3.06017100	2.39282000
H	9.20916800	-1.72880200	1.87681600
H	9.06611200	-0.10761900	-0.92585000
H	8.39176300	-0.89865200	-2.35129800
H	5.81913400	-0.54824300	-2.77606800
H	4.06801000	1.18018300	-2.88785400
H	6.36184800	3.66995800	-0.25258800
H	8.12223800	1.95921800	-0.17425600
H	5.34360700	5.26634300	-1.69485200
H	3.56660400	6.96286700	-1.68329600
H	0.60223400	3.87622700	-1.54422200
H	2.37704800	2.17164300	-1.52681900
H	0.12731700	7.83040100	0.77545000
H	-1.59549300	5.39772600	1.40072500

H	-0.70509500	7.12160400	4.52611000
H	-1.54855500	8.25851200	2.46855100
H	-2.69317400	7.01396900	2.97061900
H	0.38004600	6.61299100	-2.62357200
H	1.30864100	7.58843500	-1.48293400
C	6.96094100	-2.54315700	3.54062600
C	7.59837100	-1.16697900	3.25272500
C	0.96346600	6.05466700	1.62116000
C	0.80210000	6.64822900	3.03868300
H	-5.60262900	-6.14746200	-3.31543500
H	-4.38482600	-5.17976400	-4.12329600
H	-7.74990400	-3.00983400	-2.50113500
H	-7.76157500	-4.75054000	-2.21593800
H	-5.61785200	-3.11113800	-3.68786900
H	-6.70721900	-4.26805600	-4.42842400
H	7.80288800	-3.61570100	1.28293800
H	8.64422100	-0.06756600	1.69577200
H	-0.91196800	5.55043200	3.77617600
H	5.72464200	-4.07666100	2.60983500
H	5.16937100	-2.43822800	2.28415900
H	7.75766400	-3.27607300	3.71716300
H	6.38251800	-2.48987600	4.46735500
H	8.29850100	-0.91407200	4.05418700
H	6.81515500	-0.39978100	3.27403500
H	1.98234200	6.21535900	1.26103200
H	0.79137000	4.97344800	1.62649200
H	1.13602500	7.69297200	3.01935700

	H	1.46537300	6.12753200	3.73530300
Conf. 7	C	-5.40445200	-5.74857100	-3.03710100
	C	-4.52950900	-5.40514100	-1.81077300
	C	-5.47451200	-5.13060400	-0.64771700
	C	-6.39761000	-3.95427400	-0.97424600
	C	-7.34843500	-4.32857500	-2.10453000
	H	-3.90856200	-4.52618500	-2.00994900
	H	-3.85098800	-6.23285000	-1.58831600
	H	-6.10273700	-6.02930200	-0.50871900
	N	-4.96445800	-4.71927800	0.65983200
	N	-6.92900300	-3.65425000	0.35265500
	H	-5.77426200	-3.11169700	-1.31747500
	C	-6.48594300	-4.68853400	-3.33513100
	C	-5.96688500	-3.98667300	1.30154100
	O	-5.99406600	-3.69556200	2.48386900
	C	-4.08583600	-5.55864700	1.45552500
	C	-7.83739900	-2.53646200	0.61618000
	C	-7.21970300	-1.15707000	0.46117000
	C	-7.36159700	-0.42105200	-0.71825700
	C	-6.48799000	-0.58930000	1.51140600
	C	-5.92942400	0.67671600	1.38497800
	C	-6.07329400	1.42039700	0.20434100
	C	-6.79389200	0.84390700	-0.84969600
	C	-5.49493800	2.78320100	0.08891400
	C	-4.81530100	3.19449400	-1.06767200
	C	-5.62179800	3.70382400	1.13697100
	C	-5.09331300	4.98676600	1.02803500

C	-4.41406700	5.39227500	-0.12281700
C	-4.28063600	4.47382300	-1.17152500
C	-2.61011200	-5.41137700	1.12829900
C	-1.71662300	-6.43713900	1.44798600
C	-2.09522500	-4.24936800	0.55237800
C	-0.73620500	-4.12522700	0.28749000
C	0.16907600	-5.14739900	0.60869800
C	-0.35407300	-6.30544900	1.20379200
C	1.61842800	-5.00562800	0.31985700
C	2.23836300	-3.74827100	0.32147500
C	2.42195500	-6.11769000	0.02270900
C	3.77674200	-5.97462600	-0.25318800
C	4.38232500	-4.71477600	-0.26398600
C	3.58921700	-3.60440000	0.02393500
H	-4.37926800	-6.61403900	1.35701000
H	-4.26170900	-5.27321700	2.49711900
H	-8.19444500	-2.66867800	1.63840500
H	-8.69706800	-2.64144800	-0.04982400
H	-7.94257000	-0.82910000	-1.54003300
H	-6.34905700	-1.15776700	2.42351000
H	-5.35752200	1.09060000	2.20799000
H	-6.94168300	1.40514400	-1.76567400
H	-4.69007800	2.49623200	-1.88789400
H	-6.16562800	3.42202600	2.03162400
H	-5.23084800	5.68881200	1.84492000
C	-3.83721900	6.79234800	-0.23965900
H	-3.74995300	4.77069200	-2.06958600

H	-2.08820500	-7.34966800	1.90507200
H	-2.77214800	-3.44347800	0.29600700
H	-0.37763400	-3.22583600	-0.19914600
H	0.31335000	-7.10873300	1.49377600
H	1.66127900	-2.86659700	0.57466200
H	1.97830100	-7.10582500	-0.01609400
H	4.36763400	-6.85686200	-0.48221400
C	5.84843200	-4.58222500	-0.64462200
H	4.03878200	-2.61910600	0.01901500
N	6.44966700	-3.30871200	-0.29582700
C	7.05479900	-2.53771600	-1.28730800
N	7.88053300	-1.60453700	-0.66233800
C	7.58001600	-1.60992700	0.76784300
C	7.14883400	-3.06510400	0.96406900
C	8.66333500	-1.24017100	1.77273700
C	8.23745300	-0.38163500	-1.38559100
C	7.15648900	0.68647900	-1.39231500
C	5.99519000	0.51743600	-2.15826700
C	4.99464100	1.48094400	-2.15351900
C	5.11347400	2.65262900	-1.38952900
C	6.28023200	2.82130800	-0.63283000
C	7.28217300	1.85376400	-0.63609300
C	4.03914500	3.67678600	-1.39278300
C	4.33811900	5.04527200	-1.38877000
C	3.32509300	6.00003900	-1.39680000
C	1.98068000	5.62474500	-1.41341200
C	1.67619600	4.26062100	-1.41550700

C	2.68538400	3.30579600	-1.40432900
N	-2.38946000	6.88046800	-0.05792800
C	-1.54041700	6.65602900	-1.13443100
N	-0.25842000	6.40310500	-0.62852200
C	-0.25288400	6.77485300	0.78573500
C	-1.70496500	6.48343200	1.16887100
C	-1.09268200	6.59277300	3.56165000
C	-2.04728300	7.16334300	2.48871300
O	6.88861700	-2.65966700	-2.48786700
O	-1.85056400	6.67822500	-2.31207800
C	0.88724500	6.67241800	-1.48616200
H	-4.31242000	7.45087000	0.49195000
H	-4.03773600	7.19918800	-1.23196500
H	5.94698500	-4.67803000	-1.73029700
H	6.42263800	-5.40330200	-0.19352700
H	6.70572800	-0.96520600	0.95702500
C	6.43971600	-3.23351200	2.30182000
H	9.54123500	-1.87922700	1.62744400
H	9.15562800	0.01709200	-0.94765100
H	8.46509900	-0.69225600	-2.40658100
H	5.88648200	-0.37433800	-2.76472800
H	4.11944000	1.33875900	-2.77729000
H	6.39535100	3.70603900	-0.01667900
H	8.17488300	2.01212000	-0.03822200
H	5.37340700	5.36705900	-1.40280200
H	3.58722100	7.05384200	-1.40044000
H	0.63742600	3.95047300	-1.41297600

H	2.42133900	2.25439400	-1.38456700
H	-0.09345200	7.86648100	0.85711600
H	-1.81488200	5.39539200	1.30860100
H	-1.25127100	7.11066100	4.51201700
H	-1.92529400	8.24813800	2.39366300
H	-3.08466300	6.97556500	2.77987000
H	0.49106800	6.72553100	-2.50430800
H	1.31049300	7.66013100	-1.25147000
C	7.44599600	-2.82944200	3.40316600
C	8.06780900	-1.43360300	3.18547200
C	0.69958700	6.10242900	1.76700500
C	0.39677900	6.68061200	3.16776200
H	-5.89072900	-6.71591600	-2.86087900
H	-4.77033700	-5.88003000	-3.91838500
H	-8.02211000	-3.50339300	-2.35376700
H	-7.97169600	-5.17729200	-1.80193600
H	-5.99928300	-3.77516400	-3.69782300
H	-7.12802700	-5.03852400	-4.14856500
H	8.05758900	-3.69348300	0.98689500
H	8.98966700	-0.20370000	1.64704700
H	-1.35533600	5.54242300	3.73594600
H	6.11007900	-4.26504900	2.45348200
H	5.54681300	-2.60172500	2.33991600
H	8.24838200	-3.57674200	3.43538700
H	6.95671500	-2.86239700	4.38074700
H	8.83849200	-1.25526500	3.94091900
H	7.29698800	-0.67020800	3.34532700

	H	1.74359600	6.28681800	1.50214400
	H	0.55032800	5.01796900	1.75082100
	H	0.70983100	7.73181500	3.18714400
	H	1.00322200	6.16659500	3.91898800
Conf. 8	C	-6.24782900	-5.51592000	-2.92417500
	C	-5.12682000	-5.34628500	-1.87429900
	C	-5.80472100	-5.07822600	-0.53700100
	C	-6.64364100	-3.80025800	-0.61125900
	C	-7.82510200	-3.99646000	-1.55255900
	H	-4.46904700	-4.51255100	-2.13948700
	H	-4.50665400	-6.24615400	-1.83593800
	H	-6.48518300	-5.92433200	-0.33161700
	N	-5.01714700	-4.81675400	0.66682600
	N	-6.87420800	-3.54570400	0.80864400
	H	-6.01201200	-2.99640700	-1.02446400
	C	-7.25176000	-4.34308200	-2.94558400
	C	-5.79027000	-4.03704200	1.52952400
	O	-5.55359100	-3.82509500	2.70533300
	C	-4.09021100	-5.78223800	1.22983100
	C	-7.59845600	-2.37959600	1.30599200
	C	-7.00082800	-1.03606800	0.91894600
	C	-7.75365100	-0.08628800	0.22626300
	C	-5.68574500	-0.70847000	1.27336800
	C	-5.14480400	0.52455300	0.93165600
	C	-5.90202100	1.48499700	0.24378000
	C	-7.21906500	1.15834500	-0.09953600
	C	-5.32213600	2.81086500	-0.08869200

C	-6.02568400	3.99309500	0.17027600
C	-4.04360900	2.91915100	-0.65609100
C	-3.48999300	4.15990900	-0.94897000
C	-4.18580200	5.34041100	-0.66589900
C	-5.45956600	5.23523900	-0.10417200
C	-2.64384000	-5.61538500	0.79227200
C	-1.71371800	-6.61601100	1.09040600
C	-2.18577300	-4.46482600	0.15216700
C	-0.84267600	-4.31802100	-0.17994600
C	0.10042200	-5.30593700	0.13411100
C	-0.36882900	-6.46253600	0.77686100
C	1.53909200	-5.12357500	-0.18307300
C	2.12263100	-3.84784100	-0.17845200
C	2.37200800	-6.21115000	-0.48492800
C	3.72525800	-6.02948500	-0.75117500
C	4.29732800	-4.75493500	-0.74506800
C	3.47063000	-3.66607300	-0.46336200
H	-4.43059700	-6.80104200	0.99786800
H	-4.14664000	-5.66236900	2.31599200
H	-7.61152000	-2.48190800	2.39346700
H	-8.63273400	-2.43740000	0.95730400
H	-8.77439900	-0.31870700	-0.06225500
H	-5.09563300	-1.41563800	1.84502700
H	-4.13201500	0.76497000	1.23561000
H	-7.82120300	1.87430000	-0.64809600
H	-7.00868500	3.94036200	0.62504300
H	-3.48949800	2.01757500	-0.89293100

H	-2.51265400	4.21307600	-1.41577900
C	-3.58816500	6.70312600	-0.97535600
H	-6.01684300	6.13741600	0.12950000
H	-2.04105400	-7.51994200	1.59609000
H	-2.89062300	-3.67827800	-0.08703000
H	-0.52615100	-3.42388200	-0.70442500
H	0.33125800	-7.24021700	1.05980600
H	1.51885300	-2.98475600	0.07678000
H	1.95566700	-7.21111300	-0.52618200
H	4.34275700	-6.89421600	-0.97616700
C	5.76579900	-4.56991100	-1.08768400
H	3.89598000	-2.66981700	-0.45006300
N	6.36631800	-3.38500200	-0.50181400
C	7.09604800	-2.51010100	-1.30625300
N	7.89098800	-1.72337100	-0.47439000
C	7.45351700	-1.92275900	0.90539900
C	6.94681900	-3.36523200	0.83894200
C	8.44982900	-1.76781000	2.04756800
C	8.36864100	-0.42863400	-0.96760600
C	7.32601400	0.67674400	-0.95433200
C	6.31083900	0.70247400	-1.91961800
C	5.34281400	1.69818100	-1.90536900
C	5.34910500	2.70898100	-0.93113700
C	6.36970000	2.68313700	0.02811800
C	7.34017300	1.68373700	0.01372000
C	4.31180400	3.77083700	-0.93917100
C	4.62745400	5.10426500	-0.64986300

C	3.65325800	6.09855800	-0.70099100
C	2.33407500	5.79757300	-1.04229000
C	2.00963100	4.46504900	-1.31288500
C	2.97842200	3.47179300	-1.26216800
N	-2.22474600	6.90228500	-0.50284000
C	-1.14986000	6.71037300	-1.36274100
N	0.00843700	6.59562900	-0.58584500
C	-0.31064100	7.03522300	0.77071200
C	-1.78700000	6.64583800	0.86662100
C	-1.70264000	6.93552700	3.31717100
C	-2.44817300	7.36736100	2.03361900
O	7.04493900	-2.44585900	-2.52168900
O	-1.20147200	6.64842300	-2.57752200
C	1.29633900	6.89203600	-1.19980700
H	-4.23000700	7.48253500	-0.55430400
H	-3.54725400	6.86126100	-2.05628700
H	5.88127200	-4.46084100	-2.17043700
H	6.32870100	-5.46636000	-0.79127800
H	6.59196000	-1.26643300	1.11175800
C	6.10379500	-3.68900900	2.06603400
H	9.30931700	-2.42720400	1.88420800
H	9.23431300	-0.13765200	-0.36789100
H	8.71602500	-0.60316000	-1.98704700
H	6.29043500	-0.06523600	-2.68431400
H	4.58646800	1.71250400	-2.68175800
H	6.39318700	3.43937900	0.80483000
H	8.12069800	1.69062900	0.76881000

H	5.65092200	5.37307700	-0.41346300
H	3.93113800	7.12690400	-0.48983900
H	0.98711400	4.21051600	-1.56729900
H	2.69765700	2.44460700	-1.46579400
H	-0.24335300	8.13816000	0.80358500
H	-1.84524500	5.56366700	1.06583200
H	-2.08932600	7.49200900	4.17576000
H	-2.38519000	8.45176200	1.88966300
H	-3.50731700	7.10700600	2.11516100
H	1.09629300	7.04603600	-2.26435600
H	1.68885600	7.83659200	-0.79980900
C	7.01393900	-3.50906800	3.30189500
C	7.71326000	-2.13410100	3.35576400
C	0.45687000	6.48954100	1.96797900
C	-0.17136900	7.11601100	3.23389400
H	-6.78886200	-6.44596100	-2.71098800
H	-5.80852700	-5.63744400	-3.91831900
H	-8.43710200	-3.09218400	-1.61841000
H	-8.46898800	-4.80216200	-1.18250600
H	-6.75165700	-3.45312700	-3.34596000
H	-8.06822500	-4.57329200	-3.63601000
H	7.82354500	-4.03818300	0.84921500
H	8.83170700	-0.74465500	2.11171000
H	-1.92737000	5.87955900	3.50859600
H	5.71772700	-4.71114100	2.02858100
H	5.23842800	-3.02088600	2.11811000
H	7.77660600	-4.29738500	3.29179000

	H	6.43092300	-3.65764700	4.21520300
	H	8.41463700	-2.11354300	4.19486900
	H	6.96409200	-1.36008800	3.56103700
	H	1.51953300	6.74077700	1.91144000
	H	0.38491000	5.39728700	1.99344200
	H	0.06349500	8.18748800	3.24528000
	H	0.29389200	6.69094300	4.12783200

4 (ACN)

Conf. 1	C	0.08080400	7.31123400	3.44075500
	C	-0.29262000	7.97408300	2.09465100
	C	0.34597700	7.13661300	0.99505000
	C	1.86533600	7.11344300	1.16028000
	C	2.24440600	6.33770100	2.41433600
	H	0.08056200	9.00303800	2.04952700
	H	-1.38023300	8.01259100	1.98877700
	H	-0.02698200	6.10499200	1.09574300
	N	0.23075500	7.54050800	-0.40720600
	N	2.28095100	6.64655800	-0.16596600
	H	2.21746600	8.15139000	1.28635200
	C	1.59604300	7.07043100	3.61149800
	C	1.34380600	7.08314700	-1.09321800
	O	1.48472400	7.06776500	-2.31217000
	C	-1.04570700	7.73092700	-1.08658900
	C	3.69222300	6.56521800	-0.55009400
	C	4.22261100	5.14644200	-0.62151200
	C	3.79607900	4.28573100	-1.63971800
	C	5.14926100	4.66375600	0.30382400

C	5.63158700	3.35923500	0.22320900
C	5.20910900	2.49261000	-0.79491800
C	4.28054500	2.98643200	-1.72712100
C	5.72678900	1.10327100	-0.88626300
C	4.92087300	0.05540600	-1.36262800
C	7.03678000	0.78910300	-0.49787900
C	7.51352700	-0.51735900	-0.56836500
C	6.70856800	-1.55527400	-1.04283300
C	5.40410700	-1.24540100	-1.44500300
C	-2.00532200	6.55538800	-0.99658900
C	-3.31596500	6.74202600	-0.55186400
C	-1.61605100	5.26524100	-1.37499000
C	-2.50595200	4.19990600	-1.30126300
C	-3.82681600	4.38303600	-0.85853800
C	-4.21390200	5.67944300	-0.49067900
C	-4.77763200	3.24402600	-0.78396000
C	-4.71907900	2.18800900	-1.70959800
C	-5.76661000	3.18507500	0.20821400
C	-6.66008400	2.11777500	0.27030300
C	-6.59248400	1.06871900	-0.64784700
C	-5.60683700	1.12159900	-1.64039000
H	-0.79979400	7.93737500	-2.13086900
H	-1.52944900	8.62393200	-0.68371500
H	4.28216600	7.14996400	0.16163300
H	3.79004500	7.03813300	-1.53028400
H	3.08748600	4.64716700	-2.37743500
H	5.49767000	5.30952100	1.10340200

H	6.32971600	3.00854300	0.97449100
H	3.95264100	2.35526800	-2.54497800
H	3.89710400	0.25573700	-1.65654800
H	7.69828500	1.57372200	-0.14972900
H	8.53167200	-0.72897200	-0.25758700
C	7.24802700	-2.97370400	-1.13078500
H	4.75744600	-2.02882600	-1.82449700
H	-3.64565100	7.73364800	-0.25876600
H	-0.60623500	5.08971000	-1.72898000
H	-2.16432700	3.20895300	-1.57670200
H	-5.23113800	5.86686100	-0.16725500
H	-3.98659100	2.21422300	-2.50798600
H	-5.82933800	3.96936300	0.95359700
H	-7.41417900	2.10132700	1.05059900
C	-7.55904400	-0.09871000	-0.59453300
H	-5.54749400	0.32265700	-2.37173900
N	-6.93235100	-1.36189800	-0.19934100
C	-6.83400000	-2.39697300	-1.11984800
N	-6.67633300	-3.58534600	-0.42532900
C	-6.39541500	-3.27404500	0.97756100
C	-7.13863900	-1.94710200	1.12858200
C	-6.80806400	-4.23992900	2.07997000
C	-6.19121500	-4.78735000	-1.09343900
C	-4.69231500	-5.01950600	-0.99230300
C	-3.77408900	-4.03092100	-1.36484000
C	-2.40539100	-4.25814600	-1.27904300
C	-1.89791000	-5.48862700	-0.82919100

C	-2.82249300	-6.47820100	-0.46624900
C	-4.19286800	-6.24287900	-0.54017200
C	-0.43519300	-5.73246600	-0.74273600
C	0.10733600	-6.55209900	0.25703600
C	1.47881800	-6.78729200	0.32868300
C	2.35686500	-6.20748800	-0.58824300
C	1.82143100	-5.38617100	-1.58744000
C	0.45374300	-5.15454100	-1.66536400
N	6.45036200	-3.97878000	-0.43630400
C	5.48038500	-4.70676500	-1.10594200
N	4.63851800	-5.28522200	-0.16490100
C	5.26602600	-5.15535200	1.15351100
C	6.06088200	-3.86268700	0.97010800
C	6.37671000	-3.70874300	3.41117700
C	7.12035800	-3.73590500	2.05593800
O	-6.88279600	-2.27523200	-2.33999800
O	5.37978400	-4.82712000	-2.32306600
C	3.85029600	-6.46434000	-0.53102400
H	8.26617300	-2.99814800	-0.73522000
H	7.29510600	-3.29674900	-2.17316400
H	-7.99901700	-0.25049600	-1.58304600
H	-8.37360800	0.12057300	0.10200200
H	-5.31666900	-3.07856600	1.08625300
C	-6.66781900	-1.22255900	2.38204200
H	-7.88454600	-4.43563600	2.02583200
H	-6.71903500	-5.65376200	-0.68766600
H	-6.48746700	-4.68748400	-2.14022000

H	-4.13097000	-3.07208000	-1.72422100
H	-1.72159100	-3.46224800	-1.55006300
H	-2.47102100	-7.44885000	-0.13627500
H	-4.88318000	-7.02881300	-0.25085700
H	-0.54346500	-6.99757500	1.00050600
H	1.86689500	-7.42762000	1.11419000
H	2.48596900	-4.93854900	-2.31880300
H	0.06823500	-4.53793300	-2.46919300
H	5.98001600	-5.98712700	1.27886400
H	5.36647900	-3.01385200	1.07459800
H	7.10200700	-3.70553500	4.22943100
H	7.81339800	-4.58279300	2.00703400
H	7.70770200	-2.82138500	1.93683300
H	4.19760800	-6.79529200	-1.51283200
H	4.06543100	-7.26391900	0.18375200
C	-6.98852000	-2.14339800	3.58189800
C	-6.43470100	-3.57544400	3.42557100
C	4.41907600	-5.07413900	2.41622300
C	5.39429800	-4.88443200	3.60119400
H	-0.44405400	6.35128800	3.51122700
H	-0.28541800	7.92544800	4.26798000
H	3.32865300	6.29613500	2.54791600
H	1.88153800	5.30693500	2.34360000
H	2.09922100	8.03589100	3.74110400
H	1.77290500	6.50367600	4.52972200
H	-8.21417700	-2.16255900	1.24733700
H	-6.29306800	-5.19971900	1.98454300

	H	5.82341800	-2.76498200	3.48350700
	H	-7.17481900	-0.26177500	2.50498300
	H	-5.59301700	-1.02188300	2.31929900
	H	-8.07743300	-2.19185800	3.70045400
	H	-6.59652700	-1.70031200	4.50149600
	H	-6.78953400	-4.19537300	4.25349300
	H	-5.34162100	-3.54705600	3.50563000
	H	3.82921100	-5.98274900	2.56364900
	H	3.71938400	-4.23448900	2.34699800
	H	5.96583400	-5.81106800	3.73103400
	H	4.82752300	-4.73854200	4.52482000
Conf. 2	C	7.17369900	2.90211600	3.16492600
	C	7.54377800	3.25973300	1.70665400
	C	6.91437200	2.19825300	0.81463600
	C	7.46147700	0.81534900	1.16793500
	C	6.97953000	0.39951000	2.55055800
	H	8.63069800	3.27053100	1.57073400
	H	7.17116400	4.25848300	1.46341600
	H	5.82919600	2.19853000	1.00458400
	N	7.13844900	2.21709800	-0.63157500
	N	7.07274800	0.05225900	-0.02305800
	H	8.56257500	0.87608200	1.19772100
	C	7.51876800	1.44808200	3.55049900
	C	7.07602700	0.91826100	-1.10878800
	O	7.04105300	0.58386600	-2.28924600
	C	6.81480300	3.35240600	-1.48559000
	C	7.48570400	-1.34259400	-0.21813400

C	6.32503100	-2.31711500	-0.28645900
C	5.57964300	-2.44715900	-1.46387300
C	5.97259800	-3.11057500	0.80761300
C	4.90594100	-4.00301300	0.73339400
C	4.15821200	-4.14110100	-0.44513600
C	4.51934300	-3.34259100	-1.54312400
C	3.02901400	-5.10233800	-0.53383900
C	1.89871600	-4.82550800	-1.32169400
C	3.05449600	-6.32065800	0.15932600
C	1.99248100	-7.21807900	0.07465700
C	0.87308400	-6.93952800	-0.71225500
C	0.84562300	-5.72770300	-1.41397400
C	5.39071900	3.86996500	-1.36056800
C	5.14776900	5.23680900	-1.20583300
C	4.28918400	3.00893000	-1.41921300
C	2.99223000	3.49921200	-1.31525500
C	2.74222400	4.87327900	-1.16253700
C	3.84970500	5.73230500	-1.11743400
C	1.35640700	5.39614600	-1.04874100
C	0.28833600	4.78029400	-1.72415800
C	1.06583100	6.52031600	-0.26319300
C	-0.23466500	7.00981800	-0.16039600
C	-1.29142700	6.39051000	-0.82890500
C	-1.00818000	5.26638300	-1.61292000
H	7.00659900	3.01951900	-2.50864100
H	7.51291300	4.16630200	-1.27397600
H	8.15064000	-1.61431200	0.60478900

H	8.06204300	-1.40151900	-1.14503300
H	5.84268700	-1.84114300	-2.32368100
H	6.53374400	-3.03214800	1.73306000
H	4.64438000	-4.58626500	1.60869500
H	3.98129200	-3.44134000	-2.47887500
H	1.83594400	-3.88668400	-1.85952800
H	3.91934000	-6.58242600	0.75780600
H	2.04381500	-8.15458000	0.62078900
C	-0.27565600	-7.92981600	-0.81361200
H	-0.00716400	-5.49067800	-2.04042500
H	5.98382300	5.92755200	-1.16320900
H	4.44325100	1.94241100	-1.54077400
H	2.16349400	2.80118100	-1.33492900
H	3.69911400	6.80160000	-1.02548100
H	0.47774600	3.92498700	-2.36237100
H	1.85720400	7.00755100	0.29442400
H	-0.42796100	7.87889900	0.46024700
C	-2.70738800	6.92667900	-0.73775700
H	-1.81195200	4.77403100	-2.14976200
N	-3.66351900	5.96744300	-0.19182300
C	-4.79319300	5.61919600	-0.92122400
N	-5.73810200	5.10745000	-0.04442000
C	-5.08903400	4.86263400	1.24570000
C	-4.01112200	5.94545800	1.23182300
C	-5.89423900	4.93234800	2.53596600
C	-6.84126200	4.28520000	-0.53947000
C	-6.52647300	2.80176400	-0.63546300

C	-5.56286500	2.33015800	-1.53611400
C	-5.25909900	0.97602500	-1.61128300
C	-5.91013000	0.03871900	-0.79180100
C	-6.88166300	0.51409000	0.09934900
C	-7.18139300	1.87247900	0.17557800
C	-5.57476600	-1.40615000	-0.86556600
C	-6.56539300	-2.39137600	-0.74674000
C	-6.24236800	-3.74434200	-0.81126900
C	-4.92247300	-4.16126800	-0.99934000
C	-3.93025600	-3.18339300	-1.11465700
C	-4.24949600	-1.83172300	-1.04964500
N	-1.56848100	-7.41495100	-0.37016700
C	-2.40844800	-6.76167200	-1.25663400
N	-3.33632000	-6.03024600	-0.52193200
C	-3.26799600	-6.47934300	0.87190600
C	-1.79588200	-6.87814100	0.97304300
C	-1.97616700	-6.92062900	3.43349000
C	-1.56757900	-7.75394400	2.19730100
O	-4.92821300	5.74245800	-2.13452100
O	-2.34674700	-6.81557500	-2.48047700
C	-4.59410700	-5.63636200	-1.13979400
H	-0.04047500	-8.82427800	-0.23268100
H	-0.41503200	-8.24045600	-1.85111100
H	-3.06479300	7.18871000	-1.73654500
H	-2.72111400	7.83946600	-0.13157900
H	-4.59003000	3.88090800	1.20990700
C	-2.95614800	5.65322500	2.29018500

H	-6.38201200	5.90939700	2.62268000
H	-7.70456500	4.43750200	0.11192400
H	-7.09744100	4.68276200	-1.52313500
H	-5.05630000	3.03123900	-2.19029500
H	-4.52402900	0.63700500	-2.33227300
H	-7.39058400	-0.17887100	0.75955000
H	-7.93238400	2.21176500	0.88170300
H	-7.60152900	-2.09937800	-0.61845300
H	-7.03100500	-4.48447100	-0.71949200
H	-2.89719900	-3.48610300	-1.24129500
H	-3.45617100	-1.09630700	-1.12146000
H	-3.88196800	-7.39130900	0.96801600
H	-1.19636600	-5.96176800	1.09471900
H	-1.90673500	-7.53778100	4.33346600
H	-2.16989000	-8.66602400	2.12503800
H	-0.52040600	-8.05645200	2.28371800
H	-4.50693500	-5.88762100	-2.20063500
H	-5.41500000	-6.23873200	-0.73121000
C	-3.67860000	5.64270200	3.65716600
C	-4.90344200	4.70555600	3.70088900
C	-3.64303700	-5.54570900	2.01462800
C	-3.39428100	-6.31973800	3.32976100
H	6.09714100	3.06152500	3.29756400
H	7.67529400	3.58969700	3.85140200
H	7.34675500	-0.59396500	2.82205800
H	5.88517800	0.36369400	2.57229700
H	8.60853800	1.34248500	3.60941500

	H	7.13262100	1.23555900	4.55114300
	H	-4.48283400	6.91049500	1.48445500
	H	-6.67718400	4.16983000	2.56222400
	H	-1.25139700	-6.10746700	3.55811600
	H	-2.16959600	6.41202400	2.29096800
	H	-2.48046100	4.68699700	2.09157400
	H	-4.00225000	6.66502000	3.88623900
	H	-2.97381500	5.35807500	4.44325900
	H	-5.42262300	4.82862600	4.65534600
	H	-4.55992200	3.66494400	3.66620900
	H	-4.69085400	-5.24026000	1.95329800
	H	-3.03322500	-4.63698600	1.97582100
	H	-4.13135300	-7.12849900	3.40181800
	H	-3.57389700	-5.66099200	4.18386500
Conf. 3	C	-7.44413400	0.73690900	3.29676600
	C	-8.11792200	0.40725800	1.94462300
	C	-7.18565400	0.90820400	0.84995700
	C	-6.98297700	2.41759900	0.97839600
	C	-6.19267900	2.73771400	2.23941500
	H	-9.09277600	0.90011200	1.86314900
	H	-8.28557700	-0.67043500	1.86643600
	H	-6.20963200	0.41576500	0.98451600
	N	-7.57413200	0.80323700	-0.55810200
	N	-6.44603900	2.73913800	-0.34665700
	H	-7.97369600	2.89434100	1.07183800
	C	-7.02401200	2.21637700	3.43452000
	C	-6.97876900	1.83815400	-1.26129500

O	-6.92693100	1.94437300	-2.48170100
C	-7.86017100	-0.46946100	-1.21227600
C	-6.17701900	4.11054400	-0.75550500
C	-4.72827800	4.53875200	-0.60536600
C	-3.68057200	3.66073900	-0.89723000
C	-4.40983500	5.84997500	-0.24331800
C	-3.08584600	6.27868900	-0.19099300
C	-2.03192600	5.40673500	-0.49952300
C	-2.35787700	4.08587800	-0.84563100
C	-0.61879800	5.86256600	-0.47664400
C	0.29384300	5.41607800	-1.44701200
C	-0.15065800	6.75440200	0.49776900
C	1.17666400	7.17919800	0.50212800
C	2.08038700	6.73302600	-0.46506500
C	1.61561700	5.84424400	-1.44327600
C	-6.75188000	-1.50538900	-1.10730300
C	-7.02472400	-2.79636300	-0.64991900
C	-5.43620300	-1.20373700	-1.47849900
C	-4.42880200	-2.15610500	-1.37809400
C	-4.69735000	-3.45626300	-0.91812100
C	-6.02069600	-3.75773300	-0.56536100
C	-3.61753200	-4.47041200	-0.80539400
C	-2.52446500	-4.47287700	-1.68921600
C	-3.65090500	-5.45926700	0.18819500
C	-2.63780500	-6.40984800	0.29178000
C	-1.54998500	-6.40087200	-0.58263200
C	-1.51071300	-5.41614200	-1.57633100

H	-8.05541100	-0.23087800	-2.26034700
H	-8.78228500	-0.88021900	-0.79483200
H	-6.82612700	4.79369400	-0.19615000
H	-6.46095500	4.18487300	-1.80942400
H	-3.90415900	2.63272000	-1.15766800
H	-5.20529100	6.55091700	-0.01036900
H	-2.87226300	7.30896600	0.07019200
H	-1.56562300	3.37856800	-1.06305500
H	-0.04356500	4.74834400	-2.23152800
H	-0.82229500	7.10489800	1.27326100
H	1.51150100	7.86643600	1.27250000
C	3.52670600	7.20012400	-0.46510800
H	2.29470400	5.49939800	-2.21469000
H	-8.03775500	-3.06005500	-0.36316600
H	-5.19494500	-0.21396500	-1.84967300
H	-3.41587200	-1.87876300	-1.64524600
H	-6.27575900	-4.75675600	-0.23171700
H	-2.47841900	-3.74321400	-2.48920600
H	-4.46455400	-5.47779000	0.90375900
H	-2.69408200	-7.16247000	1.07166300
C	-0.44456800	-7.43420100	-0.48415600
H	-0.68244700	-5.39890700	-2.27676200
N	0.84792800	-6.88076400	-0.07781100
C	1.94587700	-7.01796000	-0.91929300
N	3.09814700	-6.90976400	-0.15709300
C	2.73383600	-6.40809400	1.16930200
C	1.31923300	-6.97006500	1.30738800

C	3.56877000	-6.77722200	2.38828800
C	4.38463000	-6.61913800	-0.78903700
C	4.71413600	-5.13966300	-0.89514300
C	3.96315800	-4.29063700	-1.71778800
C	4.25104500	-2.93282400	-1.79463200
C	5.30357300	-2.36954000	-1.05346000
C	6.06080800	-3.22630500	-0.24263300
C	5.76905400	-4.58607400	-0.16639900
C	5.59840800	-0.91519800	-1.11664600
C	6.91507700	-0.43582800	-1.05854900
C	7.18418900	0.92996000	-1.08822500
C	6.15118900	1.86566700	-1.18203300
C	4.83732300	1.39345700	-1.25071700
C	4.56515400	0.03021300	-1.21718500
N	4.50647100	6.16108300	-0.14953700
C	4.96866100	5.30788000	-1.13928600
N	5.51820700	4.18663900	-0.52725300
C	5.68802100	4.48564200	0.89786700
C	4.52027400	5.44455000	1.12788800
C	4.77761200	5.16278300	3.56828200
C	4.71093300	6.20873000	2.43135700
O	1.89801900	-7.20655200	-2.13067800
O	4.90554300	5.50545000	-2.34815300
C	6.45997000	3.35017100	-1.25645900
H	3.65246300	8.01558300	0.25005300
H	3.79649800	7.58362100	-1.45067700
H	-0.29413000	-7.90060200	-1.46039700

H	-0.73226100	-8.22168700	0.22039800
H	2.66694800	-5.30940500	1.12414700
C	0.57874900	-6.25763400	2.43128100
H	3.63032800	-7.86654500	2.48568600
H	5.16976800	-7.12992600	-0.22700700
H	4.34140500	-7.07231100	-1.78098100
H	3.15358100	-4.70137400	-2.31117600
H	3.66553600	-2.30486000	-2.45635600
H	6.86895900	-2.82400100	0.35734700
H	6.36788900	-5.22222100	0.47738600
H	7.74028600	-1.13632100	-1.00017200
H	8.21374800	1.27026700	-1.03816000
H	4.01966600	2.10244500	-1.31022000
H	3.53395200	-0.30317200	-1.24395300
H	6.62847800	5.05007200	1.01894700
H	3.59589000	4.85082600	1.21280900
H	4.99593400	5.66299700	4.51582800
H	5.63330600	6.79820100	2.38907000
H	3.88565400	6.90219800	2.61412200
H	6.42156900	3.68157600	-2.29782600
H	7.48043900	3.53252700	-0.89653600
C	1.37658300	-6.51125400	3.73103400
C	2.87345200	-6.15182200	3.61935600
C	5.67671500	3.37409400	1.93810100
C	5.81717200	4.04993300	3.32085900
H	-6.55719200	0.10160800	3.40481700
H	-8.11628500	0.47201100	4.11755700

	H	-6.02329800	3.81293900	2.34293600
	H	-5.21220900	2.25125700	2.20418700
	H	-7.92325000	2.83674400	3.52783900
	H	-6.45946500	2.34872200	4.36156200
	H	1.39408200	-8.03764600	1.57598800
	H	4.58980500	-6.39535300	2.30658100
	H	3.78640500	4.70738800	3.67887400
	H	-0.44070500	-6.63549200	2.54246900
	H	0.51072800	-5.18601300	2.21615100
	H	1.28217800	-7.57156000	3.99396000
	H	0.92778400	-5.94800900	4.55388500
	H	3.39105400	-6.46113300	4.53155000
	H	2.97669600	-5.06195400	3.55868100
	H	6.49869800	2.67118500	1.78039300
	H	4.74261700	2.80641200	1.87497900
	H	6.82433200	4.47626700	3.39938800
	H	5.73790100	3.29610900	4.10901200
Conf. 4	C	-5.84923500	-3.78906200	3.55807600
	C	-6.19431000	-4.70997100	2.36522400
	C	-5.86027200	-3.94028300	1.09452300
	C	-6.68442500	-2.65540800	1.02099400
	C	-6.25351100	-1.68605300	2.11331300
	H	-7.25634700	-4.97814800	2.37335600
	H	-5.62023400	-5.63774900	2.43597400
	H	-4.79395300	-3.66553700	1.13288000
	N	-6.14984900	-4.52331500	-0.21739300
	N	-6.52923100	-2.31113200	-0.39523700

H	-7.74322400	-2.91113800	1.19657400
C	-6.49929300	-2.39232700	3.46632300
C	-6.38838200	-3.49356000	-1.11413600
O	-6.46295200	-3.60336600	-2.33315000
C	-5.54867800	-5.77684200	-0.67269900
C	-7.23154600	-1.18283200	-0.98962900
C	-6.47953500	0.13238800	-0.89860300
C	-5.10068500	0.19452800	-1.12056700
C	-7.16141800	1.32420700	-0.64237500
C	-6.48921000	2.54349300	-0.61172000
C	-5.10649700	2.61184900	-0.83425100
C	-4.42654600	1.40996100	-1.08874500
C	-4.38188000	3.90778400	-0.79650900
C	-3.32783300	4.17401700	-1.68653600
C	-4.72396800	4.90584100	0.12624000
C	-4.03547900	6.11641900	0.16167700
C	-2.98450100	6.37473900	-0.72106900
C	-2.64371500	5.38326400	-1.65044700
C	-4.02954000	-5.76773000	-0.71830600
C	-3.27720000	-6.53990900	0.16963800
C	-3.34475500	-4.98103900	-1.65391400
C	-1.95540800	-4.96449800	-1.69129500
C	-1.19478800	-5.73574800	-0.79664200
C	-1.88457300	-6.52640800	0.13271800
C	0.28959500	-5.71383800	-0.83737500
C	0.98864700	-4.52569500	-1.10555800
C	1.04188600	-6.87399700	-0.60516100

C	2.43381300	-6.84557100	-0.64008500
C	3.12314900	-5.66122600	-0.91035700
C	2.37825000	-4.50092800	-1.14144300
H	-5.95549200	-5.96513300	-1.66770400
H	-5.89432400	-6.57951700	-0.01813000
H	-8.22087200	-1.07502000	-0.52782800
H	-7.39198700	-1.43926700	-2.04046900
H	-4.54934700	-0.72056100	-1.30414400
H	-8.23264000	1.30303100	-0.46840200
H	-7.04978600	3.45291100	-0.42760700
H	-3.35353800	1.42363700	-1.24233400
H	-3.05732100	3.43533000	-2.43238400
H	-5.51970900	4.72788200	0.84049000
H	-4.32014300	6.86761100	0.89138000
C	-2.22774200	7.69222900	-0.67551400
H	-1.84326000	5.56896200	-2.35797500
H	-3.78286200	-7.15832700	0.90435200
H	-3.90882500	-4.38805100	-2.36524100
H	-1.45347800	-4.36500000	-2.44215500
H	-1.32969400	-7.11997900	0.85023600
H	0.43908200	-3.60534900	-1.26740500
H	0.53607800	-7.81298600	-0.41085300
H	2.98916900	-7.76036300	-0.45871200
C	4.63758800	-5.65433200	-1.00662600
H	2.89396300	-3.56741700	-1.33577300
N	5.26501200	-4.48639200	-0.40492200
C	6.22456400	-3.77451300	-1.11761800

N	6.99731300	-3.06019700	-0.21505100
C	6.34410000	-3.10484800	1.09493000
C	5.63728700	-4.45743300	1.01233900
C	7.17529200	-3.02041200	2.36804800
C	7.78706600	-1.91318700	-0.66422700
C	7.02263400	-0.60025900	-0.71073600
C	6.00198400	-0.39696300	-1.64868400
C	5.29427400	0.79861800	-1.68506300
C	5.58012300	1.84073000	-0.78719100
C	6.60786300	1.63588900	0.14360900
C	7.31448600	0.43573900	0.17952400
C	4.81882300	3.11521900	-0.82566800
C	5.44608200	4.34600000	-0.58524700
C	4.72539000	5.53717300	-0.61774100
C	3.35618500	5.54309000	-0.89399700
C	2.72539300	4.31871100	-1.13425700
C	3.44166800	3.12765700	-1.10013700
N	-0.84317500	7.58182300	-0.21618200
C	0.16990900	7.27872300	-1.11261900
N	1.26214900	6.80430200	-0.39419600
C	1.03809600	7.10057100	1.02368700
C	-0.48685600	7.03087000	1.09308000
C	-0.36698900	6.92895600	3.55664900
C	-0.98795000	7.69744200	2.36736700
O	6.36260100	-3.78165200	-2.33606800
O	0.11533600	7.40620600	-2.33100600
C	2.59222600	6.85128000	-0.98479900

H	-2.74984500	8.39628900	-0.02422100
H	-2.18242900	8.13623800	-1.67135100
H	4.93648500	-5.65524100	-2.05841600
H	5.04067300	-6.56916200	-0.55446900
H	5.57645700	-2.31558900	1.13559400
C	4.57974300	-4.57000500	2.10220800
H	7.93528100	-3.80930000	2.37280900
H	8.65211800	-1.81441000	-0.00527700
H	8.15782900	-2.17013600	-1.65787300
H	5.77164100	-1.17984100	-2.36256100
H	4.52631100	0.93571600	-2.43777000
H	6.84366600	2.41140800	0.86335400
H	8.10167200	0.30561600	0.91525700
H	6.51118700	4.37667800	-0.38561600
H	5.23903500	6.47460200	-0.42896800
H	1.66005200	4.29890400	-1.33375800
H	2.92016300	2.19227400	-1.26846300
H	1.34667900	8.14371500	1.20825300
H	-0.78383500	5.97028500	1.12380200
H	-0.63495700	7.42395100	4.49456700
H	-0.68707600	8.75066800	2.38308400
H	-2.07859000	7.66585300	2.43581900
H	2.45248700	7.12085800	-2.03524400
H	3.17839100	7.65361100	-0.51964100
C	5.31268000	-4.44144000	3.45722200
C	6.20288600	-3.18490200	3.55840700
C	1.65797400	6.23360400	2.11137700

	C	1.16720200	6.79012000	3.46740800
	H	-4.75982200	-3.67397500	3.60290500
	H	-6.14869000	-4.27090800	4.49334100
	H	-6.82430400	-0.75487000	2.06867200
	H	-5.19575600	-1.42937100	1.99396600
	H	-7.58109000	-2.49013900	3.61582500
	H	-6.12980500	-1.76435400	4.28166500
	H	6.38433500	-5.25079100	1.18545000
	H	7.69566100	-2.06203000	2.44540100
	H	-0.81483400	5.92885400	3.59435300
	H	4.05382200	-5.52687300	2.05161000
	H	3.83305900	-3.77764900	1.98615100
	H	5.93411100	-5.33313900	3.60208000
	H	4.58292200	-4.43727900	4.27155500
	H	6.76847600	-3.21224200	4.49431400
	H	5.56264100	-2.29624400	3.60826300
	H	2.74991800	6.26099100	2.06996900
	H	1.35034600	5.19061700	1.98324500
	H	1.62583600	7.77336100	3.62593700
	H	1.52227100	6.14876900	4.27873600
Conf. 5	C	-4.65899700	-5.67684300	-3.17218900
	C	-3.97650000	-5.36132000	-1.82165300
	C	-5.08305600	-5.21223600	-0.78551600
	C	-6.02078700	-4.06922500	-1.17450600
	C	-6.79847100	-4.43364100	-2.43223300
	H	-3.39334800	-4.43692200	-1.88622000
	H	-3.28677300	-6.16615800	-1.55636500

H	-5.66419500	-6.15039800	-0.77705100
N	-4.77563400	-4.85604000	0.60322300
N	-6.72805100	-3.85592400	0.08929300
H	-5.40810700	-3.18076200	-1.39630900
C	-5.76398400	-4.66948400	-3.55573600
C	-5.88219600	-4.19818300	1.13308700
O	-6.07624700	-3.96592400	2.32098900
C	-3.98021000	-5.72844600	1.45735500
C	-7.72333100	-2.79515400	0.26810400
C	-7.16564100	-1.38346700	0.19391900
C	-7.17715300	-0.65969700	-1.00248400
C	-6.61773400	-0.77303100	1.32879700
C	-6.10404600	0.51780100	1.26807100
C	-6.11140900	1.24551700	0.06789500
C	-6.65440000	0.62967400	-1.06850800
C	-5.57407400	2.62943100	0.01225900
C	-4.79934200	3.06895500	-1.07345300
C	-5.83149700	3.54085900	1.04520700
C	-5.33389600	4.84034600	0.99330600
C	-4.55797300	5.27336000	-0.08473500
C	-4.29828200	4.36567100	-1.11909800
C	-2.48427100	-5.55419200	1.27694800
C	-1.63792800	-6.66280700	1.20774400
C	-1.91246700	-4.28038700	1.20071600
C	-0.54134200	-4.12249700	1.03955800
C	0.31341700	-5.23361500	0.94953600
C	-0.26269900	-6.50778800	1.04812500

C	1.77068900	-5.06002500	0.72372600
C	2.24722300	-4.03878800	-0.11437300
C	2.71588600	-5.90688300	1.31970100
C	4.07936900	-5.74156700	1.08228900
C	4.54534000	-4.72823500	0.24198900
C	3.60652800	-3.87746700	-0.35004700
H	-4.25287900	-6.77745300	1.28251200
H	-4.26309200	-5.48824900	2.48567900
H	-8.18543900	-2.96253600	1.24157300
H	-8.49534800	-2.93392700	-0.49122400
H	-7.61544900	-1.09888100	-1.89281400
H	-6.58641200	-1.32258700	2.26238700
H	-5.67767900	0.96243600	2.16026800
H	-6.69662000	1.17348900	-2.00547000
H	-4.57359400	2.38303600	-1.88223000
H	-6.44617500	3.23961600	1.88580000
H	-5.56675500	5.52978100	1.79832700
C	-4.01204800	6.69071300	-0.13185300
H	-3.69768200	4.68055300	-1.96557300
H	-2.05795600	-7.66245400	1.25610300
H	-2.55087500	-3.40596000	1.25821100
H	-0.12569900	-3.12230700	0.99519100
H	0.36285200	-7.38889200	0.96118800
H	1.54306000	-3.38001400	-0.60960700
H	2.38724600	-6.68933500	1.99430700
H	4.78756500	-6.40739200	1.56536500
C	6.02324600	-4.58511400	-0.07537100

H	3.94396400	-3.08921300	-1.01240200
N	6.49740200	-3.20597200	-0.07053600
C	6.88428700	-2.59217800	-1.25109700
N	7.66146200	-1.48569100	-0.93528300
C	7.54270000	-1.23237200	0.50315400
C	7.29171400	-2.64679200	1.02565100
C	8.68708800	-0.58301500	1.26897700
C	7.83797000	-0.41203000	-1.91048400
C	6.78949200	0.68770400	-1.83896700
C	5.42241800	0.38932800	-1.88133800
C	4.46688800	1.39392600	-1.79088200
C	4.83654500	2.74447200	-1.67526700
C	6.20760300	3.03998200	-1.66165300
C	7.16327600	2.02894100	-1.73112100
C	3.81065400	3.81259500	-1.55592100
C	2.54408100	3.67844200	-2.15138400
C	1.57611400	4.66569100	-2.01476900
C	1.83504900	5.83328000	-1.28846300
C	3.09637400	5.98005700	-0.70925100
C	4.06510600	4.98689400	-0.83312700
N	-2.57497700	6.80152000	0.11294400
C	-1.66785700	6.74366200	-0.93035800
N	-0.40220100	6.49539900	-0.40013700
C	-0.47499300	6.69412700	1.05156300
C	-1.92762300	6.30616000	1.32810700
C	-1.43314800	6.16262700	3.74080100
C	-2.35882400	6.82279600	2.69418500

O	6.58461900	-2.96364300	-2.38119400
O	-1.92745300	6.88928700	-2.12012600
C	0.77730300	6.91110800	-1.15903400
H	-4.53296800	7.30888400	0.60275600
H	-4.18537700	7.13038400	-1.11517600
H	6.21539000	-4.98977500	-1.07359900
H	6.61653900	-5.17084200	0.63307800
H	6.63472700	-0.63485800	0.68153300
C	6.73606100	-2.59293900	2.44194100
H	9.60369900	-1.16995200	1.14583200
H	8.83151700	0.02234200	-1.77945700
H	7.81832700	-0.89085700	-2.89203900
H	5.09961200	-0.64026700	-1.98271400
H	3.41844600	1.12040700	-1.78983400
H	6.53723000	4.07069700	-1.60382000
H	8.21618100	2.29113100	-1.70590400
H	2.31868200	2.80056700	-2.74535500
H	0.60842900	4.53379800	-2.48739500
H	3.32454200	6.87570400	-0.14048600
H	5.02021300	5.12067600	-0.33908500
H	-0.36096800	7.77308100	1.25358900
H	-1.99709300	5.20672100	1.34340500
H	-1.65428200	6.56350500	4.73379400
H	-2.27173200	7.91409400	2.72835600
H	-3.39993300	6.56910900	2.90957300
H	0.41866700	7.19575600	-2.15049300
H	1.21339500	7.80444200	-0.69518200

	C	7.80140100	-1.89145300	3.31629400
	C	8.26211200	-0.52896600	2.75466800
	C	0.45772300	5.94278300	1.99326700
	C	0.06806200	6.34389900	3.43453900
	H	-5.09530900	-6.68120600	-3.11473200
	H	-3.90672700	-5.71130100	-3.96501700
	H	-7.48771700	-3.63731500	-2.72614400
	H	-7.39502000	-5.33507000	-2.25529600
	H	-5.30072600	-3.70836000	-3.80830800
	H	-6.27382000	-5.01413000	-4.45957900
	H	8.25549200	-3.18217500	1.06151100
	H	8.89089000	0.42756100	0.90447900
	H	-1.66325900	5.09139200	3.77968300
	H	6.53444800	-3.59454700	2.83178700
	H	5.79244900	-2.03735800	2.45534800
	H	8.66989700	-2.55501700	3.40356100
	H	7.41392800	-1.75454300	4.32957600
	H	9.08801800	-0.14673200	3.36078900
	H	7.44365800	0.19374200	2.85439400
	H	1.50398500	6.19610100	1.80834300
	H	0.35055500	4.86277200	1.84596600
	H	0.34045000	7.39501600	3.58736600
	H	0.65834500	5.76439100	4.14971700
Conf. 6	C	-5.10981200	-5.34158900	-3.26473700
	C	-4.31231800	-5.18560700	-1.94980200
	C	-5.33026800	-5.06119200	-0.82381000
	C	-6.21897500	-3.83706200	-1.04368000

C	-7.10404500	-4.04028200	-2.26632100
H	-3.67611500	-4.29505300	-1.98269100
H	-3.65961900	-6.04983600	-1.80484500
H	-5.96834700	-5.96130600	-0.84616800
N	-4.90062400	-4.84171600	0.56048900
N	-6.81974900	-3.69734900	0.28456500
H	-5.56847400	-2.96819500	-1.23328700
C	-6.16598400	-4.23788600	-3.47889700
C	-5.91987000	-4.17004100	1.22744000
O	-6.00702400	-4.02431800	2.44189400
C	-4.08410100	-5.81468000	1.27458700
C	-7.74555700	-2.61593100	0.62648100
C	-7.15503600	-1.21922000	0.52717600
C	-7.38873300	-0.41466500	-0.59145600
C	-6.35596900	-0.70400400	1.55572700
C	-5.81161600	0.57271100	1.46607300
C	-6.04096800	1.38104500	0.34140200
C	-6.83882200	0.86107900	-0.68730900
C	-5.46921300	2.74887700	0.24815500
C	-4.94809700	3.23543200	-0.96129000
C	-5.44699800	3.60096600	1.36109500
C	-4.93239800	4.89147800	1.26430900
C	-4.41498300	5.37343500	0.05868800
C	-4.42817500	4.52189000	-1.05318300
C	-2.59544300	-5.64946700	1.03393700
C	-1.78746100	-6.74953400	0.74027000
C	-1.99038100	-4.39217000	1.12957100

C	-0.62435400	-4.24000900	0.92681000
C	0.19239200	-5.34050600	0.61688300
C	-0.41756100	-6.60007000	0.53452600
C	1.64606900	-5.16674700	0.36843600
C	2.13335200	-4.01646800	-0.27388800
C	2.57920700	-6.13707100	0.76079000
C	3.94056200	-5.96344000	0.51982900
C	4.41742300	-4.81736200	-0.11978600
C	3.49151700	-3.84564700	-0.51169200
H	-4.39217000	-6.83293400	1.00530900
H	-4.30614500	-5.67849400	2.33629800
H	-8.07932400	-2.80989800	1.64647900
H	-8.61617900	-2.69894100	-0.02747100
H	-8.02263900	-0.77989600	-1.39294800
H	-6.15700700	-1.31468300	2.42916300
H	-5.18849900	0.94225800	2.27273600
H	-7.05516500	1.46926600	-1.55821800
H	-4.93789300	2.59478900	-1.83588100
H	-5.86286600	3.26386500	2.30376200
H	-4.95358100	5.53686700	2.13647000
C	-3.85882600	6.78348800	-0.05019100
H	-4.02267700	4.87335500	-1.99514600
H	-2.23392400	-7.73481700	0.65151800
H	-2.59777000	-3.52448700	1.36190600
H	-0.18212800	-3.25537800	1.02616200
H	0.17608500	-7.46905800	0.27457400
H	1.43829000	-3.25653500	-0.61199200

H	2.24350200	-7.02545100	1.28359200
H	4.64009500	-6.72675400	0.84566900
C	5.89380200	-4.65159800	-0.43174800
H	3.83969300	-2.94791700	-1.00963300
N	6.39860200	-3.30950600	-0.18016700
C	7.01122200	-2.58674300	-1.19367800
N	7.76451700	-1.57397300	-0.61464600
C	7.40199900	-1.48153800	0.80214400
C	7.01436800	-2.93072900	1.09365400
C	8.42012400	-0.99305900	1.82364600
C	8.13107400	-0.39599400	-1.40453100
C	7.05423000	0.67436700	-1.46698900
C	5.87312100	0.46135900	-2.19035700
C	4.87270200	1.42585400	-2.22465500
C	5.01366900	2.64428300	-1.53980700
C	6.20277200	2.85818400	-0.82873600
C	7.20337700	1.88960100	-0.79454300
C	3.93779100	3.66781500	-1.56235500
C	4.23712400	5.03718600	-1.58971300
C	3.22453100	5.99302100	-1.58629700
C	1.87957000	5.61804100	-1.56000000
C	1.57454200	4.25358300	-1.54172500
C	2.58324400	3.29674000	-1.54216700
N	-2.40113700	6.88085500	0.02882200
C	-1.61711600	6.65292900	-1.08796700
N	-0.30854000	6.41687900	-0.66561000
C	-0.21393600	6.81994200	0.74172900

C	-1.63466700	6.52689300	1.22440400
C	-0.87387900	6.70998000	3.56732600
C	-1.90037300	7.24328800	2.54212400
O	6.90354100	-2.80617100	-2.39569100
O	-2.00125900	6.66282900	-2.25215300
C	0.78711700	6.66935300	-1.59334100
H	-4.27924400	7.41029800	0.73889900
H	-4.14413300	7.22302000	-1.00676200
H	6.06641400	-4.85531700	-1.49226700
H	6.47980000	-5.38042700	0.13981300
H	6.49828000	-0.85862600	0.89751000
C	6.23227400	-3.01695900	2.39720100
H	9.32395700	-1.61015200	1.77712300
H	9.04823500	0.02356100	-0.98644200
H	8.36181600	-0.76037800	-2.40674800
H	5.74410000	-0.46707200	-2.73517400
H	3.97940000	1.24068900	-2.81021100
H	6.33930000	3.77945100	-0.27415100
H	8.10938600	2.08334600	-0.22923800
H	5.27082300	5.36167400	-1.62586900
H	3.48723200	7.04599300	-1.60495600
H	0.53723800	3.94005700	-1.50657600
H	2.31578600	2.24694900	-1.50091500
H	-0.05696600	7.91200900	0.77267300
H	-1.72551600	5.44300100	1.40030000
H	-0.97447600	7.25821200	4.50802900
H	-1.79219900	8.32509000	2.40843100

	H	-2.91490400	7.05732600	2.90464700
	H	0.33899300	6.70724100	-2.58970900
	H	1.22488500	7.65724000	-1.39709400
	C	7.16132000	-2.48833900	3.51447900
	C	7.75368700	-1.09481200	3.21488000
	C	0.80411300	6.17794900	1.67483700
	C	0.58633300	6.79477400	3.07521000
	H	-5.60907400	-6.31781600	-3.25489400
	H	-4.42034600	-5.35533200	-4.11336600
	H	-7.75595900	-3.18016800	-2.44011300
	H	-7.74605700	-4.91604900	-2.12268700
	H	-5.65611800	-3.28864800	-3.68152800
	H	-6.75827300	-4.46732000	-4.36899900
	H	7.93820800	-3.52135200	1.21677100
	H	8.71827700	0.04107400	1.63153800
	H	-1.11713500	5.66431300	3.78993900
	H	5.93275700	-4.04529500	2.61644800
	H	5.31998600	-2.41486700	2.33205300
	H	7.98076900	-3.20372300	3.65175500
	H	6.61644500	-2.45628600	4.46196900
	H	8.47819000	-0.83240100	3.99068100
	H	6.95378200	-0.34703200	3.27133900
	H	1.82698400	6.36618000	1.34008700
	H	0.66137300	5.09256700	1.69962400
	H	0.89163300	7.84742500	3.04203900
	H	1.24348800	6.30498600	3.79910300
Conf. 7	C	-5.65327700	-5.62314900	-3.10847400

C	-4.71496100	-5.35760700	-1.90904900
C	-5.60329900	-5.08140800	-0.70349300
C	-6.48317200	-3.85663000	-0.95580000
C	-7.49577400	-4.15342700	-2.05415400
H	-4.06641500	-4.49855100	-2.10786400
H	-4.07012500	-6.22381700	-1.73905900
H	-6.26141100	-5.95643600	-0.56525700
N	-5.02585200	-4.73836300	0.60046900
N	-6.94172700	-3.57177600	0.40564500
H	-5.83785400	-3.03135500	-1.29790200
C	-6.69684700	-4.50843300	-3.32859400
C	-5.96418000	-3.98213100	1.29723300
O	-5.93301800	-3.73121400	2.49676100
C	-4.16055600	-5.65222500	1.32690500
C	-7.81305700	-2.44225200	0.73648700
C	-7.17749800	-1.07088800	0.57654100
C	-7.36424100	-0.31385200	-0.58391500
C	-6.39138500	-0.52675700	1.59999900
C	-5.82015100	0.73413700	1.46896400
C	-6.00833100	1.49800000	0.30656400
C	-6.78638600	0.94621000	-0.72101400
C	-5.41785600	2.85525100	0.18057000
C	-4.85319000	3.29586600	-1.02678600
C	-5.42104300	3.74366000	1.26518300
C	-4.88746400	5.02393900	1.14353200
C	-4.32444900	5.45960500	-0.05921300
C	-4.31337100	4.57256000	-1.14291600

C	-2.67935500	-5.50204900	1.02204100
C	-1.79496500	-6.52998100	1.36546500
C	-2.15030000	-4.34607700	0.44677100
C	-0.78383600	-4.22502800	0.21105400
C	0.11150300	-5.24779300	0.55830800
C	-0.42744300	-6.40390300	1.14663400
C	1.56929600	-5.11045700	0.30663400
C	2.19289400	-3.85441300	0.32196300
C	2.37762500	-6.22815000	0.03851600
C	3.74102400	-6.09120000	-0.19719300
C	4.35222800	-4.83258500	-0.19061600
C	3.55519100	-3.71767000	0.06936900
H	-4.46992700	-6.68839700	1.13698900
H	-4.33035700	-5.45951200	2.38996000
H	-8.12671800	-2.58540300	1.77124500
H	-8.70405500	-2.51645000	0.10997500
H	-7.98571800	-0.70081800	-1.38499000
H	-6.22319900	-1.10464700	2.50119200
H	-5.20952800	1.12660800	2.27425900
H	-6.96897100	1.51938900	-1.62293500
H	-4.82393800	2.62706100	-1.87964400
H	-5.87089800	3.44242700	2.20435400
H	-4.92878800	5.69799800	1.99300300
C	-3.74564900	6.85827100	-0.19197800
H	-3.87377200	4.88806100	-2.08243700
H	-2.17793400	-7.43902400	1.81919400
H	-2.81809800	-3.54099100	0.16549400

H	-0.41455200	-3.32701700	-0.27014600
H	0.22935700	-7.21055000	1.45082800
H	1.61283100	-2.96804900	0.55086100
H	1.93442400	-7.21620500	-0.00495700
H	4.33542600	-6.97640400	-0.40246700
C	5.83469300	-4.71551300	-0.51074600
H	4.00806000	-2.73397300	0.08452000
N	6.41569300	-3.41689900	-0.22096100
C	6.99745700	-2.67120300	-1.23624800
N	7.81481200	-1.70904000	-0.65883600
C	7.53633200	-1.66945600	0.77941400
C	7.12123500	-3.11957100	1.02886000
C	8.62790900	-1.25792900	1.75814900
C	8.17620600	-0.51060700	-1.41978000
C	7.10928300	0.57217600	-1.43844900
C	5.91909900	0.39200000	-2.15623400
C	4.93506700	1.37327200	-2.16514700
C	5.10119400	2.57703100	-1.46051500
C	6.29655600	2.75637600	-0.75037800
C	7.28114900	1.77048600	-0.74110200
C	4.04583300	3.62184800	-1.47302600
C	4.37161100	4.98530400	-1.49057500
C	3.37742300	5.96032500	-1.49815100
C	2.02517000	5.61111100	-1.49244300
C	1.69401500	4.25283600	-1.47630200
C	2.68424200	3.27700600	-1.46575100
N	-2.29101600	6.93997500	-0.05881600

C	-1.46783000	6.69005800	-1.14218000
N	-0.17806700	6.44846700	-0.66769300
C	-0.13333700	6.86631800	0.73755100
C	-1.57391400	6.59243100	1.16894400
C	-0.90006300	6.79262300	3.53673900
C	-1.88154900	7.32561900	2.46823500
O	6.82156000	-2.83902000	-2.43829100
O	-1.80798700	6.68746500	-2.31996700
C	0.95423100	6.68304800	-1.55498700
H	-4.18839100	7.51507400	0.55971200
H	-3.98654100	7.27016100	-1.17268800
H	5.98316200	-4.89714900	-1.57896200
H	6.38971900	-5.49419700	0.02656600
H	6.65981100	-1.02520600	0.95502600
C	6.42088300	-3.24760900	2.37460300
H	9.50786500	-1.89724900	1.62872400
H	9.10357400	-0.11191700	-1.00407600
H	8.38907300	-0.84590700	-2.43612400
H	5.77090400	-0.52356700	-2.71700600
H	4.03592200	1.21457400	-2.74948300
H	6.45202500	3.66496300	-0.17997100
H	8.19395500	1.93841700	-0.17845800
H	5.41171300	5.28995300	-1.51447300
H	3.66043800	7.00810200	-1.51270400
H	0.65057300	3.95918300	-1.45525900
H	2.39611300	2.23248900	-1.43019600
H	0.03314500	7.95709600	0.76302900

H	-1.68246200	5.51151000	1.35261900
H	-1.03083200	7.35134800	4.46753600
H	-1.75682500	8.40480100	2.32790000
H	-2.91082400	7.15390100	2.79422200
H	0.54275600	6.73383500	-2.56661000
H	1.40303500	7.66161700	-1.33858100
C	7.43281800	-2.79802700	3.45347800
C	8.04389000	-1.40703400	3.18158300
C	0.84263800	6.22469900	1.71501000
C	0.57854100	6.85778800	3.10015800
H	-6.17374200	-6.57354600	-2.94057800
H	-5.05993000	-5.75152000	-4.01793000
H	-8.14191500	-3.29365100	-2.25079000
H	-8.14033300	-4.98657100	-1.75367500
H	-6.18641200	-3.60468600	-3.68192800
H	-7.38600800	-4.80552100	-4.12385000
H	8.03217900	-3.74088100	1.06444500
H	8.94549400	-0.22451800	1.59495800
H	-1.16259900	5.75181400	3.76032100
H	6.10539000	-4.27725100	2.56443900
H	5.52473700	-2.61945100	2.39786600
H	8.23911700	-3.53931400	3.50586100
H	6.94869800	-2.79777400	4.43391900
H	8.82098800	-1.19935200	3.92226100
H	7.26998900	-0.64296700	3.32098800
H	1.87927300	6.39972100	1.41716500
H	0.68835800	5.14106000	1.74546800

	H	0.89619700	7.90683500	3.06875000
	H	1.20241600	6.36837500	3.85316900
Conf. 8	C	-6.85130000	-5.20260000	-2.99310000
	C	-5.60690000	-5.19680000	-2.07580000
	C	-6.10170000	-4.93130000	-0.66110000
	C	-6.79970000	-3.57270000	-0.58930000
	C	-8.09840000	-3.60750000	-1.38370000
	H	-4.90250000	-4.41740000	-2.38380000
	H	-5.08760000	-6.15680000	-2.14560000
	H	-6.83980000	-5.71190000	-0.41100000
	N	-5.15940000	-4.81790000	0.45750000
	N	-6.82530000	-3.35970000	0.85890000
	H	-6.14150000	-2.81790000	-1.04860000
	C	-7.72700000	-3.93930000	-2.84770000
	C	-5.73690000	-4.00200000	1.42420000
	O	-5.34890000	-3.88270000	2.58090000
	C	-4.31030000	-5.92260000	0.87650000
	C	-7.41080000	-2.18490000	1.49130000
	C	-6.80510000	-0.85050000	1.08250000
	C	-7.62830000	0.21820000	0.71980000
	C	-5.42090000	-0.64190000	1.09880000
	C	-4.88150000	0.59440000	0.76130000
	C	-5.70640000	1.67450000	0.40960000
	C	-7.09160000	1.46230000	0.39630000
	C	-5.12830000	3.00150000	0.07690000
	C	-5.71320000	4.18240000	0.55290000
	C	-3.97330000	3.11390000	-0.71410000

C	-3.42800000	4.35680000	-1.01550000
C	-4.00800000	5.53390000	-0.52630000
C	-5.15640000	5.42540000	0.26090000
C	-2.83000000	-5.75350000	0.56870000
C	-1.92080000	-6.69550000	1.06390000
C	-2.33000000	-4.68790000	-0.17860000
C	-0.96420000	-4.56580000	-0.42520000
C	-0.04570000	-5.49860000	0.07630000
C	-0.55720000	-6.57100000	0.82640000
C	1.41280000	-5.34600000	-0.15950000
C	2.00240000	-4.07510000	-0.23870000
C	2.25600000	-6.45990000	-0.29710000
C	3.62490000	-6.30620000	-0.49520000
C	4.20400000	-5.03610000	-0.57440000
C	3.36950000	-3.92370000	-0.44780000
H	-4.67720000	-6.84280000	0.40740000
H	-4.42880000	-6.04670000	1.95630000
H	-7.28990000	-2.33120000	2.56750000
H	-8.48340000	-2.17480000	1.28510000
H	-8.70410000	0.07860000	0.68800000
H	-4.75840000	-1.44530000	1.40000000
H	-3.80700000	0.73320000	0.80160000
H	-7.75560000	2.26920000	0.10740000
H	-6.59410000	4.12870000	1.18250000
H	-3.51180000	2.21930000	-1.11710000
H	-2.55100000	4.41460000	-1.65070000
C	-3.41780000	6.89650000	-0.85180000

H	-5.62020000	6.32260000	0.65810000
H	-2.28160000	-7.52870000	1.65940000
H	-3.01480000	-3.94460000	-0.56760000
H	-0.61240000	-3.73640000	-1.02800000
H	0.12090000	-7.30060000	1.25390000
H	1.38960000	-3.19020000	-0.11120000
H	1.83810000	-7.45940000	-0.26070000
H	4.24920000	-7.18900000	-0.59370000
C	5.69340000	-4.89120000	-0.84100000
H	3.79940000	-2.93030000	-0.49180000
N	6.26550000	-3.64540000	-0.35990000
C	6.98720000	-2.82780000	-1.21800000
N	7.75270000	-1.95950000	-0.45210000
C	7.30510000	-2.05070000	0.94000000
C	6.82060000	-3.49990000	0.98830000
C	8.28290000	-1.78540000	2.07680000
C	8.24700000	-0.71150000	-1.03530000
C	7.23840000	0.42650000	-1.04730000
C	6.07380000	0.34260000	-1.82220000
C	5.14750000	1.37810000	-1.83200000
C	5.34980000	2.54330000	-1.07370000
C	6.51920000	2.62630000	-0.30580000
C	7.44440000	1.58400000	-0.29290000
C	4.35710000	3.64760000	-1.10090000
C	4.75970000	4.99070000	-1.08930000
C	3.82360000	6.02010000	-1.14030000
C	2.45490000	5.74680000	-1.20650000

C	2.04710000	4.41010000	-1.20360000
C	2.97980000	3.37990000	-1.15110000
N	-2.01780000	7.06470000	-0.47720000
C	-1.00330000	6.79540000	-1.37910000
N	0.18920000	6.66610000	-0.67140000
C	-0.03370000	7.17000000	0.68700000
C	-1.51410000	6.84270000	0.87940000
C	-1.27950000	7.21990000	3.30630000
C	-2.08050000	7.63440000	2.05040000
O	6.95350000	-2.86720000	-2.44350000
O	-1.12780000	6.68820000	-2.59420000
C	1.45490000	6.87790000	-1.36000000
H	-4.00760000	7.67460000	-0.36190000
H	-3.46440000	7.08030000	-1.92740000
H	5.87390000	-4.91880000	-1.91950000
H	6.23060000	-5.74200000	-0.40360000
H	6.43390000	-1.39050000	1.07800000
C	5.95710000	-3.73020000	2.22090000
H	9.15220000	-2.44550000	1.98460000
H	9.14050000	-0.40890000	-0.48570000
H	8.55430000	-0.95140000	-2.05480000
H	5.90250000	-0.53680000	-2.43220000
H	4.26810000	1.29470000	-2.46030000
H	6.69930000	3.50270000	0.30640000
H	8.33680000	1.67450000	0.31800000
H	5.81560000	5.23500000	-1.06290000
H	4.16560000	7.05030000	-1.13990000

H	0.98910000	4.17670000	-1.23320000
H	2.63300000	2.35280000	-1.13420000
H	0.07810000	8.26760000	0.66620000
H	-1.60540000	5.77140000	1.11910000
H	-1.59720000	7.82290000	4.16130000
H	-1.98170000	8.70880000	1.86130000
H	-3.14250000	7.42230000	2.20110000
H	1.21370000	6.99960000	-2.41990000
H	1.90620000	7.81840000	-1.02100000
C	6.84240000	-3.43310000	3.45250000
C	7.52660000	-2.05050000	3.39910000
C	0.78190000	6.64170000	1.85890000
C	0.24920000	7.34110000	3.13080000
H	-7.45450000	-6.08670000	-2.75510000
H	-6.54000000	-5.31120000	-4.03560000
H	-8.61850000	-2.64650000	-1.34160000
H	-8.77170000	-4.36610000	-0.97010000
H	-7.19000000	-3.08230000	-3.27110000
H	-8.63800000	-4.05750000	-3.44080000
H	7.70230000	-4.15830000	1.06900000
H	8.64910000	-0.75530000	2.06000000
H	-1.53010000	6.18050000	3.54930000
H	5.58950000	-4.75890000	2.26470000
H	5.08370000	-3.07050000	2.20060000
H	7.61210000	-4.21080000	3.52390000
H	6.24230000	-3.50720000	4.36360000
H	8.21480000	-1.95160000	4.24310000

	H	6.76640000	-1.27060000	3.52580000
	H	1.84680000	6.85510000	1.73310000
	H	0.67110000	5.55500000	1.93630000
	H	0.52120000	8.40230000	3.08360000
	H	0.75000000	6.93260000	4.01280000

5a (*in vacuo*)

Conf. 1	C	-1.38780200	2.01384700	1.46125900
	C	1.40517000	1.91770300	2.63114800
	C	0.91847600	2.96055600	1.63241700
	C	-0.11614300	2.34576000	0.68681700
	H	-1.81003700	2.92969200	1.88923600
	H	-2.14931500	1.57971900	0.81126900
	H	2.16973800	2.32618900	3.29857800
	H	0.42997500	3.76873200	2.20565700
	N	1.83577100	3.55561000	0.67354400
	H	0.30324700	1.40442400	0.29274000
	N	-0.13644300	3.32784200	-0.40460800
	H	1.85435000	1.07193900	2.10118600
	C	-5.04859000	2.93082000	-1.74351400
	C	-2.97144700	4.13982000	-1.48727600
	C	-4.36030200	4.12640000	-1.52163700
	C	-2.24872000	2.95696200	-1.68954700
	C	-2.94905900	1.77441100	-1.91534700
	C	-4.34816500	1.74183600	-1.93348100
	H	-6.13419700	2.92783000	-1.76448300
	H	-2.43715000	5.06578500	-1.30436200
	C	-0.46094800	-3.12756400	1.95610300

C	-1.73425600	-3.50396400	1.54002700
C	-2.06095100	-3.50163400	0.17887800
H	-0.21695500	-3.12242700	3.01305500
C	5.25985600	2.99475000	1.85899800
C	4.27974700	2.19698200	-0.18656200
C	4.25100400	3.07585400	0.89799700
H	5.25352200	3.67145300	2.70822900
C	-1.00546900	1.00578400	2.56907700
C	0.17499200	1.46648200	3.44885700
H	-0.74648500	0.05212100	2.09547300
H	-1.87495700	0.80755800	3.20371700
H	-0.15041200	2.30313100	4.07966700
H	0.45944100	0.66003700	4.13150200
C	-1.07659200	-3.14776000	-0.74663200
C	0.50603100	-2.75848600	1.02103000
C	0.21157100	-2.78288800	-0.34151500
H	-1.33462700	-3.13565700	-1.80087700
H	1.49108300	-2.44825200	1.34493200
C	6.14332500	-2.77379300	-0.86733700
C	3.62430100	-4.15354800	0.09399300
C	3.64758000	-2.98685400	-0.88525600
C	4.82753300	-2.05918700	-0.58508300
H	6.19470800	-3.05547400	-1.92483300
H	7.00310800	-2.12985600	-0.66033100
H	2.77511000	-4.81647100	-0.09249300
H	3.79663900	-3.40584100	-1.89701200
N	2.54436000	-2.02450700	-0.94621600

H	4.80130500	-1.79895700	0.48656700
N	4.45263100	-0.89332600	-1.37820600
H	3.52817700	-3.78550500	1.12059100
C	6.27675900	2.04865300	1.73468100
C	6.29150800	1.17456300	0.65057900
C	5.28761000	1.24073400	-0.32169400
H	7.05861400	1.99448200	2.48446400
C	6.19957600	-4.02497000	0.03775900
C	4.95152500	-4.92472700	-0.08180100
H	6.30492600	-3.69759400	1.07917900
H	7.09551000	-4.60840700	-0.19282800
H	4.94930900	-5.40607500	-1.06725900
H	5.01159100	-5.73166900	0.65400700
H	3.50113800	2.24735200	-0.93755000
C	-5.63355900	-2.53991800	1.83742700
C	-5.76184800	0.38808800	1.06197100
C	-5.54459800	-0.75722600	0.08044700
C	-4.77595100	-1.89362500	0.75706200
H	-6.54461100	-2.95531100	1.39281400
H	-5.10908800	-3.36379700	2.33077100
H	-6.29554000	1.22066400	0.59653500
H	-6.53883400	-1.13980100	-0.21328300
N	-4.74374700	-0.57116800	-1.12576000
H	-3.88685400	-1.45855700	1.24264000
N	-4.35624900	-2.67247700	-0.40572900
H	-4.79748200	0.77666900	1.40487800
C	-5.97383700	-1.44368500	2.87250500

C	-6.58514300	-0.17392800	2.24360000
H	-5.05428000	-1.17086500	3.40422300
H	-6.65781700	-1.84380300	3.62659700
H	-7.59607100	-0.40410300	1.88543500
H	-6.70068600	0.59481000	3.01310300
C	1.15044900	3.89597300	-0.47817400
C	-0.73520300	2.95932100	-1.69129600
H	-0.35773400	3.68654700	-2.41286000
H	-0.36626100	1.97188000	-2.00231800
C	3.13764100	4.11004200	0.98657800
H	3.31832300	4.90648400	0.25850400
H	3.12415400	4.56743700	1.98284300
C	3.07148700	-0.81941000	-1.44466900
C	5.27523600	0.30919700	-1.52203100
H	6.29047200	-0.01645500	-1.75974800
H	4.88993300	0.84295600	-2.39205500
C	1.22312500	-2.41643000	-1.41622200
H	0.83526500	-1.57247200	-1.99208300
H	1.32128400	-3.26003500	-2.11494300
C	-4.18123900	-1.79390900	-1.47721400
C	-5.05875300	0.41835500	-2.15081300
H	-6.14390000	0.57437200	-2.19205000
H	-4.74880400	-0.01957200	-3.10303200
C	-3.46715900	-3.83287200	-0.29361100
H	-3.43262100	-4.28907100	-1.28336500
H	-3.93764100	-4.54814500	0.38535900
H	-2.40098400	0.85034600	-2.07232300

	H	-2.47628900	-3.80234500	2.27404400
	H	7.09392100	0.44949900	0.55386100
	H	-4.91393800	5.04735700	-1.37380100
	O	1.58810000	4.55942200	-1.39967600
	O	2.41829200	0.11569600	-1.87246500
	O	-3.62990900	-2.05335000	-2.53285000
Conf. 3	C	0.44831800	4.14823800	1.34549900
	C	3.43471400	3.65749200	1.57103500
	C	2.62643000	3.49439600	0.29061200
	C	1.20189000	3.04355900	0.61715100
	H	0.38483900	5.03882600	0.71114700
	H	-0.57522500	3.84565400	1.57981300
	H	4.46318700	3.96450700	1.35917400
	H	2.57346200	4.48060700	-0.20363100
	N	3.00505700	2.49319700	-0.70220000
	H	1.26761800	2.17543100	1.29693600
	N	0.74653800	2.56434000	-0.69035700
	H	3.48577500	2.70379700	2.10625200
	C	-4.22500500	3.83736900	-1.39563100
	C	-1.83934500	3.71444100	-1.78746900
	C	-3.06723900	4.33739700	-1.99233600
	C	-1.75912300	2.59139300	-0.96016500
	C	-2.91734500	2.12707500	-0.33805100
	C	-4.16208800	2.72404900	-0.55594700
	H	-5.17991100	4.31882600	-1.58350200
	H	-0.94391600	4.08771500	-2.27098500
	C	-0.64858200	-0.84062100	3.50525900

C	-2.01293600	-0.90589100	3.23105000
C	-2.52519500	-1.95523700	2.46248300
H	-0.26194200	-0.03029000	4.11405200
C	6.60186300	1.46992600	-1.11392300
C	4.71443500	0.14192800	-0.43102400
C	5.22447700	1.34303900	-0.92718500
H	7.01483400	2.39968400	-1.49369900
C	1.22443600	4.44906800	2.64816300
C	2.72507900	4.73433600	2.42311100
H	1.12261100	3.58859800	3.32031200
H	0.76532100	5.29685800	3.16443600
H	2.83327100	5.70361500	1.92108500
H	3.22750300	4.83134700	3.38965400
C	-1.64403600	-2.92934600	1.98130600
C	0.21834200	-1.81917200	3.02865000
C	-0.27443800	-2.87814300	2.25709000
H	-2.03526200	-3.75765500	1.39550600
H	1.27479200	-1.78478600	3.26829000
C	3.01662600	-2.20588500	-2.28680600
C	0.74942400	-4.04897900	-1.47383600
C	1.50819900	-3.14591900	-0.50991000
C	2.95808600	-2.97406500	-0.97181000
H	2.58330100	-1.20743400	-2.17939200
H	4.04844000	-2.07332700	-2.62277000
H	-0.29020500	-4.18330900	-1.15982500
H	1.02251800	-2.15646000	-0.51512400
N	1.72204000	-3.57556700	0.86894700

H	3.37454700	-3.98203900	-1.15425900
N	3.57321500	-2.44453900	0.25043000
H	1.21437900	-5.04100100	-1.50080400
C	7.44788800	0.40008000	-0.82942800
C	6.92392100	-0.80315500	-0.36780300
C	5.54770300	-0.94200300	-0.16096500
H	8.51757300	0.50279100	-0.97623500
C	2.22549500	-3.02910600	-3.32786600
C	0.79625900	-3.38293400	-2.86714800
H	2.77333200	-3.95614100	-3.53964700
H	2.17690900	-2.47638600	-4.27002800
H	0.19779100	-2.46478100	-2.83516100
H	0.32231500	-4.03628600	-3.60577100
H	3.65057100	0.04003500	-0.26803600
C	-4.59031500	-2.58085600	-1.03720500
C	-6.33985700	-0.26394700	-1.91935900
C	-5.27761500	-0.18628900	-0.83140800
C	-5.16276900	-1.52602800	-0.09976500
H	-3.59440800	-2.27851500	-1.37775400
H	-4.48516600	-3.54923400	-0.53847600
H	-6.44069900	0.68925800	-2.44646000
H	-4.31189700	0.01424900	-1.32168500
N	-5.44616500	0.73499000	0.29099700
H	-6.17769500	-1.85046700	0.19128100
N	-4.43368600	-1.11268900	1.09858000
H	-7.31428000	-0.49968600	-1.47738300
C	-5.56748500	-2.71179800	-2.22889300

C	-5.89764300	-1.36558700	-2.90981100
H	-6.49735700	-3.16685000	-1.86691700
H	-5.15632900	-3.40320200	-2.96997900
H	-5.01074200	-1.01096600	-3.44820600
H	-6.67338400	-1.52122600	-3.66503500
C	1.86839300	2.06906300	-1.36984200
C	-0.48287800	1.79024700	-0.80104600
H	-0.35223800	1.15484600	-1.68170400
H	-0.58179800	1.12447300	0.06782600
C	4.29658700	2.46401700	-1.36985500
H	4.10344500	2.34396000	-2.44121700
H	4.78909600	3.43264100	-1.23305400
C	2.89799600	-3.01408300	1.33948200
C	5.01073200	-2.26048100	0.37158900
H	5.23282500	-2.34042500	1.43950300
H	5.53743800	-3.08853000	-0.12700100
C	0.66442300	-3.98072400	1.78844200
H	0.08907400	-4.77888100	1.31251300
H	1.17222100	-4.41428400	2.65291400
C	-4.81204300	0.18667200	1.40897100
C	-5.41658400	2.18443800	0.12177500
H	-6.30109600	2.49731000	-0.43950400
H	-5.50567600	2.60139400	1.12833100
C	-4.01584600	-2.02277800	2.16907000
H	-4.28920100	-3.03694700	1.86980300
H	-4.57610100	-1.77894900	3.07711100
H	-2.84034500	1.26966500	0.32009800

	H	-2.68980400	-0.14336100	3.59623600
	H	7.58730400	-1.63835900	-0.16410100
	H	-3.12861700	5.20708400	-2.63766700
	O	-4.62211200	0.75511500	2.47038900
	O	1.84670100	1.37665500	-2.37355800
	O	3.28514900	-3.01835900	2.49572800

5a (ACN)

Conf. 1	C	1.50952200	-2.29738600	1.53129600
	C	-1.27162000	-2.16776100	2.72955400
	C	-0.85645300	-3.09805400	1.59780900
	C	0.21857300	-2.43874300	0.73254400
	H	1.86817100	-3.28651800	1.83609200
	H	2.29837600	-1.83154500	0.93813900
	H	-2.05933200	-2.61356400	3.34339000
	H	-0.43222100	-4.00940300	2.05126700
	N	-1.81703500	-3.49788700	0.57290600
	H	-0.13081400	-1.42721600	0.46484200
	N	0.16794700	-3.27229800	-0.47945800
	H	-1.66135300	-1.22898800	2.32282500
	C	5.10850000	-2.89315600	-1.77255200
	C	2.99625600	-4.05469200	-1.56054300
	C	4.38624200	-4.07700200	-1.59469700
	C	2.30792800	-2.84383400	-1.71184900
	C	3.04088400	-1.67323200	-1.89416700
	C	4.44048700	-1.67864500	-1.91978500
	H	6.19328700	-2.91926900	-1.78904600
	H	2.44009400	-4.97417100	-1.41310300

C	0.41687800	2.74244400	1.90629000
C	1.67396900	3.19049100	1.51196100
C	1.95832100	3.38722700	0.15377300
H	0.20529400	2.58479300	2.95817000
C	-5.22979200	-3.01206400	1.80172300
C	-4.32975900	-2.15483600	-0.25839700
C	-4.24897800	-3.05368700	0.80838500
H	-5.18168500	-3.70282700	2.63747500
C	1.19588600	-1.42035500	2.76540000
C	-0.01252700	-1.91386100	3.58787500
H	1.00016700	-0.39756900	2.42279000
H	2.07782400	-1.36710600	3.41006700
H	0.25547100	-2.84756500	4.09646400
H	-0.24133400	-1.18795100	4.37306900
C	0.94610500	3.16080300	-0.78156600
C	-0.57765600	2.50083100	0.95709000
C	-0.32735100	2.72547100	-0.39609200
H	1.16205500	3.31567600	-1.83384900
H	-1.55105400	2.14255500	1.26668900
C	-6.26945500	2.77978100	-0.65542800
C	-3.69828000	4.10595700	0.25152700
C	-3.78212800	3.01486900	-0.80692900
C	-4.93586400	2.05911300	-0.50128600
H	-6.38343800	3.13819800	-1.68421900
H	-7.11280300	2.11684900	-0.44390400
H	-2.86648100	4.78789500	0.05558400
H	-3.98990700	3.50419800	-1.77402500

N	-2.68043400	2.06485300	-1.00871300
H	-4.84447600	1.72593800	0.54518900
N	-4.59918300	0.94569900	-1.39050800
H	-3.53731000	3.66624600	1.24077600
C	-6.27067500	-2.08650300	1.72453600
C	-6.33858000	-1.19400500	0.65652200
C	-5.36327600	-1.21875400	-0.34710200
H	-7.02918700	-2.06150500	2.49908500
C	-6.27177400	3.96048300	0.34161900
C	-5.03708600	4.87648700	0.21172800
H	-6.31098800	3.55541500	1.35973100
H	-7.18166000	4.55192500	0.20817700
H	-5.09592800	5.42651700	-0.73490100
H	-5.05411900	5.62596400	1.00766700
H	-3.57530800	-2.17648400	-1.03516400
C	5.67453300	2.73605800	1.78208300
C	5.99251700	-0.19597500	1.07176100
C	5.66105400	0.90640100	0.07471800
C	4.83261000	1.99977800	0.74866200
H	6.53971600	3.20130700	1.29769000
H	5.10807600	3.53200400	2.27360700
H	6.57545300	-0.99501800	0.60568900
H	6.61290900	1.34893200	-0.26483000
N	4.83619200	0.63200200	-1.10312000
H	3.99481700	1.51335300	1.27301100
N	4.31053400	2.71216600	-0.42195900
H	5.07175300	-0.64401300	1.46014300

C	6.12453200	1.69196200	2.82935600
C	6.81015500	0.45613400	2.21068500
H	5.24392400	1.36626000	3.39534000
H	6.79987900	2.16080900	3.55029600
H	7.78798400	0.75093500	1.81166000
H	7.00512700	-0.28246300	2.99308200
C	-1.15095700	-3.73579000	-0.60686200
C	0.79539000	-2.79187200	-1.71486000
H	0.39917300	-3.41879800	-2.51578400
H	0.47127200	-1.76166000	-1.91805400
C	-3.12316600	-4.07612700	0.85055500
H	-3.30075400	-4.84599900	0.09442300
H	-3.10055900	-4.57191800	1.82531700
C	-3.22707700	0.89890000	-1.54893100
C	-5.41669400	-0.26115700	-1.52730000
H	-6.44501300	0.05899100	-1.70460500
H	-5.07316500	-0.77043600	-2.42880200
C	-1.37502800	2.50497000	-1.47784500
H	-1.01232600	1.74383100	-2.17284600
H	-1.49379000	3.43128100	-2.05530900
C	4.18013800	1.79828400	-1.46195400
C	5.18871000	-0.36958100	-2.10462800
H	6.26863300	-0.54453400	-2.07677800
H	4.94748100	0.05675400	-3.08187700
C	3.34483800	3.81184200	-0.30355100
H	3.28385900	4.28684500	-1.28295800
H	3.76720700	4.54230800	0.38944600

	H	2.51642500	-0.73019200	-2.01072200
	H	2.43251000	3.39151000	2.26074700
	H	-7.15749300	-0.48461600	0.59778900
	H	4.91460600	-5.01691600	-1.47840000
	O	-1.62971800	-4.25453000	-1.60893400
	O	-2.59303700	-0.00328600	-2.08530400
	O	3.58721900	1.99181000	-2.51793100
Conf. 2	C	-0.88111700	2.82836600	2.05064700
	C	2.14183000	2.92035200	2.28702200
	C	1.34900900	3.46241800	1.10557200
	C	0.08282600	2.63400500	0.88625000
	H	-1.17526500	3.88107900	2.12166600
	H	-1.79174400	2.24074400	1.91802100
	H	3.06162300	3.48999700	2.44579100
	H	1.05383000	4.49793900	1.34442900
	N	1.91728400	3.44349800	-0.24066100
	H	0.37104900	1.56948000	0.85864800
	N	-0.28747600	3.03700000	-0.47891000
	H	2.42684800	1.87915400	2.10350000
	C	-5.39595000	3.32837400	-0.33084600
	C	-3.20795500	3.96555800	-1.14160500
	C	-4.53552600	4.28568200	-0.86792900
	C	-2.73191300	2.67725700	-0.88236300
	C	-3.60267400	1.73149600	-0.33816600
	C	-4.93492700	2.03947900	-0.04987600
	H	-6.42936100	3.58828500	-0.12359200
	H	-2.54259900	4.71180800	-1.56228400

C	-0.00666800	-1.98562200	2.93247700
C	-1.36214600	-2.29384700	2.84951400
C	-1.89861100	-2.78765500	1.65734700
H	0.40224200	-1.59372100	3.85734500
C	5.54143300	3.23742400	0.02682300
C	4.17301200	1.83115200	-1.36655500
C	4.33111600	3.00923700	-0.63128400
H	5.67991100	4.14715100	0.60224300
C	-0.14948200	2.37191900	3.33435600
C	1.23105700	3.03140000	3.52964800
H	-0.02298800	1.28394300	3.29128800
H	-0.77788800	2.57610600	4.20578000
H	1.09230200	4.09329500	3.76524600
H	1.73031800	2.58493700	4.39409000
C	-1.04772000	-2.98202600	0.56651600
C	0.82842600	-2.16874400	1.83042000
C	0.31633500	-2.68030200	0.63717200
H	-1.45587500	-3.36966300	-0.36300400
H	1.87687200	-1.90746500	1.89233800
C	6.15144800	-3.01123200	-0.72153200
C	3.87709200	-3.97062800	1.03826800
C	3.69788600	-3.24869700	-0.29020100
C	4.84119400	-2.25989000	-0.52081800
H	6.07817000	-3.66332400	-1.59856100
H	6.98661200	-2.32617300	-0.89096500
H	3.05703400	-4.66850400	1.22870900
H	3.73614800	-4.00701500	-1.09081800

N	2.53181900	-2.38999300	-0.53540000
H	4.94313300	-1.62794200	0.37603000
N	4.28031500	-1.46049700	-1.61098000
H	3.89620800	-3.25106000	1.86303100
C	6.57260800	2.30128500	-0.05176100
C	6.40273900	1.13110300	-0.78874100
C	5.19686000	0.88443100	-1.45529900
H	7.50963500	2.48565800	0.46193700
C	6.40751300	-3.83925400	0.55807800
C	5.21758400	-4.73579600	0.95893000
H	6.62741900	-3.14855700	1.38069700
H	7.30081700	-4.45538500	0.42413500
H	5.11495200	-5.54623600	0.22765000
H	5.42727700	-5.21117300	1.92105300
H	3.23810800	1.64373600	-1.88181800
C	-4.60616300	-3.29998900	-1.52388300
C	-6.79916300	-1.20246900	-1.59979400
C	-5.51591400	-1.08841600	-0.78877500
C	-5.05252200	-2.47167900	-0.32804500
H	-3.77604300	-2.80309600	-2.03722000
H	-4.26006300	-4.29217400	-1.22060300
H	-7.15159600	-0.22175000	-1.93152000
H	-4.73891800	-0.66713800	-1.44567800
N	-5.51049200	-0.36477800	0.48436900
H	-5.90978200	-2.98477700	0.13960200
N	-4.09889400	-2.09990500	0.72459400
H	-7.59175800	-1.64962900	-0.99022600

C	-5.82907800	-3.43831700	-2.46050700
C	-6.48171900	-2.08814700	-2.82684200
H	-6.57379700	-4.07390200	-1.96699200
H	-5.53435500	-3.95988200	-3.37520400
H	-5.80807200	-1.53333900	-3.49038300
H	-7.39705900	-2.26953300	-3.39681000
C	0.89765800	3.33801100	-1.16069900
C	-1.30612900	2.28661300	-1.21403200
H	-1.10871900	2.46859700	-2.27217600
H	-1.17090900	1.21149100	-1.03582600
C	3.20390600	4.02857900	-0.58997800
H	3.08944700	4.48634900	-1.57635800
H	3.44362100	4.82789000	0.11761200
C	2.90364500	-1.44388200	-1.49275400
C	4.99577700	-0.37579900	-2.28257500
H	5.96191500	-0.76876300	-2.60511200
H	4.42370300	-0.13734400	-3.18039400
C	1.18186700	-2.93636500	-0.58876600
H	0.68869700	-2.50411300	-1.46246600
H	1.24839000	-4.01805500	-0.76173400
C	-4.55458200	-0.93025700	1.31644400
C	-5.86814600	1.03658900	0.62100100
H	-6.88493900	1.17594400	0.24613600
H	-5.89587300	1.24262300	1.69434900
C	-3.38403600	-3.08548100	1.54113900
H	-3.52971100	-4.05947300	1.07022000
H	-3.82681900	-3.13195800	2.54075400

	H	-3.22004200	0.73834100	-0.13366100
	H	-2.01223800	-2.13115900	3.70110200
	H	7.21408900	0.41367800	-0.85565800
	H	-4.90562600	5.28278300	-1.07997900
	O	1.00850700	3.47699500	-2.37383100
	O	2.13180100	-0.73589000	-2.12994900
	O	-4.18534600	-0.48028100	2.39510300
Conf. 3	C	0.14005000	3.64887600	1.53254200
	C	3.09851700	3.15131000	1.98666800
	C	2.44428500	3.29210400	0.61912300
	C	1.01266000	2.75738300	0.65865600
	H	0.11421300	4.66428200	1.12309700
	H	-0.88901600	3.28424400	1.57107000
	H	4.13071700	3.51256300	1.97497500
	H	2.41092900	4.36535600	0.36853000
	N	2.97151200	2.56369700	-0.53630200
	H	1.03316400	1.75259200	1.11449900
	N	0.72664800	2.59920300	-0.77325800
	H	3.12073100	2.09840400	2.28646700
	C	-4.21616400	3.95288100	-1.37727000
	C	-1.84696900	3.88222500	-1.87136100
	C	-3.07854300	4.53030500	-1.94128700
	C	-1.74582800	2.64718400	-1.22624900
	C	-2.88806300	2.09194700	-0.64683000
	C	-4.13162100	2.72454400	-0.71592300
	H	-5.17157500	4.46324100	-1.44829700
	H	-0.96707600	4.32970100	-2.31996500

C	-0.45809500	-0.81984200	3.54891300
C	-1.83097200	-0.86322600	3.31019300
C	-2.37859000	-1.87804000	2.52085000
H	-0.04403500	-0.03225600	4.16896800
C	6.61733400	1.66772900	-1.01004900
C	4.74595200	0.27354800	-0.41545200
C	5.24501000	1.52388200	-0.78670800
H	7.02131800	2.63418000	-1.29532200
C	0.75389300	3.64097300	2.95101500
C	2.25774500	3.98727700	2.97831200
H	0.60947400	2.64482600	3.38554400
H	0.20784500	4.33921900	3.59135300
H	2.38654500	5.04872700	2.73538900
H	2.64420900	3.85311500	3.99233600
C	-1.52339500	-2.84844200	1.98496800
C	0.38159900	-1.79178500	3.01261900
C	-0.14599000	-2.82127500	2.22291500
H	-1.94057300	-3.65137900	1.38375600
H	1.44552800	-1.76952400	3.21716100
C	2.96109300	-2.03078600	-2.41720800
C	0.71260000	-3.88728900	-1.57419300
C	1.52049800	-3.02245700	-0.61561800
C	2.94946600	-2.84933500	-1.13336800
H	2.54354100	-1.03436200	-2.24488800
H	3.97900400	-1.89733900	-2.79305000
H	-0.31331700	-4.02453400	-1.22183100
H	1.04447100	-2.03002800	-0.56470000

N	1.78994500	-3.49480300	0.74315600
H	3.34929600	-3.84999500	-1.37267100
N	3.62749300	-2.37386300	0.08059500
H	1.16819100	-4.88022300	-1.65685000
C	7.46673400	0.57133500	-0.87985900
C	6.95437200	-0.67613700	-0.53136700
C	5.58592700	-0.83469900	-0.29133500
H	8.53031500	0.68788900	-1.05704800
C	2.11498700	-2.80567400	-3.45307200
C	0.70516200	-3.17098200	-2.94334000
H	2.64767700	-3.72531700	-3.72299600
H	2.02904100	-2.21709400	-4.37062800
H	0.10911600	-2.25512900	-2.85334800
H	0.19982900	-3.79779400	-3.68326100
H	3.68668200	0.15685200	-0.23027100
C	-4.61906500	-2.73115500	-0.86102300
C	-6.39713100	-0.46351900	-1.81674200
C	-5.25145600	-0.31225500	-0.82577900
C	-5.10346800	-1.58316100	0.01234500
H	-3.65011200	-2.48422900	-1.30766300
H	-4.49147800	-3.65075600	-0.28308500
H	-6.52493900	0.43872200	-2.42142900
H	-4.32418800	-0.17296200	-1.40241700
N	-5.32038200	0.70503900	0.22621000
H	-6.09623900	-1.85642800	0.40753700
N	-4.28302300	-1.08189800	1.12248200
H	-7.33642600	-0.63810900	-1.28100500

C	-5.69116800	-2.94768400	-1.95480200
C	-6.05138600	-1.66196300	-2.73095600
H	-6.59599200	-3.34327800	-1.47841300
H	-5.35084600	-3.71338600	-2.65720800
H	-5.20435600	-1.37937900	-3.36712800
H	-6.88882200	-1.86633200	-3.40364100
C	1.93530000	2.30678400	-1.41136300
C	-0.45317900	1.85647800	-1.20709900
H	-0.23071900	1.50160100	-2.21619900
H	-0.58341700	0.96989100	-0.57218500
C	4.32987600	2.71712700	-1.02980000
H	4.27381600	2.89289100	-2.10859300
H	4.76682200	3.61485900	-0.58211100
C	2.99275500	-2.97145800	1.17187400
C	5.07187000	-2.20358700	0.12823100
H	5.36896800	-2.39522000	1.16259700
H	5.55511600	-2.97352400	-0.48892800
C	0.76108400	-3.92067900	1.68724100
H	0.15882000	-4.69233600	1.20364900
H	1.28841000	-4.39372400	2.51832300
C	-4.62865800	0.24336100	1.33914400
C	-5.37078800	2.13316400	-0.04926000
H	-6.25025400	2.33990500	-0.66349100
H	-5.53238400	2.62804800	0.91176100
C	-3.87516600	-1.91854800	2.25716000
H	-4.18381700	-2.94175400	2.03779800
H	-4.41200000	-1.59366900	3.15337800

	H	-2.79361100	1.14060400	-0.13753700
	H	-2.48472300	-0.10427400	3.72157300
	H	7.62074200	-1.52863000	-0.44334200
	H	-3.15656900	5.48602200	-2.44795700
	O	-4.37398700	0.89858200	2.34334800
	O	2.05538200	1.89061800	-2.55809500
	O	3.43712600	-3.02607900	2.31409500

5b (*in vacuo*)

Conf. 1	C	-0.23676000	3.40180000	1.60885900
	C	2.73775200	3.04268000	2.11697700
	C	2.11556900	3.35145300	0.76269200
	C	0.73375700	2.72266300	0.65048900
	H	-0.32548500	4.46446900	1.35904300
	H	-1.23676500	2.96920000	1.54869600
	H	3.73979300	3.47037200	2.20765900
	H	2.00918200	4.44680200	0.67782500
	N	2.73180300	2.87141500	-0.47754200
	H	0.81254600	1.66052100	0.94103400
	N	0.52184800	2.76721500	-0.80682300
	H	2.83430100	1.96088100	2.25071600
	C	-4.53936300	3.79041600	-1.22004700
	C	-2.20069900	3.97570100	-1.81538200
	C	-3.48720900	4.50790100	-1.78704300
	C	-1.95842300	2.71840100	-1.25818400
	C	-3.01415100	2.02702200	-0.66194900
	C	-4.31221500	2.53825500	-0.64425300
	H	-5.53990000	4.21234200	-1.21991600

H	-1.38793400	4.52473400	-2.27773400
C	-0.08478800	-1.15351300	3.49013200
C	-1.46843900	-1.19207000	3.33265700
C	-2.05331900	-2.11627300	2.46420700
H	0.35769400	-0.44250300	4.17930900
C	6.21866800	2.08967900	0.32242400
C	4.64114500	0.57708100	-0.68311700
C	5.01477300	1.88210200	-0.35248000
H	6.52238300	3.09715500	0.58975700
C	0.33075200	3.21161400	3.03497900
C	1.80114400	3.65360200	3.18470500
H	0.24659000	2.15139500	3.29921400
H	-0.29058900	3.75626600	3.75094900
H	1.85679200	4.74672700	3.11304500
H	2.16252900	3.39198400	4.18310700
C	-1.22181900	-3.00593800	1.77259800
C	0.73181700	-2.03665100	2.79317900
C	0.16748200	-2.98105300	1.92661800
H	-1.66700600	-3.74910500	1.11610300
H	1.80602200	-2.01926400	2.93318600
C	2.62708000	-1.66354100	-2.93305700
C	0.50145700	-3.57617900	-1.92334100
C	1.47870000	-2.82341600	-1.03010700
C	2.80109800	-2.61021600	-1.75512600
H	2.26159000	-0.68679400	-2.60405800
H	3.57152100	-1.49142500	-3.45679100
H	-0.45673700	-3.73807500	-1.42170700

H	1.04371300	-1.83837700	-0.79320500
N	1.95779200	-3.43448200	0.21225700
H	3.13052400	-3.58715700	-2.15230700
N	3.67310900	-2.27600100	-0.61835300
H	0.90997800	-4.56037000	-2.17814300
C	7.02798300	1.00743800	0.66217100
C	6.64356200	-0.28736400	0.32669700
C	5.44618100	-0.51383600	-0.35694100
H	7.95351300	1.17314400	1.20232400
C	1.60855500	-2.32098300	-3.89155300
C	0.29149500	-2.72402700	-3.19611200
H	2.06587100	-3.21181700	-4.33943600
H	1.39059700	-1.63624000	-4.71513900
H	-0.25554900	-1.81454400	-2.92074600
H	-0.34456600	-3.26603700	-3.90195800
H	3.70541600	0.41602000	-1.20248200
C	-4.18402600	-2.96598400	-0.79602600
C	-6.12407500	-0.85383300	-1.79447100
C	-5.02804000	-0.61633900	-0.76452900
C	-4.79729700	-1.87250300	0.06710300
H	-3.22504100	-2.63018500	-1.20458200
H	-3.99604700	-3.87782400	-0.22214400
H	-6.30980800	0.03991400	-2.39643200
H	-4.10079800	-0.38875700	-1.31309300
N	-5.20851400	0.38060100	0.29412400
H	-5.77569200	-2.23699800	0.42680900
N	-4.07794400	-1.31012400	1.21941800

H	-7.06216100	-1.11539000	-1.29316300
C	-5.19432800	-3.26328700	-1.92961100
C	-5.64214700	-2.00618000	-2.70606900
H	-6.07541500	-3.74624100	-1.49067900
H	-4.76329900	-3.98719300	-2.62676700
H	-4.80373400	-1.64191800	-3.31134700
H	-6.43391900	-2.27702200	-3.40980000
C	1.76368900	2.68238400	-1.42105200
C	-0.61127200	2.04094800	-1.37650100
H	-0.37227000	1.89028300	-2.43135700
H	-0.66632800	1.04847600	-0.90619800
C	4.14669400	3.05634800	-0.77416200
H	4.22717700	3.20887600	-1.85364900
H	4.49001400	3.97157100	-0.28040900
C	3.22753400	-2.97954400	0.48020000
C	5.08121000	-1.92564500	-0.79093400
H	5.69173100	-2.64119500	-0.23607500
H	5.31360000	-2.04460600	-1.85452200
C	1.05629600	-3.99227900	1.21726500
H	0.43681300	-4.74681800	0.72551900
H	1.69110200	-4.50448000	1.94252800
C	-4.54636700	-0.03188500	1.43036100
C	-5.46441600	1.78518200	0.01229000
H	-6.35459600	1.84972000	-0.61967000
H	-5.70946800	2.25286900	0.96870000
C	-3.56583300	-2.15731000	2.30588900
H	-3.88758600	-3.17987200	2.09370600

	H	-4.03994900	-1.84133700	3.23771800
	H	-2.80656400	1.07013100	-0.19900100
	H	-2.10291700	-0.50365400	3.87567900
	H	7.26646900	-1.12869800	0.61067100
	H	-3.67660500	5.48231400	-2.22389000
	S	2.03345500	2.39419300	-3.05597800
	S	4.11407700	-3.26666100	1.87544800
	S	-4.38339600	0.86811900	2.83420900
Conf. 2	C	-1.32034900	1.90574100	1.60079900
	C	1.49919500	1.83460300	2.71091300
	C	0.97057900	2.87883600	1.73693100
	C	-0.06920500	2.26774100	0.80737100
	H	-1.74975100	2.80826200	2.04908900
	H	-2.08660700	1.46554100	0.96180100
	H	2.27243100	2.24689700	3.36499500
	H	0.48638500	3.67641400	2.32787200
	N	1.85383900	3.51939200	0.76535900
	H	0.34907400	1.34340000	0.37529200
	N	-0.11659000	3.27748900	-0.26521400
	H	1.94636600	1.00043600	2.16205200
	C	-5.14132700	3.06511300	-1.23880700
	C	-3.01135800	4.19176900	-1.04481200
	C	-4.39946700	4.22132800	-0.98493000
	C	-2.34638400	3.00393600	-1.37329800
	C	-3.09852200	1.85899500	-1.62793800
	C	-4.49598300	1.87103500	-1.55467900
	H	-6.22546900	3.09680700	-1.18719200

H	-2.43406400	5.08747900	-0.84311600
C	-0.48644800	-3.20950400	1.97432000
C	-1.78197100	-3.49591700	1.55639000
C	-2.09370300	-3.53573600	0.19175000
H	-0.25728000	-3.16724000	3.03370300
C	5.27247500	2.86611800	1.94393000
C	4.24085800	2.07600800	-0.08031200
C	4.24988400	2.96251000	0.99873400
H	5.29577500	3.54868200	2.78808100
C	-0.89835700	0.89040200	2.68831600
C	0.29239700	1.35810100	3.54948600
H	-0.63573800	-0.05381800	2.19832900
H	-1.75212900	0.67298600	3.33772400
H	-0.03090700	2.18285900	4.19655200
H	0.60300600	0.54823600	4.21628500
C	-1.06799800	-3.32761600	-0.73361300
C	0.52143600	-2.98339200	1.03788300
C	0.24448200	-3.06418300	-0.32583400
H	-1.30516400	-3.35547200	-1.79158200
H	1.52393700	-2.74004400	1.36522300
C	6.20848100	-2.78170000	-0.56957400
C	3.74776100	-4.35454100	0.24336300
C	3.75589000	-3.21376100	-0.76459300
C	4.81716000	-2.18261100	-0.40173600
H	6.36380600	-3.07946000	-1.61228800
H	6.99257700	-2.06393700	-0.31477900
H	2.97364600	-5.09181900	0.01363500

H	4.01794800	-3.64004800	-1.74955000
N	2.58330900	-2.34904400	-0.95477100
H	4.68919800	-1.90057000	0.65644300
N	4.38956000	-1.06086600	-1.24132800
H	3.54586000	-3.97338900	1.24944200
C	6.26699000	1.89895500	1.80775300
C	6.24331400	1.01669700	0.73042400
C	5.22345500	1.09414100	-0.22447600
H	7.06234400	1.83502600	2.54227600
C	6.29950800	-4.00633300	0.37062300
C	5.14476500	-5.01297200	0.18670200
H	6.29995200	-3.64878800	1.40738400
H	7.25589000	-4.51486800	0.22061000
H	5.25639700	-5.51609700	-0.78111100
H	5.21675900	-5.79345600	0.94920700
H	3.45678600	2.14694900	-0.82289200
C	-5.49186700	-2.56839400	2.02330900
C	-5.71588600	0.38589300	1.37181900
C	-5.55971100	-0.72645200	0.34360800
C	-4.72197000	-1.86309900	0.91386000
H	-6.42770700	-2.97724600	1.62730900
H	-4.92709600	-3.40359200	2.44618000
H	-6.29747600	1.22228600	0.97744800
H	-6.56765000	-1.11724500	0.11585500
N	-4.87756500	-0.49773100	-0.93179600
H	-3.80564900	-1.43408300	1.35020100
N	-4.36203800	-2.57969500	-0.31276600

H	-4.73479500	0.78082000	1.65332100
C	-5.76834600	-1.51653500	3.12231200
C	-6.43815700	-0.23059700	2.59235200
H	-4.81539100	-1.25322100	3.59679800
H	-6.39134700	-1.95580400	3.90649900
H	-7.47206900	-0.45851000	2.30639700
H	-6.49790000	0.50714800	3.39731900
C	1.13338300	3.87866600	-0.34240800
C	-0.83965200	2.96272200	-1.50281000
H	-0.50590100	3.69265700	-2.24219500
H	-0.52674200	1.97171800	-1.85601500
C	3.17047800	4.02927200	1.10214700
H	3.38123600	4.84380000	0.40329000
H	3.15105200	4.45220000	2.11308900
C	3.03659200	-1.13214900	-1.44696700
C	5.18545900	0.15648500	-1.42125100
H	6.19895000	-0.15915000	-1.67976600
H	4.76601800	0.67234800	-2.28489600
C	1.31124000	-2.90950800	-1.39857200
H	0.92560800	-2.26652200	-2.19245700
H	1.51194600	-3.89217800	-1.84540300
C	-4.33862600	-1.68708500	-1.36489500
C	-5.26788400	0.59234200	-1.82298400
H	-6.34455600	0.76912100	-1.71650100
H	-5.08552200	0.24543100	-2.84265400
C	-3.52079000	-3.78183300	-0.27632000
H	-3.52601000	-4.19634600	-1.28358700

	H	-4.01281400	-4.49956000	0.38488800
	H	-2.59301900	0.93292900	-1.88371900
	H	-2.55244900	-3.69208800	2.29467500
	H	7.03172300	0.27790600	0.62505700
	H	-4.91052900	5.14615800	-0.74028200
	S	1.67740000	4.87803000	-1.57366100
	S	2.08659200	0.02382500	-2.20019600
	S	-3.77446000	-2.00171100	-2.91279500
Conf. 3	C	-1.00937200	2.22791700	2.20967200
	C	2.00551500	2.30545200	2.55393000
	C	1.21922200	3.13355200	1.54676500
	C	-0.00922100	2.37113800	1.06847300
	H	-1.33012000	3.21840800	2.55009900
	H	-1.90481100	1.68434300	1.90695400
	H	2.90781300	2.82614200	2.88748900
	H	0.88370600	4.05396500	2.05595000
	N	1.80779500	3.52593000	0.26571100
	H	0.30652200	1.36131900	0.75559800
	N	-0.35059600	3.12242100	-0.15194000
	H	2.32142700	1.36138700	2.09915500
	C	-5.49394700	3.26610700	-0.06304300
	C	-3.32539700	4.13193000	-0.69062700
	C	-4.66969800	4.33969200	-0.39309200
	C	-2.79541100	2.84039400	-0.65570400
	C	-3.62767100	1.77697100	-0.29989200
	C	-4.97752100	1.96923100	-0.00468000
	H	-6.54237800	3.44032800	0.16025300

H	-2.68703900	4.96549900	-0.96208800
C	0.00770400	-2.61923200	2.92794500
C	-1.35827500	-2.81353600	2.73926200
C	-1.85436900	-3.12563900	1.47231200
H	0.38357400	-2.36512900	3.91305300
C	5.49935500	3.28063500	0.45555000
C	3.99625000	2.00135400	-0.92137000
C	4.22681300	3.09993700	-0.09081400
H	5.69284600	4.12967400	1.10407300
C	-0.29832900	1.45193000	3.34114300
C	1.06185400	2.05254100	3.75241400
H	-0.14906700	0.41907600	3.00817300
H	-0.95526600	1.39887700	4.21358200
H	0.89495400	3.00472700	4.27091700
H	1.55187700	1.39195600	4.47389000
C	-0.95388100	-3.25727900	0.41172000
C	0.89173800	-2.74046300	1.85954900
C	0.41902100	-3.07319700	0.58912000
H	-1.32723900	-3.50064400	-0.57883800
H	1.94955000	-2.56316500	2.00741900
C	6.25948300	-2.59492000	-0.83901900
C	4.20348300	-4.05133900	0.84523700
C	3.88908000	-3.24164300	-0.40535500
C	4.85779000	-2.07466300	-0.54421300
H	6.25886100	-3.16293800	-1.77580500
H	6.97853700	-1.77998900	-0.95086000
H	3.50230000	-4.87991600	0.97869400

H	4.02551200	-3.90572000	-1.27772800
N	2.60146500	-2.55365300	-0.56666000
H	4.88863400	-1.52309600	0.40980700
N	4.14737700	-1.25699500	-1.52922400
H	4.13240300	-3.41757900	1.73517400
C	6.52520400	2.38527300	0.16305200
C	6.28556900	1.29822800	-0.67408500
C	5.01457000	1.09177800	-1.21980400
H	7.51302200	2.53777900	0.58386300
C	6.67316200	-3.49331600	0.34866500
C	5.64226900	-4.59296900	0.68068900
H	6.81440900	-2.85822900	1.23120900
H	7.64377100	-3.95304200	0.14261000
H	5.64479600	-5.34200600	-0.12011700
H	5.94657500	-5.11569900	1.59166100
H	3.01509500	1.85543200	-1.35543000
C	-4.07927700	-3.19816700	-1.92823900
C	-6.29806400	-1.13536600	-2.13125100
C	-5.16132400	-1.09991900	-1.11875500
C	-4.73927500	-2.51425500	-0.73907000
H	-3.19507700	-2.63435400	-2.24306300
H	-3.75285000	-4.21332600	-1.68381900
H	-6.61869400	-0.12751100	-2.40901600
H	-4.30491100	-0.60092100	-1.59745200
N	-5.36623400	-0.51501900	0.20925800
H	-5.64547100	-3.08870600	-0.47684600
N	-3.99498300	-2.25203500	0.50179400

H	-7.16512000	-1.65373600	-1.70719900
C	-5.13327700	-3.24905600	-3.05964800
C	-5.76481600	-1.87707600	-3.37887600
H	-5.92487100	-3.94885600	-2.76564400
H	-4.68011100	-3.66015400	-3.96585600
H	-5.01478700	-1.24261300	-3.86498700
H	-6.57264200	-2.00860300	-4.10404900
C	0.81494300	3.66509500	-0.66816900
C	-1.36130500	2.57223600	-1.05804500
H	-1.16433100	3.01233300	-2.03696700
H	-1.19813000	1.48979700	-1.14481400
C	3.12631300	4.12052100	0.15163400
H	3.08886400	4.81969000	-0.68921000
H	3.34117700	4.69866100	1.05737200
C	2.79851400	-1.47812400	-1.41884300
C	4.74165100	-0.07448800	-2.15711800
H	5.67350100	-0.39260700	-2.63172500
H	4.05285400	0.23086500	-2.94473100
C	1.35149700	-3.31056000	-0.58746300
H	0.82389900	-3.07287000	-1.51350700
H	1.61802100	-4.37416100	-0.62319200
C	-4.57305500	-1.16570500	1.12444200
C	-5.88769700	0.82427500	0.42281100
H	-6.84589000	0.90230900	-0.09918700
H	-6.09208300	0.90575600	1.49311100
C	-3.34680900	-3.32699300	1.26072500
H	-3.51494400	-4.25258300	0.70397000

	H	-3.84161000	-3.42264000	2.23001800
	H	-3.19994900	0.78317000	-0.24138200
	H	-2.04677700	-2.69512500	3.56657500
	H	7.09646700	0.61887000	-0.91694500
	H	-5.08150800	5.34241700	-0.42868200
	S	0.99054500	4.38173200	-2.17530200
	S	1.58435500	-0.63275700	-2.20653800
	S	-4.37909500	-0.73529500	2.73235800

9a (*in vacuo*)

Conf. 1	C	-0.53324600	0.55803800	8.87647700
	C	0.53324600	-0.55803800	8.87647700
	C	0.51554400	-1.42249900	7.59637700
	C	0.59625200	-0.47901300	6.40235700
	C	-0.59625200	0.47901300	6.40235700
	C	-0.51554400	1.42249900	7.59637700
	H	-0.39702400	1.19363500	9.75615900
	H	-1.52619700	0.10304400	8.97628400
	H	0.39702400	-1.19363500	9.75615900
	H	1.52619700	-0.10304400	8.97628400
	H	-0.40324300	-2.01625100	7.53866200
	H	1.35511800	-2.12386500	7.61265200
	H	1.51545000	0.12558200	6.50811500
	N	0.52047700	-1.01000100	5.04376300
	N	-0.52047700	1.01000100	5.04376300
	H	-1.51545000	-0.12558200	6.50811500
	H	-1.35511800	2.12386500	7.61265200
	H	0.40324300	2.01625100	7.53866200

C	-1.60062200	1.84962600	4.51628200
C	-1.18700100	2.74229700	3.36348100
C	-0.15967600	3.67557100	3.50014800
C	-1.84363900	2.65466500	2.13498600
C	-1.47520500	3.46303800	1.06759500
C	-0.43245100	4.38630200	1.19231600
C	0.21280700	4.48409300	2.42770700
C	1.60062200	-1.84962600	4.51628200
C	1.18700100	-2.74229700	3.36348100
C	0.15967600	-3.67557100	3.50014800
C	-0.21280700	-4.48409300	2.42770700
C	0.43245100	-4.38630200	1.19231600
C	1.47520500	-3.46303800	1.06759500
C	1.84363900	-2.65466500	2.13498600
C	0.00000000	-5.22411500	0.00000000
C	-0.43245100	-4.38630200	-1.19231600
C	0.00000000	5.22411500	0.00000000
C	0.43245100	4.38630200	-1.19231600
C	-0.21280700	4.48409300	-2.42770700
C	0.15967600	3.67557100	-3.50014800
C	1.18700100	2.74229700	-3.36348100
C	1.84363900	2.65466500	-2.13498600
C	1.47520500	3.46303800	-1.06759500
C	-1.47520500	-3.46303800	-1.06759500
C	0.21280700	-4.48409300	-2.42770700
C	-0.15967600	-3.67557100	-3.50014800
C	-1.18700100	-2.74229700	-3.36348100

C	-1.84363900	-2.65466500	-2.13498600
C	-1.60062200	-1.84962600	-4.51628200
N	-0.52047700	-1.01000100	-5.04376300
N	0.52047700	1.01000100	-5.04376300
C	0.59625200	0.47901300	-6.40235700
C	-0.59625200	-0.47901300	-6.40235700
C	0.51554400	1.42249900	-7.59637700
C	-0.51554400	-1.42249900	-7.59637700
C	-0.53324600	-0.55803800	-8.87647700
H	-1.94503000	2.47024100	5.34850000
H	-2.45099200	1.22847200	4.20307900
H	0.37235900	3.75351000	4.44269600
H	-2.62811700	1.91782400	2.00180600
H	-1.97698400	3.34789400	0.11272700
H	1.02501200	5.19409500	2.55110800
H	1.94503000	-2.47024100	5.34850000
H	2.45099200	-1.22847200	4.20307900
H	-0.37235900	-3.75351000	4.44269600
H	-1.02501200	-5.19409500	2.55110800
H	1.97698400	-3.34789400	0.11272700
H	2.62811700	-1.91782400	2.00180600
H	-0.82116600	-5.88118400	0.30626000
H	0.82116600	-5.88118400	-0.30626000
H	-0.82116600	5.88118400	-0.30626000
H	0.82116600	5.88118400	0.30626000
H	-1.02501200	5.19409500	-2.55110800
H	-0.37235900	3.75351000	-4.44269600

	H	2.62811700	1.91782400	-2.00180600
	H	1.97698400	3.34789400	-0.11272700
	H	-1.97698400	-3.34789400	-0.11272700
	H	1.02501200	-5.19409500	-2.55110800
	H	0.37235900	-3.75351000	-4.44269600
	H	-2.62811700	-1.91782400	-2.00180600
	H	-2.45099200	-1.22847200	-4.20307900
	H	-1.94503000	-2.47024100	-5.34850000
	C	1.60062200	1.84962600	-4.51628200
	H	1.51545000	-0.12558200	-6.50811500
	H	-1.51545000	0.12558200	-6.50811500
	H	-1.35511800	-2.12386500	-7.61265200
	H	0.40324300	-2.01625100	-7.53866200
	C	0.53324600	0.55803800	-8.87647700
	H	1.35511800	2.12386500	-7.61265200
	H	-0.40324300	2.01625100	-7.53866200
	H	-1.52619700	-0.10304400	-8.97628400
	H	-0.39702400	-1.19363500	-9.75615900
	H	1.94503000	2.47024100	-5.34850000
	H	2.45099200	1.22847200	-4.20307900
	H	1.52619700	0.10304400	-8.97628400
	H	0.39702400	1.19363500	-9.75615900
	C	0.00000000	0.00000000	4.21961100
	C	0.00000000	0.00000000	-4.21961100
	O	0.00000000	0.00000000	-3.00968200
	O	0.00000000	0.00000000	3.00968200
Conf. 2	C	8.39953400	-0.73469200	0.97963400

C	8.10158200	-1.95789300	0.08656800
C	6.61092900	-2.36452600	0.07992400
C	5.80197100	-1.12118600	-0.26615400
C	6.03831900	-0.02625000	0.77399200
C	7.47590800	0.47285100	0.69904100
H	9.44484000	-0.43770700	0.85606500
H	8.28387700	-1.02363300	2.03125600
H	8.71621100	-2.80310200	0.40901100
H	8.40595900	-1.73086600	-0.94245100
H	6.30250200	-2.74778700	1.05824400
H	6.44566800	-3.16512000	-0.64723100
H	6.15738600	-0.74463200	-1.24207800
N	4.34255200	-1.17724000	-0.28124000
N	4.92525300	0.87797800	0.48404600
H	5.88608600	-0.46962200	1.77477900
H	7.66808300	1.26167300	1.43224700
H	7.67308500	0.89431900	-0.29269900
C	4.67815500	2.02851500	1.35838200
C	3.65967900	3.00450700	0.80658900
C	3.75835600	3.49916900	-0.49339100
C	2.57830800	3.40624900	1.59116800
C	1.58846200	4.23167100	1.07175300
C	1.64420700	4.67092300	-0.25402100
C	2.75933300	4.31448200	-1.01695900
C	3.62997900	-1.94603100	-1.30658500
C	2.57059400	-2.91045300	-0.80695900
C	1.74713500	-2.60075200	0.28045600

C	0.67737800	-3.42171700	0.61190500
C	0.38435100	-4.57143300	-0.13073900
C	1.23241100	-4.89974300	-1.18860200
C	2.31384100	-4.08240200	-1.51883800
C	-0.85776400	-5.38683900	0.17718900
C	-2.14825400	-4.59699000	0.00711400
C	0.48701800	5.43609200	-0.85922900
C	-0.78906600	4.61475500	-1.02536300
C	-2.03481400	5.24731100	-1.02003600
C	-3.21180300	4.51920000	-1.18566400
C	-3.18006500	3.13482600	-1.36053100
C	-1.93488600	2.50092400	-1.37223700
C	-0.75929900	3.22651900	-1.20672700
C	-3.13976500	-4.61272800	0.98852200
C	-2.37227900	-3.82773300	-1.14238500
C	-3.53834800	-3.08948700	-1.29753200
C	-4.52530600	-3.09259800	-0.30244100
C	-4.31296000	-3.87249700	0.83456900
C	-5.77355000	-2.24097100	-0.45690100
N	-5.56290400	-0.80871200	-0.23923300
N	-4.55510700	1.15255200	-0.72663800
C	-4.97988000	1.22396400	0.67018900
C	-5.06899300	-0.26228300	1.02139500
C	-4.14229400	1.96246000	1.70755200
C	-5.87310600	-0.45417000	2.30111500
C	-5.14063900	0.31212900	3.42542100
H	5.64157100	2.53132900	1.48842300

H	4.35486700	1.69318300	2.35469000
H	4.59167600	3.19821800	-1.11885300
H	2.48560200	3.03282400	2.60586300
H	0.73510400	4.49694400	1.68761000
H	2.83098200	4.65353100	-2.04571500
H	4.37511600	-2.49740000	-1.88565200
H	3.14525700	-1.23824100	-1.99083300
H	1.91475100	-1.68527200	0.83132800
H	0.03071800	-3.14263800	1.43768400
H	1.03962000	-5.79366100	-1.77354900
H	2.94567900	-4.35127100	-2.36065600
H	-0.81076600	-5.76734700	1.20301200
H	-0.87940900	-6.26714300	-0.47511200
H	0.78909800	5.82576900	-1.83828700
H	0.25702700	6.31283100	-0.24378000
H	-2.08913400	6.32209100	-0.87361800
H	-4.16687600	5.03614600	-1.16606000
H	-1.88836400	1.42452200	-1.49493900
H	0.18882000	2.70121600	-1.19731500
H	-2.99413800	-5.20412400	1.88733300
H	-1.61431700	-3.79489700	-1.91740900
H	-3.68919300	-2.49006400	-2.18854100
H	-5.06896400	-3.90320100	1.61368000
H	-6.54666900	-2.58068700	0.23707400
H	-6.17134300	-2.33313000	-1.46875600
C	-4.46016500	2.34531500	-1.55901100
H	-6.00129600	1.64645000	0.70144800

	H	-4.04736600	-0.63479600	1.20191200
	H	-5.95615100	-1.51178500	2.56771100
	H	-6.89051200	-0.07067700	2.16477100
	C	-4.84593100	1.78504700	3.07183500
	H	-4.05142000	3.02422500	1.46665500
	H	-3.12828200	1.55094000	1.73267500
	H	-4.19425200	-0.20095200	3.63464400
	H	-5.72621600	0.26654500	4.34843000
	H	-5.32545300	3.00039700	-1.37935800
	H	-4.53077600	1.99265900	-2.59145900
	H	-5.79038500	2.34308100	3.05298100
	H	-4.23925400	2.23555200	3.86277400
	C	3.85500600	0.09278100	0.03674400
	C	-5.08708400	-0.01617000	-1.28063900
	O	-5.12815900	-0.29053400	-2.46693800
	O	2.69630400	0.43958400	-0.07645900
Conf. 3	C	-1.18913600	2.70908800	3.78878300
	C	0.32490100	2.95181200	3.61388300
	C	0.76678700	3.05381800	2.13644000
	C	-0.11905100	4.10152800	1.47371100
	C	-1.58803300	3.68200600	1.55264900
	C	-2.06640400	3.70639800	2.99884000
	H	-1.44607600	2.74832200	4.85138000
	H	-1.42873600	1.69421500	3.44959600
	H	0.88240800	2.15396000	4.11313500
	H	0.59793100	3.88318300	4.12507000
	H	0.65709900	2.09236100	1.62435800

H	1.82322600	3.32806500	2.07846000
H	0.00067600	5.04619100	2.03487600
N	0.00295600	4.39159300	0.04802900
N	-2.18293500	4.58434800	0.56950700
H	-1.66803400	2.64532800	1.18624900
H	-3.11824300	3.41732700	3.07947100
H	-1.97464200	4.71881100	3.40733300
C	-3.59571600	4.50689700	0.18817100
C	-4.00582900	3.17788400	-0.42213900
C	-3.70774200	2.88900500	-1.75805300
C	-4.66401500	2.20329000	0.33386500
C	-4.99435300	0.96865200	-0.21790100
C	-4.68396500	0.67162400	-1.54804400
C	-4.04738900	1.65649200	-2.30968100
C	1.23127100	4.89494100	-0.54808300
C	2.21090200	3.80154700	-0.93244300
C	3.56610500	3.90299800	-0.62497900
C	4.46475400	2.90653400	-1.01093500
C	4.03194000	1.78126900	-1.71232800
C	2.66871300	1.68454600	-2.02127700
C	1.77489100	2.67587000	-1.64051800
C	5.00536400	0.69090300	-2.13539000
C	4.68396500	-0.67162400	-1.54804400
C	-5.00536400	-0.69090300	-2.13539000
C	-4.03194000	-1.78126900	-1.71232800
C	-4.46475400	-2.90653400	-1.01093500
C	-3.56610500	-3.90299800	-0.62497900

C	-2.21090200	-3.80154700	-0.93244300
C	-1.77489100	-2.67587000	-1.64051800
C	-2.66871300	-1.68454600	-2.02127700
C	4.99435300	-0.96865200	-0.21790100
C	4.04738900	-1.65649200	-2.30968100
C	3.70774200	-2.88900500	-1.75805300
C	4.00582900	-3.17788400	-0.42213900
C	4.66401500	-2.20329000	0.33386500
C	3.59571600	-4.50689700	0.18817100
N	2.18293500	-4.58434800	0.56950700
N	-0.00295600	-4.39159300	0.04802900
C	0.11905100	-4.10152800	1.47371100
C	1.58803300	-3.68200600	1.55264900
C	-0.76678700	-3.05381800	2.13644000
C	2.06640400	-3.70639800	2.99884000
C	1.18913600	-2.70908800	3.78878300
H	-3.76002800	5.31724900	-0.52318000
H	-4.19796400	4.71638200	1.07560200
H	-3.20249000	3.63582800	-2.36011600
H	-4.92820100	2.41339200	1.36615800
H	-5.49701900	0.22557400	0.39318800
H	-3.81774700	1.45940700	-3.35220100
H	1.71802900	5.60705400	0.13266600
H	0.92530400	5.45228100	-1.43799600
H	3.92966300	4.76636500	-0.07547700
H	5.51565000	3.00888500	-0.75869700
H	2.30298700	0.81785900	-2.56175600

H	0.72305200	2.58013200	-1.88526500
H	6.01684200	0.98748100	-1.84275500
H	5.00807500	0.61505100	-3.22778200
H	-5.00807500	-0.61505100	-3.22778200
H	-6.01684200	-0.98748100	-1.84275500
H	-5.51565000	-3.00888500	-0.75869700
H	-3.92966300	-4.76636500	-0.07547700
H	-0.72305200	-2.58013200	-1.88526500
H	-2.30298700	-0.81785900	-2.56175600
H	5.49701900	-0.22557400	0.39318800
H	3.81774700	-1.45940700	-3.35220100
H	3.20249000	-3.63582800	-2.36011600
H	4.92820100	-2.41339200	1.36615800
H	4.19796400	-4.71638200	1.07560200
H	3.76002800	-5.31724900	-0.52318000
C	-1.23127100	-4.89494100	-0.54808300
H	-0.00067600	-5.04619100	2.03487600
H	1.66803400	-2.64532800	1.18624900
H	3.11824300	-3.41732700	3.07947100
H	1.97464200	-4.71881100	3.40733300
C	-0.32490100	-2.95181200	3.61388300
H	-1.82322600	-3.32806500	2.07846000
H	-0.65709900	-2.09236100	1.62435800
H	1.42873600	-1.69421500	3.44959600
H	1.44607600	-2.74832200	4.85138000
H	-1.71802900	-5.60705400	0.13266600
H	-0.92530400	-5.45228100	-1.43799600

	H	-0.59793100	-3.88318300	4.12507000
	H	-0.88240800	-2.15396000	4.11313500
	C	-1.23127100	4.84575100	-0.41578100
	C	1.23127100	-4.84575100	-0.41578100
	O	1.43973600	-5.37448300	-1.49347800
	O	-1.43973600	5.37448300	-1.49347800
Conf. 4	C	-2.68969300	5.75895500	-0.78265500
	C	-2.65282000	5.78698600	0.76017400
	C	-1.54552800	4.89747400	1.36973100
	C	-0.23394300	5.28980900	0.70048200
	C	-0.32314500	5.05296400	-0.80849000
	C	-1.31467000	6.02400700	-1.43643600
	H	-3.41990300	6.48904800	-1.14366400
	H	-3.04664900	4.77646400	-1.11389800
	H	-3.62813700	5.48612300	1.15334200
	H	-2.49191000	6.81957700	1.09393300
	H	-1.75109100	3.83615400	1.19626300
	H	-1.50428900	5.04355300	2.45192200
	H	-0.08126900	6.37142900	0.87066500
	N	1.00950900	4.59216700	1.01774500
	N	1.08878700	5.10302200	-1.17539700
	H	-0.70101200	4.03065400	-0.97446700
	H	-1.38640600	5.87959300	-2.51799500
	H	-0.99070700	7.05631900	-1.26325600
	C	1.57138300	4.91929500	-2.54083000
	C	1.12931500	3.63351500	-3.21906200
	C	1.65796700	2.39440800	-2.83322400

C	0.19725000	3.65741200	-4.25632800
C	-0.18858800	2.48343400	-4.90125900
C	0.34984600	1.24954000	-4.53255100
C	1.27441600	1.22427900	-3.47986200
C	1.60240200	4.62687200	2.35291600
C	1.20522200	3.44743500	3.21760900
C	0.38837500	3.59722900	4.33609600
C	0.00000000	2.49009800	5.09208100
C	0.42964400	1.20591200	4.75730300
C	1.27737800	1.06329800	3.65023600
C	1.65411100	2.16265100	2.89117800
C	0.00000000	0.00000000	5.57525200
C	-0.42964400	-1.20591200	4.75730300
C	0.00000000	0.00000000	-5.32093800
C	-0.34984600	-1.24954000	-4.53255100
C	-1.27441600	-1.22427900	-3.47986200
C	-1.65796700	-2.39440800	-2.83322400
C	-1.12931500	-3.63351500	-3.21906200
C	-0.19725000	-3.65741200	-4.25632800
C	0.18858800	-2.48343400	-4.90125900
C	-1.27737800	-1.06329800	3.65023600
C	0.00000000	-2.49009800	5.09208100
C	-0.38837500	-3.59722900	4.33609600
C	-1.20522200	-3.44743500	3.21760900
C	-1.65411100	-2.16265100	2.89117800
C	-1.60240200	-4.62687200	2.35291600
N	-1.00950900	-4.59216700	1.01774500

N	-1.08878700	-5.10302200	-1.17539700
C	0.32314500	-5.05296400	-0.80849000
C	0.23394300	-5.28980900	0.70048200
C	1.31467000	-6.02400700	-1.43643600
C	1.54552800	-4.89747400	1.36973100
C	2.65282000	-5.78698600	0.76017400
H	2.66035100	4.95416800	-2.47438300
H	1.24854000	5.77564500	-3.13933400
H	2.38783300	2.35755200	-2.03137900
H	-0.22377900	4.60547600	-4.57732400
H	-0.90778000	2.53094400	-5.71307500
H	1.70605200	0.27788800	-3.17154200
H	1.32743800	5.56645300	2.84922700
H	2.68506000	4.63126700	2.20630200
H	0.03795600	4.58570900	4.61767400
H	-0.65364300	2.62948000	5.94762500
H	1.62787700	0.07575700	3.37093900
H	2.29923000	2.03133300	2.02875700
H	-0.82050400	0.30118000	6.23684800
H	0.82050400	-0.30118000	6.23684800
H	0.83927100	-0.23811400	-5.98473800
H	-0.83927100	0.23811400	-5.98473800
H	-1.70605200	-0.27788800	-3.17154200
H	-2.38783300	-2.35755200	-2.03137900
H	0.22377900	-4.60547600	-4.57732400
H	0.90778000	-2.53094400	-5.71307500
H	-1.62787700	-0.07575700	3.37093900

	H	0.65364300	-2.62948000	5.94762500
	H	-0.03795600	-4.58570900	4.61767400
	H	-2.29923000	-2.03133300	2.02875700
	H	-2.68506000	-4.63126700	2.20630200
	H	-1.32743800	-5.56645300	2.84922700
	C	-1.57138300	-4.91929500	-2.54083000
	H	0.70101200	-4.03065400	-0.97446700
	H	0.08126900	-6.37142900	0.87066500
	H	1.50428900	-5.04355300	2.45192200
	H	1.75109100	-3.83615400	1.19626300
	C	2.68969300	-5.75895500	-0.78265500
	H	1.38640600	-5.87959300	-2.51799500
	H	0.99070700	-7.05631900	-1.26325600
	H	2.49191000	-6.81957700	1.09393300
	H	3.62813700	-5.48612300	1.15334200
	H	-2.66035100	-4.95416800	-2.47438300
	H	-1.24854000	-5.77564500	-3.13933400
	H	3.04664900	-4.77646400	-1.11389800
	H	3.41990300	-6.48904800	-1.14366400
	C	1.84266600	4.64457500	-0.09995300
	C	-1.84266600	-4.64457500	-0.09995300
	O	-3.02353800	-4.34175300	-0.12509300
	O	3.02353800	4.34175300	-0.12509300

9a (ACN)

Conf. 1	C	-0.52902900	0.56203600	8.74816400
	C	0.52902900	-0.56203600	8.74816400
	C	0.50533400	-1.42805500	7.46851100

C	0.59358800	-0.48057100	6.27954600
C	-0.59358800	0.48057100	6.27954600
C	-0.50533400	1.42805500	7.46851100
H	-0.38554700	1.19785700	9.62607100
H	-1.52559200	0.11577700	8.84760300
H	0.38554700	-1.19785700	9.62607100
H	1.52559200	-0.11577700	8.84760300
H	-0.41851100	-2.01365400	7.41165200
H	1.34132000	-2.13293000	7.48431000
H	1.51661400	0.11467000	6.38427300
N	0.52256000	-1.00501500	4.91179200
N	-0.52256000	1.00501500	4.91179200
H	-1.51661400	-0.11467000	6.38427300
H	-1.34132000	2.13293000	7.48431000
H	0.41851100	2.01365400	7.41165200
C	-1.59702300	1.85371700	4.39336600
C	-1.17468900	2.79615800	3.28262600
C	0.02624800	3.50602600	3.33284400
C	-2.01778400	2.99908100	2.18682800
C	-1.65700300	3.86042700	1.15505700
C	-0.43639500	4.54318800	1.18159200
C	0.38993700	4.36383700	2.29580800
C	1.59702300	-1.85371700	4.39336600
C	1.17468900	-2.79615800	3.28262600
C	-0.02624800	-3.50602600	3.33284400
C	-0.38993700	-4.36383700	2.29580800
C	0.43639500	-4.54318800	1.18159200

C	1.65700300	-3.86042700	1.15505700
C	2.01778400	-2.99908100	2.18682800
C	0.00000000	-5.39604400	0.00000000
C	-0.43639500	-4.54318800	-1.18159200
C	0.00000000	5.39604400	0.00000000
C	0.43639500	4.54318800	-1.18159200
C	-0.38993700	4.36383700	-2.29580800
C	-0.02624800	3.50602600	-3.33284400
C	1.17468900	2.79615800	-3.28262600
C	2.01778400	2.99908100	-2.18682800
C	1.65700300	3.86042700	-1.15505700
C	-1.65700300	-3.86042700	-1.15505700
C	0.38993700	-4.36383700	-2.29580800
C	0.02624800	-3.50602600	-3.33284400
C	-1.17468900	-2.79615800	-3.28262600
C	-2.01778400	-2.99908100	-2.18682800
C	-1.59702300	-1.85371700	-4.39336600
N	-0.52256000	-1.00501500	-4.91179200
N	0.52256000	1.00501500	-4.91179200
C	0.59358800	0.48057100	-6.27954600
C	-0.59358800	-0.48057100	-6.27954600
C	0.50533400	1.42805500	-7.46851100
C	-0.50533400	-1.42805500	-7.46851100
C	-0.52902900	-0.56203600	-8.74816400
H	-1.96433900	2.43936700	5.24047200
H	-2.43759900	1.24036100	4.04403100
H	0.69401400	3.36579700	4.17549900

H	-2.95828700	2.46056300	2.12844200
H	-2.32201000	3.98246700	0.30615100
H	1.33617800	4.89283700	2.34954100
H	1.96433900	-2.43936700	5.24047200
H	2.43759900	-1.24036100	4.04403100
H	-0.69401400	-3.36579700	4.17549900
H	-1.33617800	-4.89283700	2.34954100
H	2.32201000	-3.98246700	0.30615100
H	2.95828700	-2.46056300	2.12844200
H	-0.82284900	-6.04650700	0.31000900
H	0.82284900	-6.04650700	-0.31000900
H	-0.82284900	6.04650700	-0.31000900
H	0.82284900	6.04650700	0.31000900
H	-1.33617800	4.89283700	-2.34954100
H	-0.69401400	3.36579700	-4.17549900
H	2.95828700	2.46056300	-2.12844200
H	2.32201000	3.98246700	-0.30615100
H	-2.32201000	-3.98246700	-0.30615100
H	1.33617800	-4.89283700	-2.34954100
H	0.69401400	-3.36579700	-4.17549900
H	-2.95828700	-2.46056300	-2.12844200
H	-2.43759900	-1.24036100	-4.04403100
H	-1.96433900	-2.43936700	-5.24047200
C	1.59702300	1.85371700	-4.39336600
H	1.51661400	-0.11467000	-6.38427300
H	-1.51661400	0.11467000	-6.38427300
H	-1.34132000	-2.13293000	-7.48431000

	H	0.41851100	-2.01365400	-7.41165200
	C	0.52902900	0.56203600	-8.74816400
	H	1.34132000	2.13293000	-7.48431000
	H	-0.41851100	2.01365400	-7.41165200
	H	-1.52559200	-0.11577700	-8.84760300
	H	-0.38554700	-1.19785700	-9.62607100
	H	1.96433900	2.43936700	-5.24047200
	H	2.43759900	1.24036100	-4.04403100
	H	1.52559200	0.11577700	-8.84760300
	H	0.38554700	1.19785700	-9.62607100
	C	0.00000000	0.00000000	4.09806200
	C	0.00000000	0.00000000	-4.09806200
	O	0.00000000	0.00000000	-2.87749600
	O	0.00000000	0.00000000	2.87749600
Conf. 2	C	8.35793900	0.13155200	1.37330400
	C	8.39572600	-1.10700200	0.45446300
	C	7.03004100	-1.81878800	0.32864200
	C	6.00838400	-0.76549700	-0.07814900
	C	5.92670200	0.33414600	0.97721200
	C	7.22773900	1.12515400	1.01924100
	H	9.32302600	0.64409900	1.33522400
	H	8.22098400	-0.19561900	2.41079700
	H	9.14612300	-1.81190200	0.82281000
	H	8.72245800	-0.80077600	-0.54633300
	H	6.73847400	-2.27362400	1.28133600
	H	7.09790700	-2.62101500	-0.41154700
	H	6.34659200	-0.30701400	-1.02285100

N	4.59247000	-1.12649500	-0.21195100
N	4.67413300	0.99114200	0.58744300
H	5.78509200	-0.14243400	1.96209000
H	7.19018600	1.92238800	1.76671800
H	7.41190800	1.59350100	0.04636100
C	4.11696900	2.05992400	1.41701600
C	3.12840400	2.95633400	0.69699400
C	3.34450300	3.39822000	-0.60894500
C	1.96275400	3.36559100	1.35013400
C	1.01691400	4.15173400	0.70090000
C	1.20045300	4.54638800	-0.62894800
C	2.38973900	4.17543100	-1.26334300
C	4.17581100	-2.01263800	-1.29629300
C	2.96660400	-2.88549500	-1.01148200
C	2.50836300	-3.16425900	0.27704800
C	1.39646400	-3.98134800	0.47510300
C	0.71784000	-4.55801500	-0.60372000
C	1.20104600	-4.30156300	-1.89114700
C	2.30013500	-3.47095000	-2.09151800
C	-0.51783100	-5.41245200	-0.38705000
C	-1.81872800	-4.62402600	-0.30051600
C	0.10566100	5.29794000	-1.36750600
C	-1.21245800	4.53858800	-1.38924000
C	-2.39749000	5.12497600	-0.94324000
C	-3.59098900	4.39886400	-0.91044400
C	-3.62972700	3.06676500	-1.31923300
C	-2.44785700	2.48531200	-1.79508300

C	-1.26263300	3.20796500	-1.83041800
C	-2.65304100	-4.73889500	0.81315600
C	-2.23327300	-3.78818000	-1.34690100
C	-3.43804300	-3.09786800	-1.28144200
C	-4.27576300	-3.21698100	-0.16357700
C	-3.86425000	-4.04717900	0.87994600
C	-5.59661800	-2.46687400	-0.09912500
N	-5.47729500	-1.02459000	0.11740000
N	-4.77806300	1.03660000	-0.45921900
C	-4.95177200	1.03901500	0.99678100
C	-4.83380500	-0.45410400	1.30170900
C	-4.01722200	1.83565100	1.89844300
C	-5.38258200	-0.75533400	2.68998900
C	-4.54024500	0.05752300	3.69924700
H	4.96555800	2.65762600	1.76100700
H	3.63857500	1.64383000	2.31358400
H	4.24346400	3.09558700	-1.13402800
H	1.77686700	3.03834700	2.36816600
H	0.10515800	4.42697300	1.22090000
H	2.56230800	4.47724700	-2.29168400
H	5.02910300	-2.66040800	-1.51459100
H	3.98274200	-1.43239200	-2.20821900
H	3.01457100	-2.72567900	1.12844100
H	1.04912000	-4.16974600	1.48627600
H	0.71040100	-4.75049300	-2.74872800
H	2.64188000	-3.27467000	-3.10336600
H	-0.40215400	-5.99550300	0.53012700

H	-0.59769200	-6.13331900	-1.20780600
H	0.43786900	5.49306500	-2.39241400
H	-0.05815800	6.27585200	-0.90365000
H	-2.38914900	6.15358200	-0.59693400
H	-4.49435700	4.87342800	-0.54005300
H	-2.45615400	1.45057200	-2.12086400
H	-0.35549300	2.72694600	-2.18078000
H	-2.35985900	-5.38112200	1.63745700
H	-1.60945200	-3.67972300	-2.22733200
H	-3.74152900	-2.46271600	-2.10641900
H	-4.49668900	-4.16594500	1.75418800
H	-6.21633400	-2.87920000	0.70007500
H	-6.14376700	-2.58861700	-1.03526900
C	-4.91033000	2.25695900	-1.25055700
H	-5.98496100	1.35836900	1.21736300
H	-3.76591900	-0.72524200	1.29060100
H	-5.31593200	-1.82069200	2.92619900
H	-6.43924900	-0.47124000	2.74540700
C	-4.44947200	1.55910400	3.35645700
H	-4.07577600	2.90628300	1.68994400
H	-2.98005300	1.52570600	1.73389400
H	-3.52909800	-0.36559000	3.72672000
H	-4.95305600	-0.06307000	4.70470600
H	-5.71990500	2.87727600	-0.84557600
H	-5.20997900	1.93884800	-2.25179600
H	-5.42865000	2.02402600	3.52288500
H	-3.75449000	2.04813100	4.04472400

	C	3.83743000	0.02065100	0.04044400
	C	-5.27593900	-0.16519100	-0.95154600
	O	-5.49988500	-0.41866900	-2.13081500
	O	2.64557000	0.14399200	-0.19347400
Conf. 3	C	0.06582700	2.92970900	3.78745100
	C	1.51537000	2.41181300	3.67556600
	C	2.01485000	2.28462500	2.21790500
	C	1.76967200	3.62844800	1.54482800
	C	0.27868400	3.96396000	1.55675800
	C	-0.19030800	4.22392400	2.98210800
	H	-0.18199000	3.09501300	4.83960800
	H	-0.61981200	2.15479600	3.42494900
	H	1.59593800	1.44391500	4.17798900
	H	2.18237900	3.09660500	4.21273200
	H	1.47473500	1.49309100	1.68793100
	H	3.07544300	2.02067300	2.20834500
	H	2.30454900	4.39844300	2.12676400
	N	2.08360500	3.82867100	0.12819200
	N	0.23061500	5.03464800	0.55784000
	H	-0.27347300	3.09267600	1.16976300
	H	-1.25325100	4.47723000	3.01723100
	H	0.36257300	5.06673100	3.41085500
	C	-1.03210100	5.64337600	0.12791600
	C	-2.01485000	4.66654900	-0.49630600
	C	-1.83898600	4.21817800	-1.81043500
	C	-3.10524100	4.17486300	0.22825200
	C	-3.97999200	3.24707000	-0.33306100

C	-3.79500000	2.78403700	-1.63979900
C	-2.71753600	3.29447500	-2.37214800
C	3.43801800	3.68830200	-0.39155500
C	3.76959400	2.27988200	-0.85023300
C	4.95317100	1.65377100	-0.46110500
C	5.26557400	0.36857100	-0.91060100
C	4.40175200	-0.32504900	-1.75984500
C	3.21690000	0.31105100	-2.15566400
C	2.90603900	1.58962100	-1.70956000
C	4.72135400	-1.73547500	-2.23246600
C	3.79500000	-2.78403700	-1.63979900
C	-4.72135400	1.73547500	-2.23246600
C	-4.40175200	0.32504900	-1.75984500
C	-5.26557400	-0.36857100	-0.91060100
C	-4.95317100	-1.65377100	-0.46110500
C	-3.76959400	-2.27988200	-0.85023300
C	-2.90603900	-1.58962100	-1.70956000
C	-3.21690000	-0.31105100	-2.15566400
C	3.97999200	-3.24707000	-0.33306100
C	2.71753600	-3.29447500	-2.37214800
C	1.83898600	-4.21817800	-1.81043500
C	2.01485000	-4.66654900	-0.49630600
C	3.10524100	-4.17486300	0.22825200
C	1.03210100	-5.64337600	0.12791600
N	-0.23061500	-5.03464800	0.55784000
N	-2.08360500	-3.82867100	0.12819200
C	-1.76967200	-3.62844800	1.54482800

C	-0.27868400	-3.96396000	1.55675800
C	-2.01485000	-2.28462500	2.21790500
C	0.19030800	-4.22392400	2.98210800
C	-0.06582700	-2.92970900	3.78745100
H	-0.76771200	6.42400500	-0.58637900
H	-1.48555500	6.12829300	0.99480500
H	-1.00618500	4.59579900	-2.39297200
H	-3.27748900	4.52326200	1.24171800
H	-4.81512800	2.87780300	0.25352400
H	-2.56529400	2.96957000	-3.39631200
H	4.16114000	4.01211300	0.36654600
H	3.51712300	4.37977900	-1.23445600
H	5.63990700	2.16834200	0.20385400
H	6.19137300	-0.09826900	-0.59024400
H	2.52895400	-0.20270900	-2.81918500
H	1.98061200	2.05909700	-2.02484600
H	5.75624600	-1.97152100	-1.97081700
H	4.65261900	-1.77561300	-3.32366900
H	-4.65261900	1.77561300	-3.32366900
H	-5.75624600	1.97152100	-1.97081700
H	-6.19137300	0.09826900	-0.59024400
H	-5.63990700	-2.16834200	0.20385400
H	-1.98061200	-2.05909700	-2.02484600
H	-2.52895400	0.20270900	-2.81918500
H	4.81512800	-2.87780300	0.25352400
H	2.56529400	-2.96957000	-3.39631200
H	1.00618500	-4.59579900	-2.39297200

	H	3.27748900	-4.52326200	1.24171800
	H	1.48555500	-6.12829300	0.99480500
	H	0.76771200	-6.42400500	-0.58637900
	C	-3.43801800	-3.68830200	-0.39155500
	H	-2.30454900	-4.39844300	2.12676400
	H	0.27347300	-3.09267600	1.16976300
	H	1.25325100	-4.47723000	3.01723100
	H	-0.36257300	-5.06673100	3.41085500
	C	-1.51537000	-2.41181300	3.67556600
	H	-3.07544300	-2.02067300	2.20834500
	H	-1.47473500	-1.49309100	1.68793100
	H	0.61981200	-2.15479600	3.42494900
	H	0.18199000	-3.09501300	4.83960800
	H	-4.16114000	-4.01211300	0.36654600
	H	-3.51712300	-4.37977900	-1.23445600
	H	-2.18237900	-3.09660500	4.21273200
	H	-1.59593800	-1.44391500	4.17798900
	C	1.23981800	4.81235300	-0.36996800
	C	-1.23981800	-4.81235300	-0.36996800
	O	-1.36663800	-5.38721800	-1.44602400
	O	1.36663800	5.38721800	-1.44602400
Conf. 4	C	-2.54607000	5.82968100	-0.82829000
	C	-2.52964100	5.85812800	0.71447600
	C	-1.45699600	4.93731200	1.34044800
	C	-0.12774600	5.29323600	0.68748700
	C	-0.20359000	5.05530500	-0.82052300
	C	-1.15514800	6.05580700	-1.46339100

H	-3.24763000	6.58202700	-1.19922200
H	-2.92636200	4.85798800	-1.16492400
H	-3.51755700	5.58356000	1.09448100
H	-2.34240300	6.88496300	1.05073400
H	-1.69307800	3.88272700	1.16265500
H	-1.42637900	5.08799500	2.42201000
H	0.05482700	6.36771000	0.86075500
N	1.09877100	4.56352300	1.02359600
N	1.21757500	5.05798700	-1.16892900
H	-0.60747800	4.04452900	-0.99205300
H	-1.21627300	5.91529200	-2.54562900
H	-0.80435900	7.07759800	-1.28231600
C	1.70629600	4.86673700	-2.53257000
C	1.23371700	3.59445600	-3.21734100
C	1.72464600	2.33841600	-2.83537300
C	0.30794600	3.64927200	-4.26023700
C	-0.10984700	2.48830500	-4.91029400
C	0.38979200	1.23663700	-4.54328500
C	1.31122800	1.18091700	-3.48818600
C	1.66934300	4.60240300	2.37101200
C	1.25477000	3.42670400	3.23394500
C	0.40363600	3.58534100	4.32674000
C	0.00000000	2.48477500	5.08567400
C	0.44662700	1.19798300	4.78056500
C	1.32771800	1.04620400	3.70025100
C	1.72043100	2.13945100	2.93834600
C	0.00000000	0.00000000	5.60108200

C	-0.44662700	-1.19798300	4.78056500
C	0.00000000	0.00000000	-5.33349500
C	-0.38979200	-1.23663700	-4.54328500
C	-1.31122800	-1.18091700	-3.48818600
C	-1.72464600	-2.33841600	-2.83537300
C	-1.23371700	-3.59445600	-3.21734100
C	-0.30794600	-3.64927200	-4.26023700
C	0.10984700	-2.48830500	-4.91029400
C	-1.32771800	-1.04620400	3.70025100
C	0.00000000	-2.48477500	5.08567400
C	-0.40363600	-3.58534100	4.32674000
C	-1.25477000	-3.42670400	3.23394500
C	-1.72043100	-2.13945100	2.93834600
C	-1.66934300	-4.60240300	2.37101200
N	-1.09877100	-4.56352300	1.02359600
N	-1.21757500	-5.05798700	-1.16892900
C	0.20359000	-5.05530500	-0.82052300
C	0.12774600	-5.29323600	0.68748700
C	1.15514800	-6.05580700	-1.46339100
C	1.45699600	-4.93731200	1.34044800
C	2.52964100	-5.85812800	0.71447600
H	2.79535100	4.87524000	-2.46463500
H	1.40731600	5.73201600	-3.12895100
H	2.44841700	2.27254800	-2.02989700
H	-0.08589400	4.60904800	-4.57913500
H	-0.82551700	2.56025500	-5.72302300
H	1.71814200	0.22282100	-3.18227200

H	1.38348600	5.54286500	2.85555000
H	2.75458000	4.60726400	2.24967000
H	0.03823200	4.57415400	4.58501900
H	-0.67919000	2.63182500	5.91937600
H	1.69685300	0.05862900	3.44525700
H	2.39330700	1.99621100	2.09911400
H	-0.81601000	0.31368700	6.26133900
H	0.81601000	-0.31368700	6.26133900
H	0.83095900	-0.26600000	-5.99629300
H	-0.83095900	0.26600000	-5.99629300
H	-1.71814200	-0.22282100	-3.18227200
H	-2.44841700	-2.27254800	-2.02989700
H	0.08589400	-4.60904800	-4.57913500
H	0.82551700	-2.56025500	-5.72302300
H	-1.69685300	-0.05862900	3.44525700
H	0.67919000	-2.63182500	5.91937600
H	-0.03823200	-4.57415400	4.58501900
H	-2.39330700	-1.99621100	2.09911400
H	-2.75458000	-4.60726400	2.24967000
H	-1.38348600	-5.54286500	2.85555000
C	-1.70629600	-4.86673700	-2.53257000
H	0.60747800	-4.04452900	-0.99205300
H	-0.05482700	-6.36771000	0.86075500
H	1.42637900	-5.08799500	2.42201000
H	1.69307800	-3.88272700	1.16265500
C	2.54607000	-5.82968100	-0.82829000
H	1.21627300	-5.91529200	-2.54562900

	H	0.80435900	-7.07759800	-1.28231600
	H	2.34240300	-6.88496300	1.05073400
	H	3.51755700	-5.58356000	1.09448100
	H	-2.79535100	-4.87524000	-2.46463500
	H	-1.40731600	-5.73201600	-3.12895100
	H	2.92636200	-4.85798800	-1.16492400
	H	3.24763000	-6.58202700	-1.19922200
	C	1.94256200	4.60065900	-0.08130000
	C	-1.94256200	-4.60065900	-0.08130000
	O	-3.12857900	-4.28543500	-0.08649500
	O	3.12857900	4.28543500	-0.08649500

9b (*in vacuo*)

Conf. 1	C	-0.56425200	0.52628700	8.43517400
	C	0.56425200	-0.52628700	8.43517400
	C	0.59525700	-1.39215100	7.15550300
	C	0.61989700	-0.44254300	5.96454900
	C	-0.61989700	0.44254300	5.96454900
	C	-0.59525700	1.39215100	7.15550300
	H	-0.46528000	1.16891900	9.31458200
	H	-1.52963300	0.01543600	8.53393800
	H	0.46528000	-1.16891900	9.31458200
	H	1.52963300	-0.01543600	8.53393800
	H	-0.28790200	-2.03773100	7.09981000
	H	1.47335800	-2.04397200	7.17194700
	H	1.50577200	0.21030800	6.06483300
	N	0.57690800	-0.96612500	4.59619000
	N	-0.57690800	0.96612500	4.59619000

H	-1.50577200	-0.21030800	6.06483300
H	-1.47335800	2.04397200	7.17194700
H	0.28790200	2.03773100	7.09981000
C	-1.66352000	1.81611300	4.10279900
C	-1.24891600	2.88625100	3.10938900
C	-0.07425300	3.62240900	3.25262300
C	-2.07045800	3.15505800	2.01305200
C	-1.69707200	4.08423400	1.05176000
C	-0.48593800	4.77494600	1.15251800
C	0.30241400	4.55107700	2.28391700
C	1.66352000	-1.81611300	4.10279900
C	1.24891600	-2.88625100	3.10938900
C	0.07425300	-3.62240900	3.25262300
C	-0.30241400	-4.55107700	2.28391700
C	0.48593800	-4.77494600	1.15251800
C	1.69707200	-4.08423400	1.05176000
C	2.07045800	-3.15505800	2.01305200
C	0.00000000	-5.64175600	0.00000000
C	-0.48593800	-4.77494600	-1.15251800
C	0.00000000	5.64175600	0.00000000
C	0.48593800	4.77494600	-1.15251800
C	-0.30241400	4.55107700	-2.28391700
C	0.07425300	3.62240900	-3.25262300
C	1.24891600	2.88625100	-3.10938900
C	2.07045800	3.15505800	-2.01305200
C	1.69707200	4.08423400	-1.05176000
C	-1.69707200	-4.08423400	-1.05176000

C	0.30241400	-4.55107700	-2.28391700
C	-0.07425300	-3.62240900	-3.25262300
C	-1.24891600	-2.88625100	-3.10938900
C	-2.07045800	-3.15505800	-2.01305200
C	-1.66352000	-1.81611300	-4.10279900
N	-0.57690800	-0.96612500	-4.59619000
N	0.57690800	0.96612500	-4.59619000
C	0.61989700	0.44254300	-5.96454900
C	-0.61989700	-0.44254300	-5.96454900
C	0.59525700	1.39215100	-7.15550300
C	-0.59525700	-1.39215100	-7.15550300
C	-0.56425200	-0.52628700	-8.43517400
H	-2.10349400	2.28982000	4.98554200
H	-2.44092900	1.19395400	3.64268500
H	0.57890600	3.42909700	4.09643900
H	-2.98625100	2.58838900	1.87980700
H	-2.32887700	4.23453900	0.18260400
H	1.24036000	5.08641300	2.39598400
H	2.10349400	-2.28982000	4.98554200
H	2.44092900	-1.19395400	3.64268500
H	-0.57890600	-3.42909700	4.09643900
H	-1.24036000	-5.08641300	2.39598400
H	2.32887700	-4.23453900	0.18260400
H	2.98625100	-2.58838900	1.87980700
H	-0.80828000	-6.29247500	0.34726100
H	0.80828000	-6.29247500	-0.34726100
H	-0.80828000	6.29247500	-0.34726100

H	0.80828000	6.29247500	0.34726100
H	-1.24036000	5.08641300	-2.39598400
H	-0.57890600	3.42909700	-4.09643900
H	2.98625100	2.58838900	-1.87980700
H	2.32887700	4.23453900	-0.18260400
H	-2.32887700	-4.23453900	-0.18260400
H	1.24036000	-5.08641300	-2.39598400
H	0.57890600	-3.42909700	-4.09643900
H	-2.98625100	-2.58838900	-1.87980700
H	-2.44092900	-1.19395400	-3.64268500
H	-2.10349400	-2.28982000	-4.98554200
C	1.66352000	1.81611300	-4.10279900
H	1.50577200	-0.21030800	-6.06483300
H	-1.50577200	0.21030800	-6.06483300
H	-1.47335800	-2.04397200	-7.17194700
H	0.28790200	-2.03773100	-7.09981000
C	0.56425200	0.52628700	-8.43517400
H	1.47335800	2.04397200	-7.17194700
H	-0.28790200	2.03773100	-7.09981000
H	-1.52963300	-0.01543600	-8.53393800
H	-0.46528000	-1.16891900	-9.31458200
H	2.10349400	2.28982000	-4.98554200
H	2.44092900	1.19395400	-3.64268500
H	1.52963300	0.01543600	-8.53393800
H	0.46528000	1.16891900	-9.31458200
C	0.00000000	0.00000000	3.78169200
C	0.00000000	0.00000000	-3.78169200

	S	0.00000000	0.00000000	-2.12355200
	S	0.00000000	0.00000000	2.12355200
Conf. 2	C	8.24785100	0.21509000	1.14681300
	C	8.29207500	-0.98912100	0.18377900
	C	6.94397300	-1.73604300	0.07438200
	C	5.88077800	-0.69911100	-0.26262800
	C	5.79904400	0.35537100	0.83151800
	C	7.07651100	1.18503900	0.86643300
	H	9.19563000	0.75816200	1.09720200
	H	8.15701100	-0.15234300	2.17602500
	H	9.07468300	-1.68310900	0.50250700
	H	8.57915600	-0.63857100	-0.81479900
	H	6.69439400	-2.23342000	1.01758000
	H	7.01183600	-2.51148900	-0.69367900
	H	6.17635400	-0.19643400	-1.20072900
	N	4.47097800	-1.08834400	-0.37022700
	N	4.51057100	0.98077800	0.50950800
	H	5.70145600	-0.16404500	1.80161300
	H	7.04058500	1.95239500	1.64462500
	H	7.21456800	1.69811100	-0.09134800
	C	3.98437500	2.02620900	1.39030800
	C	3.07712400	3.04364800	0.72526200
	C	3.27104700	3.47965400	-0.58306400
	C	1.99215900	3.55289600	1.44235300
	C	1.07589800	4.40284600	0.83964000
	C	1.21063300	4.76552100	-0.50473800
	C	2.34293500	4.32322700	-1.19124000

C	4.06039700	-2.03191600	-1.40922400
C	2.99433000	-3.04184000	-1.01938700
C	2.61121400	-3.28225800	0.29862800
C	1.55639500	-4.14454100	0.58238100
C	0.86640200	-4.80567600	-0.43724000
C	1.30912500	-4.62331000	-1.75040800
C	2.35050100	-3.74672900	-2.03746700
C	-0.37929900	-5.61453100	-0.14004100
C	-1.65314500	-4.77514300	-0.07171400
C	0.09198800	5.51559900	-1.20546600
C	-1.20903900	4.72225200	-1.20936700
C	-2.40773300	5.28868200	-0.77588000
C	-3.57977900	4.53173000	-0.71660000
C	-3.58350800	3.18849800	-1.08890800
C	-2.38949000	2.62723300	-1.55564500
C	-1.22441900	3.37967100	-1.61243800
C	-2.61411300	-5.01600300	0.91147700
C	-1.91110300	-3.76176300	-1.00369900
C	-3.08611600	-3.02207900	-0.95811000
C	-4.04679400	-3.26304700	0.03121300
C	-3.79222800	-4.26987500	0.96311500
C	-5.33431800	-2.45841800	0.07726900
N	-5.14008500	-1.02058000	0.24918900
N	-4.64376600	1.08174900	-0.31037600
C	-4.60159100	1.02054300	1.15588800
C	-4.37261500	-0.46946800	1.36937900
C	-3.59359500	1.83310000	1.95899100

C	-4.71095500	-0.86174300	2.80097400
C	-3.77471500	-0.04830900	3.72371100
H	4.85759300	2.53466000	1.80940600
H	3.44231100	1.56764700	2.22762000
H	4.10871700	3.09900600	-1.15625900
H	1.82828300	3.23170300	2.46631800
H	0.20895400	4.73834100	1.39939800
H	2.47958200	4.60325200	-2.23116800
H	4.96506200	-2.56608700	-1.71387800
H	3.70169900	-1.47498100	-2.28364500
H	3.10335400	-2.74842400	1.10202500
H	1.23983300	-4.27611700	1.61235400
H	0.81149600	-5.14538800	-2.56172300
H	2.64075900	-3.58520200	-3.07160500
H	-0.26100300	-6.14832700	0.80742600
H	-0.50422500	-6.38247800	-0.91262100
H	0.39578400	5.73728000	-2.23427700
H	-0.08339200	6.48110600	-0.71947600
H	-2.42645300	6.32694100	-0.45870500
H	-4.49471600	4.99207200	-0.35517100
H	-2.37267300	1.58420300	-1.85138700
H	-0.30449300	2.90916900	-1.94167400
H	-2.44195700	-5.79284400	1.65026500
H	-1.17894600	-3.53883400	-1.77048700
H	-3.26613800	-2.25179100	-1.69982200
H	-4.52150700	-4.47891500	1.74027200
H	-5.96289500	-2.81839700	0.89678900

	H	-5.89617900	-2.57787700	-0.85105200
	C	-4.84428400	2.35149000	-1.00229500
	H	-5.61180900	1.27312200	1.52673000
	H	-3.30708100	-0.69000500	1.19779500
	H	-4.55934100	-1.93096800	2.96901300
	H	-5.76195000	-0.63736400	3.01495100
	C	-3.80691100	1.46930100	3.44655000
	H	-3.73288700	2.90630600	1.81373100
	H	-2.57482800	1.59513000	1.63808200
	H	-2.75083200	-0.41489800	3.58639300
	H	-4.03258300	-0.23526900	4.77015300
	H	-5.63770700	2.92063600	-0.49863100
	H	-5.20806500	2.10007000	-2.00136900
	H	-4.77538700	1.87105800	3.76942200
	H	-3.04907700	1.96713500	4.05817200
	C	3.68992700	0.01194500	-0.05045700
	C	-5.15157800	-0.11299100	-0.78177600
	S	2.05158700	0.13091700	-0.30673400
	S	-5.69164800	-0.40235100	-2.34593100
Conf. 3	C	2.21134000	1.71426300	3.71509800
	C	2.80656700	0.29104900	3.66179400
	C	3.12338600	-0.19288500	2.22783900
	C	3.98599400	0.88290800	1.58176300
	C	3.23467400	2.20552400	1.52421500
	C	3.03628400	2.75960800	2.92902900
	H	2.11161500	2.03022000	4.75742500
	H	1.19689600	1.69291000	3.29963100

H	2.11923200	-0.40977800	4.14414400
H	3.73160700	0.26807100	4.25052900
H	2.20532000	-0.33716600	1.64957400
H	3.63675900	-1.15673300	2.26162000
H	4.88323200	1.01960800	2.21174600
N	4.43677200	0.76753300	0.19224400
N	4.04640700	2.93531600	0.54537600
H	2.24095500	2.02569100	1.08334100
H	2.50407400	3.71392700	2.91568000
H	4.00750200	2.93080300	3.40599700
C	3.72378000	4.31400200	0.16723100
C	2.32659000	4.48131400	-0.40612200
C	2.02711100	4.04645800	-1.70207900
C	1.30236200	5.06151000	0.34757200
C	0.01206600	5.18005700	-0.16305900
C	-0.29391300	4.72897400	-1.44949100
C	0.73874200	4.17418200	-2.21246000
C	5.16893100	-0.40343500	-0.27181900
C	4.26439400	-1.54694100	-0.68914000
C	4.54009600	-2.85993200	-0.31179100
C	3.72378000	-3.91167300	-0.73124100
C	2.60837100	-3.67807200	-1.53585400
C	2.33840300	-2.35725500	-1.91633600
C	3.15143000	-1.31032100	-1.50375700
C	1.70960800	-4.81875600	-1.99108300
C	0.29391300	-4.72897400	-1.44949100
C	-1.70960800	4.81875600	-1.99108300

C	-2.60837100	3.67807200	-1.53585400
C	-3.72378000	3.91167300	-0.73124100
C	-4.54009600	2.85993200	-0.31179100
C	-4.26439400	1.54694100	-0.68914000
C	-3.15143000	1.31032100	-1.50375700
C	-2.33840300	2.35725500	-1.91633600
C	-0.01206600	-5.18005700	-0.16305900
C	-0.73874200	-4.17418200	-2.21246000
C	-2.02711100	-4.04645800	-1.70207900
C	-2.32659000	-4.48131400	-0.40612200
C	-1.30236200	-5.06151000	0.34757200
C	-3.72378000	-4.31400200	0.16723100
N	-4.04640700	-2.93531600	0.54537600
N	-4.43677200	-0.76753300	0.19224400
C	-3.98599400	-0.88290800	1.58176300
C	-3.23467400	-2.20552400	1.52421500
C	-3.12338600	0.19288500	2.22783900
C	-3.03628400	-2.75960800	2.92902900
C	-2.21134000	-1.71426300	3.71509800
H	4.48149900	4.61728500	-0.55574200
H	3.83767200	4.93896200	1.05665200
H	2.81134600	3.61256300	-2.31139900
H	1.51573000	5.43329300	1.34531200
H	-0.76591900	5.62840500	0.44682200
H	0.53596400	3.84474100	-3.22660300
H	5.85812800	-0.73621700	0.51506300
H	5.77454700	-0.07304800	-1.11976000

H	5.40013800	-3.06986000	0.31723800
H	3.96043800	-4.92564600	-0.42448800
H	1.47876400	-2.14543900	-2.54328900
H	2.92594200	-0.29560400	-1.81150300
H	2.15814900	-5.76765900	-1.68414300
H	1.67170500	-4.83115000	-3.08516900
H	-1.67170500	4.83115000	-3.08516900
H	-2.15814900	5.76765900	-1.68414300
H	-3.96043800	4.92564600	-0.42448800
H	-5.40013800	3.06986000	0.31723800
H	-2.92594200	0.29560400	-1.81150300
H	-1.47876400	2.14543900	-2.54328900
H	0.76591900	-5.62840500	0.44682200
H	-0.53596400	-3.84474100	-3.22660300
H	-2.81134600	-3.61256300	-2.31139900
H	-1.51573000	-5.43329300	1.34531200
H	-3.83767200	-4.93896200	1.05665200
H	-4.48149900	-4.61728500	-0.55574200
C	-5.16893100	0.40343500	-0.27181900
H	-4.88323200	-1.01960800	2.21174600
H	-2.24095500	-2.02569100	1.08334100
H	-2.50407400	-3.71392700	2.91568000
H	-4.00750200	-2.93080300	3.40599700
C	-2.80656700	-0.29104900	3.66179400
H	-3.63675900	1.15673300	2.26162000
H	-2.20532000	0.33716600	1.64957400
H	-1.19689600	-1.69291000	3.29963100

	H	-2.11161500	-2.03022000	4.75742500
	H	-5.85812800	0.73621700	0.51506300
	H	-5.77454700	0.07304800	-1.11976000
	H	-3.73160700	-0.26807100	4.25052900
	H	-2.11923200	0.40977800	4.14414400
	C	4.62883900	2.03372100	-0.31618000
	C	-4.62883900	-2.03372100	-0.31618000
	S	5.44377500	2.40443300	-1.73667300
	S	-5.44377500	-2.40443300	-1.73667300
Conf. 4	C	-3.18760600	5.15184300	-0.83223100
	C	-3.18206600	5.26757100	0.70687200
	C	-1.95737300	4.60041300	1.37488600
	C	-0.71961600	5.16445000	0.69008200
	C	-0.74289800	4.83430000	-0.79569000
	C	-1.86171200	5.59762200	-1.49148100
	H	-4.01757400	5.73619800	-1.23924400
	H	-3.37849500	4.10907200	-1.11160600
	H	-4.10214700	4.83316100	1.10760600
	H	-3.19569500	6.32823200	0.98603600
	H	-1.98810200	3.51260800	1.25699000
	H	-1.95818300	4.80851200	2.44704500
	H	-0.74085900	6.26337300	0.80342200
	N	0.62703700	4.70976600	1.05115800
	N	0.65865100	5.07802600	-1.14761700
	H	-0.94301300	3.75698900	-0.91190500
	H	-1.89086800	5.38007200	-2.56182400
	H	-1.71051600	6.67657500	-1.37601800

C	1.13629000	4.99893600	-2.52641700
C	0.81816300	3.69125200	-3.23008200
C	1.40867500	2.48647600	-2.82612600
C	-0.05983500	3.66317700	-4.31384100
C	-0.34090300	2.47127600	-4.97958500
C	0.25236500	1.27064600	-4.58627100
C	1.13021500	1.29925200	-3.49465400
C	1.11246300	4.81548600	2.42653000
C	0.82665600	3.58132400	3.25789000
C	0.00000000	3.62932400	4.37867000
C	-0.27004800	2.47762100	5.11867500
C	0.29074600	1.24970200	4.76640900
C	1.14389300	1.21137000	3.65513200
C	1.40425700	2.35420700	2.91086600
C	0.00000000	0.00000000	5.57941800
C	-0.29074600	-1.24970200	4.76640900
C	0.00000000	0.00000000	-5.37806700
C	-0.25236500	-1.27064600	-4.58627100
C	-1.13021500	-1.29925200	-3.49465400
C	-1.40867500	-2.48647600	-2.82612600
C	-0.81816300	-3.69125200	-3.23008200
C	0.05983500	-3.66317700	-4.31384100
C	0.34090300	-2.47127600	-4.97958500
C	-1.14389300	-1.21137000	3.65513200
C	0.27004800	-2.47762100	5.11867500
C	0.00000000	-3.62932400	4.37867000
C	-0.82665600	-3.58132400	3.25789000

C	-1.40425700	-2.35420700	2.91086600
C	-1.11246300	-4.81548600	2.42653000
N	-0.62703700	-4.70976600	1.05115800
N	-0.65865100	-5.07802600	-1.14761700
C	0.74289800	-4.83430000	-0.79569000
C	0.71961600	-5.16445000	0.69008200
C	1.86171200	-5.59762200	-1.49148100
C	1.95737300	-4.60041300	1.37488600
C	3.18206600	-5.26757100	0.70687200
H	2.21437800	5.16146300	-2.48222200
H	0.69612300	5.82882000	-3.08628200
H	2.10195100	2.48755400	-1.99206600
H	-0.52512400	4.58432000	-4.65144700
H	-1.02543100	2.47871100	-5.82204600
H	1.60504600	0.38034800	-3.16787200
H	0.65683900	5.69768200	2.89252400
H	2.18898800	4.98799200	2.36540800
H	-0.45038000	4.57176400	4.67573100
H	-0.93214200	2.53709500	5.97694800
H	1.59569500	0.26989600	3.36251100
H	2.05909900	2.30273500	2.04766700
H	-0.84925300	0.20555600	6.24130700
H	0.84925300	-0.20555600	6.24130700
H	0.85519500	-0.17342900	-6.04169100
H	-0.85519500	0.17342900	-6.04169100
H	-1.60504600	-0.38034800	-3.16787200
H	-2.10195100	-2.48755400	-1.99206600

H	0.52512400	-4.58432000	-4.65144700
H	1.02543100	-2.47871100	-5.82204600
H	-1.59569500	-0.26989600	3.36251100
H	0.93214200	-2.53709500	5.97694800
H	0.45038000	-4.57176400	4.67573100
H	-2.05909900	-2.30273500	2.04766700
H	-2.18898800	-4.98799200	2.36540800
H	-0.65683900	-5.69768200	2.89252400
C	-1.13629000	-4.99893600	-2.52641700
H	0.94301300	-3.75698900	-0.91190500
H	0.74085900	-6.26337300	0.80342200
H	1.95818300	-4.80851200	2.44704500
H	1.98810200	-3.51260800	1.25699000
C	3.18760600	-5.15184300	-0.83223100
H	1.89086800	-5.38007200	-2.56182400
H	1.71051600	-6.67657500	-1.37601800
H	3.19569500	-6.32823200	0.98603600
H	4.10214700	-4.83316100	1.10760600
H	-2.21437800	-5.16146300	-2.48222200
H	-0.69612300	-5.82882000	-3.08628200
H	3.37849500	-4.10907200	-1.11160600
H	4.01757400	-5.73619800	-1.23924400
C	1.44779600	4.84226100	-0.04788400
C	-1.44779600	-4.84226100	-0.04788400
S	3.12506100	4.73552400	-0.03864500
S	-3.12506100	-4.73552400	-0.03864500

9b (DCM)

Conf. 1	C	-0.55273500	0.53846700	8.39311700
	C	0.55273500	-0.53846700	8.39311700
	C	0.56724300	-1.40594800	7.11345700
	C	0.61231500	-0.45109500	5.92859200
	C	-0.61231500	0.45109500	5.92859200
	C	-0.56724300	1.40594800	7.11345700
	H	-0.43650800	1.18070500	9.27017000
	H	-1.52918100	0.05014200	8.49298600
	H	0.43650800	-1.18070500	9.27017000
	H	1.52918100	-0.05014200	8.49298600
	H	-0.32944800	-2.03197800	7.05669100
	H	1.43372100	-2.07232100	7.13015900
	H	1.50993000	0.18189000	6.02962700
	N	0.57236100	-0.96503000	4.54967100
	N	-0.57236100	0.96503000	4.54967100
	H	-1.50993000	-0.18189000	6.02962700
	H	-1.43372100	2.07232100	7.13015900
	H	0.32944800	2.03197800	7.05669100
	C	-1.64573300	1.84036700	4.07107900
	C	-1.22309100	2.92905000	3.10081700
	C	0.01529500	3.56635800	3.17372800
	C	-2.10835600	3.31684600	2.09205600
	C	-1.73875600	4.25910100	1.13879300
	C	-0.46765700	4.84161300	1.15879800
	C	0.38786500	4.50575100	2.21296400
	C	1.64573300	-1.84036700	4.07107900
	C	1.22309100	-2.92905000	3.10081700

C	-0.01529500	-3.56635800	3.17372800
C	-0.38786500	-4.50575100	2.21296400
C	0.46765700	-4.84161300	1.15879800
C	1.73875600	-4.25910100	1.13879300
C	2.10835600	-3.31684600	2.09205600
C	0.00000000	-5.71130700	0.00000000
C	-0.46765700	-4.84161300	-1.15879800
C	0.00000000	5.71130700	0.00000000
C	0.46765700	4.84161300	-1.15879800
C	-0.38786500	4.50575100	-2.21296400
C	-0.01529500	3.56635800	-3.17372800
C	1.22309100	2.92905000	-3.10081700
C	2.10835600	3.31684600	-2.09205600
C	1.73875600	4.25910100	-1.13879300
C	-1.73875600	-4.25910100	-1.13879300
C	0.38786500	-4.50575100	-2.21296400
C	0.01529500	-3.56635800	-3.17372800
C	-1.22309100	-2.92905000	-3.10081700
C	-2.10835600	-3.31684600	-2.09205600
C	-1.64573300	-1.84036700	-4.07107900
N	-0.57236100	-0.96503000	-4.54967100
N	0.57236100	0.96503000	-4.54967100
C	0.61231500	0.45109500	-5.92859200
C	-0.61231500	-0.45109500	-5.92859200
C	0.56724300	1.40594800	-7.11345700
C	-0.56724300	-1.40594800	-7.11345700
C	-0.55273500	-0.53846700	-8.39311700

H	-2.08106200	2.29833800	4.96304200
H	-2.43222200	1.23814400	3.60232000
H	0.71566200	3.28895200	3.95359900
H	-3.08210000	2.84223800	2.02235200
H	-2.43215100	4.50927200	0.34246600
H	1.37306400	4.95810400	2.26757500
H	2.08106200	-2.29833800	4.96304200
H	2.43222200	-1.23814400	3.60232000
H	-0.71566200	-3.28895200	3.95359900
H	-1.37306400	-4.95810400	2.26757500
H	2.43215100	-4.50927200	0.34246600
H	3.08210000	-2.84223800	2.02235200
H	-0.81586700	-6.35932100	0.33213100
H	0.81586700	-6.35932100	-0.33213100
H	-0.81586700	6.35932100	-0.33213100
H	0.81586700	6.35932100	0.33213100
H	-1.37306400	4.95810400	-2.26757500
H	-0.71566200	3.28895200	-3.95359900
H	3.08210000	2.84223800	-2.02235200
H	2.43215100	4.50927200	-0.34246600
H	-2.43215100	-4.50927200	-0.34246600
H	1.37306400	-4.95810400	-2.26757500
H	0.71566200	-3.28895200	-3.95359900
H	-3.08210000	-2.84223800	-2.02235200
H	-2.43222200	-1.23814400	-3.60232000
H	-2.08106200	-2.29833800	-4.96304200
C	1.64573300	1.84036700	-4.07107900

	H	1.50993000	-0.18189000	-6.02962700
	H	-1.50993000	0.18189000	-6.02962700
	H	-1.43372100	-2.07232100	-7.13015900
	H	0.32944800	-2.03197800	-7.05669100
	C	0.55273500	0.53846700	-8.39311700
	H	1.43372100	2.07232100	-7.13015900
	H	-0.32944800	2.03197800	-7.05669100
	H	-1.52918100	-0.05014200	-8.49298600
	H	-0.43650800	-1.18070500	-9.27017000
	H	2.08106200	2.29833800	-4.96304200
	H	2.43222200	1.23814400	-3.60232000
	H	1.52918100	0.05014200	-8.49298600
	H	0.43650800	1.18070500	-9.27017000
	C	0.00000000	0.00000000	3.74891100
	C	0.00000000	0.00000000	-3.74891100
	S	0.00000000	0.00000000	-2.07165500
	S	0.00000000	0.00000000	2.07165500
Conf. 2	C	-8.21238900	-0.51664700	1.24150400
	C	-8.35068500	0.68475100	0.28363700
	C	-7.05479900	1.51688400	0.15107900
	C	-5.93894100	0.54547000	-0.20708100
	C	-5.77146400	-0.49865300	0.88422500
	C	-6.98849800	-1.41212600	0.93707400
	H	-9.12391900	-1.11911700	1.20568300
	H	-8.12506500	-0.14712500	2.26996500
	H	-9.16693700	1.32865800	0.62165300
	H	-8.63380900	0.32069300	-0.71087700

H	-6.82039100	2.02666400	1.09143400
H	-7.18671400	2.28460700	-0.61587400
H	-6.21626900	0.02966700	-1.14161700
N	-4.55157800	1.01899100	-0.34738200
N	-4.44247500	-1.03588500	0.54319500
H	-5.68731900	0.02012300	1.85376200
H	-6.89000800	-2.17514500	1.71354400
H	-7.11147700	-1.92891600	-0.02050500
C	-3.84339600	-2.06261100	1.39855000
C	-2.93607400	-3.05557700	0.69610100
C	-3.12966200	-3.45439200	-0.62492300
C	-1.84831000	-3.58441300	1.39725000
C	-0.93042500	-4.41587600	0.76902100
C	-1.06535700	-4.74006400	-0.58614000
C	-2.19945800	-4.27891300	-1.25886700
C	-4.23438800	2.00168700	-1.38436600
C	-3.16894400	3.02463600	-1.03273400
C	-2.84167100	3.36707800	0.27946700
C	-1.78448600	4.23516200	0.54206700
C	-1.03934500	4.80445800	-0.49592900
C	-1.43320300	4.52998100	-1.80949100
C	-2.47545000	3.64603300	-2.07319500
C	0.20645400	5.61697800	-0.21197400
C	1.48619100	4.78615700	-0.11875300
C	0.04965500	-5.47812200	-1.30847600
C	1.35296600	-4.69047300	-1.28014200
C	2.52123700	-5.23145500	-0.74203200

C	3.68648600	-4.46721100	-0.63965800
C	3.71299800	-3.14192900	-1.07151900
C	2.55291700	-2.60826100	-1.64592600
C	1.39585000	-3.36915600	-1.74847600
C	2.49532100	5.14274600	0.78005400
C	1.70988400	3.67590400	-0.94256100
C	2.89902400	2.95736500	-0.87824700
C	3.90913500	3.31961700	0.01899000
C	3.68671300	4.41972900	0.84995600
C	5.22386300	2.55859800	0.07075600
N	5.08341100	1.11619600	0.25000800
N	4.72503400	-1.02059000	-0.26244600
C	4.68853800	-0.93716700	1.20828000
C	4.37201300	0.53852500	1.39965200
C	3.73934500	-1.79427500	2.03359900
C	4.71009600	0.97752200	2.81688200
C	3.83732700	0.12433700	3.76657700
H	-4.67943000	-2.59601900	1.85769200
H	-3.28248100	-1.58615800	2.21149100
H	-3.97237800	-3.06554100	-1.18500900
H	-1.68807900	-3.29871300	2.43225000
H	-0.06701000	-4.77099400	1.32217500
H	-2.33873200	-4.53116900	-2.30544000
H	-5.17072600	2.51977000	-1.60657200
H	-3.93093300	1.48313900	-2.30137700
H	-3.37793300	2.90694800	1.10095100
H	-1.51318700	4.44653600	1.57171400

H	-0.89525500	4.98161600	-2.63701700
H	-2.72520900	3.40921600	-3.10292000
H	0.08482000	6.17290900	0.72163000
H	0.33676100	6.36626800	-1.00181800
H	-0.25322900	-5.66228900	-2.34419200
H	0.21728900	-6.45842000	-0.85153800
H	2.51955400	-6.25267300	-0.37432000
H	4.57469400	-4.90573200	-0.19531000
H	2.55266400	-1.58066300	-1.99272500
H	0.50179700	-2.92259600	-2.17017200
H	2.34946700	5.99268000	1.43936800
H	0.94439400	3.36193900	-1.64130000
H	3.04360500	2.10642900	-1.53455500
H	4.44973100	4.71761300	1.56250100
H	5.83750700	2.94463200	0.88840700
H	5.78297600	2.69916700	-0.85595900
C	4.96492200	-2.29651100	-0.93386600
H	5.71482400	-1.12579700	1.56714600
H	3.29408300	0.69232700	1.23943100
H	4.49772200	2.03841600	2.96995000
H	5.77511300	0.81796300	3.01704900
C	3.95279400	-1.39342800	3.51201400
H	3.94218800	-2.85932200	1.90441700
H	2.70436100	-1.61792000	1.72550200
H	2.79203000	0.43032300	3.64397500
H	4.10454700	0.34150500	4.80428100
H	5.73176800	-2.85203600	-0.38091900

	H	5.37596700	-2.05966300	-1.91695300
	H	4.94701700	-1.73412700	3.82495500
	H	3.23224900	-1.92306600	4.14098300
	C	-3.70800400	-0.02571200	-0.03730800
	C	5.14566200	0.19024000	-0.75129800
	S	-2.05292400	-0.05427200	-0.32419300
	S	5.66429600	0.48659000	-2.33963200
Conf. 3	C	-0.43529800	2.69564500	3.69750700
	C	1.09152100	2.47041100	3.70440000
	C	1.72903600	2.50640500	2.29592800
	C	1.27808000	3.80899800	1.65085400
	C	-0.23745000	3.84512300	1.52695200
	C	-0.87528500	3.94936900	2.90559300
	H	-0.79736800	2.76749100	4.72658900
	H	-0.92478300	1.81949200	3.25658300
	H	1.31564400	1.51350400	4.18332400
	H	1.56570000	3.24366900	4.32011100
	H	1.40093500	1.65225900	1.69547200
	H	2.81710900	2.45241300	2.37726200
	H	1.59772000	4.63820000	2.30427200
	N	1.67263300	4.16585300	0.27965000
	N	-0.40386700	4.92470200	0.54135200
	H	-0.57628100	2.90769600	1.05962400
	H	-1.96549100	3.98344100	2.84565100
	H	-0.54167400	4.86488100	3.40549700
	C	-1.73646700	5.37246400	0.12515600
	C	-2.59557700	4.26780100	-0.46773000

C	-2.30702900	3.73277900	-1.72869600
C	-3.69214600	3.75559600	0.23125500
C	-4.46357700	2.72339600	-0.29986900
C	-4.16459600	2.17373900	-1.54965600
C	-3.08248700	2.70626700	-2.26005500
C	3.07391500	4.17647200	-0.12498100
C	3.56231500	2.82776400	-0.62060900
C	4.78687500	2.30835400	-0.20181000
C	5.24596900	1.08121500	-0.68456100
C	4.49220600	0.34034800	-1.59675200
C	3.26718000	0.87120700	-2.02195300
C	2.80917800	2.09240200	-1.54297800
C	4.97154400	-1.01180500	-2.10456000
C	4.16459600	-2.17373900	-1.54965600
C	-4.97154400	1.01180500	-2.10456000
C	-4.49220600	-0.34034800	-1.59675200
C	-5.24596900	-1.08121500	-0.68456100
C	-4.78687500	-2.30835400	-0.20181000
C	-3.56231500	-2.82776400	-0.62060900
C	-2.80917800	-2.09240200	-1.54297800
C	-3.26718000	-0.87120700	-2.02195300
C	4.46357700	-2.72339600	-0.29986900
C	3.08248700	-2.70626700	-2.26005500
C	2.30702900	-3.73277900	-1.72869600
C	2.59557700	-4.26780100	-0.46773000
C	3.69214600	-3.75559600	0.23125500
C	1.73646700	-5.37246400	0.12515600

N	0.40386700	-4.92470200	0.54135200
N	-1.67263300	-4.16585300	0.27965000
C	-1.27808000	-3.80899800	1.65085400
C	0.23745000	-3.84512300	1.52695200
C	-1.72903600	-2.50640500	2.29592800
C	0.87528500	-3.94936900	2.90559300
C	0.43529800	-2.69564500	3.69750700
H	-1.58380800	6.17495900	-0.59641500
H	-2.23215800	5.79919800	0.99959600
H	-1.47372600	4.12878900	-2.29781200
H	-3.95168400	4.16901800	1.20072800
H	-5.30628200	2.34076700	0.26677800
H	-2.84601900	2.31798600	-3.24532700
H	3.68527600	4.51017600	0.72067400
H	3.16962400	4.92416700	-0.91525300
H	5.38998600	2.86088300	0.51190800
H	6.20082200	0.69662000	-0.34090900
H	2.66209100	0.32107400	-2.73481900
H	1.85542300	2.48026300	-1.88272100
H	6.02408600	-1.13884000	-1.83886200
H	4.91487500	-1.02707000	-3.19705900
H	-4.91487500	1.02707000	-3.19705900
H	-6.02408600	1.13884000	-1.83886200
H	-6.20082200	-0.69662000	-0.34090900
H	-5.38998600	-2.86088300	0.51190800
H	-1.85542300	-2.48026300	-1.88272100
H	-2.66209100	-0.32107400	-2.73481900

	H	5.30628200	-2.34076700	0.26677800
	H	2.84601900	-2.31798600	-3.24532700
	H	1.47372600	-4.12878900	-2.29781200
	H	3.95168400	-4.16901800	1.20072800
	H	2.23215800	-5.79919800	0.99959600
	H	1.58380800	-6.17495900	-0.59641500
	C	-3.07391500	-4.17647200	-0.12498100
	H	-1.59772000	-4.63820000	2.30427200
	H	0.57628100	-2.90769600	1.05962400
	H	1.96549100	-3.98344100	2.84565100
	H	0.54167400	-4.86488100	3.40549700
	C	-1.09152100	-2.47041100	3.70440000
	H	-2.81710900	-2.45241300	2.37726200
	H	-1.40093500	-1.65225900	1.69547200
	H	0.92478300	-1.81949200	3.25658300
	H	0.79736800	-2.76749100	4.72658900
	H	-3.68527600	-4.51017600	0.72067400
	H	-3.16962400	-4.92416700	-0.91525300
	H	-1.56570000	-3.24366900	4.32011100
	H	-1.31564400	-1.51350400	4.18332400
	C	0.70768400	4.97583100	-0.25512300
	C	-0.70768400	-4.97583100	-0.25512300
	S	0.87528500	5.90363800	-1.66511100
	S	-0.87528500	-5.90363800	-1.66511100
Conf. 4	C	-3.15977200	5.18989700	-0.84510600
	C	-3.15940900	5.31843400	0.69267800
	C	-1.94489500	4.64331300	1.37234100

C	-0.70189500	5.19106200	0.68595700
C	-0.72206300	4.84759100	-0.79560100
C	-1.82735500	5.61843800	-1.50347700
H	-3.98033200	5.78130200	-1.25994300
H	-3.35992500	4.14713300	-1.11737200
H	-4.08421500	4.89454200	1.09297400
H	-3.16245900	6.38074400	0.96413500
H	-1.98863400	3.55520500	1.26160100
H	-1.94796500	4.86548700	2.44145000
H	-0.71038600	6.28890800	0.79318200
N	0.64742100	4.73280500	1.05618700
N	0.68921900	5.06938200	-1.14359400
H	-0.92921000	3.77188800	-0.90573400
H	-1.85508200	5.39378900	-2.57204600
H	-1.66575300	6.69604200	-1.39376900
C	1.16452200	4.99243100	-2.52450400
C	0.84085600	3.68516000	-3.22744500
C	1.41961500	2.47554100	-2.82090000
C	-0.03211200	3.66481900	-4.31599700
C	-0.31905400	2.47443500	-4.98296400
C	0.26290700	1.26825800	-4.58697500
C	1.13681000	1.28969800	-3.49139700
C	1.11802000	4.83159500	2.43854900
C	0.83048100	3.58924100	3.25853700
C	0.00000000	3.63285300	4.37758900
C	-0.27085200	2.47765700	5.11256300
C	0.29165800	1.25023800	4.75775500

C	1.14760000	1.21595300	3.64798500
C	1.40842700	2.36285400	2.90830800
C	0.00000000	0.00000000	5.56963400
C	-0.29165800	-1.25023800	4.75775500
C	0.00000000	0.00000000	-5.37924100
C	-0.26290700	-1.26825800	-4.58697500
C	-1.13681000	-1.28969800	-3.49139700
C	-1.41961500	-2.47554100	-2.82090000
C	-0.84085600	-3.68516000	-3.22744500
C	0.03211200	-3.66481900	-4.31599700
C	0.31905400	-2.47443500	-4.98296400
C	-1.14760000	-1.21595300	3.64798500
C	0.27085200	-2.47765700	5.11256300
C	0.00000000	-3.63285300	4.37758900
C	-0.83048100	-3.58924100	3.25853700
C	-1.40842700	-2.36285400	2.90830800
C	-1.11802000	-4.83159500	2.43854900
N	-0.64742100	-4.73280500	1.05618700
N	-0.68921900	-5.06938200	-1.14359400
C	0.72206300	-4.84759100	-0.79560100
C	0.70189500	-5.19106200	0.68595700
C	1.82735500	-5.61843800	-1.50347700
C	1.94489500	-4.64331300	1.37234100
C	3.15940900	-5.31843400	0.69267800
H	2.24218400	5.15470100	-2.48875300
H	0.72161900	5.82256800	-3.07997600
H	2.10505500	2.46655300	-1.98036600

H	-0.49151300	4.58838400	-4.65345200
H	-1.00114300	2.48755200	-5.82698400
H	1.60481200	0.36837500	-3.16166100
H	0.64819800	5.70533600	2.90257000
H	2.19292900	5.01433400	2.39600100
H	-0.45368000	4.57285700	4.67546700
H	-0.93603800	2.53404400	5.96834300
H	1.60305700	0.27711000	3.35242700
H	2.06338100	2.31043300	2.04515600
H	-0.84878900	0.20659900	6.23109400
H	0.84878900	-0.20659900	6.23109400
H	0.85343400	-0.18133000	-6.04229900
H	-0.85343400	0.18133000	-6.04229900
H	-1.60481200	-0.36837500	-3.16166100
H	-2.10505500	-2.46655300	-1.98036600
H	0.49151300	-4.58838400	-4.65345200
H	1.00114300	-2.48755200	-5.82698400
H	-1.60305700	-0.27711000	3.35242700
H	0.93603800	-2.53404400	5.96834300
H	0.45368000	-4.57285700	4.67546700
H	-2.06338100	-2.31043300	2.04515600
H	-2.19292900	-5.01433400	2.39600100
H	-0.64819800	-5.70533600	2.90257000
C	-1.16452200	-4.99243100	-2.52450400
H	0.92921000	-3.77188800	-0.90573400
H	0.71038600	-6.28890800	0.79318200
H	1.94796500	-4.86548700	2.44145000

H	1.98863400	-3.55520500	1.26160100
C	3.15977200	-5.18989700	-0.84510600
H	1.85508200	-5.39378900	-2.57204600
H	1.66575300	-6.69604200	-1.39376900
H	3.16245900	-6.38074400	0.96413500
H	4.08421500	-4.89454200	1.09297400
H	-2.24218400	-5.15470100	-2.48875300
H	-0.72161900	-5.82256800	-3.07997600
H	3.35992500	-4.14713300	-1.11737200
H	3.98033200	-5.78130200	-1.25994300
C	1.46256000	4.85375700	-0.03880300
C	-1.46256000	-4.85375700	-0.03880300
S	3.15687400	4.76030500	-0.01974500
S	-3.15687400	-4.76030500	-0.01974500

References

[1] Gawroński, J.; Kołbon, H.; Kwit, M.; Katrusiak, A., Designing Large Triangular Chiral Macrocycles: Efficient [3 + 3] Diamine−Dialdehyde Condensations Based on Conformational Bias. *J. Org. Chem*. **2000**, *65*, 5768

[2] Troć, A.; Gajewy, J.; Danikiewicz, W.; Kwit, M., Specific Noncovalent Association of Chiral Large-Ring Hexaimines: Ion Mobility Mass Spectrometry and PM7 Study. *Chem. Eur. J.* **2016**, *22*, 13258.

[3] Janiak, A.; Gajewy, J.; Szymkowiak, J.; Gierczyk, B.; Kwit, M. Specific non-covalent association of truncated, exo-functionalized triangular homochiral isotrianglimines through head-to-head, tail-to-tail, and honeycomb supramolecular motifs. *J. Org. Chem*. **2022**, *87*, 2356–2366.

[4] Gawroński, J.; Kwit, M.; Grajewski, J.; Gajewy, J.; Długokińska, A. Structural Constrains for the Formation of Macrocyclic Rhombimines. *Tetrahedron: Asymmetry* **2007**, *18*, 2632–2637.

[5] Kuhnert, N.; Strassnig, C.; Lopez-Periago, A. M. Synthesis of novel enantiomerically pure trianglimine and trianglamine macrocycles. *Tetrahedron: Asymmetry* **2002**, *13*, 123–128.

[6] Bennani, Y. L.; Hanessian, S. The asymmetric synthesis of α-substituted α-methyl and α-phenyl phosphonic acids: Design, carbanion geometry, reactivity and preparative aspects of chiral alkyl bicyclic phosphonamides. *Tetrahedron* **1996**, *52*, 13837–13866

[7] Szewczyk, M.; Stanek, F.; Bezłada, A.; Mlynarski, J. Zinc Acetate-Catalyzed Enantioselective Hydrosilylation of Ketones. *Adv. Synt. Catal.* **2015**, *357*, 3727–3731.

[8] Kylmälä, T.; Kuuloja, N.; Xu, Y.; Rissanen, K.; Franzén, R. Synthesis of Chlorinated Biphenyls by Suzuki Cross-Coupling Using Diamine or Diimine-Palladium Complexes. *Eur. J. Org. Chem.* **2008**, *23*, 4019–4024.

[9] Kowalczyk, R.; Sidorowicz, L.; Skarzewski, J. Asymmetric Henry reaction catalyzed by chiral secondary diamine-copper(II) complexes. *Tetrahedron: Asymmetry* **2008**, *19*, 2310–2315.

[10] Prusinowska, N.; Szymkowiak, J.; Kwit, M. Enantiopure tertiary urea and thiourea derivatives of trianglamine macrocycle – structural studies and metallogeling properties. *J. Org. Chem.* **2018**, *83*, 1167–1175.

[11] SCIGRESS 2.5, Fujitsu Ltd

[12] Becke, A. D. Density-functional Thermochemistry. III. The Role of Exact Exchange. *J. Chem. Phys.* **1993**, *98*, 5648.

[13] Lee, C.; Yang, W.; Parr, R. G. Development of the Colle-Salvetti Correlation-Energy Formula into a Functional of the Electron Density. *Phys. Rev. B* **1988**, *37*, 785.

[14] Becke, A. D. Density-Functional Exchange-Energy Approximation with Correct Asymptotic Behavior. *Phys. Rev. A* **1988**, *38*, 3098.

[15] Perdew, J. P. Density-Functional Approximation for the Correlation Energy of the Inhomogeneous Electron Gas. *Phys. Rev. B* **1986**, *33*, 8822.

[16] Scalmani, G.; Frisch, M. J. Continuous surface charge polarizable continuum models of solvation. I. General formalism. *J. Chem. Phys.* **2010**, *132*, 114110.

[17] Frisch, M. J.; Trucks, G. W.; Schlegel, H. B.; Scuseria, G. E.; Robb, M. A.; Cheeseman, J. R.; Scalmani, G.; Barone, V.; Mennucci, B.; Petersson, G. A.; Nakatsuji, H.; Caricato, M.; Li, X.; Hratchian, H. P.; Izmaylov, A. F.; Bloino, J.; Zheng, G.; Sonnenberg, J. L.; Hada, M.; Ehara, M.; Toyota, K.; Fukuda, R.; Hasegawa, J.; Ishida, M.; Nakajima, T.; Honda, Y.; Kitao, O.; Nakai, H.; Vreven, T.; Montgomery, J. A., Jr.; Peralta, J. E.; Ogliaro, F.; Bearpark, M. J.; Heyd, J.; Brothers, E. N.; Kudin, K. N.; Staroverov, V. N.; Kobayashi, R.; Normand, J.; Raghavachari, K.; Rendell, A. P.; Burant, J. C.; Iyengar, S. S.; Tomasi, J.; Cossi, M.; Rega, N.; Millam, N. J.; Klene, M.; Knox, J. E.; Cross, J. B.; Bakken, V.; Adamo, C.; Jaramillo, J.; Gomperts, R.; Stratmann, R. E.; Yazyev, O.; Austin, A. J.; Cammi, R.; Pomelli, C.; Ochterski, J. W.; Martin, R. L.; Morokuma, K.; Zakrzewski, V. G.; Voth, G. A.; Salvador, P.; Dannenberg, J. J.; Dapprich, S.; Daniels, A. D.; Farkas, Ö.; Foresman, J. B.; Ortiz, J. V.; Cioslowski, J.; Fox, D. J. Gaussian 09, revision D.01; Gaussian, Inc.: Wallingford, CT, 2009.

[18] Kwit, M.; Gawronski, J.; Boyd, D. R.; Sharma, N. D.; Kaik, M. Circular Dichroism, Optical Rotation and Absolute Configuration of 2-Cyclohexenone-*cis*-diol Type Phenol Metabolites: Redefining the Role of Substituents and 2-Cyclohexenone Conformation in Electronic Circular Dichroism Spectra. *Org. Biomol. Chem.* **2010**, *8*, 5635 and literature cited therein.

[19] Yanai, T.; Tew, D.; Handy, N. A new hybrid exchange–correlation functional using the Coulomb-attenuating method (CAM-B3LYP). *Chem. Phys. Lett.* **2004**, *393*, 51.

[20] a) Zhao, Y.; Truhlar, D. G. The M06 suite of density functionals for main group thermochemistry, thermochemical kinetics, noncovalent interactions, excited states, and transition elements: two new functionals and systematic testing of four M06-class functionals and 12 other functionals. *Theor. Chem. Acc*. **2008**, 120, 215.

[21] Jacquemin, D.; Perpète, E. A.; Ciofini, I.; Adamo, C.; Valero, R.; Zhao, Y.; Truhlar, D. G. On the Performances of the M06 Family of Density Functionals for Electronic Excitation Energies. *J. Chem. Theor*. **2010**, *6*, 2071.

[22] Harada, N.; Stephens, P. ECD cotton effect approximated by the Gaussian curve and other methods. *Chirality* **2010**, *22*, 229.

[23] CrysAlisPro 1.171.40.57a, Rigaku Oxford Diffraction, **2019**.

[24] Sheldrick, G. M. SHELXT – Integrated space-group and crystal-structure determination. *Acta Crystallogr.* **2015**, *A71*, 3–8.

[25] Sheldrick, G. M. Crystal structure refinement with SHELXL. *Acta Crystallogr.* **2015**, *C71*, 3–8.

[26] Spek, A. L. Structure validation in chemical crystallography. *Acta Crystallogr.* **2009**, *D65*, 148–155.

[27] Efimenko, Z. M.; Eliseeva, A. A.; Ivanov, D. M.; Galmés, B.; Frontera, A.; Bokach, N. A.; Kukushkin, V. Y. Bifurcated μ2-I···(N,O) Halogen Bonding: The Case of (Nitrosoguanidinate)Ni^{II} Cocrystals with Iodine(I)-Based σ-Hole Donors. *Crystal Growth & Design* **2021**, *21*, 588–596.

[28] Parsons, S.; Flack, H. D.; Wagner, T. Use of intensity quotients and differences in absolute structure refinement. *Acta Crystallogr.* **2013**, *B69*, 249–259.

[29] (a) Farrugia, L. J. WinGX and ORTEP-3 for Windows: An Update. *J. Appl. Cryst.* **2012**, *45*, 849–854. (b) Macrae, C. F.; Sovago, I.; Cottrell, S. J.; Galek, P. T. A.; McCabe, P.; Pidcock, E.; Platings, M.; Shields, G. P.; Stevens, J. S.; Towler, M.; Wood, P. A. Mercury 4.2.0: from visualization to analysis, design and prediction. *J. Appl. Cryst.* **2020**, *45*, 226–235.

[30] (a) Spackman, M. A.; Jayatilaka, D. Hirshfeld surface analysis. *CrystEngComm* **2009**, *11* ,19–32. (b) Spackman, P. R.; Turner, M. J.; McKinnon, J. J.; Wolff, S. K.; Grimwood, D. J.; Jayatilaka, D.; Spackman, M. A. CrystalExplorer: a program for Hirshfeld surface analysis, visualization and quantitative analysis of molecular crystals. *J. Appl. Cryst.* **2021**, *54*, 1006–1011.

[31] Gawroński, J.; Gawrońska, K.; Grajewski, J.; Kwit, M.; Plutecka, A.; Rychlewska, U. Trianglamines – Readily Prepared, Conformationally Flexible, Inclusion-Forming Chiral Hexaamines. *Chem. Eur. J.* **2006**, *12*, 1807–18147.

[32] Bondi, A. Van der Waals Volumes and Radii. *The Journal of Physical Chemistry* **1964**, *68*, 441–451.
